# Association between Arsenic Level, Gene Expression in Asian Population, and In Vitro Carcinogenic Bladder Tumor

**DOI:** 10.1155/2022/3459855

**Published:** 2022-01-07

**Authors:** Sonalika Singhal, Nathan A. Ruprecht, Donald Sens, Kouhyar Tavakolian, Kevin L. Gardner, Sandeep K. Singhal

**Affiliations:** ^1^Department of Pathology, School of Medicine and Health Sciences, University of North Dakota, ND, USA; ^2^Department of Biomedical Engineering, School of Electrical Engineering and Computer Science, University of North Dakota, ND, USA; ^3^Department of Pathology and Cell Biology, Columbia University Irvine Medical Center, New York, NY, USA

## Abstract

The IARC classified arsenic (As) as “carcinogenic to humans.” Despite the health consequences of arsenic exposure, there is no molecular signature available yet that can predict when exposure may lead to the development of disease. To understand the molecular processes underlying arsenic exposure and the risk of disease development, this study investigated the functional relationship between high arsenic exposure and disease risk using gene expression derived from human exposure. In this study, a three step analysis was employed: (1) the gene expression profiles obtained from two diverse arsenic-exposed Asian populations were utilized to identify differentially expressed genes associated with arsenic exposure in human subjects, (2) the gene expression profiles induced by arsenic exposure in four different myeloma cancer cell lines were used to define common genes and pathways altered by arsenic exposure, and (3) the genetic profiles of two publicly available human bladder cancer studies were used to test the significance of the common association of genes, identified in step 1 and step 2, to develop and validate a predictive model of primary bladder cancer risk associated with arsenic exposure. Our analysis shows that arsenic exposure to humans is mainly associated with organismal injury and abnormalities, immunological disease, inflammatory disease, gastrointestinal disease, and increased rates of a wide variety of cancers. In addition, arsenic exerts its toxicity by generating reactive oxygen species (ROS) and increasing ROS production causing the imbalance that leads to cell and tissue damage (oxidative stress). Oxidative stress activates inflammatory pathways leading to transformation of a normal cell to tumor cell specifically; there is significant evidence of the advancing changes in oxidative/nitrative stress during the progression of bladder cancer. Therefore, we examined the relation of differentially expressed genes due to exposure of arsenic in human and bladder cancer and developed a bladder cancer risk prediction model. In this study, integrin-linked kinase (ILK) was one of the most significant pathways identified between both arsenic exposed population which plays a key role in eliciting a protective response to oxidative damage in epidermal cells. On the other hand, several studies showed that arsenic trioxide (ATO) is useful for anticancer therapy although the mechanisms underlying its paradoxical effects are still not well understood. ATO has shown remarkable efficacy for the treatment of multiple myeloma; therefore, it will be helpful to understand the underlying cancer biology by which ATO exerts its inhibitory effect on the myeloma cells. Our study found that MAPK is one of the most active network between arsenic gene and ATO cell line which is involved in indicative of oxidative/nitrosative damage and well associated with the development of bladder cancer. The study identified a unique set of 147 genes associated with arsenic exposure and linked to molecular mechanisms of cancer. The risk prediction model shows the highest prediction ability for recurrent bladder tumors based on a very small subset (NKIRAS2, AKTIP, and HLA-DQA1) of the 147 genes resulting in AUC of 0.94 (95% CI: 0.744-0.995) and 0.75 (95% CI: 0.343-0.933) on training and validation data, respectively.

## 1. Introduction

Arsenic (As) is a ubiquitous element in the environment, ranked the 20th most abundant element on earth. The toxic impact of arsenic on human health has been documented in numerous studies leading to arsenic identification as a known carcinogen by the International Agency Research on Carcinogens (IARC), the National Toxicity Program (NTP), and the United States Environmental Protection Agency (EPA) [1, 2]. In addition to cancer, long-term exposure to arsenic has been associated with developmental effects, cardiovascular disease, neurotoxicity, and diabetes (WHO, https://www.who.int/news-room/fact-sheets/detail/As). Typically, arsenic would only be found in background levels in soil and groundwater. However, high levels of arsenic accumulate in these medians from anthropogenic activities such as indiscriminate waste disposal from mining, milling, and smelting of ores [3], raw and spent oil shale [4], and coal fly ash amendments [5]. The usage pattern in the 1960s for arsenic compounds in the United States was 77% pesticides, 18% as glass, and 4% industrial chemicals. The past use of arsenic as a pesticide in agriculture is exemplified by New Jersey, where between 1900 and 1960, it is estimated that approximately 15 million pounds of arsenic were applied to New Jersey soils alone [6]. Leaching of arsenic from soils into the water supply has now resulted in the significant contamination of drinking water in many areas of the United States and the world. This past usage of arsenic in anthropogenic activities has now resulted in exposure to arsenic being a global public health problem [7–9]. This is illustrated by the fact that over 120 million people are affected by arsenic exposure, many of which reside in Bangladesh and India [8, 10]. A recent study has modeled the role of atmospheric exposure to arsenic as being additive to overall exposure levels [11]. Despite the health consequences of arsenic exposure, there is no molecular signature that might predict the risk of developing cancer or other diseases following exposure to arsenic.

On the other hand, the use of arsenicals as therapeutic agents in medicine is very well known dating back more than 2400 years to ancient Greece and Rome [12]. In the 19th century, potassium arsenite was used to treat different types of disease [13] including diabetes, psoriasis, syphilis, skin ulcers, and joint diseases. More recently, phase I/II trials have been conducted in heavily pretreated patients with relapsed or refractory multiple myeloma shows arsenic trioxide (ATO) is the most active, single agent in acute promyelocytic leukemia (multiple myeloma: types of blood cancers) [14]. Another study suggested that ATO can be used as an effective alternative therapeutic for the treatment of retinoblastoma which is the most common intraocular cancer in children [15]. The study shows an antitumor activity of arsenic which mainly targets multiple pathways in malignant cells, resulting in the promotion of differentiation or in the induction of apoptosis, which would be very helpful to understand the molecular mechanism of arsenic-exposed cancer biology as a reverse engineering approach.

Biomarkers are classified based on exposure, effect, and susceptibility [16]. For arsenic, biomarkers of exposure have received the greatest attention and success in defining individual exposures [17]. Human susceptibility to arsenic, especially as it applies to predicting disease states, is probably the least studied area of biomarkers. A few biomarkers of interest attracting study include clastogenicity in peripheral lymphocytes, micronuclei in oral mucosa and bladder cells, and induction of heme oxygenase [16, 18, 19]. The goal of the present study was to identify differentially expressed genes in arsenic exposed humans and determine if a molecular signature could be developed that would stratify and predict the risk of urothelial cancer for those with known exposure to arsenic. Urothelial cancer, which is the most common type of bladder cancer, was chosen as an initial proof of principle since epidemiological, and other evidence is strong for the link between arsenic and the development of urothelial cancer, and there are publicly available databases for data mining [7, 20–24]. A theme of such studies shows a strong association at more extreme levels (>150 *μ*g/L) whereas there is uncertainty of health effects that may develop below this threshold. Suggested mechanisms for arsenic carcinogenesis include oxidative damage, epigenetic effects, and interference with DNA repair. In addition, the development of bladder cancer is known to have a strong association with environmental exposures from mentioned anthropogenic activities [25]. Overproduction of reactive oxygen species (ROS) due to arsenic exposure primarily follows direct toxicity or the metabolic processes of arsenic products. Inhibiting succinic dehydrogenase activity in mitochondrial complexes I and III in electron transport chain produces superoxide radical anion, while monomethylarsonic acid (MMA) and dimethylarsinic acid (DMA) will form radicals in the cell and specifically the endoplasmic reticulum [26, 27]. Since inorganic arsenic compounds tend to be more toxic than organic, ATO is of interest for its global concern along with its involvement in oxidative and nitrosative stress properties. Translational damage from reactive species can regulate MAPK family or induce extended states of inflammation, genetic, and epigenetic mechanisms such as these are indicative of oxidative/nitrosative damage and well associated with the development of bladder cancer [28–30]. ILK signaling and neuroinflammation signaling pathway were the most frequent pathways affected by the exposure of arsenic, and both of them are highly associated with oxidative stress. Oxidative stress and neuroinflammation could potentiate each other to promote progression of mental disorders [31], whereas ILK plays a complex roles in the modulation of oxidant species production [32].

The strategy used in the present study involved three steps. The first step was a blood cell gene expression analysis of two diverse human populations with known levels of exposure to arsenic. One population was stratified to low and high exposure, and the second population to low, medium, and high exposure with correlation to human global gene expression. After identifying statistically significant genes unique to the mentioned test conditions, we found that cancer was the most significant disease and lipid metabolism (which is considered as a major metabolic pathway involved in the progression of cancer) was most significant molecular and cellular functions associated with genes differentially expressed due to different levels of arsenic. Therefore, the next stage was to compare it with data from four independent myeloma cell lines that had been treated with As trioxide (ATO) to understand the molecular mechanism of cancer. Many of the genes that were up- and downregulated due to arsenic exposure are associated with cancer biology. There gene lists were then subjected to enrichment analysis to identify statistically significant pathways and further scrutinized for functional relevance. The third step was to develop a model by examining the ability of the most significant genes to predict the progression and possible development of bladder cancer using publicly available patient biopsy samples. Using this approach, we developed a robust regression model of three significant probes and corresponding gene results with AUC of 0.94 (95% CI: 0.744-0.995) and 0.75 AUC (95% CI: 0.343-0.933) on the training and validation data, respectively. The most significant pathway identified is integrin-linked kinase (ILK) which plays a key role in eliciting a protective response to oxidative damage in epidermal cells [32].

## 2. Materials and Methods

### 2.1. Data

Two publicly available gene expression datasets of previously conducted experiments were accessed from two independent populations. The set from Bangladesh (Gene Expression Omnibus GEO ID: GSE57711) had 29 individuals; 16 were males, and 13 were females. The second dataset was from Pakistan (GSE110852 ID) and had 57 individuals composed of 31 males and 26 females. In this report, the set from Bangladesh is denoted as Data1 and that from Pakistan is Data2 and remains unchanged from their original, respective studies. Data1 samples were part of a clinical trial in June 2011 [33]. For these samples, “low” exposure levels correlate to a range of 50-200 *μ*g/L, whereas “high” levels correlate to a range from 232 to 1000 *μ*g/L (there were no samples collected from patients exposed in the range of 201-231 *μ*g/L). Data2 samples were from two main districts of rural Pakistan, Lahore, and Kasur. The study is aimed at investigating the blood transcriptome profile among the exposed samples to correlate gene expression to exposure levels of As [34]. Urine sampling was used to define levels of arsenic exposure, with “low” being 0-50 *μ*g/g creatinine, “medium” as 51-100 *μ*g/g creatinine, and “high” as >101 *μ*g/g creatinine. The general characteristics of both data sets are detailed in Table 1. The results from 4 multiple myeloma cell lines treated with ATO were obtained from the GEO database, series GSE14519 [35]. These cell lines U266, MM1S, KMS11, and 8226S were exposed to ATO for 6 hr, 28 hr, and 48 hr before analysis. Gene expression profiling was used to determine differences in cell line response to ATO. This study was used as a reference point in the present study since it documents the effects of arsenic compounds on gene expression at different exposure levels.

The two databases of previously conducted experiments containing biopsies of bladder cancer were obtained from GEO, GSE13507 [36, 37] and GSE3167 [38]. The GSE13507 contained 165 samples for primary bladder cancer, 23 recurrent nonmuscle invasive tumor tissues, 58 normal-looking bladder mucosa surrounding cancer, and 10 normal bladder mucosa. This dataset was originally used in microarray analysis for the identification of genes with prognostic significance. GSE3167 contained 28 samples of superficial bladder tumors, 13 samples of muscle-invasive carcinomas, and 9 normal samples. This dataset was previously used for gene expression signatures among various stages of carcinomas. These 2 datasets were used in the present study to obtain a prognostic gene-based prediction for bladder cancer.

The QC report of the datasets was examined, and only qualified samples were included. Since the data was generated using different platforms (such as Affymetrix and Agilent), no single approach would work on those datasets. Therefore, the raw datasets were preprocessed to extract expression using the same approach provided within publication of study, for example, the Affymetrix package in R used for GSE57711 while an in-house QC pipeline (http://github.com/BiGCAT-UM/arrayQC_Module) for data GSE110852. Before applying any statistical test, the distribution of each data tested and transformed into a normal distribution using logarithm and pareto scaling (mean-centered and divided by the square root of the standard deviation of each variable) transformation. Entire gene expression analyses were performed using R Bioconductor (https://www.r-project.org). The analysis was performed using Bioconductor packages such as Stat, Dplyr (https://cran.r-project.org/web/packages/dplyr/index.html), ggplot2 (https://cran.r-project.org/web/packages/ggplot2/index.html), randomForest (https://cran.r-project.org/web/packages/randomForest/), e1071 (https://cran.r-project.org/web/packages/e1071/index .html), and pvclust (https://cran.r-project.org/web/packages/pvclust/index.html).

Sample size calculation was performed to determine if the power is sufficient to detect a biological effect. The number of samples with categories of arsenic exposure is (1) GSE57711 (low, *n* = 15/high, *n* = 14) and (2) GSE110852 (low, *n* = 18/medium, *n* = 19/high, *n* = 20). The power analysis of GSE57711 data shows that a minimum of 14 samples are required to achieve the 80% power with minimum genes 11626 (per-sample), acceptable number of false positives 5, fold change differences desired of 2, standard deviation of 0.6, and alpha (per-gene) 0.00043. These sample size computations assume that the expression of each gene is normally distributed on the log scale and believe that gene expression measurements are independent [39].

### 2.2. Machine Learning (ML) Methods

The ML machine learning-based classification approach was used to understand the population characteristics; partial least squares discriminant analysis (PLS-DA) was used in which the properties of PLS regression (PLS-R) are combined with the discrimination power of the classification technique [40]. The goal here is to determine the distribution of samples and visualize how the global gene expression profile scattered in different groups (sex and arsenic exposure) and which features best describe the differences between them.

A Random Forest (RF) was implemented to understand further which genes correlate to classification between sex and categories of arsenic concentration [41], specifically, if the gene expressions of a combination of genes can correctly differentiate between categories. This classification provides insight into which genes are expressed differentially depending on an individual's condition, such as sex and/or exposure.

The correction between the samples was calculated using the Pearson's coefficient [42, 43] and the heat map method [44]. These were used to plot the correlation coefficient values to find the most correlated samples. Hierarchical clustering, an unsupervised learning approach, was then employed to calculate a dendrogram to determine the closest related samples. A hierarchical clustering an unsupervised learning approach was then employed to calculate a dendrogram to determine the closest related samples [45, 46].

### 2.3. Statistical Methods

The statistical significance of each gene within each dataset was calculated by running *t*-tests [47] between the categories for conditions (male/female, low/high, low/medium, medium/high As exposure levels). Along with *t*-tests for pairwise comparison, one-way ANOVA [48] (Analysis of Variance) with post hoc Tukey HSD (Honestly Significant Difference) [49] tests was performed for comparing multiple groups, i.e., level of As together with sex effect. The gene is filtered based on *p* value with threshold 0.05 without statistical methods that control the false discovery rate (fdr) to avoid the loss of a large number of genes at initial level without further evaluation.

### 2.4. Pathway Enrichment Analysis

The Ingenuity Pathway Analysis (IPA) (version 2020; Ingenuity Systems; QIAGEN) [50] was used for pathway enrichment and functional analysis of the significant genes among the human arsenic exposed samples. The KEGG pathways [51], PFAM protein domains [52], Uniport keywords [53], biological processes, molecular functions, cellular components, and Reactome pathways [54] were used to find associated pathways with statistically significant genes. In addition, we further built a network of gene-gene associations using STRING [55] that leads us to the gene subsets corresponding to a part of a particular function or pathway.

### 2.5. Prediction Model

Classical univariate AUROC [56] analysis was performed to find out the prediction ability of each gene independently using logistic regression [57] method with 10-fold cross-validation approach; next, all the genes were ranked according to this AUC value, and all possible combinations of genes were tested by added one gene at a time to the logistics model of top gene in a multivariate. The final model was selected based on the highest AUC (with 95% confidence intervals CI) among all possible combinations of the selected genes, and performance was tested using the Monte Carlo cross-validation (MCCV).

The flow of the study is demonstrated by two charts (Supplementary Figure 1A and 1B). The first chart shows the flow of the analytical approaches used in parallel to analyze the Data1 and Data2 to find differently expressed genes, those specific to sex, those specific to arsenic exposure, and specific to both sex and arsenic exposure. It shows the differentially expressed genes and pathways with overlapping significance following statistical methods described and provided for clarity. The second supplementary flow chart describes and shows the flow of the next stage of this study in taking the statistically significant genes to find commonality with the four cancer cell lines and creating a bladder cancer risk predictor with high accuracy.

## 3. Results

### 3.1. Global Gene Expression Analysis of Two As Exposed Sets of Human Data

PLS-DA and Pearson correlation were performed together with patient sex and arsenic exposed level to determine the similarities and differences in global gene expression patterns between the arsenic exposed cohorts.

**Figure 1:**
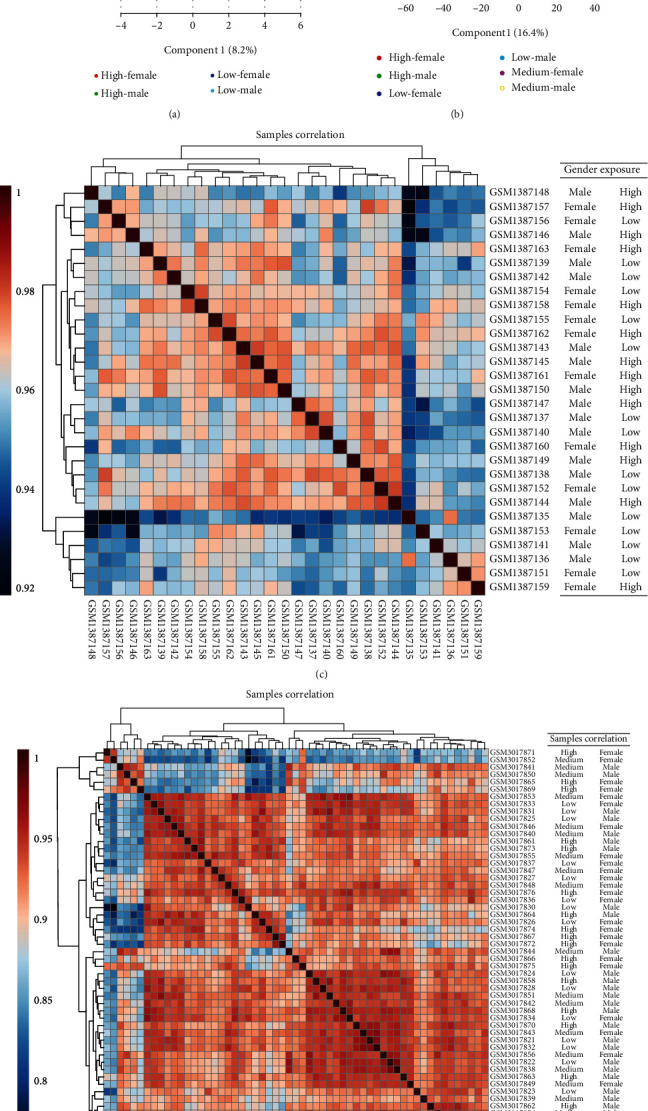
Sample distribution of gene expression profiles: (a) Data1; (B) Data2. The partial least squares discriminant analysis (PLS-DA) plot showing clusters of samples based on similarity. The first two components of PLS-DA (PC1 and PC2) of gene expression profile and overall variance between the groups are displayed. Each dot represents a sample color coded by both gender and level of arsenic exposure level. Pearson correlations were calculated between each sample of total population, and correlation coefficient values were shown by heat map of Data1 (c) and Data2 (d). The color-coding bar proves the value of correlation-coefficient. The dendrogram represents the relation between the samples created by using hierarchical clustering approach.

For the Data1 samples, PLS-DA analysis demonstrated a clear separation between high arsenic exposed females compared to low arsenic exposed males (Figure 1(a)). The global gene expression profile distribution moves from low to high for samples of high exposure of arsenic in females (HF), low exposure of arsenic in females (LF), high exposure of As in males (HM), and low exposure of As in male (LM). The analysis did not demonstrate an explicit separation between low exposure females and high exposed males. The heat map of Pearson correlation coefficients and the dendrogram among the samples show variability within Data1 (Figure 1(c)) with correlation value 0.92 to 1. There was no clear separation based solely upon arsenic exposure or sex. This provides evidence that sex and arsenic exposure has no bias impact on gene expression profile of Data1. An identical analysis of the samples in Data2 demonstrates that the distribution of global gene expression is almost the same for high and low exposure between males and females, where females with high arsenic exposure have the lowest expression profile when compared to low As exposed males (Figure 1(b)). The global gene expression profile moves low to high starting lowest in high exposure of arsenic in female (HF), to high exposure of As in male (HM), then low exposure of As in female (LF), and low exposure of As in male (LM); however, the medium exposure of As is mixed with low As exposure. The heat map of Pearson correlation coefficients and the dendrogram among the samples show variability within Data2 (Figure 1(d)) with correlation value 0.75 to 1, and there is no clear separation between either based upon arsenic exposure or sex. This indicates that this data has no bias impact for both the factors.

**Figure 2:**
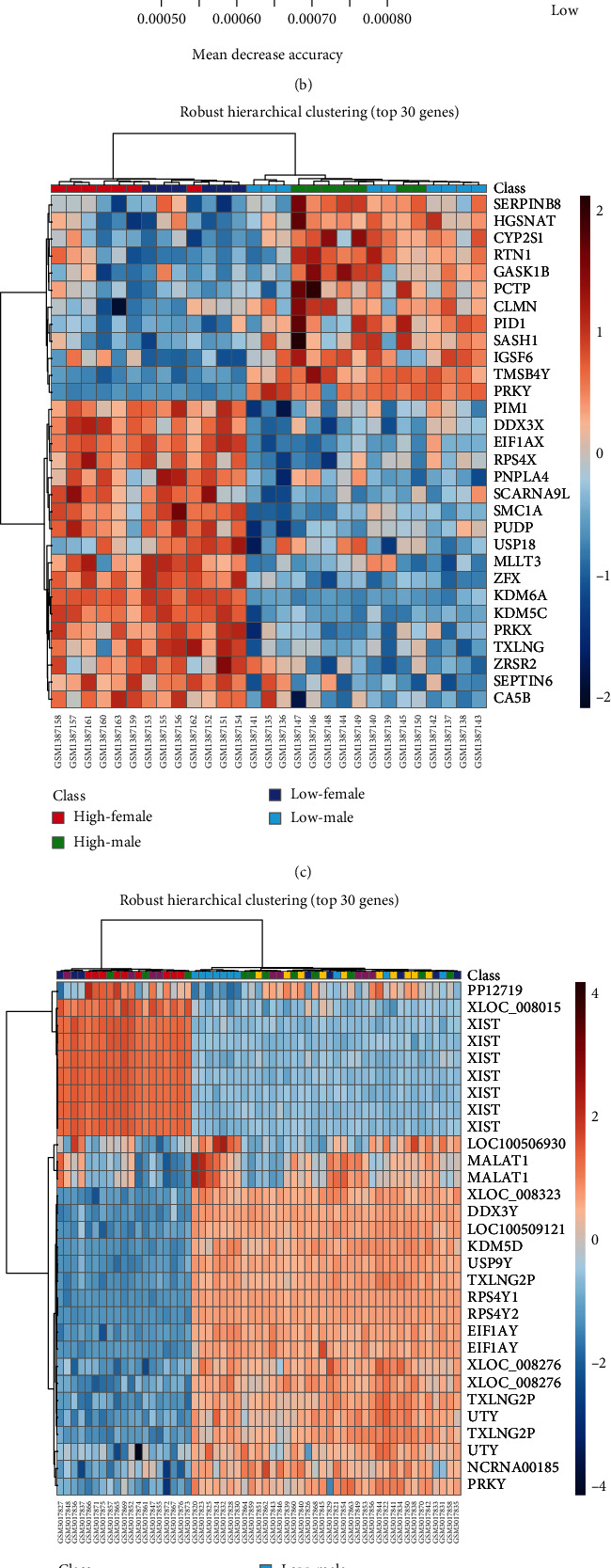
Global gene expression profile analysis. (a, b) A list of top 15 genes are displayed with mean decrease in accuracy value (*X*-axis) calculated using Random Forest Approach for Data1 and Data2, respectively. The small box (right side) and color-coding bar represent the expression value (from low to high) of each gene in different conditions. (c, d) Top 30 genes identified by robust hierarchical clustering approach for Data1 and Data2, respectively. The heat map represents the gene expression value across different samples. The top line on the *x*-axis, each box represents one sample. Two color-bar codes provide the gene expression value and condition of the sample (ultraright). (e, f) Venn diagram, overlap of most significantly differentially expressed genes and significantly associated pathways (*p* value ≤ 0.05) between both cohorts Data1 (blue) and Data2 (red), respectively. The complete lists of significant genes are provided in supplementary table-2, and significant pathways are in supplementary table-3.

As described in our methodology and shown via supplementary figures, the most significant, common genes were screened across the two datasets to distinguish difference among all four scenarios of sex and arsenic exposure in Data1 (Figure 2(a)) and all six scenarios in Data2 (Figure 2(b)). This analysis showed several probes for the genes XIST (X inactive specific transcript), MALAT1 (metastasis-associated lung adenocarcinoma transcript 1), XLOC_008276 (long intergenic nonprotein coding RNA 278), USP9Y (Ubiquitin Specific Peptidase 9 Y-Linked), SEPTIN6 (Septin 6), DDX3X (DEAD-Box Helicase 3 X-Linked), KDM6A (Lysine Demethylase 6A), and ZFX (Zinc Finger Protein X-Linked) as most significant in the RF as well as in the hierarchical clustering approach (Figures 2(a)–2(d)).

In addition to the advanced machine learning approach, the ANOVA test with post hoc test was used to compare different arsenic exposure levels together with sex. This identified 476 probes (corresponding to 476 unique genes symbols) (Supp. Table 2) that were differently expressed in Data1 and 529 probes (corresponding to 439 unique genes symbols) (Supp. Table 2) that were differently expressed in Data2 (*p* value < 0.05). An IPA analysis was performed to identify the functional relevance of these genes in terms of pathway association, resulting in identifying a total of 145 and 36 significant pathways for Data1 and Data2, respectively (Supp. Table 3). A total of 7 common genes and 6 common pathways were found to overlap for the two populations (Figures 2(e) and 2(f)). The common genes identified were CNR2 (Cannabinoid Receptor 2), GPR34 (G Protein-Coupled Receptor 34), DDHD2 (DDHD Domain Containing 2), BACE2 (Beta-Secretase 2), PRKY (Protein Kinase Y-linked Pseudogene), CST2 (Cystatin SA), and PTGDR2 (Prostaglandin D2 Receptor 2) (Figures 2(e) and 2(f)).

Organismal injury and abnormalities were the only disease common between two datasets due to combined change of arsenic level and sex, and cell morphology, cell death and survival, and cell-to-cell signaling and interaction were common molecular and cellular functions (Figures 2(g) and 2(h), Table 1(b)).

### 3.2. Sex-Specific Gene Expression

**Figure 3:**
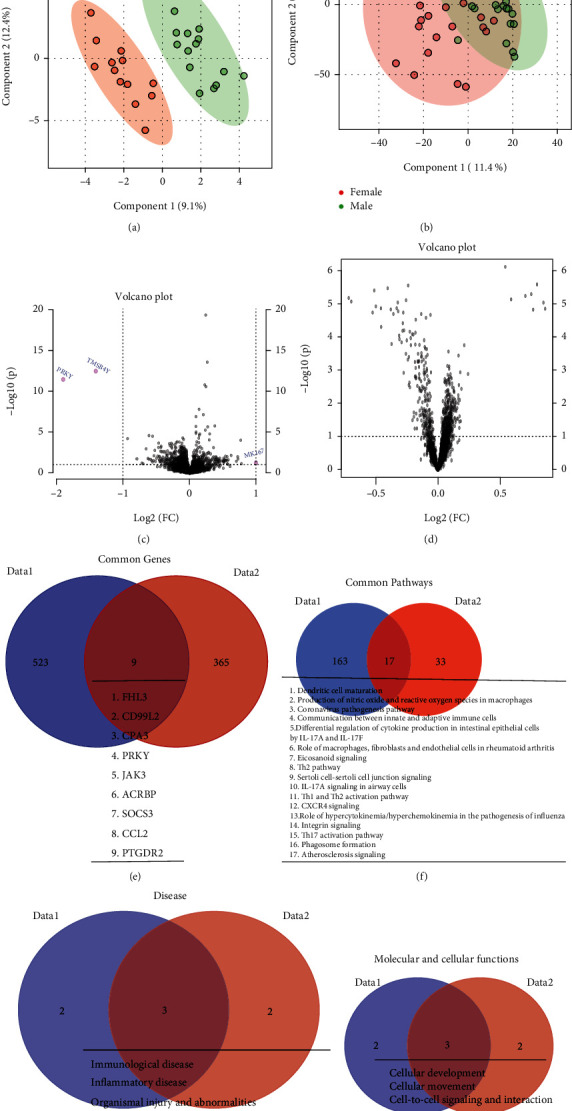
Sex-dependent genetic variations. (a, b) PLS-DA plot showing the gene expression profile distribution of each sample between females (red) and males (green) for Data1 and Data2, respectively. The first two components of PLS-DA (PC1 and PC2) of gene expression profile and overall variance between the groups are displayed. Each dot represents a sample color coded by gender. (c, d) Volcano plot displays the log2 fold change and -log10 (*p* value) of gene expression differentiating due to gender effect for Data1 and Data2, respectively. Genes with higher than two-fold (*p* value ≤ 0.05) are highlighted in red. (e, f) Venn diagram, overlap of most significantly differentially expressed genes and significantly associated pathways (*p* value ≤ 0.05) between both cohorts Data1 (blue) and Data2 (red), respectively. The names of common genes are provided in a table (underneath). The complete lists of significant genes are provided in supplementary table-4, and significant pathways are in supplementary table-5.

PLS-DA analysis was performed on Data1 and Data2 to determine only sex-based changes in overall gene expression profiles. The PLS-DA plot for Data1 demonstrated a clear separation among the 17 male and 13 female samples, with females having an overall lower gene expression profile (Figure 3(a)). The analysis of Data2 showed the same pattern among the 31 males and 26 females, with a relatively low separation because of the high variability of female gene expression profiles (Figure 3(b)). A *t*-test was used to identify the significant differentially expressed genes between the sexes (*p* ≤ 0.05). This identified 532 and 373 genes for Data1 and Data2, respectively (Supp. Table 4). Volcano plots were generated for both data sets to demonstrate high statistical significance as determined by *p* value together with a fold change difference of 2 (Figures 3(c) and 3(d)). This analysis identified 3 biologically significant genes for Data1, and no significant genes for Data2. Of the 3 genes identified, two were downregulated, PRKY (protein kinase Y-linked-pseudogene) and TMSB4Y (thymosin beta 4 Y-linked), while the one upregulated gene was KI67 (a marker of proliferation, Ki-67).

The determining overlapping significant genes (*p* value < 0.05) identified 9 genes that were common among the 532 differentially expressed genes of Data1 and the 373 genes of Data2 (Figure 3(e)). These 9 genes were FHL3 (Four And A Half LIM Domains 3), CD99L2 (CD99 Molecule Like 2), CPA3 (Carboxypeptidase A3), PRKY (protein kinase Y-linked-pseudogene), JAK3 (Janus Kinase 3), ACRBP (Adrenoceptor Beta 3), SOCS3 (Suppressor Of Cytokine Signaling 3), CCL2 (C-C Motif Chemokine Ligand 2), and PTGDR2 (prostaglandin D2 receptor 2). The genes for PRKY and PTGDR2 also appeared in the comparisons for unique overlapping genes in the global population analysis performed in the previous section. An IPA pathway analysis demonstrated that 180 pathways were significantly associated in Data1 as compared to 50 pathways in Data2 (Figures 3(c) and 3(d), Supp. Table 5, Figure 3(f)). A comparison of these pathways demonstrated that there were 17 overlapping common pathways with respect to sex in the 2 populations.

Immunological disease, inflammatory disease, organismal injury, and abnormalities were the common disease, and cellular development, cellular movement, cell-to-cell signaling, and interaction were the common molecular and cellular functions (Table 1(b)) associated due the sex difference in both the datasets.

### 3.3. As-Specific Human Gene Expression

**Figure 4:**
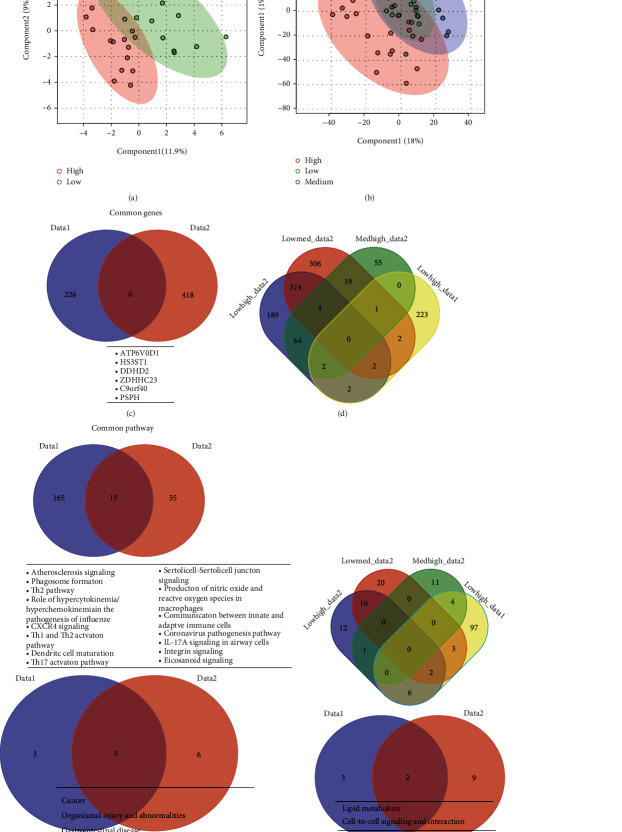
Arsenic-level dependent genetic variations. (a, b) PLS-DA plot showing the gene expression profile distribution of each sample between different levels of arsenic exposure (red, high; purple, medium; and green, low) for Data1 and Data2, respectively. The first two components of PLS-DA (PC1 and PC2) of gene expression profile and overall variance between the groups are displayed. Each dot represents a sample color coded by As-level. (c) Venn diagram, overlap of most significantly differentially expressed genes when comparing the low vs. high for Data1 and low-medium-high (*p* value ≤ 0.05) for Data2 and represented by blue (Data1) and red (Data2), respectively. The names of common genes are provided in a table (underneath). (d) Venn diagram, overlap of most significantly differentially expressed gene when compared the low vs. high for Data1 and pairwise comparison between low-medium-high with (*p* value ≤ 0.05) and represented as Data1 (yellow) and Data2 (blue, low vs. high; red, low vs. medium; green, medium vs. high), respectively. (e, f) Venn diagram, associated pathways for the genes identified in figure (c, d) with same classification and color coding described. The names of common genes are provided in a table (underneath). The complete lists of significant genes are provided in supplementary table-6 and table-8, and significant pathways are in supplementary table-7 and table-9.

An identical analysis used above for sex was employed to compare the differences in gene expression due to arsenic exposure for Data1 and Data2. PLS-DA demonstrated a prominent division of high and low arsenic exposure for those in Data1, where high exposure showed an overall low gene expression profile (Figure 4(a)). The analysis of Data2 showed a substantial division between the high versus medium levels of arsenic exposure but no separation between medium and low level of exposure (Figure 4(b)). The differentially expressed genes were identified for Data1 using the *t*-test with significance at *p* < 0.05. For Data2, the differentially expressed genes were identified using paired *t*-test and ANOVA between all three groups with a threshold level of *p* < 0.05.

This analysis identified 232 genes from Data1 and 424 genes from Data2 that were significant with 6 genes being common between the datasets (Supp. Table 6, Figure 4(c)). These 6 genes were ATP6V0D1 (ATPase H+ Transporting V0 Subunit D1), HS3ST1 (Heparan Sulfate-Glucosamine 3-Sulfotransferase 1), DDHD2 (DDHD Domain Containing 2), ZDHHC23 (Zinc Finger DHHC-Type Palmitoyltransferase 23), C9orf40 (chromosome 9 Open Reading Frame 4), and PSPH (Phosphoserine Phosphatase). All common genes were between all possible pairwise combinations of different arsenic levels of Data2 together with those of Data1 (Supp. Table 8, Figure 4(d)). The total number of significant pathways was 180 and 50, for Data1 and Data2, with 15 pathways common between them (Supp. Table 7, Figure 4(e)). The interaction of significant pathways identified by the paired analysis was also determined (Supp. Table 9, Figure 4(f)). ILK signaling and neuroinflammation signaling pathway are the most frequent pathways identified through the comparative analysis of pathways (Supp. Table 9).

Cancer, organismal injury and abnormalities, and gastrointestinal disease were the common disease, and lipid metabolism, cell-to-cell signaling, and interaction were the common molecular and cellular functions (Figures 4(c) and 4(d), Table 1(b)) associated due the sex difference in both the datasets.

### 3.4. Myeloma Cancer Cell Lines Exposed to As Trioxide (ATO)

**Figure 5:**
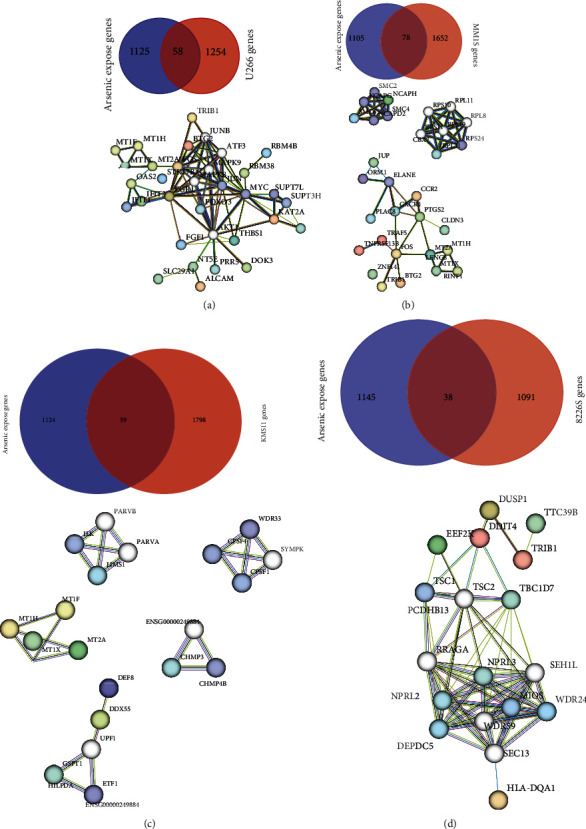
Identification of previously known arsenic exposed gene association with cancer progression. (a–d) The common arsenic exposed gene-set from Data1 and Data2 compared with differentially expressed genes within four arsenic trioxide (ATO) cell lines. Venn diagram, showing total number of common genes, (a) U266, (b) MM1S, (c) KMS11, and (d) 8226S. Interaction network functional enrichment analysis plots using STRING were demonstrated (underneath). The plots were generated with common genes identified between each cell-line and arsenic exposed gene list; the connected lines represent the degree of interconnectivity and enrichment in characteristic molecular functions.

The Methodsprovided time course and ATO exposure details for the U266, MM.1s, KMS11, and 8226/S multiple myeloma cell lines used to generate genomic data obtained from GEO. The gene expression results from Data1 and Data2 were compared with the global gene expression results from the 4 myeloma cancer cell lines exposed to ATO. The results of this comparison demonstrated that 58, 78, 59, and 38 genes were found to be commonly expressed in the arsenic exposed population and the 4 cell lines (Figures 5(a)–5(d)). An examination of this data demonstrated that there were a total of 147 unique genes (Supp. Table 10) that appeared common in the 4 cell lines and the arsenic exposed populations (Data1 and Data2). This set of 147 unique genes was used to predict urothelial cancer development and progression in the next section of results.

The functional association of common genes and interaction networks were determined by functional enrichment analysis using the STRING for each cell line (Figures 5(a)–5(d)). The gene interaction networks for each myeloma cell line identified 6 genes that were central to the interaction of the networks. These 6 genes were important transcription factors or second messengers. The EGR1 gene encodes a zinc finger protein that is a transcriptional regulator that plays a major role in cell survival, proliferation, and cell death. Its activation of p53/TP53 and TGFB1 suppresses tumor formation. MAPK8 and 9 genes are integration points for multiple biochemical signals and can influence a wide variety of cellular processes such as proliferation, differentiation, transcription regulation, and development. The FOXO3 gene functions as a transcriptional activator that regulates apoptosis and autophagy. The MYC gene is a proto-oncogene that plays a major role in cell cycle progression, apoptosis, and cellular transformation. The AKT1 gene is activated by platelet-derived growth factor and is looked upon as a survival factor that can inhibit apoptosis. The STK17B gene is a kinase involved in the regulation of apoptosis and autophagy.

**Figure 6:**
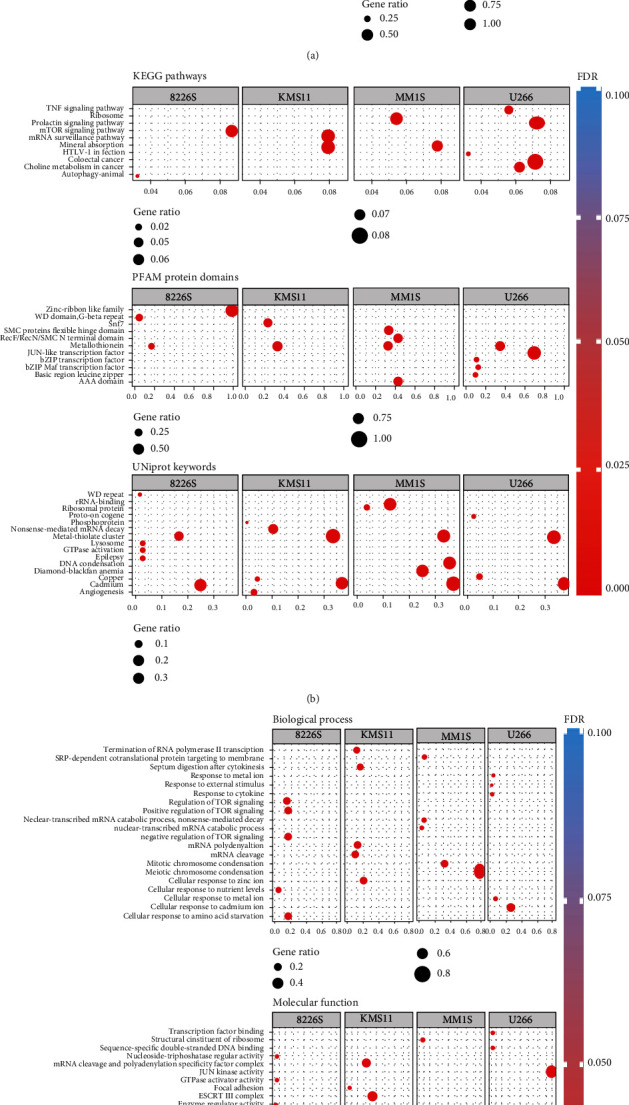
Functional analysis of arsenic exposed and cancer associated genes. To visualize the enriched terms, dot plots are generated using significantly associated pathways with arsenic exposed and cancer progression. It depicts the enrichment scores (*p* values), gene ratio as bar height, and color. The pathway databases used for significance are (a) Reactome, local STRING network clusters, (b) KEGG pathways, PFAM protein domains, Uniprot keywords, (c) biological processes, molecular functions, and cellular components.

The gene set enrichment shows various functional aspects in all four cell lines (Figure 6). The gene functions significant for U266 were associated with cellular response to the metal ion cadmium, external stimulus, and cytokine. The gene sets were also a part of pathways related to colorectal cancer, choline metabolism in cancer, HTLV-1 infection, TNF (tumor necrosis factor) signaling factor, and prolactin signaling pathway. The gene functions significant in MM1S were associated with mitotic/meiotic chromosome condensation, cellular response to Zn ion, nuclear-transcribed mRNA catabolic process, and SRP-dependent cotranslational protein targeting to the membrane. Among these genes, the pathways associated with this comparison are mineral absorption and the ribosome pathway. The biological functions related to KMS11 that are significant are a cellular response to Zn ion, mRNA polyadenylation, termination of RNA polymerase II transcription, positive regulation of viral life cycle, and viral release from the host cell. And the pathways related to these gene sets are a component of mineral absorption and mRNA surveillance. While looking at the gene sets from 8226S, the significant biological functions include TOR (target of rapamycin) signaling, response to amino acid starvation, and nutrient levels. The pathways that were related in these gene sets were mTOR signaling and autophagy pathways.

When including sex factor to identify the arsenic exposed sex specific gene association with ATO, we find an overlap of total 18 genes (BTG2, CXCR4, BACE2, EGR1, PHACTR1, CRIM1, TRIB1, TNFRSF12A, TSPAN5, RGS1, CD24, DDIT4, OLFM4, DDX3Y, PMAIP1, SLC29A1, SMAD5, and MYB) between the differentially expressed genes in arsenic exposed male/female (from Sex-Specific Gene Expression) and differently expressed genes within ATO (Supp. Table 11).

### 3.5. Bladder Cancer Prediction Model

**Figure 7:**
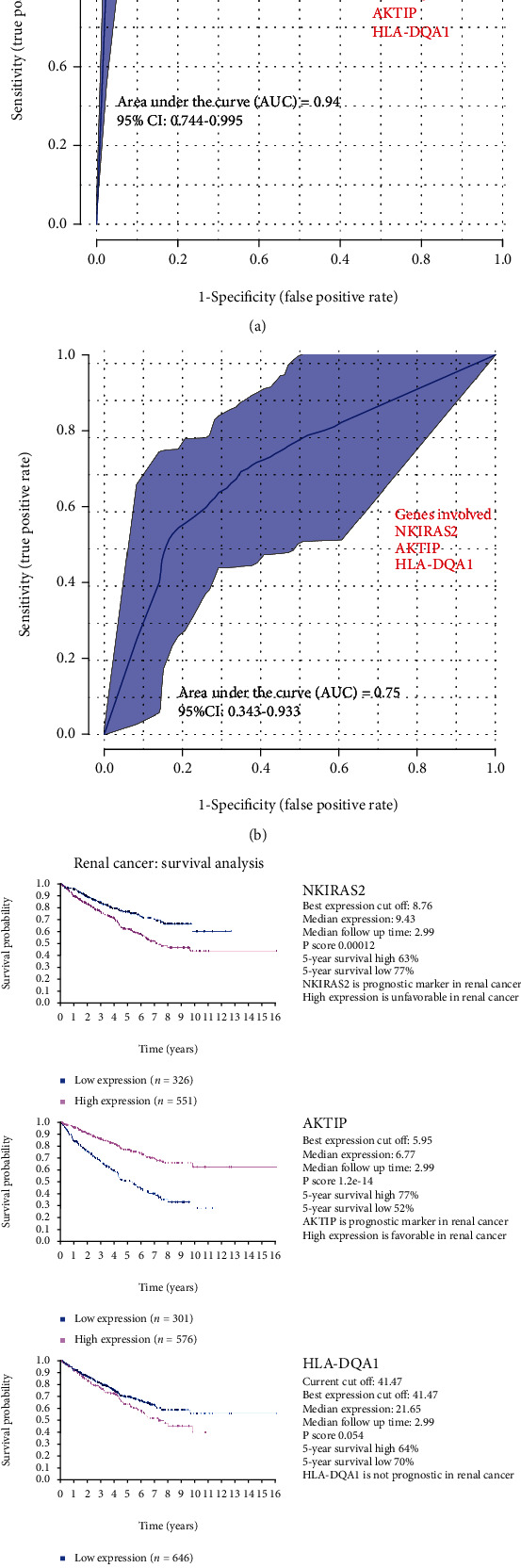
Bladder cancer prediction model. (a, b) Plot of the ROC curve as an outcome of logistic regression prediction model in multivariate fashion, by AUC ROCs. The 95% confidence intervals (CI) are shown. (a) Prediction outcome of primary tumor on GSE13507 dataset. (b) Reevaluation of prediction ability of 3 genes previously identified on GSE3167 dataset. (c) Survival outcome of three genes using the Human Protein Atlas data. Best expression cut off: based on the FPKM value of each gene, patients were classified into two groups and association between prognosis (survival), and gene expression (FPKM) was examined. The best expression cut-off refers the FPKM value that yields maximal difference with regard to survival between the two groups at the lowest log-rank *p* value. Best expression cut-off was selected based on survival analysis. Median expression refers to the median FPKM value calculated based on the gene expression (FPKM) data from all patients in this dataset. When clicking on this number, the vertical dashed line indicating cut-off, the interactive survival plot, and the Kaplan-Meier curve will be adjusted to show results based on the median expression. Median follow-up time refers to the median time (years) after diagnosis with this type of cancer, based on clinical data from all patients in this dataset. *P*score: Log-rank *p* value for Kaplan-Meier plot showing results from analysis of correlation between mRNA expression level and patient survival. 5-year survival for patients with higher expression than the expression cutoff. 5-year survival for patients with lower expression than the expression cutoff.

The 147 genes generated from the previous results section (Myeloma Cancer Cell Lines Exposed to As Trioxide) were utilized to develop a bladder cancer prediction model for the purpose of early diagnosis and prevention. Two publicly available human datasets were used as a training and validation data to test the prediction ability of those genes using the prediction model approach described in Methods. The first (GSE13507 [36, 37]) was used as a training dataset. The logistic model shows that primary tumor with three genes NKIRAS2, AKTIP, and HLA-DQA1, out of 147 with AUC 0.96 (0.82-0.99) (Figure 7(a)). The equations for the logistic model are given below with probe ID together with gene name in brackets.

GSE13507 data modeling is as follows:

- Normal vs primary tumor (GSE13507):

(1) $$\begin{array}{c} \text{logit}\left(\text{P}\right)=12.664+9.057*\text{ILMN}\_1677481\;\left(\text{NKIRAS}2-6.497*\text{ILMN}\_1665982\;\left(\text{AKTIP}\right)\right.-2.201*\text{ILMN}\_1808405\;\left(\text{HLA}-\text{DQA}1\right) \end{array}$$

Outcome Area under the curve (AUC) = 0.94(95%CI : 0.744 − 0.995) (Figure 7(a))

The same three genes were used with another set of bladder cancer data (GSE3167 [38]) to validate the primary bladder tumor predictor. It was seen that the genes (NKIRAS2, AKTIP, HLA-DQA1) show the prediction ability of AUC 0.75 (95% CI: 0.34-0.93) on this dataset (Figure 7(b)).

GSE3167 data modeling:

(b) Normal vs. primary bladder tumor model:

(2) $$\begin{array}{c} \text{logit}\left(\text{P}\right)=2.265+13.24*276\;218240\_\text{at }\left(\text{NKIRAS}2\right)-4.20*218373\_\text{at }\left(\text{AKTIP}\right)-1.55*203290\_\text{at }\left(\text{HLA}-\text{DQA}1\right) \end{array}$$

Outcome Area under the curve (AUC) = 0.75 (95%CI : 0.343 − 0.933) (Figure 7(b))

To measure the effect of the sex on this model, we wanted to include this parameter to the model, but the sex information of normal samples was not provided with data GSE13507, and therefore, we have used the intersection of arsenic exposed sex differentiated genes to find if any above gene is significantly different between male and female. We found none of those three genes were the part of 18 gene common between the sex sepecific arsenic exposed cancer gene. The human protein atlas data [58] shows that two out of three genes, i.e., NKIRAS2 (unfavorable) and AKTIP (favorable), are prognostic marker in renal cancer (Figure 7(c)).

## 4. Discussion

The major goal of the present study was to test a novel approach to elucidate genes and pathways that are associated with arsenic exposure. As we found that cancer was the top disease pathway associated with arsenic exposure, we use this list of genes and pathways to explore molecular mechanism of cancer biology with the help of ATO cell line data. We identified the major differences between male and female at gene expression level due to arsenic exposure and their role in risk prediction modeling. Studies employing other approaches have also searched for target genes associated with arsenic compounds using various cancers; for example, total arsenic concentration was related to the risk of upper tract urothelial carcinoma (UTUC), and the independent polymorphisms of the AS3MT gene were related to the risk of UTUC and bladder cancer [59]. Another study reported 19 ATO target genes associated with multiple cancer types (the most common association being pancreatic cancer) [60]. Six of these genes (AKT1, CCND1, CDKN2A, IKBKB, MAPK1, and MAPK3) were strongly associated and were used to find further mutation information. In addition, 20 ATO interacting genes were also related to other diseases such as hepatitis B, leukemia, and prostate cancer. And finally, CCND1 and MAPK1 were found to be prognostic factors in patients with pancreatic cancer. The genes responsible for metabolizing arsenic (AS3MT, GSTOs, and PNP) are of interest due to their variation in populations across different regions. Another study targeted gene associated with lung cancer and found four key genes that may affect lung cancer prognosis: MTIF2, ACOX1, CAV1, and MRPL17 [61]. This study also predicted quinostatin as a reversal to As-induced lung cell malignancy. For urothelial cancer, WNT7B, SFRP1, DNAJB2, and ATF3 were reported as target genes with cantharidin predicted as a reversal drug [62]. Some genes captured in this study have been previously identified for an association with cancer to include CNR2 that is associated with bladder cancer cell growth and motility which is linked to the cannabinoid 2 receptor-mediated modifications [63], GRR34 knockdown was shown to impair proliferation and migration of HGC-27 gastric cancer cells [64], DDHD2 as a potential cancer marker in human urine [65], and BACE2 as a prognostic marker in cervical cancer [66]. The significance of MAPK signaling, integrin-linked kinase, growth inhibitor family member 2, and NRF2-mediated oxidative stress response pathways provides an important linkage of involvement of oxidative stress and DNA damage after arsenic exposure in human which lead to carcinogenesis through dysregulation of these signaling pathways.

The key difference between these closely related studies and the current study is the process followed for capturing significant genes, where independent population data was used to generate a gene set, which was then compared to a reference dataset. In the initial analysis, two Asian populations exposed to arsenite were used to determine the common genes and pathways between the two populations based on sex and level of arsenic exposure among the 1,183 As-exposed genes. The 1,183 As-exposed genes were then correlated with the gene expression profiles of 4 multiple myeloma cell lines exposed over time to varying exposures of arsenic to generate common set of arsenic associated genes involved in canner biology, which resulted in a set of overlapping genes and relevant pathways. These genes were then examined on the patients with bladder cancer to test the cancer association with the help of developing risk prediction model. For the first time, we developed a risk prediction model for bladder cancer using an innovative new method by combining genetic data of bladder cancer risk with genetic data of arsenic exposed cancer risk factors. Importantly, we validated our model in an independent group of patients to ensure the reliability of our risk prediction, a vital step for clinical implementation.

The above process resulted in identifying 3 genes: NKIRAS2 (NFKB Inhibitor Interacting Ras Like 2), AKTIP (AKT-interacting protein), and HLA-DQA1 (Major histocompatibility complex, class II, DQ alpha 1), able to distinguish between normal urothelium and the primary urothelial carcinoma with a predictive ability of 94% using a preexisting public patient dataset. The three genes have seen only limited study as regards arsenic exposure and urothelial cancer, with the majority of information available from literature searches with bladder cancer and urothelial cancer as key words, and from web-based resources such as the Human Protein Atlas (HPA), Gene Cards (GC), NCBI, and My Cancer Genome (MCG). In most cases, the Human Protein Atlas was an excellent source of information. None of the three genes were found to be prognostic for bladder cancer (HPA). The expression of the 3 genes in urothelial cancer ranges from moderate for NKIRAS2 (NCBI), variable for AKTIP (HPA), and variable for HLA-DQA1 as determined by an immune transcriptome analysis in bladder cancer [67]. Moreover, the same genes (NKIRAS2, AKTIP, and HLA-DQA1) were also found to make a prediction ability of 75% using a validation dataset. The predictive nature of these genes clearly supports additional study to define their roles in urothelial cancer independent of sex in general and with exposure to arsenic in particular.

The studies leading up to the above prediction model also identified several interesting genes and pathways in the two populations exposed to arsenic. Three genes were identified that distinguished differences among all four scenarios of sex and arsenic exposure for the Data1 population and all six scenarios for the Data2 population. Two of these genes were noteworthy due to reports of their involvement in important biological processes. The XIST gene (X inactive specific transcript) is a noncoding RNA on the X chromosome that transcriptionally silences one of the pairs of X chromosomes for dosage equivalence between sexes. This gene is reported to be associated with several cancer types [68, 69] and has potential prognosis capabilities [70]. The expression of MALAT1 (metastasis-associated lung adenocarcinoma transcript 1) has also been associated with carcinogenesis and is a prognostic marker for lung cancer metastasis [71]. The XLOC_008276 (long intergenic nonprotein coding RNA 278) is not strongly linked to any biological process. Previous research has also found several of these genes to be significance genes in cancer progression, such as the high expression of XIST association with tumor progression and poor prognosis in bladder cancer patients [72], and high expression of MALAT1 as a possible independent prognostic factor for overall survival in patients with bladder cancer [73]. Seven common genes and six common pathways were found to overlap between the 2 populations. The CNR2 protein, while not prognostic for urothelial cancer (HPA), has been shown to modify growth and motility of human urothelial cancer cell lines [63]. The gene and protein are expressed in approximately 33% of urothelial tumors. Four of the genes, BACE2, PRKY, CST2, and PTGDR2, were reported to have no expression in urothelial cancer (HPA, GC). The remaining 2 genes, GPR34 and DDHD2, were expressed in urothelial cancer at 50% and 15%, respectively (HPA).

Nine genes were found to be overlapping when the two populations were assessed for only sex-based changes. Two of these genes, PRKY and PTGDR2, were also found in the above analysis of overlap between the two populations. Two additional genes, JAK3 and ACRBP, were reported to have no expression in urothelial cancer (HPA, NCBI, MCC). The CPA3 gene is expressed in 90% of urothelial cancers (PHA) and can induce urothelial injury but otherwise has not been studied in urothelial cancer. The remaining genes were of substantial interest for urothelial cancer and arsenic exposure. The FHL gene has been studied in a variety of cancers [74] and is prognostic for breast (favorable), renal (unfavorable), and liver (unfavorable) (HPA). The gene and protein have not been studied in urothelial cancer. The CD99L2 gene has been reported to be prognostic for urothelial cancer (unfavorable), pancreatic cancer (favorable), and lung cancer (favorable). The gene is expressed in 40% of urothelial cancers. The gene is reported to be active in a variety of tumors [75]. The SOCS3 gene is prognostic for renal cancer (unfavorable) and breast cancer (favorable) and reported to not be expressed in urothelial cancer (HPA). However, studies have reported its expression in the T24 urothelial cancer cell line [76]. The CCL2 gene is expressed in 50% of urothelial cancers and has been implicated in the growth and metastasis of urothelial cancer [77, 78]. Additionally, the 18 genes are important sex dependent genomic markers which were differently express between male and female exposed to As and associated with likelihood of cancer. Most of the genes are prognostic marker of renal cancer, whereas some of them such as BTG2, CD24, OLFM4, and BACE2 are specific to females, i.e., breast cancer and cervical cancer, and MYB specific to males, i.e., prostate cancer (Supp. Table 11).

Six genes were found to be overlapping when the two populations were assessed for level of arsenic exposure. The DDHD2 gene was also present in the above analysis of common genes between the populations in Data1 and Data2. Searching the Human Protein Atlas, ATP6V0D1 gene was prognostic for renal cancer (favorable) and pancreatic cancer (favorable); the PSPH gene was prognostic for liver cancer (unfavorable), breast cancer (unfavorable), and pancreatic cancer (favorable); and ZDHHC23 was prognostic for renal cancer (favorable), endometrial cancer (unfavorable), and thyroid cancer (unfavorable). Only the ZDHHC23 gene had confirmed expression in urothelial cancer (20%). Detailed studies in the literature for these genes in urothelial cancer were not found. The HS3ST1 gene was reported as a favorable prognostic marker for urothelial cancer, renal cancer, and endometrial cancer. Literature-based studies of these genes in urothelial cancer were not found.

## 5. Limitation

A major limitation in the current study was the lack of patient-level clinical-pathological information such as age, smoking status, and disease history, on the two populations exposed to As. Since datasets were developed on different platforms, not all the genes were present on different datasets. Therefore, to find the highest possible number of genes between those dataset, multiple testing correction was not performed which controls the Type I and Type II errors. However, the robustness of outcome was tested using different machine learning approaches such as the bladder cancer model was tested using MCCV. We did not find any genomic dataset which could provide the direct relationship between arsenic exposure and cancer in humans; therefore, we utilized the best possible option to combine the As exposed humans and cell-line to establish the relationship.

## 6. Conclusion

This study identified significant genes and pathways of interest associated with arsenic exposure in humans as well as their linkage with myeloma cancer cell lines. Oxidative stress in terms of identified genes and associated pathways shows as one of the major components associated with disease development after exposure of arsenic. To test the prediction power of those genes, we developed a regression model for urothelial carcinoma that defined a set of 3 genes: NKIRAS2, AKTIP, and HLA-DQA1, which provides the likelihood of development of primary urothelial carcinoma with same estimation for male and female.

## Figures and Tables

**Table 1:** Characteristics of arsenic exposed gene expression data. A number of samples with different characteristics such as level of arsenic exposure and gender together with the GEO ID information are provided in this table.

	Total samples	Gender		Low exposure	Medium exposure	High exposure
		Males	Females			
Data1				Water As 50–200 (*μ*g/L)	—	Water As 232–1000 (*μ*g/L)
GSE57711	29	16	13	15	—	14
Data2				Water As 122.22 ± 86.13 (*μ*g/L)	Water As 130 ± 128.9 (*μ*g/L)	Water As 148 ± 105.01 (*μ*g/L)
GSE110852	57	31	26	18	19	20

## Data Availability

Previously reported microarray data were used to support this study and are available on GEO with IDs GSE13507, GSE3167, GSE14519, GSE57711, and GSE110852. These prior studies (and datasets) are cited at relevant places within the text as references [35–38].

## Supplementary Materials

Supplementary 1 Figure S1: study flow chart part. (A) As exposed significant gene selection. (B) Find association with multiple melanoma and predictive modeling of bladder cancer.

Supplementary 2 Table S1: disease as well as molecular and cellular functions associated with statistically significant differentially expressed genes (*p* < 0.05) selected in different phenotypic conditions.

Supplementary 3 Table S2: significant pathways in Data1 and Data2 and the overlap between two (highlighted in green).

Supplementary 4 Table S3: significant pathways in Data1 and Data2 (male and female sex) and the overlap between two (highlighted in pink).

Supplementary 5 Table S4: significant pathways in Data1 and Data2 (low, medium, and high concentrations) and the overlap between various combinations (highlighted in green).

Supplementary 6 Table S5: significant pathways in Data1 (low, high concentration) and Data2 (low, medium, and high concentrations) and the overlap between two (highlighted in pink).

Supplementary 7 Table S6: significant genes in Data1 (low and high concentrations) and Data2 (low, medium, and high concentrations) and the overlap between two (highlighted in green).

Supplementary 8 Table S7: significant pathways in Data1 (low and high concentrations) and Data2 (low, medium, and high concentrations) and the overlap between two (highlighted in pink).

Supplementary 9 Table S8: significant genes in Data1 (low vs. high) and Data2 (low, medium, and high concentrations) and the overlap between various combinations.

Supplementary 10 Table S9: significant pathways in Data1 and Data2 (low, medium, and high concentrations) and the overlap between various combinations.

Supplementary 11 Table S10: list of 147 unique genes.

Supplementary 12 Table S11: survival information of 18 genes differentially expressed between sex and common between ATO and arsenic exposed human.
