# Supplementary figures and images for: Association between Arsenic Level, Gene Expression in Asian Population, and In Vitro Carcinogenic Bladder Tumor
Source: Oxid Med Cell Longev. 2022 Jan 7;2022:3459855. doi: 10.1155/2022/3459855 (PMC8760535; doi:10.1155/2022/3459855)

A

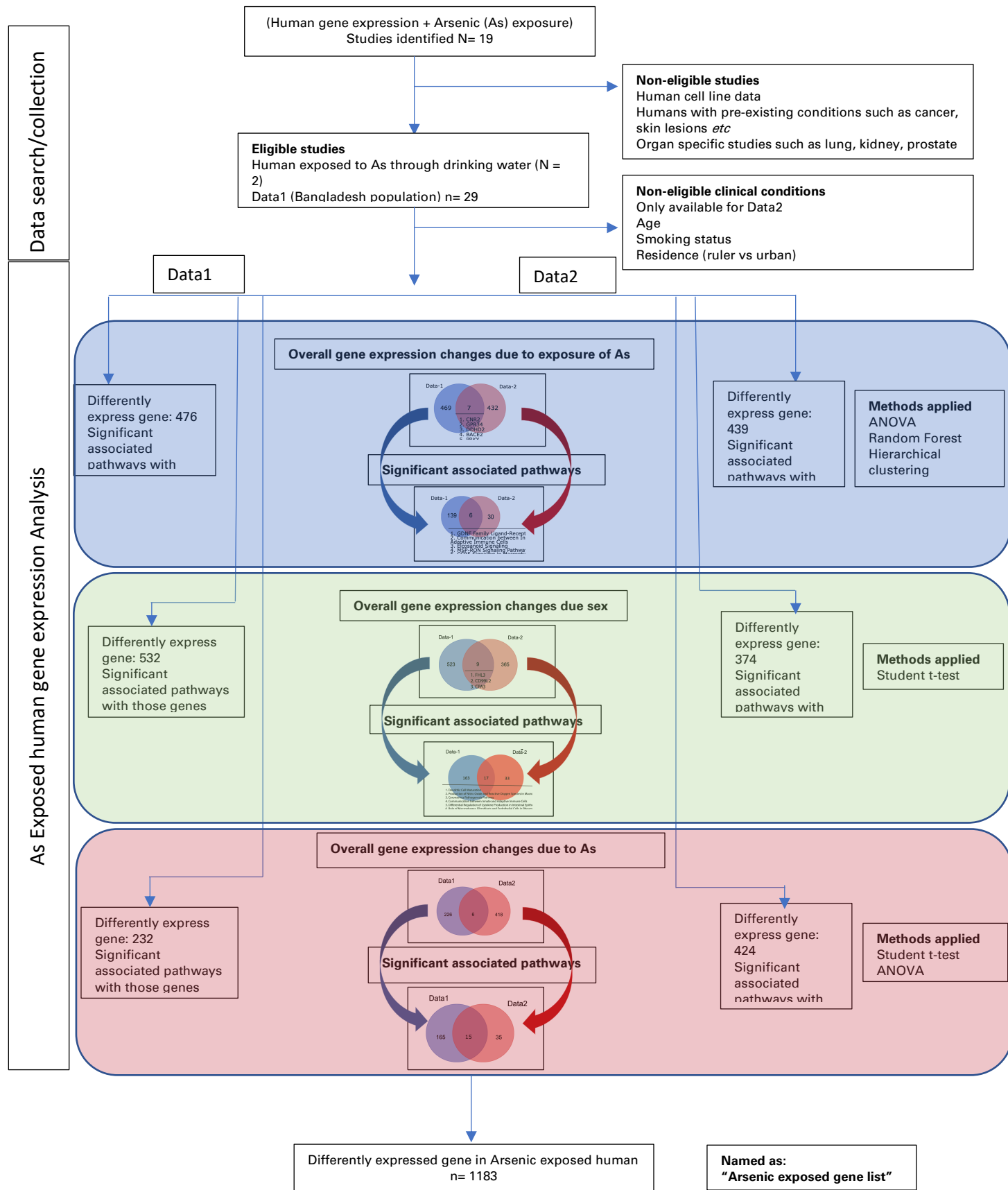

B

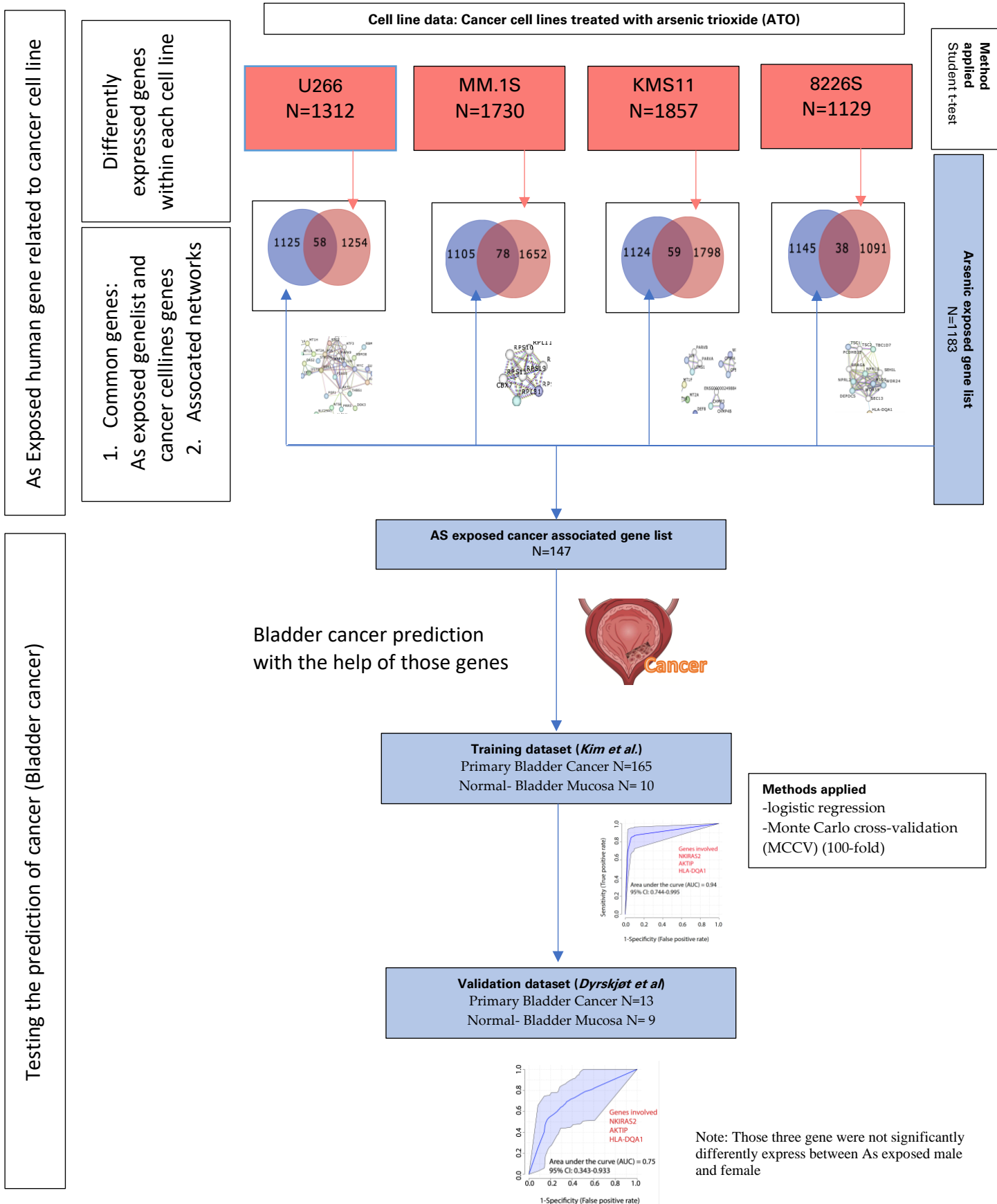

Supplement: Supplementary 1 — Figure S1: study flow chart part. (A) As exposed significant gene selection. (B) Find association with multiple melanoma and predictive modeling of bladder cancer. [file 3459855.f1.pdf]
