# Supplementary material for: Association between Arsenic Level, Gene Expression in Asian Population, and In Vitro Carcinogenic Bladder Tumor
Source: Oxid Med Cell Longev. 2022 Jan 7;2022:3459855. doi: 10.1155/2022/3459855 (PMC8760535; doi:10.1155/2022/3459855)
Supplement: Supplementary 2 — Table S1: disease as well as molecular and cellular functions associated with statistically significant differentially expressed genes (p < 0.05) selected in different phenotypic conditions. [file 3459855.f2.pdf]

Table S1. Disease as well as Molecular and Cellular Functions associated with different genes selected in different conditions.

Data1

a. Disease as well as Molecular and Cellular Functions associated with the differentially expressed genes between Low, High arsenic exposure and sex.

Disease

Name	p-value range	# Molecules
Cancer	3.75E-04 - 6.75E-18	419
Organismal Injury and Abnormalities	3.75E-04 - 6.75E-18	423
Endocrine System Disorders	2.29E-04 - 4.68E-10	334
Hematological Disease	3.44E-04 - 1.18E-09	130
Immunological Disease	3.50E-04 - 1.18E-09	172

Molecular and Cellular Functions

Name	p-value range	# Molecules
Cell Death and Survival	3.55E-04 - 9.52E-11	170
Cellular Function and Maintenance	2.88E-04 - 6.50E-10	145
Cell Morphology	3.75E-04 - 6.96E-08	45
Cell Cycle	3.61E-04 - 8.45E-08	59
Cell-To-Cell Signaling and Interaction	3.75E-04 - 1.95E-07	91

b. Disease as well as Molecular and Cellular Functions associated with the differentially expressed genes between Low vs High arsenic exposure

Disease

Name	p-value range	# Molecules
Cancer	1.10E-02 - 1.76E-14	211
Organismal Injury and Abnormalities	1.10E-02 - 1.76E-14	212
Gastrointestinal Disease	9.50E-03 - 5.81E-10	187
Endocrine System Disorders	9.95E-03 - 4.22E-07	168
Reproductive System Disease	1.10E-02 - 2.39E-06	132

Molecular and Cellular Functions

Name	p-value range	# Molecules
Protein Synthesis	1.01E-02 - 5.07E-05	43
Cell-To-Cell Signaling and Interaction	1.11E-02 - 6.81E-05	41
Cellular Assembly and Organization	9.50E-03 - 6.81E-05	25
Lipid Metabolism	9.88E-03 - 1.75E-04	11
Molecular Transport	9.88E-03 - 1.75E-04	48

c. Disease as well as Molecular and Cellular Functions associated with the differentially expressed genes between males and females

Disease

Name	p-value range	# Molecules
Connective Tissue Disorders	1.25E-05 - 1.66E-14	91
Immunological Disease	1.38E-05 - 1.66E-14	192
Inflammatory Disease	5.56E-06 - 1.66E-14	114

Organismal Injury and Abnormalities	1.43E-05 - 1.66E-14	453
Skeletal and Muscular Disorders	1.25E-05 - 1.66E-14	108

Molecular and Cellular Functions

Name	p-value range	# Molecules
Cell Death and Survival	1.28E-05 - 1.52E-12	185
Cellular Function and Maintenance	1.50E-05 - 6.78E-12	117
Cell-To-Cell Signaling and Interaction	1.48E-05 - 1.14E-11	108
Cellular Movement	1.48E-05 - 1.50E-10	145
Cellular Development	1.39E-05 - 2.07E-10	160

Data2

d. Disease as well as Molecular and Cellular Functions associated with the differentially expressed genes between Low, Medium, High arsenic exposure and sex

Disease

Name	p-value range	# Molecules
Inflammatory Response	1.21E-03 - 4.26E-12	110
Organismal Injury and Abnormalities	1.12E-03 - 4.26E-12	264
Gastrointestinal Disease	1.05E-03 - 1.22E-11	231
Connective Tissue Disorders	1.06E-03 - 4.29E-11	67
Inflammatory Disease	9.88E-04 - 4.29E-11	89

Molecular and Cellular Functions

Name	p-value range	# Molecules
Cellular Movement	1.21E-03 - 9.89E-14	103
Cellular Compromise	8.98E-04 - 2.50E-09	36
Cell Death and Survival	1.14E-03 - 5.97E-09	105
Cell-To-Cell Signaling and Interaction	9.90E-04 - 1.02E-08	62
Cell Morphology	1.21E-03 - 1.21E-07	31

e. Disease as well as Molecular and Cellular Functions associated with the differentially expressed genes between Low Medium High arsenic exposure

Disease

Name	p-value range	# Molecules
Organismal Injury and Abnormalities	4.32E-03 - 2.64E-10	269
Respiratory Disease	3.74E-03 - 2.64E-10	88
Infectious Diseases	3.09E-03 - 2.81E-10	37
Gastrointestinal Disease	4.09E-03 - 3.05E-10	153
Inflammatory Disease	3.09E-03 - 6.54E-10	70

Molecular and Cellular Functions

Name	p-value range	# Molecules
Cellular Movement	4.20E-03 - 2.37E-07	87
Cellular Compromise	3.09E-03 - 5.69E-07	31
Cellular Development	4.32E-03 - 4.72E-06	64
Cellular Growth and Proliferation	4.21E-03 - 4.72E-06	73

Cell Cycle	4.09E-03 - 7.14E-06	55

f. Disease as well as Molecular and Cellular Functions associated with the differentially expressed genes between males and females

Disease

Name	p-value range	# Molecules
Inflammatory Response	1.80E-04 - 7.99E-19	132
Organismal Injury and Abnormalities	1.85E-04 - 7.99E-19	259
Dermatological Diseases and Conditions	1.16E-04 - 3.82E-15	184
Immunological Disease	1.16E-04 - 3.82E-15	122
Inflammatory Disease	1.43E-04 - 3.42E-14	107

Molecular and Cellular Functions

Name	p-value range	# Molecules
Cell-To-Cell Signaling and Interaction	1.89E-04 - 1.60E-17	92
Cellular Movement	1.85E-04 - 2.92E-17	109
Cellular Compromise	1.42E-04 - 5.07E-12	39
Cellular Development	1.77E-04 - 3.11E-10	104
Cellular Growth and Proliferation	1.77E-04 - 3.11E-10	107

g. Disease as well as Molecular and Cellular Functions associated with the differentially expressed genes between Low High arsenic exposure

Disease

Name	p-value range	# Molecules
Cancer	4.35E-03 - 8.38E-11	340
Organismal Injury and Abnormalities	4.35E-03 - 8.38E-11	350
Inflammatory Response	4.35E-03 - 3.53E-10	113
Gastrointestinal Disease	4.35E-03 - 8.47E-09	296
Connective Tissue Disorders	3.48E-03 - 4.07E-08	79

Molecular and Cellular Functions

Name	p-value range	# Molecules
Cellular Compromise	2.49E-03 - 3.53E-10	37
Cellular Movement	4.14E-03 - 6.37E-09	110
Lipid Metabolism	4.14E-03 - 3.96E-06	36
Small Molecule Biochemistry	4.14E-03 - 3.96E-06	38
Cell-To-Cell Signaling and Interaction	3.90E-03 - 4.48E-06	61

h. Disease as well as Molecular and Cellular Functions associated with the differentially expressed genes betweenv Low Medium arsenic exposure

Disease

Name	p-value range	# Molecules
Cancer	1.25E-03 - 3.50E-13	367
Organismal Injury and Abnormalities	1.25E-03 - 3.50E-13	374
Gastrointestinal Disease	1.25E-03 - 2.83E-10	323
Respiratory Disease	8.84E-04 - 3.16E-09	116

Infectious Diseases	7.56E-04 - 5.66E-09	45

Molecular and Cellular Functions

Name	p-value range	# Molecules
Cell-To-Cell Signaling and Interaction	8.98E-04 - 1.26E-08	72
Cellular Movement	1.23E-03 - 1.90E-08	113
Cellular Compromise	2.97E-04 - 1.12E-07	38
Cellular Development	1.13E-03 - 2.45E-07	110
Cellular Growth and Proliferation	1.13E-03 - 2.45E-07	117

i. Disease as well as Molecular and Cellular Functions associated with the differentially expressed genes between Medium High arsenic exposure

Disease

Name	p-value range	# Molecules
Connective Tissue Disorders	2.40E-02 - 8.01E-06	27
Inflammatory Disease	2.40E-02 - 8.01E-06	32
Organismal Injury and Abnormalities	2.64E-02 - 8.01E-06	83
Skeletal and Muscular Disorders	2.40E-02 - 8.01E-06	27
Infectious Diseases	2.56E-02 - 1.87E-05	20

Molecular and Cellular Functions

Name	p-value range	# Molecules
Cell Morphology	2.56E-02 - 5.50E-05	20
Cellular Compromise	2.56E-02 - 5.50E-05	13
Gene Expression	2.56E-02 - 5.50E-05	13
Cellular Movement	2.56E-02 - 1.82E-04	21
Carbohydrate Metabolism	2.56E-02 - 9.86E-04	4
