# Supplementary material for: Association between Arsenic Level, Gene Expression in Asian Population, and In Vitro Carcinogenic Bladder Tumor
Source: Oxid Med Cell Longev. 2022 Jan 7;2022:3459855. doi: 10.1155/2022/3459855 (PMC8760535; doi:10.1155/2022/3459855)
Supplement: Supplementary 3 — Table S2: significant pathways in Data1 and Data2 and the overlap between two (highlighted in green). [file 3459855.f3.pdf]

Table S2. Significant pathways in Data-1 and Data-2, and the overlap between two (highlighted in green).

Data1

S.No.	Symbol	Name	f.value	p.value	X.log10.p.	FDR	Fisher.s.LSD	entrezgene	AffyID	HGNC
1	KDM6A	lysine demethylase 6A	204.8	1.02E-17	16.992	5.09E-14	high-female - high-male; low-female - high-female; high-female - low-male; low-female - high-male; low-male - high-male; low-female - low-male	7403	7403_at	HGNC:12637
2	KDM5C	lysine demethylase 5C	71.271	2.19E-12	11.66	5.47E-09	high-female - high-male; low-female - high-female; high-female - low-male; low-female - high-male; high-male - low-male; low-female - low-male	8242	8242_at	HGNC:11114
3	TMSB4Y	thymosin beta 4 Y-linked	56.911	2.60E-11	10.585	4.33E-08	high-male - high-female; low-female - high-female; low-male - high-female; high-male - low-female; high-male - low-male; low-male - low-female	9087	9087_at	HGNC:11882
4	PRKY	protein kinase Y-linked (pseudogene)	54.34	4.28E-11	10.368	5.35E-08	high-male - high-female; low-female - high-female; low-male - high-female; high-male - low-female; low-male - high-male; low-male - low-female	5616	5616_at	HGNC:9444
5	ZFX	zinc finger protein X-linked	39.957	1.09E-09	8.9622	1.02E-06	high-female - high-male; low-female - high-female; high-female - low-male; low-female - high-male; high-male - low-male; low-female - low-male	7543	7543_at	HGNC:12869
6	EIF1AX	eukaryotic translation initiation factor 1A X-linked	39.494	1.23E-09	8.9103	1.02E-06	high-female - high-male; low-female - high-female; high-female - low-male; low-female - high-male; low-male - high-male; low-female - low-male	1964	1964_at	HGNC:3250
7	SMC1A	structural maintenance of chromosomes 1A	24.427	1.33E-07	6.8764	9.49E-05	high-female - high-male; low-female - high-female; high-female - low-male; low-female - high-male; high-male - low-male; low-female - low-male	8243	8243_at	HGNC:11111

8	PRKX	protein kinase X-linked	17.646	2.31E-06	5.6356	0.0014462	high-female - high-male; low-female - high-female; high-female - low-male; low-female - high-male; high-male - low-male; low-female - low-male	5613	5613_at	HGNC:9441
9	DDX3X	DEAD-box helicase 3 X-linked	16.315	4.41E-06	5.3552	0.0024516	high-female - high-male; high-female - low-female; high-female - low-male; low-female - high-male; low-male - high-male; low-female - low-male	1654	1654_at	HGNC:2745
10	TXLNG	taxilin gamma	15.9	5.44E-06	5.2647	0.0027174	high-female - high-male; low-female - high-female; high-female - low-male; low-female - high-male; high-male - low-male; low-female - low-male	55787	55787_at	HGNC:18578
11	PNPLA4	patatin like phospholipase domain containing 4	13.133	2.40E-05	4.6199	0.010903	high-female - high-male; low-female - high-female; high-female - low-male; low-female - high-male; high-male - low-male; low-female - low-male	8228	8228_at	HGNC:24887
12	SCARNA9L	small Cajal body-specific RNA 9 like	11.662	5.70E-05	4.2438	0.023766	high-female - high-male; high-female - low-female; high-female - low-male; low-female - high-male; high-male - low-male; low-female - low-male	100158262	100158262_at	HGNC:33559
13	RPS4X	ribosomal protein S4 X-linked	11.486	6.35E-05	4.197	0.024433	high-female - high-male; high-female - low-female; high-female - low-male; low-female - high-male; low-male - high-male; low-female - low-male	6191	6191_at	HGNC:10424
14	IGSF6	immunoglobulin superfamily member 6	11.143	7.86E-05	4.1046	0.028064	high-male - high-female; high-female - low-female; low-male - high-female; high-male - low-female; low-male - high-male; low-male - low-female	10261	10261_at	HGNC:5953
15	PUDP	pseudouridine 5'-phosphatase	10.257	0.00013839	3.8589	0.046119	high-female - high-male; low-female - high-female; high-female - low-male; low-female - high-male; high-male - low-male; low-female - low-male	8226	8226_at	HGNC:16818

16	PIM1	Pim-1 proto-oncogene, serine/threonine kinase	10.066	0.00015694	3.8043	0.049035	high-female - high-male; low-female - high-female; high-female - low-male; low-female - high-male; high-male - low-male; low-female - low-male	5292	5292_at	HGNC:8986
17	MLLT3	MLLT3 super elongation complex subunit	9.5336	0.00022402	3.6497	0.065874	high-female - high-male; low-female - high-female; high-female - low-male; low-female - high-male; low-male - high-male; low-female - low-male	4300	4300_at	HGNC:7136
18	SERPINB8	serpin family B member 8	9.3304	0.0002573	3.5896	0.071459	high-male - high-female; low-female - high-female; low-male - high-female; high-male - low-female; high-male - low-male; low-male - low-female	5271	5271_at	HGNC:8952
19	CYP2S1	cytochrome P450 family 2 subfamily S member 1	9.2226	0.00027711	3.5573	0.072909	high-male - high-female; high-female - low-female; low-male - high-female; high-male - low-female; high-male - low-male; low-male - low-female	29785	29785_at	HGNC:15654
20	SEPTIN6	septin 6	8.9577	0.00033309	3.4774	0.083255	high-female - high-male; high-female - low-female; high-female - low-male; low-female - high-male; low-male - high-male; low-female - low-male	23157	23157_at	HGNC:15848
21	CA5B	carbonic anhydrase 5B	8.3437	0.00051555	3.2877	0.11509	high-female - high-male; low-female - high-female; high-female - low-male; low-female - high-male; low-male - high-male; low-female - low-male	11238	11238_at	HGNC:1378
22	ZRSR2	zinc finger CCCH-type, RNA binding motif and serine/arginine rich 2	8.3057	0.00052994	3.2758	0.11509	high-female - high-male; low-female - high-female; high-female - low-male; low-female - high-male; low-male - high-male; low-female - low-male	8233	8233_at	HGNC:23019
23	PID1	phosphotyrosine interaction domain containing 1	8.2732	0.00054256	3.2656	0.11509	high-male - high-female; high-female - low-female; low-male - high-female; high-male - low-female; high-male - low-male; low-male - low-female	55022	55022_at	HGNC:26084

24	GASK1B	golgi associated kinase 1B	8.2482	0.00055253	3.2576	0.11509	high-male - high-female; low-female - high-female; low-male - high-female; high-male - low-female; high-male - low-male; low-male - low-female	51313	51313_at	HGNC:25312
25	CLMN	calmin	8.0551	0.00063635	3.1963	0.12261	high-male - high-female; low-female - high-female; low-male - high-female; high-male - low-female; high-male - low-male; low-male - low-female	79789	79789_at	HGNC:19972
26	SASH1	SAM and SH3 domain containing 1	8.0415	0.00064275	3.192	0.12261	high-male - high-female; high-female - low-female; low-male - high-female; high-male - low-female; high-male - low-male; low-male - low-female	23328	23328_at	HGNC:19182
27	HGSNAT	heparan-alpha-glucosaminide N-acetyltransferase	7.9556	0.00068478	3.1645	0.12261	high-male - high-female; high-female - low-female; low-male - high-female; high-male - low-female; high-male - low-male; low-male - low-female	138050	138050_at	HGNC:26527
28	USP18	ubiquitin specific peptidase 18	7.9518	0.00068675	3.1632	0.12261	high-male - high-female; low-female - high-female; high-female - low-male; low-female - high-male; high-male - low-male; low-female - low-male	11274	11274_at	HGNC:12616
29	RTN1	reticulon 1	7.7284	0.00081099	3.091	0.13445	high-male - high-female; high-female - low-female; low-male - high-female; high-male - low-female; high-male - low-male; low-male - low-female	6252	6252_at	HGNC:10467
30	PCTP	phosphatidylcholine transfer protein	7.7047	0.00082556	3.0833	0.13445	high-male - high-female; high-female - low-female; low-male - high-female; high-male - low-female; high-male - low-male; low-male - low-female	58488	58488_at	HGNC:8752
31	ICOS	inducible T cell costimulator	7.6395	0.00086698	3.062	0.13445	high-female - high-male; low-female - high-female; high-female - low-male; low-female - high-male; high-male - low-male; low-female - low-male	29851	29851_at	HGNC:5351

32	SHTN1	shootin 1	7.6381	0.00086793	3.0615	0.13445	high-male - high-female; high-female - low-female; low-male - high-female; high-male - low-female; low-male - high-male; low-male - low-female	57698	57698_at	HGNC:29319
33	GLCCI1	glucocorticoid induced 1	7.6084	0.00088756	3.0518	0.13445	high-female - high-male; low-female - high-female; high-female - low-male; low-female - high-male; low-male - high-male; low-female - low-male	113263	113263_at	HGNC:18713
34	SLC36A4	solute carrier family 36 member 4	7.4697	0.00098592	3.0062	0.14496	high-male - high-female; low-female - high-female; low-male - high-female; high-male - low-female; high-male - low-male; low-male - low-female	120103	120103_at	HGNC:19660
35	TGFBI	transforming growth factor beta induced	7.398	0.0010413	2.9824	0.14503	high-male - high-female; high-female - low-female; low-male - high-female; high-male - low-female; low-male - high-male; low-male - low-female	7045	7045_at	HGNC:11771
36	DLEU1	deleted in lymphocytic leukemia 1	7.3941	0.0010444	2.9811	0.14503	high-female - high-male; low-female - high-female; high-female - low-male; low-female - high-male; high-male - low-male; low-female - low-male	10301	10301_at	HGNC:13747
37	HNMT	histamine N-methyltransferase	7.3311	0.001096	2.9602	0.14808	high-male - high-female; high-female - low-female; low-male - high-female; high-male - low-female; high-male - low-male; low-male - low-female	3176	3176_at	HGNC:5028
38	KIAA0930	KIAA0930	7.264	0.001154	2.9378	0.15181	high-male - high-female; high-female - low-female; low-male - high-female; high-male - low-female; high-male - low-male; low-male - low-female	23313	23313_at	HGNC:1314
39	HPSE	heparanase	7.0281	0.0013856	2.8584	0.17309	high-male - high-female; low-female - high-female; low-male - high-female; high-male - low-female; high-male - low-male; low-male - low-female	10855	10855_at	HGNC:5164

40	OASL	2'-5'-oligoadenylate synthetase like	7.0083	0.0014071	2.8517	0.17309	high-female - high-male; low-female - high-female; high-female - low-male; low-female - high-male; high-male - low-male; low-female - low-male	8638	8638_at	HGNC:8090
41	CREG1	cellular repressor of E1A stimulated genes 1	6.9527	0.0014698	2.8328	0.17309	high-male - high-female; low-female - high-female; low-male - high-female; high-male - low-female; high-male - low-male; low-male - low-female	8804	8804_at	HGNC:2351
42	PRCP	prolylcarboxypeptidase	6.9266	0.0015002	2.8238	0.17309	high-male - high-female; high-female - low-female; low-male - high-female; high-male - low-female; high-male - low-male; low-male - low-female	5547	5547_at	HGNC:9344
43	FBXO5	F-box protein 5	6.8966	0.001536	2.8136	0.17309	high-male - high-female; low-female - high-female; high-female - low-male; low-female - high-male; high-male - low-male; low-female - low-male	26271	26271_at	HGNC:13584
44	PSMG1	proteasome assembly chaperone 1	6.8841	0.0015512	2.8093	0.17309	high-male - high-female; low-female - high-female; high-female - low-male; low-female - high-male; high-male - low-male; low-female - low-male	8624	8624_at	HGNC:3043
45	ZNF799	zinc finger protein 799	6.8364	0.0016107	2.793	0.17309	high-male - high-female; low-female - high-female; low-male - high-female; low-female - high-male; low-male - high-male; low-female - low-male	90576	90576_at	HGNC:28071
46	DIPK1A	divergent protein kinase domain 1A	6.7809	0.001683	2.7739	0.17309	high-female - high-male; low-female - high-female; high-female - low-male; low-female - high-male; high-male - low-male; low-female - low-male	388650	388650_at	HGNC:32213
47	ZNF138	zinc finger protein 138	6.779	0.0016855	2.7733	0.17309	high-female - high-male; low-female - high-female; low-male - high-female; low-female - high-male; low-male - high-male; low-female - low-male	7697	7697_at	HGNC:12922

48	PLXDC2	plexin domain containing 2	6.7789	0.0016856	2.7732	0.17309	high-male - high-female; high-female - low-female; low-male - high-female; high-male - low-female; high-male - low-male; low-male - low-female	84898	84898_at	HGNC:21013
49	IGLV7-46	immunoglobulin lambda variable 7-46 (gene/pseudogene)	6.7707	0.0016966	2.7704	0.17309	high-female - high-male; low-female - high-female; high-female - low-male; low-female - high-male; high-male - low-male; low-female - low-male	28775	28775_at	HGNC:5930
50	CISH	cytokine inducible SH2 containing protein	6.6704	0.0018375	2.7358	0.18371	high-female - high-male; high-female - low-female; high-female - low-male; low-female - high-male; high-male - low-male; low-female - low-male	1154	1154_at	HGNC:1984
51	SCPEP1	serine carboxypeptidase 1	6.612	0.0019252	2.7155	0.18871	high-male - high-female; low-female - high-female; low-male - high-female; high-male - low-female; high-male - low-male; low-male - low-female	59342	59342_at	HGNC:29507
52	LPIN2	lipin 2	6.5798	0.0019755	2.7043	0.18892	high-female - high-male; low-female - high-female; high-female - low-male; low-female - high-male; low-male - high-male; low-female - low-male	9663	9663_at	HGNC:14450
53	CTSO	cathepsin O	6.5059	0.0020966	2.6785	0.18892	high-male - high-female; high-female - low-female; low-male - high-female; high-male - low-female; high-male - low-male; low-male - low-female	1519	1519_at	HGNC:2542
54	EPB41L3	erythrocyte membrane protein band 4.1 like 3	6.4998	0.0021069	2.6764	0.18892	high-male - high-female; high-female - low-female; low-male - high-female; high-male - low-female; high-male - low-male; low-male - low-female	23136	23136_at	HGNC:3380
55	FCER1A	Fc fragment of IgE receptor Ia	6.4993	0.0021076	2.6762	0.18892	high-male - high-female; low-female - high-female; low-male - high-female; high-male - low-female; low-male - high-male; low-male - low-female	2205	2205_at	HGNC:3609

56	ZNF510	zinc finger protein 510	6.4942	0.0021164	2.6744	0.18892	high-female - high-male; low-female - high-female; low-male - high-female; low-female - high-male; low-male - high-male; low-female - low-male	22869	22869_at	HGNC:29161
57	CTTNBP2NL	CTTNBP2 N-terminal like	6.3215	0.0024343	2.6136	0.2131	high-male - high-female; high-female - low-female; low-male - high-female; high-male - low-female; high-male - low-male; low-male - low-female	55917	55917_at	HGNC:25330
58	BCL2	BCL2 apoptosis regulator	6.2957	0.002486	2.6045	0.2131	high-female - high-male; low-female - high-female; high-female - low-male; low-female - high-male; high-male - low-male; low-female - low-male	596	596_at	HGNC:990
59	LIPA	lipase A, lysosomal acid type	6.2713	0.0025362	2.5958	0.2131	high-male - high-female; high-female - low-female; high-female - low-male; high-male - low-female; high-male - low-male; low-male - low-female	3988	3988_at	HGNC:6617
60	UBE2E2	ubiquitin conjugating enzyme E2 E2	6.2381	0.0026059	2.584	0.2131	high-male - high-female; high-female - low-female; low-male - high-female; high-male - low-female; high-male - low-male; low-male - low-female	7325	7325_at	HGNC:12478
61	PLA2G4A	phospholipase A2 group IVA	6.2281	0.0026273	2.5805	0.2131	high-male - high-female; low-female - high-female; low-male - high-female; high-male - low-female; high-male - low-male; low-male - low-female	5321	5321_at	HGNC:9035
62	THAP2	THAP domain containing 2	6.2209	0.002643	2.5779	0.2131	high-male - high-female; low-female - high-female; high-female - low-male; low-female - high-male; high-male - low-male; low-female - low-male	83591	83591_at	HGNC:20854
63	SLC2A9	solute carrier family 2 member 9	6.0969	0.0029268	2.5336	0.23133	high-male - high-female; low-female - high-female; low-male - high-female; high-male - low-female; high-male - low-male; low-male - low-female	56606	56606_at	HGNC:13446

64	ZNF43	zinc finger protein 43	6.0583	0.0030218	2.5197	0.23133	high-male - high-female; low-female - high-female; low-male - high-female; low-female - high-male; low-male - high-male; low-female - low-male	7594	7594_at	HGNC:13109
65	CD300LB	CD300 molecule like family member b	6.0424	0.0030619	2.514	0.23133	high-male - high-female; low-female - high-female; low-male - high-female; high-male - low-female; high-male - low-male; low-male - low-female	124599	124599_at	HGNC:30811
66	CPM	carboxypeptidase M	6.0253	0.0031056	2.5079	0.23133	high-male - high-female; low-female - high-female; low-male - high-female; high-male - low-female; high-male - low-male; low-male - low-female	1368	1368_at	HGNC:2311
67	SLC16A1	solute carrier family 16 member 1	5.9995	0.0031728	2.4986	0.23133	high-female - high-male; low-female - high-female; high-female - low-male; low-female - high-male; high-male - low-male; low-female - low-male	6566	6566_at	HGNC:10922
68	GPR15	G protein-coupled receptor 15	5.994	0.0031874	2.4966	0.23133	high-male - high-female; high-female - low-female; low-male - high-female; high-male - low-female; high-male - low-male; low-male - low-female	2838	2838_at	HGNC:4469
69	TMEM68	transmembrane protein 68	5.9919	0.003193	2.4958	0.23133	high-male - high-female; low-female - high-female; low-male - high-female; low-female - high-male; high-male - low-male; low-female - low-male	137695	137695_at	HGNC:26510
70	ZNF749	zinc finger protein 749	5.9472	0.0033141	2.4796	0.23439	high-male - high-female; low-female - high-female; high-female - low-male; low-female - high-male; high-male - low-male; low-female - low-male	388567	388567_at	HGNC:32783
71	SMAD5	SMAD family member 5	5.9418	0.0033289	2.4777	0.23439	high-male - high-female; low-female - high-female; low-male - high-female; high-male - low-female; low-male - high-male; low-male - low-female	4090	4090_at	HGNC:6771

72	DPP4	dipeptidyl peptidase 4	5.9153	0.0034037	2.4681	0.23632	high-female - high-male; high-female - low-female; high-female - low-male; low-female - high-male; low-male - high-male; low-female - low-male	1803	1803_at	HGNC:3009
73	CDC14A	cell division cycle 14A	5.8916	0.0034718	2.4594	0.23775	high-female - high-male; high-female - low-female; high-female - low-male; low-female - high-male; low-male - high-male; low-male - low-female	8556	8556_at	HGNC:1718
74	IGLV9-49	immunoglobulin lambda variable 9-49	5.8099	0.0037181	2.4297	0.25117	high-male - high-female; low-female - high-female; high-female - low-male; low-female - high-male; high-male - low-male; low-female - low-male	28773	28773_at	HGNC:5933
75	PLEKHF2	pleckstrin homology and FYVE domain containing 2	5.7447	0.0039281	2.4058	0.26182	high-male - high-female; low-female - high-female; low-male - high-female; low-female - high-male; high-male - low-male; low-female - low-male	79666	79666_at	HGNC:20757
76	LAX1	lymphocyte transmembrane adaptor 1	5.6937	0.004101	2.3871	0.26229	high-female - high-male; low-female - high-female; high-female - low-male; low-female - high-male; low-male - high-male; low-female - low-male	54900	54900_at	HGNC:26005
77	LDLRAD3	low density lipoprotein receptor class A domain containing 3	5.6915	0.0041087	2.3863	0.26229	high-male - high-female; high-female - low-female; low-male - high-female; high-male - low-female; high-male - low-male; low-male - low-female	143458	143458_at	HGNC:27046
78	CPVL	carboxypeptidase vitellogenic like	5.6907	0.0041116	2.386	0.26229	high-male - high-female; high-female - low-female; low-male - high-female; high-male - low-female; high-male - low-male; low-male - low-female	54504	54504_at	HGNC:14399
79	ZNF230	zinc finger protein 230	5.6812	0.004145	2.3825	0.26229	high-male - high-female; low-female - high-female; low-male - high-female; low-female - high-male; high-male - low-male; low-female - low-male	7773	7773_at	HGNC:13024

80	IRF8	interferon regulatory factor 8	5.6625	0.004211	2.3756	0.26314	high-male - high-female; low-female - high-female; low-male - high-female; high-male - low-female; low-male - high-male; low-male - low-female	3394	3394_at	HGNC:5358
81	STS	steroid sulfatase	5.6381	0.0042992	2.3666	0.26533	high-female - high-male; low-female - high-female; high-female - low-male; low-female - high-male; high-male - low-male; low-female - low-male	412	412_at	HGNC:11425
82	NIPSNAP3B	nipsnap homolog 3B	5.6102	0.0044027	2.3563	0.26537	high-male - high-female; low-female - high-female; low-male - high-female; low-female - high-male; low-male - high-male; low-male - low-female	55335	55335_at	HGNC:23641
83	LY6E	lymphocyte antigen 6 family member E	5.5775	0.004527	2.3442	0.26537	high-male - high-female; low-female - high-female; high-female - low-male; high-male - low-female; high-male - low-male; low-female - low-male	4061	4061_at	HGNC:6727
84	SLC37A2	solute carrier family 37 member 2	5.5731	0.004544	2.3426	0.26537	high-male - high-female; low-female - high-female; low-male - high-female; high-male - low-female; high-male - low-male; low-female - low-male	219855	219855_at	HGNC:20644
85	DTX1	deltex E3 ubiquitin ligase 1	5.5519	0.0046272	2.3347	0.26537	high-male - high-female; low-female - high-female; low-male - high-female; low-female - high-male; low-male - high-male; low-female - low-male	1840	1840_at	HGNC:3060
86	PKIG	cAMP-dependent protein kinase inhibitor gamma	5.5474	0.0046451	2.333	0.26537	high-male - high-female; low-female - high-female; high-female - low-male; low-female - high-male; high-male - low-male; low-female - low-male	11142	11142_at	HGNC:9019
87	RAB31	RAB31, member RAS oncogene family	5.5301	0.0047144	2.3266	0.26537	high-male - high-female; high-female - low-female; low-male - high-female; high-male - low-female; high-male - low-male; low-male - low-female	11031	11031_at	HGNC:9771

88	TNFSF13	TNF superfamily member 13	5.5232	0.0047423	2.324	0.26537	high-male - high-female; low-female - high-female; low-male - high-female; high-male - low-female; high-male - low-male; low-male - low-female	8741	8741_at	HGNC:11928
89	HLA-DMA	major histocompatibility complex, class II, DM alpha	5.5192	0.0047586	2.3225	0.26537	high-male - high-female; high-female - low-female; low-male - high-female; high-male - low-female; high-male - low-male; low-male - low-female	3108	3108_at	HGNC:4934
90	CEBPG	CCAAT enhancer binding protein gamma	5.5145	0.0047776	2.3208	0.26537	high-male - high-female; low-female - high-female; low-male - high-female; high-male - low-female; high-male - low-male; low-male - low-female	1054	1054_at	HGNC:1837
91	OPN3	opsin 3	5.4903	0.0048778	2.3118	0.26765	high-male - high-female; low-female - high-female; low-male - high-female; high-male - low-female; high-male - low-male; low-female - low-male	23596	23596_at	HGNC:14007
92	FCER1G	Fc fragment of IgE receptor Ig	5.4693	0.0049666	2.3039	0.26765	high-female - high-male; high-female - low-female; high-female - low-male; high-male - low-female; high-male - low-male; low-male - low-female	2207	2207_at	HGNC:3611
93	MSH2	mutS homolog 2	5.4539	0.0050327	2.2982	0.26765	high-male - high-female; low-female - high-female; low-male - high-female; low-female - high-male; high-male - low-male; low-female - low-male	4436	4436_at	HGNC:7325
94	ANKRD50	ankyrin repeat domain 50	5.4539	0.0050328	2.2982	0.26765	high-male - high-female; low-female - high-female; low-male - high-female; high-male - low-female; high-male - low-male; low-male - low-female	57182	57182_at	HGNC:29223
95	TSHZ3	teashirt zinc finger homeobox 3	5.4043	0.0052525	2.2796	0.27111	high-male - high-female; high-female - low-female; low-male - high-female; high-male - low-female; high-male - low-male; low-male - low-female	57616	57616_at	HGNC:30700

96	FAR1	fatty acyl-CoA reductase 1	5.399	0.0052768	2.2776	0.27111	high-male - high-female; low-female - high-female; low-male - high-female; high-male - low-female; high-male - low-male; low-male - low-female	84188	84188_at	HGNC:26222
97	ZNF649	zinc finger protein 649	5.3921	0.0053084	2.275	0.27111	high-female - high-male; low-female - high-female; low-male - high-female; low-female - high-male; low-male - high-male; low-female - low-male	65251	65251_at	HGNC:25741
98	TMEM156	transmembrane protein 156	5.3803	0.0053625	2.2706	0.27111	high-female - high-male; low-female - high-female; low-male - high-female; low-female - high-male; low-male - high-male; low-female - low-male	80008	80008_at	HGNC:26260
99	METTL18	methyltransferase like 18	5.3789	0.005369	2.2701	0.27111	high-male - high-female; low-female - high-female; low-male - high-female; low-female - high-male; low-male - high-male; low-female - low-male	92342	92342_at	HGNC:28793
100	MT1H	metallothionein 1H	5.3582	0.0054662	2.2623	0.27219	high-male - high-female; high-female - low-female; high-female - low-male; high-male - low-female; high-male - low-male; low-female - low-male	4496	4496_at	HGNC:7400
101	MS4A6A	membrane spanning 4-domains A6A	5.3456	0.0055261	2.2576	0.27219	high-male - high-female; high-female - low-female; low-male - high-female; high-male - low-female; high-male - low-male; low-male - low-female	64231	64231_at	HGNC:13375
102	IDH1	isocitrate dehydrogenase (NADP(+)) 1	5.3398	0.0055538	2.2554	0.27219	high-male - high-female; low-female - high-female; low-male - high-female; high-male - low-female; high-male - low-male; low-male - low-female	3417	3417_at	HGNC:5382
103	PIM2	Pim-2 proto-oncogene, serine/threonine kinase	5.3236	0.0056325	2.2493	0.27319	high-female - high-male; high-female - low-female; high-female - low-male; low-female - high-male; high-male - low-male; low-female - low-male	11040	11040_at	HGNC:8987

104	HLA-DPB2	major histocompatibility complex, class II, DP beta 2 (pseudogene)	5.3079	0.0057098	2.2434	0.27319	high-male - high-female; high-female - low-female; low-male - high-female; high-male - low-female; high-male - low-male; low-male - low-female	3116	3116_at	HGNC:4941
105	GINS2	GINS complex subunit 2	5.2944	0.0057773	2.2383	0.27319	high-male - high-female; low-female - high-female; high-female - low-male; low-female - high-male; high-male - low-male; low-female - low-male	51659	51659_at	HGNC:24575
106	MPZL2	myelin protein zero like 2	5.269	0.0059063	2.2287	0.27319	high-male - high-female; low-female - high-female; low-male - high-female; high-male - low-female; high-male - low-male; low-male - low-female	10205	10205_at	HGNC:3496
107	C3orf14	chromosome 3 open reading frame 14	5.2649	0.0059272	2.2271	0.27319	high-male - high-female; high-female - low-female; low-male - high-female; high-male - low-female; high-male - low-male; low-male - low-female	57415	57415_at	HGNC:25024
108	NAPRT	nicotinate phosphoribosyltransferase	5.2437	0.0060377	2.2191	0.27319	high-male - high-female; low-female - high-female; low-male - high-female; high-male - low-female; high-male - low-male; low-male - low-female	93100	93100_at	HGNC:30450
109	EEF2K	eukaryotic elongation factor 2 kinase	5.2435	0.0060388	2.219	0.27319	high-male - high-female; low-female - high-female; low-male - high-female; low-female - high-male; low-male - high-male; low-male - low-female	29904	29904_at	HGNC:24615
110	LMBR1	limb development membrane protein 1	5.2415	0.0060494	2.2183	0.27319	high-male - high-female; low-female - high-female; low-male - high-female; low-female - high-male; low-male - high-male; low-male - low-female	64327	64327_at	HGNC:13243
111	RIOK2	RIO kinase 2	5.2383	0.0060659	2.2171	0.27319	high-male - high-female; low-female - high-female; low-male - high-female; low-female - high-male; high-male - low-male; low-female - low-male	55781	55781_at	HGNC:18999

112	OXSM	3-oxoacyl-ACP synthase, mitochondrial	5.2031	0.0062555	2.2037	0.2786	high-male - high-female; low-female - high-female; high-female - low-male; low-female - high-male; high-male - low-male; low-female - low-male	54995	54995_at	HGNC:26063
113	SERTAD3	SERTA domain containing 3	5.1927	0.0063124	2.1998	0.2786	high-male - high-female; low-female - high-female; low-male - high-female; high-male - low-female; high-male - low-male; low-male - low-female	29946	29946_at	HGNC:17931
114	SMIM3	small integral membrane protein 3	5.1828	0.0063677	2.196	0.2786	high-male - high-female; high-female - low-female; high-female - low-male; high-male - low-female; high-male - low-male; low-male - low-female	85027	85027_at	HGNC:30248
115	SLC44A1	solute carrier family 44 member 1	5.1754	0.0064091	2.1932	0.2786	high-male - high-female; low-female - high-female; low-male - high-female; high-male - low-female; high-male - low-male; low-male - low-female	23446	23446_at	HGNC:18798
116	SETD7	SET domain containing 7, histone lysine methyltransferase	5.1554	0.0065225	2.1856	0.27903	high-male - high-female; low-female - high-female; low-male - high-female; high-male - low-female; low-male - high-male; low-male - low-female	80854	80854_at	HGNC:30412
117	ZNF440	zinc finger protein 440	5.1473	0.0065689	2.1825	0.27903	high-male - high-female; low-female - high-female; low-male - high-female; low-female - high-male; low-male - high-male; low-female - low-male	126070	126070_at	HGNC:20874
118	BACE2	beta-secretase 2	5.1443	0.0065864	2.1814	0.27903	high-male - high-female; low-female - high-female; low-male - high-female; low-female - high-male; low-male - high-male; low-female - low-male	25825	25825_at	HGNC:934
119	MT1X	metallothionein 1X	5.1107	0.0067836	2.1685	0.28497	high-female - high-male; high-female - low-female; high-female - low-male; high-male - low-female; high-male - low-male; low-female - low-male	4501	4501_at	HGNC:7405

120	CCDC144NL	CCDC144A N-terminal pseudogene	5.0926	0.0068925	2.1616	0.28711	high-female - high-male; low-female - high-female; low-male - high-female; low-female - high-male; low-male - high-male; low-male - low-female	339184	339184_at	HGNC:33735
121	RDX	radixin	5.0832	0.0069494	2.1581	0.28711	high-male - high-female; low-female - high-female; low-male - high-female; high-male - low-female; high-male - low-male; low-female - low-male	5962	5962_at	HGNC:9944
122	RAB34	RAB34, member RAS oncogene family	5.0261	0.0073085	2.1362	0.29646	high-male - high-female; low-female - high-female; low-male - high-female; high-male - low-female; high-male - low-male; low-male - low-female	83871	83871_at	HGNC:16519
123	LARP4	La ribonucleoprotein 4	5.0038	0.0074544	2.1276	0.29646	high-male - high-female; low-female - high-female; low-male - high-female; low-female - high-male; high-male - low-male; low-female - low-male	113251	113251_at	HGNC:24320
124	SYAP1	synapse associated protein 1	4.997	0.0074991	2.125	0.29646	high-female - high-male; low-female - high-female; high-female - low-male; low-female - high-male; high-male - low-male; low-female - low-male	94056	94056_at	HGNC:16273
125	SLC31A1	solute carrier family 31 member 1	4.9761	0.0076394	2.1169	0.29646	high-male - high-female; high-female - low-female; low-male - high-female; high-male - low-female; high-male - low-male; low-male - low-female	1317	1317_at	HGNC:11016
126	SUDS3	SDS3 homolog, SIN3A corepressor complex component	4.9706	0.0076771	2.1148	0.29646	high-male - high-female; low-female - high-female; low-male - high-female; low-female - high-male; low-male - high-male; low-female - low-male	64426	64426_at	HGNC:29545
127	CKS2	CDC28 protein kinase regulatory subunit 2	4.9632	0.0077272	2.112	0.29646	high-male - high-female; low-female - high-female; high-female - low-male; low-female - high-male; high-male - low-male; low-female - low-male	1164	1164_at	HGNC:2000

128	GLCE	glucuronic acid epimerase	4.9561	0.0077763	2.1092	0.29646	high-male - high-female; low-female - high-female; low-male - high-female; high-male - low-female; high-male - low-male; low-male - low-female	26035	26035_at	HGNC:17855
129	RBM3	RNA binding motif protein 3	4.9558	0.0077781	2.1091	0.29646	high-male - high-female; high-female - low-female; low-male - high-female; high-male - low-female; high-male - low-male; low-male - low-female	5935	5935_at	HGNC:9900
130	CCDC88A	coiled-coil domain containing 88A	4.9531	0.0077971	2.1081	0.29646	high-male - high-female; high-female - low-female; low-male - high-female; high-male - low-female; low-male - high-male; low-male - low-female	55704	55704_at	HGNC:25523
131	LRIG1	leucine rich repeats and immunoglobulin like domains 1	4.9523	0.0078025	2.1078	0.29646	high-female - high-male; low-female - high-female; low-male - high-female; low-female - high-male; low-male - high-male; low-female - low-male	26018	26018_at	HGNC:17360
132	TAS2R20	taste 2 receptor member 20	4.9486	0.0078282	2.1063	0.29646	high-female - high-male; low-female - high-female; low-male - high-female; low-female - high-male; low-male - high-male; low-male - low-female	259295	259295_at	HGNC:19109
133	BANF1	BAF nuclear assembly factor 1	4.931	0.0079521	2.0995	0.29889	high-male - high-female; low-female - high-female; low-male - high-female; high-male - low-female; high-male - low-male; low-female - low-male	8815	8815_at	HGNC:17397
134	CDK6	cyclin dependent kinase 6	4.921	0.0080227	2.0957	0.2993	high-female - high-male; high-female - low-female; high-female - low-male; low-female - high-male; high-male - low-male; low-female - low-male	1021	1021_at	HGNC:1777
135	FOLR3	folate receptor gamma	4.9035	0.0081488	2.0889	0.30073	high-male - high-female; high-female - low-female; low-male - high-female; high-male - low-female; high-male - low-male; low-male - low-female	2352	2352_at	HGNC:3795

136	FEM1B	fem-1 homolog B	4.899	0.0081816	2.0872	0.30073	high-male - high-female; low-female - high-female; low-male - high-female; high-male - low-female; high-male - low-male; low-female - low-male	10116	10116_at	HGNC:3649
137	SNORD38B	small nucleolar RNA, C/D box 38B	4.8817	0.0083085	2.0805	0.30317	high-male - high-female; low-female - high-female; low-male - high-female; low-female - high-male; high-male - low-male; low-female - low-male	94163	94163_at	HGNC:30356
138	ZNF844	zinc finger protein 844	4.8388	0.0086336	2.0638	0.31175	high-female - high-male; low-female - high-female; low-male - high-female; low-female - high-male; low-male - high-male; low-female - low-male	284391	284391_at	HGNC:25932
139	ZNF141	zinc finger protein 141	4.8323	0.0086839	2.0613	0.31175	high-female - high-male; low-female - high-female; low-male - high-female; low-female - high-male; low-male - high-male; low-female - low-male	7700	7700_at	HGNC:12926
140	SNORD47	small nucleolar RNA, C/D box 47	4.8263	0.0087308	2.0589	0.31175	high-male - high-female; low-female - high-female; high-female - low-male; low-female - high-male; high-male - low-male; low-female - low-male	26802	26802_at	HGNC:10187
141	MGST1	microsomal glutathione S-transferase 1	4.8014	0.0089274	2.0493	0.3136	high-male - high-female; high-female - low-female; low-male - high-female; high-male - low-female; high-male - low-male; low-male - low-female	4257	4257_at	HGNC:7061
142	ZBED1	zinc finger BED-type containing 1	4.7987	0.0089496	2.0482	0.3136	high-male - high-female; low-female - high-female; low-male - high-female; high-male - low-female; high-male - low-male; low-female - low-male	9189	9189_at	HGNC:447
143	CCDC149	coiled-coil domain containing 149	4.796	0.0089708	2.0472	0.3136	high-male - high-female; low-female - high-female; low-male - high-female; high-male - low-female; high-male - low-male; low-female - low-male	91050	91050_at	HGNC:25405

144	PLA2G15	phospholipase A2 group XV	4.7875	0.0090401	2.0438	0.31383	high-male - high-female; low-female - high-female; low-male - high-female; high-male - low-female; high-male - low-male; low-male - low-female	23659	23659_at	HGNC:17163
145	ZBTB25	zinc finger and BTB domain containing 25	4.723	0.0095799	2.0186	0.32916	high-female - high-male; low-female - high-female; low-male - high-female; low-female - high-male; low-male - high-male; low-male - low-female	7597	7597_at	HGNC:13112
146	RHOH	ras homolog family member H	4.7191	0.0096134	2.0171	0.32916	high-female - high-male; low-female - high-female; high-female - low-male; low-female - high-male; high-male - low-male; low-female - low-male	399	399_at	HGNC:686
147	LACC1	laccase domain containing 1	4.6737	0.010016	1.9993	0.34062	high-male - high-female; high-female - low-female; low-male - high-female; high-male - low-female; low-male - high-male; low-male - low-female	144811	144811_at	HGNC:26789
148	ABHD10	abhydrolase domain containing 10	4.6241	0.010476	1.9798	0.35107	high-male - high-female; low-female - high-female; low-male - high-female; low-female - high-male; high-male - low-male; low-female - low-male	55347	55347_at	HGNC:25656
149	SNAI3	snail family transcriptional repressor 3	4.62	0.010515	1.9782	0.35107	high-male - high-female; low-female - high-female; low-male - high-female; high-male - low-female; high-male - low-male; low-male - low-female	333929	333929_at	HGNC:18411
150	MYPOP	Myb related transcription factor, partner of profilin	4.6144	0.010569	1.976	0.35107	high-male - high-female; high-female - low-female; low-male - high-female; high-male - low-female; high-male - low-male; low-male - low-female	339344	339344_at	HGNC:20178
151	TRAPPC2L	trafficking protein particle complex 2 like	4.5863	0.010842	1.9649	0.35107	high-male - high-female; low-female - high-female; high-female - low-male; low-female - high-male; high-male - low-male; low-female - low-male	51693	51693_at	HGNC:30887

152	ITSN1	intersectin 1	4.5774	0.01093	1.9614	0.35107	high-male - high-female; low-female - high-female; low-male - high-female; high-male - low-female; high-male - low-male; low-male - low-female	6453	6453_at	HGNC:6183
153	MAN2B1	mannosidase alpha class 2B member 1	4.5772	0.010932	1.9613	0.35107	high-male - high-female; high-female - low-female; low-male - high-female; high-male - low-female; high-male - low-male; low-male - low-female	4125	4125_at	HGNC:6826
154	MORN2	MORN repeat containing 2	4.562	0.011085	1.9553	0.35107	high-male - high-female; high-female - low-female; low-male - high-female; high-male - low-female; high-male - low-male; low-male - low-female	729967	729967_at	HGNC:30166
155	SLC43A3	solute carrier family 43 member 3	4.5618	0.011087	1.9552	0.35107	high-male - high-female; high-female - low-female; low-male - high-female; high-male - low-female; high-male - low-male; low-male - low-female	29015	29015_at	HGNC:17466
156	CXCL16	C-X-C motif chemokine ligand 16	4.5572	0.011133	1.9534	0.35107	high-male - high-female; high-female - low-female; low-male - high-female; high-male - low-female; high-male - low-male; low-male - low-female	58191	58191_at	HGNC:16642
157	CFD	complement factor D	4.5536	0.01117	1.952	0.35107	high-male - high-female; high-female - low-female; low-male - high-female; high-male - low-female; high-male - low-male; low-male - low-female	1675	1675_at	HGNC:2771
158	CRIM1	cysteine rich transmembrane BMP regulator 1	4.5465	0.011243	1.9491	0.35107	high-female - high-male; low-female - high-female; high-female - low-male; low-female - high-male; low-male - high-male; low-female - low-male	51232	51232_at	HGNC:2359
159	SNX25	sorting nexin 25	4.5425	0.011283	1.9476	0.35107	high-female - high-male; low-female - high-female; low-male - high-female; low-female - high-male; low-male - high-male; low-female - low-male	83891	83891_at	HGNC:21883

160	GNG5	G protein subunit gamma 5	4.5422	0.011287	1.9474	0.35107	high-male - high-female; high-female - low-female; low-male - high-female; high-male - low-female; high-male - low-male; low-male - low-female	2787	2787_at	HGNC:4408
161	GVINP1	GTPase, very large interferon inducible pseudogene 1	4.5357	0.011354	1.9448	0.35107	high-female - high-male; low-female - high-female; high-female - low-male; low-female - high-male; low-male - high-male; low-female - low-male	387751	387751_at	HGNC:25813
162	H4C9	H4 clustered histone 9	4.5283	0.011431	1.9419	0.35107	high-female - high-male; low-female - high-female; high-female - low-male; low-female - high-male; high-male - low-male; low-female - low-male	8294	8294_at	HGNC:4793
163	TBXAS1	thromboxane A synthase 1	4.5267	0.011447	1.9413	0.35107	high-male - high-female; high-female - low-female; low-male - high-female; high-male - low-female; high-male - low-male; low-male - low-female	6916	6916_at	HGNC:11609
164	SLC30A1	solute carrier family 30 member 1	4.4948	0.011787	1.9286	0.35927	high-male - high-female; low-female - high-female; low-male - high-female; high-male - low-female; high-male - low-male; low-female - low-male	7779	7779_at	HGNC:11012
165	TRAV19	T cell receptor alpha variable 19	4.4879	0.011861	1.9259	0.35936	high-female - high-male; high-female - low-female; high-female - low-male; low-female - high-male; low-male - high-male; low-female - low-male	28664	28664_at	HGNC:12115
166	GPX7	glutathione peroxidase 7	4.4757	0.011994	1.921	0.3612	high-female - high-male; low-female - high-female; high-female - low-male; low-female - high-male; high-male - low-male; low-female - low-male	2882	2882_at	HGNC:4559
167	CIAPIN1	cytokine induced apoptosis inhibitor 1	4.4415	0.012377	1.9074	0.36622	high-male - high-female; low-female - high-female; high-female - low-male; low-female - high-male; high-male - low-male; low-female - low-male	57019	57019_at	HGNC:28050

168	CD99L2	CD99 molecule like 2	4.4347	0.012454	1.9047	0.36622	high-male - high-female; high-female - low-female; low-male - high-female; high-male - low-female; high-male - low-male; low-male - low-female	83692	83692_at	HGNC:18237
169	IRAK3	interleukin 1 receptor associated kinase 3	4.4316	0.01249	1.9034	0.36622	high-male - high-female; high-female - low-female; low-male - high-female; high-male - low-female; high-male - low-male; low-male - low-female	11213	11213_at	HGNC:17020
170	HNRNPA0	heterogeneous nuclear ribonucleoprotein A0	4.4275	0.012537	1.9018	0.36622	high-male - high-female; low-female - high-female; low-male - high-female; high-male - low-female; high-male - low-male; low-female - low-male	10949	10949_at	HGNC:5030
171	FAH	fumarylacetoacetate hydrolase	4.4249	0.012566	1.9008	0.36622	high-male - high-female; low-female - high-female; low-male - high-female; high-male - low-female; high-male - low-male; low-male - low-female	2184	2184_at	HGNC:3579
172	ENTPD1	ectonucleoside triphosphate diphosphohydrolase 1	4.422	0.0126	1.8996	0.36622	high-male - high-female; high-female - low-female; low-male - high-female; high-male - low-female; high-male - low-male; low-male - low-female	953	953_at	HGNC:3363
173	OFD1	OFD1 centriole and centriolar satellite protein	4.4135	0.0127	1.8962	0.36696	high-female - high-male; low-female - high-female; high-female - low-male; low-female - high-male; low-male - high-male; low-female - low-male	8481	8481_at	HGNC:2567
174	PSIP1	PC4 and SFRS1 interacting protein 1	4.4012	0.012843	1.8913	0.36899	high-female - high-male; low-female - high-female; low-male - high-female; low-female - high-male; low-male - high-male; low-female - low-male	11168	11168_at	HGNC:9527
175	FRG1BP	FSHD region gene 1 family member B, pseudogene	4.3931	0.01294	1.8881	0.36963	high-male - high-female; high-female - low-female; low-male - high-female; high-male - low-female; high-male - low-male; low-male - low-female	284802	284802_at	HGNC:15792

176	CXXC5	CXXC finger protein 5	4.3795	0.013103	1.8826	0.37216	high-male - high-female; low-female - high-female; low-male - high-female; low-female - high-male; high-male - low-male; low-female - low-male	51523	51523_at	HGNC:26943
177	SEMA4A	semaphorin 4A	4.3616	0.013321	1.8755	0.37621	high-male - high-female; high-female - low-female; high-female - low-male; high-male - low-female; high-male - low-male; low-male - low-female	64218	64218_at	HGNC:10729
178	MFSD2A	major facilitator superfamily domain containing 2A	4.3551	0.013402	1.8728	0.37637	high-male - high-female; low-female - high-female; low-male - high-female; high-male - low-female; high-male - low-male; low-female - low-male	84879	84879_at	HGNC:25897
179	ZNF429	zinc finger protein 429	4.3482	0.013486	1.8701	0.37664	high-male - high-female; low-female - high-female; low-male - high-female; low-female - high-male; high-male - low-male; low-female - low-male	353088	353088_at	HGNC:20817
180	HMGN1	high mobility group nucleosome binding domain 1	4.331	0.013703	1.8632	0.37931	high-male - high-female; low-female - high-female; high-female - low-male; low-female - high-male; high-male - low-male; low-female - low-male	3150	3150_at	HGNC:4984
181	ZBTB16	zinc finger and BTB domain containing 16	4.3269	0.013756	1.8615	0.37931	high-male - high-female; low-female - high-female; low-male - high-female; low-female - high-male; low-male - high-male; low-female - low-male	7704	7704_at	HGNC:12930
182	OGFRL1	opioid growth factor receptor like 1	4.3226	0.01381	1.8598	0.37931	high-male - high-female; high-female - low-female; low-male - high-female; high-male - low-female; high-male - low-male; low-male - low-female	79627	79627_at	HGNC:21378
183	ERCC6L2	ERCC excision repair 6 like 2	4.3158	0.013896	1.8571	0.37961	high-female - high-male; low-female - high-female; low-male - high-female; low-female - high-male; low-male - high-male; low-female - low-male	375748	375748_at	HGNC:26922

184	ZNF570	zinc finger protein 570	4.3099	0.013974	1.8547	0.37964	high-male - high-female; low-female - high-female; low-male - high-female; low-female - high-male; low-male - high-male; low-female - low-male	148268	148268_at	HGNC:26416
185	CARD11	caspase recruitment domain family member 11	4.298	0.014128	1.8499	0.38175	high-male - high-female; low-female - high-female; low-male - high-female; low-female - high-male; low-male - high-male; low-female - low-male	84433	84433_at	HGNC:16393
186	ARSD	arylsulfatase D	4.2886	0.014252	1.8461	0.38304	high-female - high-male; low-female - high-female; high-female - low-male; low-female - high-male; high-male - low-male; low-female - low-male	414	414_at	HGNC:717
187	RHOQ	ras homolog family member Q	4.2777	0.014396	1.8417	0.38485	high-male - high-female; low-female - high-female; low-male - high-female; low-female - high-male; high-male - low-male; low-female - low-male	23433	23433_at	HGNC:17736
188	SPIC	Spi-C transcription factor	4.2391	0.014922	1.8262	0.39678	high-female - high-male; high-female - low-female; high-female - low-male; high-male - low-female; high-male - low-male; low-female - low-male	121599	121599_at	HGNC:29549
189	CST2	cystatin SA	4.2324	0.015016	1.8234	0.39718	high-male - high-female; low-female - high-female; low-male - high-female; high-male - low-female; high-male - low-male; low-male - low-female	1470	1470_at	HGNC:2474
190	JMJD8	jumonji domain containing 8	4.2172	0.015229	1.8173	0.40065	high-male - high-female; low-female - high-female; low-male - high-female; high-male - low-female; high-male - low-male; low-male - low-female	339123	339123_at	HGNC:14148
191	MRPS18A	mitochondrial ribosomal protein S18A	4.2117	0.015308	1.8151	0.40065	high-male - high-female; low-female - high-female; low-male - high-female; high-male - low-female; high-male - low-male; low-female - low-male	55168	55168_at	HGNC:14515

192	ARID5B	AT-rich interaction domain 5B	4.1948	0.015552	1.8082	0.40068	high-female - high-male; low-female - high-female; high-female - low-male; low-female - high-male; high-male - low-male; low-female - low-male	84159	84159_at	HGNC:17362
193	GRAMD4	GRAM domain containing 4	4.1935	0.015569	1.8077	0.40068	high-male - high-female; low-female - high-female; low-male - high-female; high-male - low-female; high-male - low-male; low-male - low-female	23151	23151_at	HGNC:29113
194	GPR160	G protein-coupled receptor 160	4.1929	0.015578	1.8075	0.40068	high-male - high-female; low-female - high-female; low-male - high-female; high-male - low-female; high-male - low-male; low-male - low-female	26996	26996_at	HGNC:23693
195	NUP210	nucleoporin 210	4.1869	0.015666	1.805	0.40068	high-female - high-male; low-female - high-female; high-female - low-male; low-female - high-male; low-male - high-male; low-female - low-male	23225	23225_at	HGNC:30052
196	CD93	CD93 molecule	4.1839	0.01571	1.8038	0.40068	high-male - high-female; low-female - high-female; low-male - high-female; high-male - low-female; high-male - low-male; low-male - low-female	22918	22918_at	HGNC:15855
197	GBGT1	globoside alpha-1,3-N-acetylgalactosaminyltransferase 1 (FORS blood group)	4.1712	0.015898	1.7987	0.40278	high-male - high-female; low-female - high-female; low-male - high-female; high-male - low-female; high-male - low-male; low-male - low-female	26301	26301_at	HGNC:20460
198	PLIN2	perilipin 2	4.1675	0.015953	1.7972	0.40278	high-male - high-female; low-female - high-female; low-male - high-female; low-female - high-male; high-male - low-male; low-female - low-male	123	123_at	HGNC:248
199	G6PC3	glucose-6-phosphatase catalytic subunit 3	4.1606	0.016056	1.7944	0.4033	high-male - high-female; high-female - low-female; low-male - high-female; high-male - low-female; high-male - low-male; low-male - low-female	92579	92579_at	HGNC:24861

200	H2BC7	H2B clustered histone 7	4.1553	0.016135	1.7922	0.4033	high-male - high-female; low-female - high-female; high-female - low-male; high-male - low-female; high-male - low-male; low-female - low-male	8343	8343_at	HGNC:4752
201	ACP5	acid phosphatase 5, tartrate resistant	4.1436	0.016314	1.7874	0.40573	high-male - high-female; low-female - high-female; low-male - high-female; high-male - low-female; high-male - low-male; low-female - low-male	54	54_at	HGNC:124
202	SNORD116-29	small nucleolar RNA, C/D box 116-29	4.1345	0.016453	1.7837	0.4068	high-female - high-male; low-female - high-female; high-female - low-male; low-female - high-male; high-male - low-male; low-female - low-male	100033821	100033821_at	HGNC:33348
203	PTGDR2	prostaglandin D2 receptor 2	4.127	0.016569	1.7807	0.4068	high-male - high-female; low-female - high-female; low-male - high-female; high-male - low-female; low-male - high-male; low-male - low-female	11251	11251_at	HGNC:4502
204	ZNF107	zinc finger protein 107	4.125	0.016601	1.7799	0.4068	high-female - high-male; low-female - high-female; low-male - high-female; low-female - high-male; low-male - high-male; low-female - low-male	51427	51427_at	HGNC:12887
205	ZNF678	zinc finger protein 678	4.0894	0.017165	1.7654	0.41764	high-female - high-male; low-female - high-female; low-male - high-female; low-female - high-male; low-male - high-male; low-female - low-male	339500	339500_at	HGNC:28652
206	FAM118A	family with sequence similarity 118 member A	4.0795	0.017324	1.7614	0.41764	high-male - high-female; high-female - low-female; low-male - high-female; high-male - low-female; high-male - low-male; low-male - low-female	55007	55007_at	HGNC:1313
207	ABITRAM	actin binding transcription modulator	4.077	0.017365	1.7603	0.41764	high-male - high-female; low-female - high-female; low-male - high-female; high-male - low-female; high-male - low-male; low-male - low-female	54942	54942_at	HGNC:1364

208	ALDH2	aldehyde dehydrogenase 2 family member	4.0763	0.017377	1.76	0.41764	high-male - high-female; high-female - low-female; low-male - high-female; high-male - low-female; high-male - low-male; low-male - low-female	217	217_at	HGNC:404
209	RRAS2	RAS related 2	4.0678	0.017516	1.7566	0.41895	high-male - high-female; low-female - high-female; high-female - low-male; low-female - high-male; high-male - low-male; low-female - low-male	22800	22800_at	HGNC:17271
210	ATP6V0B	ATPase H+ transporting V0 subunit b	4.0467	0.017867	1.7479	0.42422	high-male - high-female; high-female - low-female; high-female - low-male; high-male - low-female; high-male - low-male; low-male - low-female	533	533_at	HGNC:861
211	CNTLN	centlein	4.0431	0.017929	1.7464	0.42422	high-male - high-female; high-female - low-female; low-male - high-female; high-male - low-female; high-male - low-male; low-male - low-female	54875	54875_at	HGNC:23432
212	UGDH	UDP-glucose 6-dehydrogenase	4.0367	0.018037	1.7438	0.42422	high-male - high-female; low-female - high-female; low-male - high-female; high-male - low-female; high-male - low-male; low-female - low-male	7358	7358_at	HGNC:12525
213	PAK1	p21 (RAC1) activated kinase 1	4.0344	0.018075	1.7429	0.42422	high-male - high-female; high-female - low-female; low-male - high-female; high-male - low-female; high-male - low-male; low-male - low-female	5058	5058_at	HGNC:8590
214	SHQ1	SHQ1, H/ACA ribonucleoprotein assembly factor	4.0269	0.018204	1.7398	0.42525	high-male - high-female; low-female - high-female; high-female - low-male; low-female - high-male; high-male - low-male; low-female - low-male	55164	55164_at	HGNC:25543
215	HLA-DMB	major histocompatibility complex, class II, DM beta	4.0112	0.018476	1.7334	0.42867	high-male - high-female; low-female - high-female; low-male - high-female; high-male - low-female; high-male - low-male; low-male - low-female	3109	3109_at	HGNC:4935

216	ZNF468	zinc finger protein 468	4.0086	0.018522	1.7323	0.42867	high-male - high-female; low-female - high-female; high-female - low-male; low-female - high-male; high-male - low-male; low-female - low-male	90333	90333_at	HGNC:33105
217	RNASE6	ribonuclease A family member k6	3.9921	0.018812	1.7256	0.43338	high-male - high-female; low-female - high-female; low-male - high-female; high-male - low-female; high-male - low-male; low-male - low-female	6039	6039_at	HGNC:10048
218	MIS18BP1	MIS18 binding protein 1	3.98	0.019028	1.7206	0.43368	high-male - high-female; low-female - high-female; low-male - high-female; high-male - low-female; high-male - low-male; low-male - low-female	55320	55320_at	HGNC:20190
219	GRAP	GRB2 related adaptor protein	3.9793	0.019041	1.7203	0.43368	high-male - high-female; low-female - high-female; low-male - high-female; low-female - high-male; high-male - low-male; low-female - low-male	10750	10750_at	HGNC:4562
220	MAP10	microtubule associated protein 10	3.9769	0.019086	1.7193	0.43368	high-male - high-female; low-female - high-female; low-male - high-female; low-female - high-male; high-male - low-male; low-female - low-male	54627	54627_at	HGNC:29265
221	CD86	CD86 molecule	3.9658	0.019286	1.7147	0.43497	high-male - high-female; high-female - low-female; low-male - high-female; high-male - low-female; high-male - low-male; low-male - low-female	942	942_at	HGNC:1705
222	BLVRB	biliverdin reductase B	3.9545	0.019494	1.7101	0.43497	high-male - high-female; low-female - high-female; low-male - high-female; high-male - low-female; high-male - low-male; low-male - low-female	645	645_at	HGNC:1063
223	NPC2	NPC intracellular cholesterol transporter 2	3.953	0.019522	1.7095	0.43497	high-male - high-female; high-female - low-female; low-male - high-female; high-male - low-female; high-male - low-male; low-male - low-female	10577	10577_at	HGNC:14537

224	DDX55	DEAD-box helicase 55	3.953	0.019523	1.7095	0.43497	high-female - high-male; low-female - high-female; low-male - high-female; low-female - high-male; low-male - high-male; low-female - low-male	57696	57696_at	HGNC:20085
225	BCAT1	branched chain amino acid transaminase 1	3.9281	0.01999	1.6992	0.43497	high-male - high-female; low-female - high-female; low-male - high-female; low-female - high-male; high-male - low-male; low-female - low-male	586	586_at	HGNC:976
226	SIGLEC6	sialic acid binding Ig like lectin 6	3.9268	0.020013	1.6987	0.43497	high-male - high-female; low-female - high-female; high-female - low-male; low-female - high-male; high-male - low-male; low-female - low-male	946	946_at	HGNC:10875
227	TBC1D31	TBC1 domain family member 31	3.9254	0.020039	1.6981	0.43497	high-male - high-female; low-female - high-female; low-male - high-female; low-female - high-male; high-male - low-male; low-female - low-male	93594	93594_at	HGNC:30888
228	SKP1	S-phase kinase associated protein 1	3.9249	0.02005	1.6979	0.43497	high-male - high-female; high-female - low-female; high-female - low-male; high-male - low-female; high-male - low-male; low-male - low-female	6500	6500_at	HGNC:10899
229	ZNF426	zinc finger protein 426	3.9192	0.020159	1.6955	0.43497	high-male - high-female; low-female - high-female; low-male - high-female; low-female - high-male; low-male - high-male; low-female - low-male	79088	79088_at	HGNC:20725
230	ZNF486	zinc finger protein 486	3.9137	0.020265	1.6933	0.43497	high-male - high-female; low-female - high-female; high-female - low-male; low-female - high-male; high-male - low-male; low-female - low-male	90649	90649_at	HGNC:20807
231	CD1C	CD1c molecule	3.9096	0.020343	1.6916	0.43497	high-male - high-female; low-female - high-female; low-male - high-female; high-male - low-female; low-male - high-male; low-male - low-female	911	911_at	HGNC:1636

232	KCNQ5	potassium voltage-gated channel subfamily Q member 5	3.9078	0.020379	1.6908	0.43497	high-female - high-male; low-female - high-female; low-male - high-female; low-female - high-male; low-male - high-male; low-female - low-male	56479	56479_at	HGNC:6299
233	RPL31	ribosomal protein L31	3.9065	0.020403	1.6903	0.43497	high-male - high-female; low-female - high-female; low-male - high-female; low-female - high-male; low-male - high-male; low-female - low-male	6160	6160_at	HGNC:10334
234	SMC6	structural maintenance of chromosomes 6	3.9046	0.02044	1.6895	0.43497	high-male - high-female; low-female - high-female; low-male - high-female; low-female - high-male; low-male - high-male; low-female - low-male	79677	79677_at	HGNC:20466
235	PLXND1	plexin D1	3.9034	0.020465	1.689	0.43497	high-male - high-female; low-female - high-female; low-male - high-female; high-male - low-female; high-male - low-male; low-female - low-male	23129	23129_at	HGNC:9107
236	ABHD12	abhydrolase domain containing 12	3.8924	0.020679	1.6845	0.43497	high-male - high-female; low-female - high-female; low-male - high-female; high-male - low-female; high-male - low-male; low-female - low-male	26090	26090_at	HGNC:15868
237	FGL2	fibrinogen like 2	3.8908	0.02071	1.6838	0.43497	high-male - high-female; high-female - low-female; low-male - high-female; high-male - low-female; high-male - low-male; low-male - low-female	10875	10875_at	HGNC:3696
238	CRTAP	cartilage associated protein	3.8873	0.02078	1.6824	0.43497	high-male - high-female; low-female - high-female; low-male - high-female; high-male - low-female; high-male - low-male; low-male - low-female	10491	10491_at	HGNC:2379
239	CD1D	CD1d molecule	3.8859	0.020806	1.6818	0.43497	high-male - high-female; low-female - high-female; low-male - high-female; high-male - low-female; high-male - low-male; low-male - low-female	912	912_at	HGNC:1637

240	TPRA1	transmembrane protein adipocyte associated 1	3.8783	0.020959	1.6786	0.43497	high-male - high-female; low-female - high-female; low-male - high-female; high-male - low-female; high-male - low-male; low-male - low-female	131601	131601_at	HGNC:30413
241	BNIP3	BCL2 interacting protein 3	3.8777	0.02097	1.6784	0.43497	high-male - high-female; low-female - high-female; high-female - low-male; low-female - high-male; high-male - low-male; low-female - low-male	664	664_at	HGNC:1084
242	NUP205	nucleoporin 205	3.8618	0.02129	1.6718	0.43722	high-male - high-female; low-female - high-female; low-male - high-female; low-female - high-male; low-male - high-male; low-female - low-male	23165	23165_at	HGNC:18658
243	AKTIP	AKT interacting protein	3.8583	0.021361	1.6704	0.43722	high-male - high-female; low-female - high-female; low-male - high-female; low-female - high-male; low-male - high-male; low-female - low-male	64400	64400_at	HGNC:16710
244	MCTP1	multiple C2 and transmembrane domain containing 1	3.8563	0.021403	1.6695	0.43722	high-male - high-female; high-female - low-female; low-male - high-female; high-male - low-female; high-male - low-male; low-male - low-female	79772	79772_at	HGNC:26183
245	ITK	IL2 inducible T cell kinase	3.8486	0.02156	1.6663	0.43722	high-female - high-male; high-female - low-female; high-female - low-male; low-female - high-male; low-male - high-male; low-female - low-male	3702	3702_at	HGNC:6171
246	COBLL1	cordon-bleu WH2 repeat protein like 1	3.8394	0.021751	1.6625	0.43722	high-male - high-female; low-female - high-female; low-male - high-female; low-female - high-male; low-male - high-male; low-female - low-male	22837	22837_at	HGNC:23571
247	NT5E	5'-nucleotidase ecto	3.8386	0.021766	1.6622	0.43722	high-female - high-male; low-female - high-female; low-male - high-female; low-female - high-male; low-male - high-male; low-female - low-male	4907	4907_at	HGNC:8021

248	ZHX2	zinc fingers and homeoboxes 2	3.8376	0.021787	1.6618	0.43722	high-female - high-male; low-female - high-female; low-male - high-female; low-female - high-male; low-male - high-male; low-female - low-male	22882	22882_at	HGNC:18513
249	ZNF626	zinc finger protein 626	3.8363	0.021816	1.6612	0.43722	high-male - high-female; low-female - high-female; high-female - low-male; low-female - high-male; high-male - low-male; low-female - low-male	199777	199777_at	HGNC:30461
250	FHL3	four and a half LIM domains 3	3.8339	0.021865	1.6602	0.43722	high-male - high-female; high-female - low-female; low-male - high-female; high-male - low-female; high-male - low-male; low-male - low-female	2275	2275_at	HGNC:3704
251	TRAF5	TNF receptor associated factor 5	3.8282	0.021984	1.6579	0.43748	high-female - high-male; low-female - high-female; low-male - high-female; low-female - high-male; low-male - high-male; low-female - low-male	7188	7188_at	HGNC:12035
252	UBE2A	ubiquitin conjugating enzyme E2 A	3.8205	0.022146	1.6547	0.43748	high-male - high-female; low-female - high-female; low-male - high-female; high-male - low-female; high-male - low-male; low-male - low-female	7319	7319_at	HGNC:12472
253	NAAA	N-acylethanolamine acid amidase	3.8152	0.02226	1.6525	0.43748	high-male - high-female; low-female - high-female; low-male - high-female; high-male - low-female; high-male - low-male; low-male - low-female	27163	27163_at	HGNC:736
254	SAT2	spermidine/spermine N1-acetyltransferase family member 2	3.8089	0.022393	1.6499	0.43748	high-male - high-female; low-female - high-female; low-male - high-female; high-male - low-female; high-male - low-male; low-female - low-male	112483	112483_at	HGNC:23160
255	PMS2P5	PMS1 homolog 2, mismatch repair system component pseudogene 5	3.8077	0.022419	1.6494	0.43748	high-male - high-female; low-female - high-female; low-male - high-female; low-female - high-male; low-male - high-male; low-female - low-male	5383	5383_at	HGNC:9130

256	PLCB1	phospholipase C beta 1	3.8046	0.022487	1.6481	0.43748	high-male - high-female; low-female - high-female; low-male - high-female; high-male - low-female; high-male - low-male; low-male - low-female	23236	23236_at	HGNC:15917
257	QARS1	glutaminyl-tRNA synthetase 1	3.8044	0.022491	1.648	0.43748	high-male - high-female; high-female - low-female; low-male - high-female; high-male - low-female; high-male - low-male; low-male - low-female	5859	5859_at	HGNC:9751
258	HIGD1A	HIG1 hypoxia inducible domain family member 1A	3.7944	0.022708	1.6438	0.43999	high-male - high-female; high-female - low-female; high-female - low-male; high-male - low-female; high-male - low-male; low-female - low-male	25994	25994_at	HGNC:29527
259	H2AC13	H2A clustered histone 13	3.7697	0.023252	1.6335	0.44698	high-male - high-female; low-female - high-female; high-female - low-male; low-female - high-male; high-male - low-male; low-female - low-male	8329	8329_at	HGNC:4725
260	MBD4	methyl-CpG binding domain 4, DNA glycosylase	3.7674	0.023302	1.6326	0.44698	high-male - high-female; low-female - high-female; low-male - high-female; low-female - high-male; low-male - high-male; low-female - low-male	8930	8930_at	HGNC:6919
261	GRK3	G protein-coupled receptor kinase 3	3.7658	0.023337	1.632	0.44698	high-male - high-female; low-female - high-female; low-male - high-female; high-male - low-female; high-male - low-male; low-male - low-female	157	157_at	HGNC:290
262	GGTA1P	glycoprotein alpha-galactosyltransferase 1, pseudogene	3.7536	0.023612	1.6269	0.44873	high-male - high-female; high-female - low-female; high-female - low-male; high-male - low-female; high-male - low-male; low-male - low-female	2681	2681_at	HGNC:4253
263	NCF2	neutrophil cytosolic factor 2	3.7494	0.023708	1.6251	0.44873	high-male - high-female; high-female - low-female; low-male - high-female; high-male - low-female; high-male - low-male; low-male - low-female	4688	4688_at	HGNC:7661

264	NDFIP2	Nedd4 family interacting protein 2	3.7462	0.023783	1.6237	0.44873	high-male - high-female; low-female - high-female; low-male - high-female; low-female - high-male; high-male - low-male; low-female - low-male	54602	54602_at	HGNC:18537
265	CD27	CD27 molecule	3.7423	0.02387	1.6221	0.44873	high-female - high-male; low-female - high-female; high-female - low-male; low-female - high-male; low-male - high-male; low-female - low-male	939	939_at	HGNC:11922
266	GTF2H1	general transcription factor IIH subunit 1	3.742	0.023877	1.622	0.44873	high-male - high-female; low-female - high-female; low-male - high-female; high-male - low-female; high-male - low-male; low-female - low-male	2965	2965_at	HGNC:4655
267	C1D	C1D nuclear receptor corepressor	3.7307	0.024138	1.6173	0.45163	high-male - high-female; low-female - high-female; low-male - high-female; high-male - low-female; high-male - low-male; low-female - low-male	10438	10438_at	HGNC:29911
268	TBC1D10C	TBC1 domain family member 10C	3.7256	0.024257	1.6152	0.45163	high-female - high-male; low-female - high-female; low-male - high-female; low-female - high-male; low-male - high-male; low-female - low-male	374403	374403_at	HGNC:24702
269	DPH5	diphthamide biosynthesis 5	3.7199	0.024389	1.6128	0.45163	high-female - high-male; low-female - high-female; high-female - low-male; low-female - high-male; low-male - high-male; low-female - low-male	51611	51611_at	HGNC:24270
270	PLPP1	phospholipid phosphatase 1	3.7108	0.024604	1.609	0.45163	high-female - high-male; low-female - high-female; high-female - low-male; low-female - high-male; low-male - high-male; low-female - low-male	8611	8611_at	HGNC:9228
271	DNAJC8	DnaJ heat shock protein family (Hsp40) member C8	3.7088	0.024652	1.6081	0.45163	high-male - high-female; low-female - high-female; low-male - high-female; high-male - low-female; high-male - low-male; low-female - low-male	22826	22826_at	HGNC:15470

272	SLC25A19	solute carrier family 25 member 19	3.7051	0.024739	1.6066	0.45163	high-male - high-female; low-female - high-female; low-male - high-female; high-male - low-female; high-male - low-male; low-male - low-female	60386	60386_at	HGNC:14409
273	PCNA	proliferating cell nuclear antigen	3.703	0.024792	1.6057	0.45163	high-female - high-male; low-female - high-female; high-female - low-male; low-female - high-male; high-male - low-male; low-female - low-male	5111	5111_at	HGNC:8729
274	ECRP	ribonuclease A family member 2 pseudogene	3.7012	0.024833	1.605	0.45163	high-male - high-female; low-female - high-female; low-male - high-female; high-male - low-female; high-male - low-male; low-male - low-female	643332	643332_at	
275	ZNF124	zinc finger protein 124	3.6942	0.025003	1.602	0.45163	high-male - high-female; low-female - high-female; low-male - high-female; high-male - low-female; high-male - low-male; low-female - low-male	7678	7678_at	HGNC:12907
276	CPPED1	calcineurin like phosphoesterase domain containing 1	3.6859	0.025204	1.5985	0.45163	high-male - high-female; high-female - low-female; low-male - high-female; high-male - low-female; high-male - low-male; low-male - low-female	55313	55313_at	HGNC:25632
277	TUBA1B	tubulin alpha 1b	3.6811	0.02532	1.5965	0.45163	high-male - high-female; high-female - low-female; high-female - low-male; high-male - low-female; high-male - low-male; low-female - low-male	10376	10376_at	HGNC:18809
278	PKP2	plakophilin 2	3.6777	0.025402	1.5951	0.45163	high-male - high-female; high-female - low-female; high-female - low-male; high-male - low-female; high-male - low-male; low-male - low-female	5318	5318_at	HGNC:9024
279	GOLPH3L	golgi phosphoprotein 3 like	3.6734	0.025509	1.5933	0.45163	high-female - high-male; low-female - high-female; high-female - low-male; low-female - high-male; low-male - high-male; low-female - low-male	55204	55204_at	HGNC:24882

280	SNORD49B	small nucleolar RNA, C/D box 49B	3.6722	0.025538	1.5928	0.45163	high-male - high-female; low-female - high-female; high-female - low-male; low-female - high-male; high-male - low-male; low-female - low-male	692087	692087_at	HGNC:32721
281	GAPT	GRB2 binding adaptor protein, transmembrane	3.6651	0.025713	1.5898	0.45163	high-male - high-female; low-female - high-female; low-male - high-female; high-male - low-female; high-male - low-male; low-male - low-female	202309	202309_at	HGNC:26588
282	FES	FES proto-oncogene, tyrosine kinase	3.6632	0.025761	1.589	0.45163	high-male - high-female; low-female - high-female; low-male - high-female; high-male - low-female; high-male - low-male; low-male - low-female	2242	2242_at	HGNC:3657
283	LARP1B	La ribonucleoprotein 1B	3.6549	0.025968	1.5856	0.45163	high-male - high-female; low-female - high-female; low-male - high-female; low-female - high-male; high-male - low-male; low-female - low-male	55132	55132_at	HGNC:24704
284	KLF13	Kruppel like factor 13	3.654	0.02599	1.5852	0.45163	high-male - high-female; low-female - high-female; low-male - high-female; low-female - high-male; low-male - high-male; low-female - low-male	51621	51621_at	HGNC:13672
285	PRLR	prolactin receptor	3.6508	0.026072	1.5838	0.45163	high-male - high-female; low-female - high-female; low-male - high-female; high-male - low-female; high-male - low-male; low-male - low-female	5618	5618_at	HGNC:9446
286	RRP15	ribosomal RNA processing 15 homolog	3.6484	0.026133	1.5828	0.45163	high-male - high-female; low-female - high-female; low-male - high-female; low-female - high-male; low-male - high-male; low-female - low-male	51018	51018_at	HGNC:24255
287	MT2A	metallothionein 2A	3.6473	0.026161	1.5823	0.45163	high-female - high-male; high-female - low-female; high-female - low-male; high-male - low-female; high-male - low-male; low-female - low-male	4502	4502_at	HGNC:7406

288	ZNF385A	zinc finger protein 385A	3.6436	0.026255	1.5808	0.45163	high-male - high-female; high-female - low-female; low-male - high-female; high-male - low-female; high-male - low-male; low-male - low-female	25946	25946_at	HGNC:17521
289	NUDT16P1	nudix hydrolase 16 pseudogene 1	3.6391	0.026368	1.5789	0.45163	high-male - high-female; high-female - low-female; low-male - high-female; high-male - low-female; high-male - low-male; low-male - low-female	152195	152195_at	HGNC:27189
290	FRY	FRY microtubule binding protein	3.6351	0.026471	1.5772	0.45163	high-male - high-female; high-female - low-female; low-male - high-female; high-male - low-female; low-male - high-male; low-male - low-female	10129	10129_at	HGNC:20367
291	ANK3	ankyrin 3	3.6329	0.026528	1.5763	0.45163	high-female - high-male; high-female - low-female; low-male - high-female; high-male - low-female; low-male - high-male; low-male - low-female	288	288_at	HGNC:494
292	NUP88	nucleoporin 88	3.6305	0.02659	1.5753	0.45163	high-male - high-female; low-female - high-female; low-male - high-female; low-female - high-male; low-male - high-male; low-female - low-male	4927	4927_at	HGNC:8067
293	RBIS	ribosomal biogenesis factor	3.6293	0.02662	1.5748	0.45163	high-male - high-female; low-female - high-female; low-male - high-female; high-male - low-female; high-male - low-male; low-male - low-female	401466	401466_at	HGNC:32235
294	CD33	CD33 molecule	3.6284	0.026642	1.5744	0.45163	high-male - high-female; high-female - low-female; low-male - high-female; high-male - low-female; high-male - low-male; low-male - low-female	945	945_at	HGNC:1659
295	DNAJC10	DnaJ heat shock protein family (Hsp40) member C10	3.6212	0.026828	1.5714	0.45163	high-male - high-female; low-female - high-female; low-male - high-female; low-female - high-male; low-male - high-male; low-female - low-male	54431	54431_at	HGNC:24637

296	PTGDS	prostaglandin D2 synthase	3.6207	0.026842	1.5712	0.45163	high-male - high-female; low-female - high-female; low-male - high-female; high-male - low-female; low-male - high-male; low-male - low-female	5730	5730_at	HGNC:9592
297	MAD2L1BP	MAD2L1 binding protein	3.6147	0.026998	1.5687	0.45163	high-male - high-female; low-female - high-female; low-male - high-female; high-male - low-female; high-male - low-male; low-female - low-male	9587	9587_at	HGNC:21059
298	SLC31A2	solute carrier family 31 member 2	3.6124	0.027058	1.5677	0.45163	high-male - high-female; high-female - low-female; high-female - low-male; high-male - low-female; high-male - low-male; low-male - low-female	1318	1318_at	HGNC:11017
299	CLIC2	chloride intracellular channel 2	3.6065	0.027214	1.5652	0.45163	high-male - high-female; high-female - low-female; low-male - high-female; high-male - low-female; high-male - low-male; low-male - low-female	1193	1193_at	HGNC:2063
300	FCGR2B	Fc fragment of IgG receptor IIb	3.6056	0.027238	1.5648	0.45163	high-male - high-female; low-female - high-female; low-male - high-female; low-female - high-male; high-male - low-male; low-female - low-male	2213	2213_at	HGNC:3618
301	C11orf65	chromosome 11 open reading frame 65	3.6016	0.027345	1.5631	0.45163	high-male - high-female; low-female - high-female; low-male - high-female; low-female - high-male; low-male - high-male; low-female - low-male	160140	160140_at	HGNC:28519
302	SUSD1	sushi domain containing 1	3.6	0.027386	1.5625	0.45163	high-male - high-female; high-female - low-female; high-female - low-male; high-male - low-female; high-male - low-male; low-male - low-female	64420	64420_at	HGNC:25413
303	PCID2	PCI domain containing 2	3.5964	0.027481	1.561	0.45163	high-female - high-male; low-female - high-female; high-female - low-male; low-female - high-male; low-male - high-male; low-female - low-male	55795	55795_at	HGNC:25653

304	CYP1B1	cytochrome P450 family 1 subfamily B member 1	3.5951	0.027515	1.5604	0.45163	high-male - high-female; low-female - high-female; low-male - high-female; high-male - low-female; high-male - low-male; low-female - low-male	1545	1545_at	HGNC:2597
305	TFEC	transcription factor EC	3.5937	0.027555	1.5598	0.45163	high-male - high-female; low-female - high-female; low-male - high-female; high-male - low-female; high-male - low-male; low-male - low-female	22797	22797_at	HGNC:11754
306	ACSS2	acyl-CoA synthetase short chain family member 2	3.5844	0.027804	1.5559	0.45273	high-male - high-female; low-female - high-female; low-male - high-female; high-male - low-female; high-male - low-male; low-male - low-female	55902	55902_at	HGNC:15814
307	TOMM34	translocase of outer mitochondrial membrane 34	3.5823	0.027861	1.555	0.45273	high-male - high-female; low-female - high-female; low-male - high-female; high-male - low-female; low-male - high-male; low-male - low-female	10953	10953_at	HGNC:15746
308	C4orf46	chromosome 4 open reading frame 46	3.5795	0.027937	1.5538	0.45273	high-male - high-female; low-female - high-female; high-female - low-male; high-male - low-female; high-male - low-male; low-female - low-male	201725	201725_at	HGNC:27320
309	LAMP3	lysosomal associated membrane protein 3	3.5777	0.027984	1.5531	0.45273	high-female - high-male; low-female - high-female; high-female - low-male; low-female - high-male; high-male - low-male; low-female - low-male	27074	27074_at	HGNC:14582
310	ABI3	ABI family member 3	3.5729	0.028116	1.551	0.45273	high-male - high-female; high-female - low-female; high-female - low-male; high-male - low-female; high-male - low-male; low-female - low-male	51225	51225_at	HGNC:29859
311	GOLIM4	golgi integral membrane protein 4	3.5711	0.028166	1.5503	0.45273	high-male - high-female; low-female - high-female; low-male - high-female; high-male - low-female; high-male - low-male; low-male - low-female	27333	27333_at	HGNC:15448

312	HEATR5B	HEAT repeat containing 5B	3.5569	0.028557	1.5443	0.45705	high-female - high-male; low-female - high-female; low-male - high-female; low-female - high-male; low-male - high-male; low-female - low-male	54497	54497_at	HGNC:29273
313	TRBV25-1	T cell receptor beta variable 25-1	3.5492	0.028772	1.541	0.45705	high-male - high-female; low-female - high-female; high-female - low-male; low-female - high-male; high-male - low-male; low-female - low-male	28562	28562_at	HGNC:12205
314	SLC49A4	solute carrier family 49 member 4	3.5439	0.028921	1.5388	0.45705	high-male - high-female; low-female - high-female; low-male - high-female; high-male - low-female; high-male - low-male; low-male - low-female	84925	84925_at	HGNC:16628
315	SNORA52	small nucleolar RNA, H/ACA box 52	3.5429	0.028949	1.5384	0.45705	high-male - high-female; low-female - high-female; high-female - low-male; low-female - high-male; high-male - low-male; low-female - low-male	619565	619565_at	HGNC:32645
316	ZNF724	zinc finger protein 724	3.5417	0.028983	1.5379	0.45705	high-male - high-female; low-female - high-female; high-female - low-male; low-female - high-male; high-male - low-male; low-female - low-male	440519	440519_at	HGNC:32460
317	TAS2R50	taste 2 receptor member 50	3.5367	0.029124	1.5357	0.45705	high-female - high-male; high-female - low-female; low-male - high-female; low-female - high-male; low-male - high-male; low-male - low-female	259296	259296_at	HGNC:18882
318	JDP2	Jun dimerization protein 2	3.5356	0.029155	1.5353	0.45705	high-male - high-female; low-female - high-female; low-male - high-female; high-male - low-female; low-male - high-male; low-male - low-female	122953	122953_at	HGNC:17546
319	SPECC1	sperm antigen with calponin homology and coiled-coil domains 1	3.5352	0.029166	1.5351	0.45705	high-male - high-female; low-female - high-female; low-male - high-female; high-male - low-female; high-male - low-male; low-male - low-female	92521	92521_at	HGNC:30615

320	DGKD	diacylglycerol kinase delta	3.5261	0.029427	1.5313	0.4597	high-male - high-female; low-female - high-female; low-male - high-female; low-female - high-male; low-male - high-male; low-female - low-male	8527	8527_at	HGNC:2851
321	CST3	cystatin C	3.5152	0.02974	1.5267	0.46315	high-male - high-female; high-female - low-female; low-male - high-female; high-male - low-female; high-male - low-male; low-male - low-female	1471	1471_at	HGNC:2475
322	FLNB	filamin B	3.5077	0.029959	1.5235	0.46511	high-female - high-male; low-female - high-female; low-male - high-female; low-female - high-male; low-male - high-male; low-female - low-male	2317	2317_at	HGNC:3755
323	TRGV9	T cell receptor gamma variable 9	3.4913	0.030441	1.5165	0.47088	high-female - high-male; high-female - low-female; high-female - low-male; low-female - high-male; low-male - high-male; low-female - low-male	6983	6983_at	HGNC:12295
324	PSEN1	presenilin 1	3.4887	0.030519	1.5154	0.47088	high-male - high-female; high-female - low-female; low-male - high-female; high-male - low-female; high-male - low-male; low-male - low-female	5663	5663_at	HGNC:9508
325	HSD17B12	hydroxysteroid 17-beta dehydrogenase 12	3.4832	0.030684	1.5131	0.47124	high-male - high-female; low-female - high-female; low-male - high-female; high-male - low-female; high-male - low-male; low-male - low-female	51144	51144_at	HGNC:18646
326	C1orf131	chromosome 1 open reading frame 131	3.4751	0.030926	1.5097	0.47124	high-male - high-female; low-female - high-female; low-male - high-female; high-male - low-female; high-male - low-male; low-female - low-male	128061	128061_at	HGNC:25332
327	MRPL19	mitochondrial ribosomal protein L19	3.4732	0.030986	1.5088	0.47124	high-male - high-female; low-female - high-female; low-male - high-female; low-female - high-male; high-male - low-male; low-female - low-male	9801	9801_at	HGNC:14052

328	REXO2	RNA exonuclease 2	3.4727	0.030999	1.5086	0.47124	high-female - high-male; low-female - high-female; high-female - low-male; low-female - high-male; low-male - high-male; low-female - low-male	25996	25996_at	HGNC:17851
329	SERPINB2	serpin family B member 2	3.4706	0.031064	1.5077	0.47124	high-male - high-female; low-female - high-female; low-male - high-female; high-male - low-female; high-male - low-male; low-male - low-female	5055	5055_at	HGNC:8584
330	GPR34	G protein-coupled receptor 34	3.4677	0.031151	1.5065	0.47124	high-male - high-female; low-female - high-female; low-male - high-female; high-male - low-female; high-male - low-male; low-female - low-male	2857	2857_at	HGNC:4490
331	ZNF597	zinc finger protein 597	3.4661	0.031203	1.5058	0.47124	high-male - high-female; low-female - high-female; low-male - high-female; high-male - low-female; high-male - low-male; low-female - low-male	146434	146434_at	HGNC:26573
332	TIMM29	translocase of inner mitochondrial membrane 29	3.4622	0.031321	1.5042	0.47161	high-male - high-female; low-female - high-female; low-male - high-female; low-female - high-male; low-male - high-male; low-female - low-male	90580	90580_at	HGNC:25152
333	KIF11	kinesin family member 11	3.4526	0.031617	1.5001	0.47277	high-female - high-male; low-female - high-female; high-female - low-male; low-female - high-male; high-male - low-male; low-female - low-male	3832	3832_at	HGNC:6388
334	SLC24A4	solute carrier family 24 member 4	3.4494	0.031716	1.4987	0.47277	high-male - high-female; low-female - high-female; low-male - high-female; high-male - low-female; high-male - low-male; low-male - low-female	123041	123041_at	HGNC:10978
335	SAP30L	SAP30 like	3.4489	0.031732	1.4985	0.47277	high-male - high-female; low-female - high-female; low-male - high-female; low-female - high-male; high-male - low-male; low-female - low-male	79685	79685_at	HGNC:25663

336	ADI1	acireductone dioxygenase 1	3.4464	0.031809	1.4974	0.47277	high-male - high-female; low-female - high-female; low-male - high-female; high-male - low-female; high-male - low-male; low-female - low-male	55256	55256_at	HGNC:30576
337	TAS2R19	taste 2 receptor member 19	3.4444	0.031871	1.4966	0.47277	high-female - high-male; low-female - high-female; low-male - high-female; low-female - high-male; low-male - high-male; low-male - low-female	259294	259294_at	HGNC:19108
338	CMPK2	cytidine/uridine monophosphate kinase 2	3.4405	0.031992	1.495	0.47316	high-male - high-female; low-female - high-female; high-female - low-male; low-female - high-male; high-male - low-male; low-female - low-male	129607	129607_at	HGNC:27015
339	COG8	component of oligomeric golgi complex 8	3.4202	0.032636	1.4863	0.47833	high-male - high-female; low-female - high-female; low-male - high-female; low-female - high-male; high-male - low-male; low-female - low-male	84342	84342_at	HGNC:18623
340	DOCK7	dedicator of cytokinesis 7	3.4164	0.032758	1.4847	0.47833	high-male - high-female; low-female - high-female; low-male - high-female; high-male - low-female; low-male - high-male; low-male - low-female	85440	85440_at	HGNC:19190
341	VEGFA	vascular endothelial growth factor A	3.4162	0.032765	1.4846	0.47833	high-male - high-female; low-female - high-female; low-male - high-female; high-male - low-female; high-male - low-male; low-male - low-female	7422	7422_at	HGNC:12680
342	C5orf15	chromosome 5 open reading frame 15	3.414	0.032836	1.4837	0.47833	high-male - high-female; low-female - high-female; low-male - high-female; high-male - low-female; high-male - low-male; low-female - low-male	56951	56951_at	HGNC:20656
343	SERPING1	serpin family G member 1	3.4133	0.032857	1.4834	0.47833	high-male - high-female; high-female - low-female; high-female - low-male; high-male - low-female; high-male - low-male; low-female - low-male	710	710_at	HGNC:1228

344	CBL	Cbl proto-oncogene	3.4115	0.032916	1.4826	0.47833	high-male - high-female; low-female - high-female; low-male - high-female; high-male - low-female; high-male - low-male; low-male - low-female	867	867_at	HGNC:1541
345	ACTA2	actin alpha 2, smooth muscle	3.401	0.033259	1.4781	0.47977	high-female - high-male; low-female - high-female; high-female - low-male; low-female - high-male; high-male - low-male; low-female - low-male	59	59_at	HGNC:130
346	RPL22L1	ribosomal protein L22 like 1	3.4003	0.033281	1.4778	0.47977	high-female - high-male; high-female - low-female; high-female - low-male; low-female - high-male; high-male - low-male; low-female - low-male	200916	200916_at	HGNC:27610
347	SLC38A11	solute carrier family 38 member 11	3.3992	0.033315	1.4774	0.47977	high-female - high-male; low-female - high-female; low-male - high-female; low-female - high-male; low-male - high-male; low-female - low-male	151258	151258_at	HGNC:26836
348	PLA2G7	phospholipase A2 group VII	3.3967	0.033399	1.4763	0.47977	high-male - high-female; low-female - high-female; low-male - high-female; high-male - low-female; high-male - low-male; low-male - low-female	7941	7941_at	HGNC:9040
349	FEN1	flap structure-specific endonuclease 1	3.3874	0.033704	1.4723	0.48199	high-female - high-male; low-female - high-female; high-female - low-male; low-female - high-male; high-male - low-male; low-female - low-male	2237	2237_at	HGNC:3650
350	CTNNBIP1	catenin beta interacting protein 1	3.3862	0.033746	1.4718	0.48199	high-male - high-female; low-female - high-female; low-male - high-female; high-male - low-female; high-male - low-male; low-female - low-male	56998	56998_at	HGNC:16913
351	PLCG1	phospholipase C gamma 1	3.3817	0.033893	1.4699	0.4827	high-female - high-male; low-female - high-female; low-male - high-female; low-female - high-male; low-male - high-male; low-female - low-male	5335	5335_at	HGNC:9065

352	ZNF17	zinc finger protein 17	3.3739	0.034156	1.4665	0.48507	high-male - high-female; low-female - high-female; low-male - high-female; low-female - high-male; high-male - low-male; low-female - low-male	7565	7565_at	HGNC:12958
353	GBA	glucosylceramidase beta	3.3688	0.034325	1.4644	0.4861	high-male - high-female; low-female - high-female; low-male - high-female; high-male - low-female; high-male - low-male; low-male - low-female	2629	2629_at	HGNC:4177
354	CCNA2	cyclin A2	3.3656	0.034435	1.463	0.48627	high-female - high-male; low-female - high-female; high-female - low-male; low-female - high-male; high-male - low-male; low-female - low-male	890	890_at	HGNC:1578
355	MIR29A	microRNA 29a	3.3598	0.034633	1.4605	0.48645	high-male - high-female; low-female - high-female; low-male - high-female; high-male - low-female; high-male - low-male; low-male - low-female	407021	407021_at	HGNC:31616
356	EGLN3	egl-9 family hypoxia inducible factor 3	3.3567	0.034737	1.4592	0.48645	high-male - high-female; low-female - high-female; low-male - high-female; high-male - low-female; high-male - low-male; low-female - low-male	112399	112399_at	HGNC:14661
357	QSOX1	quiescin sulfhydryl oxidase 1	3.3534	0.034852	1.4578	0.48645	high-male - high-female; low-female - high-female; low-male - high-female; high-male - low-female; high-male - low-male; low-female - low-male	5768	5768_at	HGNC:9756
358	ADARB1	adenosine deaminase RNA specific B1	3.3516	0.034912	1.457	0.48645	high-female - high-male; low-female - high-female; low-male - high-female; low-female - high-male; low-male - high-male; low-female - low-male	104	104_at	HGNC:226
359	IL13RA1	interleukin 13 receptor subunit alpha 1	3.351	0.034934	1.4568	0.48645	high-male - high-female; high-female - low-female; low-male - high-female; high-male - low-female; high-male - low-male; low-male - low-female	3597	3597_at	HGNC:5974

360	AP3S1	adaptor related protein complex 3 subunit sigma 1	3.3438	0.035183	1.4537	0.48679	high-male - high-female; high-female - low-female; high-female - low-male; high-male - low-female; high-male - low-male; low-male - low-female	1176	1176_at	HGNC:2013
361	SNORD116-4	small nucleolar RNA, C/D box 116-4	3.3425	0.035227	1.4531	0.48679	high-male - high-female; low-female - high-female; high-female - low-male; low-female - high-male; high-male - low-male; low-female - low-male	100033416	100033416_at	HGNC:33070
362	LY96	lymphocyte antigen 96	3.3401	0.035311	1.4521	0.48679	high-male - high-female; high-female - low-female; high-female - low-male; high-male - low-female; high-male - low-male; low-male - low-female	23643	23643_at	HGNC:17156
363	FCRL5	Fc receptor like 5	3.3391	0.035348	1.4516	0.48679	high-male - high-female; low-female - high-female; high-female - low-male; low-female - high-male; high-male - low-male; low-female - low-male	83416	83416_at	HGNC:18508
364	TMEM106B	transmembrane protein 106B	3.3294	0.035685	1.4475	0.48714	high-male - high-female; low-female - high-female; high-female - low-male; low-female - high-male; high-male - low-male; low-female - low-male	54664	54664_at	HGNC:22407
365	ACSS1	acyl-CoA synthetase short chain family member 1	3.3274	0.035756	1.4467	0.48714	high-female - high-male; low-female - high-female; low-male - high-female; low-female - high-male; low-male - high-male; low-female - low-male	84532	84532_at	HGNC:16091
366	SF3B4	splicing factor 3b subunit 4	3.3255	0.035823	1.4458	0.48714	high-male - high-female; low-female - high-female; low-male - high-female; high-male - low-female; high-male - low-male; low-female - low-male	10262	10262_at	HGNC:10771
367	PDIA4	protein disulfide isomerase family A member 4	3.3253	0.035832	1.4457	0.48714	high-female - high-male; low-female - high-female; high-female - low-male; low-female - high-male; high-male - low-male; low-female - low-male	9601	9601_at	HGNC:30167

368	PLD3	phospholipase D family member 3	3.3245	0.035861	1.4454	0.48714	high-male - high-female; low-female - high-female; low-male - high-female; high-male - low-female; high-male - low-male; low-male - low-female	23646	23646_at	HGNC:17158
369	SLC25A24	solute carrier family 25 member 24	3.3206	0.035996	1.4437	0.48729	high-male - high-female; low-female - high-female; low-male - high-female; high-male - low-female; high-male - low-male; low-male - low-female	29957	29957_at	HGNC:20662
370	DHFR2	dihydrofolate reductase 2	3.3166	0.036139	1.442	0.48729	high-male - high-female; low-female - high-female; high-female - low-male; low-female - high-male; high-male - low-male; low-female - low-male	200895	200895_at	HGNC:27309
371	ZNF28	zinc finger protein 28	3.3159	0.036164	1.4417	0.48729	high-male - high-female; low-female - high-female; low-male - high-female; low-female - high-male; high-male - low-male; low-female - low-male	7576	7576_at	HGNC:13073
372	ZNF770	zinc finger protein 770	3.31	0.036375	1.4392	0.48856	high-male - high-female; low-female - high-female; low-male - high-female; low-female - high-male; high-male - low-male; low-female - low-male	54989	54989_at	HGNC:26061
373	FAM13A	family with sequence similarity 13 member A	3.3078	0.036454	1.4383	0.48856	high-female - high-male; low-female - high-female; high-female - low-male; low-female - high-male; low-male - high-male; low-female - low-male	10144	10144_at	HGNC:19367
374	BCL11A	BAF chromatin remodeling complex subunit BCL11A	3.3034	0.036614	1.4363	0.4894	high-male - high-female; low-female - high-female; low-male - high-female; low-female - high-male; high-male - low-male; low-female - low-male	53335	53335_at	HGNC:13221
375	FAM118B	family with sequence similarity 118 member B	3.289	0.03714	1.4302	0.494	high-male - high-female; low-female - high-female; low-male - high-female; high-male - low-female; high-male - low-male; low-male - low-female	79607	79607_at	HGNC:26110

376	GUSBP5	GUSB pseudogene 5	3.2867	0.037223	1.4292	0.494	high-male - high-female; low-female - high-female; low-male - high-female; low-female - high-male; low-male - high-male; low-female - low-male	441046	441046_at	HGNC:42319
377	CD160	CD160 molecule	3.2859	0.037255	1.4288	0.494	high-female - high-male; low-female - high-female; high-female - low-male; low-female - high-male; high-male - low-male; low-female - low-male	11126	11126_at	HGNC:17013
378	RBM4B	RNA binding motif protein 4B	3.2825	0.037379	1.4274	0.49434	high-male - high-female; low-female - high-female; low-male - high-female; low-female - high-male; low-male - high-male; low-female - low-male	83759	83759_at	HGNC:28842
379	H1-5	H1.5 linker histone, cluster member	3.2788	0.037518	1.4258	0.49486	high-female - high-male; low-female - high-female; high-female - low-male; low-female - high-male; high-male - low-male; low-female - low-male	3009	3009_at	HGNC:4719
380	SPEF2	sperm flagellar 2	3.2705	0.037825	1.4222	0.49572	high-female - high-male; low-female - high-female; low-male - high-female; low-female - high-male; low-male - high-male; low-male - low-female	79925	79925_at	HGNC:26293
381	PEX1	peroxisomal biogenesis factor 1	3.2688	0.037888	1.4215	0.49572	high-female - high-male; low-female - high-female; low-male - high-female; low-female - high-male; low-male - high-male; low-female - low-male	5189	5189_at	HGNC:8850
382	SPOCD1	SPOC domain containing 1	3.267	0.037956	1.4207	0.49572	high-male - high-female; high-female - low-female; high-female - low-male; high-male - low-female; high-male - low-male; low-female - low-male	90853	90853_at	HGNC:26338
383	DLST	dihydrolipoamide S-succinyltransferase	3.2664	0.03798	1.4204	0.49572	high-male - high-female; low-female - high-female; low-male - high-female; low-female - high-male; low-male - high-male; low-male - low-female	1743	1743_at	HGNC:2911

384	ZNF780B	zinc finger protein 780B	3.2593	0.038247	1.4174	0.49735	high-female - high-male; low-female - high-female; low-male - high-female; low-female - high-male; low-male - high-male; low-female - low-male	163131	163131_at	HGNC:33109
385	HLTF	helicase like transcription factor	3.2565	0.038355	1.4162	0.49735	high-male - high-female; low-female - high-female; low-male - high-female; low-female - high-male; low-male - high-male; low-female - low-male	6596	6596_at	HGNC:11099
386	NHEJ1	non-homologous end joining factor 1	3.2552	0.038403	1.4156	0.49735	high-male - high-female; low-female - high-female; low-male - high-female; low-female - high-male; low-male - high-male; low-female - low-male	79840	79840_at	HGNC:25737
387	SMIM12	small integral membrane protein 12	3.2492	0.038632	1.4131	0.49902	high-male - high-female; low-female - high-female; high-female - low-male; high-male - low-female; high-male - low-male; low-female - low-male	113444	113444_at	HGNC:25154
388	CNR2	cannabinoid receptor 2	3.236	0.039144	1.4073	0.50386	high-male - high-female; low-female - high-female; low-male - high-female; low-female - high-male; low-male - high-male; low-female - low-male	1269	1269_at	HGNC:2160
389	RBMX	RNA binding motif protein X-linked	3.2336	0.039236	1.4063	0.50386	high-male - high-female; low-female - high-female; low-male - high-female; low-female - high-male; high-male - low-male; low-female - low-male	27316	27316_at	HGNC:9910
390	FEZ2	fasciculation and elongation protein zeta 2	3.2285	0.039434	1.4041	0.50386	high-male - high-female; low-female - high-female; low-male - high-female; high-male - low-female; high-male - low-male; low-male - low-female	9637	9637_at	HGNC:3660
391	ADTRP	androgen dependent TFPI regulating protein	3.2283	0.039443	1.404	0.50386	high-female - high-male; low-female - high-female; high-female - low-male; low-female - high-male; high-male - low-male; low-female - low-male	84830	84830_at	HGNC:21214

392	EIF2AK4	eukaryotic translation initiation factor 2 alpha kinase 4	3.2258	0.03954	1.403	0.50386	high-male - high-female; low-female - high-female; low-male - high-female; low-female - high-male; high-male - low-male; low-female - low-male	440275	440275_at	HGNC:19687
393	ZNF506	zinc finger protein 506	3.2223	0.039678	1.4015	0.50386	high-female - high-male; low-female - high-female; low-male - high-female; low-female - high-male; low-male - high-male; low-female - low-male	440515	440515_at	HGNC:23780
394	TMC6	transmembrane channel like 6	3.2214	0.039712	1.4011	0.50386	high-female - high-male; low-female - high-female; low-male - high-female; low-female - high-male; low-male - high-male; low-female - low-male	11322	11322_at	HGNC:18021
395	ARHGEF18	Rho/Rac guanine nucleotide exchange factor 18	3.2121	0.040082	1.397	0.50727	high-female - high-male; low-female - high-female; low-male - high-female; low-female - high-male; low-male - high-male; low-female - low-male	23370	23370_at	HGNC:17090
396	H4C13	H4 clustered histone 13	3.2093	0.040193	1.3959	0.50738	high-male - high-female; low-female - high-female; high-female - low-male; low-female - high-male; high-male - low-male; low-female - low-male	8368	8368_at	HGNC:4791
397	TSR3	TSR3 ribosome maturation factor	3.2046	0.040383	1.3938	0.50849	high-male - high-female; low-female - high-female; high-female - low-male; low-female - high-male; high-male - low-male; low-female - low-male	115939	115939_at	HGNC:14175
398	CLCN4	chloride voltage-gated channel 4	3.1998	0.040575	1.3917	0.50964	high-male - high-female; low-female - high-female; low-male - high-female; low-female - high-male; high-male - low-male; low-female - low-male	1183	1183_at	HGNC:2022
399	MPV17L2	MPV17 mitochondrial inner membrane protein like 2	3.1969	0.040693	1.3905	0.50964	high-male - high-female; low-female - high-female; low-male - high-female; high-male - low-female; high-male - low-male; low-female - low-male	84769	84769_at	HGNC:28177

400	HK2	hexokinase 2	3.1948	0.040779	1.3896	0.50964	high-male - high-female; low-female - high-female; low-male - high-female; high-male - low-female; high-male - low-male; low-male - low-female	3099	3099_at	HGNC:4923
401	CEP170	centrosomal protein 170	3.1825	0.041281	1.3843	0.51154	high-male - high-female; low-female - high-female; low-male - high-female; high-male - low-female; high-male - low-male; low-female - low-male	9859	9859_at	HGNC:28920
402	H2BC14	H2B clustered histone 14	3.1812	0.041335	1.3837	0.51154	high-female - high-male; high-female - low-female; high-female - low-male; low-female - high-male; high-male - low-male; low-female - low-male	8342	8342_at	HGNC:4750
403	KLHL5	kelch like family member 5	3.178	0.041465	1.3823	0.51154	high-female - high-male; high-female - low-female; high-female - low-male; high-male - low-female; low-male - high-male; low-male - low-female	51088	51088_at	HGNC:6356
404	BHLHE41	basic helix-loop-helix family member e41	3.1767	0.041519	1.3818	0.51154	high-female - high-male; low-female - high-female; high-female - low-male; low-female - high-male; low-male - high-male; low-female - low-male	79365	79365_at	HGNC:16617
405	POLR2E	RNA polymerase II subunit E	3.1767	0.041519	1.3817	0.51154	high-male - high-female; low-female - high-female; high-female - low-male; high-male - low-female; high-male - low-male; low-female - low-male	5434	5434_at	HGNC:9192
406	SNX30	sorting nexin family member 30	3.1761	0.041545	1.3815	0.51154	high-male - high-female; low-female - high-female; low-male - high-female; high-male - low-female; low-male - high-male; low-male - low-female	401548	401548_at	HGNC:23685
407	GBP5	guanylate binding protein 5	3.1699	0.041801	1.3788	0.51184	high-female - high-male; low-female - high-female; high-female - low-male; low-female - high-male; low-male - high-male; low-female - low-male	115362	115362_at	HGNC:19895

408	SKAP2	src kinase associated phosphoprotein 2	3.1697	0.04181	1.3787	0.51184	high-male - high-female; low-female - high-female; low-male - high-female; high-male - low-female; high-male - low-male; low-male - low-female	8935	8935_at	HGNC:15687
409	TPMT	thiopurine S-methyltransferase	3.1681	0.041877	1.378	0.51184	high-male - high-female; high-female - low-female; low-male - high-female; high-male - low-female; high-male - low-male; low-male - low-female	7172	7172_at	HGNC:12014
410	PRMT7	protein arginine methyltransferase 7	3.1595	0.04224	1.3743	0.51404	high-female - high-male; low-female - high-female; low-male - high-female; low-female - high-male; low-male - high-male; low-female - low-male	54496	54496_at	HGNC:25557
411	SNORD116-24	small nucleolar RNA, C/D box 116-24	3.1589	0.042263	1.374	0.51404	high-female - high-male; low-female - high-female; high-female - low-male; low-female - high-male; high-male - low-male; low-female - low-male	100033435	100033435_at	HGNC:33090
412	USP6NL	USP6 N-terminal like	3.142	0.042982	1.3667	0.52062	high-male - high-female; low-female - high-female; low-male - high-female; low-female - high-male; high-male - low-male; low-female - low-male	9712	9712_at	HGNC:16858
413	GFI1	growth factor independent 1 transcriptional repressor	3.1394	0.043095	1.3656	0.52062	high-female - high-male; low-female - high-female; high-female - low-male; low-female - high-male; high-male - low-male; low-female - low-male	2672	2672_at	HGNC:4237
414	PPIA	peptidylprolyl isomerase A	3.1365	0.043219	1.3643	0.52062	high-male - high-female; low-female - high-female; low-male - high-female; low-female - high-male; high-male - low-male; low-female - low-male	5478	5478_at	HGNC:9253
415	DDHD2	DDHD domain containing 2	3.1353	0.043269	1.3638	0.52062	high-male - high-female; low-female - high-female; low-male - high-female; low-female - high-male; low-male - high-male; low-female - low-male	23259	23259_at	HGNC:29106

416	TCN2	transcobalamin 2	3.1341	0.043324	1.3633	0.52062	high-male - high-female; low-female - high-female; low-male - high-female; high-male - low-female; high-male - low-male; low-male - low-female	6948	6948_at	HGNC:11653
417	CD40LG	CD40 ligand	3.1311	0.043451	1.362	0.52089	high-female - high-male; low-female - high-female; high-female - low-male; low-female - high-male; high-male - low-male; low-female - low-male	959	959_at	HGNC:11935
418	ZNF615	zinc finger protein 615	3.127	0.043633	1.3602	0.52182	high-male - high-female; low-female - high-female; low-male - high-female; high-male - low-female; high-male - low-male; low-female - low-male	284370	284370_at	HGNC:24740
419	OSGEPL1	O-sialoglycoprotein endopeptidase like 1	3.1203	0.043924	1.3573	0.52285	high-female - high-male; low-female - high-female; low-male - high-female; low-female - high-male; low-male - high-male; low-female - low-male	64172	64172_at	HGNC:23075
420	MFSD1	major facilitator superfamily domain containing 1	3.1168	0.044079	1.3558	0.52285	high-male - high-female; high-female - low-female; high-female - low-male; high-male - low-female; high-male - low-male; low-male - low-female	64747	64747_at	HGNC:25874
421	NCEH1	neutral cholesterol ester hydrolase 1	3.1126	0.044263	1.354	0.52285	high-male - high-female; low-female - high-female; low-male - high-female; high-male - low-female; high-male - low-male; low-male - low-female	57552	57552_at	HGNC:29260
422	TIMP1	TIMP metallopeptidase inhibitor 1	3.1069	0.044517	1.3515	0.52285	high-male - high-female; high-female - low-female; high-female - low-male; high-male - low-female; high-male - low-male; low-male - low-female	7076	7076_at	HGNC:11820
423	FAM214A	family with sequence similarity 214 member A	3.1042	0.044638	1.3503	0.52285	high-female - high-male; low-female - high-female; low-male - high-female; low-female - high-male; low-male - high-male; low-female - low-male	56204	56204_at	HGNC:25609

424	PAPSS2	3'-phosphoadenosine 5'-phosphosulfate synthase 2	3.1032	0.044682	1.3499	0.52285	high-male - high-female; high-female - low-female; high-female - low-male; high-male - low-female; high-male - low-male; low-male - low-female	9060	9060_at	HGNC:8604
425	C9orf64	chromosome 9 open reading frame 64	3.1021	0.044733	1.3494	0.52285	high-male - high-female; low-female - high-female; low-male - high-female; high-male - low-female; high-male - low-male; low-male - low-female	84267	84267_at	HGNC:28144
426	CLUAP1	clusterin associated protein 1	3.102	0.044737	1.3493	0.52285	high-male - high-female; low-female - high-female; low-male - high-female; low-female - high-male; low-male - high-male; low-female - low-male	23059	23059_at	HGNC:19009
427	PRNP	prion protein	3.1011	0.044778	1.3489	0.52285	high-male - high-female; low-female - high-female; low-male - high-female; high-male - low-female; high-male - low-male; low-female - low-male	5621	5621_at	HGNC:9449
428	CD28	CD28 molecule	3.1001	0.04482	1.3485	0.52285	high-female - high-male; low-female - high-female; low-male - high-female; low-female - high-male; low-male - high-male; low-female - low-male	940	940_at	HGNC:1653
429	TYMS	thymidylate synthetase	3.0975	0.044937	1.3474	0.52285	high-female - high-male; low-female - high-female; high-female - low-male; low-female - high-male; high-male - low-male; low-female - low-male	7298	7298_at	HGNC:12441
430	PBX3	PBX homeobox 3	3.0967	0.044974	1.347	0.52285	high-male - high-female; low-female - high-female; low-male - high-female; high-male - low-female; high-male - low-male; low-male - low-female	5090	5090_at	HGNC:8634
431	MIR181B1	microRNA 181b-1	3.0904	0.045258	1.3443	0.52316	high-male - high-female; low-female - high-female; low-male - high-female; high-male - low-female; high-male - low-male; low-female - low-male	406955	406955_at	HGNC:31550

432	CWF19L1	CWF19 like cell cycle control factor 1	3.0865	0.045434	1.3426	0.52316	high-male - high-female; low-female - high-female; low-male - high-female; high-male - low-female; high-male - low-male; low-female - low-male	55280	55280_at	HGNC:25613
433	TAS2R10	taste 2 receptor member 10	3.0832	0.045588	1.3412	0.52316	high-female - high-male; low-female - high-female; low-male - high-female; low-female - high-male; low-male - high-male; low-male - low-female	50839	50839_at	HGNC:14918
434	TRGV3	T cell receptor gamma variable 3	3.081	0.045688	1.3402	0.52316	high-male - high-female; low-female - high-female; low-male - high-female; high-male - low-female; high-male - low-male; low-male - low-female	6976	6976_at	HGNC:12288
435	EIF2AK3	eukaryotic translation initiation factor 2 alpha kinase 3	3.0796	0.045753	1.3396	0.52316	high-male - high-female; low-female - high-female; low-male - high-female; low-female - high-male; high-male - low-male; low-female - low-male	9451	9451_at	HGNC:3255
436	PGAP1	post-GPI attachment to proteins inositol deacylase 1	3.0708	0.046155	1.3358	0.52316	high-female - high-male; low-female - high-female; high-female - low-male; low-female - high-male; low-male - high-male; low-female - low-male	80055	80055_at	HGNC:25712
437	MT1DP	metallothionein 1D, pseudogene	3.0704	0.046175	1.3356	0.52316	high-male - high-female; low-female - high-female; high-female - low-male; low-female - high-male; high-male - low-male; low-female - low-male	326343	326343_at	HGNC:7396
438	PSENEN	presenilin enhancer, gamma-secretase subunit	3.0668	0.046343	1.334	0.52316	high-male - high-female; high-female - low-female; high-female - low-male; high-male - low-female; high-male - low-male; low-male - low-female	55851	55851_at	HGNC:30100
439	H2AC17	H2A clustered histone 17	3.0634	0.0465	1.3325	0.52316	high-male - high-female; low-female - high-female; high-female - low-male; high-male - low-female; high-male - low-male; low-female - low-male	8336	8336_at	HGNC:4735

440	IFT57	intraflagellar transport 57	3.0601	0.046655	1.3311	0.52316	high-male - high-female; low-female - high-female; low-male - high-female; low-female - high-male; high-male - low-male; low-female - low-male	55081	55081_at	HGNC:17367
441	SLFN12	schlafen family member 12	3.0587	0.046721	1.3305	0.52316	high-male - high-female; low-female - high-female; low-male - high-female; high-male - low-female; high-male - low-male; low-male - low-female	55106	55106_at	HGNC:25500
442	TMEM150B	transmembrane protein 150B	3.0579	0.046756	1.3302	0.52316	high-male - high-female; low-female - high-female; low-male - high-female; high-male - low-female; high-male - low-male; low-female - low-male	284417	284417_at	HGNC:34415
443	YAE1	YAE1 maturation factor of ABCE1	3.0579	0.04676	1.3301	0.52316	high-male - high-female; low-female - high-female; low-male - high-female; high-male - low-female; high-male - low-male; low-female - low-male	57002	57002_at	HGNC:24857
444	LOC100128398	uncharacterized LOC100128398	3.0546	0.046911	1.3287	0.52316	high-male - high-female; low-female - high-female; low-male - high-female; low-female - high-male; low-male - high-male; low-female - low-male	100128398	100128398_at	
445	TSGA10	testis specific 10	3.0543	0.046928	1.3286	0.52316	high-male - high-female; low-female - high-female; low-male - high-female; low-female - high-male; high-male - low-male; low-female - low-male	80705	80705_at	HGNC:14927
446	GUSBP1	GUSB pseudogene 1	3.053	0.046989	1.328	0.52316	high-female - high-male; high-female - low-female; high-female - low-male; high-male - low-female; low-male - high-male; low-male - low-female	728411	728411_at	HGNC:13670
447	WDR89	WD repeat domain 89	3.0485	0.047199	1.3261	0.52316	high-female - high-male; low-female - high-female; high-female - low-male; low-female - high-male; low-male - high-male; low-female - low-male	112840	112840_at	HGNC:20489

448	ERMP1	endoplasmic reticulum metallopeptidase 1	3.0461	0.047313	1.325	0.52316	high-female - high-male; low-female - high-female; low-male - high-female; low-female - high-male; low-male - high-male; low-female - low-male	79956	79956_at	HGNC:23703
449	MRPL35	mitochondrial ribosomal protein L35	3.0456	0.04734	1.3248	0.52316	high-female - high-male; low-female - high-female; high-female - low-male; low-female - high-male; high-male - low-male; low-female - low-male	51318	51318_at	HGNC:14489
450	SAMM50	SAMM50 sorting and assembly machinery component	3.0453	0.047354	1.3246	0.52316	high-male - high-female; low-female - high-female; low-male - high-female; high-male - low-female; high-male - low-male; low-male - low-female	25813	25813_at	HGNC:24276
451	SNAPIN	SNAP associated protein	3.0452	0.04736	1.3246	0.52316	high-male - high-female; low-female - high-female; low-male - high-female; high-male - low-female; high-male - low-male; low-female - low-male	23557	23557_at	HGNC:17145
452	H3C12	H3 clustered histone 12	3.0429	0.047468	1.3236	0.52316	high-female - high-male; low-female - high-female; high-female - low-male; low-female - high-male; high-male - low-male; low-female - low-male	8356	8356_at	HGNC:4774
453	MYC	MYC proto-oncogene, bHLH transcription factor	3.0415	0.047533	1.323	0.52316	high-female - high-male; low-female - high-female; high-female - low-male; low-female - high-male; low-male - high-male; low-female - low-male	4609	4609_at	HGNC:7553
454	KYNU	kynureninase	3.0349	0.047853	1.3201	0.52316	high-male - high-female; low-female - high-female; low-male - high-female; high-male - low-female; high-male - low-male; low-male - low-female	8942	8942_at	HGNC:6469
455	PPIG	peptidylprolyl isomerase G	3.0334	0.047923	1.3195	0.52316	high-male - high-female; low-female - high-female; high-female - low-male; low-female - high-male; high-male - low-male; low-female - low-male	9360	9360_at	HGNC:14650

456	SNORD48	small nucleolar RNA, C/D box 48	3.0325	0.047966	1.3191	0.52316	high-female - high-male; low-female - high-female; high-female - low-male; low-female - high-male; low-male - high-male; low-female - low-male	26801	26801_at	HGNC:10188
457	METTL21A	methyltransferase like 21A	3.0298	0.048096	1.3179	0.52316	high-male - high-female; low-female - high-female; low-male - high-female; high-male - low-female; high-male - low-male; low-female - low-male	151194	151194_at	HGNC:30476
458	NEU1	neuraminidase 1	3.0298	0.048097	1.3179	0.52316	high-male - high-female; low-female - high-female; low-male - high-female; high-male - low-female; high-male - low-male; low-female - low-male	4758	4758_at	HGNC:7758
459	NOC3L	NOC3 like DNA replication regulator	3.0292	0.048123	1.3176	0.52316	high-male - high-female; low-female - high-female; low-male - high-female; low-female - high-male; low-male - high-male; low-female - low-male	64318	64318_at	HGNC:24034
460	JUP	junction plakoglobin	3.0289	0.04814	1.3175	0.52316	high-male - high-female; high-female - low-female; high-female - low-male; high-male - low-female; high-male - low-male; low-female - low-male	3728	3728_at	HGNC:6207
461	OSGIN2	oxidative stress induced growth inhibitor family member 2	3.0242	0.048369	1.3154	0.52361	high-male - high-female; low-female - high-female; low-male - high-female; low-female - high-male; high-male - low-male; low-female - low-male	734	734_at	HGNC:1355
462	GAS7	growth arrest specific 7	3.0144	0.048845	1.3112	0.52361	high-male - high-female; low-female - high-female; low-male - high-female; high-male - low-female; high-male - low-male; low-male - low-female	8522	8522_at	HGNC:4169
463	ZFP30	ZFP30 zinc finger protein	3.0139	0.048873	1.3109	0.52361	high-female - high-male; low-female - high-female; low-male - high-female; low-female - high-male; low-male - high-male; low-female - low-male	22835	22835_at	HGNC:29555

464	SIPA1L3	signal induced proliferation associated 1 like 3	3.0128	0.048927	1.3104	0.52361	high-male - high-female; low-female - high-female; low-male - high-female; low-female - high-male; low-male - high-male; low-female - low-male	23094	23094_at	HGNC:23801
465	SNORA80E	small nucleolar RNA, H/ACA box 80E	3.0113	0.049	1.3098	0.52361	high-female - high-male; low-female - high-female; high-female - low-male; low-female - high-male; high-male - low-male; low-female - low-male	677823	677823_at	HGNC:32635
466	RNF135	ring finger protein 135	3.0111	0.049011	1.3097	0.52361	high-male - high-female; low-female - high-female; low-male - high-female; high-male - low-female; high-male - low-male; low-male - low-female	84282	84282_at	HGNC:21158
467	PATJ	PATJ crumbs cell polarity complex component	3.0099	0.04907	1.3092	0.52361	high-female - high-male; low-female - high-female; low-male - high-female; low-female - high-male; low-male - high-male; low-female - low-male	10207	10207_at	HGNC:28881
468	DCLRE1C	DNA cross-link repair 1C	3.0041	0.049358	1.3066	0.52361	high-male - high-female; low-female - high-female; low-male - high-female; low-female - high-male; low-male - high-male; low-female - low-male	64421	64421_at	HGNC:17642
469	NEMP2	nuclear envelope integral membrane protein 2	3.0037	0.049376	1.3065	0.52361	high-male - high-female; low-female - high-female; low-male - high-female; low-female - high-male; low-male - high-male; low-female - low-male	100131211	100131211_at	HGNC:33700
470	IFIT5	interferon induced protein with tetratricopeptide repeats 5	3.0032	0.049404	1.3062	0.52361	high-male - high-female; low-female - high-female; high-female - low-male; low-female - high-male; high-male - low-male; low-female - low-male	24138	24138_at	HGNC:13328
471	CHPT1	choline phosphotransferase 1	3.0011	0.049507	1.3053	0.52361	high-male - high-female; low-female - high-female; low-male - high-female; high-male - low-female; low-male - high-male; low-male - low-female	56994	56994_at	HGNC:17852

472	BTLA	B and T lymphocyte associated	2.9987	0.049626	1.3043	0.52361	high-female - high-male; low-female - high-female; low-male - high-female; low-female - high-male; low-male - high-male; low-female - low-male	151888	151888_at	HGNC:21087
473	PLK1	polo like kinase 1	2.9982	0.049648	1.3041	0.52361	high-female - high-male; low-female - high-female; high-female - low-male; low-female - high-male; high-male - low-male; low-female - low-male	5347	5347_at	HGNC:9077
474	GPX1	glutathione peroxidase 1	2.9971	0.049708	1.3036	0.52361	high-male - high-female; low-female - high-female; low-male - high-female; high-male - low-female; high-male - low-male; low-male - low-female	2876	2876_at	HGNC:4553
475	RIC1	RIC1 homolog, RAB6A GEF complex partner 1	2.9927	0.049925	1.3017	0.52361	high-male - high-female; low-female - high-female; low-male - high-female; high-male - low-female; low-male - high-male; low-male - low-female	57589	57589_at	HGNC:17686
476	SCARNA5	small Cajal body-specific RNA 5	2.9916	0.04998	1.3012	0.52361	high-female - high-male; low-female - high-female; high-female - low-male; low-female - high-male; high-male - low-male; low-female - low-male	677775	677775_at	HGNC:32561
	Data2									
**S.No.**	**Symbol**	**Name**	**f.value**	**p.value**	**X.log10.p.**	**FDR**	**Fisher.s.LSD**	**SPOT_ID**	**REFSEQ**	**GB_ACC**

1	USP9Y	ubiquitin specific peptidase 9, Y-linked	13.335	2.55E-08	7.5936	0.00012743	High_Male - High_Female;<br>Low_Female - High_Female;<br>Low_Male - High_Female;<br>Medium_Female - High_Female;<br>Medium_Male - High_Female;<br>High_Male - Low_Female;<br>Low_Male - High_Male;<br>High_Male - Medium_Female;<br>Medium_Male - High_Male;<br>Low_Male - Low_Female;<br>Low_Female - Medium_Female;<br>Medium_Male - Low_Female;<br>Low_Male - Medium_Female;<br>Low_Male - Medium_Male;<br>Medium_Male - Medium_Female	A_33_P3217700	NM_004654	NM_004654
2	TXLNG2P	taxilin gamma 2, pseudogene	11.762	1.38E-07	6.8616	0.00019628	High_Male - High_Female;<br>Low_Female - High_Female;<br>Low_Male - High_Female;<br>Medium_Female - High_Female;<br>Medium_Male - High_Female;<br>High_Male - Low_Female;<br>Low_Male - High_Male;<br>High_Male - Medium_Female;<br>Medium_Male - High_Male;<br>Low_Male - Low_Female;<br>Low_Female - Medium_Female;<br>Medium_Male - Low_Female;<br>Low_Male - Medium_Female;<br>Low_Male - Medium_Male;<br>Medium_Male - Medium_Female	A_33_P3260223	NR_045128	NR_045128

3	TXLNG2P	taxilin gamma 2, pseudogene	11.649	1.56E-07	6.8072	0.00019628	High_Male - High_Female; Low_Female - High_Female; Low_Male - High_Female; Medium_Female - High_Female; Medium_Male - High_Female; High_Male - Low_Female; Low_Male - High_Male; High_Male - Medium_Female; Medium_Male - High_Male; Low_Male - Low_Female; Low_Female - Medium_Female; Medium_Male - Low_Female; Low_Male - Medium_Female; Medium_Male - Low_Male; Medium_Male - Medium_Female	A_23_P364792	NR_045129	NR_045129
4	XIST	X (inactive)-specific transcript (non-protein coding)	11.642	1.57E-07	6.8039	0.00019628	High_Female - High_Male; High_Female - Low_Female; High_Female - Low_Male; High_Female - Medium_Female; High_Female - Medium_Male; Low_Female - High_Male; High_Male - Low_Male; Medium_Female - High_Male; High_Male - Medium_Male; Low_Female - Low_Male; Medium_Female - Low_Female; Low_Female - Medium_Male; Medium_Female - Low_Male; Medium_Male - Low_Male; Medium_Female - Medium_Male	A_19_P00323692	NR_001564	NR_001564

5	EIF1AY	eukaryotic translation initiation factor 1A, Y-linked	11.196	2.59E-07	6.5865	0.00025823	High_Male - High_Female; Low_Female - High_Female; Low_Male - High_Female; Medium_Female - High_Female; Medium_Male - High_Female; High_Male - Low_Female; Low_Male - High_Male; High_Male - Medium_Female; Medium_Male - High_Male; Low_Male - Low_Female; Low_Female - Medium_Female; Medium_Male - Low_Female; Low_Male - Medium_Female; Low_Male - Medium_Male; Medium_Male - Medium_Female	A_24_P237511	NM_004681	NM_004681
6	XLOC_008323		10.852	3.84E-07	6.416	0.00025823	High_Male - High_Female; Low_Female - High_Female; Low_Male - High_Female; Medium_Female - High_Female; Medium_Male - High_Female; High_Male - Low_Female; Low_Male - High_Male; High_Male - Medium_Female; Medium_Male - High_Male; Low_Male - Low_Female; Low_Female - Medium_Female; Medium_Male - Low_Female; Low_Male - Medium_Female; Low_Male - Medium_Male; Medium_Male - Medium_Female	linc|TCONS_00017647		

7	XIST	X (inactive)-specific transcript (non-protein coding)	10.814	4.01E-07	6.3973	0.00025823	High_Female - High_Male; High_Female - Low_Female; High_Female - Low_Male; High_Female - Medium_Female; High_Female - Medium_Male; Low_Female - High_Male; High_Male - Low_Male; Medium_Female - High_Male; High_Male - Medium_Male; Low_Female - Low_Male; Medium_Female - Low_Female; Low_Female - Medium_Male; Medium_Female - Low_Male; Medium_Male - Low_Male; Medium_Female - Medium_Male	A_19_P00319151	NR_001564	NR_001564
8	XIST	X (inactive)-specific transcript (non-protein coding)	10.787	4.13E-07	6.3838	0.00025823	High_Female - High_Male; High_Female - Low_Female; High_Female - Low_Male; High_Female - Medium_Female; High_Female - Medium_Male; Low_Female - High_Male; High_Male - Low_Male; Medium_Female - High_Male; High_Male - Medium_Male; Low_Female - Low_Male; Medium_Female - Low_Female; Low_Female - Medium_Male; Medium_Female - Low_Male; Medium_Male - Low_Male; Medium_Female - Medium_Male	A_19_P00331623	NR_001564	NR_001564

9	XIST	X (inactive)-specific transcript (non-protein coding)	10.653	4.83E-07	6.3164	0.00026808	High_Female - High_Male; High_Female - Low_Female; High_Female - Low_Male; High_Female - Medium_Female; High_Female - Medium_Male; Low_Female - High_Male; High_Male - Low_Male; Medium_Female - High_Male; High_Male - Medium_Male; Low_Female - Low_Male; Medium_Female - Low_Female; Low_Female - Medium_Male; Medium_Female - Low_Male; Medium_Male - Low_Male; Medium_Female - Medium_Male	A_21_P0006538	NR_001564	NR_001564
10	KDM5D	lysine (K)-specific demethylase 5D	10.518	5.65E-07	6.2483	0.0002822	High_Male - High_Female; Low_Female - High_Female; Low_Male - High_Female; Medium_Female - High_Female; Medium_Male - High_Female; High_Male - Low_Female; Low_Male - High_Male; High_Male - Medium_Female; Medium_Male - High_Male; Low_Male - Low_Female; Low_Female - Medium_Female; Medium_Male - Low_Female; Low_Male - Medium_Female; Medium_Male - Low_Male; Medium_Male - Medium_Female	A_23_P137238	NM_004653	NM_004653

11	XIST	X (inactive)-specific transcript (non-protein coding)	10.364	6.76E-07	6.1698	0.00030737	High_Female - High_Male; High_Female - Low_Female; High_Female - Low_Male; High_Female - Medium_Female; High_Female - Medium_Male; Low_Female - High_Male; High_Male - Low_Male; Medium_Female - High_Male; High_Male - Medium_Male; Low_Female - Low_Male; Medium_Female - Low_Female; Low_Female - Medium_Male; Medium_Female - Low_Male; Low_Male - Medium_Male; Medium_Female - Medium_Male	A_19_P00329511	NR_001564	NR_001564
12	XIST	X (inactive)-specific transcript (non-protein coding)	10.191	8.29E-07	6.0813	0.00031531	High_Female - High_Male; High_Female - Low_Female; High_Female - Low_Male; High_Female - Medium_Female; High_Female - Medium_Male; Low_Female - High_Male; High_Male - Low_Male; Medium_Female - High_Male; High_Male - Medium_Male; Low_Female - Low_Male; Medium_Female - Low_Female; Low_Female - Medium_Male; Medium_Female - Low_Male; Low_Male - Medium_Male; Medium_Female - Medium_Male	A_19_P00802872	NR_001564	NR_001564

13	RPS4Y1	ribosomal protein S4, Y-linked 1	10.165	8.55E-07	6.0682	0.00031531	High_Male - High_Female; Low_Female - High_Female; Low_Male - High_Female; Medium_Female - High_Female; Medium_Male - High_Female; High_Male - Low_Female; Low_Male - High_Male; High_Male - Medium_Female; Medium_Male - High_Male; Low_Male - Low_Female; Low_Female - Medium_Female; Medium_Male - Low_Female; Low_Male - Medium_Female; Low_Male - Medium_Male; Medium_Male - Medium_Female	A_23_P259314	NM_001008	NM_001008
14	LOC100509121		10.026	1.01E-06	5.9964	0.00031531	High_Male - High_Female; Low_Female - High_Female; Low_Male - High_Female; Medium_Female - High_Female; Medium_Male - High_Female; High_Male - Low_Female; Low_Male - High_Male; High_Male - Medium_Female; Medium_Male - High_Male; Low_Male - Low_Female; Low_Female - Medium_Female; Medium_Male - Low_Female; Low_Male - Medium_Female; Medium_Male - Low_Male; Medium_Male - Medium_Female	A_21_P0013996	XM_003120334	XM_003120334

15	UTY	ubiquitously transcribed tetratricopeptide repeat gene, Y-linked	10.026	1.01E-06	5.9963	0.00031531	High_Male - High_Female; Low_Female - High_Female; Low_Male - High_Female; Medium_Female - High_Female; Medium_Male - High_Female; Low_Female - High_Male; Low_Male - High_Male; Medium_Female - High_Male; Medium_Male - High_Male; Low_Male - Low_Female; Low_Female - Medium_Female; Medium_Male - Low_Female; Low_Male - Medium_Female; Medium_Male - Low_Male; Medium_Male - Medium_Female	A_33_P3228977		BC012581
16	PP12719	uncharacterized LOC100653022	10.009	1.03E-06	5.9879	0.00031531	High_Female - High_Male; High_Female - Low_Female; High_Female - Low_Male; High_Female - Medium_Female; High_Female - Medium_Male; High_Male - Low_Female; High_Male - Low_Male; High_Male - Medium_Female; Medium_Male - High_Male; Low_Female - Low_Male; Medium_Female - Low_Female; Medium_Male - Low_Female; Medium_Female - Low_Male; Medium_Male - Low_Male; Medium_Male - Medium_Female	A_21_P0014028	XM_003403527	XM_003403527

17	RPS4Y2	ribosomal protein S4, Y-linked 2	9.9114	1.16E-06	5.9371	0.00031531	High_Male - High_Female;<br>Low_Female - High_Female;<br>Low_Male - High_Female;<br>Medium_Female - High_Female;<br>Medium_Male - High_Female;<br>High_Male - Low_Female;<br>Low_Male - High_Male;<br>High_Male - Medium_Female;<br>Medium_Male - High_Male;<br>Low_Male - Low_Female;<br>Low_Female - Medium_Female;<br>Medium_Male - Low_Female;<br>Low_Male - Medium_Female;<br>Low_Male - Medium_Male;<br>Medium_Male - Medium_Female	A_23_P324384	NM_001039567	NM_001039567
18	XIST	X (inactive)-specific transcript (non-protein coding)	9.8979	1.17E-06	5.9301	0.00031531	High_Female - High_Male;<br>High_Female - Low_Female;<br>High_Female - Low_Male;<br>High_Female - Medium_Female;<br>High_Female - Medium_Male;<br>Low_Female - High_Male;<br>High_Male - Low_Male;<br>Medium_Female - High_Male;<br>High_Male - Medium_Male;<br>Low_Female - Low_Male;<br>Medium_Female - Low_Female;<br>Low_Female - Medium_Male;<br>Medium_Female - Low_Male;<br>Medium_Male - Low_Male;<br>Medium_Female - Medium_Male	A_33_P3341686	NR_001564	NR_001564

19	XLOC_008015		9.8812	1.20E-06	5.9214	0.00031531	High_Female - High_Male; High_Female - Low_Female; High_Female - Low_Male; High_Female - Medium_Female; High_Female - Medium_Male; Low_Female - High_Male; High_Male - Low_Male; Medium_Female - High_Male; High_Male - Medium_Male; Low_Female - Low_Male; Medium_Female - Low_Female; Low_Female - Medium_Male; Medium_Female - Low_Male; Medium_Male - Low_Male; Medium_Female - Medium_Male	linc|TCONS_00017433		
20	NCRNA00185	non-protein coding RNA 185	9.8036	1.32E-06	5.8809	0.00031889	High_Male - High_Female; Low_Female - High_Female; Low_Male - High_Female; Medium_Female - High_Female; Medium_Male - High_Female; High_Male - Low_Female; Low_Male - High_Male; High_Male - Medium_Female; Medium_Male - High_Male; Low_Male - Low_Female; Medium_Female - Low_Female; Medium_Male - Low_Female; Low_Male - Medium_Female; Medium_Male - Low_Male; Medium_Male - Medium_Female	A_23_P73848	NR_001544	NR_001544

21	UTY	ubiquitously transcribed tetratricopeptide repeat gene, Y-linked	9.7826	1.35E-06	5.87	0.00031889	High_Male - High_Female; Low_Female - High_Female; Low_Male - High_Female; Medium_Female - High_Female; Medium_Male - High_Female; High_Male - Low_Female; Low_Male - High_Male; High_Male - Medium_Female; Medium_Male - High_Male; Low_Male - Low_Female; Medium_Female - Low_Female; Medium_Male - Low_Female; Low_Male - Medium_Female; Medium_Male - Low_Male; Medium_Male - Medium_Female	A_23_P329835	NM_007125	NM_007125
22	DDX3Y	DEAD (Asp-Glu-Ala-Asp) box polypeptide 3, Y-linked	9.7498	1.40E-06	5.8528	0.00031889	High_Male - High_Female; Low_Female - High_Female; Low_Male - High_Female; Medium_Female - High_Female; Medium_Male - High_Female; High_Male - Low_Female; Low_Male - High_Male; High_Male - Medium_Female; Medium_Male - High_Male; Low_Male - Low_Female; Low_Female - Medium_Female; Medium_Male - Low_Female; Low_Male - Medium_Female; Low_Male - Medium_Male; Medium_Male - Medium_Female	A_33_P3224331	NM_001122665	NM_001122665

23	MALAT1		9.4769	1.95E-06	5.7091	0.00042469	High_Male - High_Female;<br>Low_Female - High_Female;<br>Low_Male - High_Female;<br>Medium_Female - High_Female;<br>Medium_Male - High_Female;<br>Low_Female - High_Male;<br>Low_Male - High_Male;<br>Medium_Female - High_Male;<br>Medium_Male - High_Male;<br>Low_Male - Low_Female;<br>Low_Female - Medium_Female;<br>Low_Female - Medium_Male;<br>Low_Male - Medium_Female;<br>Low_Male - Medium_Male;<br>Medium_Male - Medium_Female	A_19_P00805006	NR_002819	NR_002819
24	EIF1AY	eukaryotic translation initiation factor 1A, Y-linked	9.3622	2.25E-06	5.6481	0.00046834	High_Male - High_Female;<br>Low_Female - High_Female;<br>Low_Male - High_Female;<br>Medium_Female - High_Female;<br>Medium_Male - High_Female;<br>High_Male - Low_Female;<br>Low_Male - High_Male;<br>High_Male - Medium_Female;<br>Medium_Male - High_Male;<br>Low_Male - Low_Female;<br>Low_Female - Medium_Female;<br>Medium_Male - Low_Female;<br>Low_Male - Medium_Female;<br>Medium_Male - Low_Male;<br>Medium_Male - Medium_Female	A_23_P148629	NM_004681	NM_004681

25	MALAT1		9.2065	2.72E-06	5.5649	0.00054456	High_Male - High_Female; Low_Female - High_Female; Low_Male - High_Female; Medium_Female - High_Female; Medium_Male - High_Female; Low_Female - High_Male; Low_Male - High_Male; Medium_Female - High_Male; Medium_Male - High_Male; Low_Male - Low_Female; Low_Female - Medium_Female; Low_Female - Medium_Male; Low_Male - Medium_Female; Low_Male - Medium_Male; Medium_Male - Medium_Female	A_19_P00809614	NR_002819	NR_002819
26	XLOC_008276		8.9388	3.80E-06	5.4204	0.00073025	High_Male - High_Female; Low_Female - High_Female; Low_Male - High_Female; Medium_Female - High_Female; Medium_Male - High_Female; Low_Female - High_Male; Low_Male - High_Male; High_Male - Medium_Female; Medium_Male - High_Male; Low_Male - Low_Female; Low_Female - Medium_Female; Medium_Male - Low_Female; Low_Male - Medium_Female; Low_Male - Medium_Male; Medium_Male - Medium_Female	ens|ENST00000425031|ens|ENST00000435867|tc|THC2649420|linc|TCONS_00017607		

27	LOC10050693 0	uncharacterized LOC100506930	8.7521	4.80E-06	5.3187	0.00088887	High_Male - High_Female; Low_Female - High_Female; Low_Male - High_Female; High_Female - Medium_Female; Medium_Male - High_Female; Low_Female - High_Male; Low_Male - High_Male; High_Male - Medium_Female; Medium_Male - High_Male; Low_Male - Low_Female; Low_Female - Medium_Female; Low_Female - Medium_Male; Low_Male - Medium_Female; Low_Male - Medium_Male; Medium_Male - Medium_Female	A_21_P0011696	NR_038279	NR_038279
28	PRKY	protein kinase, Y-linked, pseudogene	8.4677	6.89E-06	5.1619	0.0012296	High_Male - High_Female; Low_Female - High_Female; Low_Male - High_Female; Medium_Female - High_Female; Medium_Male - High_Female; High_Male - Low_Female; Low_Male - High_Male; High_Male - Medium_Female; Medium_Male - High_Male; Low_Male - Low_Female; Medium_Female - Low_Female; Medium_Male - Low_Female; Low_Male - Medium_Female; Medium_Male - Low_Male; Medium_Male - Medium_Female	A_23_P137248	NR_028062	NR_028062

29	TXLNG2P	taxilin gamma 2, pseudogene	8.1242	1.07E-05	4.9699	0.0018473	High_Male - High_Female; Low_Female - High_Female; Low_Male - High_Female; Medium_Female - High_Female; Medium_Male - High_Female; Low_Female - High_Male; Low_Male - High_Male; High_Male - Medium_Female; Medium_Male - High_Male; Low_Male - Low_Female; Low_Female - Medium_Female; Medium_Male - Low_Female; Low_Male - Medium_Female; Medium_Male - Low_Male; Medium_Male - Medium_Female	A_23_P96658	NR_045128	NR_045128
30	XLOC_008276		8.0445	1.19E-05	4.9249	0.0019807	High_Male - High_Female; Low_Female - High_Female; Low_Male - High_Female; Medium_Female - High_Female; Medium_Male - High_Female; Low_Female - High_Male; Low_Male - High_Male; High_Male - Medium_Female; Medium_Male - High_Male; Low_Male - Low_Female; Low_Female - Medium_Female; Medium_Male - Low_Female; Low_Male - Medium_Female; Low_Male - Medium_Male; Medium_Male - Medium_Female	ens|ENST00000444263|ens|ENST00000420327|linc|TCONS_00017606|linc|TCONS_00016999		

31	LOC100506003		8.0045	1.25E-05	4.9023	0.0020194	High_Male - High_Female; Low_Female - High_Female; Low_Male - High_Female; Medium_Female - High_Female; Medium_Male - High_Female; High_Male - Low_Female; Low_Male - High_Male; High_Male - Medium_Female; Medium_Male - High_Male; Low_Male - Low_Female; Low_Female - Medium_Female; Medium_Male - Low_Female; Low_Male - Medium_Female; Low_Male - Medium_Male; Medium_Male - Medium_Female	A_21_P0014436	XR_109785	XR_109785
32	XLOC_008276		7.9503	1.34E-05	4.8716	0.0020998	High_Male - High_Female; Low_Female - High_Female; Low_Male - High_Female; Medium_Female - High_Female; Medium_Male - High_Female; High_Male - Low_Female; Low_Male - High_Male; High_Male - Medium_Female; Medium_Male - High_Male; Low_Male - Low_Female; Low_Female - Medium_Female; Medium_Male - Low_Female; Low_Male - Medium_Female; Medium_Male - Low_Male; Medium_Male - Medium_Female	ens|ENST00000425031|tc|THC2649420|linc|TCONS_00017607		

33	XLOC_008185		7.8133	1.61E-05	4.7935	0.002437	High_Female - High_Male;<br>High_Female - Low_Female;<br>High_Female - Low_Male;<br>High_Female - Medium_Female;<br>High_Female - Medium_Male;<br>Low_Female - High_Male;<br>High_Male - Low_Male;<br>Medium_Female - High_Male;<br>High_Male - Medium_Male;<br>Low_Female - Low_Male;<br>Medium_Female - Low_Female;<br>Low_Female - Medium_Male;<br>Medium_Female - Low_Male;<br>Medium_Male - Low_Male;<br>Medium_Female - Medium_Male	linc|TCONS_00017074		
34	SFN	stratifin	7.6947	1.88E-05	4.7255	0.0027663	High_Male - High_Female;<br>Low_Female - High_Female;<br>Low_Male - High_Female;<br>High_Female - Medium_Female;<br>High_Female - Medium_Male;<br>Low_Female - High_Male;<br>Low_Male - High_Male;<br>High_Male - Medium_Female;<br>High_Male - Medium_Male;<br>Low_Male - Low_Female;<br>Low_Female - Medium_Female;<br>Low_Female - Medium_Male;<br>Low_Male - Medium_Female;<br>Low_Male - Medium_Male;<br>Medium_Female - Medium_Male	A_33_P3389286	NM_006142	NM_006142

35	RGS1	regulator of G-protein signaling 1	7.6575	1.98E-05	4.7041	0.0028229	High_Male - High_Female;<br>Low_Female - High_Female;<br>Low_Male - High_Female;<br>Medium_Female - High_Female;<br>Medium_Male - High_Female;<br>High_Male - Low_Female;<br>Low_Male - High_Male;<br>High_Male - Medium_Female;<br>High_Male - Medium_Male;<br>Low_Male - Low_Female;<br>Low_Female - Medium_Female;<br>Medium_Male - Low_Female;<br>Low_Male - Medium_Female;<br>Low_Male - Medium_Male;<br>Medium_Male - Medium_Female	A_23_P97141	NM_002922	NM_002922
36	TTTY15	testis-specific transcript, Y-linked 15 (non-protein coding)	7.3469	2.99E-05	4.524	0.0041262	High_Male - High_Female;<br>Low_Female - High_Female;<br>Low_Male - High_Female;<br>Medium_Female - High_Female;<br>Medium_Male - High_Female;<br>High_Male - Low_Female;<br>Low_Male - High_Male;<br>High_Male - Medium_Female;<br>Medium_Male - High_Male;<br>Low_Male - Low_Female;<br>Low_Female - Medium_Female;<br>Medium_Male - Low_Female;<br>Low_Male - Medium_Female;<br>Low_Male - Medium_Male;<br>Medium_Male - Medium_Female	A_21_P0006594	NR_001545	NR_001545

37	IL8	interleukin 8	7.3317	3.05E-05	4.5151	0.0041262	High_Male - High_Female; Low_Female - High_Female; Low_Male - High_Female; High_Female - Medium_Female; Medium_Male - High_Female; Low_Female - High_Male; Low_Male - High_Male; High_Male - Medium_Female; Medium_Male - High_Male; Low_Male - Low_Female; Low_Female - Medium_Female; Low_Female - Medium_Male; Low_Male - Medium_Female; Low_Male - Medium_Male; Medium_Male - Medium_Female	A_32_P87013	NM_000584	NM_000584
38	EGR3	early growth response 3	7.2622	3.35E-05	4.4744	0.0044122	High_Male - High_Female; Low_Female - High_Female; Low_Male - High_Female; Medium_Female - High_Female; Medium_Male - High_Female; High_Male - Low_Female; Low_Male - High_Male; High_Male - Medium_Female; High_Male - Medium_Male; Low_Male - Low_Female; Low_Female - Medium_Female; Low_Female - Medium_Male; Low_Male - Medium_Female; Low_Male - Medium_Male; Medium_Male - Medium_Female	A_23_P216225	NM_004430	NM_004430

39	PTGS2	prostaglandin-endoperoxide synthase 2 (prostaglandin G/H synthase and cyclooxygenase)	7.1725	3.79E-05	4.4217	0.0047569	High_Male - High_Female; Low_Female - High_Female; Low_Male - High_Female; Medium_Female - High_Female; Medium_Male - High_Female; Low_Female - High_Male; Low_Male - High_Male; High_Male - Medium_Female; High_Male - Medium_Male; Low_Male - Low_Female; Low_Female - Medium_Female; Low_Female - Medium_Male; Low_Male - Medium_Female; Low_Male - Medium_Male; Medium_Male - Medium_Female	A_24_P250922	NM_000963	NM_000963
40	CXCR4	chemokine (C-X-C motif) receptor 4	7.1687	3.81E-05	4.4195	0.0047569	High_Male - High_Female; Low_Female - High_Female; Low_Male - High_Female; Medium_Female - High_Female; Medium_Male - High_Female; High_Male - Low_Female; Low_Male - High_Male; High_Male - Medium_Female; High_Male - Medium_Male; Low_Male - Low_Female; Low_Female - Medium_Female; Medium_Male - Low_Female; Low_Male - Medium_Female; Low_Male - Medium_Male; Medium_Male - Medium_Female	A_23_P102000	NM_001008540	NM_001008540

41	XLOC_012515		6.9761	4.95E-05	4.3056	0.0060331	High_Female - High_Male; High_Female - Low_Female; High_Female - Low_Male; High_Female - Medium_Female; High_Female - Medium_Male; High_Male - Low_Female; High_Male - Low_Male; High_Male - Medium_Female; High_Male - Medium_Male; Low_Female - Low_Male; Medium_Female - Low_Female; Medium_Male - Low_Female; Medium_Female - Low_Male; Medium_Male - Low_Male; Medium_Female - Medium_Male	ens|ENST00000508851|tc|THC2707471|linc|TCONS_00025694|linc|TCONS_00025237		
42	IL8	interleukin 8	6.9492	5.13E-05	4.2895	0.0061107	High_Male - High_Female; Low_Female - High_Female; Low_Male - High_Female; Medium_Female - High_Female; Medium_Male - High_Female; Low_Female - High_Male; Low_Male - High_Male; Medium_Female - High_Male; Medium_Male - High_Male; Low_Male - Low_Female; Low_Female - Medium_Female; Low_Female - Medium_Male; Low_Male - Medium_Female; Low_Male - Medium_Male; Medium_Female - Medium_Male	ens|ENST00000401931|tc|THC2544321|tc|THC2508924		

43	EGR2	early growth response 2	6.9194	5.35E-05	4.2718	0.0062172	High_Male - High_Female; Low_Female - High_Female; Low_Male - High_Female; Medium_Female - High_Female; Medium_Male - High_Female; Low_Female - High_Male; Low_Male - High_Male; High_Male - Medium_Female; High_Male - Medium_Male; Low_Male - Low_Female; Low_Female - Medium_Female; Low_Female - Medium_Male; Low_Male - Medium_Female; Low_Male - Medium_Male; Medium_Male - Medium_Female	A_23_P46936	NM_000399	NM_000399
44	ZFY	zinc finger protein, Y-linked	6.5715	8.65E-05	4.0628	0.009831	High_Male - High_Female; Low_Female - High_Female; Low_Male - High_Female; Medium_Female - High_Female; Medium_Male - High_Female; High_Male - Low_Female; Low_Male - High_Male; High_Male - Medium_Female; Medium_Male - High_Male; Low_Male - Low_Female; Low_Female - Medium_Female; Medium_Male - Low_Female; Low_Male - Medium_Female; Medium_Male - Low_Male; Medium_Male - Medium_Female	A_24_P942743	NM_003411	NM_003411

45	TTTY15	testis-specific transcript, Y-linked 15 (non-protein coding)	6.5332	9.13E-05	4.0396	0.0099916	High_Male - High_Female; Low_Female - High_Female; Low_Male - High_Female; Medium_Female - High_Female; Medium_Male - High_Female; High_Male - Low_Female; Low_Male - High_Male; High_Male - Medium_Female; Medium_Male - High_Male; Low_Male - Low_Female; Low_Female - Medium_Female; Medium_Male - Low_Female; Low_Male - Medium_Female; Low_Male - Medium_Male; Medium_Male - Medium_Female	A_24_P348861	NR_001545	NR_001545
46	LOC643072	uncharacterized LOC643072	6.5281	9.19E-05	4.0365	0.0099916	High_Female - High_Male; High_Female - Low_Female; High_Female - Low_Male; High_Female - Medium_Female; High_Female - Medium_Male; High_Male - Low_Female; High_Male - Low_Male; High_Male - Medium_Female; High_Male - Medium_Male; Low_Female - Low_Male; Low_Female - Medium_Female; Low_Female - Medium_Male; Medium_Female - Low_Male; Medium_Male - Low_Male; Medium_Female - Medium_Male	A_21_P0014102	XR_108434	XR_108434

47	DDX3Y	DEAD (Asp-Glu-Ala-Asp) box polypeptide 3, Y-linked	6.4925	9.66E-05	4.0148	0.010049	High_Male - High_Female; Low_Female - High_Female; Low_Male - High_Female; Medium_Female - High_Female; Medium_Male - High_Female; High_Male - Low_Female; Low_Male - High_Male; High_Male - Medium_Female; Medium_Male - High_Male; Low_Male - Low_Female; Low_Female - Medium_Female; Medium_Male - Low_Female; Low_Male - Medium_Female; Medium_Male - Low_Male; Medium_Male - Medium_Female	A_24_P130936	NM_004660	NM_004660
48	FOSB	FBJ murine osteosarcoma viral oncogene homolog B	6.4808	9.82E-05	4.0078	0.010049	High_Male - High_Female; Low_Female - High_Female; Low_Male - High_Female; Medium_Female - High_Female; Medium_Male - High_Female; High_Male - Low_Female; Low_Male - High_Male; High_Male - Medium_Female; High_Male - Medium_Male; Low_Male - Low_Female; Low_Female - Medium_Female; Low_Female - Medium_Male; Low_Male - Medium_Female; Low_Male - Medium_Male; Medium_Male - Medium_Female	A_23_P429998	NM_006732	NM_006732

49	AREG	amphiregulin	6.4789	9.85E-05	4.0066	0.010049	High_Male - High_Female;<br>Low_Female - High_Female;<br>Low_Male - High_Female;<br>High_Female - Medium_Female;<br>Medium_Male - High_Female;<br>High_Male - Low_Female;<br>Low_Male - High_Male;<br>High_Male - Medium_Female;<br>High_Male - Medium_Male;<br>Low_Male - Low_Female;<br>Low_Female - Medium_Female;<br>Medium_Male - Low_Female;<br>Low_Male - Medium_Female;<br>Low_Male - Medium_Male;<br>Medium_Male - Medium_Female	A_23_P259071	NM_001657	NM_001657
50	SNAR-B2	small ILF3/NF90-associated RNA B2	6.4542	0.00010197	3.9915	0.010195	High_Female - High_Male;<br>High_Female - Low_Female;<br>High_Female - Low_Male;<br>High_Female - Medium_Female;<br>High_Female - Medium_Male;<br>Low_Female - High_Male;<br>High_Male - Low_Male;<br>Medium_Female - High_Male;<br>Medium_Male - High_Male;<br>Low_Female - Low_Male;<br>Medium_Female - Low_Female;<br>Medium_Male - Low_Female;<br>Medium_Female - Low_Male;<br>Medium_Male - Low_Male;<br>Medium_Female - Medium_Male	A_21_P0000507	NR_024230	NR_024230

51	EGR1	early growth response 1	6.3979	0.00011035	3.9572	0.010817	High_Male - High_Female; Low_Female - High_Female; Low_Male - High_Female; Medium_Female - High_Female; Medium_Male - High_Female; High_Male - Low_Female; Low_Male - High_Male; High_Male - Medium_Female; High_Male - Medium_Male; Low_Male - Low_Female; Medium_Female - Low_Female; Low_Female - Medium_Male; Low_Male - Medium_Female; Low_Male - Medium_Male; Medium_Female - Medium_Male	A_23_P214080	NM_001964	NM_001964
52	UTY	ubiquitously transcribed tetratricopeptide repeat gene, Y-linked	6.2118	0.00014351	3.8431	0.013796	High_Male - High_Female; Low_Female - High_Female; Low_Male - High_Female; Medium_Female - High_Female; Medium_Male - High_Female; Low_Female - High_Male; Low_Male - High_Male; Medium_Female - High_Male; Medium_Male - High_Male; Low_Male - Low_Female; Low_Female - Medium_Female; Medium_Male - Low_Female; Low_Male - Medium_Female; Low_Male - Medium_Male; Medium_Male - Medium_Female	A_23_P160004	NM_182660	NM_182660

53	ADORA3	adenosine A3 receptor	6.1923	0.00014755	3.8311	0.013917	High_Male - High_Female; High_Female - Low_Female; Low_Male - High_Female; Medium_Female - High_Female; Medium_Male - High_Female; High_Male - Low_Female; High_Male - Low_Male; High_Male - Medium_Female; High_Male - Medium_Male; Low_Male - Low_Female; Medium_Female - Low_Female; Medium_Male - Low_Female; Medium_Female - Low_Male; Medium_Male - Low_Male; Medium_Male - Medium_Female	A_23_P137931	NM_000677	NM_000677
54	CCL3L3	chemokine (C-C motif) ligand 3-like 3	6.157	0.00015513	3.8093	0.014327	High_Male - High_Female; Low_Female - High_Female; Low_Male - High_Female; Medium_Female - High_Female; Medium_Male - High_Female; Low_Female - High_Male; Low_Male - High_Male; High_Male - Medium_Female; High_Male - Medium_Male; Low_Female - Low_Male; Low_Female - Medium_Female; Low_Female - Medium_Male; Low_Male - Medium_Female; Low_Male - Medium_Male; Medium_Female - Medium_Male	A_33_P3296181	NM_001001437	NM_001001437

55	XLOC_l2_015892		6.1458	0.00015762	3.8024	0.014327	High_Male - High_Female; Low_Female - High_Female; Low_Male - High_Female; Medium_Female - High_Female; Medium_Male - High_Female; High_Male - Low_Female; Low_Male - High_Male; High_Male - Medium_Female; Medium_Male - High_Male; Low_Male - Low_Female; Low_Female - Medium_Female; Medium_Male - Low_Female; Low_Male - Medium_Female; Low_Male - Medium_Male; Medium_Male - Medium_Female	ens|ENST00000430079|ens|ENST00000460561|tc|THC2783530|linc|TCONS_l2_00030900		
56	G0S2	G0/G1switch 2	5.7336	0.0002849	3.5453	0.025432	High_Male - High_Female; Low_Female - High_Female; Low_Male - High_Female; Medium_Female - High_Female; Medium_Male - High_Female; Low_Female - High_Male; Low_Male - High_Male; High_Male - Medium_Female; Medium_Male - High_Male; Low_Male - Low_Female; Low_Female - Medium_Female; Low_Female - Medium_Male; Low_Male - Medium_Female; Low_Male - Medium_Male; Medium_Male - Medium_Female	A_23_P74609	NM_015714	NM_015714

57	PMAIP1	phorbol-12-myristate-13-acetate-induced protein 1	5.5138	0.00039242	3.4063	0.034416	High_Male - High_Female; Low_Female - High_Female; Low_Male - High_Female; Medium_Female - High_Female; Medium_Male - High_Female; High_Male - Low_Female; High_Male - Low_Male; High_Male - Medium_Female; High_Male - Medium_Male; Low_Male - Low_Female; Low_Female - Medium_Female; Low_Female - Medium_Male; Low_Male - Medium_Female; Low_Male - Medium_Male; Medium_Male - Medium_Female	A_33_P3619171	NM_021127	NM_021127
58	FGFR2	fibroblast growth factor receptor 2	5.487	0.00040819	3.3891	0.035182	High_Male - High_Female; Low_Female - High_Female; Low_Male - High_Female; Medium_Female - High_Female; Medium_Male - High_Female; High_Male - Low_Female; High_Male - Low_Male; High_Male - Medium_Female; High_Male - Medium_Male; Low_Male - Low_Female; Medium_Female - Low_Female; Medium_Male - Low_Female; Low_Male - Medium_Female; Medium_Male - Low_Male; Medium_Male - Medium_Female	A_23_P202334	NM_022970	NM_022970

59	FOS	FBJ murine osteosarcoma viral oncogene homolog	5.4662	0.00042083	3.3759	0.035656	High_Male - High_Female; Low_Female - High_Female; Low_Male - High_Female; Medium_Female - High_Female; Medium_Male - High_Female; Low_Female - High_Male; Low_Male - High_Male; Medium_Female - High_Male; Medium_Male - High_Male; Low_Male - Low_Female; Low_Female - Medium_Female; Low_Female - Medium_Male; Low_Male - Medium_Female; Low_Male - Medium_Male; Medium_Female - Medium_Male	A_23_P106194	NM_005252	NM_005252
60	PHACTR1	phosphatase and actin regulator 1	5.4522	0.00042955	3.367	0.035789	High_Male - High_Female; Low_Female - High_Female; Low_Male - High_Female; Medium_Female - High_Female; Medium_Male - High_Female; Low_Female - High_Male; Low_Male - High_Male; High_Male - Medium_Female; Medium_Male - High_Male; Low_Male - Low_Female; Low_Female - Medium_Female; Low_Female - Medium_Male; Low_Male - Medium_Female; Low_Male - Medium_Male; Medium_Male - Medium_Female	A_24_P943566		AB051520

61	DUSP2	dual specificity phosphatase 2	5.328	0.00051573	3.2876	0.041844	High_Male - High_Female; Low_Female - High_Female; Low_Male - High_Female; Medium_Female - High_Female; Medium_Male - High_Female; High_Male - Low_Female; Low_Male - High_Male; High_Male - Medium_Female; High_Male - Medium_Male; Low_Male - Low_Female; Low_Female - Medium_Female; Medium_Male - Low_Female; Low_Male - Medium_Female; Low_Male - Medium_Male; Medium_Male - Medium_Female	A_24_P37409	NM_004418	NM_004418
62	P2RY2	purinergic receptor P2Y, G-protein coupled, 2	5.3174	0.00052391	3.2807	0.041844	High_Male - High_Female; Low_Female - High_Female; Low_Male - High_Female; Medium_Female - High_Female; Medium_Male - High_Female; High_Male - Low_Female; High_Male - Low_Male; High_Male - Medium_Female; Medium_Male - High_Male; Low_Male - Low_Female; Medium_Female - Low_Female; Medium_Male - Low_Female; Low_Male - Medium_Female; Medium_Male - Low_Male; Medium_Male - Medium_Female	A_23_P24903	NM_176072	NM_176072

63	STK17B	serine/threonine kinase 17b	5.313	0.00052733	3.2779	0.041844	High_Female - High_Male; Low_Female - High_Female; Low_Male - High_Female; High_Female - Medium_Female; Medium_Male - High_Female; Low_Female - High_Male; Low_Male - High_Male; Medium_Female - High_Male; Medium_Male - High_Male; Low_Male - Low_Female; Low_Female - Medium_Female; Medium_Male - Low_Female; Low_Male - Medium_Female; Low_Male - Medium_Male; Medium_Male - Medium_Female	A_33_P3371650		BC052561
64	PRSS33	protease, serine, 33	5.2609	0.00056954	3.2445	0.044487	High_Male - High_Female; Low_Female - High_Female; Low_Male - High_Female; Medium_Female - High_Female; Medium_Male - High_Female; High_Male - Low_Female; High_Male - Low_Male; High_Male - Medium_Female; High_Male - Medium_Male; Low_Male - Low_Female; Medium_Female - Low_Female; Medium_Male - Low_Female; Medium_Female - Low_Male; Medium_Male - Low_Male; Medium_Male - Medium_Female	A_24_P327084	NM_152891	NM_152891

65	XLOC_000683		5.2309	0.0005955	3.2251	0.045317	High_Female - High_Male; High_Female - Low_Female; High_Female - Low_Male; High_Female - Medium_Female; High_Female - Medium_Male; High_Male - Low_Female; High_Male - Low_Male; High_Male - Medium_Female; Medium_Male - High_Male; Low_Female - Low_Male; Medium_Female - Low_Female; Medium_Male - Low_Female; Medium_Female - Low_Male; Medium_Male - Low_Male; Medium_Male - Medium_Female	tc|THC2749835|linc|TCONS_00002281		
66			5.2277	0.00059831	3.2231	0.045317	High_Male - High_Female; Low_Female - High_Female; Low_Male - High_Female; Medium_Female - High_Female; High_Female - Medium_Male; Low_Female - High_Male; High_Male - Low_Male; Medium_Female - High_Male; High_Male - Medium_Male; Low_Female - Low_Male; Low_Female - Medium_Female; Low_Female - Medium_Male; Medium_Female - Low_Male; Low_Male - Medium_Male; Medium_Female - Medium_Male	A_33_P3415962		AK130540

67	PER1	period homolog 1 (Drosophila)	5.1996	0.00062376	3.205	0.04654	High_Male - High_Female; Low_Female - High_Female; Low_Male - High_Female; High_Female - Medium_Female; Medium_Male - High_Female; High_Male - Low_Female; Low_Male - High_Male; High_Male - Medium_Female; High_Male - Medium_Male; Low_Male - Low_Female; Low_Female - Medium_Female; Medium_Male - Low_Female; Low_Male - Medium_Female; Low_Male - Medium_Male; Medium_Male - Medium_Female	A_23_P89589	NM_002616	NM_002616
68	SNAR-D	small ILF3/NF90-associated RNA D	5.1542	0.00066734	3.1757	0.049059	High_Female - High_Male; High_Female - Low_Female; High_Female - Low_Male; High_Female - Medium_Female; High_Female - Medium_Male; Low_Female - High_Male; High_Male - Low_Male; Medium_Female - High_Male; Medium_Male - High_Male; Low_Female - Low_Male; Medium_Female - Low_Female; Medium_Male - Low_Female; Medium_Female - Low_Male; Medium_Male - Low_Male; Medium_Female - Medium_Male	A_21_P0000508	NR_024243	NR_024243

69	LOC10050725 4	uncharacterized LOC100507254	5.1184	0.0007039	3.1525	0.050997	High_Male - High_Female; Low_Female - High_Female; Low_Male - High_Female; Medium_Female - High_Female; Medium_Male - High_Female; High_Male - Low_Female; Low_Male - High_Male; Medium_Female - High_Male; Medium_Male - High_Male; Low_Male - Low_Female; Medium_Female - Low_Female; Medium_Male - Low_Female; Medium_Female - Low_Male; Medium_Male - Low_Male; Medium_Male - Medium_Female	A_21_P0004942	NR_038981	NR_038981
70	XLOC_012515		5.0702	0.00075638	3.1213	0.054016	High_Female - High_Male; High_Female - Low_Female; High_Female - Low_Male; High_Female - Medium_Female; High_Female - Medium_Male; High_Male - Low_Female; High_Male - Low_Male; High_Male - Medium_Female; High_Male - Medium_Male; Low_Female - Low_Male; Medium_Female - Low_Female; Medium_Male - Low_Female; Medium_Female - Low_Male; Medium_Male - Low_Male; Medium_Male - Medium_Female	ens|ENST00000508851|tc|THC2707471|linc|TCONS_00025237		

71	SLC29A1	solute carrier family 29 (nucleoside transporters), member 1	5.0422	0.00078869	3.1031	0.055531	High_Male - High_Female; Low_Female - High_Female; Low_Male - High_Female; Medium_Female - High_Female; Medium_Male - High_Female; High_Male - Low_Female; High_Male - Low_Male; High_Male - Medium_Female; Medium_Male - High_Male; Low_Male - Low_Female; Medium_Female - Low_Female; Medium_Male - Low_Female; Low_Male - Medium_Female; Medium_Male - Low_Male; Medium_Male - Medium_Female	A_23_P133694	NM_001078177	NM_001078177
72	TNFRSF12A	tumor necrosis factor receptor superfamily, member 12A	5.0115	0.00082585	3.0831	0.057339	High_Male - High_Female; High_Female - Low_Female; High_Female - Low_Male; High_Female - Medium_Female; Medium_Male - High_Female; High_Male - Low_Female; High_Male - Low_Male; High_Male - Medium_Female; High_Male - Medium_Male; Low_Male - Low_Female; Medium_Female - Low_Female; Medium_Male - Low_Female; Medium_Female - Low_Male; Medium_Male - Low_Male; Medium_Male - Medium_Female	A_23_P49338	NM_016639	NM_016639

73	XLOC_003772		4.9875	0.00085606	3.0675	0.058386	High_Male - High_Female; Low_Female - High_Female; Low_Male - High_Female; Medium_Female - High_Female; Medium_Male - High_Female; Low_Female - High_Male; High_Male - Low_Male; Medium_Female - High_Male; High_Male - Medium_Male; Low_Female - Low_Male; Low_Female - Medium_Female; Low_Female - Medium_Male; Medium_Female - Low_Male; Low_Male - Medium_Male; Medium_Female - Medium_Male	linc|TCONS_00008317|linc|TCONS_00008929		
74	LOC100506342	uncharacterized LOC100506342	4.9811	0.00086429	3.0633	0.058386	High_Male - High_Female; Low_Female - High_Female; Low_Male - High_Female; Medium_Female - High_Female; Medium_Male - High_Female; Low_Female - High_Male; Low_Male - High_Male; High_Male - Medium_Female; High_Male - Medium_Male; Low_Female - Low_Male; Low_Female - Medium_Female; Low_Female - Medium_Male; Low_Male - Medium_Female; Low_Male - Medium_Male; Medium_Male - Medium_Female	A_33_P3514859	XR_108862	XR_108862

75	LOC100652951	uncharacterized LOC100652951	4.9439	0.00091386	3.0391	0.060549	High_Male - High_Female; Low_Female - High_Female; Low_Male - High_Female; High_Female - Medium_Female; Medium_Male - High_Female; Low_Female - High_Male; Low_Male - High_Male; High_Male - Medium_Female; Medium_Male - High_Male; Low_Male - Low_Female; Low_Female - Medium_Female; Low_Female - Medium_Male; Low_Male - Medium_Female; Low_Male - Medium_Male; Medium_Male - Medium_Female	A_21_P0014898	XR_132888	XR_132888
76	ABP1	amiloride binding protein 1 (amine oxidase (copper-containing))	4.9391	0.00092053	3.036	0.060549	High_Male - High_Female; High_Female - Low_Female; High_Female - Low_Male; Medium_Female - High_Female; Medium_Male - High_Female; High_Male - Low_Female; High_Male - Low_Male; High_Male - Medium_Female; Medium_Male - High_Male; Low_Female - Low_Male; Medium_Female - Low_Female; Medium_Male - Low_Female; Medium_Female - Low_Male; Medium_Male - Low_Male; Medium_Male - Medium_Female	A_23_P59452	NM_001091	NM_001091

77	XLOC_011047		4.9145	0.00095515	3.0199	0.062011	High_Male - High_Female; Low_Female - High_Female; Low_Male - High_Female; High_Female - Medium_Female; Medium_Male - High_Female; High_Male - Low_Female; High_Male - Low_Male; High_Male - Medium_Female; Medium_Male - High_Male; Low_Male - Low_Female; Low_Female - Medium_Female; Medium_Male - Low_Female; Low_Male - Medium_Female; Medium_Male - Low_Male; Medium_Male - Medium_Female	tc|THC2533854|linc|TCONS_00023118|linc|TCONS_00023117|linc|TCONS_00022763		
78	BTG2	BTG family, member 2	4.8941	0.00098504	3.0065	0.06252	High_Male - High_Female; Low_Female - High_Female; Low_Male - High_Female; Medium_Female - High_Female; Medium_Male - High_Female; Low_Female - High_Male; Low_Male - High_Male; High_Male - Medium_Female; High_Male - Medium_Male; Low_Male - Low_Female; Low_Female - Medium_Female; Low_Female - Medium_Male; Low_Male - Medium_Female; Low_Male - Medium_Male; Medium_Male - Medium_Female	A_23_P62901	NM_006763	NM_006763

79	LOC10050725<br>4	uncharacterized LOC100507254	4.8921	0.00098801	3.0052	0.06252	High_Male - High_Female;<br>Low_Female - High_Female;<br>Low_Male - High_Female;<br>Medium_Female - High_Female;<br>Medium_Male - High_Female;<br>Low_Female - High_Male;<br>Low_Male - High_Male;<br>Medium_Female - High_Male;<br>Medium_Male - High_Male;<br>Low_Male - Low_Female;<br>Medium_Female - Low_Female;<br>Medium_Male - Low_Female;<br>Medium_Female - Low_Male;<br>Medium_Male - Low_Male;<br>Medium_Male - Medium_Female	A_19_P00322058	NR_038981	NR_038981
80	ATOH8	atonal homolog 8 (Drosophila)	4.8725	0.0010176	2.9924	0.063587	High_Male - High_Female;<br>Low_Female - High_Female;<br>Low_Male - High_Female;<br>Medium_Female - High_Female;<br>Medium_Male - High_Female;<br>Low_Female - High_Male;<br>High_Male - Low_Male;<br>High_Male - Medium_Female;<br>High_Male - Medium_Male;<br>Low_Female - Low_Male;<br>Low_Female - Medium_Female;<br>Low_Female - Medium_Male;<br>Low_Male - Medium_Female;<br>Low_Male - Medium_Male;<br>Medium_Female - Medium_Male	A_33_P3392740		AL831857

81	SEC14L3	SEC14-like 3 (S. cerevisiae)	4.8144	0.0011107	2.9544	0.067958	High_Male - High_Female;<br>Low_Female - High_Female;<br>Low_Male - High_Female;<br>Medium_Female - High_Female;<br>High_Female - Medium_Male;<br>Low_Female - High_Male;<br>Low_Male - High_Male;<br>Medium_Female - High_Male;<br>High_Male - Medium_Male;<br>Low_Male - Low_Female;<br>Low_Female - Medium_Female;<br>Low_Female - Medium_Male;<br>Low_Male - Medium_Female;<br>Low_Male - Medium_Male;<br>Medium_Female - Medium_Male	A_33_P3383551		AK131358
82	LOC391322	D-dopachrome tautomerase-like	4.812	0.0011147	2.9528	0.067958	High_Female - High_Male;<br>Low_Female - High_Female;<br>Low_Male - High_Female;<br>High_Female - Medium_Female;<br>High_Female - Medium_Male;<br>Low_Female - High_Male;<br>Low_Male - High_Male;<br>High_Male - Medium_Female;<br>Medium_Male - High_Male;<br>Low_Male - Low_Female;<br>Low_Female - Medium_Female;<br>Low_Female - Medium_Male;<br>Low_Male - Medium_Female;<br>Low_Male - Medium_Male;<br>Medium_Male - Medium_Female	A_33_P3379341	NM_001144931	NM_001144931

83	DDIT4	DNA-damage-inducible transcript 4	4.8038	0.0011287	2.9474	0.06798	High_Male - High_Female; High_Female - Low_Female; Low_Male - High_Female; High_Female - Medium_Female; Medium_Male - High_Female; High_Male - Low_Female; Low_Male - High_Male; High_Male - Medium_Female; High_Male - Medium_Male; Low_Male - Low_Female; Low_Female - Medium_Female; Medium_Male - Low_Female; Low_Male - Medium_Female; Low_Male - Medium_Male; Medium_Male - Medium_Female	A_23_P104318	NM_019058	NM_019058
84	XLOC_012043		4.7882	0.0011555	2.9372	0.068769	High_Male - High_Female; Low_Female - High_Female; Low_Male - High_Female; Medium_Female - High_Female; High_Female - Medium_Male; Low_Female - High_Male; High_Male - Low_Male; Medium_Female - High_Male; High_Male - Medium_Male; Low_Female - Low_Male; Low_Female - Medium_Female; Low_Female - Medium_Male; Medium_Female - Low_Male; Low_Male - Medium_Male; Medium_Female - Medium_Male	linc|TCONS_00025079|linc|TCONS_00025080|linc|TCONS_00024759		

85	LOC100507540	uncharacterized LOC100507540	4.7452	0.0012333	2.9089	0.072534	High_Male - High_Female; Low_Female - High_Female; High_Female - Low_Male; Medium_Female - High_Female; Medium_Male - High_Female; High_Male - Low_Female; High_Male - Low_Male; High_Male - Medium_Female; High_Male - Medium_Male; Low_Female - Low_Male; Medium_Female - Low_Female; Medium_Male - Low_Female; Medium_Female - Low_Male; Medium_Male - Low_Male; Medium_Male - Medium_Female	A_21_P0013589	XR_108989	XR_108989
86	CYP1B1-AS1		4.6971	0.0013267	2.8772	0.077117	High_Female - High_Male; High_Female - Low_Female; High_Female - Low_Male; High_Female - Medium_Female; High_Female - Medium_Male; High_Male - Low_Female; High_Male - Low_Male; High_Male - Medium_Female; Medium_Male - High_Male; Low_Female - Low_Male; Medium_Female - Low_Female; Medium_Male - Low_Female; Medium_Female - Low_Male; Medium_Male - Low_Male; Medium_Male - Medium_Female	A_19_P00807643	NR_027252	NR_027252

87	XLOC_008151		4.6765	0.0013689	2.8636	0.078658	High_Male - High_Female; Low_Female - High_Female; Low_Male - High_Female; Medium_Female - High_Female; Medium_Male - High_Female; Low_Female - High_Male; Low_Male - High_Male; High_Male - Medium_Female; High_Male - Medium_Male; Low_Female - Low_Male; Low_Female - Medium_Female; Low_Female - Medium_Male; Low_Male - Medium_Female; Low_Male - Medium_Male; Medium_Female - Medium_Male	A_21_P0006579		BC015977
88	TBC1D7	TBC1 domain family, member 7	4.6346	0.0014589	2.836	0.082878	High_Male - High_Female; Low_Female - High_Female; Low_Male - High_Female; Medium_Female - High_Female; Medium_Male - High_Female; Low_Female - High_Male; Low_Male - High_Male; High_Male - Medium_Female; High_Male - Medium_Male; Low_Female - Low_Male; Low_Female - Medium_Female; Low_Female - Medium_Male; Low_Male - Medium_Female; Low_Male - Medium_Male; Medium_Female - Medium_Male	A_33_P3311907	NM_001143965	NM_001143965

89	VAT1	vesicle amine transport protein 1 homolog (T. californica)	4.6119	0.0015102	2.821	0.084826	High_Male - High_Female; Low_Female - High_Female; Low_Male - High_Female; Medium_Female - High_Female; Medium_Male - High_Female; Low_Female - High_Male; High_Male - Low_Male; Medium_Female - High_Male; High_Male - Medium_Male; Low_Female - Low_Male; Low_Female - Medium_Female; Low_Female - Medium_Male; Medium_Female - Low_Male; Low_Male - Medium_Male; Medium_Female - Medium_Male	A_24_P152527	NM_006373	NM_006373
90	SMPD3	sphingomyelin phosphodiesterase 3, neutral membrane (neutral sphingomyelinase II)	4.6038	0.001529	2.8156	0.084925	High_Male - High_Female; Low_Female - High_Female; Low_Male - High_Female; Medium_Female - High_Female; Medium_Male - High_Female; High_Male - Low_Female; High_Male - Low_Male; High_Male - Medium_Female; Medium_Male - High_Male; Low_Male - Low_Female; Medium_Female - Low_Female; Medium_Male - Low_Female; Medium_Female - Low_Male; Medium_Male - Low_Male; Medium_Male - Medium_Female	A_23_P163567	NM_018667	NM_018667

91	CYP4F12	cytochrome P450, family 4, subfamily F, polypeptide 12	4.5762	0.0015948	2.7973	0.087493	High_Male - High_Female; High_Female - Low_Female; High_Female - Low_Male; High_Female - Medium_Female; Medium_Male - High_Female; High_Male - Low_Female; High_Male - Low_Male; High_Male - Medium_Female; High_Male - Medium_Male; Low_Male - Low_Female; Medium_Female - Low_Female; Medium_Male - Low_Female; Medium_Female - Low_Male; Medium_Male - Low_Male; Medium_Male - Medium_Female	A_23_P108280	NM_023944	NM_023944
92	CLC	Charcot-Leyden crystal protein	4.5698	0.0016102	2.7931	0.087493	High_Male - High_Female; Low_Female - High_Female; Low_Male - High_Female; Medium_Female - High_Female; Medium_Male - High_Female; High_Male - Low_Female; High_Male - Low_Male; High_Male - Medium_Female; High_Male - Medium_Male; Low_Male - Low_Female; Medium_Female - Low_Female; Medium_Male - Low_Female; Medium_Female - Low_Male; Medium_Male - Low_Male; Medium_Male - Medium_Female	A_23_P101683	NM_001828	NM_001828

93	RHOXF1	Rhox homeobox family, member 1	4.5192	0.0017398	2.7595	0.093521	High_Female - High_Male; Low_Female - High_Female; Low_Male - High_Female; High_Female - Medium_Female; High_Female - Medium_Male; Low_Female - High_Male; Low_Male - High_Male; Medium_Female - High_Male; Medium_Male - High_Male; Low_Female - Low_Male; Low_Female - Medium_Female; Low_Female - Medium_Male; Low_Male - Medium_Female; Low_Male - Medium_Male; Medium_Female - Medium_Male	A_23_P85082	NM_139282	NM_139282
94	XLOC_l2_015178		4.4652	0.0018898	2.7236	0.1005	High_Male - High_Female; Low_Female - High_Female; Low_Male - High_Female; Medium_Female - High_Female; Medium_Male - High_Female; Low_Female - High_Male; Low_Male - High_Male; Medium_Female - High_Male; Medium_Male - High_Male; Low_Male - Low_Female; Medium_Female - Low_Female; Medium_Male - Low_Female; Medium_Female - Low_Male; Medium_Male - Low_Male; Medium_Male - Medium_Female	A_21_P0013637		CB121594

95	EMR1	egf-like module containing, mucin-like, hormone receptor-like 1	4.447	0.0019432	2.7115	0.10225	High_Male - High_Female; Low_Female - High_Female; Low_Male - High_Female; Medium_Female - High_Female; Medium_Male - High_Female; High_Male - Low_Female; High_Male - Low_Male; High_Male - Medium_Female; High_Male - Medium_Male; Low_Male - Low_Female; Medium_Female - Low_Female; Medium_Male - Low_Female; Medium_Female - Low_Male; Medium_Male - Low_Male; Medium_Male - Medium_Female	A_33_P3215803	NM_001974	NM_001974
96	SIGLEC8	sialic acid binding Ig-like lectin 8	4.428	0.0020006	2.6988	0.10315	High_Male - High_Female; Low_Female - High_Female; Low_Male - High_Female; Medium_Female - High_Female; Medium_Male - High_Female; High_Male - Low_Female; High_Male - Low_Male; High_Male - Medium_Female; Medium_Male - High_Male; Low_Male - Low_Female; Medium_Female - Low_Female; Medium_Male - Low_Female; Medium_Female - Low_Male; Medium_Male - Low_Male; Medium_Male - Medium_Female	A_24_P40001	NM_014442	NM_014442

97	GALNT14	UDP-N-acetyl-alpha-D-galactosamine:polypeptide N-acetylgalactosaminyltransferase 14 (GalNAc-T14)	4.4164	0.0020365	2.6911	0.10315	High_Female - High_Male; High_Female - Low_Female; High_Female - Low_Male; Medium_Female - High_Female; High_Female - Medium_Male; High_Male - Low_Female; High_Male - Low_Male; Medium_Female - High_Male; Medium_Male - High_Male; Low_Female - Low_Male; Medium_Female - Low_Female; Medium_Male - Low_Female; Medium_Female - Low_Male; Medium_Male - Low_Male; Medium_Female - Medium_Male	A_23_P67847	NM_024572	NM_024572
98	OR2M7	olfactory receptor, family 2, subfamily M, member 7	4.4146	0.0020423	2.6899	0.10315	High_Male - High_Female; Low_Female - High_Female; Low_Male - High_Female; Medium_Female - High_Female; Medium_Male - High_Female; Low_Female - High_Male; High_Male - Low_Male; Medium_Female - High_Male; High_Male - Medium_Male; Low_Female - Low_Male; Low_Female - Medium_Female; Low_Female - Medium_Male; Medium_Female - Low_Male; Medium_Male - Low_Male; Medium_Female - Medium_Male	A_33_P3311046	NM_001004691	NM_001004691

99	SHISA4	shisa homolog 4 (Xenopus laevis)	4.4144	0.0020428	2.6898	0.10315	High_Male - High_Female; Low_Female - High_Female; Low_Male - High_Female; Medium_Female - High_Female; Medium_Male - High_Female; Low_Female - High_Male; Low_Male - High_Male; High_Male - Medium_Female; High_Male - Medium_Male; Low_Male - Low_Female; Low_Female - Medium_Female; Low_Female - Medium_Male; Low_Male - Medium_Female; Low_Male - Medium_Male; Medium_Male - Medium_Female	A_23_P115573	NM_198149	NM_198149
100	CST7	cystatin F (leukocystatin)	4.4055	0.002071	2.6838	0.10353	High_Female - High_Male; High_Female - Low_Female; High_Female - Low_Male; High_Female - Medium_Female; High_Female - Medium_Male; Low_Female - High_Male; High_Male - Low_Male; Medium_Female - High_Male; Medium_Male - High_Male; Low_Female - Low_Male; Medium_Female - Low_Female; Medium_Male - Low_Female; Medium_Female - Low_Male; Medium_Male - Low_Male; Medium_Male - Medium_Female	A_23_P68601	NM_003650	NM_003650

101	CCL23	chemokine (C-C motif) ligand 23	4.3906	0.002119	2.6739	0.10488	High_Male - High_Female; Low_Female - High_Female; Low_Male - High_Female; Medium_Female - High_Female; Medium_Male - High_Female; High_Male - Low_Female; High_Male - Low_Male; High_Male - Medium_Female; Medium_Male - High_Male; Low_Male - Low_Female; Medium_Female - Low_Female; Medium_Male - Low_Female; Low_Male - Medium_Female; Medium_Male - Low_Male; Medium_Male - Medium_Female	A_24_P133905	NM_005064	NM_005064
102			4.3741	0.0021735	2.6628	0.10652	High_Male - High_Female; Low_Female - High_Female; Low_Male - High_Female; Medium_Female - High_Female; Medium_Male - High_Female; High_Male - Low_Female; High_Male - Low_Male; High_Male - Medium_Female; Medium_Male - High_Male; Low_Male - Low_Female; Medium_Female - Low_Female; Medium_Male - Low_Female; Low_Male - Medium_Female; Medium_Male - Low_Male; Medium_Male - Medium_Female	tc|THC2567789		

103	C9orf40	chromosome 9 open reading frame 40	4.3663	0.0021997	2.6576	0.10676	High_Male - High_Female;<br>High_Female - Low_Female;<br>High_Female - Low_Male;<br>Medium_Female - High_Female;<br>Medium_Male - High_Female;<br>High_Male - Low_Female;<br>High_Male - Low_Male;<br>High_Male - Medium_Female;<br>High_Male - Medium_Male;<br>Low_Female - Low_Male;<br>Medium_Female - Low_Female;<br>Medium_Male - Low_Female;<br>Medium_Female - Low_Male;<br>Medium_Male - Low_Male;<br>Medium_Male - Medium_Female	A_23_P43425	NM_017998	NM_017998
104	SPNS3	spinster homolog 3 (Drosophila)	4.3086	0.0024042	2.619	0.11556	High_Male - High_Female;<br>Low_Female - High_Female;<br>Low_Male - High_Female;<br>Medium_Female - High_Female;<br>Medium_Male - High_Female;<br>High_Male - Low_Female;<br>High_Male - Low_Male;<br>High_Male - Medium_Female;<br>High_Male - Medium_Male;<br>Low_Male - Low_Female;<br>Medium_Female - Low_Female;<br>Medium_Male - Low_Female;<br>Low_Male - Medium_Female;<br>Medium_Male - Low_Male;<br>Medium_Male - Medium_Female	A_23_P100963	NM_182538	NM_182538

105	AREG	amphiregulin	4.2829	0.0025014	2.6018	0.11909	High_Male - High_Female; Low_Female - High_Female; Low_Male - High_Female; Medium_Female - High_Female; Medium_Male - High_Female; High_Male - Low_Female; Low_Male - High_Male; High_Male - Medium_Female; High_Male - Medium_Male; Low_Male - Low_Female; Low_Female - Medium_Female; Medium_Male - Low_Female; Low_Male - Medium_Female; Low_Male - Medium_Male; Medium_Male - Medium_Female	A_33_P3419190	NM_001657	NM_001657
106	CACNG6	calcium channel, voltage-dependent, gamma subunit 6	4.2677	0.0025609	2.5916	0.12077	High_Male - High_Female; Low_Female - High_Female; Low_Male - High_Female; Medium_Female - High_Female; Medium_Male - High_Female; High_Male - Low_Female; High_Male - Low_Male; High_Male - Medium_Female; High_Male - Medium_Male; Low_Male - Low_Female; Medium_Female - Low_Female; Medium_Male - Low_Female; Low_Male - Medium_Female; Medium_Male - Low_Male; Medium_Male - Medium_Female	A_23_P501933	NM_145814	NM_145814

107	TFF3	trefoil factor 3 (intestinal)	4.2173	0.0027685	2.5578	0.12909	High_Male - High_Female; High_Female - Low_Female; Low_Male - High_Female; Medium_Female - High_Female; Medium_Male - High_Female; High_Male - Low_Female; High_Male - Low_Male; High_Male - Medium_Female; High_Male - Medium_Male; Low_Male - Low_Female; Medium_Female - Low_Female; Medium_Male - Low_Female; Medium_Female - Low_Male; Medium_Male - Low_Male; Medium_Male - Medium_Female	A_33_P3334305	NM_003226	NM_003226
108	CA14	carbonic anhydrase XIV	4.2125	0.002789	2.5546	0.12909	High_Male - High_Female; Low_Female - High_Female; Low_Male - High_Female; Medium_Female - High_Female; High_Female - Medium_Male; Low_Female - High_Male; Low_Male - High_Male; High_Male - Medium_Female; High_Male - Medium_Male; Low_Female - Low_Male; Low_Female - Medium_Female; Low_Female - Medium_Male; Low_Male - Medium_Female; Low_Male - Medium_Male; Medium_Female - Medium_Male	A_23_P63379	NM_012113	NM_012113

109	PIK3R6	phosphoinositide-3-kinase, regulatory subunit 6	4.1926	0.0028763	2.5412	0.13192	High_Male - High_Female; High_Female - Low_Female; High_Female - Low_Male; Medium_Female - High_Female; Medium_Male - High_Female; High_Male - Low_Female; High_Male - Low_Male; Medium_Female - High_Male; Medium_Male - High_Male; Low_Male - Low_Female; Medium_Female - Low_Female; Medium_Male - Low_Female; Medium_Female - Low_Male; Medium_Male - Low_Male; Medium_Male - Medium_Female	A_24_P366509	NM_001010855	NM_001010855
110	TSIX		4.1833	0.0029182	2.5349	0.13262	High_Female - High_Male; High_Female - Low_Female; High_Female - Low_Male; High_Female - Medium_Female; High_Female - Medium_Male; Low_Female - High_Male; High_Male - Low_Male; High_Male - Medium_Female; High_Male - Medium_Male; Low_Female - Low_Male; Low_Female - Medium_Female; Low_Female - Medium_Male; Medium_Female - Low_Male; Medium_Male - Low_Male; Medium_Female - Medium_Male	A_19_P00316565	NR_003255	NR_003255

111	USP9Y	ubiquitin specific peptidase 9, Y-linked	4.1761	0.002951	2.53	0.1329	High_Male - High_Female; Low_Female - High_Female; Low_Male - High_Female; Medium_Female - High_Female; Medium_Male - High_Female; Low_Female - High_Male; Low_Male - High_Male; Medium_Female - High_Male; Medium_Male - High_Male; Low_Male - Low_Female; Low_Female - Medium_Female; Medium_Male - Low_Female; Low_Male - Medium_Female; Low_Male - Medium_Male; Medium_Male - Medium_Female	A_33_P3725324	NM_004654	NM_004654
112	TAGAP	T-cell activation RhoGTPase activating protein	4.1487	0.0030791	2.5116	0.13743	High_Female - High_Male; Low_Female - High_Female; Low_Male - High_Female; Medium_Female - High_Female; Medium_Male - High_Female; Low_Female - High_Male; Low_Male - High_Male; Medium_Female - High_Male; Medium_Male - High_Male; Low_Female - Low_Male; Low_Female - Medium_Female; Low_Female - Medium_Male; Low_Male - Medium_Female; Low_Male - Medium_Male; Medium_Female - Medium_Male	A_23_P339588	NM_138810	NM_138810

113	CELA2B	chymotrypsin-like elastase family, member 2B	4.1285	0.0031769	2.498	0.14054	High_Male - High_Female; Low_Female - High_Female; Low_Male - High_Female; Medium_Female - High_Female; Medium_Male - High_Female; Low_Female - High_Male; High_Male - Low_Male; High_Male - Medium_Female; High_Male - Medium_Male; Low_Female - Low_Male; Low_Female - Medium_Female; Low_Female - Medium_Male; Medium_Female - Low_Male; Low_Male - Medium_Male; Medium_Female - Medium_Male	A_23_P51711	NM_015849	NM_015849
114	MMP19	matrix metallopeptidase 19	4.113	0.0032544	2.4875	0.14271	High_Male - High_Female; Low_Female - High_Female; Low_Male - High_Female; Medium_Female - High_Female; Medium_Male - High_Female; Low_Female - High_Male; Low_Male - High_Male; High_Male - Medium_Female; High_Male - Medium_Male; Low_Female - Low_Male; Low_Female - Medium_Female; Low_Female - Medium_Male; Low_Male - Medium_Female; Low_Male - Medium_Male; Medium_Female - Medium_Male	A_24_P184445	NM_002429	NM_002429

115	BMS1P5	BMS1 pseudogene 5	4.1046	0.0032972	2.4819	0.14312	High_Male - High_Female; Low_Female - High_Female; Low_Male - High_Female; Medium_Female - High_Female; Medium_Male - High_Female; Low_Female - High_Male; Low_Male - High_Male; Medium_Female - High_Male; Medium_Male - High_Male; Low_Male - Low_Female; Low_Female - Medium_Female; Medium_Male - Low_Female; Low_Male - Medium_Female; Low_Male - Medium_Male; Medium_Male - Medium_Female	A_21_P0010878		BC065722
116	XLOC_005505		4.0999	0.0033212	2.4787	0.14312	High_Male - High_Female; High_Female - Low_Female; Low_Male - High_Female; Medium_Female - High_Female; Medium_Male - High_Female; High_Male - Low_Female; High_Male - Low_Male; High_Male - Medium_Female; Medium_Male - High_Male; Low_Male - Low_Female; Medium_Female - Low_Female; Medium_Male - Low_Female; Low_Male - Medium_Female; Medium_Male - Low_Male; Medium_Male - Medium_Female	A_21_P0004605		CR607309

117	NR4A2	nuclear receptor subfamily 4, group A, member 2	4.0884	0.0033811	2.4709	0.14446	High_Male - High_Female; Low_Female - High_Female; Low_Male - High_Female; Medium_Female - High_Female; Medium_Male - High_Female; High_Male - Low_Female; Low_Male - High_Male; High_Male - Medium_Female; High_Male - Medium_Male; Low_Male - Low_Female; Low_Female - Medium_Female; Medium_Male - Low_Female; Low_Male - Medium_Female; Low_Male - Medium_Male; Medium_Male - Medium_Female	A_33_P3299066	NM_006186	NM_006186
118	XLOC_009548		4.0826	0.0034122	2.467	0.14455	High_Female - High_Male; Low_Female - High_Female; Low_Male - High_Female; Medium_Female - High_Female; High_Female - Medium_Male; Low_Female - High_Male; Low_Male - High_Male; Medium_Female - High_Male; High_Male - Medium_Male; Low_Male - Low_Female; Medium_Female - Low_Female; Low_Female - Medium_Male; Medium_Female - Low_Male; Low_Male - Medium_Male; Medium_Female - Medium_Male	linc|TCONS_00019754		

119	XLOC_002172		4.0714	0.0034717	2.4595	0.14493	High_Male - High_Female; Low_Female - High_Female; Low_Male - High_Female; Medium_Female - High_Female; Medium_Male - High_Female; Low_Female - High_Male; Low_Male - High_Male; High_Male - Medium_Female; High_Male - Medium_Male; Low_Female - Low_Male; Low_Female - Medium_Female; Low_Female - Medium_Male; Low_Male - Medium_Female; Low_Male - Medium_Male; Medium_Female - Medium_Male	ens|ENST00000437233|linc|TCONS_00003324		
120	CD69	CD69 molecule	4.0651	0.0035063	2.4551	0.14493	High_Male - High_Female; Low_Female - High_Female; Low_Male - High_Female; High_Female - Medium_Female; Medium_Male - High_Female; High_Male - Low_Female; Low_Male - High_Male; High_Male - Medium_Female; High_Male - Medium_Male; Low_Male - Low_Female; Low_Female - Medium_Female; Medium_Male - Low_Female; Low_Male - Medium_Female; Low_Male - Medium_Male; Medium_Male - Medium_Female	A_23_P87879	NM_001781	NM_001781

121	DOCK9	dedicator of cytokinesis 9	4.0647	0.0035081	2.4549	0.14493	High_Male - High_Female; Low_Female - High_Female; Low_Male - High_Female; High_Female - Medium_Female; High_Female - Medium_Male; Low_Female - High_Male; Low_Male - High_Male; High_Male - Medium_Female; High_Male - Medium_Male; Low_Female - Low_Male; Low_Female - Medium_Female; Low_Female - Medium_Male; Low_Male - Medium_Female; Low_Male - Medium_Male; Medium_Female - Medium_Male	A_33_P3410279		AK090793
122	PTGDR2	prostaglandin D2 receptor 2	4.0533	0.0035713	2.4472	0.14559	High_Male - High_Female; Low_Female - High_Female; Low_Male - High_Female; Medium_Female - High_Female; Medium_Male - High_Female; High_Male - Low_Female; High_Male - Low_Male; High_Male - Medium_Female; High_Male - Medium_Male; Low_Male - Low_Female; Medium_Female - Low_Female; Medium_Male - Low_Female; Medium_Female - Low_Male; Medium_Male - Low_Male; Medium_Male - Medium_Female	A_24_P115932	NM_004778	NM_004778

123	PRSS33	protease, serine, 33	4.0509	0.0035843	2.4456	0.14559	High_Male - High_Female; High_Female - Low_Female; Low_Male - High_Female; Medium_Female - High_Female; Medium_Male - High_Female; High_Male - Low_Female; High_Male - Low_Male; High_Male - Medium_Female; High_Male - Medium_Male; Low_Male - Low_Female; Medium_Female - Low_Female; Medium_Male - Low_Female; Medium_Female - Low_Male; Medium_Male - Low_Male; Medium_Male - Medium_Female	A_33_P3312779	NM_152891	NM_152891
124	DISC1	disrupted in schizophrenia 1	4.0461	0.0036113	2.4423	0.14559	High_Female - High_Male; High_Female - Low_Female; High_Female - Low_Male; High_Female - Medium_Female; High_Female - Medium_Male; Low_Female - High_Male; High_Male - Low_Male; Medium_Female - High_Male; High_Male - Medium_Male; Low_Female - Low_Male; Low_Female - Medium_Female; Low_Female - Medium_Male; Medium_Female - Low_Male; Medium_Male - Low_Male; Medium_Female - Medium_Male	A_21_P0000052	NM_001164554	NM_001164554

125	BAMBI	BMP and activin membrane-bound inhibitor homolog (Xenopus laevis)	4.0261	0.0037256	2.4288	0.14899	High_Female - High_Male; High_Female - Low_Female; High_Female - Low_Male; High_Female - Medium_Female; Medium_Male - High_Female; High_Male - Low_Female; High_Male - Low_Male; High_Male - Medium_Female; Medium_Male - High_Male; Low_Female - Low_Male; Medium_Female - Low_Female; Medium_Male - Low_Female; Medium_Female - Low_Male; Medium_Male - Low_Male; Medium_Male - Medium_Female	A_23_P52207	NM_012342	NM_012342
126	AKNA	AT-hook transcription factor	4.0191	0.0037665	2.4241	0.14943	High_Male - High_Female; Low_Female - High_Female; Low_Male - High_Female; Medium_Female - High_Female; Medium_Male - High_Female; Low_Female - High_Male; Low_Male - High_Male; High_Male - Medium_Female; Medium_Male - High_Male; Low_Male - Low_Female; Low_Female - Medium_Female; Low_Female - Medium_Male; Low_Male - Medium_Female; Low_Male - Medium_Male; Medium_Male - Medium_Female	A_24_P912439		AB075848

127	CCL4	chemokine (C-C motif) ligand 4	3.9996	0.0038833	2.4108	0.15285	High_Male - High_Female; Low_Female - High_Female; Low_Male - High_Female; Medium_Female - High_Female; Medium_Male - High_Female; High_Male - Low_Female; Low_Male - High_Male; High_Male - Medium_Female; High_Male - Medium_Male; Low_Male - Low_Female; Low_Female - Medium_Female; Medium_Male - Low_Female; Low_Male - Medium_Female; Low_Male - Medium_Male; Medium_Male - Medium_Female	A_23_P207564	NM_002984	NM_002984
128	LRRN3	leucine rich repeat neuronal 3	3.9862	0.0039649	2.4018	0.15448	High_Male - High_Female; Low_Female - High_Female; Low_Male - High_Female; Medium_Female - High_Female; Medium_Male - High_Female; High_Male - Low_Female; Low_Male - High_Male; High_Male - Medium_Female; High_Male - Medium_Male; Low_Male - Low_Female; Low_Female - Medium_Female; Low_Female - Medium_Male; Low_Male - Medium_Female; Low_Male - Medium_Male; Medium_Male - Medium_Female	A_23_P31376	NM_018334	NM_018334

129	C3AR1	complement component 3a receptor 1	3.9828	0.0039863	2.3994	0.15448	High_Male - High_Female; Low_Female - High_Female; Low_Male - High_Female; Medium_Female - High_Female; Medium_Male - High_Female; High_Male - Low_Female; High_Male - Low_Male; High_Male - Medium_Female; High_Male - Medium_Male; Low_Female - Low_Male; Low_Female - Medium_Female; Medium_Male - Low_Female; Medium_Female - Low_Male; Medium_Male - Low_Male; Medium_Male - Medium_Female	A_23_P2431	NM_004054	NM_004054
130	XLOC_001595		3.9372	0.0042805	2.3685	0.16456	High_Female - High_Male; High_Female - Low_Female; High_Female - Low_Male; High_Female - Medium_Female; High_Female - Medium_Male; High_Male - Low_Female; Low_Male - High_Male; High_Male - Medium_Female; Medium_Male - High_Male; Low_Male - Low_Female; Medium_Female - Low_Female; Medium_Male - Low_Female; Low_Male - Medium_Female; Medium_Male - Low_Male; Medium_Male - Medium_Female	linc|TCONS_00003799		

131	CCL4	chemokine (C-C motif) ligand 4	3.9325	0.0043123	2.3653	0.16456	High_Male - High_Female;<br>Low_Female - High_Female;<br>Low_Male - High_Female;<br>Medium_Female - High_Female;<br>Medium_Male - High_Female;<br>High_Male - Low_Female;<br>Low_Male - High_Male;<br>High_Male - Medium_Female;<br>High_Male - Medium_Male;<br>Low_Male - Low_Female;<br>Low_Female - Medium_Female;<br>Medium_Male - Low_Female;<br>Low_Male - Medium_Female;<br>Low_Male - Medium_Male;<br>Medium_Male - Medium_Female	A_33_P3354607	NM_002984	NM_002984
132	RAB44	RAB44, member RAS oncogene family	3.916	0.004425	2.3541	0.16758	High_Male - High_Female;<br>High_Female - Low_Female;<br>Low_Male - High_Female;<br>Medium_Female - High_Female;<br>Medium_Male - High_Female;<br>High_Male - Low_Female;<br>High_Male - Low_Male;<br>High_Male - Medium_Female;<br>Medium_Male - High_Male;<br>Low_Male - Low_Female;<br>Medium_Female - Low_Female;<br>Medium_Male - Low_Female;<br>Low_Male - Medium_Female;<br>Medium_Male - Low_Male;<br>Medium_Male - Medium_Female	A_33_P3424328	XM_003403441	XM_003403441

133	CEBPE	CCAAT/enhancer binding protein (C/EBP), epsilon	3.9103	0.0044644	2.3502	0.16771	High_Male - High_Female; High_Female - Low_Female; Low_Male - High_Female; Medium_Female - High_Female; Medium_Male - High_Female; High_Male - Low_Female; High_Male - Low_Male; High_Male - Medium_Female; High_Male - Medium_Male; Low_Male - Low_Female; Medium_Female - Low_Female; Medium_Male - Low_Female; Medium_Female - Low_Male; Medium_Male - Low_Male; Medium_Male - Medium_Female	A_33_P3387991	NM_001805	NM_001805
134	LOC643802	u3 small nucleolar ribonucleoprotein protein MPP10-like	3.9059	0.0044956	2.3472	0.16771	High_Female - High_Male; High_Female - Low_Female; High_Female - Low_Male; High_Female - Medium_Female; High_Female - Medium_Male; High_Male - Low_Female; High_Male - Low_Male; High_Male - Medium_Female; High_Male - Medium_Male; Low_Female - Low_Male; Medium_Female - Low_Female; Medium_Male - Low_Female; Medium_Female - Low_Male; Medium_Male - Low_Male; Medium_Male - Medium_Female	A_21_P0011505		BX537874

135	RAP1GAP2	RAP1 GTPase activating protein 2	3.8695	0.0047595	2.3224	0.17615	High_Female - High_Male; High_Female - Low_Female; High_Female - Low_Male; High_Female - Medium_Female; High_Female - Medium_Male; High_Male - Low_Female; High_Male - Low_Male; High_Male - Medium_Female; High_Male - Medium_Male; Low_Male - Low_Female; Medium_Female - Low_Female; Medium_Male - Low_Female; Medium_Female - Low_Male; Medium_Male - Low_Male; Medium_Male - Medium_Female	A_23_P412214	NM_015085	NM_015085
136	LOC100129534	small nuclear ribonucleoprotein polypeptide N pseudogene	3.8651	0.0047922	2.3195	0.17615	High_Male - High_Female; Low_Female - High_Female; Low_Male - High_Female; Medium_Female - High_Female; Medium_Male - High_Female; Low_Female - High_Male; Low_Male - High_Male; Medium_Female - High_Male; High_Male - Medium_Male; Low_Male - Low_Female; Low_Female - Medium_Female; Low_Female - Medium_Male; Low_Male - Medium_Female; Low_Male - Medium_Male; Medium_Female - Medium_Male	A_33_P3271395	NR_024489	NR_024489

137	VSTM1	V-set and transmembrane domain containing 1	3.8472	0.0049288	2.3073	0.17966	High_Male - High_Female; Low_Female - High_Female; Low_Male - High_Female; Medium_Female - High_Female; Medium_Male - High_Female; High_Male - Low_Female; High_Male - Low_Male; High_Male - Medium_Female; Medium_Male - High_Male; Low_Male - Low_Female; Medium_Female - Low_Female; Medium_Male - Low_Female; Medium_Female - Low_Male; Medium_Male - Low_Male; Medium_Male - Medium_Female	A_33_P3514487	NM_198481	NM_198481
138			3.8416	0.0049723	2.3034	0.17966	High_Female - High_Male; High_Female - Low_Female; High_Female - Low_Male; High_Female - Medium_Female; High_Female - Medium_Male; Low_Female - High_Male; High_Male - Low_Male; Medium_Female - High_Male; High_Male - Medium_Male; Low_Female - Low_Male; Medium_Female - Low_Female; Low_Female - Medium_Male; Medium_Female - Low_Male; Medium_Male - Low_Male; Medium_Female - Medium_Male	A_33_P3343394		

139	CCR3	chemokine (C-C motif) receptor 3	3.8386	0.0049957	2.3014	0.17966	High_Male - High_Female; Low_Female - High_Female; Low_Male - High_Female; Medium_Female - High_Female; Medium_Male - High_Female; High_Male - Low_Female; High_Male - Low_Male; High_Male - Medium_Female; High_Male - Medium_Male; Low_Male - Low_Female; Medium_Female - Low_Female; Medium_Male - Low_Female; Low_Male - Medium_Female; Medium_Male - Low_Male; Medium_Male - Medium_Female	A_23_P250302	NM_001837	NM_001837
140	LTC4S	leukotriene C4 synthase	3.8276	0.0050824	2.2939	0.18083	High_Male - High_Female; High_Female - Low_Female; Low_Male - High_Female; Medium_Female - High_Female; Medium_Male - High_Female; High_Male - Low_Female; High_Male - Low_Male; High_Male - Medium_Female; Medium_Male - High_Male; Low_Male - Low_Female; Medium_Female - Low_Female; Medium_Male - Low_Female; Low_Male - Medium_Female; Medium_Male - Low_Male; Medium_Male - Medium_Female	A_24_P397294	NM_145867	NM_145867

141	PMP22	peripheral myelin protein 22	3.8254	0.0051006	2.2924	0.18083	High_Male - High_Female;<br>Low_Female - High_Female;<br>Low_Male - High_Female;<br>Medium_Female - High_Female;<br>Medium_Male - High_Female;<br>High_Male - Low_Female;<br>High_Male - Low_Male;<br>High_Male - Medium_Female;<br>High_Male - Medium_Male;<br>Low_Male - Low_Female;<br>Medium_Female - Low_Female;<br>Medium_Male - Low_Female;<br>Medium_Female - Low_Male;<br>Medium_Male - Low_Male;<br>Medium_Male - Medium_Female	A_23_P100711	NM_000304	NM_000304
142	KCNJ15	potassium inwardly-rectifying channel, subfamily J, member 15	3.8091	0.0052328	2.2813	0.18302	High_Female - High_Male;<br>High_Female - Low_Female;<br>High_Female - Low_Male;<br>High_Female - Medium_Female;<br>High_Female - Medium_Male;<br>Low_Female - High_Male;<br>High_Male - Low_Male;<br>Medium_Female - High_Male;<br>High_Male - Medium_Male;<br>Low_Female - Low_Male;<br>Medium_Female - Low_Female;<br>Low_Female - Medium_Male;<br>Medium_Female - Low_Male;<br>Medium_Male - Low_Male;<br>Medium_Female - Medium_Male	A_33_P3376140	NM_170736	NM_170736

143	XLOC_006338		3.8084	0.005238	2.2808	0.18302	High_Female - High_Male; High_Female - Low_Female; High_Female - Low_Male; High_Female - Medium_Female; High_Female - Medium_Male; High_Male - Low_Female; High_Male - Low_Male; High_Male - Medium_Female; Medium_Male - High_Male; Low_Female - Low_Male; Low_Female - Medium_Female; Medium_Male - Low_Female; Medium_Female - Low_Male; Medium_Male - Low_Male; Medium_Male - Medium_Female	linc|TCONS_00014249|linc|TCONS_00013675		
144	CYP1B1-AS1		3.8043	0.0052721	2.278	0.18302	High_Female - High_Male; High_Female - Low_Female; High_Female - Low_Male; High_Female - Medium_Female; High_Female - Medium_Male; High_Male - Low_Female; High_Male - Low_Male; High_Male - Medium_Female; Medium_Male - High_Male; Low_Male - Low_Female; Medium_Female - Low_Female; Medium_Male - Low_Female; Medium_Female - Low_Male; Medium_Male - Low_Male; Medium_Male - Medium_Female	A_19_P00316135	NR_027252	NR_027252

145	XLOC_l2_015752		3.7944	0.005355	2.2712	0.18462	High_Male - High_Female; Low_Female - High_Female; Low_Male - High_Female; Medium_Female - High_Female; Medium_Male - High_Female; Low_Female - High_Male; Low_Male - High_Male; High_Male - Medium_Female; High_Male - Medium_Male; Low_Female - Low_Male; Low_Female - Medium_Female; Low_Female - Medium_Male; Low_Male - Medium_Female; Low_Male - Medium_Male; Medium_Female - Medium_Male	linc|TCONS_l2_00030522		
146	XLOC_000683		3.7876	0.0054124	2.2666	0.18532	High_Female - High_Male; High_Female - Low_Female; High_Female - Low_Male; High_Female - Medium_Female; High_Female - Medium_Male; High_Male - Low_Female; High_Male - Low_Male; High_Male - Medium_Female; Medium_Male - High_Male; Low_Female - Low_Male; Low_Female - Medium_Female; Medium_Male - Low_Female; Medium_Female - Low_Male; Medium_Male - Low_Male; Medium_Male - Medium_Female	A_21_P0001093		BQ637851

147	OPLAH	5-oxoprolinase (ATP-hydrolysing)	3.7676	0.0055854	2.2529	0.18814	High_Female - High_Male; High_Female - Low_Female; High_Female - Low_Male; High_Female - Medium_Female; High_Female - Medium_Male; High_Male - Low_Female; High_Male - Low_Male; High_Male - Medium_Female; High_Male - Medium_Male; Low_Female - Low_Male; Medium_Female - Low_Female; Low_Female - Medium_Male; Medium_Female - Low_Male; Medium_Male - Low_Male; Medium_Female - Medium_Male	A_23_P170186	NM_017570	NM_017570
148	ALAS2	aminolevulinate, delta-, synthase 2	3.7653	0.0056058	2.2514	0.18814	High_Female - High_Male; High_Female - Low_Female; High_Female - Low_Male; High_Female - Medium_Female; High_Female - Medium_Male; Low_Female - High_Male; High_Male - Low_Male; Medium_Female - High_Male; Medium_Male - High_Male; Low_Female - Low_Male; Medium_Female - Low_Female; Medium_Male - Low_Female; Medium_Female - Low_Male; Medium_Male - Low_Male; Medium_Female - Medium_Male	A_32_P385587	NM_000032	NM_000032

149	PRSS41	protease, serine, 41	3.7651	0.0056078	2.2512	0.18814	High_Male - High_Female;<br>High_Female - Low_Female;<br>Low_Male - High_Female;<br>Medium_Female - High_Female;<br>Medium_Male - High_Female;<br>High_Male - Low_Female;<br>High_Male - Low_Male;<br>High_Male - Medium_Female;<br>Medium_Male - High_Male;<br>Low_Male - Low_Female;<br>Medium_Female - Low_Female;<br>Medium_Male - Low_Female;<br>Low_Male - Medium_Female;<br>Medium_Male - Low_Male;<br>Medium_Male - Medium_Female	A_33_P3416634	NM_001135086	NM_001135086
150	MXI1	MAX interactor 1	3.7556	0.005692	2.2447	0.18824	High_Female - High_Male;<br>Low_Female - High_Female;<br>Low_Male - High_Female;<br>Medium_Female - High_Female;<br>Medium_Male - High_Female;<br>Low_Female - High_Male;<br>Low_Male - High_Male;<br>Medium_Female - High_Male;<br>Medium_Male - High_Male;<br>Low_Female - Low_Male;<br>Medium_Female - Low_Female;<br>Medium_Male - Low_Female;<br>Medium_Female - Low_Male;<br>Medium_Male - Low_Male;<br>Medium_Female - Medium_Male	A_33_P3383029	NM_130439	NM_130439

151	TRIB1	tribbles homolog 1 (Drosophila)	3.7522	0.0057225	2.2424	0.18824	High_Male - High_Female; Low_Female - High_Female; Low_Male - High_Female; Medium_Female - High_Female; Medium_Male - High_Female; High_Male - Low_Female; Low_Male - High_Male; High_Male - Medium_Female; High_Male - Medium_Male; Low_Male - Low_Female; Low_Female - Medium_Female; Low_Female - Medium_Male; Low_Male - Medium_Female; Low_Male - Medium_Male; Medium_Male - Medium_Female	A_24_P252497	NM_025195	NM_025195
152	XLOC_008015		3.7521	0.0057235	2.2423	0.18824	High_Female - High_Male; High_Female - Low_Female; High_Female - Low_Male; High_Female - Medium_Female; High_Female - Medium_Male; Low_Female - High_Male; High_Male - Low_Male; Medium_Female - High_Male; High_Male - Medium_Male; Low_Female - Low_Male; Medium_Female - Low_Female; Low_Female - Medium_Male; Medium_Female - Low_Male; Low_Male - Medium_Male; Medium_Female - Medium_Male	linc|TCONS_00017433		

153	DSP	desmoplakin	3.7257	0.0059659	2.2243	0.19492	High_Male - High_Female; Low_Female - High_Female; Low_Male - High_Female; Medium_Female - High_Female; Medium_Male - High_Female; Low_Female - High_Male; Low_Male - High_Male; Medium_Female - High_Male; Medium_Male - High_Male; Low_Male - Low_Female; Medium_Female - Low_Female; Medium_Male - Low_Female; Medium_Female - Low_Male; Medium_Male - Low_Male; Medium_Male - Medium_Female	A_32_P157945	NM_004415	NM_004415
154	XLOC_l2_000253		3.7072	0.0061426	2.2116	0.198	High_Male - High_Female; Low_Female - High_Female; Low_Male - High_Female; Medium_Female - High_Female; Medium_Male - High_Female; High_Male - Low_Female; High_Male - Low_Male; High_Male - Medium_Female; High_Male - Medium_Male; Low_Female - Low_Male; Low_Female - Medium_Female; Low_Female - Medium_Male; Medium_Female - Low_Male; Low_Male - Medium_Male; Medium_Female - Medium_Male	A_21_P0010532		BX117229

155	LIMS3-LOC440895	LIMS3-LOC440895 readthrough	3.7041	0.0061728	2.2095	0.198	High_Female - High_Male; Low_Female - High_Female; High_Female - Low_Male; High_Female - Medium_Female; High_Female - Medium_Male; Low_Female - High_Male; High_Male - Low_Male; High_Male - Medium_Female; Medium_Male - High_Male; Low_Female - Low_Male; Low_Female - Medium_Female; Low_Female - Medium_Male; Low_Male - Medium_Female; Medium_Male - Low_Male; Medium_Male - Medium_Female	A_21_P0000527	NR_027145	NR_027145
156	GPR34	G protein-coupled receptor 34	3.6992	0.0062206	2.2062	0.198	High_Male - High_Female; Low_Female - High_Female; Low_Male - High_Female; Medium_Female - High_Female; Medium_Male - High_Female; High_Male - Low_Female; High_Male - Low_Male; High_Male - Medium_Female; High_Male - Medium_Male; Low_Male - Low_Female; Medium_Female - Low_Female; Medium_Male - Low_Female; Low_Male - Medium_Female; Medium_Male - Low_Male; Medium_Male - Medium_Female	A_23_P11201	NM_001097579	NM_001097579

157	PSPH	phosphoserine phosphatase	3.6957	0.0062546	2.2038	0.198	High_Male - High_Female; High_Female - Low_Female; High_Female - Low_Male; Medium_Female - High_Female; Medium_Male - High_Female; High_Male - Low_Female; High_Male - Low_Male; Medium_Female - High_Male; Medium_Male - High_Male; Low_Male - Low_Female; Medium_Female - Low_Female; Medium_Male - Low_Female; Medium_Female - Low_Male; Medium_Male - Low_Male; Medium_Female - Medium_Male	A_32_P78816	NM_004577	NM_004577
158	DUSP1	dual specificity phosphatase 1	3.6954	0.0062581	2.2036	0.198	High_Female - High_Male; Low_Female - High_Female; Low_Male - High_Female; Medium_Female - High_Female; Medium_Male - High_Female; Low_Female - High_Male; Low_Male - High_Male; Medium_Female - High_Male; Medium_Male - High_Male; Low_Male - Low_Female; Low_Female - Medium_Female; Low_Female - Medium_Male; Low_Male - Medium_Female; Low_Male - Medium_Male; Medium_Female - Medium_Male	A_23_P110712	NM_004417	NM_004417

159	TPTE2P6	transmembrane phosphoinositide 3-phosphatase and tensin homolog 2 pseudogene 6	3.6827	0.006385	2.1948	0.20028	High_Male - High_Female; Low_Female - High_Female; Low_Male - High_Female; Medium_Female - High_Female; High_Female - Medium_Male; Low_Female - High_Male; High_Male - Low_Male; High_Male - Medium_Female; High_Male - Medium_Male; Low_Female - Low_Male; Low_Female - Medium_Female; Low_Female - Medium_Male; Medium_Female - Low_Male; Low_Male - Medium_Male; Medium_Female - Medium_Male	A_32_P89827	NR_002815	NR_002815
160			3.6801	0.0064104	2.1931	0.20028	High_Male - High_Female; Low_Female - High_Female; Low_Male - High_Female; Medium_Female - High_Female; Medium_Male - High_Female; Low_Female - High_Male; Low_Male - High_Male; High_Male - Medium_Female; High_Male - Medium_Male; Low_Male - Low_Female; Low_Female - Medium_Female; Low_Female - Medium_Male; Low_Male - Medium_Female; Low_Male - Medium_Male; Medium_Female - Medium_Male	ens|ENST00000433238		

161			3.6762	0.0064507	2.1904	0.20029	High_Female - High_Male; High_Female - Low_Female; High_Female - Low_Male; Medium_Female - High_Female; High_Female - Medium_Male; Low_Female - High_Male; Low_Male - High_Male; Medium_Female - High_Male; High_Male - Medium_Male; Low_Male - Low_Female; Medium_Female - Low_Female; Low_Female - Medium_Male; Medium_Female - Low_Male; Low_Male - Medium_Male; Medium_Female - Medium_Male	A_33_P3399755		
162	LOC10050689 7		3.6569	0.0066502	2.1772	0.20449	High_Male - High_Female; Low_Female - High_Female; Low_Male - High_Female; Medium_Female - High_Female; Medium_Male - High_Female; High_Male - Low_Female; High_Male - Low_Male; High_Male - Medium_Female; Medium_Male - High_Male; Low_Female - Low_Male; Low_Female - Medium_Female; Medium_Male - Low_Female; Low_Male - Medium_Female; Medium_Male - Low_Male; Medium_Male - Medium_Female	A_21_P0014234	XR_133523	XR_133523

163	XLOC_003650		3.6514	0.0067084	2.1734	0.20449	High_Male - High_Female;<br>Low_Female - High_Female;<br>Low_Male - High_Female;<br>Medium_Female - High_Female;<br>High_Female - Medium_Male;<br>Low_Female - High_Male;<br>High_Male - Low_Male;<br>High_Male - Medium_Female;<br>High_Male - Medium_Male;<br>Low_Female - Low_Male;<br>Low_Female - Medium_Female;<br>Low_Female - Medium_Male;<br>Low_Male - Medium_Female;<br>Low_Male - Medium_Male;<br>Medium_Female - Medium_Male	A_21_P0003848		CR742343
164	XLOC_011248		3.6513	0.0067086	2.1734	0.20449	High_Female - High_Male;<br>Low_Female - High_Female;<br>Low_Male - High_Female;<br>Medium_Female - High_Female;<br>Medium_Male - High_Female;<br>Low_Female - High_Male;<br>Low_Male - High_Male;<br>Medium_Female - High_Male;<br>Medium_Male - High_Male;<br>Low_Male - Low_Female;<br>Medium_Female - Low_Female;<br>Medium_Male - Low_Female;<br>Low_Male - Medium_Female;<br>Low_Male - Medium_Male;<br>Medium_Female - Medium_Male	A_21_P0008570		BC035091

165	LINC00230A	long intergenic non-protein coding RNA 230A	3.6343	0.0068914	2.1617	0.2066	High_Male - High_Female; Low_Female - High_Female; Low_Male - High_Female; Medium_Female - High_Female; Medium_Male - High_Female; Low_Female - High_Male; Low_Male - High_Male; Medium_Female - High_Male; Medium_Male - High_Male; Low_Male - Low_Female; Low_Female - Medium_Female; Medium_Male - Low_Female; Low_Male - Medium_Female; Medium_Male - Low_Male; Medium_Male - Medium_Female	A_33_P3308232	NR_002161	NR_002161
166	RFPL4A	ret finger protein-like 4A	3.6322	0.006914	2.1603	0.2066	High_Female - High_Male; Low_Female - High_Female; High_Female - Low_Male; High_Female - Medium_Female; Medium_Male - High_Female; Low_Female - High_Male; Low_Male - High_Male; Medium_Female - High_Male; Medium_Male - High_Male; Low_Female - Low_Male; Low_Female - Medium_Female; Medium_Male - Low_Female; Medium_Female - Low_Male; Medium_Male - Low_Male; Medium_Male - Medium_Female	A_33_P3366127	NM_001145014	NM_001145014

167	ANXA3	annexin A3	3.6309	0.0069288	2.1593	0.2066	High_Female - High_Male; High_Female - Low_Female; High_Female - Low_Male; High_Female - Medium_Female; High_Female - Medium_Male; Low_Female - High_Male; High_Male - Low_Male; Medium_Female - High_Male; High_Male - Medium_Male; Low_Female - Low_Male; Low_Female - Medium_Female; Low_Female - Medium_Male; Medium_Female - Low_Male; Medium_Male - Low_Male; Medium_Female - Medium_Male	A_23_P121716	NM_005139	NM_005139
168			3.6296	0.0069431	2.1584	0.2066	High_Female - High_Male; High_Female - Low_Female; High_Female - Low_Male; Medium_Female - High_Female; High_Female - Medium_Male; Low_Female - High_Male; Low_Male - High_Male; Medium_Female - High_Male; Medium_Male - High_Male; Low_Male - Low_Female; Medium_Female - Low_Female; Low_Female - Medium_Male; Medium_Female - Low_Male; Low_Male - Medium_Male; Medium_Female - Medium_Male	A_33_P3422712		

169	XLOC_l2_004546		3.5933	0.0073535	2.1335	0.21751	High_Male - High_Female; Low_Female - High_Female; Low_Male - High_Female; Medium_Female - High_Female; Medium_Male - High_Female; High_Male - Low_Female; Low_Male - High_Male; Medium_Female - High_Male; High_Male - Medium_Male; Low_Male - Low_Female; Medium_Female - Low_Female; Low_Female - Medium_Male; Low_Male - Medium_Female; Low_Male - Medium_Male; Medium_Female - Medium_Male	linc|TCONS_l2_00008281		
170	LOC100131510	uncharacterized LOC100131510	3.5881	0.0074136	2.13	0.21778	High_Male - High_Female; Low_Female - High_Female; High_Female - Low_Male; High_Female - Medium_Female; High_Female - Medium_Male; Low_Female - High_Male; High_Male - Low_Male; High_Male - Medium_Female; High_Male - Medium_Male; Low_Female - Low_Male; Low_Female - Medium_Female; Low_Female - Medium_Male; Medium_Female - Low_Male; Low_Male - Medium_Male; Medium_Female - Medium_Male	A_24_P323754	XR_109960	XR_109960

171	BACE2	beta-site APP-cleaving enzyme 2	3.5787	0.0075249	2.1235	0.21778	High_Male - High_Female; Low_Female - High_Female; Low_Male - High_Female; Medium_Female - High_Female; Medium_Male - High_Female; High_Male - Low_Female; High_Male - Low_Male; High_Male - Medium_Female; Medium_Male - High_Male; Low_Male - Low_Female; Medium_Female - Low_Female; Medium_Male - Low_Female; Low_Male - Medium_Female; Medium_Male - Low_Male; Medium_Male - Medium_Female	A_23_P154875	NM_012105	NM_012105
172			3.578	0.0075328	2.123	0.21778	High_Female - High_Male; Low_Female - High_Female; High_Female - Low_Male; Medium_Female - High_Female; High_Female - Medium_Male; Low_Female - High_Male; High_Male - Low_Male; Medium_Female - High_Male; High_Male - Medium_Male; Low_Female - Low_Male; Medium_Female - Low_Female; Low_Female - Medium_Male; Medium_Female - Low_Male; Medium_Male - Low_Male; Medium_Female - Medium_Male	A_33_P3387016		

173	CRABP2	cellular retinoic acid binding protein 2	3.5777	0.0075366	2.1228	0.21778	High_Male - High_Female; Low_Female - High_Female; Low_Male - High_Female; Medium_Female - High_Female; Medium_Male - High_Female; Low_Female - High_Male; High_Male - Low_Male; Medium_Female - High_Male; High_Male - Medium_Male; Low_Female - Low_Male; Low_Female - Medium_Female; Low_Female - Medium_Male; Medium_Female - Low_Male; Low_Male - Medium_Male; Medium_Female - Medium_Male	A_33_P3294826	NM_001199723	NM_001199723
174	TSIX	TSIX transcript, XIST antisense RNA (non-protein coding)	3.5722	0.0076031	2.119	0.21843	High_Female - High_Male; High_Female - Low_Female; High_Female - Low_Male; High_Female - Medium_Female; High_Female - Medium_Male; Low_Female - High_Male; High_Male - Low_Male; Medium_Female - High_Male; High_Male - Medium_Male; Low_Female - Low_Male; Low_Female - Medium_Female; Low_Female - Medium_Male; Medium_Female - Low_Male; Medium_Male - Low_Male; Medium_Female - Medium_Male	A_33_P3405911	NR_003255	NR_003255

175	XLOC_010052		3.5613	0.007735	2.1115	0.22028	High_Male - High_Female; Low_Female - High_Female; High_Female - Low_Male; High_Female - Medium_Female; Medium_Male - High_Female; Low_Female - High_Male; High_Male - Low_Male; High_Male - Medium_Female; High_Male - Medium_Male; Low_Female - Low_Male; Low_Female - Medium_Female; Low_Female - Medium_Male; Medium_Female - Low_Male; Medium_Male - Low_Male; Medium_Male - Medium_Female	linc|TCONS_00020761		
176	SNAR-G2	small ILF3/NF90-associated RNA G2	3.5576	0.0077807	2.109	0.22028	High_Female - High_Male; High_Female - Low_Female; High_Female - Low_Male; High_Female - Medium_Female; High_Female - Medium_Male; Low_Female - High_Male; High_Male - Low_Male; Medium_Female - High_Male; Medium_Male - High_Male; Low_Female - Low_Male; Medium_Female - Low_Female; Medium_Male - Low_Female; Medium_Female - Low_Male; Medium_Male - Low_Male; Medium_Female - Medium_Male	A_21_P0000509	NR_024244	NR_024244

177	XLOC_014215		3.5547	0.0078162	2.107	0.22028	High_Male - High_Female;<br>Low_Female - High_Female;<br>Low_Male - High_Female;<br>Medium_Female - High_Female;<br>Medium_Male - High_Female;<br>Low_Female - High_Male;<br>High_Male - Low_Male;<br>High_Male - Medium_Female;<br>High_Male - Medium_Male;<br>Low_Female - Low_Male;<br>Low_Female - Medium_Female;<br>Low_Female - Medium_Male;<br>Low_Male - Medium_Female;<br>Low_Male - Medium_Male;<br>Medium_Female - Medium_Male	A_21_P0010355		DB089380
178	THBS3	thrombospondin 3	3.5525	0.0078437	2.1055	0.22028	High_Male - High_Female;<br>High_Female - Low_Female;<br>High_Female - Low_Male;<br>Medium_Female - High_Female;<br>High_Female - Medium_Male;<br>High_Male - Low_Female;<br>High_Male - Low_Male;<br>High_Male - Medium_Female;<br>High_Male - Medium_Male;<br>Low_Female - Low_Male;<br>Medium_Female - Low_Female;<br>Medium_Male - Low_Female;<br>Medium_Female - Low_Male;<br>Medium_Male - Low_Male;<br>Medium_Female - Medium_Male	A_33_P3222867		CR933610

179	XLOC_l2_015849		3.5476	0.0079044	2.1021	0.22075	High_Female - High_Male; Low_Female - High_Female; Low_Male - High_Female; High_Female - Medium_Female; Medium_Male - High_Female; Low_Female - High_Male; Low_Male - High_Male; Medium_Female - High_Male; Medium_Male - High_Male; Low_Male - Low_Female; Low_Female - Medium_Female; Medium_Male - Low_Female; Low_Male - Medium_Female; Low_Male - Medium_Male; Medium_Male - Medium_Female	A_21_P0013836		BX111592
180	TTC24	tetratricopeptide repeat domain 24	3.5437	0.0079533	2.0995	0.22088	High_Male - High_Female; Low_Female - High_Female; Low_Male - High_Female; Medium_Female - High_Female; High_Female - Medium_Male; Low_Female - High_Male; High_Male - Low_Male; Medium_Female - High_Male; High_Male - Medium_Male; Low_Female - Low_Male; Low_Female - Medium_Female; Low_Female - Medium_Male; Medium_Female - Low_Male; Low_Male - Medium_Male; Medium_Female - Medium_Male	A_33_P3297345	NM_001105669	NM_001105669

181	ALOX15	arachidonate 15-lipoxygenase	3.5281	0.0081528	2.0887	0.22517	High_Male - High_Female; Low_Female - High_Female; Low_Male - High_Female; Medium_Female - High_Female; Medium_Male - High_Female; High_Male - Low_Female; High_Male - Low_Male; High_Male - Medium_Female; Medium_Male - High_Male; Low_Male - Low_Female; Medium_Female - Low_Female; Medium_Male - Low_Female; Low_Male - Medium_Female; Medium_Male - Low_Male; Medium_Male - Medium_Female	A_23_P55373	NM_001140	NM_001140
182	ABTB2	ankyrin repeat and BTB (POZ) domain containing 2	3.5093	0.0084001	2.0757	0.23073	High_Male - High_Female; Low_Female - High_Female; Low_Male - High_Female; Medium_Female - High_Female; Medium_Male - High_Female; High_Male - Low_Female; High_Male - Low_Male; High_Male - Medium_Female; High_Male - Medium_Male; Low_Male - Low_Female; Medium_Female - Low_Female; Medium_Male - Low_Female; Low_Male - Medium_Female; Medium_Male - Low_Male; Medium_Male - Medium_Female	A_23_P356616	NM_145804	NM_145804

183	XLOC_007052		3.5039	0.0084721	2.072	0.23113	High_Female - High_Male; High_Female - Low_Female; Low_Male - High_Female; Medium_Female - High_Female; High_Female - Medium_Male; Low_Female - High_Male; Low_Male - High_Male; Medium_Female - High_Male; High_Male - Medium_Male; Low_Male - Low_Female; Medium_Female - Low_Female; Low_Female - Medium_Male; Medium_Female - Low_Male; Low_Male - Medium_Male; Medium_Female - Medium_Male	A_19_P00316401		BX500531
184	RAB44	RAB44, member RAS oncogene family	3.4992	0.0085356	2.0688	0.23113	High_Male - High_Female; Low_Female - High_Female; Low_Male - High_Female; Medium_Female - High_Female; Medium_Male - High_Female; High_Male - Low_Female; High_Male - Low_Male; High_Male - Medium_Female; Medium_Male - High_Male; Low_Male - Low_Female; Medium_Female - Low_Female; Medium_Male - Low_Female; Low_Male - Medium_Female; Medium_Male - Low_Male; Medium_Male - Medium_Female	A_21_P0013023		AK125083

185			3.4978	0.0085536	2.0678	0.23113	High_Male - High_Female; Low_Female - High_Female; Low_Male - High_Female; Medium_Female - High_Female; Medium_Male - High_Female; Low_Female - High_Male; Low_Male - High_Male; High_Male - Medium_Female; High_Male - Medium_Male; Low_Female - Low_Male; Low_Female - Medium_Female; Low_Female - Medium_Male; Low_Male - Medium_Female; Low_Male - Medium_Male; Medium_Female - Medium_Male	A_33_P3691615		AK094945
186	LOC286058	uncharacterized LOC286058	3.4883	0.0086848	2.0612	0.23217	High_Male - High_Female; Low_Female - High_Female; Low_Male - High_Female; Medium_Female - High_Female; Medium_Male - High_Female; High_Male - Low_Female; Low_Male - High_Male; High_Male - Medium_Female; High_Male - Medium_Male; Low_Male - Low_Female; Low_Female - Medium_Female; Low_Female - Medium_Male; Low_Male - Medium_Female; Low_Male - Medium_Male; Medium_Male - Medium_Female	A_33_P3630780		AL833160

187	FCRL2	Fc receptor-like 2	3.4872	0.0086997	2.0605	0.23217	High_Female - High_Male; Low_Female - High_Female; Low_Male - High_Female; Medium_Female - High_Female; Medium_Male - High_Female; Low_Female - High_Male; Low_Male - High_Male; Medium_Female - High_Male; Medium_Male - High_Male; Low_Male - Low_Female; Low_Female - Medium_Female; Medium_Male - Low_Female; Low_Male - Medium_Female; Low_Male - Medium_Male; Medium_Male - Medium_Female	A_24_P319647	NM_030764	NM_030764
188	CCDC144A	coiled-coil domain containing 144A	3.4785	0.0088207	2.0545	0.23217	High_Female - High_Male; Low_Female - High_Female; Low_Male - High_Female; Medium_Female - High_Female; Medium_Male - High_Female; Low_Female - High_Male; Low_Male - High_Male; Medium_Female - High_Male; Medium_Male - High_Male; Low_Male - Low_Female; Medium_Female - Low_Female; Medium_Male - Low_Female; Low_Male - Medium_Female; Low_Male - Medium_Male; Medium_Male - Medium_Female	A_21_P0011527	NM_014695	NM_014695

189			3.474	0.0088835	2.0514	0.23217	High_Male - High_Female; Low_Female - High_Female; Low_Male - High_Female; Medium_Female - High_Female; Medium_Male - High_Female; Low_Female - High_Male; High_Male - Low_Male; High_Male - Medium_Female; High_Male - Medium_Male; Low_Female - Low_Male; Low_Female - Medium_Female; Low_Female - Medium_Male; Low_Male - Medium_Female; Low_Male - Medium_Male; Medium_Female - Medium_Male	A_33_P3325102		CR604878
190	EMR4P	egf-like module containing, mucin-like, hormone receptor-like 4 pseudogene	3.473	0.0088974	2.0507	0.23217	High_Male - High_Female; Low_Female - High_Female; Low_Male - High_Female; Medium_Female - High_Female; Medium_Male - High_Female; High_Male - Low_Female; High_Male - Low_Male; High_Male - Medium_Female; Medium_Male - High_Male; Low_Male - Low_Female; Medium_Female - Low_Female; Medium_Male - Low_Female; Low_Male - Medium_Female; Medium_Male - Low_Male; Medium_Male - Medium_Female	A_33_P3247320	NR_024075	NR_024075

191	PGLYRP1	peptidoglycan recognition protein 1	3.4717	0.0089167	2.0498	0.23217	High_Male - High_Female; High_Female - Low_Female; High_Female - Low_Male; High_Female - Medium_Female; Medium_Male - High_Female; High_Male - Low_Female; High_Male - Low_Male; High_Male - Medium_Female; High_Male - Medium_Male; Low_Female - Low_Male; Medium_Female - Low_Female; Medium_Male - Low_Female; Medium_Female - Low_Male; Medium_Male - Low_Male; Medium_Male - Medium_Female	A_23_P208747	NM_005091	NM_005091
192	XLOC_012642		3.4716	0.0089172	2.0498	0.23217	High_Male - High_Female; Low_Female - High_Female; Low_Male - High_Female; Medium_Female - High_Female; Medium_Male - High_Female; High_Male - Low_Female; High_Male - Low_Male; High_Male - Medium_Female; Medium_Male - High_Male; Low_Male - Low_Female; Medium_Female - Low_Female; Medium_Male - Low_Female; Low_Male - Medium_Female; Medium_Male - Low_Male; Medium_Male - Medium_Female	A_21_P0009466		DB516369

193	SCARNA6	small Cajal body-specific RNA 6	3.464	0.009026	2.0445	0.23379	High_Male - High_Female; Low_Female - High_Female; Low_Male - High_Female; Medium_Female - High_Female; High_Female - Medium_Male; Low_Female - High_Male; High_Male - Low_Male; Medium_Female - High_Male; High_Male - Medium_Male; Low_Female - Low_Male; Medium_Female - Low_Female; Low_Female - Medium_Male; Medium_Female - Low_Male; Low_Male - Medium_Male; Medium_Female - Medium_Male	A_33_P3230837	NR_003006	NR_003006
194	ANKRD36BP2	ankyrin repeat domain 36B pseudogene 2	3.4585	0.0091048	2.0407	0.23445	High_Female - High_Male; Low_Female - High_Female; Low_Male - High_Female; Medium_Female - High_Female; High_Female - Medium_Male; Low_Female - High_Male; Low_Male - High_Male; Medium_Female - High_Male; Medium_Male - High_Male; Low_Male - Low_Female; Low_Female - Medium_Female; Low_Female - Medium_Male; Low_Male - Medium_Female; Low_Male - Medium_Male; Medium_Female - Medium_Male	A_24_P341089	NR_015424	NR_015424

195	CCDC64B	coiled-coil domain containing 64B	3.4537	0.0091746	2.0374	0.23445	High_Female - High_Male; High_Female - Low_Female; High_Female - Low_Male; High_Female - Medium_Female; High_Female - Medium_Male; High_Male - Low_Female; High_Male - Low_Male; Medium_Female - High_Male; Medium_Male - High_Male; Low_Male - Low_Female; Medium_Female - Low_Female; Medium_Male - Low_Female; Medium_Female - Low_Male; Medium_Male - Low_Male; Medium_Female - Medium_Male	A_33_P3335590	NM_001103175	NM_001103175
196	GADD45G	growth arrest and DNA-damage-inducible, gamma	3.4525	0.0091922	2.0366	0.23445	High_Male - High_Female; High_Female - Low_Female; High_Female - Low_Male; Medium_Female - High_Female; Medium_Male - High_Female; High_Male - Low_Female; High_Male - Low_Male; High_Male - Medium_Female; High_Male - Medium_Male; Low_Female - Low_Male; Medium_Female - Low_Female; Medium_Male - Low_Female; Medium_Female - Low_Male; Medium_Male - Low_Male; Medium_Female - Medium_Male	A_33_P3252394	NM_006705	NM_006705

197	P2RY14	purinergic receptor P2Y, G-protein coupled, 14	3.445	0.0093028	2.0314	0.23606	High_Male - High_Female; Low_Female - High_Female; Low_Male - High_Female; Medium_Female - High_Female; Medium_Male - High_Female; High_Male - Low_Female; High_Male - Low_Male; High_Male - Medium_Female; High_Male - Medium_Male; Low_Male - Low_Female; Medium_Female - Low_Female; Medium_Male - Low_Female; Medium_Female - Low_Male; Medium_Male - Low_Male; Medium_Male - Medium_Female	A_24_P165864	NM_014879	NM_014879
198			3.4352	0.0094479	2.0247	0.23854	High_Female - High_Male; High_Female - Low_Female; High_Female - Low_Male; High_Female - Medium_Female; High_Female - Medium_Male; Low_Female - High_Male; High_Male - Low_Male; Medium_Female - High_Male; Medium_Male - High_Male; Low_Female - Low_Male; Low_Female - Medium_Female; Medium_Male - Low_Female; Medium_Female - Low_Male; Medium_Male - Low_Male; Medium_Male - Medium_Female	A_33_P3364493		

199	LOC203274	uncharacterized LOC203274	3.429	0.0095415	2.0204	0.23964	High_Female - High_Male; High_Female - Low_Female; High_Female - Low_Male; High_Female - Medium_Female; High_Female - Medium_Male; Low_Female - High_Male; High_Male - Low_Male; Medium_Female - High_Male; Medium_Male - High_Male; Low_Female - Low_Male; Low_Female - Medium_Female; Medium_Male - Low_Female; Medium_Female - Low_Male; Medium_Male - Low_Male; Medium_Male - Medium_Female	A_33_P3474859		BC110369
200	OR6T1	olfactory receptor, family 6, subfamily T, member 1	3.423	0.009633	2.0162	0.23964	High_Male - High_Female; Low_Female - High_Female; High_Female - Low_Male; Medium_Female - High_Female; High_Female - Medium_Male; High_Male - Low_Female; High_Male - Low_Male; High_Male - Medium_Female; High_Male - Medium_Male; Low_Female - Low_Male; Low_Female - Medium_Female; Low_Female - Medium_Male; Medium_Female - Low_Male; Medium_Male - Low_Male; Medium_Female - Medium_Male	A_33_P3342469	NM_001005187	NM_001005187

201	SPRR2A	small proline-rich protein 2A	3.4205	0.0096714	2.0145	0.23964	High_Male - High_Female; Low_Female - High_Female; Low_Male - High_Female; Medium_Female - High_Female; High_Female - Medium_Male; Low_Female - High_Male; High_Male - Low_Male; Medium_Female - High_Male; High_Male - Medium_Male; Low_Female - Low_Male; Medium_Female - Low_Female; Low_Female - Medium_Male; Medium_Female - Low_Male; Low_Male - Medium_Male; Medium_Female - Medium_Male	A_33_P3260426	NM_005988	NM_005988
202	MXI1	MAX interactor 1	3.4197	0.0096833	2.014	0.23964	High_Female - High_Male; Low_Female - High_Female; Low_Male - High_Female; Medium_Female - High_Female; Medium_Male - High_Female; Low_Female - High_Male; Low_Male - High_Male; Medium_Female - High_Male; Medium_Male - High_Male; Low_Male - Low_Female; Medium_Female - Low_Female; Medium_Male - Low_Female; Medium_Female - Low_Male; Medium_Male - Low_Male; Medium_Female - Medium_Male	A_33_P3263902	NM_005962	NM_005962

203	XLOC_l2_014694		3.4087	0.009854	2.0064	0.24243	High_Male - High_Female; Low_Female - High_Female; Low_Male - High_Female; High_Female - Medium_Female; Medium_Male - High_Female; High_Male - Low_Female; Low_Male - High_Male; High_Male - Medium_Female; Medium_Male - High_Male; Low_Male - Low_Female; Low_Female - Medium_Female; Medium_Male - Low_Female; Low_Male - Medium_Female; Medium_Male - Low_Male; Medium_Male - Medium_Female	tc|THC2707131|linc|TCONS_l2_00028604		
204	LOC100506328	uncharacterized LOC100506328	3.405	0.0099123	2.0038	0.24243	High_Female - High_Male; High_Female - Low_Female; High_Female - Low_Male; High_Female - Medium_Female; High_Female - Medium_Male; High_Male - Low_Female; Low_Male - High_Male; High_Male - Medium_Female; Medium_Male - High_Male; Low_Male - Low_Female; Low_Female - Medium_Female; Medium_Male - Low_Female; Low_Male - Medium_Female; Medium_Male - Low_Male; Medium_Male - Medium_Female	A_19_P00317277		AK055877

205	1-Mar	mitochondrial amidoxime reducing component 1	3.4032	0.0099417	2.0025	0.24243	High_Female - High_Male; High_Female - Low_Female; High_Female - Low_Male; High_Female - Medium_Female; High_Female - Medium_Male; High_Male - Low_Female; High_Male - Low_Male; Medium_Female - High_Male; High_Male - Medium_Male; Low_Male - Low_Female; Medium_Female - Low_Female; Medium_Male - Low_Female; Medium_Female - Low_Male; Medium_Male - Low_Male; Medium_Female - Medium_Male	A_33_P3293362	NM_022746	NM_022746
206	FLNA	filamin A, alpha	3.3994	0.010002	1.9999	0.24271	High_Male - High_Female; Low_Female - High_Female; Low_Male - High_Female; Medium_Female - High_Female; Medium_Male - High_Female; Low_Female - High_Male; High_Male - Low_Male; Medium_Female - High_Male; High_Male - Medium_Male; Low_Female - Low_Male; Low_Female - Medium_Female; Low_Female - Medium_Male; Medium_Female - Low_Male; Low_Male - Medium_Male; Medium_Female - Medium_Male	A_33_P3226755		AK125630

207	LRRD1	leucine-rich repeats and death domain containing 1	3.3909	0.010138	1.9941	0.2431	High_Female - High_Male; High_Female - Low_Female; High_Female - Low_Male; Medium_Female - High_Female; High_Female - Medium_Male; Low_Female - High_Male; Low_Male - High_Male; Medium_Female - High_Male; High_Male - Medium_Male; Low_Male - Low_Female; Medium_Female - Low_Female; Low_Female - Medium_Male; Medium_Female - Low_Male; Low_Male - Medium_Male; Medium_Female - Medium_Male	A_33_P3420446	NM_001161528	NM_001161528
208	SAG	S-antigen; retina and pineal gland (arrestin)	3.3885	0.010176	1.9924	0.2431	High_Male - High_Female; Low_Female - High_Female; Low_Male - High_Female; Medium_Female - High_Female; Medium_Male - High_Female; High_Male - Low_Female; High_Male - Low_Male; High_Male - Medium_Female; Medium_Male - High_Male; Low_Male - Low_Female; Medium_Female - Low_Female; Medium_Male - Low_Female; Low_Male - Medium_Female; Medium_Male - Low_Male; Medium_Male - Medium_Female	A_23_P5853	NM_000541	NM_000541

209	TCL1A	T-cell leukemia/lymphoma 1A	3.3868	0.010204	1.9912	0.2431	High_Male - High_Female;<br>Low_Female - High_Female;<br>Low_Male - High_Female;<br>Medium_Female - High_Female;<br>Medium_Male - High_Female;<br>Low_Female - High_Male;<br>Low_Male - High_Male;<br>Medium_Female - High_Male;<br>Medium_Male - High_Male;<br>Low_Male - Low_Female;<br>Medium_Female - Low_Female;<br>Medium_Male - Low_Female;<br>Low_Male - Medium_Female;<br>Medium_Male - Low_Male;<br>Medium_Male - Medium_Female	A_23_P357717	NM_021966	NM_021966
210	HRASLS2	HRAS-like suppressor 2	3.3863	0.010212	1.9909	0.2431	High_Female - High_Male;<br>High_Female - Low_Female;<br>High_Female - Low_Male;<br>High_Female - Medium_Female;<br>High_Female - Medium_Male;<br>High_Male - Low_Female;<br>Low_Male - High_Male;<br>Medium_Female - High_Male;<br>Medium_Male - High_Male;<br>Low_Male - Low_Female;<br>Medium_Female - Low_Female;<br>Medium_Male - Low_Female;<br>Medium_Female - Low_Male;<br>Medium_Male - Low_Male;<br>Medium_Male - Medium_Female	A_23_P105012	NM_017878	NM_017878

211			3.3771	0.010363	1.9845	0.24552	High_Male - High_Female; Low_Female - High_Female; Low_Male - High_Female; Medium_Female - High_Female; Medium_Male - High_Female; Low_Female - High_Male; Low_Male - High_Male; Medium_Female - High_Male; Medium_Male - High_Male; Low_Male - Low_Female; Low_Female - Medium_Female; Medium_Male - Low_Female; Low_Male - Medium_Female; Low_Male - Medium_Male; Medium_Male - Medium_Female	A_33_P3370019		AK093943
212	FAAH	fatty acid amide hydrolase	3.3719	0.010449	1.9809	0.24639	High_Male - High_Female; Low_Female - High_Female; Low_Male - High_Female; Medium_Female - High_Female; Medium_Male - High_Female; Low_Female - High_Male; Low_Male - High_Male; Medium_Female - High_Male; High_Male - Medium_Male; Low_Female - Low_Male; Low_Female - Medium_Female; Low_Female - Medium_Male; Low_Male - Medium_Female; Low_Male - Medium_Male; Medium_Female - Medium_Male	A_33_P3261803	NM_001441	NM_001441

213	XLOC_005939		3.3668	0.010534	1.9774	0.24648	High_Male - High_Female; Low_Female - High_Female; Low_Male - High_Female; Medium_Female - High_Female; Medium_Male - High_Female; Low_Female - High_Male; High_Male - Low_Male; Medium_Female - High_Male; High_Male - Medium_Male; Low_Female - Low_Male; Low_Female - Medium_Female; Low_Female - Medium_Male; Medium_Female - Low_Male; Low_Male - Medium_Male; Medium_Female - Medium_Male	linc|TCONS_00012372|linc|TCONS_00012371|linc|TCONS_00012370		
214	XPNPEP3	X-prolyl aminopeptidase (aminopeptidase P) 3, putative	3.3658	0.010552	1.9767	0.24648	High_Male - High_Female; Low_Female - High_Female; Low_Male - High_Female; Medium_Female - High_Female; Medium_Male - High_Female; Low_Female - High_Male; Low_Male - High_Male; Medium_Female - High_Male; Medium_Male - High_Male; Low_Male - Low_Female; Low_Female - Medium_Female; Medium_Male - Low_Female; Low_Male - Medium_Female; Low_Male - Medium_Male; Medium_Male - Medium_Female	A_21_P0000158	NM_001204827	NM_001204827

215	TMEM146	transmembrane protein 146	3.36	0.01065	1.9727	0.24762	High_Male - High_Female;<br>Low_Female - High_Female;<br>Low_Male - High_Female;<br>Medium_Female - High_Female;<br>Medium_Male - High_Female;<br>Low_Female - High_Male;<br>Low_Male - High_Male;<br>Medium_Female - High_Male;<br>High_Male - Medium_Male;<br>Low_Male - Low_Female;<br>Medium_Female - Low_Female;<br>Low_Female - Medium_Male;<br>Low_Male - Medium_Female;<br>Low_Male - Medium_Male;<br>Medium_Female - Medium_Male	A_33_P3335257	NM_152784	NM_152784
216	LOC145837	uncharacterized LOC145837	3.3548	0.010737	1.9691	0.24849	High_Male - High_Female;<br>Low_Female - High_Female;<br>Low_Male - High_Female;<br>Medium_Female - High_Female;<br>Medium_Male - High_Female;<br>Low_Female - High_Male;<br>Low_Male - High_Male;<br>High_Male - Medium_Female;<br>High_Male - Medium_Male;<br>Low_Female - Low_Male;<br>Low_Female - Medium_Female;<br>Low_Female - Medium_Male;<br>Low_Male - Medium_Female;<br>Low_Male - Medium_Male;<br>Medium_Female - Medium_Male	A_21_P0008647	NR_026979	NR_026979

217	LOC645195	uncharacterized LOC645195	3.3518	0.010789	1.967	0.24856	High_Male - High_Female;<br>Low_Female - High_Female;<br>Low_Male - High_Female;<br>Medium_Female - High_Female;<br>Medium_Male - High_Female;<br>Low_Female - High_Male;<br>Low_Male - High_Male;<br>Medium_Female - High_Male;<br>Medium_Male - High_Male;<br>Low_Male - Low_Female;<br>Low_Female - Medium_Female;<br>Low_Female - Medium_Male;<br>Low_Male - Medium_Female;<br>Low_Male - Medium_Male;<br>Medium_Female - Medium_Male	A_32_P224234		AK123450
218	IL3RA	interleukin 3 receptor, alpha (low affinity)	3.3381	0.011027	1.9576	0.25285	High_Male - High_Female;<br>Low_Female - High_Female;<br>Low_Male - High_Female;<br>Medium_Female - High_Female;<br>Medium_Male - High_Female;<br>High_Male - Low_Female;<br>High_Male - Low_Male;<br>High_Male - Medium_Female;<br>Medium_Male - High_Male;<br>Low_Male - Low_Female;<br>Low_Female - Medium_Female;<br>Medium_Male - Low_Female;<br>Low_Male - Medium_Female;<br>Medium_Male - Low_Male;<br>Medium_Male - Medium_Female	A_32_P217750	NM_002183	NM_002183

219	CLDN5	claudin 5	3.3299	0.011171	1.9519	0.25451	High_Female - High_Male;<br>High_Female - Low_Female;<br>High_Female - Low_Male;<br>Medium_Female - High_Female;<br>High_Female - Medium_Male;<br>Low_Female - High_Male;<br>High_Male - Low_Male;<br>Medium_Female - High_Male;<br>High_Male - Medium_Male;<br>Low_Female - Low_Male;<br>Medium_Female - Low_Female;<br>Low_Female - Medium_Male;<br>Medium_Female - Low_Male;<br>Medium_Male - Low_Male;<br>Medium_Female - Medium_Male	A_33_P3285540	NM_001130861	NM_001130861
220	LOC10013431<br>7	uncharacterized LOC100134317	3.3281	0.011204	1.9506	0.25451	High_Male - High_Female;<br>Low_Female - High_Female;<br>Low_Male - High_Female;<br>Medium_Female - High_Female;<br>Medium_Male - High_Female;<br>Low_Female - High_Male;<br>Low_Male - High_Male;<br>High_Male - Medium_Female;<br>High_Male - Medium_Male;<br>Low_Male - Low_Female;<br>Low_Female - Medium_Female;<br>Low_Female - Medium_Male;<br>Low_Male - Medium_Female;<br>Low_Male - Medium_Male;<br>Medium_Male - Medium_Female	A_21_P0000582	NR_029389	NR_029389

221	ZNHIT2	zinc finger, HIT-type containing 2	3.3231	0.011294	1.9471	0.25451	High_Male - High_Female; Low_Female - High_Female; Low_Male - High_Female; Medium_Female - High_Female; Medium_Male - High_Female; Low_Female - High_Male; Low_Male - High_Male; High_Male - Medium_Female; High_Male - Medium_Male; Low_Male - Low_Female; Low_Female - Medium_Female; Low_Female - Medium_Male; Low_Male - Medium_Female; Low_Male - Medium_Male; Medium_Female - Medium_Male	A_33_P3213747	NM_014205	NM_014205
222	XLOC_007635		3.3226	0.011302	1.9468	0.25451	High_Female - High_Male; Low_Female - High_Female; Low_Male - High_Female; High_Female - Medium_Female; High_Female - Medium_Male; Low_Female - High_Male; Low_Male - High_Male; High_Male - Medium_Female; Medium_Male - High_Male; Low_Male - Low_Female; Low_Female - Medium_Female; Low_Female - Medium_Male; Low_Male - Medium_Female; Low_Male - Medium_Male; Medium_Male - Medium_Female	linc|TCONS_00016263		

223	TGM2	transglutaminase 2 (C polypeptide, protein-glutamine-gamma-glutamyltransferase)	3.3169	0.011406	1.9429	0.25569	High_Female - High_Male; Low_Female - High_Female; Low_Male - High_Female; High_Female - Medium_Female; High_Female - Medium_Male; Low_Female - High_Male; Low_Male - High_Male; Medium_Female - High_Male; High_Male - Medium_Male; Low_Male - Low_Female; Low_Female - Medium_Female; Low_Female - Medium_Male; Low_Male - Medium_Female; Low_Male - Medium_Male; Medium_Female - Medium_Male	A_24_P923251	NM_198951	NM_198951
224	LOC100506328	uncharacterized LOC100506328	3.309	0.011551	1.9374	0.25779	High_Female - High_Male; High_Female - Low_Female; High_Female - Low_Male; High_Female - Medium_Female; High_Female - Medium_Male; High_Male - Low_Female; High_Male - Low_Male; High_Male - Medium_Female; Medium_Male - High_Male; Low_Male - Low_Female; Medium_Female - Low_Female; Medium_Male - Low_Female; Medium_Female - Low_Male; Medium_Male - Low_Male; Medium_Male - Medium_Female	A_19_P00321148		AK055877

225	C1orf190	chromosome 1 open reading frame 190	3.3003	0.011711	1.9314	0.25964	High_Male - High_Female; Low_Female - High_Female; Low_Male - High_Female; Medium_Female - High_Female; High_Female - Medium_Male; Low_Female - High_Male; Low_Male - High_Male; Medium_Female - High_Male; High_Male - Medium_Male; Low_Male - Low_Female; Medium_Female - Low_Female; Low_Female - Medium_Male; Low_Male - Medium_Female; Low_Male - Medium_Male; Medium_Female - Medium_Male	A_33_P3400823	NM_001013615	NM_001013615
226	C12orf51	chromosome 12 open reading frame 51	3.2989	0.011738	1.9304	0.25964	High_Male - High_Female; Low_Female - High_Female; Low_Male - High_Female; Medium_Female - High_Female; Medium_Male - High_Female; Low_Female - High_Male; Low_Male - High_Male; Medium_Female - High_Male; High_Male - Medium_Male; Low_Male - Low_Female; Low_Female - Medium_Female; Low_Female - Medium_Male; Low_Male - Medium_Female; Low_Male - Medium_Male; Medium_Female - Medium_Male	A_23_P309292	NM_001109662	NM_001109662

227	IL5RA	interleukin 5 receptor, alpha	3.2909	0.011888	1.9249	0.2618	High_Male - High_Female; Low_Female - High_Female; Low_Male - High_Female; Medium_Female - High_Female; Medium_Male - High_Female; High_Male - Low_Female; High_Male - Low_Male; High_Male - Medium_Female; High_Male - Medium_Male; Low_Male - Low_Female; Medium_Female - Low_Female; Medium_Male - Low_Female; Low_Male - Medium_Female; Medium_Male - Low_Male; Medium_Male - Medium_Female	A_33_P3328254	NM_175725	NM_175725
228	XLOC_002996		3.2848	0.012005	1.9206	0.26198	High_Male - High_Female; Low_Female - High_Female; Low_Male - High_Female; Medium_Female - High_Female; Medium_Male - High_Female; Low_Female - High_Male; Low_Male - High_Male; Medium_Female - High_Male; High_Male - Medium_Male; Low_Male - Low_Female; Medium_Female - Low_Female; Low_Female - Medium_Male; Low_Male - Medium_Female; Low_Male - Medium_Male; Medium_Female - Medium_Male	linc|TCONS_00006377		

229	ATCAY	ataxia, cerebellar, Cayman type	3.2845	0.012011	1.9204	0.26198	High_Male - High_Female; Low_Female - High_Female; Low_Male - High_Female; Medium_Female - High_Female; High_Female - Medium_Male; Low_Female - High_Male; Low_Male - High_Male; Medium_Female - High_Male; High_Male - Medium_Male; Low_Male - Low_Female; Medium_Female - Low_Female; Low_Female - Medium_Male; Medium_Female - Low_Male; Low_Male - Medium_Male; Medium_Female - Medium_Male	A_24_P40094	NM_033064	NM_033064
230	CRISPLD2	cysteine-rich secretory protein LCCL domain containing 2	3.281	0.012078	1.918	0.26198	High_Female - High_Male; High_Female - Low_Female; High_Female - Low_Male; High_Female - Medium_Female; High_Female - Medium_Male; High_Male - Low_Female; High_Male - Low_Male; High_Male - Medium_Female; High_Male - Medium_Male; Low_Female - Low_Male; Medium_Female - Low_Female; Medium_Male - Low_Female; Medium_Female - Low_Male; Medium_Male - Low_Male; Medium_Female - Medium_Male	A_23_P106602	NM_031476	NM_031476

231			3.2771	0.012154	1.9153	0.26198	High_Female - High_Male; High_Female - Low_Female; Low_Male - High_Female; Medium_Female - High_Female; High_Female - Medium_Male; Low_Female - High_Male; Low_Male - High_Male; Medium_Female - High_Male; Medium_Male - High_Male; Low_Male - Low_Female; Medium_Female - Low_Female; Low_Female - Medium_Male; Medium_Female - Low_Male; Low_Male - Medium_Male; Medium_Female - Medium_Male	A_33_P3382887		
232	GPR141	G protein-coupled receptor 141	3.2768	0.012158	1.9151	0.26198	High_Female - High_Male; High_Female - Low_Female; High_Female - Low_Male; High_Female - Medium_Female; High_Female - Medium_Male; Low_Female - High_Male; High_Male - Low_Male; Medium_Female - High_Male; Medium_Male - High_Male; Low_Female - Low_Male; Medium_Female - Low_Female; Medium_Male - Low_Female; Medium_Female - Low_Male; Medium_Male - Low_Male; Medium_Male - Medium_Female	A_24_P334718	NM_181791	NM_181791

233	HCG18	HLA complex group 18 (non-protein coding)	3.2722	0.01225	1.9119	0.26281	High_Male - High_Female; Low_Female - High_Female; Low_Male - High_Female; Medium_Female - High_Female; Medium_Male - High_Female; Low_Female - High_Male; Low_Male - High_Male; Medium_Female - High_Male; High_Male - Medium_Male; Low_Male - Low_Female; Low_Female - Medium_Female; Low_Female - Medium_Male; Low_Male - Medium_Female; Low_Male - Medium_Male; Medium_Female - Medium_Male	A_19_P00321473	NR_024052	NR_024052
234	RGS14	regulator of G-protein signaling 14	3.2666	0.012359	1.908	0.26396	High_Female - High_Male; High_Female - Low_Female; High_Female - Low_Male; High_Female - Medium_Female; High_Female - Medium_Male; High_Male - Low_Female; High_Male - Low_Male; Medium_Female - High_Male; Medium_Male - High_Male; Low_Female - Low_Male; Medium_Female - Low_Female; Medium_Male - Low_Female; Medium_Female - Low_Male; Medium_Male - Low_Male; Medium_Male - Medium_Female	A_23_P124927	NM_006480	NM_006480

235	BMS1P1	BMS1 pseudogene 1	3.2638	0.012414	1.9061	0.26396	High_Female - High_Male; Low_Female - High_Female; Low_Male - High_Female; Medium_Female - High_Female; Medium_Male - High_Female; Low_Female - High_Male; Low_Male - High_Male; Medium_Female - High_Male; Medium_Male - High_Male; Low_Male - Low_Female; Low_Female - Medium_Female; Medium_Male - Low_Female; Low_Male - Medium_Female; Low_Male - Medium_Male; Medium_Male - Medium_Female	A_21_P0010879	NR_026566	NR_026566
236	LRRC14	leucine rich repeat containing 14	3.2574	0.012542	1.9016	0.26396	High_Male - High_Female; Low_Female - High_Female; Low_Male - High_Female; Medium_Female - High_Female; Medium_Male - High_Female; Low_Female - High_Male; Low_Male - High_Male; Medium_Female - High_Male; Medium_Male - High_Male; Low_Male - Low_Female; Low_Female - Medium_Female; Medium_Male - Low_Female; Low_Male - Medium_Female; Low_Male - Medium_Male; Medium_Male - Medium_Female	A_24_P363745	NM_014665	NM_014665

237			3.2569	0.012551	1.9013	0.26396	High_Male - High_Female; Low_Female - High_Female; Low_Male - High_Female; Medium_Female - High_Female; Medium_Male - High_Female; Low_Female - High_Male; High_Male - Low_Male; High_Male - Medium_Female; High_Male - Medium_Male; Low_Female - Low_Male; Low_Female - Medium_Female; Low_Female - Medium_Male; Medium_Female - Low_Male; Low_Male - Medium_Male; Medium_Female - Medium_Male	ens|ENST00000552290		
238	NEURL	neuralized homolog (Drosophila)	3.2538	0.012615	1.8991	0.26396	High_Female - High_Male; High_Female - Low_Female; Low_Male - High_Female; Medium_Female - High_Female; High_Female - Medium_Male; Low_Female - High_Male; Low_Male - High_Male; Medium_Female - High_Male; High_Male - Medium_Male; Low_Male - Low_Female; Medium_Female - Low_Female; Low_Female - Medium_Male; Medium_Female - Low_Male; Low_Male - Medium_Male; Medium_Female - Medium_Male	A_33_P3293524	NM_004210	NM_004210

239			3.2483	0.012725	1.8953	0.26396	High_Male - High_Female;<br>Low_Female - High_Female;<br>Low_Male - High_Female;<br>Medium_Female - High_Female;<br>Medium_Male - High_Female;<br>Low_Female - High_Male;<br>Low_Male - High_Male;<br>Medium_Female - High_Male;<br>High_Male - Medium_Male;<br>Low_Male - Low_Female;<br>Medium_Female - Low_Female;<br>Low_Female - Medium_Male;<br>Low_Male - Medium_Female;<br>Low_Male - Medium_Male;<br>Medium_Female - Medium_Male	A_33_P3339202		
240	HILPDA	hypoxia inducible lipid droplet-associated	3.2472	0.012747	1.8946	0.26396	High_Male - High_Female;<br>High_Female - Low_Female;<br>High_Female - Low_Male;<br>Medium_Female - High_Female;<br>Medium_Male - High_Female;<br>High_Male - Low_Female;<br>High_Male - Low_Male;<br>High_Male - Medium_Female;<br>High_Male - Medium_Male;<br>Low_Male - Low_Female;<br>Medium_Female - Low_Female;<br>Medium_Male - Low_Female;<br>Medium_Female - Low_Male;<br>Medium_Male - Low_Male;<br>Medium_Male - Medium_Female	A_23_P20022	NM_013332	NM_013332

241	XLOC_l2_007424		3.2441	0.01281	1.8924	0.26396	High_Female - High_Male; High_Female - Low_Female; High_Female - Low_Male; High_Female - Medium_Female; High_Female - Medium_Male; Low_Female - High_Male; High_Male - Low_Male; Medium_Female - High_Male; Medium_Male - High_Male; Low_Female - Low_Male; Medium_Female - Low_Female; Low_Female - Medium_Male; Medium_Female - Low_Male; Medium_Male - Low_Male; Medium_Female - Medium_Male	A_21_P0011814		CB178477
242	ARHGEF4	Rho guanine nucleotide exchange factor (GEF) 4	3.242	0.012853	1.891	0.26396	High_Male - High_Female; Low_Female - High_Female; Low_Male - High_Female; Medium_Female - High_Female; Medium_Male - High_Female; Low_Female - High_Male; Low_Male - High_Male; Medium_Female - High_Male; Medium_Male - High_Male; Low_Male - Low_Female; Low_Female - Medium_Female; Low_Female - Medium_Male; Low_Male - Medium_Female; Low_Male - Medium_Male; Medium_Male - Medium_Female	A_23_P253221	NM_032995	NM_032995

243	XLOC_002975		3.2405	0.012884	1.89	0.26396	High_Male - High_Female; Low_Female - High_Female; Low_Male - High_Female; Medium_Female - High_Female; Medium_Male - High_Female; Low_Female - High_Male; High_Male - Low_Male; Medium_Female - High_Male; High_Male - Medium_Male; Low_Female - Low_Male; Low_Female - Medium_Female; Low_Female - Medium_Male; Medium_Female - Low_Male; Medium_Male - Low_Male; Medium_Female - Medium_Male	linc|TCONS_00006356		
244	XLOC_l2_013931		3.2391	0.012913	1.889	0.26396	High_Female - High_Male; Low_Female - High_Female; Low_Male - High_Female; Medium_Female - High_Female; Medium_Male - High_Female; Low_Female - High_Male; Low_Male - High_Male; Medium_Female - High_Male; Medium_Male - High_Male; Low_Male - Low_Female; Low_Female - Medium_Female; Medium_Male - Low_Female; Low_Male - Medium_Female; Low_Male - Medium_Male; Medium_Male - Medium_Female	tc|THC2539716|tc|THC2567033|linc|TCONS_l2_00026784		

245	IGFBP2	insulin-like growth factor binding protein 2, 36kDa	3.2358	0.012983	1.8866	0.26396	High_Female - High_Male;<br>High_Female - Low_Female;<br>High_Female - Low_Male;<br>High_Female - Medium_Female;<br>High_Female - Medium_Male;<br>Low_Female - High_Male;<br>High_Male - Low_Male;<br>Medium_Female - High_Male;<br>High_Male - Medium_Male;<br>Low_Female - Low_Male;<br>Low_Female - Medium_Female;<br>Low_Female - Medium_Male;<br>Medium_Female - Low_Male;<br>Low_Male - Medium_Male;<br>Medium_Female - Medium_Male	A_23_P119943	NM_000597	NM_000597
246	PRSS36	protease, serine, 36	3.2354	0.01299	1.8864	0.26396	High_Female - High_Male;<br>High_Female - Low_Female;<br>Low_Male - High_Female;<br>Medium_Female - High_Female;<br>High_Female - Medium_Male;<br>Low_Female - High_Male;<br>Low_Male - High_Male;<br>Medium_Female - High_Male;<br>High_Male - Medium_Male;<br>Low_Male - Low_Female;<br>Medium_Female - Low_Female;<br>Low_Female - Medium_Male;<br>Medium_Female - Low_Male;<br>Low_Male - Medium_Male;<br>Medium_Female - Medium_Male	A_24_P412734	NM_173502	NM_173502

247	MXD3	MAX dimerization protein 3	3.2289	0.013125	1.8819	0.26487	High_Female - High_Male; High_Female - Low_Female; High_Female - Low_Male; High_Female - Medium_Female; High_Female - Medium_Male; High_Male - Low_Female; High_Male - Low_Male; High_Male - Medium_Female; High_Male - Medium_Male; Low_Female - Low_Male; Medium_Female - Low_Female; Medium_Male - Low_Female; Medium_Female - Low_Male; Medium_Male - Low_Male; Medium_Female - Medium_Male	A_24_P417706	NM_001142935	NM_001142935
248			3.2282	0.01314	1.8814	0.26487	High_Female - High_Male; High_Female - Low_Female; High_Female - Low_Male; Medium_Female - High_Female; High_Female - Medium_Male; Low_Female - High_Male; Low_Male - High_Male; Medium_Female - High_Male; High_Male - Medium_Male; Low_Male - Low_Female; Medium_Female - Low_Female; Low_Female - Medium_Male; Medium_Female - Low_Male; Low_Male - Medium_Male; Medium_Female - Medium_Male	A_33_P3363305		

249	GFRA4	GDNF family receptor alpha 4	3.2156	0.013408	1.8726	0.26918	High_Male - High_Female;<br>Low_Female - High_Female;<br>Low_Male - High_Female;<br>Medium_Female - High_Female;<br>Medium_Male - High_Female;<br>High_Male - Low_Female;<br>High_Male - Low_Male;<br>High_Male - Medium_Female;<br>High_Male - Medium_Male;<br>Low_Female - Low_Male;<br>Low_Female - Medium_Female;<br>Low_Female - Medium_Male;<br>Medium_Female - Low_Male;<br>Low_Male - Medium_Male;<br>Medium_Female - Medium_Male	A_33_P3317589	NM_145762	NM_145762
250	C17orf103	chromosome 17 open reading frame 103	3.209	0.013551	1.868	0.27096	High_Male - High_Female;<br>Low_Female - High_Female;<br>Low_Male - High_Female;<br>Medium_Female - High_Female;<br>Medium_Male - High_Female;<br>Low_Female - High_Male;<br>Low_Male - High_Male;<br>Medium_Female - High_Male;<br>High_Male - Medium_Male;<br>Low_Male - Low_Female;<br>Low_Female - Medium_Female;<br>Low_Female - Medium_Male;<br>Low_Male - Medium_Female;<br>Low_Male - Medium_Male;<br>Medium_Female - Medium_Male	A_33_P3334015	NM_152914	NM_152914

251			3.1993	0.013761	1.8613	0.27407	High_Male - High_Female; Low_Female - High_Female; Low_Male - High_Female; Medium_Female - High_Female; Medium_Male - High_Female; High_Male - Low_Female; High_Male - Low_Male; High_Male - Medium_Female; High_Male - Medium_Male; Low_Male - Low_Female; Medium_Female - Low_Female; Low_Female - Medium_Male; Low_Male - Medium_Female; Low_Male - Medium_Male; Medium_Female - Medium_Male	A_33_P3335401		
252	CCDC144A	coiled-coil domain containing 144A	3.1924	0.013915	1.8565	0.27413	High_Male - High_Female; Low_Female - High_Female; Low_Male - High_Female; Medium_Female - High_Female; Medium_Male - High_Female; Low_Female - High_Male; Low_Male - High_Male; Medium_Female - High_Male; Medium_Male - High_Male; Low_Male - Low_Female; Low_Female - Medium_Female; Low_Female - Medium_Male; Low_Male - Medium_Female; Low_Male - Medium_Male; Medium_Male - Medium_Female	A_33_P3390580	NM_014695	NM_014695

253	TAOK2	TAO kinase 2	3.1904	0.013959	1.8551	0.27413	High_Male - High_Female;<br>Low_Female - High_Female;<br>Low_Male - High_Female;<br>Medium_Female - High_Female;<br>High_Female - Medium_Male;<br>Low_Female - High_Male;<br>Low_Male - High_Male;<br>High_Male - Medium_Female;<br>High_Male - Medium_Male;<br>Low_Female - Low_Male;<br>Low_Female - Medium_Female;<br>Low_Female - Medium_Male;<br>Low_Male - Medium_Female;<br>Low_Male - Medium_Male;<br>Medium_Female - Medium_Male	A_33_P3346098	NM_016151	NM_016151
254	UBE2O	ubiquitin-conjugating enzyme E2O	3.1895	0.013979	1.8545	0.27413	High_Female - High_Male;<br>Low_Female - High_Female;<br>Low_Male - High_Female;<br>Medium_Female - High_Female;<br>High_Female - Medium_Male;<br>Low_Female - High_Male;<br>Low_Male - High_Male;<br>Medium_Female - High_Male;<br>Medium_Male - High_Male;<br>Low_Female - Low_Male;<br>Medium_Female - Low_Female;<br>Low_Female - Medium_Male;<br>Medium_Female - Low_Male;<br>Low_Male - Medium_Male;<br>Medium_Female - Medium_Male	A_24_P172993	NM_022066	NM_022066

255	SERPINA1	serpin peptidase inhibitor, clade A (alpha-1 antiproteinase, antitrypsin), member 1	3.1893	0.013984	1.8544	0.27413	High_Male - High_Female; Low_Female - High_Female; Low_Male - High_Female; Medium_Female - High_Female; Medium_Male - High_Female; High_Male - Low_Female; Low_Male - High_Male; High_Male - Medium_Female; Medium_Male - High_Male; Low_Male - Low_Female; Low_Female - Medium_Female; Medium_Male - Low_Female; Low_Male - Medium_Female; Low_Male - Medium_Male; Medium_Male - Medium_Female	A_33_P3289659		BX248257
256	DSP	desmoplakin	3.1828	0.01413	1.8498	0.27593	High_Female - High_Male; Low_Female - High_Female; Low_Male - High_Female; Medium_Female - High_Female; Medium_Male - High_Female; Low_Female - High_Male; Low_Male - High_Male; Medium_Female - High_Male; Medium_Male - High_Male; Low_Male - Low_Female; Medium_Female - Low_Female; Medium_Male - Low_Female; Medium_Female - Low_Male; Medium_Male - Low_Male; Medium_Male - Medium_Female	A_33_P3402565	NM_004415	NM_004415

257	BNIP3L	BCL2/adenovirus E1B 19kDa interacting protein 3-like	3.1785	0.014227	1.8469	0.27642	High_Female - High_Male; Low_Female - High_Female; Low_Male - High_Female; Medium_Female - High_Female; High_Female - Medium_Male; Low_Female - High_Male; Low_Male - High_Male; Medium_Female - High_Male; Medium_Male - High_Male; Low_Male - Low_Female; Low_Female - Medium_Female; Low_Female - Medium_Male; Low_Male - Medium_Female; Low_Male - Medium_Male; Medium_Female - Medium_Male	A_33_P3396951	NM_004331	NM_004331
258	FLJ39051	uncharacterized LOC399972	3.1749	0.014309	1.8444	0.27642	High_Female - High_Male; High_Female - Low_Female; High_Female - Low_Male; High_Female - Medium_Female; High_Female - Medium_Male; Low_Female - High_Male; High_Male - Low_Male; Medium_Female - High_Male; Medium_Male - High_Male; Low_Female - Low_Male; Medium_Female - Low_Female; Medium_Male - Low_Female; Medium_Female - Low_Male; Medium_Male - Low_Male; Medium_Female - Medium_Male	A_21_P0000607	NR_033839	NR_033839

259	ARGFX	arginine-fifty homeobox	3.1744	0.014321	1.844	0.27642	High_Male - High_Female; Low_Female - High_Female; Low_Male - High_Female; Medium_Female - High_Female; High_Female - Medium_Male; Low_Female - High_Male; High_Male - Low_Male; High_Male - Medium_Female; High_Male - Medium_Male; Low_Female - Low_Male; Low_Female - Medium_Female; Low_Female - Medium_Male; Low_Male - Medium_Female; Low_Male - Medium_Male; Medium_Female - Medium_Male	A_33_P3209706	NM_001012659	NM_001012659
260	CHRM4	cholinergic receptor, muscarinic 4	3.1689	0.014448	1.8402	0.27776	High_Male - High_Female; High_Female - Low_Female; Low_Male - High_Female; Medium_Female - High_Female; Medium_Male - High_Female; High_Male - Low_Female; High_Male - Low_Male; High_Male - Medium_Female; High_Male - Medium_Male; Low_Male - Low_Female; Medium_Female - Low_Female; Medium_Male - Low_Female; Medium_Female - Low_Male; Medium_Male - Low_Male; Medium_Female - Medium_Male	A_33_P3367850	NM_000741	NM_000741

261	C2orf63	chromosome 2 open reading frame 63	3.1665	0.014502	1.8386	0.27776	High_Female - High_Male; Low_Female - High_Female; Low_Male - High_Female; High_Female - Medium_Female; Medium_Male - High_Female; Low_Female - High_Male; Low_Male - High_Male; High_Male - Medium_Female; Medium_Male - High_Male; Low_Female - Low_Male; Low_Female - Medium_Female; Low_Female - Medium_Male; Low_Male - Medium_Female; Low_Male - Medium_Male; Medium_Male - Medium_Female	A_23_P257417	NM_152385	NM_152385
262	OR1S2	olfactory receptor, family 1, subfamily S, member 2	3.1539	0.014799	1.8298	0.2808	High_Male - High_Female; Low_Female - High_Female; Low_Male - High_Female; Medium_Female - High_Female; Medium_Male - High_Female; Low_Female - High_Male; High_Male - Low_Male; High_Male - Medium_Female; High_Male - Medium_Male; Low_Female - Low_Male; Low_Female - Medium_Female; Low_Female - Medium_Male; Medium_Female - Low_Male; Low_Male - Medium_Male; Medium_Female - Medium_Male	A_23_P13294	NM_001004459	NM_001004459

263	XLOC_001595		3.153	0.01482	1.8292	0.2808	High_Female - High_Male;<br>High_Female - Low_Female;<br>High_Female - Low_Male;<br>High_Female - Medium_Female;<br>High_Female - Medium_Male;<br>High_Male - Low_Female;<br>Low_Male - High_Male;<br>Medium_Female - High_Male;<br>Medium_Male - High_Male;<br>Low_Male - Low_Female;<br>Medium_Female - Low_Female;<br>Medium_Male - Low_Female;<br>Medium_Female - Low_Male;<br>Medium_Male - Low_Male;<br>Medium_Male - Medium_Female	linc|TCONS_00003800		
264	PLAC8	placenta-specific 8	3.1526	0.014829	1.8289	0.2808	High_Female - High_Male;<br>Low_Female - High_Female;<br>Low_Male - High_Female;<br>Medium_Female - High_Female;<br>Medium_Male - High_Female;<br>Low_Female - High_Male;<br>Low_Male - High_Male;<br>Medium_Female - High_Male;<br>Medium_Male - High_Male;<br>Low_Female - Low_Male;<br>Low_Female - Medium_Female;<br>Medium_Male - Low_Female;<br>Low_Male - Medium_Female;<br>Medium_Male - Low_Male;<br>Medium_Male - Medium_Female	A_33_P3241989	NM_001130715	NM_001130715

265	XLOC_004828		3.1289	0.015402	1.8124	0.28946	High_Male - High_Female; Low_Female - High_Female; Low_Male - High_Female; Medium_Female - High_Female; Medium_Male - High_Female; Low_Female - High_Male; Low_Male - High_Male; High_Male - Medium_Female; High_Male - Medium_Male; Low_Female - Low_Male; Low_Female - Medium_Female; Low_Female - Medium_Male; Low_Male - Medium_Female; Low_Male - Medium_Male; Medium_Female - Medium_Male	ens|ENST00000504349|linc|TCONS_00009667		
266	ANKRD20A5P	ankyrin repeat domain 20 family, member A5, pseudogene	3.1256	0.015483	1.8101	0.28946	High_Female - High_Male; High_Female - Low_Female; High_Female - Low_Male; High_Female - Medium_Female; High_Female - Medium_Male; High_Male - Low_Female; Low_Male - High_Male; High_Male - Medium_Female; High_Male - Medium_Male; Low_Male - Low_Female; Low_Female - Medium_Female; Medium_Male - Low_Female; Low_Male - Medium_Female; Low_Male - Medium_Male; Medium_Male - Medium_Female	A_32_P68942	NR_040113	NR_040113

267	XLOC_007868		3.123	0.015547	1.8083	0.28946	High_Male - High_Female; Low_Female - High_Female; Low_Male - High_Female; Medium_Female - High_Female; Medium_Male - High_Female; Low_Female - High_Male; High_Male - Low_Male; Medium_Female - High_Male; High_Male - Medium_Male; Low_Female - Low_Male; Medium_Female - Low_Female; Low_Female - Medium_Male; Medium_Female - Low_Male; Low_Male - Medium_Male; Medium_Female - Medium_Male	linc|TCONS_00016491		
268	LOC100652883	arachidonate 15-lipoxygenase-like	3.1209	0.015601	1.8068	0.28946	High_Male - High_Female; High_Female - Low_Female; Low_Male - High_Female; Medium_Female - High_Female; Medium_Male - High_Female; High_Male - Low_Female; Low_Male - High_Male; High_Male - Medium_Female; Medium_Male - High_Male; Low_Male - Low_Female; Medium_Female - Low_Female; Medium_Male - Low_Female; Low_Male - Medium_Female; Medium_Male - Low_Male; Medium_Male - Medium_Female	A_21_P0014014	XM_003403469	XM_003403469

269	HS3ST1	heparan sulfate (glucosamine) 3-O-sulfotransferase 1	3.1205	0.01561	1.8066	0.28946	High_Female - High_Male; Low_Female - High_Female; Low_Male - High_Female; Medium_Female - High_Female; Medium_Male - High_Female; Low_Female - High_Male; Low_Male - High_Male; Medium_Female - High_Male; Medium_Male - High_Male; Low_Male - Low_Female; Medium_Female - Low_Female; Medium_Male - Low_Female; Low_Male - Medium_Female; Medium_Male - Low_Male; Medium_Male - Medium_Female	A_23_P121657	NM_005114	NM_005114
270	XLOC_002125		3.1196	0.015634	1.8059	0.28946	High_Male - High_Female; Low_Female - High_Female; Low_Male - High_Female; Medium_Female - High_Female; Medium_Male - High_Female; Low_Female - High_Male; Low_Male - High_Male; High_Male - Medium_Female; Medium_Male - High_Male; Low_Female - Low_Male; Low_Female - Medium_Female; Low_Female - Medium_Male; Low_Male - Medium_Female; Low_Male - Medium_Male; Medium_Male - Medium_Female	A_21_P0002057		AL355732

271	LOC100129216	beta-defensin 131-like	3.1093	0.015893	1.7988	0.29317	High_Male - High_Female; Low_Female - High_Female; Low_Male - High_Female; Medium_Female - High_Female; High_Female - Medium_Male; Low_Female - High_Male; High_Male - Low_Male; Medium_Female - High_Male; High_Male - Medium_Male; Low_Female - Low_Male; Medium_Female - Low_Female; Low_Female - Medium_Male; Medium_Female - Low_Male; Low_Male - Medium_Male; Medium_Female - Medium_Male	A_33_P3255531	NM_001242853	NM_001242853
272	FLOT2	flotillin 2	3.1019	0.016083	1.7936	0.29513	High_Female - High_Male; High_Female - Low_Female; High_Female - Low_Male; High_Female - Medium_Female; High_Female - Medium_Male; High_Male - Low_Female; High_Male - Low_Male; Medium_Female - High_Male; Medium_Male - High_Male; Low_Female - Low_Male; Medium_Female - Low_Female; Medium_Male - Low_Female; Medium_Female - Low_Male; Medium_Male - Low_Male; Medium_Female - Medium_Male	A_24_P253818	NM_004475	NM_004475

273	C1orf140	uncharacterized LOC400804	3.1006	0.016118	1.7927	0.29513	High_Female - High_Male;<br>High_Female - Low_Female;<br>High_Female - Low_Male;<br>High_Female - Medium_Female;<br>High_Female - Medium_Male;<br>High_Male - Low_Female;<br>High_Male - Low_Male;<br>Medium_Female - High_Male;<br>Medium_Male - High_Male;<br>Low_Female - Low_Male;<br>Medium_Female - Low_Female;<br>Medium_Male - Low_Female;<br>Medium_Female - Low_Male;<br>Medium_Male - Low_Male;<br>Medium_Male - Medium_Female	A_33_P3295091	NR_024236	NR_024236
274	DDHD2	DDHD domain containing 2	3.0955	0.016249	1.7892	0.29646	High_Female - High_Male;<br>Low_Female - High_Female;<br>Low_Male - High_Female;<br>Medium_Female - High_Female;<br>Medium_Male - High_Female;<br>Low_Female - High_Male;<br>Low_Male - High_Male;<br>Medium_Female - High_Male;<br>Medium_Male - High_Male;<br>Low_Male - Low_Female;<br>Low_Female - Medium_Female;<br>Medium_Male - Low_Female;<br>Low_Male - Medium_Female;<br>Low_Male - Medium_Male;<br>Medium_Male - Medium_Female	A_21_P0000039	NM_001164234	NM_001164234

275	PNMA6C	paraneoplastic antigen like 6C	3.0918	0.016344	1.7866	0.29711	High_Male - High_Female; High_Female - Low_Female; Low_Male - High_Female; High_Female - Medium_Female; Medium_Male - High_Female; High_Male - Low_Female; High_Male - Low_Male; High_Male - Medium_Female; Medium_Male - High_Male; Low_Male - Low_Female; Low_Female - Medium_Female; Medium_Male - Low_Female; Low_Male - Medium_Female; Medium_Male - Low_Male; Medium_Male - Medium_Female	A_33_P3233436	NM_001170944	NM_001170944
276	XLOC_007219		3.0824	0.016593	1.7801	0.3002	High_Female - High_Male; High_Female - Low_Female; High_Female - Low_Male; High_Female - Medium_Female; High_Female - Medium_Male; Low_Female - High_Male; Low_Male - High_Male; Medium_Female - High_Male; High_Male - Medium_Male; Low_Female - Low_Male; Low_Female - Medium_Female; Low_Female - Medium_Male; Low_Male - Medium_Female; Low_Male - Medium_Male; Medium_Female - Medium_Male	A_21_P0005631		BC014119

277	LOC100131820	uncharacterized LOC100131820	3.0804	0.016647	1.7787	0.3002	High_Female - High_Male; Low_Female - High_Female; Low_Male - High_Female; High_Female - Medium_Female; Medium_Male - High_Female; Low_Female - High_Male; Low_Male - High_Male; Medium_Female - High_Male; Medium_Male - High_Male; Low_Female - Low_Male; Low_Female - Medium_Female; Low_Female - Medium_Male; Low_Male - Medium_Female; Medium_Male - Low_Male; Medium_Male - Medium_Female	A_33_P3275630		AK125613
278	XLOC_011570		3.0786	0.016695	1.7774	0.3002	High_Male - High_Female; Low_Female - High_Female; Low_Male - High_Female; Medium_Female - High_Female; Medium_Male - High_Female; Low_Female - High_Male; Low_Male - High_Male; Medium_Female - High_Male; High_Male - Medium_Male; Low_Female - Low_Male; Low_Female - Medium_Female; Low_Female - Medium_Male; Low_Male - Medium_Female; Low_Male - Medium_Male; Medium_Female - Medium_Male	linc|TCONS_00023775		

279			3.073	0.016845	1.7735	0.3018	High_Male - High_Female; Low_Female - High_Female; Low_Male - High_Female; Medium_Female - High_Female; Medium_Male - High_Female; High_Male - Low_Female; Low_Male - High_Male; Medium_Female - High_Male; High_Male - Medium_Male; Low_Male - Low_Female; Medium_Female - Low_Female; Low_Female - Medium_Male; Low_Male - Medium_Female; Low_Male - Medium_Male; Medium_Female - Medium_Male	A_33_P3378354		CR615161
280	ANKRD36	ankyrin repeat domain 36	3.0708	0.016904	1.772	0.3018	High_Female - High_Male; High_Female - Low_Female; Low_Male - High_Female; High_Female - Medium_Female; High_Female - Medium_Male; Low_Female - High_Male; Low_Male - High_Male; Medium_Female - High_Male; Medium_Male - High_Male; Low_Male - Low_Female; Medium_Female - Low_Female; Low_Female - Medium_Male; Low_Male - Medium_Female; Low_Male - Medium_Male; Medium_Female - Medium_Male	A_21_P0011812	NM_001164315	NM_001164315

281	EPN2	epsin 2	3.0647	0.017071	1.7677	0.3037	High_Male - High_Female;<br>Low_Female - High_Female;<br>Low_Male - High_Female;<br>Medium_Female - High_Female;<br>Medium_Male - High_Female;<br>High_Male - Low_Female;<br>High_Male - Low_Male;<br>High_Male - Medium_Female;<br>Medium_Male - High_Male;<br>Low_Male - Low_Female;<br>Medium_Female - Low_Female;<br>Medium_Male - Low_Female;<br>Low_Male - Medium_Female;<br>Medium_Male - Low_Male;<br>Medium_Male - Medium_Female	A_23_P89310	NM_014964	NM_014964
282	XLOC_009790		3.0598	0.017207	1.7643	0.30502	High_Male - High_Female;<br>Low_Female - High_Female;<br>High_Female - Low_Male;<br>Medium_Female - High_Female;<br>High_Female - Medium_Male;<br>Low_Female - High_Male;<br>High_Male - Low_Male;<br>Medium_Female - High_Male;<br>High_Male - Medium_Male;<br>Low_Female - Low_Male;<br>Low_Female - Medium_Female;<br>Low_Female - Medium_Male;<br>Medium_Female - Low_Male;<br>Low_Male - Medium_Male;<br>Medium_Female - Medium_Male	linc|TCONS_00020459		

283	XLOC_013445		3.0555	0.017324	1.7614	0.30602	High_Female - High_Male;<br>Low_Female - High_Female;<br>Low_Male - High_Female;<br>High_Female - Medium_Female;<br>Medium_Male - High_Female;<br>Low_Female - High_Male;<br>Low_Male - High_Male;<br>High_Male - Medium_Female;<br>Medium_Male - High_Male;<br>Low_Female - Low_Male;<br>Low_Female - Medium_Female;<br>Medium_Male - Low_Female;<br>Low_Male - Medium_Female;<br>Medium_Male - Low_Male;<br>Medium_Male - Medium_Female	linc|TCONS_00028489		
284	ADCY4	adenylate cyclase 4	3.0503	0.017469	1.7577	0.3075	High_Male - High_Female;<br>Low_Female - High_Female;<br>Low_Male - High_Female;<br>Medium_Female - High_Female;<br>Medium_Male - High_Female;<br>Low_Female - High_Male;<br>Low_Male - High_Male;<br>Medium_Female - High_Male;<br>High_Male - Medium_Male;<br>Low_Male - Low_Female;<br>Medium_Female - Low_Female;<br>Low_Female - Medium_Male;<br>Low_Male - Medium_Female;<br>Low_Male - Medium_Male;<br>Medium_Female - Medium_Male	A_33_P3337161	NM_001198568	NM_001198568

285	PAQR6	progestin and adipoQ receptor family member VI	3.0448	0.017625	1.7539	0.30915	High_Female - High_Male; High_Female - Low_Female; High_Female - Low_Male; High_Female - Medium_Female; High_Female - Medium_Male; High_Male - Low_Female; High_Male - Low_Male; Medium_Female - High_Male; High_Male - Medium_Male; Low_Female - Low_Male; Medium_Female - Low_Female; Medium_Male - Low_Female; Medium_Female - Low_Male; Medium_Male - Low_Male; Medium_Female - Medium_Male	A_23_P97283	NM_024897	NM_024897
286	XLOC_l2_013963		3.0388	0.017795	1.7497	0.31104	High_Male - High_Female; Low_Female - High_Female; Low_Male - High_Female; Medium_Female - High_Female; Medium_Male - High_Female; Low_Female - High_Male; Low_Male - High_Male; Medium_Female - High_Male; High_Male - Medium_Male; Low_Female - Low_Male; Low_Female - Medium_Female; Low_Female - Medium_Male; Low_Male - Medium_Female; Low_Male - Medium_Male; Medium_Female - Medium_Male	ens|ENST00000451962|tc|THC2722577|linc|TCONS_l2_00026848|linc|TCONS_l2_00026847		

287	SLC16A14	solute carrier family 16, member 14 (monocarboxylic acid transporter 14)	3.0228	0.018259	1.7385	0.31698	High_Male - High_Female; Low_Female - High_Female; Low_Male - High_Female; High_Female - Medium_Female; Medium_Male - High_Female; High_Male - Low_Female; Low_Male - High_Male; High_Male - Medium_Female; Medium_Male - High_Male; Low_Male - Low_Female; Low_Female - Medium_Female; Medium_Male - Low_Female; Low_Male - Medium_Female; Medium_Male - Low_Male; Medium_Male - Medium_Female	A_24_P342829	NM_152527	NM_152527
288	HNF1A-AS1	HNF1A antisense RNA 1 (non-protein coding)	3.0202	0.018333	1.7368	0.31698	High_Male - High_Female; Low_Female - High_Female; Low_Male - High_Female; Medium_Female - High_Female; Medium_Male - High_Female; Low_Female - High_Male; Low_Male - High_Male; Medium_Female - High_Male; High_Male - Medium_Male; Low_Male - Low_Female; Low_Female - Medium_Female; Low_Female - Medium_Male; Low_Male - Medium_Female; Low_Male - Medium_Male; Medium_Female - Medium_Male	A_24_P314515	NR_024345	NR_024345

289	TMEM164	transmembrane protein 164	3.0181	0.018395	1.7353	0.31698	High_Male - High_Female; Low_Female - High_Female; Low_Male - High_Female; Medium_Female - High_Female; Medium_Male - High_Female; High_Male - Low_Female; High_Male - Low_Male; High_Male - Medium_Female; High_Male - Medium_Male; Low_Male - Low_Female; Medium_Female - Low_Female; Medium_Male - Low_Female; Low_Male - Medium_Female; Medium_Male - Low_Male; Medium_Male - Medium_Female	A_23_P96041	NM_032227	NM_032227
290	XLOC_l2_001085		3.0166	0.018441	1.7342	0.31698	High_Male - High_Female; Low_Female - High_Female; Low_Male - High_Female; Medium_Female - High_Female; Medium_Male - High_Female; High_Male - Low_Female; Low_Male - High_Male; Medium_Female - High_Male; Medium_Male - High_Male; Low_Male - Low_Female; Medium_Female - Low_Female; Medium_Male - Low_Female; Low_Male - Medium_Female; Medium_Male - Low_Male; Medium_Male - Medium_Female	A_21_P0010646		AK090412

291	TNS1	tensin 1	3.0152	0.018483	1.7332	0.31698	High_Female - High_Male; Low_Female - High_Female; Low_Male - High_Female; Medium_Female - High_Female; High_Female - Medium_Male; Low_Female - High_Male; Low_Male - High_Male; Medium_Female - High_Male; Medium_Male - High_Male; Low_Male - Low_Female; Medium_Female - Low_Female; Low_Female - Medium_Male; Medium_Female - Low_Male; Low_Male - Medium_Male; Medium_Female - Medium_Male	A_33_P3738458	NM_022648	NM_022648
292	XLOC_004308		3.0083	0.018687	1.7285	0.31698	High_Female - High_Male; High_Female - Low_Female; High_Female - Low_Male; High_Female - Medium_Female; High_Female - Medium_Male; High_Male - Low_Female; High_Male - Low_Male; Medium_Female - High_Male; High_Male - Medium_Male; Low_Female - Low_Male; Medium_Female - Low_Female; Medium_Male - Low_Female; Medium_Female - Low_Male; Medium_Male - Low_Male; Medium_Female - Medium_Male	ens|ENST00000505844|ens|ENST00000507730|ens|ENST00000508677|tc|THC2714968		

293	RAB44	RAB44, member RAS oncogene family	3.0071	0.018722	1.7276	0.31698	High_Male - High_Female;<br>High_Female - Low_Female;<br>Low_Male - High_Female;<br>Medium_Female - High_Female;<br>Medium_Male - High_Female;<br>High_Male - Low_Female;<br>High_Male - Low_Male;<br>High_Male - Medium_Female;<br>High_Male - Medium_Male;<br>Low_Male - Low_Female;<br>Medium_Female - Low_Female;<br>Medium_Male - Low_Female;<br>Low_Male - Medium_Female;<br>Medium_Male - Low_Male;<br>Medium_Male - Medium_Female	A_33_P3349912		AK125083
294	MFSD6L	major facilitator superfamily domain containing 6-like	3.0002	0.018932	1.7228	0.31698	High_Male - High_Female;<br>Low_Female - High_Female;<br>Low_Male - High_Female;<br>High_Female - Medium_Female;<br>Medium_Male - High_Female;<br>High_Male - Low_Female;<br>High_Male - Low_Male;<br>High_Male - Medium_Female;<br>Medium_Male - High_Male;<br>Low_Male - Low_Female;<br>Low_Female - Medium_Female;<br>Medium_Male - Low_Female;<br>Low_Male - Medium_Female;<br>Medium_Male - Low_Male;<br>Medium_Male - Medium_Female	A_33_P3393331	NM_152599	NM_152599

295	NR4A2	nuclear receptor subfamily 4, group A, member 2	3.0001	0.018934	1.7227	0.31698	High_Male - High_Female; Low_Female - High_Female; Low_Male - High_Female; High_Female - Medium_Female; Medium_Male - High_Female; High_Male - Low_Female; Low_Male - High_Male; High_Male - Medium_Female; High_Male - Medium_Male; Low_Male - Low_Female; Low_Female - Medium_Female; Low_Female - Medium_Male; Low_Male - Medium_Female; Low_Male - Medium_Male; Medium_Male - Medium_Female	A_23_P131208	NM_006186	NM_006186
296	XLOC_011924		2.9975	0.019014	1.7209	0.31698	High_Male - High_Female; Low_Female - High_Female; Low_Male - High_Female; Medium_Female - High_Female; Medium_Male - High_Female; Low_Female - High_Male; Low_Male - High_Male; High_Male - Medium_Female; High_Male - Medium_Male; Low_Male - Low_Female; Low_Female - Medium_Female; Low_Female - Medium_Male; Low_Male - Medium_Female; Low_Male - Medium_Male; Medium_Female - Medium_Male	A_21_P0009023		BI055436

297	XLOC_007868		2.9962	0.019054	1.72	0.31698	High_Male - High_Female; Low_Female - High_Female; Low_Male - High_Female; Medium_Female - High_Female; Medium_Male - High_Female; Low_Female - High_Male; Low_Male - High_Male; Medium_Female - High_Male; High_Male - Medium_Male; Low_Male - Low_Female; Low_Female - Medium_Female; Low_Female - Medium_Male; Low_Male - Medium_Female; Low_Male - Medium_Male; Medium_Female - Medium_Male	linc|TCONS_00016491		
298	CNR2	cannabinoid receptor 2 (macrophage)	2.9928	0.019157	1.7177	0.31698	High_Male - High_Female; Low_Female - High_Female; Low_Male - High_Female; Medium_Female - High_Female; Medium_Male - High_Female; High_Male - Low_Female; High_Male - Low_Male; High_Male - Medium_Female; Medium_Male - High_Male; Low_Male - Low_Female; Medium_Female - Low_Female; Medium_Male - Low_Female; Low_Male - Medium_Female; Medium_Male - Low_Male; Medium_Male - Medium_Female	A_23_P310931	NM_001841	NM_001841

299	PDK2	pyruvate dehydrogenase kinase, isozyme 2	2.9924	0.01917	1.7174	0.31698	High_Male - High_Female; Low_Female - High_Female; Low_Male - High_Female; Medium_Female - High_Female; Medium_Male - High_Female; High_Male - Low_Female; Low_Male - High_Male; High_Male - Medium_Female; High_Male - Medium_Male; Low_Male - Low_Female; Medium_Female - Low_Female; Medium_Male - Low_Female; Low_Male - Medium_Female; Low_Male - Medium_Male; Medium_Male - Medium_Female	A_21_P0000146	NM_001199900	NM_001199900
300	OVGP1	oviductal glycoprotein 1, 120kDa	2.9911	0.01921	1.7165	0.31698	High_Female - High_Male; Low_Female - High_Female; Low_Male - High_Female; High_Female - Medium_Female; Medium_Male - High_Female; Low_Female - High_Male; Low_Male - High_Male; High_Male - Medium_Female; Medium_Male - High_Male; Low_Male - Low_Female; Low_Female - Medium_Female; Low_Female - Medium_Male; Low_Male - Medium_Female; Low_Male - Medium_Male; Medium_Male - Medium_Female	A_23_P103756	NM_002557	NM_002557

301	CD69	CD69 molecule	2.9888	0.019281	1.7149	0.31698	High_Male - High_Female; Low_Female - High_Female; Low_Male - High_Female; Medium_Female - High_Female; Medium_Male - High_Female; High_Male - Low_Female; Low_Male - High_Male; High_Male - Medium_Female; High_Male - Medium_Male; Low_Male - Low_Female; Low_Female - Medium_Female; Medium_Male - Low_Female; Low_Male - Medium_Female; Low_Male - Medium_Male; Medium_Male - Medium_Female	A_33_P3241021		AK303383
302	WNK4	WNK lysine deficient protein kinase 4	2.9883	0.019297	1.7145	0.31698	High_Male - High_Female; Low_Female - High_Female; Low_Male - High_Female; Medium_Female - High_Female; Medium_Male - High_Female; Low_Female - High_Male; Low_Male - High_Male; High_Male - Medium_Female; High_Male - Medium_Male; Low_Female - Low_Male; Low_Female - Medium_Female; Low_Female - Medium_Male; Low_Male - Medium_Female; Low_Male - Medium_Male; Medium_Female - Medium_Male	A_33_P3377994	NM_032387	NM_032387

303	OLFM4	olfactomedin 4	2.9875	0.01932	1.714	0.31698	High_Female - High_Male; High_Female - Low_Female; High_Female - Low_Male; Medium_Female - High_Female; High_Female - Medium_Male; High_Male - Low_Female; High_Male - Low_Male; Medium_Female - High_Male; Medium_Male - High_Male; Low_Male - Low_Female; Medium_Female - Low_Female; Medium_Male - Low_Female; Medium_Female - Low_Male; Medium_Male - Low_Male; Medium_Female - Medium_Male	A_24_P181254	NM_006418	NM_006418
304	DPP6	dipeptidyl-peptidase 6	2.9868	0.019343	1.7135	0.31698	High_Male - High_Female; Low_Female - High_Female; Low_Male - High_Female; Medium_Female - High_Female; Medium_Male - High_Female; Low_Female - High_Male; High_Male - Low_Male; High_Male - Medium_Female; High_Male - Medium_Male; Low_Female - Low_Male; Low_Female - Medium_Female; Low_Female - Medium_Male; Medium_Female - Low_Male; Low_Male - Medium_Male; Medium_Female - Medium_Male	A_24_P59111		BC035912

305	XLOC_004535		2.9837	0.01944	1.7113	0.31698	High_Male - High_Female; Low_Female - High_Female; Low_Male - High_Female; Medium_Female - High_Female; Medium_Male - High_Female; Low_Female - High_Male; High_Male - Low_Male; High_Male - Medium_Female; High_Male - Medium_Male; Low_Female - Low_Male; Low_Female - Medium_Female; Low_Female - Medium_Male; Low_Male - Medium_Female; Low_Male - Medium_Male; Medium_Female - Medium_Male	ens|ENST00000509456|linc|TCONS_00009501		
306	COL20A1	collagen, type XX, alpha 1	2.9826	0.019473	1.7106	0.31698	High_Female - High_Male; High_Female - Low_Female; High_Female - Low_Male; High_Female - Medium_Female; High_Female - Medium_Male; Low_Female - High_Male; High_Male - Low_Male; Medium_Female - High_Male; Medium_Male - High_Male; Low_Female - Low_Male; Medium_Female - Low_Female; Medium_Male - Low_Female; Medium_Female - Low_Male; Medium_Male - Low_Male; Medium_Male - Medium_Female	A_33_P3395952	NM_020882	NM_020882

307	ALPL	alkaline phosphatase, liver/bone/kidney	2.9818	0.019499	1.71	0.31698	High_Female - High_Male; High_Female - Low_Female; High_Female - Low_Male; High_Female - Medium_Female; High_Female - Medium_Male; High_Male - Low_Female; High_Male - Low_Male; Medium_Female - High_Male; Medium_Male - High_Male; Low_Female - Low_Male; Medium_Female - Low_Female; Medium_Male - Low_Female; Medium_Female - Low_Male; Medium_Male - Low_Male; Medium_Female - Medium_Male	A_24_P353619	NM_000478	NM_000478
308	PSPHP1	phosphoserine phosphatase pseudogene 1	2.9808	0.01953	1.7093	0.31698	High_Male - High_Female; High_Female - Low_Female; High_Female - Low_Male; Medium_Female - High_Female; Medium_Male - High_Female; High_Male - Low_Female; High_Male - Low_Male; Medium_Female - High_Male; Medium_Male - High_Male; Low_Male - Low_Female; Medium_Female - Low_Female; Medium_Male - Low_Female; Medium_Female - Low_Male; Medium_Male - Low_Male; Medium_Female - Medium_Male	A_21_P0013198		BC065228

309	GGT8P	gamma-glutamyltransferase 8 pseudogene	2.9717	0.019817	1.703	0.31932	High_Female - High_Male;<br>Low_Female - High_Female;<br>Low_Male - High_Female;<br>High_Female - Medium_Female;<br>Medium_Male - High_Female;<br>Low_Female - High_Male;<br>Low_Male - High_Male;<br>Medium_Female - High_Male;<br>Medium_Male - High_Male;<br>Low_Male - Low_Female;<br>Low_Female - Medium_Female;<br>Medium_Male - Low_Female;<br>Low_Male - Medium_Female;<br>Low_Male - Medium_Male;<br>Medium_Male - Medium_Female	A_24_P375322	NR_003503	NR_003503
310	KRTAP19-2	keratin associated protein 19-2	2.9704	0.019861	1.702	0.31932	High_Female - High_Male;<br>High_Female - Low_Female;<br>Low_Male - High_Female;<br>Medium_Female - High_Female;<br>High_Female - Medium_Male;<br>Low_Female - High_Male;<br>Low_Male - High_Male;<br>Medium_Female - High_Male;<br>High_Male - Medium_Male;<br>Low_Male - Low_Female;<br>Medium_Female - Low_Female;<br>Low_Female - Medium_Male;<br>Medium_Female - Low_Male;<br>Low_Male - Medium_Male;<br>Medium_Female - Medium_Male	A_33_P3311267	NM_181608	NM_181608

311			2.9702	0.019866	1.7019	0.31932	High_Female - High_Male; High_Female - Low_Female; High_Female - Low_Male; High_Female - Medium_Female; High_Female - Medium_Male; High_Male - Low_Female; High_Male - Low_Male; Medium_Female - High_Male; High_Male - Medium_Male; Low_Female - Low_Male; Medium_Female - Low_Female; Low_Female - Medium_Male; Medium_Female - Low_Male; Low_Male - Medium_Male; Medium_Female - Medium_Male	tc|THC2739199		
312	CCDC144A	coiled-coil domain containing 144A	2.9666	0.019982	1.6994	0.32016	High_Male - High_Female; Low_Female - High_Female; Low_Male - High_Female; Medium_Female - High_Female; Medium_Male - High_Female; Low_Female - High_Male; Low_Male - High_Male; Medium_Female - High_Male; Medium_Male - High_Male; Low_Male - Low_Female; Medium_Female - Low_Female; Medium_Male - Low_Female; Low_Male - Medium_Female; Low_Male - Medium_Male; Medium_Female - Medium_Male	A_21_P0011525	NM_014695	NM_014695

313	LOC100132987	uncharacterized LOC100132987	2.9631	0.020093	1.697	0.32082	High_Male - High_Female; Low_Female - High_Female; Low_Male - High_Female; Medium_Female - High_Female; Medium_Male - High_Female; High_Male - Low_Female; Low_Male - High_Male; High_Male - Medium_Female; High_Male - Medium_Male; Low_Male - Low_Female; Medium_Female - Low_Female; Low_Female - Medium_Male; Low_Male - Medium_Female; Low_Male - Medium_Male; Medium_Female - Medium_Male	A_21_P0000862	NR_038985	NR_038985
314	XLOC_l2_013963		2.9613	0.020152	1.6957	0.32082	High_Male - High_Female; Low_Female - High_Female; Low_Male - High_Female; Medium_Female - High_Female; Medium_Male - High_Female; Low_Female - High_Male; High_Male - Low_Male; Medium_Female - High_Male; High_Male - Medium_Male; Low_Female - Low_Male; Medium_Female - Low_Female; Low_Female - Medium_Male; Medium_Female - Low_Male; Low_Male - Medium_Male; Medium_Female - Medium_Male	linc|TCONS_l2_00026845		

315	ACOT11	acyl-CoA thioesterase 11	2.9565	0.020309	1.6923	0.32229	High_Male - High_Female; High_Female - Low_Female; Low_Male - High_Female; Medium_Female - High_Female; Medium_Male - High_Female; High_Male - Low_Female; High_Male - Low_Male; High_Male - Medium_Female; High_Male - Medium_Male; Low_Male - Low_Female; Medium_Female - Low_Female; Medium_Male - Low_Female; Medium_Female - Low_Male; Medium_Male - Low_Male; Medium_Male - Medium_Female	A_23_P417415	NM_147161	NM_147161
316	LOC339807	uncharacterized LOC339807	2.9452	0.020679	1.6845	0.32713	High_Male - High_Female; Low_Female - High_Female; Low_Male - High_Female; Medium_Female - High_Female; High_Female - Medium_Male; Low_Female - High_Male; Low_Male - High_Male; High_Male - Medium_Female; High_Male - Medium_Male; Low_Female - Low_Male; Low_Female - Medium_Female; Low_Female - Medium_Male; Low_Male - Medium_Female; Low_Male - Medium_Male; Medium_Female - Medium_Male	A_33_P3512350	NR_034023	NR_034023

317			2.9398	0.02086	1.6807	0.32895	High_Male - High_Female; Low_Female - High_Female; Low_Male - High_Female; Medium_Female - High_Female; Medium_Male - High_Female; Low_Female - High_Male; Low_Male - High_Male; Medium_Female - High_Male; High_Male - Medium_Male; Low_Male - Low_Female; Low_Female - Medium_Female; Low_Female - Medium_Male; Low_Male - Medium_Female; Low_Male - Medium_Male; Medium_Female - Medium_Male	A_33_P3369266		CR997556
318	OLIG2	oligodendrocyte lineage transcription factor 2	2.9358	0.020995	1.6779	0.3294	High_Male - High_Female; Low_Female - High_Female; Low_Male - High_Female; Medium_Female - High_Female; Medium_Male - High_Female; High_Male - Low_Female; High_Male - Low_Male; High_Male - Medium_Female; Medium_Male - High_Male; Low_Male - Low_Female; Medium_Female - Low_Female; Medium_Male - Low_Female; Medium_Female - Low_Male; Medium_Male - Low_Male; Medium_Male - Medium_Female	A_23_P211079	NM_005806	NM_005806

319	LPCAT2	lysophosphatidylcholine acyltransferase 2	2.9319	0.021125	1.6752	0.3294	High_Male - High_Female; High_Female - Low_Female; High_Female - Low_Male; High_Female - Medium_Female; High_Female - Medium_Male; High_Male - Low_Female; High_Male - Low_Male; High_Male - Medium_Female; High_Male - Medium_Male; Low_Female - Low_Male; Medium_Female - Low_Female; Medium_Male - Low_Female; Medium_Female - Low_Male; Medium_Male - Low_Male; Medium_Male - Medium_Female	A_23_P141021	NM_017839	NM_017839
320	XLOC_007268		2.931	0.021156	1.6746	0.3294	High_Male - High_Female; Low_Female - High_Female; High_Female - Low_Male; Medium_Female - High_Female; High_Female - Medium_Male; Low_Female - High_Male; High_Male - Low_Male; Medium_Female - High_Male; High_Male - Medium_Male; Low_Female - Low_Male; Low_Female - Medium_Female; Low_Female - Medium_Male; Medium_Female - Low_Male; Medium_Male - Low_Male; Medium_Female - Medium_Male	A_21_P0005997		BC041456

321	NAMPT	nicotinamide phosphoribosyltransferase	2.9302	0.021183	1.674	0.3294	High_Female - High_Male;<br>Low_Female - High_Female;<br>Low_Male - High_Female;<br>High_Female - Medium_Female;<br>Medium_Male - High_Female;<br>Low_Female - High_Male;<br>Low_Male - High_Male;<br>High_Male - Medium_Female;<br>Medium_Male - High_Male;<br>Low_Male - Low_Female;<br>Low_Female - Medium_Female;<br>Low_Female - Medium_Male;<br>Low_Male - Medium_Female;<br>Low_Male - Medium_Male;<br>Medium_Male - Medium_Female	A_33_P3364869		AK023341
322			2.9292	0.021217	1.6733	0.3294	High_Male - High_Female;<br>Low_Female - High_Female;<br>Low_Male - High_Female;<br>Medium_Female - High_Female;<br>Medium_Male - High_Female;<br>High_Male - Low_Female;<br>Low_Male - High_Male;<br>High_Male - Medium_Female;<br>High_Male - Medium_Male;<br>Low_Male - Low_Female;<br>Medium_Female - Low_Female;<br>Low_Female - Medium_Male;<br>Low_Male - Medium_Female;<br>Low_Male - Medium_Male;<br>Medium_Female - Medium_Male	A_24_P281636		AY010113

323	C19orf59	chromosome 19 open reading frame 59	2.9233	0.021419	1.6692	0.32964	High_Female - High_Male; High_Female - Low_Female; High_Female - Low_Male; High_Female - Medium_Female; High_Female - Medium_Male; High_Male - Low_Female; High_Male - Low_Male; Medium_Female - High_Male; Medium_Male - High_Male; Low_Female - Low_Male; Medium_Female - Low_Female; Medium_Male - Low_Female; Medium_Female - Low_Male; Medium_Male - Low_Male; Medium_Female - Medium_Male	A_23_P330561	NM_174918	NM_174918
324	ABCD4	ATP-binding cassette, sub-family D (ALD), member 4	2.9233	0.02142	1.6692	0.32964	High_Male - High_Female; Low_Female - High_Female; Low_Male - High_Female; Medium_Female - High_Female; Medium_Male - High_Female; Low_Female - High_Male; High_Male - Low_Male; Medium_Female - High_Male; High_Male - Medium_Male; Low_Female - Low_Male; Low_Female - Medium_Female; Low_Female - Medium_Male; Medium_Female - Low_Male; Low_Male - Medium_Male; Medium_Female - Medium_Male	A_33_P3399598		AK125109

325			2.9179	0.021608	1.6654	0.32964	High_Male - High_Female; Low_Female - High_Female; Low_Male - High_Female; Medium_Female - High_Female; Medium_Male - High_Female; Low_Female - High_Male; Low_Male - High_Male; High_Male - Medium_Female; High_Male - Medium_Male; Low_Male - Low_Female; Low_Female - Medium_Female; Low_Female - Medium_Male; Low_Male - Medium_Female; Low_Male - Medium_Male; Medium_Female - Medium_Male	A_33_P3415648	XR_132817	XR_132817
326	KCNG1	potassium voltage-gated channel, subfamily G, member 1	2.9177	0.021615	1.6652	0.32964	High_Male - High_Female; Low_Female - High_Female; Low_Male - High_Female; Medium_Female - High_Female; Medium_Male - High_Female; Low_Female - High_Male; Low_Male - High_Male; Medium_Female - High_Male; Medium_Male - High_Male; Low_Male - Low_Female; Low_Female - Medium_Female; Medium_Male - Low_Female; Low_Male - Medium_Female; Medium_Male - Low_Male; Medium_Male - Medium_Female	A_23_P210581	NM_002237	NM_002237

327	ZNRF3	zinc and ring finger 3	2.9176	0.021616	1.6652	0.32964	High_Male - High_Female; Low_Female - High_Female; Low_Male - High_Female; Medium_Female - High_Female; Medium_Male - High_Female; High_Male - Low_Female; High_Male - Low_Male; High_Male - Medium_Female; High_Male - Medium_Male; Low_Male - Low_Female; Medium_Female - Low_Female; Medium_Male - Low_Female; Low_Male - Medium_Female; Medium_Male - Low_Male; Medium_Male - Medium_Female	A_24_P162373	NM_001206998	NM_001206998
328	XLOC_003405		2.9126	0.02179	1.6617	0.32964	High_Female - High_Male; Low_Female - High_Female; Low_Male - High_Female; High_Female - Medium_Female; Medium_Male - High_Female; Low_Female - High_Male; Low_Male - High_Male; Medium_Female - High_Male; Medium_Male - High_Male; Low_Female - Low_Male; Low_Female - Medium_Female; Medium_Male - Low_Female; Low_Male - Medium_Female; Medium_Male - Low_Male; Medium_Male - Medium_Female	tc|THC2503819|linc|TCONS_00006771|linc|TCONS_00006770|linc|TCONS_00007353		

329	APBA2	amyloid beta (A4) precursor protein-binding, family A, member 2	2.9122	0.021805	1.6614	0.32964	High_Male - High_Female;<br>Low_Female - High_Female;<br>Low_Male - High_Female;<br>Medium_Female - High_Female;<br>Medium_Male - High_Female;<br>Low_Female - High_Male;<br>Low_Male - High_Male;<br>Medium_Female - High_Male;<br>Medium_Male - High_Male;<br>Low_Male - Low_Female;<br>Low_Female - Medium_Female;<br>Low_Female - Medium_Male;<br>Low_Male - Medium_Female;<br>Low_Male - Medium_Male;<br>Medium_Male - Medium_Female	A_33_P3344504		AK124794
330			2.9103	0.02187	1.6601	0.32964	High_Male - High_Female;<br>Low_Female - High_Female;<br>Low_Male - High_Female;<br>Medium_Female - High_Female;<br>High_Female - Medium_Male;<br>Low_Female - High_Male;<br>Low_Male - High_Male;<br>Medium_Female - High_Male;<br>High_Male - Medium_Male;<br>Low_Female - Low_Male;<br>Low_Female - Medium_Female;<br>Low_Female - Medium_Male;<br>Medium_Female - Low_Male;<br>Low_Male - Medium_Male;<br>Medium_Female - Medium_Male	A_33_P3322103		

331	XLOC_000535		2.9099	0.021886	1.6598	0.32964	High_Male - High_Female; Low_Female - High_Female; Low_Male - High_Female; Medium_Female - High_Female; Medium_Male - High_Female; High_Male - Low_Female; Low_Male - High_Male; High_Male - Medium_Female; High_Male - Medium_Male; Low_Male - Low_Female; Medium_Female - Low_Female; Low_Female - Medium_Male; Low_Male - Medium_Female; Low_Male - Medium_Male; Medium_Female - Medium_Male	linc|TCONS_00001250		
332	FCGR1B	Fc fragment of IgG, high affinity Ib, receptor (CD64)	2.9097	0.021892	1.6597	0.32964	High_Male - High_Female; Low_Female - High_Female; High_Female - Low_Male; High_Female - Medium_Female; High_Female - Medium_Male; Low_Female - High_Male; High_Male - Low_Male; High_Male - Medium_Female; High_Male - Medium_Male; Low_Female - Low_Male; Low_Female - Medium_Female; Low_Female - Medium_Male; Medium_Female - Low_Male; Medium_Male - Low_Male; Medium_Female - Medium_Male	A_23_P63390	NM_001017986	NM_001017986

333	XLOC_007895		2.9037	0.022105	1.6555	0.33184	High_Male - High_Female; Low_Female - High_Female; Low_Male - High_Female; Medium_Female - High_Female; Medium_Male - High_Female; Low_Female - High_Male; Low_Male - High_Male; High_Male - Medium_Female; High_Male - Medium_Male; Low_Male - Low_Female; Low_Female - Medium_Female; Low_Female - Medium_Male; Low_Male - Medium_Female; Low_Male - Medium_Male; Medium_Female - Medium_Male	linc|TCONS_00016511		
334	KLF14	Kruppel-like factor 14	2.895	0.022417	1.6494	0.33537	High_Male - High_Female; Low_Female - High_Female; Low_Male - High_Female; Medium_Female - High_Female; Medium_Male - High_Female; Low_Female - High_Male; Low_Male - High_Male; Medium_Female - High_Male; High_Male - Medium_Male; Low_Male - Low_Female; Low_Female - Medium_Female; Low_Female - Medium_Male; Low_Male - Medium_Female; Low_Male - Medium_Male; Medium_Female - Medium_Male	A_33_P3374947	NM_138693	NM_138693

335	IDO1	indoleamine 2,3-dioxygenase 1	2.8934	0.022474	1.6483	0.33537	High_Male - High_Female;<br>Low_Female - High_Female;<br>Low_Male - High_Female;<br>Medium_Female - High_Female;<br>Medium_Male - High_Female;<br>High_Male - Low_Female;<br>High_Male - Low_Male;<br>High_Male - Medium_Female;<br>High_Male - Medium_Male;<br>Low_Female - Low_Male;<br>Medium_Female - Low_Female;<br>Medium_Male - Low_Female;<br>Medium_Female - Low_Male;<br>Medium_Male - Low_Male;<br>Medium_Male - Medium_Female	A_23_P112026	NM_002164	NM_002164
336			2.882	0.022888	1.6404	0.34003	High_Male - High_Female;<br>Low_Female - High_Female;<br>Low_Male - High_Female;<br>Medium_Female - High_Female;<br>Medium_Male - High_Female;<br>High_Male - Low_Female;<br>High_Male - Low_Male;<br>High_Male - Medium_Female;<br>High_Male - Medium_Male;<br>Low_Female - Low_Male;<br>Low_Female - Medium_Female;<br>Low_Female - Medium_Male;<br>Medium_Female - Low_Male;<br>Low_Male - Medium_Male;<br>Medium_Female - Medium_Male	tc|THC2481061		

337	VPRBP	Vpr (HIV-1) binding protein	2.8811	0.022923	1.6397	0.34003	High_Male - High_Female; Low_Female - High_Female; Low_Male - High_Female; Medium_Female - High_Female; Medium_Male - High_Female; Low_Female - High_Male; Low_Male - High_Male; Medium_Female - High_Male; Medium_Male - High_Male; Low_Male - Low_Female; Low_Female - Medium_Female; Medium_Male - Low_Female; Low_Male - Medium_Female; Low_Male - Medium_Male; Medium_Male - Medium_Female	A_23_P212595	NM_014703	NM_014703
338	LOC497256	uncharacterized LOC497256	2.8772	0.023067	1.637	0.34101	High_Male - High_Female; Low_Female - High_Female; Low_Male - High_Female; Medium_Female - High_Female; Medium_Male - High_Female; Low_Female - High_Male; Low_Male - High_Male; High_Male - Medium_Female; High_Male - Medium_Male; Low_Female - Low_Male; Low_Female - Medium_Female; Low_Female - Medium_Male; Low_Male - Medium_Female; Low_Male - Medium_Male; Medium_Female - Medium_Male	A_33_P3760937		AK094988

339	UNC5C	unc-5 homolog C (C. elegans)	2.8746	0.023165	1.6352	0.34101	High_Male - High_Female; Low_Female - High_Female; Low_Male - High_Female; Medium_Female - High_Female; Medium_Male - High_Female; Low_Female - High_Male; Low_Male - High_Male; High_Male - Medium_Female; High_Male - Medium_Male; Low_Male - Low_Female; Low_Female - Medium_Female; Low_Female - Medium_Male; Low_Male - Medium_Female; Low_Male - Medium_Male; Medium_Female - Medium_Male	A_23_P69617	NM_003728	NM_003728
340	NR4A3	nuclear receptor subfamily 4, group A, member 3	2.8738	0.023194	1.6346	0.34101	High_Male - High_Female; Low_Female - High_Female; Low_Male - High_Female; Medium_Female - High_Female; Medium_Male - High_Female; High_Male - Low_Female; High_Male - Low_Male; High_Male - Medium_Female; High_Male - Medium_Male; Low_Male - Low_Female; Low_Female - Medium_Female; Medium_Male - Low_Female; Low_Male - Medium_Female; Medium_Male - Low_Male; Medium_Male - Medium_Female	A_23_P398566	NM_173200	NM_173200

341	DUX4	double homeobox 4	2.8606	0.023691	1.6254	0.34521	High_Male - High_Female; Low_Female - High_Female; Low_Male - High_Female; Medium_Female - High_Female; High_Female - Medium_Male; Low_Female - High_Male; Low_Male - High_Male; High_Male - Medium_Female; High_Male - Medium_Male; Low_Female - Low_Male; Low_Female - Medium_Female; Low_Female - Medium_Male; Low_Male - Medium_Female; Low_Male - Medium_Male; Medium_Female - Medium_Male	A_33_P3258699	NM_033178	NM_033178
342	CD248	CD248 molecule, endosialin	2.8596	0.023727	1.6247	0.34521	High_Male - High_Female; Low_Female - High_Female; Low_Male - High_Female; Medium_Female - High_Female; Medium_Male - High_Female; High_Male - Low_Female; High_Male - Low_Male; High_Male - Medium_Female; Medium_Male - High_Male; Low_Male - Low_Female; Low_Female - Medium_Female; Medium_Male - Low_Female; Low_Male - Medium_Female; Medium_Male - Low_Male; Medium_Male - Medium_Female	A_33_P3337485	NM_020404	NM_020404

343	SNORA39	small nucleolar RNA, H/ACA box 39	2.8582	0.023784	1.6237	0.34521	High_Male - High_Female; Low_Female - High_Female; Low_Male - High_Female; Medium_Female - High_Female; High_Female - Medium_Male; High_Male - Low_Female; High_Male - Low_Male; High_Male - Medium_Female; High_Male - Medium_Male; Low_Female - Low_Male; Low_Female - Medium_Female; Low_Female - Medium_Male; Low_Male - Medium_Female; Low_Male - Medium_Male; Medium_Female - Medium_Male	A_21_P0000327	NR_002972	NR_002972
344	TMCC2	transmembrane and coiled-coil domain family 2	2.8574	0.023814	1.6232	0.34521	High_Female - High_Male; High_Female - Low_Female; High_Female - Low_Male; Medium_Female - High_Female; High_Female - Medium_Male; Low_Female - High_Male; Low_Male - High_Male; Medium_Female - High_Male; Medium_Male - High_Male; Low_Female - Low_Male; Medium_Female - Low_Female; Medium_Male - Low_Female; Medium_Female - Low_Male; Medium_Male - Low_Male; Medium_Female - Medium_Male	A_32_P133840	NM_014858	NM_014858

345	CA4	carbonic anhydrase IV	2.8571	0.023824	1.623	0.34521	High_Female - High_Male; High_Female - Low_Female; High_Female - Low_Male; High_Female - Medium_Female; High_Female - Medium_Male; High_Male - Low_Female; High_Male - Low_Male; Medium_Female - High_Male; High_Male - Medium_Male; Low_Female - Low_Male; Medium_Female - Low_Female; Medium_Male - Low_Female; Medium_Female - Low_Male; Medium_Male - Low_Male; Medium_Female - Medium_Male	A_23_P4096	NM_000717	NM_000717
346			2.8504	0.024083	1.6183	0.34656	High_Male - High_Female; Low_Female - High_Female; Low_Male - High_Female; Medium_Female - High_Female; Medium_Male - High_Female; Low_Female - High_Male; Low_Male - High_Male; High_Male - Medium_Female; High_Male - Medium_Male; Low_Female - Low_Male; Low_Female - Medium_Female; Low_Female - Medium_Male; Low_Male - Medium_Female; Low_Male - Medium_Male; Medium_Female - Medium_Male	tc|THC2764040		

347	LOC100134391	uncharacterized LOC100134391	2.8497	0.02411	1.6178	0.34656	High_Male - High_Female; Low_Female - High_Female; Low_Male - High_Female; Medium_Female - High_Female; Medium_Male - High_Female; Low_Female - High_Male; Low_Male - High_Male; High_Male - Medium_Female; High_Male - Medium_Male; Low_Male - Low_Female; Low_Female - Medium_Female; Low_Female - Medium_Male; Low_Male - Medium_Female; Low_Male - Medium_Male; Medium_Female - Medium_Male	A_21_P0014355	XR_109425	XR_109425
348	XLOC_000751		2.8493	0.024126	1.6175	0.34656	High_Male - High_Female; Low_Female - High_Female; Low_Male - High_Female; Medium_Female - High_Female; Medium_Male - High_Female; High_Male - Low_Female; Low_Male - High_Male; Medium_Female - High_Male; High_Male - Medium_Male; Low_Male - Low_Female; Medium_Female - Low_Female; Low_Female - Medium_Male; Low_Male - Medium_Female; Low_Male - Medium_Male; Medium_Female - Medium_Male	linc|TCONS_00001436		

349	XLOC_l2_015855		2.847	0.024215	1.6159	0.34685	High_Male - High_Female; Low_Female - High_Female; Low_Male - High_Female; Medium_Female - High_Female; Medium_Male - High_Female; Low_Female - High_Male; Low_Male - High_Male; High_Male - Medium_Female; High_Male - Medium_Male; Low_Male - Low_Female; Low_Female - Medium_Female; Low_Female - Medium_Male; Low_Male - Medium_Female; Low_Male - Medium_Male; Medium_Female - Medium_Male	ens|ENST00000444722|linc|TCONS_l2_00030882		
350	FCGR1B	Fc fragment of IgG, high affinity Ib, receptor (CD64)	2.8441	0.02433	1.6139	0.3475	High_Female - High_Male; Low_Female - High_Female; High_Female - Low_Male; High_Female - Medium_Female; High_Female - Medium_Male; Low_Female - High_Male; High_Male - Low_Male; High_Male - Medium_Female; High_Male - Medium_Male; Low_Female - Low_Male; Low_Female - Medium_Female; Low_Female - Medium_Male; Medium_Female - Low_Male; Medium_Male - Low_Male; Medium_Female - Medium_Male	A_21_P0010728	NM_001244910	NM_001244910

351	XLOC_012166		2.8362	0.024641	1.6083	0.35094	High_Male - High_Female; Low_Female - High_Female; Low_Male - High_Female; Medium_Female - High_Female; Medium_Male - High_Female; Low_Female - High_Male; High_Male - Low_Male; High_Male - Medium_Female; High_Male - Medium_Male; Low_Female - Low_Male; Low_Female - Medium_Female; Low_Female - Medium_Male; Low_Male - Medium_Female; Low_Male - Medium_Male; Medium_Female - Medium_Male	linc|TCONS_00025346		
352	XLOC_l2_004611		2.8303	0.024876	1.6042	0.35328	High_Male - High_Female; Low_Female - High_Female; Low_Male - High_Female; Medium_Female - High_Female; Medium_Male - High_Female; Low_Female - High_Male; Low_Male - High_Male; Medium_Female - High_Male; Medium_Male - High_Male; Low_Male - Low_Female; Low_Female - Medium_Female; Low_Female - Medium_Male; Low_Male - Medium_Female; Low_Male - Medium_Male; Medium_Male - Medium_Female	tc|THC2520829|linc|TCONS_l2_00008540		

353	XLOC_007407		2.8244	0.02511	1.6002	0.3551	High_Male - High_Female; Low_Female - High_Female; Low_Male - High_Female; Medium_Female - High_Female; Medium_Male - High_Female; Low_Female - High_Male; Low_Male - High_Male; High_Male - Medium_Female; High_Male - Medium_Male; Low_Female - Low_Male; Low_Female - Medium_Female; Low_Female - Medium_Male; Low_Male - Medium_Female; Low_Male - Medium_Male; Medium_Male - Medium_Female	linc|TCONS_00016016|linc|TCONS_00016015|linc|TCONS_00016621		
354	NCAPH	non-SMC condensin I complex, subunit H	2.8234	0.025151	1.5994	0.3551	High_Male - High_Female; Low_Female - High_Female; Low_Male - High_Female; Medium_Female - High_Female; Medium_Male - High_Female; Low_Female - High_Male; Low_Male - High_Male; High_Male - Medium_Female; High_Male - Medium_Male; Low_Female - Low_Male; Low_Female - Medium_Female; Low_Female - Medium_Male; Low_Male - Medium_Female; Low_Male - Medium_Male; Medium_Female - Medium_Male	A_33_P3230259	NM_015341	NM_015341

355	XLOC_005082		2.8201	0.025287	1.5971	0.3551	High_Female - High_Male; High_Female - Low_Female; Low_Male - High_Female; Medium_Female - High_Female; High_Female - Medium_Male; Low_Female - High_Male; Low_Male - High_Male; Medium_Female - High_Male; Medium_Male - High_Male; Low_Male - Low_Female; Medium_Female - Low_Female; Low_Female - Medium_Male; Low_Male - Medium_Female; Low_Male - Medium_Male; Medium_Female - Medium_Male	linc|TCONS_00010555		
356			2.8181	0.025365	1.5958	0.3551	High_Male - High_Female; Low_Female - High_Female; Low_Male - High_Female; Medium_Female - High_Female; Medium_Male - High_Female; Low_Female - High_Male; Low_Male - High_Male; High_Male - Medium_Female; High_Male - Medium_Male; Low_Male - Low_Female; Low_Female - Medium_Female; Low_Female - Medium_Male; Low_Male - Medium_Female; Low_Male - Medium_Male; Medium_Female - Medium_Male	A_33_P3260110		AK126960

357	XLOC_003400		2.8169	0.025416	1.5949	0.3551	High_Female - High_Male; High_Female - Low_Female; High_Female - Low_Male; Medium_Female - High_Female; Medium_Male - High_Female; High_Male - Low_Female; High_Male - Low_Male; Medium_Female - High_Male; Medium_Male - High_Male; Low_Female - Low_Male; Medium_Female - Low_Female; Medium_Male - Low_Female; Medium_Female - Low_Male; Medium_Male - Low_Male; Medium_Male - Medium_Female	linc|TCONS_00007354		
358	LOC100505978	uncharacterized LOC100505978	2.8165	0.025431	1.5946	0.3551	High_Male - High_Female; Low_Female - High_Female; Low_Male - High_Female; Medium_Female - High_Female; High_Female - Medium_Male; Low_Female - High_Male; Low_Male - High_Male; Medium_Female - High_Male; High_Male - Medium_Male; Low_Female - Low_Male; Low_Female - Medium_Female; Low_Female - Medium_Male; Low_Male - Medium_Female; Low_Male - Medium_Male; Medium_Female - Medium_Male	A_21_P0000836	NR_038912	NR_038912

359	AMPD3	adenosine monophosphate deaminase 3	2.8099	0.025705	1.59	0.35744	High_Male - High_Female; Low_Female - High_Female; Low_Male - High_Female; Medium_Female - High_Female; High_Female - Medium_Male; High_Male - Low_Female; High_Male - Low_Male; High_Male - Medium_Female; High_Male - Medium_Male; Low_Female - Low_Male; Medium_Female - Low_Female; Low_Female - Medium_Male; Medium_Female - Low_Male; Low_Male - Medium_Male; Medium_Female - Medium_Male	A_33_P3216938		EF537581
360			2.809	0.025741	1.5894	0.35744	High_Male - High_Female; Low_Female - High_Female; Low_Male - High_Female; Medium_Female - High_Female; Medium_Male - High_Female; Low_Female - High_Male; Low_Male - High_Male; High_Male - Medium_Female; High_Male - Medium_Male; Low_Female - Low_Male; Low_Female - Medium_Female; Low_Female - Medium_Male; Low_Male - Medium_Female; Low_Male - Medium_Male; Medium_Female - Medium_Male	A_33_P3277096		AJ315539

361	OLIG1	oligodendrocyte transcription factor 1	2.8067	0.025837	1.5878	0.35779	High_Male - High_Female; High_Female - Low_Female; High_Female - Low_Male; Medium_Female - High_Female; Medium_Male - High_Female; High_Male - Low_Female; High_Male - Low_Male; High_Male - Medium_Female; Medium_Male - High_Male; Low_Male - Low_Female; Medium_Female - Low_Female; Medium_Male - Low_Female; Medium_Female - Low_Male; Medium_Male - Low_Male; Medium_Male - Medium_Female	A_23_P154849	NM_138983	NM_138983
362	XLOC_008221		2.7995	0.026137	1.5828	0.35941	High_Male - High_Female; Low_Female - High_Female; Low_Male - High_Female; Medium_Female - High_Female; Medium_Male - High_Female; High_Male - Low_Female; High_Male - Low_Male; High_Male - Medium_Female; Medium_Male - High_Male; Low_Male - Low_Female; Medium_Female - Low_Female; Medium_Male - Low_Female; Low_Male - Medium_Female; Medium_Male - Low_Male; Medium_Male - Medium_Female	A_21_P0006461		BX331476

363	XLOC_008609		2.7991	0.026153	1.5825	0.35941	High_Male - High_Female;<br>Low_Female - High_Female;<br>Low_Male - High_Female;<br>Medium_Female - High_Female;<br>Medium_Male - High_Female;<br>Low_Female - High_Male;<br>Low_Male - High_Male;<br>High_Male - Medium_Female;<br>High_Male - Medium_Male;<br>Low_Female - Low_Male;<br>Low_Female - Medium_Female;<br>Low_Female - Medium_Male;<br>Low_Male - Medium_Female;<br>Low_Male - Medium_Male;<br>Medium_Female - Medium_Male	linc|TCONS_00018331		
364	XLOC_002263		2.7987	0.02617	1.5822	0.35941	High_Male - High_Female;<br>Low_Female - High_Female;<br>Low_Male - High_Female;<br>Medium_Female - High_Female;<br>Medium_Male - High_Female;<br>High_Male - Low_Female;<br>High_Male - Low_Male;<br>Medium_Female - High_Male;<br>High_Male - Medium_Male;<br>Low_Female - Low_Male;<br>Medium_Female - Low_Female;<br>Low_Female - Medium_Male;<br>Medium_Female - Low_Male;<br>Low_Male - Medium_Male;<br>Medium_Female - Medium_Male	linc|TCONS_00004378		

365	XLOC_007974		2.7969	0.026248	1.5809	0.35949	High_Male - High_Female; Low_Female - High_Female; Low_Male - High_Female; Medium_Female - High_Female; High_Female - Medium_Male; Low_Female - High_Male; High_Male - Low_Male; Medium_Female - High_Male; High_Male - Medium_Male; Low_Female - Low_Male; Low_Female - Medium_Female; Low_Female - Medium_Male; Medium_Female - Low_Male; Low_Male - Medium_Male; Medium_Female - Medium_Male	linc|TCONS_00017163		
366	HMGCLL1	3-hydroxymethyl-3-methylglutaryl-CoA lyase-like 1	2.7938	0.026377	1.5788	0.3598	High_Male - High_Female; Low_Female - High_Female; Low_Male - High_Female; Medium_Female - High_Female; High_Female - Medium_Male; Low_Female - High_Male; High_Male - Low_Male; Medium_Female - High_Male; High_Male - Medium_Male; Low_Female - Low_Male; Low_Female - Medium_Female; Low_Female - Medium_Male; Medium_Female - Low_Male; Low_Male - Medium_Male; Medium_Female - Medium_Male	A_33_P3214012	NM_019036	NM_019036

367	ACCSL	1-aminocyclopropane-1-carboxylate synthase homolog (Arabidopsis)(non-functional)-like	2.7914	0.026481	1.5771	0.3598	High_Male - High_Female; Low_Female - High_Female; Low_Male - High_Female; Medium_Female - High_Female; Medium_Male - High_Female; Low_Female - High_Male; High_Male - Low_Male; High_Male - Medium_Female; High_Male - Medium_Male; Low_Female - Low_Male; Low_Female - Medium_Female; Low_Female - Medium_Male; Low_Male - Medium_Female; Low_Male - Medium_Male; Medium_Female - Medium_Male	A_33_P3255924	NM_001031854	NM_001031854
368			2.7913	0.026487	1.577	0.3598	High_Male - High_Female; Low_Female - High_Female; Low_Male - High_Female; Medium_Female - High_Female; Medium_Male - High_Female; Low_Female - High_Male; Low_Male - High_Male; High_Male - Medium_Female; High_Male - Medium_Male; Low_Male - Low_Female; Low_Female - Medium_Female; Low_Female - Medium_Male; Low_Male - Medium_Female; Low_Male - Medium_Male; Medium_Female - Medium_Male	A_33_P3332627		

369	XLOC_011719		2.7854	0.026736	1.5729	0.3622	High_Male - High_Female; Low_Female - High_Female; Low_Male - High_Female; Medium_Female - High_Female; Medium_Male - High_Female; Low_Female - High_Male; Low_Male - High_Male; Medium_Female - High_Male; High_Male - Medium_Male; Low_Male - Low_Female; Low_Female - Medium_Female; Low_Female - Medium_Male; Low_Male - Medium_Female; Low_Male - Medium_Male; Medium_Female - Medium_Male	tc|THC2675915|tc|THC2693028|linc|TCONS_00024395|linc|TCONS_00024396		
370	KIR2DL5A	killer cell immunoglobulin-like receptor, two domains, long cytoplasmic tail, 5A	2.7813	0.026913	1.57	0.36362	High_Male - High_Female; High_Female - Low_Female; Low_Male - High_Female; Medium_Female - High_Female; Medium_Male - High_Female; High_Male - Low_Female; Low_Male - High_Male; Medium_Female - High_Male; High_Male - Medium_Male; Low_Male - Low_Female; Medium_Female - Low_Female; Medium_Male - Low_Female; Low_Male - Medium_Female; Low_Male - Medium_Male; Medium_Female - Medium_Male	A_33_P3270346	NM_020535	NM_020535

371	GSTT1	glutathione S-transferase theta 1	2.7772	0.027091	1.5672	0.36503	High_Female - High_Male;<br>High_Female - Low_Female;<br>Low_Male - High_Female;<br>High_Female - Medium_Female;<br>High_Female - Medium_Male;<br>Low_Female - High_Male;<br>Low_Male - High_Male;<br>High_Male - Medium_Female;<br>Medium_Male - High_Male;<br>Low_Male - Low_Female;<br>Low_Female - Medium_Female;<br>Low_Female - Medium_Male;<br>Low_Male - Medium_Female;<br>Low_Male - Medium_Male;<br>Medium_Male - Medium_Female	A_23_P254944	NM_000853	NM_000853
372			2.7751	0.027186	1.5657	0.36533	High_Female - High_Male;<br>High_Female - Low_Female;<br>High_Female - Low_Male;<br>Medium_Female - High_Female;<br>Medium_Male - High_Female;<br>Low_Female - High_Male;<br>Low_Male - High_Male;<br>Medium_Female - High_Male;<br>Medium_Male - High_Male;<br>Low_Female - Low_Male;<br>Medium_Female - Low_Female;<br>Medium_Male - Low_Female;<br>Medium_Female - Low_Male;<br>Medium_Male - Low_Male;<br>Medium_Female - Medium_Male	A_24_P64100		AF495725

373	LOC645638	WDNM1-like pseudogene	2.7687	0.027465	1.5612	0.36757	High_Male - High_Female;<br>Low_Female - High_Female;<br>Low_Male - High_Female;<br>High_Female - Medium_Female;<br>Medium_Male - High_Female;<br>High_Male - Low_Female;<br>High_Male - Low_Male;<br>High_Male - Medium_Female;<br>Medium_Male - High_Male;<br>Low_Male - Low_Female;<br>Low_Female - Medium_Female;<br>Medium_Male - Low_Female;<br>Low_Male - Medium_Female;<br>Medium_Male - Low_Male;<br>Medium_Male - Medium_Female	A_21_P0009342	NR_030732	NR_030732
374	RAB2B	RAB2B, member RAS oncogene family	2.7679	0.027499	1.5607	0.36757	High_Female - High_Male;<br>Low_Female - High_Female;<br>High_Female - Low_Male;<br>Medium_Female - High_Female;<br>High_Female - Medium_Male;<br>Low_Female - High_Male;<br>Low_Male - High_Male;<br>Medium_Female - High_Male;<br>Medium_Male - High_Male;<br>Low_Female - Low_Male;<br>Medium_Female - Low_Female;<br>Low_Female - Medium_Male;<br>Medium_Female - Low_Male;<br>Low_Male - Medium_Male;<br>Medium_Female - Medium_Male	A_33_P3295803	NM_032846	NM_032846

375	KREMEN1	kringle containing transmembrane protein 1	2.7555	0.028055	1.552	0.37399	High_Female - High_Male; High_Female - Low_Female; High_Female - Low_Male; High_Female - Medium_Female; High_Female - Medium_Male; Low_Female - High_Male; High_Male - Low_Male; Medium_Female - High_Male; High_Male - Medium_Male; Low_Female - Low_Male; Medium_Female - Low_Female; Low_Female - Medium_Male; Medium_Female - Low_Male; Medium_Male - Low_Male; Medium_Female - Medium_Male	A_23_P68851	NM_001039570	NM_001039570
376	FCGR1B	Fc fragment of IgG, high affinity Ib, receptor (CD64)	2.751	0.028258	1.5489	0.3757	High_Male - High_Female; Low_Female - High_Female; High_Female - Low_Male; High_Female - Medium_Female; High_Female - Medium_Male; Low_Female - High_Male; High_Male - Low_Male; High_Male - Medium_Female; High_Male - Medium_Male; Low_Female - Low_Male; Low_Female - Medium_Female; Low_Female - Medium_Male; Medium_Female - Low_Male; Medium_Male - Low_Male; Medium_Female - Medium_Male	A_21_P0010561	NM_001244910	NM_001244910

377	HOTAIR		2.7487	0.028366	1.5472	0.37613	High_Male - High_Female; Low_Female - High_Female; Low_Male - High_Female; Medium_Female - High_Female; Medium_Male - High_Female; Low_Female - High_Male; Low_Male - High_Male; High_Male - Medium_Female; High_Male - Medium_Male; Low_Female - Low_Male; Low_Female - Medium_Female; Low_Female - Medium_Male; Low_Male - Medium_Female; Low_Male - Medium_Male; Medium_Female - Medium_Male	A_19_P00330464	NR_003716	NR_003716
378	GDF5	growth differentiation factor 5	2.7459	0.028493	1.5453	0.37682	High_Male - High_Female; Low_Female - High_Female; Low_Male - High_Female; Medium_Female - High_Female; Medium_Male - High_Female; Low_Female - High_Male; Low_Male - High_Male; High_Male - Medium_Female; High_Male - Medium_Male; Low_Male - Low_Female; Low_Female - Medium_Female; Low_Female - Medium_Male; Low_Male - Medium_Female; Low_Male - Medium_Male; Medium_Female - Medium_Male	A_23_P259955	NM_000557	NM_000557

379	LOC10013361 2	uncharacterized LOC100133612	2.7436	0.0286	1.5436	0.37687	High_Male - High_Female; Low_Female - High_Female; Low_Male - High_Female; Medium_Female - High_Female; Medium_Male - High_Female; Low_Female - High_Male; Low_Male - High_Male; High_Male - Medium_Female; High_Male - Medium_Male; Low_Female - Low_Male; Low_Female - Medium_Female; Low_Female - Medium_Male; Low_Male - Medium_Female; Low_Male - Medium_Male; Medium_Female - Medium_Male	A_21_P0010510		AK130173
380	C5orf38	chromosome 5 open reading frame 38	2.7425	0.028648	1.5429	0.37687	High_Male - High_Female; Low_Female - High_Female; High_Female - Low_Male; High_Female - Medium_Female; High_Female - Medium_Male; Low_Female - High_Male; High_Male - Low_Male; High_Male - Medium_Female; High_Male - Medium_Male; Low_Female - Low_Male; Low_Female - Medium_Female; Low_Female - Medium_Male; Low_Male - Medium_Female; Low_Male - Medium_Male; Medium_Female - Medium_Male	A_23_P331235	NM_178569	NM_178569

381	XLOC_005780		2.7384	0.028838	1.54	0.37837	High_Female - High_Male; High_Female - Low_Female; High_Female - Low_Male; High_Female - Medium_Female; High_Female - Medium_Male; Low_Female - High_Male; Low_Male - High_Male; High_Male - Medium_Female; Medium_Male - High_Male; Low_Female - Low_Male; Low_Female - Medium_Female; Low_Female - Medium_Male; Low_Male - Medium_Female; Medium_Male - Low_Male; Medium_Male - Medium_Female	linc|TCONS_00012221		
382	XLOC_l2_015212		2.7348	0.029005	1.5375	0.37958	High_Female - High_Male; High_Female - Low_Female; High_Female - Low_Male; High_Female - Medium_Female; High_Female - Medium_Male; High_Male - Low_Female; High_Male - Low_Male; High_Male - Medium_Female; Medium_Male - High_Male; Low_Female - Low_Male; Low_Female - Medium_Female; Medium_Male - Low_Female; Medium_Female - Low_Male; Medium_Male - Low_Male; Medium_Male - Medium_Female	linc|TCONS_l2_00029343		

383	TTTY10	testis-specific transcript, Y-linked 10 (non-protein coding)	2.7321	0.02913	1.5357	0.37988	High_Male - High_Female; Low_Female - High_Female; Low_Male - High_Female; Medium_Female - High_Female; Medium_Male - High_Female; High_Male - Low_Female; Low_Male - High_Male; Medium_Female - High_Male; Medium_Male - High_Male; Low_Male - Low_Female; Medium_Female - Low_Female; Medium_Male - Low_Female; Low_Male - Medium_Female; Medium_Male - Low_Male; Medium_Male - Medium_Female	A_23_P320622	NR_001542	NR_001542
384	XLOC_004375		2.7311	0.029181	1.5349	0.37988	High_Male - High_Female; Low_Female - High_Female; Low_Male - High_Female; Medium_Female - High_Female; Medium_Male - High_Female; High_Male - Low_Female; High_Male - Low_Male; High_Male - Medium_Female; High_Male - Medium_Male; Low_Male - Low_Female; Low_Female - Medium_Female; Low_Female - Medium_Male; Low_Male - Medium_Female; Low_Male - Medium_Male; Medium_Female - Medium_Male	linc|TCONS_00009956		

385			2.7283	0.029309	1.533	0.3799	High_Male - High_Female; Low_Female - High_Female; Low_Male - High_Female; Medium_Female - High_Female; Medium_Male - High_Female; Low_Female - High_Male; Low_Male - High_Male; Medium_Female - High_Male; High_Male - Medium_Male; Low_Male - Low_Female; Low_Female - Medium_Female; Low_Female - Medium_Male; Low_Male - Medium_Female; Low_Male - Medium_Male; Medium_Female - Medium_Male	A_33_P3252181		AK097351
386	NPRL3	nitrogen permease regulator-like 3 (S. cerevisiae)	2.7278	0.029334	1.5326	0.3799	High_Female - High_Male; Low_Female - High_Female; Low_Male - High_Female; Medium_Female - High_Female; Medium_Male - High_Female; Low_Female - High_Male; Low_Male - High_Male; Medium_Female - High_Male; Medium_Male - High_Male; Low_Female - Low_Male; Medium_Female - Low_Female; Low_Female - Medium_Male; Medium_Female - Low_Male; Medium_Male - Low_Male; Medium_Female - Medium_Male	A_23_P100326	NM_001039476	NM_001039476

387	SLC26A8	solute carrier family 26, member 8	2.7259	0.029424	1.5313	0.38008	High_Female - High_Male; High_Female - Low_Female; High_Female - Low_Male; High_Female - Medium_Female; High_Female - Medium_Male; High_Male - Low_Female; High_Male - Low_Male; Medium_Female - High_Male; Medium_Male - High_Male; Low_Female - Low_Male; Medium_Female - Low_Female; Medium_Male - Low_Female; Medium_Female - Low_Male; Medium_Male - Low_Male; Medium_Male - Medium_Female	A_23_P30950	NM_052961	NM_052961
388	LOC100128262	uncharacterized LOC100128262	2.7209	0.029661	1.5278	0.38052	High_Male - High_Female; Low_Female - High_Female; Low_Male - High_Female; Medium_Female - High_Female; Medium_Male - High_Female; Low_Female - High_Male; Low_Male - High_Male; Medium_Female - High_Male; Medium_Male - High_Male; Low_Female - Low_Male; Medium_Female - Low_Female; Medium_Male - Low_Female; Medium_Female - Low_Male; Medium_Male - Low_Male; Medium_Female - Medium_Male	A_21_P0014586	XR_110396	XR_110396

389	XLOC_004710		2.7196	0.029725	1.5269	0.38052	High_Male - High_Female; Low_Female - High_Female; Low_Male - High_Female; Medium_Female - High_Female; Medium_Male - High_Female; Low_Female - High_Male; Low_Male - High_Male; High_Male - Medium_Female; High_Male - Medium_Male; Low_Female - Low_Male; Low_Female - Medium_Female; Low_Female - Medium_Male; Low_Male - Medium_Female; Low_Male - Medium_Male; Medium_Female - Medium_Male	linc|TCONS_00010248		
390	FAM129C	family with sequence similarity 129, member C	2.718	0.029801	1.5258	0.38052	High_Male - High_Female; Low_Female - High_Female; Low_Male - High_Female; Medium_Female - High_Female; Medium_Male - High_Female; Low_Female - High_Male; Low_Male - High_Male; Medium_Female - High_Male; Medium_Male - High_Male; Low_Male - Low_Female; Low_Female - Medium_Female; Medium_Male - Low_Female; Low_Male - Medium_Female; Medium_Male - Low_Male; Medium_Male - Medium_Female	A_33_P3350259	NM_001098524	NM_001098524

391	LOC100289580	uncharacterized LOC100289580	2.7173	0.029837	1.5253	0.38052	High_Male - High_Female; Low_Female - High_Female; Low_Male - High_Female; Medium_Female - High_Female; Medium_Male - High_Female; Low_Female - High_Male; Low_Male - High_Male; High_Male - Medium_Female; High_Male - Medium_Male; Low_Male - Low_Female; Low_Female - Medium_Female; Low_Female - Medium_Male; Low_Male - Medium_Female; Low_Male - Medium_Male; Medium_Female - Medium_Male	A_33_P3419621		AK127064
392	CDC42EP1	CDC42 effector protein (Rho GTPase binding) 1	2.7172	0.029839	1.5252	0.38052	High_Male - High_Female; Low_Female - High_Female; Low_Male - High_Female; Medium_Female - High_Female; Medium_Male - High_Female; High_Male - Low_Female; Low_Male - High_Male; Medium_Female - High_Male; Medium_Male - High_Male; Low_Male - Low_Female; Medium_Female - Low_Female; Medium_Male - Low_Female; Low_Male - Medium_Female; Low_Male - Medium_Male; Medium_Female - Medium_Male	A_33_P3407424	NM_152243	NM_152243

393	PRR5	proline rich 5 (renal)	2.709	0.030234	1.5195	0.38458	High_Male - High_Female; Low_Female - High_Female; Low_Male - High_Female; Medium_Female - High_Female; Medium_Male - High_Female; Low_Female - High_Male; Low_Male - High_Male; High_Male - Medium_Female; High_Male - Medium_Male; Low_Male - Low_Female; Low_Female - Medium_Female; Low_Female - Medium_Male; Low_Male - Medium_Female; Low_Male - Medium_Male; Medium_Female - Medium_Male	A_23_P80382	NM_015366	NM_015366
394	XLOC_013981		2.7052	0.030421	1.5168	0.38551	High_Male - High_Female; Low_Female - High_Female; Low_Male - High_Female; Medium_Female - High_Female; Medium_Male - High_Female; Low_Female - High_Male; Low_Male - High_Male; High_Male - Medium_Female; High_Male - Medium_Male; Low_Male - Low_Female; Low_Female - Medium_Female; Low_Female - Medium_Male; Low_Male - Medium_Female; Low_Male - Medium_Male; Medium_Female - Medium_Male	linc|TCONS_00029251|linc|TCONS_00029061		

395	XLOC_l2_002886		2.7044	0.030461	1.5162	0.38551	High_Male - High_Female; Low_Female - High_Female; Low_Male - High_Female; Medium_Female - High_Female; Medium_Male - High_Female; Low_Female - High_Male; Low_Male - High_Male; Medium_Female - High_Male; High_Male - Medium_Male; Low_Male - Low_Female; Low_Female - Medium_Female; Low_Female - Medium_Male; Low_Male - Medium_Female; Low_Male - Medium_Male; Medium_Female - Medium_Male	tc|THC2553586|linc|TCONS_l2_00005503		
396	XLOC_003839		2.7025	0.030554	1.5149	0.38571	High_Female - High_Male; High_Female - Low_Female; High_Female - Low_Male; High_Female - Medium_Female; High_Female - Medium_Male; Low_Female - High_Male; Low_Male - High_Male; Medium_Female - High_Male; Medium_Male - High_Male; Low_Female - Low_Male; Low_Female - Medium_Female; Medium_Male - Low_Female; Medium_Female - Low_Male; Medium_Male - Low_Male; Medium_Male - Medium_Female	tc|THC2771827|linc|TCONS_00008387|linc|TCONS_00008983|linc|TCONS_00008982		

397	LOC100287633	protein FAM27E2-like	2.6973	0.030812	1.5113	0.38798	High_Male - High_Female; Low_Female - High_Female; Low_Male - High_Female; Medium_Female - High_Female; Medium_Male - High_Female; Low_Female - High_Male; Low_Male - High_Male; High_Male - Medium_Female; High_Male - Medium_Male; Low_Female - Low_Male; Low_Female - Medium_Female; Low_Female - Medium_Male; Low_Male - Medium_Female; Low_Male - Medium_Male; Medium_Female - Medium_Male	A_21_P0013934	XM_002342728	XM_002342728
398	TACR2	tachykinin receptor 2	2.6954	0.030907	1.5099	0.38821	High_Male - High_Female; Low_Female - High_Female; Low_Male - High_Female; Medium_Female - High_Female; High_Female - Medium_Male; Low_Female - High_Male; Low_Male - High_Male; Medium_Female - High_Male; High_Male - Medium_Male; Low_Male - Low_Female; Medium_Female - Low_Female; Low_Female - Medium_Male; Low_Male - Medium_Female; Low_Male - Medium_Male; Medium_Female - Medium_Male	A_23_P24135	NM_001057	NM_001057

399	MAP7	microtubule-associated protein 7	2.6889	0.031231	1.5054	0.3899	High_Male - High_Female; Low_Female - High_Female; Low_Male - High_Female; High_Female - Medium_Female; High_Female - Medium_Male; High_Male - Low_Female; Low_Male - High_Male; High_Male - Medium_Female; High_Male - Medium_Male; Low_Male - Low_Female; Low_Female - Medium_Female; Low_Female - Medium_Male; Low_Male - Medium_Female; Low_Male - Medium_Male; Medium_Male - Medium_Female	A_33_P3375576	NM_003980	NM_003980
400	MAP7D2	MAP7 domain containing 2	2.6849	0.031431	1.5026	0.3899	High_Male - High_Female; Low_Female - High_Female; Low_Male - High_Female; Medium_Female - High_Female; High_Female - Medium_Male; Low_Female - High_Male; High_Male - Low_Male; Medium_Female - High_Male; High_Male - Medium_Male; Low_Female - Low_Male; Low_Female - Medium_Female; Low_Female - Medium_Male; Medium_Female - Low_Male; Low_Male - Medium_Male; Medium_Female - Medium_Male	A_24_P7652	NM_152780	NM_152780

401	LOC92659	uncharacterized LOC92659	2.6839	0.031485	1.5019	0.3899	High_Male - High_Female; Low_Female - High_Female; Low_Male - High_Female; Medium_Female - High_Female; High_Female - Medium_Male; High_Male - Low_Female; Low_Male - High_Male; High_Male - Medium_Female; High_Male - Medium_Male; Low_Male - Low_Female; Medium_Female - Low_Female; Low_Female - Medium_Male; Low_Male - Medium_Female; Low_Male - Medium_Male; Medium_Female - Medium_Male	A_33_P3238543	NR_015454	NR_015454
402	XLOC_006593		2.6832	0.031519	1.5014	0.3899	High_Male - High_Female; Low_Female - High_Female; Low_Male - High_Female; Medium_Female - High_Female; Medium_Male - High_Female; Low_Female - High_Male; Low_Male - High_Male; High_Male - Medium_Female; High_Male - Medium_Male; Low_Female - Low_Male; Low_Female - Medium_Female; Low_Female - Medium_Male; Low_Male - Medium_Female; Low_Male - Medium_Male; Medium_Female - Medium_Male	A_21_P0005332		BG185100

403			2.6829	0.031535	1.5012	0.3899	High_Male - High_Female;<br>Low_Female - High_Female;<br>Low_Male - High_Female;<br>Medium_Female - High_Female;<br>Medium_Male - High_Female;<br>Low_Female - High_Male;<br>Low_Male - High_Male;<br>High_Male - Medium_Female;<br>High_Male - Medium_Male;<br>Low_Male - Low_Female;<br>Low_Female - Medium_Female;<br>Low_Female - Medium_Male;<br>Low_Male - Medium_Female;<br>Low_Male - Medium_Male;<br>Medium_Female - Medium_Male	A_19_P00319372		XR_110395
404	SH2D2A	SH2 domain containing 2A	2.6823	0.031565	1.5008	0.3899	High_Male - High_Female;<br>Low_Female - High_Female;<br>Low_Male - High_Female;<br>High_Female - Medium_Female;<br>Medium_Male - High_Female;<br>High_Male - Low_Female;<br>Low_Male - High_Male;<br>High_Male - Medium_Female;<br>Medium_Male - High_Male;<br>Low_Male - Low_Female;<br>Low_Female - Medium_Female;<br>Medium_Male - Low_Female;<br>Low_Male - Medium_Female;<br>Low_Male - Medium_Male;<br>Medium_Male - Medium_Female	A_21_P0000028	NM_001161444	NM_001161444

405	SYNGR1	synaptogyrin 1	2.6818	0.031588	1.5005	0.3899	High_Male - High_Female; Low_Female - High_Female; Low_Male - High_Female; Medium_Female - High_Female; Medium_Male - High_Female; Low_Female - High_Male; High_Male - Low_Male; Medium_Female - High_Male; High_Male - Medium_Male; Low_Female - Low_Male; Low_Female - Medium_Female; Low_Female - Medium_Male; Medium_Female - Low_Male; Low_Male - Medium_Male; Medium_Female - Medium_Male	A_23_P348063	NM_004711	NM_004711
406	E2F2	E2F transcription factor 2	2.676	0.031888	1.4964	0.39263	High_Female - High_Male; High_Female - Low_Female; Low_Male - High_Female; Medium_Female - High_Female; High_Female - Medium_Male; Low_Female - High_Male; Low_Male - High_Male; Medium_Female - High_Male; Medium_Male - High_Male; Low_Male - Low_Female; Medium_Female - Low_Female; Medium_Male - Low_Female; Medium_Female - Low_Male; Low_Male - Medium_Male; Medium_Female - Medium_Male	A_23_P408955	NM_004091	NM_004091

407	REREP3	arginine-glutamic acid dipeptide (RE) repeats pseudogene 3	2.6687	0.032264	1.4913	0.39559	High_Male - High_Female; Low_Female - High_Female; Low_Male - High_Female; Medium_Female - High_Female; Medium_Male - High_Female; Low_Female - High_Male; Low_Male - High_Male; Medium_Female - High_Male; High_Male - Medium_Male; Low_Female - Low_Male; Low_Female - Medium_Female; Low_Female - Medium_Male; Low_Male - Medium_Female; Low_Male - Medium_Male; Medium_Female - Medium_Male	A_33_P3259861	NR_033735	NR_033735
408	OBSCN	obscurin, cytoskeletal calmodulin and titin-interacting RhoGEF	2.6682	0.032287	1.491	0.39559	High_Male - High_Female; Low_Female - High_Female; Low_Male - High_Female; Medium_Female - High_Female; Medium_Male - High_Female; Low_Female - High_Male; Low_Male - High_Male; High_Male - Medium_Female; High_Male - Medium_Male; Low_Male - Low_Female; Low_Female - Medium_Female; Low_Female - Medium_Male; Low_Male - Medium_Female; Low_Male - Medium_Male; Medium_Female - Medium_Male	A_24_P119685	NM_052843	NM_052843

409			2.6663	0.032387	1.4896	0.39584	High_Male - High_Female;<br>Low_Female - High_Female;<br>Low_Male - High_Female;<br>Medium_Female - High_Female;<br>Medium_Male - High_Female;<br>Low_Female - High_Male;<br>Low_Male - High_Male;<br>Medium_Female - High_Male;<br>High_Male - Medium_Male;<br>Low_Male - Low_Female;<br>Low_Female - Medium_Female;<br>Low_Female - Medium_Male;<br>Low_Male - Medium_Female;<br>Low_Male - Medium_Male;<br>Medium_Female - Medium_Male	A_33_P3291329		
410	ACACB	acetyl-CoA carboxylase beta	2.6623	0.032598	1.4868	0.39672	High_Male - High_Female;<br>Low_Female - High_Female;<br>Low_Male - High_Female;<br>Medium_Female - High_Female;<br>Medium_Male - High_Female;<br>High_Male - Low_Female;<br>High_Male - Low_Male;<br>High_Male - Medium_Female;<br>Medium_Male - High_Male;<br>Low_Male - Low_Female;<br>Medium_Female - Low_Female;<br>Medium_Male - Low_Female;<br>Low_Male - Medium_Female;<br>Medium_Male - Low_Male;<br>Medium_Male - Medium_Female	A_33_P3334220	NM_001093	NM_001093

411			2.6606	0.032685	1.4857	0.39672	High_Male - High_Female;<br>Low_Female - High_Female;<br>Low_Male - High_Female;<br>Medium_Female - High_Female;<br>Medium_Male - High_Female;<br>Low_Female - High_Male;<br>Low_Male - High_Male;<br>Medium_Female - High_Male;<br>High_Male - Medium_Male;<br>Low_Female - Low_Male;<br>Low_Female - Medium_Female;<br>Low_Female - Medium_Male;<br>Medium_Female - Low_Male;<br>Low_Male - Medium_Male;<br>Medium_Female - Medium_Male	A_33_P3392927		AB529253
412	CST2	cystatin SA	2.6595	0.032747	1.4848	0.39672	High_Male - High_Female;<br>Low_Female - High_Female;<br>Low_Male - High_Female;<br>Medium_Female - High_Female;<br>High_Female - Medium_Male;<br>Low_Female - High_Male;<br>Low_Male - High_Male;<br>Medium_Female - High_Male;<br>High_Male - Medium_Male;<br>Low_Female - Low_Male;<br>Low_Female - Medium_Female;<br>Low_Female - Medium_Male;<br>Medium_Female - Low_Male;<br>Low_Male - Medium_Male;<br>Medium_Female - Medium_Male	A_24_P237175	NM_001322	NM_001322

413	XLOC_009389		2.6553	0.032966	1.4819	0.39672	High_Female - High_Male; Low_Female - High_Female; High_Female - Low_Male; Medium_Female - High_Female; High_Female - Medium_Male; Low_Female - High_Male; Low_Male - High_Male; Medium_Female - High_Male; Medium_Male - High_Male; Low_Female - Low_Male; Medium_Female - Low_Female; Low_Female - Medium_Male; Medium_Female - Low_Male; Medium_Male - Low_Male; Medium_Female - Medium_Male	tc|THC2788642|linc|TCONS_00019588		
414			2.655	0.032981	1.4817	0.39672	High_Female - High_Male; High_Female - Low_Female; Low_Male - High_Female; Medium_Female - High_Female; High_Female - Medium_Male; Low_Female - High_Male; Low_Male - High_Male; Medium_Female - High_Male; High_Male - Medium_Male; Low_Male - Low_Female; Medium_Female - Low_Female; Low_Female - Medium_Male; Medium_Female - Low_Male; Low_Male - Medium_Male; Medium_Female - Medium_Male	A_33_P3250750		CU677518

415	LOC100128252	uncharacterized LOC100128252	2.6549	0.032987	1.4817	0.39672	High_Male - High_Female;<br>Low_Female - High_Female;<br>Low_Male - High_Female;<br>Medium_Female - High_Female;<br>Medium_Male - High_Female;<br>Low_Female - High_Male;<br>Low_Male - High_Male;<br>Medium_Female - High_Male;<br>Medium_Male - High_Male;<br>Low_Male - Low_Female;<br>Medium_Female - Low_Female;<br>Medium_Male - Low_Female;<br>Low_Male - Medium_Female;<br>Medium_Male - Low_Male;<br>Medium_Male - Medium_Female	A_33_P3259973	NR_036522	NR_036522
416	SYCE3	synaptonemal complex central element protein 3	2.6543	0.033022	1.4812	0.39672	High_Female - High_Male;<br>High_Female - Low_Female;<br>High_Female - Low_Male;<br>High_Female - Medium_Female;<br>High_Female - Medium_Male;<br>Low_Female - High_Male;<br>High_Male - Low_Male;<br>High_Male - Medium_Female;<br>High_Male - Medium_Male;<br>Low_Female - Low_Male;<br>Low_Female - Medium_Female;<br>Low_Female - Medium_Male;<br>Low_Male - Medium_Female;<br>Low_Male - Medium_Male;<br>Medium_Female - Medium_Male	A_33_P3250887	NM_001123225	NM_001123225

417	CCL3	chemokine (C-C motif) ligand 3	2.6529	0.033093	1.4803	0.39672	High_Male - High_Female; Low_Female - High_Female; Low_Male - High_Female; Medium_Female - High_Female; Medium_Male - High_Female; Low_Female - High_Male; Low_Male - High_Male; High_Male - Medium_Female; High_Male - Medium_Male; Low_Male - Low_Female; Low_Female - Medium_Female; Low_Female - Medium_Male; Low_Male - Medium_Female; Low_Male - Medium_Male; Medium_Female - Medium_Male	A_33_P3316273	NM_002983	NM_002983
418	NEAT1	nuclear paraspeckle assembly transcript 1 (non-protein coding)	2.6411	0.033729	1.472	0.40338	High_Female - High_Male; Low_Female - High_Female; Low_Male - High_Female; High_Female - Medium_Female; Medium_Male - High_Female; Low_Female - High_Male; Low_Male - High_Male; Medium_Female - High_Male; Medium_Male - High_Male; Low_Male - Low_Female; Low_Female - Medium_Female; Low_Female - Medium_Male; Low_Male - Medium_Female; Low_Male - Medium_Male; Medium_Male - Medium_Female	A_33_P3263538		AF001893

419	GBP3	guanylate binding protein 3	2.6376	0.033919	1.4696	0.40468	High_Male - High_Female;<br>High_Female - Low_Female;<br>High_Female - Low_Male;<br>High_Female - Medium_Female;<br>High_Female - Medium_Male;<br>High_Male - Low_Female;<br>High_Male - Low_Male;<br>High_Male - Medium_Female;<br>High_Male - Medium_Male;<br>Low_Female - Low_Male;<br>Low_Female - Medium_Female;<br>Low_Female - Medium_Male;<br>Medium_Female - Low_Male;<br>Medium_Male - Low_Male;<br>Medium_Male - Medium_Female	A_23_P51487	NM_018284	NM_018284
420	KRTAP4-12	keratin associated protein 4-12	2.6359	0.034014	1.4683	0.40485	High_Male - High_Female;<br>Low_Female - High_Female;<br>Low_Male - High_Female;<br>Medium_Female - High_Female;<br>Medium_Male - High_Female;<br>Low_Female - High_Male;<br>Low_Male - High_Male;<br>High_Male - Medium_Female;<br>High_Male - Medium_Male;<br>Low_Female - Low_Male;<br>Low_Female - Medium_Female;<br>Low_Female - Medium_Male;<br>Low_Male - Medium_Female;<br>Low_Male - Medium_Male;<br>Medium_Female - Medium_Male	A_23_P15798	NM_031854	NM_031854

421	XLOC_012571		2.6343	0.034102	1.4672	0.40493	High_Male - High_Female; Low_Female - High_Female; Low_Male - High_Female; High_Female - Medium_Female; High_Female - Medium_Male; Low_Female - High_Male; High_Male - Low_Male; High_Male - Medium_Female; High_Male - Medium_Male; Low_Female - Low_Male; Low_Female - Medium_Female; Low_Female - Medium_Male; Low_Male - Medium_Female; Low_Male - Medium_Male; Medium_Female - Medium_Male	linc|TCONS_00025761		
422	TMEM176B	transmembrane protein 176B	2.6318	0.034238	1.4655	0.40558	High_Male - High_Female; Low_Female - High_Female; Low_Male - High_Female; Medium_Female - High_Female; Medium_Male - High_Female; High_Male - Low_Female; High_Male - Low_Male; Medium_Female - High_Male; High_Male - Medium_Male; Low_Male - Low_Female; Medium_Female - Low_Female; Medium_Male - Low_Female; Medium_Female - Low_Male; Low_Male - Medium_Male; Medium_Female - Medium_Male	A_23_P157007	NM_014020	NM_014020

423	HLA-DRB6	major histocompatibility complex, class II, DR beta 6 (pseudogene)	2.6293	0.034379	1.4637	0.40628	High_Male - High_Female; Low_Female - High_Female; Low_Male - High_Female; Medium_Female - High_Female; Medium_Male - High_Female; High_Male - Low_Female; High_Male - Low_Male; High_Male - Medium_Female; Medium_Male - High_Male; Low_Female - Low_Male; Low_Female - Medium_Female; Medium_Male - Low_Female; Low_Male - Medium_Female; Medium_Male - Low_Male; Medium_Male - Medium_Female	A_24_P169013	NR_001298	NR_001298
424	LOC389906	zinc finger protein 839 pseudogene	2.625	0.034618	1.4607	0.40815	High_Female - High_Male; High_Female - Low_Female; High_Female - Low_Male; High_Female - Medium_Female; High_Female - Medium_Male; Low_Female - High_Male; High_Male - Low_Male; Medium_Female - High_Male; High_Male - Medium_Male; Low_Female - Low_Male; Medium_Female - Low_Female; Low_Female - Medium_Male; Medium_Female - Low_Male; Low_Male - Medium_Male; Medium_Female - Medium_Male	A_33_P3325229	NR_034031	NR_034031

425	XLOC_007868		2.6219	0.034792	1.4585	0.40923	High_Male - High_Female; Low_Female - High_Female; Low_Male - High_Female; Medium_Female - High_Female; Medium_Male - High_Female; Low_Female - High_Male; Low_Male - High_Male; High_Male - Medium_Female; High_Male - Medium_Male; Low_Male - Low_Female; Low_Female - Medium_Female; Low_Female - Medium_Male; Low_Male - Medium_Female; Low_Male - Medium_Male; Medium_Female - Medium_Male	linc|TCONS_00016491		
426	HEBP1	heme binding protein 1	2.6147	0.035192	1.4536	0.41193	High_Male - High_Female; Low_Female - High_Female; High_Female - Low_Male; Medium_Female - High_Female; Medium_Male - High_Female; High_Male - Low_Female; High_Male - Low_Male; Medium_Female - High_Male; Medium_Male - High_Male; Low_Female - Low_Male; Medium_Female - Low_Female; Medium_Male - Low_Female; Medium_Female - Low_Male; Medium_Male - Low_Male; Medium_Female - Medium_Male	A_23_P117082	NM_015987	NM_015987

427	TREML1	triggering receptor expressed on myeloid cells-like 1	2.6144	0.035211	1.4533	0.41193	High_Female - High_Male; High_Female - Low_Female; High_Female - Low_Male; Medium_Female - High_Female; High_Female - Medium_Male; Low_Female - High_Male; Low_Male - High_Male; Medium_Female - High_Male; Medium_Male - High_Male; Low_Female - Low_Male; Medium_Female - Low_Female; Low_Female - Medium_Male; Medium_Female - Low_Male; Medium_Male - Low_Male; Medium_Female - Medium_Male	A_33_P3381777	NM_178174	NM_178174
428	KIRREL	kin of IRRE like (Drosophila)	2.6114	0.035381	1.4512	0.41193	High_Male - High_Female; Low_Female - High_Female; Low_Male - High_Female; Medium_Female - High_Female; High_Female - Medium_Male; Low_Female - High_Male; High_Male - Low_Male; Medium_Female - High_Male; High_Male - Medium_Male; Low_Female - Low_Male; Medium_Female - Low_Female; Low_Female - Medium_Male; Medium_Female - Low_Male; Low_Male - Medium_Male; Medium_Female - Medium_Male	A_33_P3221489	NM_018240	NM_018240

429	XLOC_009000		2.6107	0.03542	1.4507	0.41193	High_Male - High_Female; Low_Female - High_Female; Low_Male - High_Female; Medium_Female - High_Female; High_Female - Medium_Male; Low_Female - High_Male; Low_Male - High_Male; High_Male - Medium_Female; High_Male - Medium_Male; Low_Female - Low_Male; Low_Female - Medium_Female; Low_Female - Medium_Male; Low_Male - Medium_Female; Low_Male - Medium_Male; Medium_Female - Medium_Male	linc|TCONS_00018649		
430	POU4F2	POU class 4 homeobox 2	2.6105	0.035433	1.4506	0.41193	High_Male - High_Female; Low_Female - High_Female; High_Female - Low_Male; Medium_Female - High_Female; High_Female - Medium_Male; Low_Female - High_Male; High_Male - Low_Male; High_Male - Medium_Female; High_Male - Medium_Male; Low_Female - Low_Male; Low_Female - Medium_Female; Low_Female - Medium_Male; Medium_Female - Low_Male; Medium_Male - Low_Male; Medium_Female - Medium_Male	A_23_P302038	NM_004575	NM_004575

431	LINC00482	long intergenic non-protein coding RNA 482	2.6087	0.035535	1.4493	0.41206	High_Female - High_Male; High_Female - Low_Female; High_Female - Low_Male; High_Female - Medium_Female; High_Female - Medium_Male; High_Male - Low_Female; High_Male - Low_Male; High_Male - Medium_Female; High_Male - Medium_Male; Low_Female - Low_Male; Low_Female - Medium_Female; Medium_Male - Low_Female; Medium_Female - Low_Male; Medium_Male - Low_Male; Medium_Male - Medium_Female	A_24_P323084	NR_038080	NR_038080
432	XLOC_004093		2.6074	0.035609	1.4484	0.41206	High_Male - High_Female; Low_Female - High_Female; Low_Male - High_Female; Medium_Female - High_Female; High_Female - Medium_Male; Low_Female - High_Male; Low_Male - High_Male; Medium_Female - High_Male; High_Male - Medium_Male; Low_Female - Low_Male; Low_Female - Medium_Female; Low_Female - Medium_Male; Low_Male - Medium_Female; Low_Male - Medium_Male; Medium_Female - Medium_Male	linc|TCONS_00008588		

433	XLOC_006756		2.6048	0.035759	1.4466	0.41284	High_Male - High_Female; Low_Female - High_Female; Low_Male - High_Female; Medium_Female - High_Female; Medium_Male - High_Female; High_Male - Low_Female; Low_Male - High_Male; Medium_Female - High_Male; High_Male - Medium_Male; Low_Male - Low_Female; Medium_Female - Low_Female; Low_Female - Medium_Male; Low_Male - Medium_Female; Low_Male - Medium_Male; Medium_Female - Medium_Male	A_19_P00315554		BC015784
434	GFRA1	GDNF family receptor alpha 1	2.602	0.035922	1.4446	0.41323	High_Male - High_Female; High_Female - Low_Female; High_Female - Low_Male; High_Female - Medium_Female; High_Female - Medium_Male; High_Male - Low_Female; High_Male - Low_Male; High_Male - Medium_Female; High_Male - Medium_Male; Low_Female - Low_Male; Low_Female - Medium_Female; Medium_Male - Low_Female; Medium_Female - Low_Male; Medium_Male - Low_Male; Medium_Male - Medium_Female	A_33_P3293336	NM_005264	NM_005264

435	LOC10050696 5	uncharacterized LOC100506965	2.6004	0.036016	1.4435	0.41323	High_Female - High_Male; Low_Female - High_Female; Low_Male - High_Female; High_Female - Medium_Female; Medium_Male - High_Female; Low_Female - High_Male; Low_Male - High_Male; Medium_Female - High_Male; Medium_Male - High_Male; Low_Male - Low_Female; Low_Female - Medium_Female; Low_Female - Medium_Male; Low_Male - Medium_Female; Low_Male - Medium_Male; Medium_Male - Medium_Female	A_19_P00803019	XR_110300	XR_110300
436	XLOC_000762		2.5999	0.036041	1.4432	0.41323	High_Male - High_Female; Low_Female - High_Female; Low_Male - High_Female; Medium_Female - High_Female; Medium_Male - High_Female; Low_Female - High_Male; Low_Male - High_Male; High_Male - Medium_Female; High_Male - Medium_Male; Low_Female - Low_Male; Low_Female - Medium_Female; Low_Female - Medium_Male; Low_Male - Medium_Female; Low_Male - Medium_Male; Medium_Female - Medium_Male	A_21_P0001486		AA773613

437			2.5962	0.03626	1.4406	0.41429	High_Male - High_Female;<br>Low_Female - High_Female;<br>Low_Male - High_Female;<br>Medium_Female - High_Female;<br>Medium_Male - High_Female;<br>High_Male - Low_Female;<br>High_Male - Low_Male;<br>Medium_Female - High_Male;<br>High_Male - Medium_Male;<br>Low_Female - Low_Male;<br>Medium_Female - Low_Female;<br>Low_Female - Medium_Male;<br>Medium_Female - Low_Male;<br>Medium_Male - Low_Male;<br>Medium_Female - Medium_Male	A_33_P3287862		AK301306
438	FAM27E3	family with sequence similarity 27, member E3	2.5955	0.036299	1.4401	0.41429	High_Female - High_Male;<br>High_Female - Low_Female;<br>High_Female - Low_Male;<br>High_Female - Medium_Female;<br>Medium_Male - High_Female;<br>High_Male - Low_Female;<br>High_Male - Low_Male;<br>High_Male - Medium_Female;<br>Medium_Male - High_Male;<br>Low_Female - Low_Male;<br>Medium_Female - Low_Female;<br>Medium_Male - Low_Female;<br>Medium_Female - Low_Male;<br>Medium_Male - Low_Male;<br>Medium_Male - Medium_Female	A_32_P112623	XM_001720463	XM_001720463

439	DDX43	DEAD (Asp-Glu-Ala-Asp) box polypeptide 43	2.5932	0.036438	1.4385	0.41492	High_Male - High_Female; High_Female - Low_Female; Low_Male - High_Female; Medium_Female - High_Female; Medium_Male - High_Female; High_Male - Low_Female; Low_Male - High_Male; High_Male - Medium_Female; Medium_Male - High_Male; Low_Male - Low_Female; Medium_Female - Low_Female; Medium_Male - Low_Female; Low_Male - Medium_Female; Low_Male - Medium_Male; Medium_Male - Medium_Female	A_23_P156445	NM_018665	NM_018665
440	CSNK1G2	casein kinase 1, gamma 2	2.5898	0.036638	1.4361	0.41625	High_Male - High_Female; Low_Female - High_Female; Low_Male - High_Female; Medium_Female - High_Female; Medium_Male - High_Female; Low_Female - High_Male; Low_Male - High_Male; High_Male - Medium_Female; High_Male - Medium_Male; Low_Male - Low_Female; Low_Female - Medium_Female; Low_Female - Medium_Male; Low_Male - Medium_Female; Low_Male - Medium_Male; Medium_Female - Medium_Male	A_33_P3259017	NM_001319	NM_001319

441	MPP1	membrane protein, palmitoylated 1, 55kDa	2.5855	0.036887	1.4331	0.41814	High_Female - High_Male; Low_Female - High_Female; Low_Male - High_Female; Medium_Female - High_Female; Medium_Male - High_Female; Low_Female - High_Male; Low_Male - High_Male; Medium_Female - High_Male; Medium_Male - High_Male; Low_Female - Low_Male; Low_Female - Medium_Female; Low_Female - Medium_Male; Low_Male - Medium_Female; Low_Male - Medium_Male; Medium_Female - Medium_Male	A_33_P3247858	NM_001166461	NM_001166461
442	XLOC_004188		2.5767	0.037419	1.4269	0.42321	High_Male - High_Female; Low_Female - High_Female; Low_Male - High_Female; Medium_Female - High_Female; High_Female - Medium_Male; Low_Female - High_Male; Low_Male - High_Male; High_Male - Medium_Female; High_Male - Medium_Male; Low_Female - Low_Male; Low_Female - Medium_Female; Low_Female - Medium_Male; Low_Male - Medium_Female; Low_Male - Medium_Male; Medium_Female - Medium_Male	linc|TCONS_00008670		

443	PAQR9	progestin and adipoQ receptor family member IX	2.5719	0.037707	1.4236	0.4255	High_Female - High_Male; High_Female - Low_Female; High_Female - Low_Male; High_Female - Medium_Female; High_Female - Medium_Male; Low_Female - High_Male; Low_Male - High_Male; Medium_Female - High_Male; Medium_Male - High_Male; Low_Female - Low_Male; Medium_Female - Low_Female; Medium_Male - Low_Female; Medium_Female - Low_Male; Medium_Male - Low_Male; Medium_Female - Medium_Male	A_32_P196193	NM_198504	NM_198504
444	LOC100506546	uncharacterized LOC100506546	2.5671	0.038	1.4202	0.42636	High_Male - High_Female; High_Female - Low_Female; High_Female - Low_Male; High_Female - Medium_Female; Medium_Male - High_Female; High_Male - Low_Female; High_Male - Low_Male; High_Male - Medium_Female; Medium_Male - High_Male; Low_Male - Low_Female; Medium_Female - Low_Female; Medium_Male - Low_Female; Low_Male - Medium_Female; Medium_Male - Low_Male; Medium_Male - Medium_Female	A_21_P0014063	XR_108325	XR_108325

445	OR8G2	olfactory receptor, family 8, subfamily G, member 2	2.5654	0.038101	1.4191	0.42636	High_Male - High_Female; Low_Female - High_Female; Low_Male - High_Female; Medium_Female - High_Female; Medium_Male - High_Female; Low_Female - High_Male; High_Male - Low_Male; Medium_Female - High_Male; High_Male - Medium_Male; Low_Female - Low_Male; Low_Female - Medium_Female; Low_Female - Medium_Male; Medium_Female - Low_Male; Low_Male - Medium_Male; Medium_Female - Medium_Male	A_33_P3262089	NM_001007249	NM_001007249
446	KANK2	KN motif and ankyrin repeat domains 2	2.565	0.038126	1.4188	0.42636	High_Female - High_Male; High_Female - Low_Female; High_Female - Low_Male; Medium_Female - High_Female; High_Female - Medium_Male; Low_Female - High_Male; Low_Male - High_Male; Medium_Female - High_Male; Medium_Male - High_Male; Low_Male - Low_Female; Medium_Female - Low_Female; Medium_Male - Low_Female; Medium_Female - Low_Male; Low_Male - Medium_Male; Medium_Female - Medium_Male	A_23_P50426	NM_015493	NM_015493

447	LOC652119	double homeobox protein 4-like protein 4-like	2.5636	0.038212	1.4178	0.42636	High_Male - High_Female; Low_Female - High_Female; Low_Male - High_Female; Medium_Female - High_Female; Medium_Male - High_Female; Low_Female - High_Male; Low_Male - High_Male; High_Male - Medium_Female; High_Male - Medium_Male; Low_Male - Low_Female; Low_Female - Medium_Female; Low_Female - Medium_Male; Low_Male - Medium_Female; Low_Male - Medium_Male; Medium_Male - Medium_Female	A_33_P3417123	XM_001720798	XM_001720798
448	XLOC_l2_001421		2.5628	0.038266	1.4172	0.42636	High_Male - High_Female; Low_Female - High_Female; Low_Male - High_Female; Medium_Female - High_Female; Medium_Male - High_Female; Low_Female - High_Male; High_Male - Low_Male; High_Male - Medium_Female; High_Male - Medium_Male; Low_Female - Low_Male; Low_Female - Medium_Female; Low_Female - Medium_Male; Medium_Female - Low_Male; Low_Male - Medium_Male; Medium_Female - Medium_Male	linc|TCONS_l2_00002836|linc|TCONS_l2_00002838|linc|TCONS_l2_00002835		

449			2.5623	0.038295	1.4169	0.42636	High_Male - High_Female;<br>Low_Female - High_Female;<br>Low_Male - High_Female;<br>Medium_Female - High_Female;<br>High_Female - Medium_Male;<br>Low_Female - High_Male;<br>Low_Male - High_Male;<br>High_Male - Medium_Female;<br>High_Male - Medium_Male;<br>Low_Female - Low_Male;<br>Low_Female - Medium_Female;<br>Low_Female - Medium_Male;<br>Low_Male - Medium_Female;<br>Low_Male - Medium_Male;<br>Medium_Female - Medium_Male	A_33_P3395310		BX096650
450	XLOC_009255		2.5575	0.038593	1.4135	0.42709	High_Female - High_Male;<br>High_Female - Low_Female;<br>Low_Male - High_Female;<br>Medium_Female - High_Female;<br>High_Female - Medium_Male;<br>Low_Female - High_Male;<br>Low_Male - High_Male;<br>Medium_Female - High_Male;<br>High_Male - Medium_Male;<br>Low_Male - Low_Female;<br>Medium_Female - Low_Female;<br>Low_Female - Medium_Male;<br>Medium_Female - Low_Male;<br>Low_Male - Medium_Male;<br>Medium_Female - Medium_Male	A_21_P0007129		AK055250

451			2.5561	0.03868	1.4125	0.42709	High_Male - High_Female; Low_Female - High_Female; Low_Male - High_Female; Medium_Female - High_Female; Medium_Male - High_Female; High_Male - Low_Female; Low_Male - High_Male; High_Male - Medium_Female; High_Male - Medium_Male; Low_Male - Low_Female; Low_Female - Medium_Female; Medium_Male - Low_Female; Low_Male - Medium_Female; Low_Male - Medium_Male; Medium_Male - Medium_Female	A_33_P3346333		
452	CEACAM18	carcinoembryonic antigen-related cell adhesion molecule 18	2.5548	0.03876	1.4116	0.42709	High_Male - High_Female; Low_Female - High_Female; Low_Male - High_Female; Medium_Female - High_Female; High_Female - Medium_Male; High_Male - Low_Female; Low_Male - High_Male; Medium_Female - High_Male; High_Male - Medium_Male; Low_Male - Low_Female; Medium_Female - Low_Female; Low_Female - Medium_Male; Low_Male - Medium_Female; Low_Male - Medium_Male; Medium_Female - Medium_Male	A_33_P3249818	NM_001080405	NM_001080405

453	PDE2A	phosphodiesterase 2A, cGMP-stimulated	2.5502	0.039047	1.4084	0.42709	High_Female - High_Male; High_Female - Low_Female; High_Female - Low_Male; High_Female - Medium_Female; High_Female - Medium_Male; Low_Female - High_Male; Low_Male - High_Male; Medium_Female - High_Male; High_Male - Medium_Male; Low_Female - Low_Male; Medium_Female - Low_Female; Low_Female - Medium_Male; Medium_Female - Low_Male; Low_Male - Medium_Male; Medium_Female - Medium_Male	A_23_P401106	NM_002599	NM_002599
454	DACH1	dachshund homolog 1 (Drosophila)	2.5494	0.039098	1.4078	0.42709	High_Male - High_Female; High_Female - Low_Female; High_Female - Low_Male; High_Female - Medium_Female; Medium_Male - High_Female; High_Male - Low_Female; High_Male - Low_Male; High_Male - Medium_Female; High_Male - Medium_Male; Low_Male - Low_Female; Medium_Female - Low_Female; Medium_Male - Low_Female; Medium_Female - Low_Male; Medium_Male - Low_Male; Medium_Male - Medium_Female	A_33_P3316786	NM_080759	NM_080759

455	XLOC_005780		2.5474	0.039222	1.4065	0.42709	High_Female - High_Male;<br>Low_Female - High_Female;<br>High_Female - Low_Male;<br>High_Female - Medium_Female;<br>Medium_Male - High_Female;<br>Low_Female - High_Male;<br>Low_Male - High_Male;<br>Medium_Female - High_Male;<br>Medium_Male - High_Male;<br>Low_Female - Low_Male;<br>Low_Female - Medium_Female;<br>Medium_Male - Low_Female;<br>Low_Male - Medium_Female;<br>Medium_Male - Low_Male;<br>Medium_Male - Medium_Female	A_21_P0004765		BF507452
456	HPR	haptoglobin-related protein	2.547	0.039251	1.4061	0.42709	High_Male - High_Female;<br>High_Female - Low_Female;<br>High_Female - Low_Male;<br>Medium_Female - High_Female;<br>High_Female - Medium_Male;<br>High_Male - Low_Female;<br>High_Male - Low_Male;<br>Medium_Female - High_Male;<br>High_Male - Medium_Male;<br>Low_Female - Low_Male;<br>Medium_Female - Low_Female;<br>Medium_Male - Low_Female;<br>Medium_Female - Low_Male;<br>Medium_Male - Low_Male;<br>Medium_Female - Medium_Male	A_33_P3289236	NM_020995	NM_020995

457	CD24	CD24 molecule	2.5464	0.03929	1.4057	0.42709	High_Male - High_Female; High_Female - Low_Female; High_Female - Low_Male; Medium_Female - High_Female; Medium_Male - High_Female; High_Male - Low_Female; High_Male - Low_Male; High_Male - Medium_Female; Medium_Male - High_Male; Low_Male - Low_Female; Medium_Female - Low_Female; Medium_Male - Low_Female; Medium_Female - Low_Male; Medium_Male - Low_Male; Medium_Male - Medium_Female	A_33_P3369844	NM_013230	NM_013230
458	TNS1	tensin 1	2.5457	0.039331	1.4053	0.42709	High_Female - High_Male; Low_Female - High_Female; Low_Male - High_Female; High_Female - Medium_Female; High_Female - Medium_Male; Low_Female - High_Male; Low_Male - High_Male; Medium_Female - High_Male; High_Male - Medium_Male; Low_Male - Low_Female; Low_Female - Medium_Female; Low_Female - Medium_Male; Low_Male - Medium_Female; Low_Male - Medium_Male; Medium_Female - Medium_Male	A_24_P105733	NM_022648	NM_022648

459	PI16	peptidase inhibitor 16	2.5446	0.039402	1.4045	0.42709	High_Female - High_Male; High_Female - Low_Female; High_Female - Low_Male; High_Female - Medium_Female; High_Female - Medium_Male; Low_Female - High_Male; Low_Male - High_Male; Medium_Female - High_Male; High_Male - Medium_Male; Low_Female - Low_Male; Medium_Female - Low_Female; Low_Female - Medium_Male; Medium_Female - Low_Male; Low_Male - Medium_Male; Medium_Female - Medium_Male	A_33_P3215640	NM_153370	NM_153370
460	EYA1	eyes absent homolog 1 (Drosophila)	2.5429	0.039512	1.4033	0.42709	High_Male - High_Female; Low_Female - High_Female; Low_Male - High_Female; Medium_Female - High_Female; Medium_Male - High_Female; Low_Female - High_Male; Low_Male - High_Male; High_Male - Medium_Female; High_Male - Medium_Male; Low_Female - Low_Male; Low_Female - Medium_Female; Low_Female - Medium_Male; Low_Male - Medium_Female; Low_Male - Medium_Male; Medium_Female - Medium_Male	A_33_P3366667	NM_000503	NM_000503

461	BMS1P5	BMS1 pseudogene 5	2.5415	0.039599	1.4023	0.42709	High_Female - High_Male; Low_Female - High_Female; Low_Male - High_Female; Medium_Female - High_Female; Medium_Male - High_Female; Low_Female - High_Male; Low_Male - High_Male; Medium_Female - High_Male; Medium_Male - High_Male; Low_Male - Low_Female; Low_Female - Medium_Female; Medium_Male - Low_Female; Low_Male - Medium_Female; Low_Male - Medium_Male; Medium_Male - Medium_Female	A_21_P0010877		BC065722
462	XLOC_l2_014423		2.5415	0.0396	1.4023	0.42709	High_Male - High_Female; Low_Female - High_Female; Low_Male - High_Female; Medium_Female - High_Female; Medium_Male - High_Female; High_Male - Low_Female; Low_Male - High_Male; Medium_Female - High_Male; Medium_Male - High_Male; Low_Male - Low_Female; Medium_Female - Low_Female; Medium_Male - Low_Female; Low_Male - Medium_Female; Low_Male - Medium_Male; Medium_Male - Medium_Female	ens|ENST00000522393|ens|ENST00000523017|tc|THC2542402|tc|THC2521390		

463	XLOC_010366		2.5409	0.039634	1.4019	0.42709	High_Male - High_Female; Low_Female - High_Female; Low_Male - High_Female; Medium_Female - High_Female; Medium_Male - High_Female; Low_Female - High_Male; Low_Male - High_Male; High_Male - Medium_Female; High_Male - Medium_Male; Low_Female - Low_Male; Low_Female - Medium_Female; Low_Female - Medium_Male; Low_Male - Medium_Female; Low_Male - Medium_Male; Medium_Female - Medium_Male	ens|ENST00000425609|tc|THC2707351|linc|TCONS_00021542		
464	XLOC_005900		2.5405	0.039662	1.4016	0.42709	High_Female - High_Male; High_Female - Low_Female; High_Female - Low_Male; Medium_Female - High_Female; High_Female - Medium_Male; High_Male - Low_Female; High_Male - Low_Male; Medium_Female - High_Male; High_Male - Medium_Male; Low_Female - Low_Male; Medium_Female - Low_Female; Medium_Male - Low_Female; Medium_Female - Low_Male; Medium_Male - Low_Male; Medium_Female - Medium_Male	A_21_P0004617		AK130765

465	HOXA9	homeobox A9	2.5386	0.039785	1.4003	0.42709	High_Male - High_Female; High_Female - Low_Female; Low_Male - High_Female; Medium_Female - High_Female; Medium_Male - High_Female; High_Male - Low_Female; High_Male - Low_Male; High_Male - Medium_Female; High_Male - Medium_Male; Low_Male - Low_Female; Medium_Female - Low_Female; Medium_Male - Low_Female; Medium_Female - Low_Male; Medium_Male - Low_Male; Medium_Female - Medium_Male	A_23_P500998	NM_152739	NM_152739
466	SMR3A	submaxillary gland androgen regulated protein 3A	2.5374	0.03986	1.3995	0.42709	High_Male - High_Female; Low_Female - High_Female; Low_Male - High_Female; Medium_Female - High_Female; Medium_Male - High_Female; Low_Female - High_Male; Low_Male - High_Male; High_Male - Medium_Female; High_Male - Medium_Male; Low_Male - Low_Female; Low_Female - Medium_Female; Low_Female - Medium_Male; Low_Male - Medium_Female; Low_Male - Medium_Male; Medium_Female - Medium_Male	A_23_P41365	NM_012390	NM_012390

467	XLOC_005981		2.5363	0.039933	1.3987	0.42709	High_Male - High_Female; High_Female - Low_Female; High_Female - Low_Male; High_Female - Medium_Female; Medium_Male - High_Female; High_Male - Low_Female; High_Male - Low_Male; High_Male - Medium_Female; High_Male - Medium_Male; Low_Female - Low_Male; Medium_Female - Low_Female; Medium_Male - Low_Female; Medium_Female - Low_Male; Medium_Male - Low_Male; Medium_Male - Medium_Female	A_19_P00315705		BG434565
468			2.5355	0.039984	1.3981	0.42709	High_Female - High_Male; High_Female - Low_Female; High_Female - Low_Male; High_Female - Medium_Female; High_Female - Medium_Male; Low_Female - High_Male; Low_Male - High_Male; Medium_Female - High_Male; High_Male - Medium_Male; Low_Female - Low_Male; Low_Female - Medium_Female; Low_Female - Medium_Male; Low_Male - Medium_Female; Low_Male - Medium_Male; Medium_Female - Medium_Male	ens|ENST00000471090|tc|THC2518594		

469	LOC158863	uncharacterized LOC158863	2.53	0.040336	1.3943	0.42993	High_Female - High_Male; Low_Female - High_Female; Low_Male - High_Female; Medium_Female - High_Female; Medium_Male - High_Female; Low_Female - High_Male; Low_Male - High_Male; Medium_Female - High_Male; Medium_Male - High_Male; Low_Male - Low_Female; Low_Female - Medium_Female; Low_Female - Medium_Male; Low_Male - Medium_Female; Low_Male - Medium_Male; Medium_Male - Medium_Female	A_33_P3576797		AL110203
470			2.5241	0.040726	1.3901	0.43177	High_Male - High_Female; Low_Female - High_Female; Low_Male - High_Female; Medium_Female - High_Female; Medium_Male - High_Female; High_Male - Low_Female; High_Male - Low_Male; High_Male - Medium_Female; High_Male - Medium_Male; Low_Male - Low_Female; Low_Female - Medium_Female; Low_Female - Medium_Male; Low_Male - Medium_Female; Low_Male - Medium_Male; Medium_Female - Medium_Male	ens|ENST00000513564|ens|ENST00000505967		

471	MGLL	monoglyceride lipase	2.5238	0.04074	1.39	0.43177	High_Female - High_Male; High_Female - Low_Female; High_Female - Low_Male; High_Female - Medium_Female; High_Female - Medium_Male; Low_Female - High_Male; Low_Male - High_Male; Medium_Female - High_Male; Medium_Male - High_Male; Low_Female - Low_Male; Low_Female - Medium_Female; Low_Female - Medium_Male; Medium_Female - Low_Male; Low_Male - Medium_Male; Medium_Female - Medium_Male	A_33_P3281795	NM_007283	NM_007283
472	BEND7	BEN domain containing 7	2.5234	0.040767	1.3897	0.43177	High_Male - High_Female; High_Female - Low_Female; High_Female - Low_Male; Medium_Female - High_Female; Medium_Male - High_Female; High_Male - Low_Female; High_Male - Low_Male; High_Male - Medium_Female; High_Male - Medium_Male; Low_Male - Low_Female; Medium_Female - Low_Female; Medium_Male - Low_Female; Medium_Female - Low_Male; Medium_Male - Low_Male; Medium_Female - Medium_Male	A_23_P312358	NM_152751	NM_152751

473	XLOC_000884		2.5169	0.0412	1.3851	0.43509	High_Male - High_Female; Low_Female - High_Female; Low_Male - High_Female; Medium_Female - High_Female; High_Female - Medium_Male; Low_Female - High_Male; Low_Male - High_Male; Medium_Female - High_Male; High_Male - Medium_Male; Low_Male - Low_Female; Low_Female - Medium_Female; Low_Female - Medium_Male; Low_Male - Medium_Female; Low_Male - Medium_Male; Medium_Female - Medium_Male	ens|ENST00000420549|linc|TCONS_00000554		
474	LTF	lactotransferrin	2.5154	0.041298	1.3841	0.43509	High_Female - High_Male; High_Female - Low_Female; High_Female - Low_Male; Medium_Female - High_Female; High_Female - Medium_Male; High_Male - Low_Female; High_Male - Low_Male; Medium_Female - High_Male; High_Male - Medium_Male; Low_Male - Low_Female; Medium_Female - Low_Female; Medium_Male - Low_Female; Medium_Female - Low_Male; Low_Male - Medium_Male; Medium_Female - Medium_Male	A_23_P166848	NM_002343	NM_002343

475	CMTM5	CKLF-like MARVEL transmembrane domain containing 5	2.5123	0.041502	1.3819	0.43509	High_Female - High_Male; Low_Female - High_Female; High_Female - Low_Male; Medium_Female - High_Female; High_Female - Medium_Male; Low_Female - High_Male; Low_Male - High_Male; Medium_Female - High_Male; Medium_Male - High_Male; Low_Female - Low_Male; Low_Female - Medium_Female; Low_Female - Medium_Male; Medium_Female - Low_Male; Low_Male - Medium_Male; Medium_Female - Medium_Male	A_23_P106042	NM_001037288	NM_001037288
476	CCL2	chemokine (C-C motif) ligand 2	2.5119	0.04153	1.3816	0.43509	High_Male - High_Female; Low_Female - High_Female; Low_Male - High_Female; Medium_Female - High_Female; Medium_Male - High_Female; High_Male - Low_Female; High_Male - Low_Male; High_Male - Medium_Female; High_Male - Medium_Male; Low_Male - Low_Female; Low_Female - Medium_Female; Medium_Male - Low_Female; Low_Male - Medium_Female; Medium_Male - Low_Male; Medium_Male - Medium_Female	A_23_P89431	NM_002982	NM_002982

477	KREMEN1	kringle containing transmembrane protein 1	2.5114	0.041565	1.3813	0.43509	High_Female - High_Male; High_Female - Low_Female; High_Female - Low_Male; High_Female - Medium_Female; High_Female - Medium_Male; Low_Female - High_Male; High_Male - Low_Male; Medium_Female - High_Male; Medium_Male - High_Male; Low_Female - Low_Male; Low_Female - Medium_Female; Low_Female - Medium_Male; Medium_Female - Low_Male; Medium_Male - Low_Male; Medium_Male - Medium_Female	A_33_P3262431	NM_032045	NM_032045
478	XLOC_006277		2.5096	0.041684	1.38	0.43509	High_Female - High_Male; High_Female - Low_Female; High_Female - Low_Male; High_Female - Medium_Female; High_Female - Medium_Male; High_Male - Low_Female; High_Male - Low_Male; Medium_Female - High_Male; Medium_Male - High_Male; Low_Female - Low_Male; Medium_Female - Low_Female; Medium_Male - Low_Female; Medium_Female - Low_Male; Medium_Male - Low_Male; Medium_Male - Medium_Female	linc|TCONS_00013611		

479	LOC100190939	uncharacterized LOC100190939	2.5066	0.041884	1.378	0.43509	High_Male - High_Female; Low_Female - High_Female; Low_Male - High_Female; Medium_Female - High_Female; High_Female - Medium_Male; Low_Female - High_Male; Low_Male - High_Male; Medium_Female - High_Male; High_Male - Medium_Male; Low_Male - Low_Female; Medium_Female - Low_Female; Low_Female - Medium_Male; Low_Male - Medium_Female; Low_Male - Medium_Male; Medium_Female - Medium_Male	A_21_P0008060	NR_024458	NR_024458
480	XLOC_013744		2.5066	0.041889	1.3779	0.43509	High_Male - High_Female; Low_Female - High_Female; Low_Male - High_Female; Medium_Female - High_Female; Medium_Male - High_Female; High_Male - Low_Female; High_Male - Low_Male; High_Male - Medium_Female; High_Male - Medium_Male; Low_Female - Low_Male; Low_Female - Medium_Female; Low_Female - Medium_Male; Low_Male - Medium_Female; Low_Male - Medium_Male; Medium_Female - Medium_Male	linc|TCONS_00028390		

481			2.5058	0.041944	1.3773	0.43509	High_Male - High_Female; High_Female - Low_Female; High_Female - Low_Male; High_Female - Medium_Female; Medium_Male - High_Female; High_Male - Low_Female; High_Male - Low_Male; High_Male - Medium_Female; Medium_Male - High_Male; Low_Male - Low_Female; Medium_Female - Low_Female; Medium_Male - Low_Female; Medium_Female - Low_Male; Medium_Male - Low_Male; Medium_Male - Medium_Female	A_33_P3340852		S55273
482	CCR2	chemokine (C-C motif) receptor 2	2.5045	0.042032	1.3764	0.43509	High_Female - High_Male; High_Female - Low_Female; High_Female - Low_Male; Medium_Female - High_Female; High_Female - Medium_Male; High_Male - Low_Female; High_Male - Low_Male; Medium_Female - High_Male; High_Male - Medium_Male; Low_Female - Low_Male; Medium_Female - Low_Female; Low_Female - Medium_Male; Medium_Female - Low_Male; Medium_Male - Low_Male; Medium_Female - Medium_Male	A_23_P212354	NM_001123041	NM_001123041

483	XLOC_008079		2.5043	0.04204	1.3763	0.43509	High_Female - High_Male; High_Female - Low_Female; High_Female - Low_Male; High_Female - Medium_Female; High_Female - Medium_Male; High_Male - Low_Female; High_Male - Low_Male; Medium_Female - High_Male; Medium_Male - High_Male; Low_Female - Low_Male; Medium_Female - Low_Female; Medium_Male - Low_Female; Medium_Female - Low_Male; Medium_Male - Low_Male; Medium_Female - Medium_Male	ens|ENST00000498732|linc|TCONS_00017468		
484	LRG1	leucine-rich alpha-2-glycoprotein 1	2.5031	0.042125	1.3755	0.43509	High_Female - High_Male; High_Female - Low_Female; High_Female - Low_Male; High_Female - Medium_Female; High_Female - Medium_Male; High_Male - Low_Female; High_Male - Low_Male; High_Male - Medium_Female; High_Male - Medium_Male; Low_Female - Low_Male; Medium_Female - Low_Female; Medium_Male - Low_Female; Medium_Female - Low_Male; Medium_Male - Low_Male; Medium_Female - Medium_Male	A_23_P50638	NM_052972	NM_052972

485	LOC100505857		2.5	0.042334	1.3733	0.43635	High_Male - High_Female; Low_Female - High_Female; Low_Male - High_Female; Medium_Female - High_Female; Medium_Male - High_Female; Low_Female - High_Male; Low_Male - High_Male; High_Male - Medium_Female; Medium_Male - High_Male; Low_Female - Low_Male; Low_Female - Medium_Female; Low_Female - Medium_Male; Low_Male - Medium_Female; Low_Male - Medium_Male; Medium_Male - Medium_Female	A_21_P0014925		DA569430
486	LOC100128252	uncharacterized LOC100128252	2.4978	0.042483	1.3718	0.43698	High_Male - High_Female; Low_Female - High_Female; Low_Male - High_Female; Medium_Female - High_Female; Medium_Male - High_Female; Low_Female - High_Male; Low_Male - High_Male; High_Male - Medium_Female; Medium_Male - High_Male; Low_Male - Low_Female; Low_Female - Medium_Female; Medium_Male - Low_Female; Low_Male - Medium_Female; Medium_Male - Low_Male; Medium_Male - Medium_Female	A_32_P204795	NR_036522	NR_036522

487	C9orf29	chromosome 9 open reading frame 29	2.49	0.043022	1.3663	0.44161	High_Male - High_Female;<br>Low_Female - High_Female;<br>Low_Male - High_Female;<br>Medium_Female - High_Female;<br>Medium_Male - High_Female;<br>Low_Female - High_Male;<br>Low_Male - High_Male;<br>Medium_Female - High_Male;<br>High_Male - Medium_Male;<br>Low_Male - Low_Female;<br>Low_Female - Medium_Female;<br>Low_Female - Medium_Male;<br>Low_Male - Medium_Female;<br>Low_Male - Medium_Male;<br>Medium_Female - Medium_Male	A_33_P3399438	NR_034087	NR_034087
488	CAV2	caveolin 2	2.4871	0.04322	1.3643	0.44274	High_Male - High_Female;<br>Low_Female - High_Female;<br>Low_Male - High_Female;<br>Medium_Female - High_Female;<br>High_Female - Medium_Male;<br>Low_Female - High_Male;<br>High_Male - Low_Male;<br>High_Male - Medium_Female;<br>High_Male - Medium_Male;<br>Low_Female - Low_Male;<br>Low_Female - Medium_Female;<br>Low_Female - Medium_Male;<br>Medium_Female - Low_Male;<br>Low_Male - Medium_Male;<br>Medium_Female - Medium_Male	A_23_P123071	NM_001233	NM_001233

489	LOC100653060	putative POM121-like protein 1-like	2.4844	0.043409	1.3624	0.44289	High_Female - High_Male; High_Female - Low_Female; High_Female - Low_Male; Medium_Female - High_Female; High_Female - Medium_Male; Low_Female - High_Male; High_Male - Low_Male; Medium_Female - High_Male; High_Male - Medium_Male; Low_Female - Low_Male; Medium_Female - Low_Female; Low_Female - Medium_Male; Medium_Female - Low_Male; Medium_Male - Low_Male; Medium_Female - Medium_Male	A_32_P49668	XM_003403393	XM_003403393
490	KIR2DL5A	killer cell immunoglobulin-like receptor, two domains, long cytoplasmic tail, 5A	2.4844	0.043412	1.3624	0.44289	High_Male - High_Female; High_Female - Low_Female; Low_Male - High_Female; Medium_Female - High_Female; Medium_Male - High_Female; High_Male - Low_Female; Low_Male - High_Male; Medium_Female - High_Male; Medium_Male - High_Male; Low_Male - Low_Female; Medium_Female - Low_Female; Medium_Male - Low_Female; Low_Male - Medium_Female; Low_Male - Medium_Male; Medium_Female - Medium_Male	A_24_P5890	NM_020535	NM_020535

491	SNORD90	small nucleolar RNA, C/D box 90	2.4818	0.043596	1.3606	0.44387	High_Male - High_Female; Low_Female - High_Female; Low_Male - High_Female; Medium_Female - High_Female; Medium_Male - High_Female; Low_Female - High_Male; Low_Male - High_Male; Medium_Female - High_Male; Medium_Male - High_Male; Low_Male - Low_Female; Medium_Female - Low_Female; Medium_Male - Low_Female; Low_Male - Medium_Female; Low_Male - Medium_Male; Medium_Male - Medium_Female	A_21_P0000387	NR_003071	NR_003071
492	LOC100653245	ig heavy chain V-III region VH26-like	2.4731	0.04421	1.3545	0.4492	High_Female - High_Male; High_Female - Low_Female; Low_Male - High_Female; Medium_Female - High_Female; Medium_Male - High_Female; High_Male - Low_Female; Low_Male - High_Male; Medium_Female - High_Male; Medium_Male - High_Male; Low_Male - Low_Female; Medium_Female - Low_Female; Medium_Male - Low_Female; Medium_Female - Low_Male; Low_Male - Medium_Male; Medium_Female - Medium_Male	A_24_P627503	XM_003403831	XM_003403831

493	LOC100507195	uncharacterized LOC100507195	2.4713	0.044335	1.3533	0.44956	High_Female - High_Male; High_Female - Low_Female; High_Female - Low_Male; High_Female - Medium_Female; High_Female - Medium_Male; High_Male - Low_Female; High_Male - Low_Male; High_Male - Medium_Female; Medium_Male - High_Male; Low_Female - Low_Male; Low_Female - Medium_Female; Medium_Male - Low_Female; Low_Male - Medium_Female; Medium_Male - Low_Male; Medium_Male - Medium_Female	A_21_P0014580	XR_110374	XR_110374
494	C4BPA	complement component 4 binding protein, alpha	2.4678	0.044591	1.3508	0.45059	High_Male - High_Female; Low_Female - High_Female; Low_Male - High_Female; High_Female - Medium_Female; High_Female - Medium_Male; Low_Female - High_Male; Low_Male - High_Male; High_Male - Medium_Female; High_Male - Medium_Male; Low_Female - Low_Male; Low_Female - Medium_Female; Low_Female - Medium_Male; Low_Male - Medium_Female; Low_Male - Medium_Male; Medium_Female - Medium_Male	A_23_P97541	NM_000715	NM_000715

495	LOC728975	uncharacterized LOC728975	2.4674	0.044617	1.3505	0.45059	High_Female - High_Male; High_Female - Low_Female; High_Female - Low_Male; High_Female - Medium_Female; High_Female - Medium_Male; Low_Female - High_Male; High_Male - Low_Male; Medium_Female - High_Male; High_Male - Medium_Male; Low_Female - Low_Male; Medium_Female - Low_Female; Low_Female - Medium_Male; Medium_Female - Low_Male; Low_Male - Medium_Male; Medium_Female - Medium_Male	A_33_P3337019	XR_110914	XR_110914
496	TNNT1	troponin T type 1 (skeletal, slow)	2.4647	0.044807	1.3487	0.45064	High_Male - High_Female; High_Female - Low_Female; High_Female - Low_Male; Medium_Female - High_Female; Medium_Male - High_Female; High_Male - Low_Female; High_Male - Low_Male; High_Male - Medium_Female; High_Male - Medium_Male; Low_Male - Low_Female; Medium_Female - Low_Female; Medium_Male - Low_Female; Medium_Female - Low_Male; Medium_Male - Low_Male; Medium_Male - Medium_Female	A_33_P3397865	NM_003283	NM_003283

497	XLOC_009567		2.4637	0.044882	1.3479	0.45064	High_Male - High_Female; Low_Female - High_Female; Low_Male - High_Female; Medium_Female - High_Female; Medium_Male - High_Female; Low_Female - High_Male; Low_Male - High_Male; High_Male - Medium_Female; High_Male - Medium_Male; Low_Male - Low_Female; Low_Female - Medium_Female; Low_Female - Medium_Male; Low_Male - Medium_Female; Low_Male - Medium_Male; Medium_Female - Medium_Male	ens|ENST00000457746|linc|TCONS_00019176		
498	XLOC_013832		2.4626	0.044961	1.3472	0.45064	High_Male - High_Female; Low_Female - High_Female; Low_Male - High_Female; Medium_Female - High_Female; Medium_Male - High_Female; High_Male - Low_Female; High_Male - Low_Male; High_Male - Medium_Female; High_Male - Medium_Male; Low_Male - Low_Female; Medium_Female - Low_Female; Medium_Male - Low_Female; Medium_Female - Low_Male; Low_Male - Medium_Male; Medium_Female - Medium_Male	linc|TCONS_00028459		

499	XLOC_008752		2.4611	0.045069	1.3461	0.45064	High_Male - High_Female; Low_Female - High_Female; Low_Male - High_Female; Medium_Female - High_Female; Medium_Male - High_Female; Low_Female - High_Male; High_Male - Low_Male; High_Male - Medium_Female; High_Male - Medium_Male; Low_Female - Low_Male; Low_Female - Medium_Female; Low_Female - Medium_Male; Low_Male - Medium_Female; Low_Male - Medium_Male; Medium_Female - Medium_Male	ens|ENST00000455612|ens|ENST00000449071|linc|TCONS_00017940|linc|TCONS_00017941		
500	MIA	melanoma inhibitory activity	2.4611	0.045073	1.3461	0.45064	High_Female - High_Male; High_Female - Low_Female; High_Female - Low_Male; High_Female - Medium_Female; Medium_Male - High_Female; High_Male - Low_Female; High_Male - Low_Male; High_Male - Medium_Female; Medium_Male - High_Male; Low_Female - Low_Male; Medium_Female - Low_Female; Medium_Male - Low_Female; Medium_Female - Low_Male; Medium_Male - Low_Male; Medium_Male - Medium_Female	A_23_P4714	NM_006533	NM_006533

501	LOC340335	uncharacterized LOC340335	2.4557	0.045461	1.3424	0.45315	High_Male - High_Female; Low_Female - High_Female; Low_Male - High_Female; Medium_Female - High_Female; Medium_Male - High_Female; Low_Female - High_Male; Low_Male - High_Male; Medium_Female - High_Male; Medium_Male - High_Male; Low_Male - Low_Female; Low_Female - Medium_Female; Medium_Male - Low_Female; Low_Male - Medium_Female; Low_Male - Medium_Male; Medium_Male - Medium_Female	A_33_P3812038		AK074459
502	GSPT1	G1 to S phase transition 1	2.4551	0.045506	1.3419	0.45315	High_Female - High_Male; Low_Female - High_Female; Low_Male - High_Female; Medium_Female - High_Female; Medium_Male - High_Female; Low_Female - High_Male; Low_Male - High_Male; Medium_Female - High_Male; Medium_Male - High_Male; Low_Male - Low_Female; Medium_Female - Low_Female; Low_Female - Medium_Male; Medium_Female - Low_Male; Low_Male - Medium_Male; Medium_Female - Medium_Male	A_24_P73669	NM_002094	NM_002094

503	SORD	sorbitol dehydrogenase	2.4508	0.045824	1.3389	0.45505	High_Male - High_Female;<br>Low_Female - High_Female;<br>Low_Male - High_Female;<br>Medium_Female - High_Female;<br>Medium_Male - High_Female;<br>High_Male - Low_Female;<br>High_Male - Low_Male;<br>High_Male - Medium_Female;<br>High_Male - Medium_Male;<br>Low_Male - Low_Female;<br>Medium_Female - Low_Female;<br>Medium_Male - Low_Female;<br>Medium_Female - Low_Male;<br>Medium_Male - Low_Male;<br>Medium_Male - Medium_Female	A_23_P77103	NM_003104	NM_003104
504	F13A1	coagulation factor XIII, A1 polypeptide	2.4486	0.045985	1.3374	0.45505	High_Female - High_Male;<br>Low_Female - High_Female;<br>High_Female - Low_Male;<br>Medium_Female - High_Female;<br>High_Female - Medium_Male;<br>Low_Female - High_Male;<br>High_Male - Low_Male;<br>Medium_Female - High_Male;<br>Medium_Male - High_Male;<br>Low_Female - Low_Male;<br>Medium_Female - Low_Female;<br>Low_Female - Medium_Male;<br>Medium_Female - Low_Male;<br>Medium_Male - Low_Male;<br>Medium_Female - Medium_Male	A_33_P3416097	NM_000129	NM_000129

505	IER5L	immediate early response 5-like	2.4482	0.046017	1.3371	0.45505	High_Male - High_Female; Low_Female - High_Female; Low_Male - High_Female; Medium_Female - High_Female; Medium_Male - High_Female; Low_Female - High_Male; Low_Male - High_Male; Medium_Female - High_Male; High_Male - Medium_Male; Low_Male - Low_Female; Low_Female - Medium_Female; Low_Female - Medium_Male; Low_Male - Medium_Female; Low_Male - Medium_Male; Medium_Female - Medium_Male	A_33_P3302312	NM_203434	NM_203434
506	GPR87	G protein-coupled receptor 87	2.4476	0.046061	1.3367	0.45505	High_Male - High_Female; Low_Female - High_Female; High_Female - Low_Male; Medium_Female - High_Female; High_Female - Medium_Male; Low_Female - High_Male; High_Male - Low_Male; Medium_Female - High_Male; High_Male - Medium_Male; Low_Female - Low_Male; Medium_Female - Low_Female; Low_Female - Medium_Male; Medium_Female - Low_Male; Medium_Male - Low_Male; Medium_Female - Medium_Male	A_23_P121120	NM_023915	NM_023915

507	SOAT2	sterol O-acyltransferase 2	2.4415	0.046515	1.3324	0.45694	High_Female - High_Male; Low_Female - High_Female; Low_Male - High_Female; Medium_Female - High_Female; High_Female - Medium_Male; Low_Female - High_Male; Low_Male - High_Male; Medium_Female - High_Male; High_Male - Medium_Male; Low_Male - Low_Female; Low_Female - Medium_Female; Low_Female - Medium_Male; Low_Male - Medium_Female; Low_Male - Medium_Male; Medium_Female - Medium_Male	A_23_P25475	NM_003578	NM_003578
508	TRAK2	trafficking protein, kinesin binding 2	2.4404	0.046599	1.3316	0.45694	High_Female - High_Male; High_Female - Low_Female; High_Female - Low_Male; Medium_Female - High_Female; Medium_Male - High_Female; Low_Female - High_Male; High_Male - Low_Male; Medium_Female - High_Male; Medium_Male - High_Male; Low_Female - Low_Male; Medium_Female - Low_Female; Medium_Male - Low_Female; Medium_Female - Low_Male; Medium_Male - Low_Male; Medium_Female - Medium_Male	A_23_P209426	NM_015049	NM_015049

509	MOV10L1	Mov10l1, Moloney leukemia virus 10-like 1, homolog (mouse)	2.4402	0.046615	1.3315	0.45694	High_Male - High_Female; Low_Female - High_Female; Low_Male - High_Female; Medium_Female - High_Female; Medium_Male - High_Female; Low_Female - High_Male; Low_Male - High_Male; Medium_Female - High_Male; High_Male - Medium_Male; Low_Male - Low_Female; Low_Female - Medium_Female; Low_Female - Medium_Male; Low_Male - Medium_Female; Low_Male - Medium_Male; Medium_Female - Medium_Male	A_23_P143774	NM_018995	NM_018995
510	CEACAM8	carcinoembryonic antigen-related cell adhesion molecule 8	2.4392	0.046692	1.3308	0.45694	High_Female - High_Male; High_Female - Low_Female; High_Female - Low_Male; Medium_Female - High_Female; High_Female - Medium_Male; High_Male - Low_Female; High_Male - Low_Male; Medium_Female - High_Male; High_Male - Medium_Male; Low_Male - Low_Female; Medium_Female - Low_Female; Medium_Male - Low_Female; Medium_Female - Low_Male; Medium_Male - Low_Male; Medium_Female - Medium_Male	A_23_P380240	NM_001816	NM_001816

511	LOC100190986	uncharacterized LOC100190986	2.4389	0.046709	1.3306	0.45694	High_Female - High_Male; Low_Female - High_Female; Low_Male - High_Female; High_Female - Medium_Female; Medium_Male - High_Female; Low_Female - High_Male; Low_Male - High_Male; High_Male - Medium_Female; Medium_Male - High_Male; Low_Male - Low_Female; Low_Female - Medium_Female; Medium_Male - Low_Female; Low_Male - Medium_Female; Medium_Male - Low_Male; Medium_Male - Medium_Female	A_24_P693321	NR_024456	NR_024456
512	CLPS	colipase, pancreatic	2.4333	0.047135	1.3267	0.46021	High_Male - High_Female; Low_Female - High_Female; Low_Male - High_Female; Medium_Female - High_Female; High_Female - Medium_Male; Low_Female - High_Male; Low_Male - High_Male; Medium_Female - High_Male; High_Male - Medium_Male; Low_Male - Low_Female; Medium_Female - Low_Female; Low_Female - Medium_Male; Low_Male - Medium_Female; Low_Male - Medium_Male; Medium_Female - Medium_Male	A_23_P8142	NM_001832	NM_001832

513			2.4295	0.047421	1.324	0.4621	High_Female - High_Male; High_Female - Low_Female; High_Female - Low_Male; High_Female - Medium_Female; High_Female - Medium_Male; High_Male - Low_Female; Low_Male - High_Male; Medium_Female - High_Male; High_Male - Medium_Male; Low_Male - Low_Female; Medium_Female - Low_Female; Low_Female - Medium_Male; Medium_Female - Low_Male; Low_Male - Medium_Male; Medium_Female - Medium_Male	A_33_P3233819		
514	CNKSR3	CNKSR family member 3	2.4277	0.04756	1.3228	0.46255	High_Male - High_Female; Low_Female - High_Female; High_Female - Low_Male; Medium_Female - High_Female; Medium_Male - High_Female; High_Male - Low_Female; High_Male - Low_Male; High_Male - Medium_Female; Medium_Male - High_Male; Low_Female - Low_Male; Low_Female - Medium_Female; Medium_Male - Low_Female; Medium_Female - Low_Male; Medium_Male - Low_Male; Medium_Male - Medium_Female	A_23_P134085	NM_173515	NM_173515

515	HBZ	hemoglobin, zeta	2.4264	0.047661	1.3218	0.46263	High_Female - High_Male; High_Female - Low_Female; High_Female - Low_Male; High_Female - Medium_Female; High_Female - Medium_Male; High_Male - Low_Female; Low_Male - High_Male; High_Male - Medium_Female; High_Male - Medium_Male; Low_Male - Low_Female; Medium_Female - Low_Female; Low_Female - Medium_Male; Low_Male - Medium_Female; Low_Male - Medium_Male; Medium_Female - Medium_Male	A_21_P0011413	NM_005332	NM_005332
516	ZNF488	zinc finger protein 488	2.4246	0.047801	1.3206	0.4631	High_Male - High_Female; Low_Female - High_Female; Low_Male - High_Female; Medium_Female - High_Female; High_Female - Medium_Male; Low_Female - High_Male; Low_Male - High_Male; Medium_Female - High_Male; High_Male - Medium_Male; Low_Female - Low_Male; Low_Female - Medium_Female; Low_Female - Medium_Male; Low_Male - Medium_Female; Low_Male - Medium_Male; Medium_Female - Medium_Male	A_23_P23966	NM_153034	NM_153034

517	LSR	lipolysis stimulated lipoprotein receptor	2.4202	0.048135	1.3175	0.46543	High_Female - High_Male; High_Female - Low_Female; Low_Male - High_Female; High_Female - Medium_Female; High_Female - Medium_Male; High_Male - Low_Female; Low_Male - High_Male; Medium_Female - High_Male; High_Male - Medium_Male; Low_Male - Low_Female; Medium_Female - Low_Female; Low_Female - Medium_Male; Low_Male - Medium_Female; Low_Male - Medium_Male; Medium_Female - Medium_Male	A_23_P142389	NM_205834	NM_205834
518	XLOC_005127		2.4125	0.048736	1.3122	0.46955	High_Male - High_Female; Low_Female - High_Female; High_Female - Low_Male; Medium_Female - High_Female; High_Female - Medium_Male; Low_Female - High_Male; High_Male - Low_Male; Medium_Female - High_Male; High_Male - Medium_Male; Low_Female - Low_Male; Low_Female - Medium_Female; Low_Female - Medium_Male; Medium_Female - Low_Male; Medium_Male - Low_Male; Medium_Female - Medium_Male	A_19_P00316820		AK091028

519	XLOC_010065		2.4124	0.048749	1.312	0.46955	High_Male - High_Female;<br>Low_Female - High_Female;<br>Low_Male - High_Female;<br>Medium_Female - High_Female;<br>Medium_Male - High_Female;<br>Low_Female - High_Male;<br>Low_Male - High_Male;<br>High_Male - Medium_Female;<br>High_Male - Medium_Male;<br>Low_Female - Low_Male;<br>Low_Female - Medium_Female;<br>Low_Female - Medium_Male;<br>Low_Male - Medium_Female;<br>Low_Male - Medium_Male;<br>Medium_Female - Medium_Male	linc|TCONS_00020777		
520	ORMDL3	ORM1-like 3 (S. cerevisiae)	2.4093	0.048991	1.3099	0.47021	High_Male - High_Female;<br>Low_Female - High_Female;<br>Low_Male - High_Female;<br>Medium_Female - High_Female;<br>Medium_Male - High_Female;<br>Low_Female - High_Male;<br>Low_Male - High_Male;<br>High_Male - Medium_Female;<br>High_Male - Medium_Male;<br>Low_Male - Low_Female;<br>Low_Female - Medium_Female;<br>Low_Female - Medium_Male;<br>Low_Male - Medium_Female;<br>Low_Male - Medium_Male;<br>Medium_Female - Medium_Male	A_23_P129829	NM_139280	NM_139280

521	LOC100129387	uncharacterized LOC100129387	2.4091	0.049008	1.3097	0.47021	High_Female - High_Male; Low_Female - High_Female; Low_Male - High_Female; Medium_Female - High_Female; Medium_Male - High_Female; Low_Female - High_Male; Low_Male - High_Male; Medium_Female - High_Male; Medium_Male - High_Male; Low_Male - Low_Female; Medium_Female - Low_Female; Medium_Male - Low_Female; Low_Male - Medium_Female; Medium_Male - Low_Male; Medium_Male - Medium_Female	A_33_P3333777	NR_024490	NR_024490
522	LOC100505904	uncharacterized LOC100505904	2.4079	0.049099	1.3089	0.47021	High_Female - High_Male; High_Female - Low_Female; High_Female - Low_Male; High_Female - Medium_Female; High_Female - Medium_Male; Low_Female - High_Male; High_Male - Low_Male; High_Male - Medium_Female; High_Male - Medium_Male; Low_Female - Low_Male; Low_Female - Medium_Female; Low_Female - Medium_Male; Low_Male - Medium_Female; Medium_Male - Low_Male; Medium_Male - Medium_Female	A_21_P0005210	XR_110640	XR_110640

523			2.4064	0.049216	1.3079	0.47042	High_Male - High_Female;<br>Low_Female - High_Female;<br>Low_Male - High_Female;<br>Medium_Female - High_Female;<br>Medium_Male - High_Female;<br>Low_Female - High_Male;<br>Low_Male - High_Male;<br>High_Male - Medium_Female;<br>High_Male - Medium_Male;<br>Low_Male - Low_Female;<br>Low_Female - Medium_Female;<br>Low_Female - Medium_Male;<br>Low_Male - Medium_Female;<br>Low_Male - Medium_Male;<br>Medium_Female - Medium_Male	A_33_P3240340		
524			2.404	0.049411	1.3062	0.47119	High_Male - High_Female;<br>Low_Female - High_Female;<br>Low_Male - High_Female;<br>Medium_Female - High_Female;<br>Medium_Male - High_Female;<br>Low_Female - High_Male;<br>Low_Male - High_Male;<br>High_Male - Medium_Female;<br>High_Male - Medium_Male;<br>Low_Male - Low_Female;<br>Low_Female - Medium_Female;<br>Low_Female - Medium_Male;<br>Low_Male - Medium_Female;<br>Low_Male - Medium_Male;<br>Medium_Female - Medium_Male	A_33_P3271885		

525	MIR22HG	MIR22 host gene (non-protein coding)	2.3995	0.049767	1.3031	0.47119	High_Male - High_Female;<br>Low_Female - High_Female;<br>Low_Male - High_Female;<br>Medium_Female - High_Female;<br>Medium_Male - High_Female;<br>Low_Female - High_Male;<br>Low_Male - High_Male;<br>Medium_Female - High_Male;<br>High_Male - Medium_Male;<br>Low_Male - Low_Female;<br>Low_Female - Medium_Female;<br>Low_Female - Medium_Male;<br>Low_Male - Medium_Female;<br>Low_Male - Medium_Male;<br>Medium_Female - Medium_Male	A_24_P253723	NR_028502	NR_028502
526	XLOC_l2_001953		2.3992	0.049791	1.3028	0.47119	High_Female - High_Male;<br>Low_Female - High_Female;<br>Low_Male - High_Female;<br>High_Female - Medium_Female;<br>Medium_Male - High_Female;<br>Low_Female - High_Male;<br>Low_Male - High_Male;<br>High_Male - Medium_Female;<br>Medium_Male - High_Male;<br>Low_Female - Low_Male;<br>Low_Female - Medium_Female;<br>Medium_Male - Low_Female;<br>Low_Male - Medium_Female;<br>Medium_Male - Low_Male;<br>Medium_Male - Medium_Female	A_21_P0010944		AL832069

527	LOC100507195	uncharacterized LOC100507195	2.3989	0.049814	1.3027	0.47119	High_Female - High_Male; High_Female - Low_Female; High_Female - Low_Male; High_Female - Medium_Female; High_Female - Medium_Male; High_Male - Low_Female; High_Male - Low_Male; High_Male - Medium_Female; Medium_Male - High_Male; Low_Male - Low_Female; Medium_Female - Low_Female; Medium_Male - Low_Female; Medium_Female - Low_Male; Medium_Male - Low_Male; Medium_Male - Medium_Female	A_21_P0007880	XR_110374	XR_110374
528	RGS12	regulator of G-protein signaling 12	2.3984	0.049856	1.3023	0.47119	High_Male - High_Female; Low_Female - High_Female; Low_Male - High_Female; Medium_Female - High_Female; Medium_Male - High_Female; Low_Female - High_Male; Low_Male - High_Male; High_Male - Medium_Female; High_Male - Medium_Male; Low_Male - Low_Female; Low_Female - Medium_Female; Low_Female - Medium_Male; Low_Male - Medium_Female; Low_Male - Medium_Male; Medium_Female - Medium_Male	A_23_P69513	NM_198229	NM_198229

529	CAT	catalase	2.397	0.049972	1.3013	0.47119	High_Male - High_Female; Low_Female - High_Female; Low_Male - High_Female; Medium_Female - High_Female; Medium_Male - High_Female; High_Male - Low_Female; High_Male - Low_Male; High_Male - Medium_Female; Medium_Male - High_Male; Low_Male - Low_Female; Medium_Female - Low_Female; Medium_Male - Low_Female; Medium_Female - Low_Male; Medium_Male - Low_Male; Medium_Male - Medium_Female	A_23_P105138	NM_001752	NM_001752

Total in Data 1	476
Total in Data 2	529
Unique in Data 1	476
Unique in Data 2	439
Common in both	7
