# Supplementary material for: Association between Arsenic Level, Gene Expression in Asian Population, and In Vitro Carcinogenic Bladder Tumor
Source: Oxid Med Cell Longev. 2022 Jan 7;2022:3459855. doi: 10.1155/2022/3459855 (PMC8760535; doi:10.1155/2022/3459855)
Supplement: Supplementary 4 — Table S3: significant pathways in Data1 and Data2 (male and female sex) and the overlap between two (highlighted in pink). [file 3459855.f4.pdf]

Table S3. Significant pathways in Data-1 and Data-2 (male, and female genders) and the overlap between two (highlighted in pink).

	Data1			
S.No.	Ingenuity Canonical Pathways	-log(p-value)	Ratio	Molecules
1	CD28 Signaling in T Helper Cells	5.41E+00	1.00E-01	ACTR3,CARD11,CD28,CDC42,FCER1G,HLA-DMA,HLA-DMB,ITK,PAK1,PIK3R1,PLCG1,TRGV9
2	Macropinocytosis Signaling	4.79E+00	1.18E-01	CDC42,ITGB3,ITGB6,PAK1,PIK3R1,PLCG1,PRKD1,RRAS2,USP6NL
3	IL-8 Signaling	4.41E+00	7.00E-02	BCL2,BRAF,CDC42,CXCR2,GNG5,IRAK3,ITGB3,NCF2,PIK3R1,PLD3,PRKD1,RHOQ,RRAS2,VEGFA
4	Virus Entry via Endocytic Pathways	4.35E+00	9.35E-02	ACTA2,CDC42,FLNB,ITGB3,ITGB6,ITSN1,PIK3R1,PLCG1,PRKD1,RRAS2
5	Role of NFAT in Regulation of the Immune Response	4.25E+00	7.18E-02	CD28,FCER1A,FCER1G,FCGR2B,GNG5,HLA-DMA,HLA-DMB,ITK,PIK3R1,PLCB1,PLCG1,RRAS2,TRGV9
6	Fcγ Receptor-mediated Phagocytosis in Macrophages and Monocytes	4.05E+00	9.57E-02	ACTA2,ACTR3,CBL,CDC42,PAK1,PIK3R1,PLCG1,PLD3,PRKD1
7	Tec Kinase Signaling	4.05E+00	7.32E-02	ACTA2,CDC42,FCER1A,FCER1G,GNG5,ITK,PAK1,PIK3R1,PLCG1,PRKD1,RHOQ,TRGV9
8	Protein Ubiquitination Pathway	3.51E+00	5.49E-02	CBL,DNAJC22,DNAJC8,HSPA2,IFNG,PSMC1,PSMD2,PSMD7,SKP1,UBE2A,UBE2D1,UBE2E2,UBE2R2,UBE3A,USP18
9	iCOS-iCOSL Signaling in T Helper Cells	3.50E+00	8.11E-02	CD28,FCER1G,HLA-DMA,HLA-DMB,ICOS,ITK,PIK3R1,PLCG1,TRGV9
10	Graft-versus-Host Disease Signaling	3.50E+00	1.25E-01	CD28,FCER1G,HLA-DMA,HLA-DMB,IFNG,TRGV9
11	Th2 Pathway	3.48E+00	7.35E-02	BHLHE41,CD28,GFI1,HLA-DMA,HLA-DMB,ICOS,IFNG,PIK3R1,PSEN1,PTGDR2
12	Acute Myeloid Leukemia Signaling	3.47E+00	8.99E-02	BRAF,IDH1,JUP,MYC,PIK3R1,PIM1,PIM2,RRAS2
13	Th1 and Th2 Activation Pathway	3.28E+00	6.43E-02	BHLHE41,CD28,GFI1,HLA-DMA,HLA-DMB,ICOS,IFNG,mir-29,PIK3R1,PSEN1,PTGDR2
14	Phospholipase C Signaling	3.27E+00	5.45E-02	ARHGEF18,CDC42,FCER1G,FCGR2B,GNG5,ITK,PLA2G4A,PLCB1,PLCG1,PLD3,PRKD1,RHOQ,RRAS2,TRGV9
15	T Helper Cell Differentiation	3.27E+00	9.59E-02	CD28,FCER1G,HLA-DMA,HLA-DMB,ICOS,IFNG,TRGV9
16	T Cell Exhaustion Signaling Pathway	3.19E+00	6.29E-02	BTLA,CD28,FCER1G,HLA-DMA,HLA-DMB,IFNG,PIK3R1,PLCG1,RRAS2,TRGV9,VEGFA
17	VEGF Signaling	3.16E+00	8.08E-02	ACTA2,BCL2,EIF1AX,EIF2S1,PIK3R1,PLCG1,RRAS2,VEGFA
18	Phagosome Formation	3.12E+00	7.20E-02	CDC42,FCER1A,FCER1G,FCGR2B,PIK3R1,PLCB1,PLCG1,PRKD1,RHOQ
19	Prostanoid Biosynthesis	3.10E+00	3.00E-01	CYP2S1,PTGDS,TBXAS1
20	NER Pathway	3.05E+00	7.77E-02	GTF2H1,H4C13,H4C9,HMGN1,PCNA,POLD2,POLR2E,RFC4
21	PKCθ Signaling in T Lymphocytes	3.04E+00	6.45E-02	CACNA1C,CARD11,CD28,FCER1G,HLA-DMA,HLA-DMB,PIK3R1,PLCG1,RRAS2,TRGV9
22	Hepatic Fibrosis Signaling Pathway	3.03E+00	4.62E-02	ACTA2,BCL2,BRAF,CACNA1C,CDC42,IL1R1,IRAK3,ITGB3,MYC,NCF2,PIK3R1,PLCG1,PRKD1,RHOQ,RRAS2,TIMP1,VEGFA
23	Dendritic Cell Maturation	3.03E+00	6.01E-02	CD1C,CD1D,FCER1G,FCGR2B,HLA-DMA,HLA-DMB,IRF8,PIK3R1,PLCB1,PLCG1,TRGV9
24	T Cell Receptor Signaling	2.99E+00	7.62E-02	CARD11,CBL,CD28,ITK,PIK3R1,PLCG1,RASA1,RRAS2
25	HER-2 Signaling in Breast Cancer	2.91E+00	8.33E-02	CDC42,ITGB3,ITGB6,PIK3R1,PLCG1,PRKD1,RRAS2

Total in Data 1	145
Total in Data 2	36
Common in both	6
Unique in Data 1	139
Unique in Data 2	30

26	Type I Diabetes Mellitus Signaling	2.84E+00	7.21E-02	BCL2,CD28,FCER1G,HLA-DMA,HLA-DMB,IFNG,IL1R1,TRGV9
27	Lipid Antigen Presentation by CD1	2.83E+00	1.54E-01	CD1C,CD1D,FCER1G,TRGV9
28	Phospholipases	2.83E+00	9.38E-02	PLA2G15,PLA2G4A,PLA2G7,PLCB1,PLCG1,PLD3
29	EIF2 Signaling	2.82E+00	5.36E-02	ACTA2,BCL2,EIF1AX,EIF2AK3,EIF2AK4,EIF2S1,MYC,PIK3R1,RPS4X,RPSA,RRAS2,VEGFA
30	Calcium-induced T Lymphocyte Apoptosis	2.76E+00	9.09E-02	FCER1G,HLA-DMA,HLA-DMB,PLCG1,PRKD1,TRGV9
31	Pyridoxal 5'-phosphate Salvage Pathway	2.76E+00	9.09E-02	BRAF,CDK1,PAK1,PIM1,PLK1,PRKX
32	Molecular Mechanisms of Cancer	2.75E+00	4.35E-02	ARHGEF18,BCL2,BRAF,CBL,CDC42,CDK1,MYC,PAK1,PIK3R1,PLCB1,PRKD1,PSEN1,RASA1,RHOQ,RRAS2,SMAD5,SMAD9
33	Altered T Cell and B Cell Signaling in Rheumatoid Arthritis	2.73E+00	7.78E-02	CD28,FCER1G,HLA-DMA,HLA-DMB,IFNG,TNFSF13,TRGV9
34	fMLP Signaling in Neutrophils	2.71E+00	6.90E-02	ACTR3,CDC42,GNG5,NCF2,PIK3R1,PLCB1,PRKD1,RRAS2
35	Role of Tissue Factor in Cancer	2.69E+00	6.84E-02	CDC42,ITGB3,P4HB,PAK1,PIK3R1,PLCB1,RRAS2,VEGFA
36	Nur77 Signaling in T Lymphocytes	2.69E+00	8.82E-02	BCL2,CD28,FCER1G,PCNA,PRKD1,TRGV9
37	Th1 Pathway	2.60E+00	6.61E-02	CD28,HLA-DMA,HLA-DMB,ICOS,IFNG,mir-29,PIK3R1,PSEN1
38	Autoimmune Thyroid Disease Signaling	2.60E+00	1.02E-01	CD28,FCER1G,HLA-DMA,HLA-DMB,TRGV9
39	Amyotrophic Lateral Sclerosis Signaling	2.55E+00	7.22E-02	BCL2,CACNA1C,GPX1,GRIA4,PAK1,PIK3R1,VEGFA
40	Salvage Pathways of Pyrimidine Ribonucleotides	2.52E+00	7.14E-02	BRAF,CDK1,CMPK2,PAK1,PIM1,PLK1,PRKX
41	Integrin Signaling	2.51E+00	5.16E-02	ACTA2,ACTR3,BRAF,CDC42,ITGB3,ITGB6,PAK1,PIK3R1,PLCG1,RHOQ,RRAS2
42	Glioma Invasiveness Signaling	2.50E+00	8.11E-02	CDC42,ITGB3,PIK3R1,RHOQ,RRAS2,TIMP1
43	Hypoxia Signaling in the Cardiovascular System	2.50E+00	8.11E-02	P4HB,UBE2A,UBE2D1,UBE2E2,UBE2R2,VEGFA
44	Mismatch Repair in Eukaryotes	2.47E+00	1.88E-01	FEN1,PCNA,RFC4
45	P2Y Purigenic Receptor Signaling Pathway	2.47E+00	6.30E-02	GNG5,ITGB3,MYC,PIK3R1,PLCB1,PLCG1,PRKD1,RRAS2
46	dTMP De Novo Biosynthesis	2.44E+00	4.00E-01	DHFR2,TYMS
47	Endothelin-1 Signaling	2.42E+00	5.32E-02	BRAF,MYC,PIK3R1,PLA2G4A,PLA2G7,PLCB1,PLCG1,PLD3,PRKD1,RRAS2
48	ILK Signaling	2.39E+00	5.26E-02	ACTA2,CDC42,FLNB,ITGB3,ITGB6,MYC,PIK3R1,RHOQ,TMSB4Y,VEGFA
49	Role of BRCA1 in DNA Damage Response	2.34E+00	7.50E-02	HLTF,IFNG,PLK1,RBBP8,RFC4,SMARCB1
50	RAR Activation	2.32E+00	5.15E-02	GTF2H1,HLTF,PIK3R1,PNPLA4,PRKD1,RDH13,SMAD5,SMAD9,SMARCB1,VEGFA
51	STAT3 Pathway	2.31E+00	5.93E-02	BCL2,CXCR2,IL13RA1,IL1R1,MYC,PIM1,RRAS2,VEGFA
52	Prolactin Signaling	2.31E+00	7.41E-02	MYC,PIK3R1,PLCG1,PRKD1,PRLR,RRAS2
53	Paxillin Signaling	2.29E+00	6.48E-02	ACTA2,CDC42,ITGB3,ITGB6,PAK1,PIK3R1,RRAS2
54	Leukocyte Extravasation Signaling	2.28E+00	5.08E-02	ACTA2,CDC42,ITGB3,ITK,NCF2,PIK3R1,PLCG1,PRKD1,RDX,TIMP1
55	MSP-RON Signaling Pathway	2.28E+00	8.62E-02	ACTA2,IFNG,KLK2,MST1R,PIK3R1
56	Systemic Lupus Erythematosus Signaling	2.27E+00	4.80E-02	CBL,CD28,FCER1G,FCGR2B,PIK3R1,PIM2,PLCG1,PRPF4,RRAS2,SF3B4,TRGV9
57	Cdc42 Signaling	2.26E+00	5.39E-02	ACTR3,CDC42,FCER1G,HLA-DMA,HLA-DMB,ITK,PAK1,RASA1,TRGV9
58	VEGF Family Ligand-Receptor Interactions	2.23E+00	7.14E-02	PIK3R1,PLA2G4A,PLCG1,PRKD1,RRAS2,VEGFA
59	HGF Signaling	2.23E+00	6.31E-02	CDC42,ETS2,PAK1,PIK3R1,PLCG1,PRKD1,RRAS2
60	Allograft Rejection Signaling	2.18E+00	6.98E-02	CD28,FCER1G,HLA-DMA,HLA-DMB,IFNG,TRGV9
61	Antiproliferative Role of TOB in T Cell Signaling	2.17E+00	1.03E-01	CD28,FCER1G,SKP1,TRGV9
62	Thrombopoietin Signaling	2.12E+00	7.94E-02	MYC,PIK3R1,PLCG1,PRKD1,RRAS2

63	Fc Epsilon RI Signaling	2.10E+00	5.98E-02	FCER1A,FCER1G,PIK3R1,PLA2G4A,PLCG1,PRKD1,RRAS2
64	Signaling by Rho Family GTPases	2.08E+00	4.51E-02	ACTA2,ACTR3,ARHGEF18,CDC42,GNG5,NCF2,PAK1,PIK3R1,RDX,RHOQ,SEPTIN6
65	Mitotic Roles of Polo-Like Kinase	2.04E+00	7.58E-02	CDK1,FBXO5,KIF11,PLK1,SMC1A
66	BAG2 Signaling Pathway	2.02E+00	9.30E-02	BAG2,HSPA2,MYC,UBE2D1
67	ErbB4 Signaling	2.01E+00	7.46E-02	PIK3R1,PLCG1,PRKD1,PSEN1,RRAS2
68	G Beta Gamma Signaling	2.01E+00	5.74E-02	CACNA1C,CDC42,GNG5,PAK1,PLCG1,PRKD1,RRAS2
69	CCR5 Signaling in Macrophages	2.00E+00	6.38E-02	CACNA1C,FCER1G,GNG5,PLCG1,PRKD1,TRGV9
70	ErbB Signaling	2.00E+00	6.38E-02	CDC42,PAK1,PIK3R1,PLCG1,PRKD1,RRAS2
71	CCR3 Signaling in Eosinophils	1.97E+00	5.65E-02	GNG5,PAK1,PIK3R1,PLA2G4A,PLCB1,PRKD1,RRAS2
72	Glutathione Redox Reactions I	1.97E+00	1.25E-01	GPX1,GPX7,MGST1
73	Neuregulin Signaling	1.96E+00	6.25E-02	MYC,PIK3R1,PLCG1,PRKD1,PSEN1,RRAS2
74	14-3-3-mediated Signaling	1.92E+00	5.51E-02	CBL,PIK3R1,PLCB1,PLCG1,PRKD1,RRAS2,TUBA1B
75	Gαq Signaling	1.91E+00	5.06E-02	CDC42,GNG5,PIK3R1,PLCB1,PLCG1,PLD3,PRKD1,RHOQ
76	Estrogen Receptor Signaling	1.91E+00	3.96E-02	BCL2,CACNA1C,GNG5,MYC,PAK1,PCNA,PIK3R1,PLCB1,PLCG1,PRKD1,RRAS2,TRRAP,VEGFA
77	Ephrin B Signaling	1.89E+00	6.94E-02	CBL,CDC42,GNG5,ITSN1,PAK1
78	Caveolar-mediated Endocytosis Signaling	1.86E+00	6.85E-02	ACTA2,FLNB,ITGB3,ITGB6,ITSN1
79	Systemic Lupus Erythematosus In T Cell Signaling Pathway	1.85E+00	3.89E-02	CBL,CD28,CDC42,FCER1G,HLA-DMA,HLA-DMB,ICOS,PIK3R1,PLCG1,RDX,RHOQ,RRAS2,TRGV9
80	Axonal Guidance Signaling	1.85E+00	3.51E-02	ACTR3,CDC42,FES,GNG5,ITSN1,PAK1,PIK3R1,PLCB1,PLCG1,PLXND1,PRKD1,RASA1,RRAS2,SEMA4A,SRGAP1,TUBA1B,VEGFA
81	Neuroinflammation Signaling Pathway	1.84E+00	4.01E-02	BACE2,BCL2,HLA-DMA,HLA-DMB,IFNG,IL1R1,IRAK3,NCF2,PIK3R1,PLA2G4A,PLCG1,PSEN1
82	SAPK/JNK Signaling	1.84E+00	5.88E-02	CDC42,FCER1G,GNG5,PIK3R1,RRAS2,TRGV9
83	Erythropoietin Signaling	1.79E+00	6.58E-02	CBL,PIK3R1,PLCG1,PRKD1,RRAS2
84	GDNF Family Ligand-Receptor Interactions	1.79E+00	6.58E-02	CDC42,PIK3R1,PLCG1,RASA1,RRAS2
85	CXCR4 Signaling	1.78E+00	4.79E-02	CDC42,GNG5,PAK1,PIK3R1,PLCB1,PRKD1,RHOQ,RRAS2
86	PD-1, PD-L1 cancer immunotherapy pathway	1.76E+00	5.66E-02	CD28,HLA-DMA,HLA-DMB,IFNG,PIK3R1,PLCG1
87	Agrin Interactions at Neuromuscular Junction	1.75E+00	6.41E-02	ACTA2,CDC42,ITGB3,PAK1,RRAS2
88	Germ Cell-Sertoli Cell Junction Signaling	1.73E+00	4.68E-02	ACTA2,CDC42,JUP,PAK1,PIK3R1,RHOQ,RRAS2,TUBA1B
89	HIF1α Signaling	1.72E+00	4.39E-02	BRAF,HK2,HSPA2,NCF2,PIK3R1,PLCG1,PRKD1,RRAS2,VEGFA
90	Systemic Lupus Erythematosus In B Cell Signaling Pathway	1.72E+00	4.00E-02	BCL2,CARD11,CBL,FCGR2B,IFNG,MYC,PIK3R1,PIM2,PRKD1,RRAS2,TNFSF13
91	Methylthiopropionate Biosynthesis	1.71E+00	1.00E+00	ADI1
92	Hereditary Breast Cancer Signaling	1.71E+00	5.00E-02	CDK1,HLTF,PIK3R1,POLR2E,RFC4,RRAS2,SMARCB1
93	Renal Cell Carcinoma Signaling	1.71E+00	6.25E-02	CDC42,PAK1,PIK3R1,RRAS2,VEGFA
94	Role of MAPK Signaling in the Pathogenesis of Influenza	1.71E+00	6.25E-02	BCL2,IFNG,PLA2G4A,PLA2G7,RRAS2
95	CREB Signaling in Neurons	1.70E+00	4.35E-02	CACNA1C,GNG5,GRIA4,PIK3R1,PLCB1,PLCG1,POLR2E,PRKD1,RRAS2
96	LPS-stimulated MAPK Signaling	1.66E+00	6.10E-02	CDC42,PAK1,PIK3R1,PRKD1,RRAS2
97	mTOR Signaling	1.66E+00	4.29E-02	CDC42,PIK3R1,PLD3,PRKD1,RHOQ,RPS4X,RPSA,RRAS2,VEGFA
98	G Protein Signaling Mediated by Tubby	1.66E+00	9.68E-02	GNG5,PLCB1,PLCG1
99	Glucose and Glucose-1-phosphate Degradation	1.66E+00	1.67E-01	HK2,RGN
100	Acetone Degradation I (to Methylglyoxal)	1.66E+00	9.68E-02	CYP1B1,CYP2S1,PTGR2
101	BER pathway	1.66E+00	1.67E-01	FEN1,PCNA

102	Rac Signaling	1.66E+00	5.36E-02	ACTR3,CDC42,NCF2,PAK1,PIK3R1,RRAS2
103	Unfolded protein response	1.63E+00	7.14E-02	BCL2,EIF2AK3,HSPA2,P4HB
104	NF-κB Signaling	1.62E+00	4.47E-02	BRAF,CARD11,FCER1G,IL1R1,IRAK3,PIK3R1,RRAS2,TRGV9
105	Role of CHK Proteins in Cell Cycle Checkpoint Control	1.61E+00	7.02E-02	CDK1,PCNA,PLK1,RFC4
106	RhoGDI Signaling	1.61E+00	4.44E-02	ACTA2,ACTR3,ARHGEF18,CDC42,GNG5,PAK1,RDX,RHOQ
107	Ephrin Receptor Signaling	1.61E+00	4.44E-02	ACTR3,CDC42,GNG5,ITSN1,PAK1,RASA1,RRAS2,VEGFA
108	IL-4 Signaling	1.60E+00	5.88E-02	HLA-DMA,HLA-DMB,IL13RA1,PIK3R1,RRAS2
109	PDGF Signaling	1.59E+00	5.81E-02	MYC,PIK3R1,PLCG1,RASA1,RRAS2
110	Cancer Drug Resistance By Drug Efflux	1.59E+00	6.90E-02	BRAF,mir-181,PIK3R1,RRAS2
111	p38 MAPK Signaling	1.56E+00	5.08E-02	EEF2K,HMGN1,IL1R1,IRAK3,MYC,PLA2G4A
112	Cytotoxic T Lymphocyte-mediated Apoptosis of Target Cells	1.56E+00	8.82E-02	BCL2,FCER1G,TRGV9
113	Role of JAK2 in Hormone-like Cytokine Signaling	1.56E+00	8.82E-02	HLTF,PRLR,SH2B3
114	Role of Osteoblasts, Osteoclasts and Chondrocytes in Rheumatoid Arthritis	1.55E+00	4.09E-02	ACP5,BCL2,CBL,IFNG,IL1R1,ITGB3,PIK3R1,SMAD5,SMAD9
115	Semaphorin Signaling in Neurons	1.54E+00	6.67E-02	CDC42,FES,PAK1,RHOQ
116	CTLA4 Signaling in Cytotoxic T Lymphocytes	1.53E+00	5.62E-02	CD28,FCER1G,PIK3R1,PLCG1,TRGV9
117	DNA Methylation and Transcriptional Repression Signaling	1.52E+00	8.57E-02	H4C13,H4C9,RBBP4
118	Production of Nitric Oxide and Reactive Oxygen Species in Macrophages	1.51E+00	4.26E-02	CDC42,IFNG,IRF8,NCF2,PIK3R1,PLCG1,PRKD1,RHOQ
119	OX40 Signaling Pathway	1.51E+00	5.56E-02	BCL2,FCER1G,HLA-DMA,HLA-DMB,TRGV9
120	Synaptic Long Term Depression	1.50E+00	4.23E-02	CACNA1C,GRIA4,PLA2G4A,PLA2G7,PLCB1,PLCG1,PRKD1,RRAS2
121	Th17 Activation Pathway	1.49E+00	5.49E-02	FCER1G,IFNG,IL1R1,IRAK3,TRGV9
122	Aldosterone Signaling in Epithelial Cells	1.46E+00	4.43E-02	DNAJC22,DNAJC8,HSPA2,PIK3R1,PLCB1,PLCG1,PRKD1
123	Clathrin-mediated Endocytosis Signaling	1.45E+00	4.15E-02	ACTA2,ACTR3,CBL,CDC42,ITGB3,ITGB6,PIK3R1,VEGFA
124	ERK/MAPK Signaling	1.45E+00	4.15E-02	BRAF,ETS2,MYC,PAK1,PIK3R1,PLA2G4A,PLCG1,RRAS2
125	Regulation of Actin-based Motility by Rho	1.44E+00	5.32E-02	ACTA2,ACTR3,CDC42,PAK1,RHOQ
126	FAK Signaling	1.42E+00	5.26E-02	ACTA2,PAK1,PIK3R1,PLCG1,RRAS2
127	UDP-D-xylose and UDP-D-glucuronate Biosynthesis	1.42E+00	5.00E-01	UGDH
128	Palmitate Biosynthesis I (Animals)	1.42E+00	5.00E-01	OXSM
129	Sulfate Activation for Sulfonation	1.42E+00	5.00E-01	PAPSS2
130	Fatty Acid Biosynthesis Initiation II	1.42E+00	5.00E-01	OXSM
131	Natural Killer Cell Signaling	1.41E+00	4.06E-02	CDC42,FCER1G,HSPA2,IFNG,PAK1,PIK3R1,PLCG1,RRAS2
132	Communication between Innate and Adaptive Immune Cells	1.41E+00	5.21E-02	CD28,FCER1G,IFNG,TNFSF13,TRGV9
133	TGF-β Signaling	1.41E+00	5.21E-02	BCL2,CDC42,RRAS2,SMAD5,SMAD9
134	Eicosanoid Signaling	1.41E+00	6.06E-02	PLA2G4A,PLA2G7,PTGDS,TBXAS1
135	Antigen Presentation Pathway	1.40E+00	7.69E-02	HLA-DMA,HLA-DMB,IFNG
136	Gap Junction Signaling	1.40E+00	4.04E-02	ACTA2,GRIA4,PIK3R1,PLCB1,PLCG1,PRKD1,RRAS2,TUBA1B
137	Synaptic Long Term Potentiation	1.40E+00	4.65E-02	CACNA1C,GRIA4,PLCB1,PLCG1,PRKD1,RRAS2
138	p70S6K Signaling	1.40E+00	4.65E-02	EEF2K,PIK3R1,PLCB1,PLCG1,PRKD1,RRAS2
139	HMGB1 Signaling	1.37E+00	4.24E-02	CDC42,IFNG,IL1R1,PIK3R1,RHOQ,RRAS2,TNFSF13
140	Glioblastoma Multiforme Signaling	1.37E+00	4.24E-02	CDC42,MYC,PIK3R1,PLCB1,PLCG1,RHOQ,RRAS2
141	Senescence Pathway	1.37E+00	3.64E-02	ASXL3,BRAF,CACNA1C,CDK1,ETS2,PIK3R1,RRAS2,SMAD5,SMAD9,YPEL3

142	Adipogenesis pathway	1.33E+00	4.48E-02	DGKD,GTF2H1,RBBP4,SAP30L,SMAD5,SMAD9
143	Neuropathic Pain Signaling In Dorsal Horn Neurons	1.33E+00	4.95E-02	GRIA4,PIK3R1,PLCB1,PLCG1,PRKD1
144	Retinol Biosynthesis	1.32E+00	7.14E-02	DDHD2,PNPLA4,RDH13
145	Insulin Secretion Signaling Pathway	1.32E+00	3.70E-02	CACNA1C,EIF2AK3,EIF2S1,PC,PIK3R1,PLCB1,PLCG1,PRKD1,PRLR
	**Data2**			
**S.No.**	**Ingenuity Canonical Pathways**	**-log(p-value)**	**Ratio**	**Molecules**
1	Atherosclerosis Signaling	4.53E+00	7.09E-02	ALOX15,CCL2,CCR2,CCR3,CXCL8,CXCR4,PLAAT2,SERPINA1,TNFRSF12A
2	Granulocyte Adhesion and Diapedesis	3.38E+00	5.00E-02	CCL2,CCL23,CCL3,CCL3L3,CCL4,CLDN5,CXCL8,CXCR4,MMP19
3	Agranulocyte Adhesion and Diapedesis	3.16E+00	4.66E-02	CCL2,CCL23,CCL3,CCL3L3,CCL4,CLDN5,CXCL8,CXCR4,MMP19
4	Gαi Signaling	3.03E+00	5.60E-02	ADCY4,ADORA3,CHRM4,CNR2,P2RY14,RGS12,RGS14
5	Breast Cancer Regulation by Stathmin1	2.93E+00	2.88E-02	ADGRE1,ADORA3,ARHGEF4,C3AR1,CCR2,CCR3,CHRM4,CNR2,E2F2,GPR141,GPR34,GPR87,P2RY14,P2RY2,PIK3R6,PTGDR2,TACR2
6	Differential Regulation of Cytokine Production in Macrophages and T Helper Cells by IL-17A and IL-17F	2.88E+00	1.67E-01	CCL2,CCL3,CCL4
7	Role of Hypercytokinemia/hyperchemokinemia in the Pathogenesis of Influenza	2.71E+00	9.30E-02	CCL2,CCL3,CCL4,CXCL8
8	G-Protein Coupled Receptor Signaling	2.67E+00	3.68E-02	ADCY4,ADORA3,CHRM4,CNR2,DUSP1,P2RY14,PDE2A,PIK3R6,RGS12,RGS14
9	cAMP-mediated signaling	2.66E+00	3.95E-02	ADCY4,ADORA3,CHRM4,CNR2,DUSP1,P2RY14,PDE2A,RGS12,RGS14
10	Coronavirus Pathogenesis Pathway	2.57E+00	4.67E-02	CCL2,CXCL8,E2F2,FOS,PTGS2,RPS4Y1,RPS4Y2
11	Differential Regulation of Cytokine Production in Intestinal Epithelial Cells by IL-17A and IL-17F	2.56E+00	1.30E-01	CCL2,CCL3,CCL4
12	Chemokine Signaling	2.50E+00	6.25E-02	CCL2,CCL4,CCR3,CXCR4,FOS
13	Role of IL-17A in Arthritis	2.35E+00	7.41E-02	CCL2,CXCL8,PIK3R6,PTGS2
14	Pathogenesis of Multiple Sclerosis	2.29E+00	2.22E-01	CCL3,CCL4
15	Endocannabinoid Neuronal Synapse Pathway	2.28E+00	4.69E-02	ADCY4,CACNG6,CNR2,FAAH,MGLL,PTGS2
16	MSP-RON Signaling Pathway	2.24E+00	6.90E-02	CCL2,CCR2,IL3RA,PIK3R6
17	Communication between Innate and Adaptive Immune Cells	2.17E+00	5.21E-02	CCL3,CCL3L3,CCL4,CXCL8,IGHG1
18	Eicosanoid Signaling	2.04E+00	6.06E-02	ALOX15,LTC4S,PLAAT2,PTGS2
19	Sorbitol Degradation I	1.91E+00	1.00E+00	SORD
20	GDNF Family Ligand-Receptor Interactions	1.84E+00	5.26E-02	FOS,GFRA1,GFRA4,PIK3R6
21	Role of IL-17F in Allergic Inflammatory Airway Diseases	1.82E+00	7.14E-02	CCL2,CCL4,CXCL8
22	Thyroid Cancer Signaling	1.78E+00	5.06E-02	CXCL8,CXCR4,FOS,PIK3R6
23	IL-17 Signaling	1.76E+00	5.00E-02	CCL2,CXCL8,PIK3R6,PTGS2
24	Triacylglycerol Degradation	1.69E+00	6.38E-02	DDHD2,FAAH,MGLL
25	CCR5 Signaling in Macrophages	1.53E+00	4.26E-02	CACNG6,CCL3,CCL4,FOS
26	p53 Signaling	1.48E+00	4.08E-02	GADD45G,PIK3R6,PMAIP1,SFN
27	Opioid Signaling Pathway	1.46E+00	2.83E-02	ADCY4,CACNG6,FOS,FOSB,RGS1,RGS12,RGS14
28	VEGF Signaling	1.46E+00	4.04E-02	EIF1AY,PIK3R6,SFN,SH2D2A
29	Anandamide Degradation	1.44E+00	3.33E-01	FAAH
30	Biotin-carboxyl Carrier Protein Assembly	1.44E+00	3.33E-01	ACACB
31	IL-17A Signaling in Gastric Cells	1.42E+00	8.00E-02	CXCL8,FOS
32	Relaxin Signaling	1.41E+00	3.33E-02	ADCY4,FOS,PDE2A,PIK3R6,RLN1

33	IGF-1 Signaling	1.40E+00	3.85E-02	FOS,IGFBP2,PIK3R6,SFN
34	Gustation Pathway	1.37E+00	3.25E-02	ADCY4,CACNG6,P2RY14,P2RY2,PDE2A
35	CD40 Signaling	1.33E+00	4.62E-02	FOS,PIK3R6,PTGS2
36	Osteoarthritis Pathway	1.32E+00	2.84E-02	ALPL,CXCL8,DDIT4,GDF5,NAMPT,PTGS2
