# Supplementary material for: Association between Arsenic Level, Gene Expression in Asian Population, and In Vitro Carcinogenic Bladder Tumor
Source: Oxid Med Cell Longev. 2022 Jan 7;2022:3459855. doi: 10.1155/2022/3459855 (PMC8760535; doi:10.1155/2022/3459855)
Supplement: Supplementary 5 — Table S4: significant pathways in Data1 and Data2 (low, medium, and high concentrations) and the overlap between various combinations (highlighted in green). [file 3459855.f5.pdf]

Table S4. Significant pathways in Data-1 and Data-2 (low, medium, and high concentrations) and the overlap between various combinations (highlighted in green).

Data1

S.No.	Symbol	Name	t.stat	p.value	X.log10.p.	FDR	entrezgene	AffyID	HGNC
1	KDM6A	lysine demethylase 6A	24.622	4.94E-20	19.307	2.47E-16	7403	7403_at	HGNC:12637
2	KDM5C	lysine demethylase 5C	14.551	2.68E-14	13.572	6.70E-11	8242	8242_at	HGNC:11114
3	TMSB4Y	thymosin beta 4 Y-linked	-13.063	3.47E-13	12.459	5.79E-10	9087	9087_at	HGNC:11882
4	PRKY	protein kinase Y-linked (pseudogene)	-11.806	3.60E-12	11.444	4.50E-09	5616	5616_at	HGNC:9444
5	ZFX	zinc finger protein X-linked	11.047	1.61E-11	10.794	1.61E-08	7543	7543_at	HGNC:12869
6	EIF1AX	eukaryotic translation initiation factor 1A X-linked	10.8	2.66E-11	10.575	2.21E-08	1964	1964_at	HGNC:3250
7	SMC1A	structural maintenance of chromosomes 1A	7.9299	1.59E-08	7.7977	1.14E-05	8243	8243_at	HGNC:11111
8	PRKX	protein kinase X-linked	7.3111	7.28E-08	7.1376	4.55E-05	5613	5613_at	HGNC:9441
9	DDX3X	DEAD-box helicase 3 X-linked	7.0964	1.25E-07	6.9037	6.93E-05	1654	1654_at	HGNC:2745
10	SCARNA9L	small Cajal body-specific RNA 9 like	6.0741	1.74E-06	5.7593	0.00087011	100158262	100158262_at	HGNC:33559
11	RPS4X	ribosomal protein S4 X-linked	6.0184	2.02E-06	5.6956	0.00091597	6191	6191_at	HGNC:10424
12	TXLNG	taxilin gamma	5.9777	2.24E-06	5.649	0.00093476	55787	55787_at	HGNC:18578
13	PUDP	pseudouridine 5'-phosphatase	5.6264	5.69E-06	5.2447	0.0021892	8226	8226_at	HGNC:16818
14	MLLT3	MLLT3 super elongation complex subunit	5.4228	9.80E-06	5.0087	0.0034999	4300	4300_at	HGNC:7136
15	SEPTIN6	septin 6	5.2857	1.41E-05	4.8494	0.0047143	23157	23157_at	HGNC:15848
16	PNPLA4	patatin like phospholipase domain containing 4	5.2284	1.65E-05	4.7826	0.00494	8228	8228_at	HGNC:24887
17	PIM1	Pim-1 proto-oncogene, serine/threonine kinase	5.2216	1.68E-05	4.7747	0.00494	5292	5292_at	HGNC:8986
18	CA5B	carbonic anhydrase 5B	5.0924	2.38E-05	4.6241	0.0065992	11238	11238_at	HGNC:1378
19	PID1	phosphotyrosine interaction domain containing 1	-5.052	2.65E-05	4.577	0.006969	55022	55022_at	HGNC:26084
20	CYP2S1	cytochrome P450 family 2 subfamily S member 1	-4.9809	3.21E-05	4.494	0.008014	29785	29785_at	HGNC:15654
21	HGSNAT	heparan-alpha-glucosaminide N-acetyltransferase	-4.8025	5.18E-05	4.2858	0.012328	138050	138050_at	HGNC:26527

22	ICOS	inducible T cell costimulator	4.7815	5.48E-05	4.2612	0.012452	29851	29851_at	HGNC:5351
23	SASH1	SAM and SH3 domain containing 1	-4.7326	6.25E-05	4.2041	0.013584	23328	23328_at	HGNC:19182
24	IGSF6	immunoglobulin superfamily member 6	-4.6523	7.75E-05	4.1104	0.016152	10261	10261_at	HGNC:5953
25	ZRSR2	zinc finger CCCH-type, RNA binding motif and serine/arginine rich 2	4.5985	8.96E-05	4.0478	0.017913	8233	8233_at	HGNC:23019
26	IGLV7-46	immunoglobulin lambda variable 7-46 (gene/pseudogene)	4.5648	9.81E-05	4.0084	0.018858	28775	28775_at	HGNC:5930
27	LPIN2	lipin 2	4.3953	0.00015446	3.8112	0.027611	9663	9663_at	HGNC:14450
28	CLMN	calmin	-4.3949	0.00015465	3.8106	0.027611	79789	79789_at	HGNC:19972
29	TGFBI	transforming growth factor beta induced	-4.3764	0.00016248	3.7892	0.028008	7045	7045_at	HGNC:11771
30	BCL2	BCL2 apoptosis regulator	4.3115	0.00019329	3.7138	0.03188	596	596_at	HGNC:990
31	FCER1A	Fc fragment of IgE receptor Ia	-4.303	0.0001977	3.704	0.03188	2205	2205_at	HGNC:3609
32	GPR15	G protein-coupled receptor 15	-4.262	0.00022056	3.6565	0.033716	2838	2838_at	HGNC:4469
33	SCPEP1	serine carboxypeptidase 1	-4.2554	0.00022452	3.6487	0.033716	59342	59342_at	HGNC:29507
34	HNMT	histamine N-methyltransferase	-4.2475	0.00022932	3.6396	0.033716	3176	3176_at	HGNC:5028
35	PLA2G4A	phospholipase A2 group IVA	-4.1982	0.00026151	3.5825	0.037351	5321	5321_at	HGNC:9035
36	LDLRAD3	low density lipoprotein receptor class A domain containing 3	-4.0888	0.00034976	3.4562	0.047278	143458	143458_at	HGNC:27046
37	CPM	carboxypeptidase M	-4.0886	0.00034992	3.456	0.047278	1368	1368_at	HGNC:2311
38	GLCCI1	glucocorticoid induced 1	4.0512	0.00038643	3.4129	0.050836	113263	113263_at	HGNC:18713
39	SLC2A9	solute carrier family 2 member 9	-3.9919	0.00045211	3.3448	0.057952	56606	56606_at	HGNC:13446
40	UBE2E2	ubiquitin conjugating enzyme E2 E2	-3.9608	0.00049092	3.309	0.061353	7325	7325_at	HGNC:12478
41	TNFSF13	TNF superfamily member 13	-3.9216	0.00054434	3.2641	0.06637	8741	8741_at	HGNC:11928
42	MS4A6A	membrane spanning 4-domains A6A	-3.8984	0.00057868	3.2376	0.068877	64231	64231_at	HGNC:13375
43	KIAA0930	KIAA0930	-3.8852	0.00059918	3.2224	0.069658	23313	23313_at	HGNC:1314
44	LAX1	lymphocyte transmembrane adaptor 1	3.8556	0.00064771	3.1886	0.073009	54900	54900_at	HGNC:26005
45	EPB41L3	erythrocyte membrane protein band 4.1 like 3	-3.846	0.00066419	3.1777	0.073009	23136	23136_at	HGNC:3380

46	CCDC88A	coiled-coil domain containing 88A	-3.8317	0.0006896	3.1614	0.073009	55704	55704_at	HGNC:25523
47	IDH1	isocitrate dehydrogenase (NADP(+)) 1	-3.8086	0.00073267	3.1351	0.073009	3417	3417_at	HGNC:5382
48	SLC36A4	solute carrier family 36 member 4	-3.805	0.00073975	3.1309	0.073009	120103	120103_at	HGNC:19660
49	DPP4	dipeptidyl peptidase 4	3.8049	0.00073993	3.1308	0.073009	1803	1803_at	HGNC:3009
50	ANKRD50	ankyrin repeat domain 50	-3.8023	0.00074491	3.1279	0.073009	57182	57182_at	HGNC:29223
51	LACC1	laccase domain containing 1	-3.799	0.00075143	3.1241	0.073009	144811	144811_at	HGNC:26789
52	HLA-DPB2	major histocompatibility complex, class II, DP beta 2 (pseudogene)	-3.7949	0.00075945	3.1195	0.073009	3116	3116_at	HGNC:4941
53	OASL	2'-5'-oligoadenylate synthetase like	3.7782	0.00079339	3.1005	0.074833	8638	8638_at	HGNC:8090
54	GASK1B	golgi associated kinase 1B	-3.7704	0.0008099	3.0916	0.074976	51313	51313_at	HGNC:25312
55	SERPINB8	serpin family B member 8	-3.75	0.00085416	3.0685	0.077094	5271	5271_at	HGNC:8952
56	SETD7	SET domain containing 7, histone lysine methyltransferase	-3.7458	0.00086362	3.0637	0.077094	80854	80854_at	HGNC:30412
57	RTN1	reticulon 1	-3.7245	0.00091304	3.0395	0.078809	6252	6252_at	HGNC:10467
58	OGFRL1	opioid growth factor receptor like 1	-3.7239	0.00091436	3.0389	0.078809	79627	79627_at	HGNC:21378
59	CPVL	carboxypeptidase vitellogenic like	-3.6851	0.0010118	2.9949	0.084715	54504	54504_at	HGNC:14399
60	DLEU1	deleted in lymphocytic leukemia 1	3.6832	0.0010168	2.9928	0.084715	10301	10301_at	HGNC:13747
61	SLC31A1	solute carrier family 31 member 1	-3.6717	0.0010477	2.9798	0.08531	1317	1317_at	HGNC:11016
62	IRAK3	interleukin 1 receptor associated kinase 3	-3.6679	0.0010581	2.9755	0.08531	11213	11213_at	HGNC:17020
63	C3orf14	chromosome 3 open reading frame 14	-3.6478	0.0011148	2.9528	0.08762	57415	57415_at	HGNC:25024
64	CTSO	cathepsin O	-3.6429	0.0011292	2.9472	0.08762	1519	1519_at	HGNC:2542
65	GNG5	G protein subunit gamma 5	-3.6394	0.0011393	2.9434	0.08762	2787	2787_at	HGNC:4408
66	SYAP1	synapse associated protein 1	3.6324	0.0011604	2.9354	0.087895	94056	94056_at	HGNC:16273
67	PLXDC2	plexin domain containing 2	-3.5342	0.001496	2.8251	0.11087	84898	84898_at	HGNC:21013

68	ENTPD1	ectonucleoside triphosphate diphosphohydrolase 1	-3.5172	0.0015628	2.8061	0.11087	953	953_at	HGNC:3363
69	CREG1	cellular repressor of E1A stimulated genes 1	-3.5163	0.0015662	2.8051	0.11087	8804	8804_at	HGNC:2351
70	PRCP	prolylcarboxypeptidase	-3.5156	0.0015692	2.8043	0.11087	5547	5547_at	HGNC:9344
71	GLCE	glucuronic acid epimerase	-3.5143	0.0015747	2.8028	0.11087	26035	26035_at	HGNC:17855
72	PIM2	Pim-2 proto-oncogene, serine/threonine kinase	3.49	0.001676	2.7757	0.11636	11040	11040_at	HGNC:8987
73	FAM118A	family with sequence similarity 118 member A	-3.4808	0.0017162	2.7654	0.11752	55007	55007_at	HGNC:1313
74	CDK6	cyclin dependent kinase 6	3.4464	0.0018745	2.7271	0.12576	1021	1021_at	HGNC:1777
75	SHTN1	shootin 1	-3.4438	0.0018868	2.7243	0.12576	57698	57698_at	HGNC:29319
76	NAPRT	nicotinate phosphoribosyltransferase	-3.4221	0.0019947	2.7001	0.1312	93100	93100_at	HGNC:30450
77	SMAD5	SMAD family member 5	-3.4148	0.0020321	2.692	0.13193	4090	4090_at	HGNC:6771
78	CD300LB	CD300 molecule like family member b	-3.4006	0.0021071	2.6763	0.13309	124599	124599_at	HGNC:30811
79	CEBPG	CCAAT enhancer binding protein gamma	-3.4005	0.0021079	2.6762	0.13309	1054	1054_at	HGNC:1837
80	RNASE6	ribonuclease A family member k6	-3.3964	0.0021298	2.6717	0.13309	6039	6039_at	HGNC:10048
81	MPZL2	myelin protein zero like 2	-3.3816	0.0022117	2.6553	0.1365	10205	10205_at	HGNC:3496
82	GBGT1	globoside alpha-1,3-N-acetylgalactosaminyltransferase 1 (FORS blood group)	-3.3698	0.0022793	2.6422	0.13706	26301	26301_at	HGNC:20460
83	ABITRAM	actin binding transcription modulator	-3.3626	0.0023214	2.6343	0.13706	54942	54942_at	HGNC:1364
84	QARS1	glutaminyl-tRNA synthetase 1	-3.3601	0.002336	2.6315	0.13706	5859	5859_at	HGNC:9751
85	FAR1	fatty acyl-CoA reductase 1	-3.3581	0.0023479	2.6293	0.13706	84188	84188_at	HGNC:26222
86	FAH	fumarylacetoacetate hydrolase	-3.3466	0.0024176	2.6166	0.13706	2184	2184_at	HGNC:3579
87	SLC49A4	solute carrier family 49 member 4	-3.3451	0.002427	2.6149	0.13706	84925	84925_at	HGNC:16628
88	HLA-DMA	major histocompatibility complex, class II, DM alpha	-3.3421	0.0024456	2.6116	0.13706	3108	3108_at	HGNC:4934
89	PTGDR2	prostaglandin D2 receptor 2	-3.3409	0.002453	2.6103	0.13706	11251	11251_at	HGNC:4502

90	CST2	cystatin SA	-3.3384	0.0024683	2.6076	0.13706	1470	1470_at	HGNC:2474
91	FRY	FRY microtubule binding protein	-3.3342	0.002495	2.6029	0.13706	10129	10129_at	HGNC:20367
92	GPX7	glutathione peroxidase 7	3.3261	0.0025469	2.594	0.13715	2882	2882_at	HGNC:4559
93	STS	steroid sulfatase	3.3232	0.0025652	2.5909	0.13715	412	412_at	HGNC:11425
94	PLCB1	phospholipase C beta 1	-3.3211	0.002579	2.5886	0.13715	23236	23236_at	HGNC:15917
95	SLC43A3	solute carrier family 43 member 3	-3.3004	0.0027179	2.5658	0.14165	29015	29015_at	HGNC:17466
96	PLA2G15	phospholipase A2 group XV	-3.3001	0.0027202	2.5654	0.14165	23659	23659_at	HGNC:17163
97	JDP2	Jun dimerization protein 2	-3.2842	0.0028317	2.548	0.14594	122953	122953_at	HGNC:17546
98	NAAA	N-acylethanolamine acid amidase	-3.2791	0.0028681	2.5424	0.1463	27163	27163_at	HGNC:736
99	RBM3	RNA binding motif protein 3	-3.2732	0.0029115	2.5359	0.14702	5935	5935_at	HGNC:9900
100	MAN2B1	mannosidase alpha class 2B member 1	-3.2668	0.0029585	2.5289	0.1479	4125	4125_at	HGNC:6826
101	CTTNBP2NL	CTTNBP2 N-terminal like	-3.2581	0.0030239	2.5194	0.14842	55917	55917_at	HGNC:25330
102	ITK	IL2 inducible T cell kinase	3.2576	0.0030283	2.5188	0.14842	3702	3702_at	HGNC:6171
103	CD160	CD160 molecule	3.2517	0.0030732	2.5124	0.14895	11126	11126_at	HGNC:17013
104	PLPP1	phospholipid phosphatase 1	3.2484	0.0030988	2.5088	0.14895	8611	8611_at	HGNC:9228
105	PSEN1	presenilin 1	-3.2421	0.0031489	2.5018	0.14992	5663	5663_at	HGNC:9508
106	GAPT	GRB2 binding adaptor protein, transmembrane	-3.215	0.0033708	2.4723	0.1576	202309	202309_at	HGNC:26588
107	FAM13A	family with sequence similarity 13 member A	3.2134	0.0033843	2.4705	0.1576	10144	10144_at	HGNC:19367
108	CD33	CD33 molecule	-3.2109	0.0034048	2.4679	0.1576	945	945_at	HGNC:1659
109	CFD	complement factor D	-3.1772	0.0037053	2.4312	0.16965	1675	1675_at	HGNC:2771
110	SLC24A4	solute carrier family 24 member 4	-3.1737	0.0037378	2.4274	0.16965	123041	123041_at	HGNC:10978
111	CNTLN	centlein	-3.17	0.0037719	2.4234	0.16965	54875	54875_at	HGNC:23432
112	CBL	Cbl proto-oncogene	-3.167	0.003801	2.4201	0.16965	867	867_at	HGNC:1541
113	CD93	CD93 molecule	-3.146	0.0040054	2.3974	0.17719	22918	22918_at	HGNC:15855
114	HLA-DMB	major histocompatibility complex, class II, DM beta	-3.142	0.0040452	2.3931	0.17733	3109	3109_at	HGNC:4935
115	MGST1	microsomal glutathione S-transferase 1	-3.1372	0.0040934	2.3879	0.17733	4257	4257_at	HGNC:7061
116	NUDT16P1	nudix hydrolase 16 pseudogene 1	-3.1328	0.0041393	2.3831	0.17733	152195	152195_at	HGNC:27189

117	FRG1BP	FSHD region gene 1 family member B, pseudogene	-3.1299	0.0041693	2.3799	0.17733	284802	284802_at	HGNC:15792
118	SNORD116-29	small nucleolar RNA, C/D box 116-29	3.1283	0.0041859	2.3782	0.17733	100033821	100033821_at	HGNC:33348
119	GPR160	G protein-coupled receptor 160	-3.1208	0.0042645	2.3701	0.17915	26996	26996_at	HGNC:23693
120	FGL2	fibrinogen like 2	-3.1109	0.0043705	2.3595	0.18029	10875	10875_at	HGNC:3696
121	GFI1	growth factor independent 1 transcriptional repressor	3.1097	0.0043838	2.3581	0.18029	2672	2672_at	HGNC:4237
122	CD1D	CD1d molecule	-3.1082	0.0043999	2.3566	0.18029	912	912_at	HGNC:1637
123	TFEC	transcription factor EC	-3.0863	0.0046456	2.333	0.18786	22797	22797_at	HGNC:11754
124	ARID5B	AT-rich interaction domain 5B	3.0837	0.0046752	2.3302	0.18786	84159	84159_at	HGNC:17362
125	SNORD14E	small nucleolar RNA, C/D box 14E	3.0816	0.0046998	2.3279	0.18786	85391	85391_at	HGNC:30354
126	IRF8	interferon regulatory factor 8	-3.0786	0.004735	2.3247	0.18786	3394	3394_at	HGNC:5358
127	CISH	cytokine inducible SH2 containing protein	3.0678	0.0048621	2.3132	0.19138	1154	1154_at	HGNC:1984
128	TBXAS1	thromboxane A synthase 1	-3.0412	0.0051925	2.2846	0.20213	6916	6916_at	HGNC:11609
129	FES	FES proto-oncogene, tyrosine kinase	-3.0394	0.0052159	2.2827	0.20213	2242	2242_at	HGNC:3657
130	CRTAP	cartilage associated protein	-3.0303	0.0053343	2.2729	0.20512	10491	10491_at	HGNC:2379
131	PAK1	p21 (RAC1) activated kinase 1	-3.015	0.0055374	2.2567	0.21131	5058	5058_at	HGNC:8590
132	PCTP	phosphatidylcholine transfer protein	-3.0062	0.0056594	2.2472	0.21262	58488	58488_at	HGNC:8752
133	PLD3	phospholipase D family member 3	-3.0037	0.0056936	2.2446	0.21262	23646	23646_at	HGNC:17158
134	GBP5	guanylate binding protein 5	3.0033	0.0056995	2.2442	0.21262	115362	115362_at	HGNC:19895
135	APLP2	amyloid beta precursor like protein 2	-2.9949	0.0058177	2.2352	0.21428	334	334_at	HGNC:598
136	TSHZ3	teashirt zinc finger homeobox 3	-2.9941	0.0058297	2.2344	0.21428	57616	57616_at	HGNC:30700
137	MIS18BP1	MIS18 binding protein 1	-2.9651	0.0062571	2.2036	0.22832	55320	55320_at	HGNC:20190
138	TYMS	thymidylate synthetase	2.9533	0.0064411	2.191	0.23064	7298	7298_at	HGNC:12441
139	KCTD3	potassium channel tetramerization domain containing 3	-2.9526	0.0064509	2.1904	0.23064	51133	51133_at	HGNC:21305

140	PDIA4	protein disulfide isomerase family A member 4	2.9521	0.0064591	2.1898	0.23064	9601	9601_at	HGNC:30167
141	PGAP1	post-GPI attachment to proteins inositol deacylase 1	2.9438	0.0065916	2.181	0.23217	80055	80055_at	HGNC:25712
142	CD38	CD38 molecule	2.9436	0.006595	2.1808	0.23217	952	952_at	HGNC:1667
143	SULF2	sulfatase 2	-2.9323	0.0067787	2.1689	0.23697	55959	55959_at	HGNC:20392
144	DAPK1	death associated protein kinase 1	-2.917	0.0070349	2.1527	0.24422	1612	1612_at	HGNC:2674
145	SLC16A1	solute carrier family 16 member 1	2.9115	0.0071301	2.1469	0.24582	6566	6566_at	HGNC:10922
146	SERPINB2	serpin family B member 2	-2.9073	0.0072033	2.1425	0.24664	5055	5055_at	HGNC:8584
147	NUP210	nucleoporin 210	2.902	0.0072959	2.1369	0.24811	23225	23225_at	HGNC:30052
148	GRK3	G protein-coupled receptor kinase 3	-2.8968	0.0073874	2.1315	0.24952	157	157_at	HGNC:290
149	FUCA1	alpha-L-fucosidase 1	-2.8907	0.0074988	2.125	0.25159	2517	2517_at	HGNC:4006
150	SLAMF6	SLAM family member 6	2.878	0.0077308	2.1118	0.25764	114836	114836_at	HGNC:21392
151	ZNF516	zinc finger protein 516	-2.8722	0.0078414	2.1056	0.25847	9658	9658_at	HGNC:28990
152	PRLR	prolactin receptor	-2.8712	0.0078591	2.1046	0.25847	5618	5618_at	HGNC:9446
153	CD86	CD86 molecule	-2.8664	0.007952	2.0995	0.25982	942	942_at	HGNC:1705
154	SPECC1	sperm antigen with calponin homology and coiled-coil domains 1	-2.8593	0.0080889	2.0921	0.26184	92521	92521_at	HGNC:30615
155	CD27	CD27 molecule	2.8563	0.0081478	2.089	0.26184	939	939_at	HGNC:11922
156	TPRA1	transmembrane protein adipocyte associated 1	-2.8551	0.0081711	2.0877	0.26184	131601	131601_at	HGNC:30413
157	SKAP2	src kinase associated phosphoprotein 2	-2.8516	0.0082399	2.0841	0.26236	8935	8935_at	HGNC:15687
158	CCNA2	cyclin A2	2.8479	0.0083128	2.0803	0.26254	890	890_at	HGNC:1578
159	GPR174	G protein-coupled receptor 174	2.8446	0.0083803	2.0767	0.26254	84636	84636_at	HGNC:30245
160	RHOH	ras homolog family member H	2.8428	0.0084168	2.0749	0.26254	399	399_at	HGNC:686
161	IL13RA1	interleukin 13 receptor subunit alpha 1	-2.8357	0.0085596	2.0675	0.26254	3597	3597_at	HGNC:5974
162	CARD8	caspase recruitment domain family member 8	2.8346	0.0085842	2.0663	0.26254	22900	22900_at	HGNC:17057
163	TRAV19	T cell receptor alpha variable 19	2.8316	0.0086458	2.0632	0.26254	28664	28664_at	HGNC:12115
164	SIRPA	signal regulatory protein alpha	-2.8304	0.0086693	2.062	0.26254	140885	140885_at	HGNC:9662
165	DOCK7	dedicator of cytokinesis 7	-2.8292	0.0086957	2.0607	0.26254	85440	85440_at	HGNC:19190

166	ALDH2	aldehyde dehydrogenase 2 family member	-2.8281	0.0087181	2.0596	0.26254	217	217_at	HGNC:404
167	MAP3K3	mitogen-activated protein kinase kinase kinase 3	-2.8245	0.0087942	2.0558	0.26325	4215	4215_at	HGNC:6855
168	CPA3	carboxypeptidase A3	-2.8215	0.0088575	2.0527	0.26356	1359	1359_at	HGNC:2298
169	GAS7	growth arrest specific 7	-2.8155	0.0089853	2.0465	0.26578	8522	8522_at	HGNC:4169
170	CLEC10A	C-type lectin domain containing 10A	-2.8088	0.0091313	2.0395	0.26778	10462	10462_at	HGNC:16916
171	SNORA36A	small nucleolar RNA, H/ACA box 36A	2.8073	0.009163	2.038	0.26778	677817	677817_at	HGNC:32628
172	NCF2	neutrophil cytosolic factor 2	-2.8031	0.009257	2.0335	0.26778	4688	4688_at	HGNC:7661
173	FHL3	four and a half LIM domains 3	-2.7987	0.0093539	2.029	0.26778	2275	2275_at	HGNC:3704
174	GPRC5D	G protein-coupled receptor class C group 5 member D	2.7981	0.0093673	2.0284	0.26778	55507	55507_at	HGNC:13310
175	SLC25A24	solute carrier family 25 member 24	-2.7978	0.0093741	2.0281	0.26778	29957	29957_at	HGNC:20662
176	ATP6V1B2	ATPase H+ transporting V1 subunit B2	-2.7952	0.0094314	2.0254	0.26788	526	526_at	HGNC:854
177	ACSS2	acyl-CoA synthetase short chain family member 2	-2.7903	0.0095422	2.0204	0.26884	55902	55902_at	HGNC:15814
178	KYNU	kynureninase	-2.7869	0.0096217	2.0167	0.26884	8942	8942_at	HGNC:6469
179	CLIC2	chloride intracellular channel 2	-2.7867	0.0096263	2.0165	0.26884	1193	1193_at	HGNC:2063
180	HOPX	HOP homeobox	2.7831	0.0097093	2.0128	0.26965	84525	84525_at	HGNC:24961
181	PCED1B	PC-esterase domain containing 1B	2.7791	0.0098015	2.0087	0.26966	91523	91523_at	HGNC:28255
182	ARRDC3	arrestin domain containing 3	2.7777	0.0098339	2.0073	0.26966	57561	57561_at	HGNC:29263
183	OFD1	OFD1 centriole and centriolar satellite protein	2.7761	0.0098715	2.0056	0.26966	8481	8481_at	HGNC:2567
184	GRAMD4	GRAM domain containing 4	-2.7699	0.010019	1.9992	0.27145	23151	23151_at	HGNC:29113
185	RPL22L1	ribosomal protein L22 like 1	2.7688	0.010046	1.998	0.27145	200916	200916_at	HGNC:27610
186	SNORD116-26	small nucleolar RNA, C/D box 116-26	2.7623	0.010201	1.9914	0.27415	100033438	100033438_at	HGNC:33092
187	SNORD116-14	small nucleolar RNA, C/D box 116-14	2.7581	0.010304	1.987	0.27546	100033426	100033426_at	HGNC:33080
188	B3GALT2	beta-1,3-galactosyltransferase 2	-2.7533	0.010423	1.982	0.27714	8707	8707_at	HGNC:917

189	H4C9	H4 clustered histone 9	2.7346	0.010895	1.9628	0.28818	8294	8294_at	HGNC:4793
190	CD40LG	CD40 ligand	2.7279	0.011069	1.9559	0.29014	959	959_at	HGNC:11935
191	CARD6	caspase recruitment domain family member 6	-2.7272	0.011086	1.9552	0.29014	84674	84674_at	HGNC:16394
192	RASSF4	Ras association domain family member 4	-2.7239	0.011172	1.9519	0.29089	83937	83937_at	HGNC:20793
193	DIPK1A	divergent protein kinase domain 1A	2.7146	0.011421	1.9423	0.29582	388650	388650_at	HGNC:32213
194	SLC38A5	solute carrier family 38 member 5	2.7085	0.011587	1.936	0.29857	92745	92745_at	HGNC:18070
195	ALDH3B1	aldehyde dehydrogenase 3 family member B1	-2.7008	0.011799	1.9281	0.30248	221	221_at	HGNC:410
196	GPX1	glutathione peroxidase 1	-2.6956	0.011945	1.9228	0.30365	2876	2876_at	HGNC:4553
197	SEC11A	SEC11 homolog A, signal peptidase complex subunit	-2.6948	0.011966	1.922	0.30365	23478	23478_at	HGNC:17718
198	SLC44A1	solute carrier family 44 member 1	-2.6856	0.012229	1.9126	0.3045	23446	23446_at	HGNC:18798
199	IGHV3-13	immunoglobulin heavy variable 3-13	2.6818	0.012339	1.9087	0.3045	28449	28449_at	HGNC:5581
200	CPPED1	calcineurin like phosphoesterase domain containing 1	-2.6806	0.012372	1.9075	0.3045	55313	55313_at	HGNC:25632
201	JAML	junction adhesion molecule like	-2.6803	0.012383	1.9072	0.3045	120425	120425_at	HGNC:19084
202	FRG1	FSHD region gene 1	-2.6791	0.012417	1.906	0.3045	2483	2483_at	HGNC:3954
203	H4C8	H4 clustered histone 8	2.6755	0.012522	1.9023	0.3045	8365	8365_at	HGNC:4788
204	MAML3	mastermind like transcriptional coactivator 3	-2.6755	0.012522	1.9023	0.3045	55534	55534_at	HGNC:16272
205	UAP1	UDP-N-acetylglucosamine pyrophosphorylase 1	2.6753	0.012528	1.9021	0.3045	6675	6675_at	HGNC:12457
206	NLN	neurolysin	-2.6739	0.012571	1.9006	0.3045	57486	57486_at	HGNC:16058
207	TIGAR	TP53 induced glycolysis regulatory phosphatase	-2.6712	0.012649	1.8979	0.3045	57103	57103_at	HGNC:1185
208	VEGFA	vascular endothelial growth factor A	-2.6705	0.01267	1.8972	0.3045	7422	7422_at	HGNC:12680
209	CD99L2	CD99 molecule like 2	-2.663	0.012894	1.8896	0.30716	83692	83692_at	HGNC:18237
210	ACER3	alkaline ceramidase 3	-2.6623	0.012915	1.8889	0.30716	55331	55331_at	HGNC:16066
211	CST3	cystatin C	-2.6543	0.013161	1.8807	0.30716	1471	1471_at	HGNC:2475
212	SPTAN1	spectrin alpha, non-erythrocytic 1	2.653	0.013199	1.8794	0.30716	6709	6709_at	HGNC:11273

213	RAB31	RAB31, member RAS oncogene family	-2.6529	0.013202	1.8793	0.30716	11031	11031_at	HGNC:9771
214	SGPL1	sphingosine-1-phosphate lyase 1	-2.6527	0.013208	1.8792	0.30716	8879	8879_at	HGNC:10817
215	H1-5	H1.5 linker histone, cluster member	2.6527	0.013211	1.8791	0.30716	3009	3009_at	HGNC:4719
216	NEK3	NIMA related kinase 3	-2.6499	0.013296	1.8763	0.30772	4752	4752_at	HGNC:7746
217	RNF135	ring finger protein 135	-2.6379	0.013673	1.8641	0.31354	84282	84282_at	HGNC:21158
218	TLR8	toll like receptor 8	-2.6334	0.013819	1.8595	0.31354	51311	51311_at	HGNC:15632
219	ALG10	ALG10 alpha-1,2-glucosyltransferase	-2.6307	0.013906	1.8568	0.31354	84920	84920_at	HGNC:23162
220	CYSLTR2	cysteinyl leukotriene receptor 2	-2.6297	0.013939	1.8558	0.31354	57105	57105_at	HGNC:18274
221	TCN2	transcobalamin 2	-2.6276	0.014006	1.8537	0.31354	6948	6948_at	HGNC:11653
222	ITSN1	intersectin 1	-2.6257	0.014069	1.8517	0.31354	6453	6453_at	HGNC:6183
223	C20orf194	chromosome 20 open reading frame 194	-2.6254	0.014077	1.8515	0.31354	25943	25943_at	HGNC:17721
224	ACTA2	actin alpha 2, smooth muscle	2.6253	0.01408	1.8514	0.31354	59	59_at	HGNC:130
225	RASSF2	Ras association domain family member 2	-2.6244	0.014112	1.8504	0.31354	9770	9770_at	HGNC:9883
226	USP18	ubiquitin specific peptidase 18	2.6171	0.014351	1.8431	0.31606	11274	11274_at	HGNC:12616
227	MZB1	marginal zone B and B1 cell specific protein	2.6171	0.014352	1.8431	0.31606	51237	51237_at	HGNC:30125
228	UBE2T	ubiquitin conjugating enzyme E2 T	2.6122	0.014515	1.8382	0.31751	29089	29089_at	HGNC:25009
229	SUSD4	sushi domain containing 4	-2.6114	0.014545	1.8373	0.31751	55061	55061_at	HGNC:25470
230	CD1C	CD1c molecule	-2.6064	0.014715	1.8323	0.31936	911	911_at	HGNC:1636
231	CASP1	caspase 1	-2.6051	0.014757	1.831	0.31936	834	834_at	HGNC:1499
232	CALCRL	calcitonin receptor like receptor	-2.6	0.014934	1.8258	0.32079	10203	10203_at	HGNC:16709
233	ENG	endoglin	-2.5968	0.015045	1.8226	0.32079	2022	2022_at	HGNC:3349
234	SNORA80E	small nucleolar RNA, H/ACA box 80E	2.5965	0.015055	1.8223	0.32079	677823	677823_at	HGNC:32635
235	KCTD12	potassium channel tetramerization domain containing 12	-2.594	0.015144	1.8198	0.32079	115207	115207_at	HGNC:14678
236	FOLR3	folate receptor gamma	-2.5934	0.015165	1.8192	0.32079	2352	2352_at	HGNC:3795
237	PADI2	peptidyl arginine deiminase 2	-2.5915	0.015229	1.8173	0.32079	11240	11240_at	HGNC:18341
238	GM2A	GM2 ganglioside activator	-2.5903	0.015273	1.8161	0.32079	2760	2760_at	HGNC:4367
239	HK2	hexokinase 2	-2.5863	0.015416	1.812	0.3218	3099	3099_at	HGNC:4923
240	PLA2G7	phospholipase A2 group VII	-2.5853	0.015449	1.8111	0.3218	7941	7941_at	HGNC:9040

241	MORN2	MORN repeat containing 2	-2.5784	0.015699	1.8041	0.32564	729967	729967_at	HGNC:30166
242	TPMT	thiopurine S-methyltransferase	-2.5762	0.015779	1.8019	0.32594	7172	7172_at	HGNC:12014
243	RPS27A	ribosomal protein S27a	-2.5733	0.015884	1.7991	0.32676	6233	6233_at	HGNC:10417
244	KSR1	kinase suppressor of ras 1	-2.57	0.016006	1.7957	0.32793	8844	8844_at	HGNC:6465
245	RAB34	RAB34, member RAS oncogene family	-2.5623	0.016292	1.788	0.33243	83871	83871_at	HGNC:16519
246	ARSD	arylsulfatase D	2.5561	0.016527	1.7818	0.33585	414	414_at	HGNC:717
247	G6PC3	glucose-6-phosphatase catalytic subunit 3	-2.5444	0.016978	1.7701	0.34278	92579	92579_at	HGNC:24861
248	HAAO	3-hydroxyanthranilate 3,4-dioxygenase	-2.5409	0.017114	1.7667	0.34278	23498	23498_at	HGNC:4796
249	VCAN	versican	-2.5406	0.017128	1.7663	0.34278	1462	1462_at	HGNC:2464
250	GATA6	GATA binding protein 6	-2.5402	0.017143	1.7659	0.34278	2627	2627_at	HGNC:4174
251	SNORD116-24	small nucleolar RNA, C/D box 116-24	2.5374	0.017253	1.7631	0.34362	100033435	100033435_at	HGNC:33090
252	SHCBP1	SHC binding and spindle associated 1	2.5235	0.017811	1.7493	0.35102	79801	79801_at	HGNC:29547
253	UBXN2B	UBX domain protein 2B	-2.5212	0.017907	1.747	0.35102	137886	137886_at	HGNC:27035
254	FRRS1	ferric chelate reductase 1	-2.5206	0.017929	1.7464	0.35102	391059	391059_at	HGNC:27622
255	PLK1	polo like kinase 1	2.5206	0.017932	1.7464	0.35102	5347	5347_at	HGNC:9077
256	UBE2A	ubiquitin conjugating enzyme E2 A	-2.5179	0.018043	1.7437	0.35102	7319	7319_at	HGNC:12472
257	JAK3	Janus kinase 3	2.5178	0.018046	1.7436	0.35102	3718	3718_at	HGNC:6193
258	CCL2	C-C motif chemokine ligand 2	2.5066	0.018513	1.7325	0.35377	6347	6347_at	HGNC:10618
259	SERTAD3	SERTA domain containing 3	-2.5051	0.018579	1.731	0.35377	29946	29946_at	HGNC:17931
260	SCARNA5	small Cajal body-specific RNA 5	2.5024	0.018691	1.7284	0.35377	677775	677775_at	HGNC:32561
261	NCEH1	neutral cholesterol ester hydrolase 1	-2.5011	0.018749	1.727	0.35377	57552	57552_at	HGNC:29260
262	DCBLD1	discoidin, CUB and LCCL domain containing 1	-2.4988	0.018845	1.7248	0.35377	285761	285761_at	HGNC:21479
263	SLC37A3	solute carrier family 37 member 3	2.4983	0.018865	1.7243	0.35377	84255	84255_at	HGNC:20651
264	HPGDS	hematopoietic prostaglandin D synthase	-2.4973	0.01891	1.7233	0.35377	27306	27306_at	HGNC:17890
265	SLC7A7	solute carrier family 7 member 7	-2.4972	0.018913	1.7232	0.35377	9056	9056_at	HGNC:11065
266	NPC2	NPC intracellular cholesterol transporter 2	-2.4962	0.018956	1.7222	0.35377	10577	10577_at	HGNC:14537

267	FMO5	flavin containing dimethylaniline monoxygenase 5	-2.4913	0.019171	1.7174	0.35377	2330	2330_at	HGNC:3773
268	NRG1	neuregulin 1	-2.4907	0.019198	1.7168	0.35377	3084	3084_at	HGNC:7997
269	IL18	interleukin 18	-2.4888	0.01928	1.7149	0.35377	3606	3606_at	HGNC:5986
270	GZMA	granzyme A	2.4884	0.019297	1.7145	0.35377	3001	3001_at	HGNC:4708
271	IGHV1OR15-9	immunoglobulin heavy variable 1/OR15-9 (non-functional)	2.4872	0.01935	1.7133	0.35377	390531	390531_at	HGNC:5569
272	MCTP1	multiple C2 and transmembrane domain containing 1	-2.4844	0.019473	1.7106	0.35377	79772	79772_at	HGNC:26183
273	PGD	phosphogluconate dehydrogenase	-2.4833	0.019524	1.7094	0.35377	5226	5226_at	HGNC:8891
274	LOC648987	uncharacterized LOC648987	-2.4824	0.01956	1.7086	0.35377	648987	648987_at	
275	SGMS2	sphingomyelin synthase 2	-2.4821	0.019576	1.7083	0.35377	166929	166929_at	HGNC:28395
276	LMAN1	lectin, mannose binding 1	2.4818	0.019588	1.708	0.35377	3998	3998_at	HGNC:6631
277	SPATS2	spermatogenesis associated serine rich 2	2.4815	0.019603	1.7077	0.35377	65244	65244_at	HGNC:18650
278	SOCS3	suppressor of cytokine signaling 3	2.4776	0.019777	1.7038	0.35441	9021	9021_at	HGNC:19391
279	VSIG4	V-set and immunoglobulin domain containing 4	-2.4775	0.01978	1.7038	0.35441	11326	11326_at	HGNC:17032
280	RBIS	ribosomal biogenesis factor	-2.473	0.019984	1.6993	0.35678	401466	401466_at	HGNC:32235
281	GOLIM4	golgi integral membrane protein 4	-2.4681	0.020206	1.6945	0.35946	27333	27333_at	HGNC:15448
282	SLC14A1	solute carrier family 14 member 1 (Kidd blood group)	2.4657	0.020317	1.6921	0.35998	6563	6563_at	HGNC:10918
283	PLBD1	phospholipase B domain containing 1	-2.4635	0.020422	1.6899	0.35998	79887	79887_at	HGNC:26215
284	NBPF1	NBPF member 1	-2.4622	0.020478	1.6887	0.35998	55672	55672_at	HGNC:26088
285	DPYD	dihydropyrimidine dehydrogenase	-2.4596	0.020601	1.6861	0.35998	1806	1806_at	HGNC:3012
286	RAB32	RAB32, member RAS oncogene family	-2.4578	0.020683	1.6844	0.35998	10981	10981_at	HGNC:9772
287	TNFSF13B	TNF superfamily member 13b	-2.4576	0.020693	1.6842	0.35998	10673	10673_at	HGNC:11929
288	MIR29A	microRNA 29a	-2.4566	0.020739	1.6832	0.35998	407021	407021_at	HGNC:31616
289	NIPSNAP3B	nipsnap homolog 3B	-2.4522	0.020948	1.6788	0.36138	55335	55335_at	HGNC:23641

290	EEF1DP3	eukaryotic translation elongation factor 1 delta pseudogene 3	-2.4503	0.021037	1.677	0.36138	196549	196549_at	HGNC:30486
291	SEC11C	SEC11 homolog C, signal peptidase complex subunit	2.4484	0.021132	1.6751	0.36138	90701	90701_at	HGNC:23400
292	PCID2	PCI domain containing 2	2.4478	0.021159	1.6745	0.36138	55795	55795_at	HGNC:25653
293	RRM2	ribonucleotide reductase regulatory subunit M2	2.4449	0.021297	1.6717	0.36138	6241	6241_at	HGNC:10452
294	ZBP1	Z-DNA binding protein 1	2.4386	0.021603	1.6655	0.36138	81030	81030_at	HGNC:16176
295	IFFO2	intermediate filament family orphan 2	2.4379	0.021638	1.6648	0.36138	126917	126917_at	HGNC:27006
296	SYCP2	synaptonemal complex protein 2	-2.4356	0.02175	1.6625	0.36138	10388	10388_at	HGNC:11490
297	PCLAF	PCNA clamp associated factor	2.4318	0.021936	1.6589	0.36138	9768	9768_at	HGNC:28961
298	NAGA	alpha-N-acetylgalactosaminidase	-2.4315	0.021951	1.6585	0.36138	4668	4668_at	HGNC:7631
299	CDKN3	cyclin dependent kinase inhibitor 3	2.431	0.021976	1.658	0.36138	1033	1033_at	HGNC:1791
300	CLCN5	chloride voltage-gated channel 5	-2.4304	0.022002	1.6575	0.36138	1184	1184_at	HGNC:2023
301	SNORD116-25	small nucleolar RNA, C/D box 116-25	2.4302	0.022015	1.6573	0.36138	100033436	100033436_at	HGNC:33091
302	SNAI3	snail family transcriptional repressor 3	-2.4301	0.022019	1.6572	0.36138	333929	333929_at	HGNC:18411
303	S1PR3	sphingosine-1-phosphate receptor 3	-2.428	0.022123	1.6552	0.36138	1903	1903_at	HGNC:3167
304	FPR3	formyl peptide receptor 3	-2.4275	0.022149	1.6547	0.36138	2359	2359_at	HGNC:3828
305	PTGDS	prostaglandin D2 synthase	-2.4269	0.022179	1.6541	0.36138	5730	5730_at	HGNC:9592
306	KIF11	kinesin family member 11	2.426	0.022226	1.6531	0.36138	3832	3832_at	HGNC:6388
307	ATF4	activating transcription factor 4	-2.4255	0.022247	1.6527	0.36138	468	468_at	HGNC:786
308	PCNA	proliferating cell nuclear antigen	2.4238	0.022332	1.6511	0.36138	5111	5111_at	HGNC:8729
309	ASB13	ankyrin repeat and SOCS box containing 13	-2.4237	0.022338	1.651	0.36138	79754	79754_at	HGNC:19765
310	C2CD2L	C2CD2 like	-2.42	0.022527	1.6473	0.36326	9854	9854_at	HGNC:29000
311	PIP5K1A	phosphatidylinositol-4-phosphate 5-kinase type 1 alpha	-2.4178	0.022635	1.6452	0.36384	8394	8394_at	HGNC:8994

312	CRIM1	cysteine rich transmembrane BMP regulator 1	2.416	0.022727	1.6435	0.36415	51232	51232_at	HGNC:2359
313	ARHGAP24	Rho GTPase activating protein 24	-2.4129	0.02289	1.6404	0.36558	83478	83478_at	HGNC:25361
314	C9orf64	chromosome 9 open reading frame 64	-2.4045	0.023324	1.6322	0.37133	84267	84267_at	HGNC:28144
315	SAMM50	SAMM50 sorting and assembly machinery component	-2.3998	0.023571	1.6276	0.37235	25813	25813_at	HGNC:24276
316	LMO2	LIM domain only 2	-2.3985	0.023638	1.6264	0.37235	4005	4005_at	HGNC:6642
317	PSAT1	phosphoserine aminotransferase 1	2.3961	0.023765	1.6241	0.37235	29968	29968_at	HGNC:19129
318	AP3M2	adaptor related protein complex 3 subunit mu 2	2.395	0.023823	1.623	0.37235	10947	10947_at	HGNC:570
319	TRGV3	T cell receptor gamma variable 3	-2.3944	0.023854	1.6224	0.37235	6976	6976_at	HGNC:12288
320	MYBL2	MYB proto-oncogene like 2	2.3918	0.023993	1.6199	0.37235	4605	4605_at	HGNC:7548
321	ZNF831	zinc finger protein 831	2.3906	0.024061	1.6187	0.37235	128611	128611_at	HGNC:16167
322	HPSE	heparanase	-2.39	0.024092	1.6181	0.37235	10855	10855_at	HGNC:5164
323	H3C8	H3 clustered histone 8	2.3894	0.024123	1.6176	0.37235	8355	8355_at	HGNC:4772
324	ERBB2	erb-b2 receptor tyrosine kinase 2	2.3874	0.024231	1.6156	0.37235	2064	2064_at	HGNC:3430
325	LAMP3	lysosomal associated membrane protein 3	2.3873	0.024236	1.6155	0.37235	27074	27074_at	HGNC:14582
326	CCNB2	cyclin B2	2.3865	0.024282	1.6147	0.37235	9133	9133_at	HGNC:1580
327	CORO1B	coronin 1B	-2.384	0.024418	1.6123	0.3733	57175	57175_at	HGNC:2253
328	GVINP1	GTPase, very large interferon inducible pseudogene 1	2.3809	0.024589	1.6093	0.37381	387751	387751_at	HGNC:25813
329			2.3806	0.024602	1.609	0.37381	100507025	100507025_at	
330	SNX30	sorting nexin family member 30	-2.3739	0.02497	1.6026	0.37618	401548	401548_at	HGNC:23685
331	GLA	galactosidase alpha	-2.3739	0.024975	1.6025	0.37618	2717	2717_at	HGNC:4296
332	TRGV9	T cell receptor gamma variable 9	2.3724	0.025058	1.6011	0.37618	6983	6983_at	HGNC:12295
333	SNORD116-23	small nucleolar RNA, C/D box 116-23	2.3724	0.025058	1.601	0.37618	100033434	100033434_at	HGNC:33089
334	C20orf27	chromosome 20 open reading frame 27	-2.3683	0.025287	1.5971	0.3784	54976	54976_at	HGNC:15873
335	JMJD8	jumonji domain containing 8	-2.3659	0.025422	1.5948	0.3784	339123	339123_at	HGNC:14148
336	CPNE8	copine 8	-2.3657	0.025433	1.5946	0.3784	144402	144402_at	HGNC:23498
337	FNIP2	folliculin interacting protein 2	-2.3618	0.025656	1.5908	0.38037	57600	57600_at	HGNC:29280

338	LOC642131	immunoglobulin IGHV1OR15-3-like pseudogene	2.3591	0.02581	1.5882	0.38037	642131	642131_at	
339	SLC25A40	solute carrier family 25 member 40	-2.3581	0.025866	1.5873	0.38037	55972	55972_at	HGNC:29680
340	LDOC1	LDOC1 regulator of NFKB signaling	-2.358	0.025871	1.5872	0.38037	23641	23641_at	HGNC:6548
341	WARS1	tryptophanyl-tRNA synthetase 1	-2.3549	0.026049	1.5842	0.38187	7453	7453_at	HGNC:12729
342	CALHM2	calcium homeostasis modulator family member 2	-2.3502	0.026324	1.5796	0.38202	51063	51063_at	HGNC:23493
343	CFP	complement factor properdin	-2.3491	0.026389	1.5786	0.38202	5199	5199_at	HGNC:8864
344	BICD2	BICD cargo adaptor 2	-2.3486	0.026416	1.5781	0.38202	23299	23299_at	HGNC:17208
345	GAS2L3	growth arrest specific 2 like 3	-2.3469	0.026518	1.5765	0.38202	283431	283431_at	HGNC:27475
346	BCORL1	BCL6 corepressor like 1	-2.3466	0.026536	1.5762	0.38202	63035	63035_at	HGNC:25657
347	CHPT1	choline phosphotransferase 1	-2.346	0.026573	1.5756	0.38202	56994	56994_at	HGNC:17852
348	LOC100132356	uncharacterized LOC100132356	-2.3456	0.026594	1.5752	0.38202	100132356	100132356_at	
349	BLVRB	biliverdin reductase B	-2.3412	0.026853	1.571	0.38463	645	645_at	HGNC:1063
350	GK3P	glycerol kinase 3 pseudogene	-2.3349	0.027233	1.5649	0.38897	2713	2713_at	HGNC:4292
351	MYC	MYC proto-oncogene, bHLH transcription factor	2.3321	0.027402	1.5622	0.38924	4609	4609_at	HGNC:7553
352	H3C2	H3 clustered histone 2	2.332	0.027408	1.5621	0.38924	8358	8358_at	HGNC:4776
353	SLC25A19	solute carrier family 25 member 19	-2.3283	0.027633	1.5586	0.39133	60386	60386_at	HGNC:14409
354	USP32P1	ubiquitin specific peptidase 32 pseudogene 1	-2.3266	0.027737	1.5569	0.39169	162632	162632_at	HGNC:43570
355	ANKRD18A	ankyrin repeat domain 18A	-2.3187	0.028221	1.5494	0.39739	253650	253650_at	HGNC:23643
356	EVI5	ecotropic viral integration site 5	-2.3165	0.02836	1.5473	0.39823	7813	7813_at	HGNC:3501
357	DUSP3	dual specificity phosphatase 3	-2.3119	0.028647	1.5429	0.40006	1845	1845_at	HGNC:3069
358	LIPA	lipase A, lysosomal acid type	-2.3119	0.02865	1.5429	0.40006	3988	3988_at	HGNC:6617
359	TRDV2	T cell receptor delta variable 2	2.31	0.028767	1.5411	0.4001	28517	28517_at	HGNC:12263
360	MIRLET7G	microRNA let-7g	-2.3093	0.028814	1.5404	0.4001	406890	406890_at	HGNC:31485
361	RPL13	ribosomal protein L13	2.308	0.028893	1.5392	0.4001	6137	6137_at	HGNC:10303
362	REXO2	RNA exonuclease 2	2.3057	0.029042	1.537	0.40105	25996	25996_at	HGNC:17851

363	STAB1	stabilin 1	-2.302	0.029281	1.5334	0.40294	23166	23166_at	HGNC:18628
364	SAMD3	sterile alpha motif domain containing 3	2.3002	0.029392	1.5318	0.40294	154075	154075_at	HGNC:21574
365	ZNF101	zinc finger protein 101	2.2998	0.029421	1.5313	0.40294	94039	94039_at	HGNC:12881
366	NCAPG	non-SMC condensin I complex subunit G	2.2952	0.029716	1.527	0.40318	64151	64151_at	HGNC:24304
367	RPS26	ribosomal protein S26	-2.2948	0.029744	1.5266	0.40318	6231	6231_at	HGNC:10414
368	LOC401261	uncharacterized LOC401261	-2.2947	0.029752	1.5265	0.40318	401261	401261_at	
369	HSD17B12	hydroxysteroid 17-beta dehydrogenase 12	-2.2939	0.029804	1.5257	0.40318	51144	51144_at	HGNC:18646
370	NMUR1	neuromedin U receptor 1	2.2933	0.029841	1.5252	0.40318	10316	10316_at	HGNC:4518
371	CXCL16	C-X-C motif chemokine ligand 16	-2.2883	0.030174	1.5204	0.40542	58191	58191_at	HGNC:16642
372	FDPSP2	farnesyl diphosphate synthase pseudogene 2	2.2873	0.030238	1.5195	0.40542	619190	619190_at	HGNC:3633
373	MANF	mesencephalic astrocyte derived neurotrophic factor	2.2859	0.030331	1.5181	0.40542	7873	7873_at	HGNC:15461
374	H3C3	H3 clustered histone 3	2.2839	0.030463	1.5162	0.40542	8352	8352_at	HGNC:4768
375	CTSZ	cathepsin Z	-2.2827	0.030546	1.515	0.40542	1522	1522_at	HGNC:2547
376	HDAC3	histone deacetylase 3	-2.2816	0.030621	1.514	0.40542	8841	8841_at	HGNC:4854
377	ADPGK	ADP dependent glucokinase	-2.2811	0.030653	1.5135	0.40542	83440	83440_at	HGNC:25250
378	NFAM1	NFAT activating protein with ITAM motif 1	-2.281	0.030656	1.5135	0.40542	150372	150372_at	HGNC:29872
379	SEMA3C	semaphorin 3C	-2.2794	0.030764	1.512	0.40578	10512	10512_at	HGNC:10725
380	RGL1	ral guanine nucleotide dissociation stimulator like 1	-2.2741	0.031126	1.5069	0.40706	23179	23179_at	HGNC:30281
381	F11R	F11 receptor	-2.274	0.031131	1.5068	0.40706	50848	50848_at	HGNC:14685
382	SLAMF7	SLAM family member 7	2.2732	0.031186	1.506	0.40706	57823	57823_at	HGNC:21394
383	TCTEX1D2	Tctex1 domain containing 2	2.2732	0.031187	1.506	0.40706	255758	255758_at	HGNC:28482
384	ZNF510	zinc finger protein 510	2.2705	0.031369	1.5035	0.40837	22869	22869_at	HGNC:29161
385	ARF3	ADP ribosylation factor 3	-2.2656	0.031704	1.4989	0.41166	377	377_at	HGNC:654
386	SLC36A1	solute carrier family 36 member 1	-2.2637	0.031838	1.4971	0.41232	206358	206358_at	HGNC:18761
387	CIDEB	cell death inducing DFFA like effector b	-2.2625	0.031923	1.4959	0.41236	27141	27141_at	HGNC:1977
388	LTA4H	leukotriene A4 hydrolase	2.2591	0.032156	1.4927	0.4143	4048	4048_at	HGNC:6710
389	MRPL35	mitochondrial ribosomal protein L35	2.2569	0.032313	1.4906	0.41525	51318	51318_at	HGNC:14489

390	DTL	denticleless E3 ubiquitin protein ligase homolog	2.2555	0.03241	1.4893	0.41542	51514	51514_at	HGNC:30288
391	TPCN1	two pore segment channel 1	-2.254	0.032516	1.4879	0.41573	53373	53373_at	HGNC:18182
392	TMEM272	transmembrane protein 272	2.2503	0.032783	1.4844	0.41707	283521	283521_at	HGNC:26737
393	ADTRP	androgen dependent TFPI regulating protein	2.2502	0.032788	1.4843	0.41707	84830	84830_at	HGNC:21214
394	GOLPH3L	golgi phosphoprotein 3 like	2.2461	0.033078	1.4805	0.41744	55204	55204_at	HGNC:24882
395	H3C12	H3 clustered histone 12	2.2447	0.033179	1.4791	0.41744	8356	8356_at	HGNC:4774
396	CAMSAP1	calmodulin regulated spectrin associated protein 1	-2.2438	0.033246	1.4783	0.41744	157922	157922_at	HGNC:19946
397	TALDO1	transaldolase 1	-2.243	0.033303	1.4775	0.41744	6888	6888_at	HGNC:11559
398	TK2	thymidine kinase 2	-2.2423	0.033354	1.4769	0.41744	7084	7084_at	HGNC:11831
399	FKBP11	FKBP prolyl isomerase 11	2.2401	0.033514	1.4748	0.41744	51303	51303_at	HGNC:18624
400	H2BC14	H2B clustered histone 14	2.2396	0.033551	1.4743	0.41744	8342	8342_at	HGNC:4750
401	CDC25B	cell division cycle 25B	2.2395	0.033556	1.4742	0.41744	994	994_at	HGNC:1726
402	CD84	CD84 molecule	2.2384	0.033641	1.4731	0.41744	8832	8832_at	HGNC:1704
403	SLC22A15	solute carrier family 22 member 15	-2.2382	0.033654	1.473	0.41744	55356	55356_at	HGNC:20301
404	RDX	radixin	-2.2338	0.033973	1.4689	0.41744	5962	5962_at	HGNC:9944
405	ZNF138	zinc finger protein 138	2.2326	0.034062	1.4677	0.41744	7697	7697_at	HGNC:12922
406	SNORD48	small nucleolar RNA, C/D box 48	2.2324	0.034076	1.4676	0.41744	26801	26801_at	HGNC:10188
407	SOX5	SRY-box transcription factor 5	2.2309	0.034191	1.4661	0.41744	6660	6660_at	HGNC:11201
408	SLFN12	schlafen family member 12	-2.2302	0.034239	1.4655	0.41744	55106	55106_at	HGNC:25500
409	H4C2	H4 clustered histone 2	2.2302	0.03424	1.4655	0.41744	8366	8366_at	HGNC:4789
410	PEMT	phosphatidylethanolamine N-methyltransferase	-2.2288	0.034349	1.4641	0.41744	10400	10400_at	HGNC:8830
411	GINS2	GINS complex subunit 2	2.2287	0.034352	1.464	0.41744	51659	51659_at	HGNC:24575
412	ATP11A	ATPase phospholipid transporting 11A	-2.228	0.034404	1.4634	0.41744	23250	23250_at	HGNC:13552
413	TRANK1	tetratricopeptide repeat and ankyrin repeat containing 1	2.224	0.034701	1.4597	0.41767	9881	9881_at	HGNC:29011
414	CD2	CD2 molecule	2.2238	0.034716	1.4595	0.41767	914	914_at	HGNC:1639
415	ARRDC4	arrestin domain containing 4	-2.2229	0.034784	1.4586	0.41767	91947	91947_at	HGNC:28087

416	IGHA1	immunoglobulin heavy constant alpha 1	2.2229	0.034785	1.4586	0.41767	3493	3493_at	HGNC:5478
417	RBMXL1	RBMX like 1	-2.222	0.034854	1.4577	0.41767	494115	494115_at	HGNC:25073
418	GPRIN3	GPRIN family member 3	2.2205	0.034965	1.4564	0.41767	285513	285513_at	HGNC:27733
419	DMXL2	Dmx like 2	-2.2197	0.035024	1.4556	0.41767	23312	23312_at	HGNC:2938
420	CD302	CD302 molecule	-2.2189	0.035092	1.4548	0.41767	9936	9936_at	HGNC:30843
421	HLA-DQB2	major histocompatibility complex, class II, DQ beta 2	-2.2152	0.03537	1.4514	0.41998	3120	3120_at	HGNC:4945
422	UBE2D1	ubiquitin conjugating enzyme E2 D1	-2.2139	0.035471	1.4501	0.4201	7321	7321_at	HGNC:12474
423	MSR1	macrophage scavenger receptor 1	-2.2117	0.035638	1.4481	0.4201	4481	4481_at	HGNC:7376
424	ADPRM	ADP-ribose/CDP-alcohol diphosphatase, manganese dependent	2.2104	0.035737	1.4469	0.4201	56985	56985_at	HGNC:30925
425	MYPOP	Myb related transcription factor, partner of profilin	-2.2099	0.035777	1.4464	0.4201	339344	339344_at	HGNC:20178
426	SLC43A2	solute carrier family 43 member 2	-2.2086	0.03588	1.4451	0.4201	124935	124935_at	HGNC:23087
427	CDK5	cyclin dependent kinase 5	-2.2085	0.035884	1.4451	0.4201	1020	1020_at	HGNC:1774
428	IGLV9-49	immunoglobulin lambda variable 9-49	2.2062	0.036062	1.4429	0.42121	28773	28773_at	HGNC:5933
429	CHP1	calcineurin like EF-hand protein 1	-2.2035	0.036269	1.4405	0.42166	11261	11261_at	HGNC:17433
430	TOP2A	DNA topoisomerase II alpha	2.2035	0.03627	1.4404	0.42166	7153	7153_at	HGNC:11989
431	MTFR1L	mitochondrial fission regulator 1 like	-2.202	0.036386	1.4391	0.42182	56181	56181_at	HGNC:28836
432	LYPLA1	lysophospholipase 1	-2.2002	0.036529	1.4374	0.42182	10434	10434_at	HGNC:6737
433	LTB	lymphotoxin beta	2.1991	0.036618	1.4363	0.42182	4050	4050_at	HGNC:6711
434	IGLV1-40	immunoglobulin lambda variable 1-40	2.1991	0.036621	1.4363	0.42182	28825	28825_at	HGNC:5877
435	DNTTIP1	deoxynucleotidyltransferase terminal interacting protein 1	-2.1943	0.036999	1.4318	0.42419	116092	116092_at	HGNC:16160
436	SNORD14C	small nucleolar RNA, C/D box 14C	2.1942	0.037003	1.4318	0.42419	85389	85389_at	HGNC:30352
437	SLC16A5	solute carrier family 16 member 5	-2.1923	0.037157	1.43	0.42419	9121	9121_at	HGNC:10926
438	SLC46A2	solute carrier family 46 member 2	-2.1922	0.037167	1.4298	0.42419	57864	57864_at	HGNC:16055
439	PDE4B	phosphodiesterase 4B	2.1905	0.037302	1.4283	0.42476	5142	5142_at	HGNC:8781
440	HDAC9	histone deacetylase 9	2.1879	0.037508	1.4259	0.42506	9734	9734_at	HGNC:14065

441	MIRLET7I	microRNA let-7i	-2.1877	0.037527	1.4257	0.42506	406891	406891_at	HGNC:31486
442	CCDC149	coiled-coil domain containing 149	-2.1863	0.037639	1.4244	0.42506	91050	91050_at	HGNC:25405
443	IRF5	interferon regulatory factor 5	-2.1859	0.037668	1.424	0.42506	3663	3663_at	HGNC:6120
444	SPNS1	sphingolipid transporter 1 (putative)	-2.1836	0.037853	1.4219	0.42619	83985	83985_at	HGNC:30621
445	XCR1	X-C motif chemokine receptor 1	-2.1819	0.037996	1.4203	0.42678	2829	2829_at	HGNC:1625
446	ARHGAP42	Rho GTPase activating protein 42	2.1809	0.038077	1.4193	0.42678	143872	143872_at	HGNC:26545
447	RNF157	ring finger protein 157	2.1777	0.038339	1.4164	0.42877	114804	114804_at	HGNC:29402
448	KCNA3	potassium voltage-gated channel subfamily A member 3	2.174	0.03864	1.413	0.42996	3738	3738_at	HGNC:6221
449			2.1737	0.038665	1.4127	0.42996	221016	221016_at	
450	FBXO36	F-box protein 36	-2.172	0.038808	1.4111	0.42996	130888	130888_at	HGNC:27020
451	ARL14EP	ADP ribosylation factor like GTPase 14 effector protein	2.171	0.038887	1.4102	0.42996	120534	120534_at	HGNC:26798
452	CD81	CD81 molecule	2.1673	0.039203	1.4067	0.42996	975	975_at	HGNC:1701
453	CADM1	cell adhesion molecule 1	-2.1664	0.039276	1.4059	0.42996	23705	23705_at	HGNC:5951
454	SNORD116-13	small nucleolar RNA, C/D box 116-13	2.1663	0.039282	1.4058	0.42996	100033425	100033425_at	HGNC:33079
455	ANO5	anoctamin 5	-2.1657	0.039336	1.4052	0.42996	203859	203859_at	HGNC:27337
456	TTYH3	tweety family member 3	-2.1653	0.039368	1.4049	0.42996	80727	80727_at	HGNC:22222
457			2.1651	0.039383	1.4047	0.42996	100652927	100652927_at	
458	SNORD49B	small nucleolar RNA, C/D box 49B	2.1643	0.039448	1.404	0.42996	692087	692087_at	HGNC:32721
459	TRBV5-1	T cell receptor beta variable 5-1	2.1639	0.039484	1.4036	0.42996	28614	28614_at	HGNC:12218
460	LTBR	lymphotoxin beta receptor	-2.1627	0.039583	1.4025	0.42996	4055	4055_at	HGNC:6718
461	ATP8B2	ATPase phospholipid transporting 8B2	2.1596	0.039848	1.3996	0.42996	57198	57198_at	HGNC:13534
462	PRKCQ	protein kinase C theta	2.1594	0.039866	1.3994	0.42996	5588	5588_at	HGNC:9410
463	STAT4	signal transducer and activator of transcription 4	2.1593	0.039878	1.3993	0.42996	6775	6775_at	HGNC:11365
464	CRISPLD2	cysteine rich secretory protein LCCL domain containing 2	-2.1581	0.039974	1.3982	0.42996	83716	83716_at	HGNC:25248
465	RXRA	retinoid X receptor alpha	-2.1548	0.04026	1.3951	0.42996	6256	6256_at	HGNC:10477

466	SIAH1	siah E3 ubiquitin protein ligase 1	-2.1546	0.04028	1.3949	0.42996	6477	6477_at	HGNC:10857
467	RCBTB2	RCC1 and BTB domain containing protein 2	-2.1545	0.040285	1.3949	0.42996	1102	1102_at	HGNC:1914
468	RIC1	RIC1 homolog, RAB6A GEF complex partner 1	-2.1541	0.040317	1.3945	0.42996	57589	57589_at	HGNC:17686
469	SNORD116-1	small nucleolar RNA, C/D box 116-1	2.1531	0.040403	1.3936	0.42996	100033413	100033413_at	HGNC:33067
470	ANXA2	annexin A2	-2.1519	0.040506	1.3925	0.42996	302	302_at	HGNC:537
471	TMOD2	tropomodulin 2	-2.1519	0.04051	1.3924	0.42996	29767	29767_at	HGNC:11872
472	GCSAML	germinal center associated signaling and motility like	-2.1482	0.040825	1.3891	0.43187	148823	148823_at	HGNC:29583
473	EMILIN2	elastin microfibril interfacer 2	-2.1472	0.040915	1.3881	0.43187	84034	84034_at	HGNC:19881
474	HERC6	HECT and RLD domain containing E3 ubiquitin protein ligase family member 6	2.1466	0.040969	1.3875	0.43187	55008	55008_at	HGNC:26072
475	BAIAP2	BAR/IMD domain containing adaptor protein 2	-2.1455	0.041062	1.3866	0.43187	10458	10458_at	HGNC:947
476	FBXO5	F-box protein 5	2.1429	0.041286	1.3842	0.43187	26271	26271_at	HGNC:13584
477	ZNF385A	zinc finger protein 385A	-2.1398	0.041567	1.3813	0.43187	25946	25946_at	HGNC:17521
478	LXN	latexin	2.1394	0.041599	1.3809	0.43187	56925	56925_at	HGNC:13347
479	PNPLA1	patatin like phospholipase domain containing 1	-2.1394	0.041602	1.3809	0.43187	285848	285848_at	HGNC:21246
480	ZNF486	zinc finger protein 486	2.139	0.041633	1.3806	0.43187	90649	90649_at	HGNC:20807
481	DUSP16	dual specificity phosphatase 16	2.1385	0.041679	1.3801	0.43187	80824	80824_at	HGNC:17909
482	GCC2	GRIP and coiled-coil domain containing 2	2.1374	0.041778	1.3791	0.43187	9648	9648_at	HGNC:23218
483	TRAV17	T cell receptor alpha variable 17	2.1373	0.041784	1.379	0.43187	28666	28666_at	HGNC:12113
484	RNF217	ring finger protein 217	-2.137	0.041813	1.3787	0.43187	154214	154214_at	HGNC:21487
485	MCM8	minichromosome maintenance 8 homologous recombination repair factor	2.1357	0.041926	1.3775	0.43214	84515	84515_at	HGNC:16147
486	NMI	N-myc and STAT interactor	2.1299	0.042443	1.3722	0.43579	9111	9111_at	HGNC:7854
487	CORO2A	coronin 2A	-2.1298	0.042455	1.3721	0.43579	7464	7464_at	HGNC:2255
488	IL21R	interleukin 21 receptor	2.1263	0.042767	1.3689	0.43618	50615	50615_at	HGNC:6006
489	PBX3	PBX homeobox 3	-2.1247	0.042911	1.3674	0.43618	5090	5090_at	HGNC:8634

490	FEZ2	fasciculation and elongation protein zeta 2	-2.1236	0.043016	1.3664	0.43618	9637	9637_at	HGNC:3660
491	FAM118B	family with sequence similarity 118 member B	-2.1234	0.043036	1.3662	0.43618	79607	79607_at	HGNC:26110
492	TRAPPC3	trafficking protein particle complex 3	-2.1232	0.043048	1.366	0.43618	27095	27095_at	HGNC:19942
493	CRIP2	cysteine rich protein 2	-2.1226	0.043103	1.3655	0.43618	1397	1397_at	HGNC:2361
494	ACRBP	acrosin binding protein	-2.1226	0.043104	1.3655	0.43618	84519	84519_at	HGNC:17195
495	IFITM1	interferon induced transmembrane protein 1	2.1202	0.043326	1.3633	0.43755	8519	8519_at	HGNC:5412
496	NHS	NHS actin remodeling regulator	-2.1167	0.043644	1.3601	0.43987	4810	4810_at	HGNC:7820
497	MEFV	MEFV innate immuity regulator, pyrin	-2.1147	0.043826	1.3583	0.44012	4210	4210_at	HGNC:6998
498	H3C7	H3 clustered histone 7	2.1137	0.043926	1.3573	0.44012	8968	8968_at	HGNC:4773
499	LONRF3	LON peptidase N-terminal domain and ring finger 3	-2.1136	0.043933	1.3572	0.44012	79836	79836_at	HGNC:21152
500	FCRL6	Fc receptor like 6	2.1117	0.044105	1.3555	0.44096	343413	343413_at	HGNC:31910
501	SLAMF1	signaling lymphocytic activation molecule family member 1	2.1108	0.044194	1.3546	0.44097	6504	6504_at	HGNC:10903
502	FUCA2	alpha-L-fucosidase 2	-2.1091	0.044355	1.3531	0.44163	2519	2519_at	HGNC:4008
503	TSPAN5	tetraspanin 5	2.1073	0.044521	1.3514	0.44163	10098	10098_at	HGNC:17753
504	APOBEC3G	apolipoprotein B mRNA editing enzyme catalytic subunit 3G	2.1063	0.044612	1.3505	0.44163	60489	60489_at	HGNC:17357
505	STX11	syntaxin 11	-2.1063	0.044613	1.3505	0.44163	8676	8676_at	HGNC:11429
506	CMTM3	CKLF like MARVEL transmembrane domain containing 3	-2.105	0.044732	1.3494	0.44193	123920	123920_at	HGNC:19174
507	ECRP	ribonuclease A family member 2 pseudogene	-2.102	0.045022	1.3466	0.44311	643332	643332_at	
508	S1PR1	sphingosine-1-phosphate receptor 1	2.1019	0.045029	1.3465	0.44311	1901	1901_at	HGNC:3165
509	TNFRSF17	TNF receptor superfamily member 17	2.1009	0.045127	1.3456	0.4432	608	608_at	HGNC:11913
510	TENT5C	terminal nucleotidyltransferase 5C	2.0981	0.045392	1.343	0.44484	54855	54855_at	HGNC:24712
511	SNORA1	small nucleolar RNA, H/ACA box 1	2.0969	0.045501	1.342	0.44484	677792	677792_at	HGNC:32557
512	CSF2RA	colony stimulating factor 2 receptor alpha subunit	-2.0963	0.04556	1.3414	0.44484	1438	1438_at	HGNC:2435

513	CCNE2	cyclin E2	2.0931	0.045872	1.3385	0.447	9134	9134_at	HGNC:1590
514	ZC2HC1A	zinc finger C2HC-type containing 1A	-2.0919	0.045986	1.3374	0.447	51101	51101_at	HGNC:24277
515	MAL	mal, T cell differentiation protein	2.0912	0.04605	1.3368	0.447	4118	4118_at	HGNC:6817
516	IGHG1	immunoglobulin heavy constant gamma 1 (G1m marker)	2.0842	0.046737	1.3303	0.45234	3500	3500_at	HGNC:5525
517	PATL2	PAT1 homolog 2	2.0837	0.046781	1.3299	0.45234	197135	197135_at	HGNC:33630
518	ASGR2	asialoglycoprotein receptor 2	-2.082	0.046947	1.3284	0.45307	433	433_at	HGNC:743
519	TMA16	translation machinery associated 16 homolog	-2.0785	0.047297	1.3252	0.45469	55319	55319_at	HGNC:25638
520	PILRA	paired immunoglobin like type 2 receptor alpha	-2.0779	0.047351	1.3247	0.45469	29992	29992_at	HGNC:20396
521	ZFHX3	zinc finger homeobox 3	-2.0776	0.047388	1.3243	0.45469	463	463_at	HGNC:777
522	LCK	LCK proto-oncogene, Src family tyrosine kinase	2.0747	0.047677	1.3217	0.45649	3932	3932_at	HGNC:6524
523	PLP2	proteolipid protein 2	-2.0734	0.047798	1.3206	0.45649	5355	5355_at	HGNC:9087
524	SH3BGRL	SH3 domain binding glutamate rich protein like	-2.0721	0.047934	1.3194	0.45649	6451	6451_at	HGNC:10823
525	FLVCR2	FLVCR heme transporter 2	-2.0707	0.04807	1.3181	0.45649	55640	55640_at	HGNC:20105
526	SLC45A3	solute carrier family 45 member 3	-2.0705	0.048094	1.3179	0.45649	85414	85414_at	HGNC:8642
527	TPX2	TPX2 microtubule nucleation factor	2.0699	0.048149	1.3174	0.45649	22974	22974_at	HGNC:1249
528	TMEM156	transmembrane protein 156	2.0693	0.048215	1.3168	0.45649	80008	80008_at	HGNC:26260
529	MPP7	membrane palmitoylated protein 7	-2.0659	0.048557	1.3137	0.4587	143098	143098_at	HGNC:26542
530	TOMM34	translocase of outer mitochondrial membrane 34	-2.0651	0.048632	1.3131	0.4587	10953	10953_at	HGNC:15746
531	SNORA62	small nucleolar RNA, H/ACA box 62	2.0582	0.049342	1.3068	0.46379	6044	6044_at	HGNC:10107
532	CEP78	centrosomal protein 78	2.058	0.049358	1.3066	0.46379	84131	84131_at	HGNC:25740
533	ARL8A	ADP ribosylation factor like GTPase 8A	-2.0565	0.049515	1.3053	0.4644	127829	127829_at	HGNC:25192
534	OSBPL1A	oxysterol binding protein like 1A	-2.054	0.049772	1.303	0.46586	114876	114876_at	HGNC:16398
535	CEP170	centrosomal protein 170	-2.0532	0.049857	1.3023	0.46586	9859	9859_at	HGNC:28920
	Data2								

S.No.	Symbol	ID	t.stat	p.value	X.log10.p.	FDR	GB_ACC	LOCUSLINK_ID
1	XLOC_008015	A_19_P00327297	5.568	7.94E-07	6.1004	0.0036082		NA
2	XIST	A_19_P00323692	5.2363	2.65E-06	5.5761	0.0036082	NR_001564	NA
3	RGS1	A_23_P97141	-5.2161	2.85E-06	5.5445	0.0036082	NM_002922	5996
4	AREG	A_23_P259071	-5.1631	3.45E-06	5.4616	0.0036082	NM_001657	374
5	USP9Y	A_33_P3217700	-5.1202	4.03E-06	5.3946	0.0036082	NM_004654	8287
6	XIST	A_19_P00331623	5.0487	5.21E-06	5.2836	0.0036082	NR_001564	NA
7	XIST	A_19_P00319151	5.0131	5.91E-06	5.2283	0.0036082	NR_001564	NA
8	RPS4Y1	A_23_P259314	-4.9731	6.81E-06	5.1665	0.0036082	NM_001008	6192
9	XIST	A_21_P0006538	4.9464	7.49E-06	5.1253	0.0036082	NR_001564	NA
10	NCRNA00185	A_23_P73848	-4.9306	7.93E-06	5.1009	0.0036082	NR_001544	55410
11	RPS4Y2	A_23_P324384	-4.9043	8.70E-06	5.0605	0.0036082	NM_001039567	140032
12	TTTY15	A_24_P348861	-4.8858	9.29E-06	5.0321	0.0036082	NR_001545	64595
13	XIST	A_19_P00329511	4.8829	9.38E-06	5.0276	0.0036082	NR_001564	NA
14	DDX3Y	A_33_P3224331	-4.8095	1.22E-05	4.9152	0.0040214	NM_001122665	8653
15	XLOC_008323	A_21_P0006606	-4.773	1.38E-05	4.8596	0.0040214		NA
16	LOC100509121	A_21_P0013996	-4.7713	1.39E-05	4.8569	0.0040214	XM_003120334	NA
17	XIST	A_33_P3341686	4.7621	1.44E-05	4.8429	0.0040214	NR_001564	7503
18	KDM5D	A_23_P137238	-4.7477	1.51E-05	4.821	0.0040214	NM_004653	8284
19	XIST	A_19_P00802872	4.7442	1.53E-05	4.8158	0.0040214	NR_001564	NA
20	TXLNG2P	A_23_P364792	-4.7119	1.71E-05	4.7666	0.0042641	NR_045129	246126
21	EIF1AY	A_24_P237511	-4.6872	1.87E-05	4.7293	0.0042641	NM_004681	9086
22	UTY	A_23_P329835	-4.6855	1.88E-05	4.7266	0.0042641	NM_007125	7404
23	TXLNG2P	A_33_P3260223	-4.6577	2.07E-05	4.6846	0.0043229	NR_045128	246126
24	P2RY2	A_23_P24903	-4.6566	2.08E-05	4.6829	0.0043229	NM_176072	5029
25	CXCR4	A_23_P102000	-4.6397	2.20E-05	4.6574	0.0044012	NM_001008540	7852
26	PRKY	A_23_P137248	-4.5824	2.68E-05	4.5712	0.0051609	NR_028062	5616
27	EIF1AY	A_23_P148629	-4.5437	3.07E-05	4.5131	0.0056809	NM_004681	9086
28	LOC100506003	A_21_P0014436	-4.4724	3.92E-05	4.4068	0.0069979	XR_109785	NA
29	EGR3	A_23_P216225	-4.3969	5.07E-05	4.2948	0.0087436	NM_004430	1960
30	AREG	A_33_P3419190	-4.3269	6.43E-05	4.1916	0.010719	NM_001657	374
31	ZFY	A_24_P942743	-4.3165	6.66E-05	4.1763	0.010745	NM_003411	7544
32	XLOC_l2_015892	A_21_P0013879	-4.2994	7.06E-05	4.1513	0.011027		NA
33	CACNG6	A_23_P501933	-4.2787	7.57E-05	4.121	0.011465	NM_145814	59285
34	DUSP2	A_24_P37409	-4.248	8.39E-05	4.0763	0.012334	NM_004418	1844
35	XLOC_008276	A_21_P0006590	-4.2391	8.64E-05	4.0633	0.012346		NA
36	TXLNG2P	A_23_P96658	-4.2193	9.24E-05	4.0345	0.012826	NR_045128	246126
37	FGFR2	A_23_P202334	-4.1701	0.00010885	3.9632	0.014707	NM_022970	2263
38	UTY	A_33_P3228977	-4.1463	0.00011783	3.9288	0.0155	BC012581	7404
39	SPNS3	A_23_P100963	-4.126	0.000126	3.8996	0.015873	NM_182538	201305
40	DDX3Y	A_24_P130936	-4.1237	0.00012701	3.8962	0.015873	NM_004660	8653
41	CCL23	A_24_P133905	-4.0843	0.00014465	3.8397	0.017636	NM_005064	6368
42	TTTY15	A_21_P0006594	-4.0492	0.00016237	3.7895	0.019259	NR_001545	NA
43	XLOC_008276	A_21_P0006589	-4.0387	0.0001681	3.7744	0.019259		NA
44	XLOC_011047	A_21_P0008446	-4.0299	0.00017299	3.762	0.019259		NA
45	PRSS41	A_33_P3416634	-4.0239	0.00017645	3.7534	0.019259	NM_001135086	360226

46	XLOC_008185	A_21_P0006456	4.0225	0.00017722	3.7515	0.019259		NA
47	BACE2	A_23_P154875	-3.987	0.00019907	3.701	0.021173	NM_012105	25825
48	PMAIP1	A_33_P3619171	-3.9508	0.00022394	3.6499	0.023322	NM_021127	5366
49	PER1	A_23_P89589	-3.9417	0.0002307	3.6369	0.023536	NM_002616	5187
50	CLDN5	A_33_P3285540	3.8881	0.00027437	3.5617	0.027431	NM_001130861	7122
51	SLC29A1	A_23_P133694	-3.8553	0.00030495	3.5158	0.029891	NM_001078177	2030
52	PRSS33	A_24_P327084	-3.8393	0.00032107	3.4934	0.030331	NM_152891	260429
53	FOSB	A_23_P429998	-3.8388	0.00032157	3.4927	0.030331	NM_006732	2354
54	DDIT4	A_23_P104318	-3.816	0.00034584	3.4611	0.032016	NM_019058	54541
55		A_33_P3387016	3.8009	0.000363	3.4401	0.032994		NA
56	CCL4	A_23_P207564	-3.7823	0.00038512	3.4144	0.033909	NM_002984	6351
57	CCL4	A_33_P3354607	-3.7748	0.00039449	3.404	0.033909	NM_002984	6351
58	XLOC_005505	A_21_P0004605	-3.7712	0.00039902	3.399	0.033909	CR607309	NA
59	CD69	A_23_P87879	-3.7702	0.00040021	3.3977	0.033909	NM_001781	969
60	XLOC_008276	A_21_P0006588	-3.7114	0.00048216	3.3168	0.03959		NA
61	GPR34	A_23_P11201	-3.7108	0.0004831	3.316	0.03959	NM_001097579	2857
62	IL5RA	A_33_P3328254	-3.6832	0.000527	3.2782	0.042227	NM_175725	3568
63	ALOX15	A_23_P55373	-3.6738	0.00054269	3.2655	0.042227	NM_001140	246
64	SMPD3	A_23_P163567	-3.671	0.00054757	3.2616	0.042227	NM_018667	55512
65	RAB44	A_33_P3424328	-3.6664	0.00055548	3.2553	0.042227	XM_003403441	401258
66	XLOC_012642	A_21_P0009466	-3.6643	0.0005592	3.2524	0.042227	DB516369	NA
67	NR4A2	A_33_P3299066	-3.6604	0.00056595	3.2472	0.042227	NM_006186	4929
68	CLC	A_23_P101683	-3.6439	0.00059609	3.2247	0.043821	NM_001828	1178
69	RAB44	A_21_P0013023	-3.5765	0.00073518	3.1336	0.053263	AK125083	NA
70	MGLL	A_33_P3281795	3.5697	0.00075084	3.1245	0.053621	NM_007283	11343
71	DISC1	A_21_P0000052	3.5613	0.00077058	3.1132	0.054255	NM_001164554	NA
72	PTGDR2	A_24_P115932	-3.5479	0.00080313	3.0952	0.055762	NM_004778	11251
73	SIGLEC8	A_24_P40001	-3.5272	0.00085583	3.0676	0.058607	NM_014442	27181
74	EPN2	A_23_P89310	-3.5079	0.00090826	3.0418	0.060734	NM_014964	22905
75	ANXA3	A_23_P121716	3.5068	0.00091119	3.0404	0.060734	NM_005139	306
76	CCR3	A_23_P250302	-3.4854	0.00097284	3.012	0.06399	NM_001837	1232
77	VSTM1	A_33_P3514487	-3.4595	0.0010527	2.9777	0.068343	NM_198481	284415
78	TSIX	A_33_P3405911	3.4274	0.0011608	2.9353	0.073044	NR_003255	9383
79	RAB44	A_33_P3349912	-3.4263	0.0011644	2.9339	0.073044	AK125083	401258
80	TMEM164	A_23_P96041	-3.425	0.0011689	2.9322	0.073044	NM_032227	84187
81	UTY	A_23_P160004	-3.4149	0.0012054	2.9189	0.073228	NM_182660	7404
82	LOC100652883	A_21_P0014014	-3.4141	0.0012082	2.9179	0.073228	XM_003403469	NA
83	LTC4S	A_24_P397294	-3.412	0.0012158	2.9151	0.073228	NM_145867	4056
84		A_33_P3343394	3.3874	0.0013098	2.8828	0.076784		NA
85	SAG	A_23_P5853	-3.3865	0.0013133	2.8816	0.076784	NM_000541	6295
86	SNAR-D	A_21_P0000508	3.3845	0.0013209	2.8791	0.076784	NR_024243	NA
87	CMTM5	A_23_P106042	3.3689	0.0013846	2.8587	0.079065	NM_001037288	116173
88	XLOC_l2_014694	A_21_P0013548	-3.3672	0.0013918	2.8564	0.079065		NA
89	CNR2	A_23_P310931	-3.3434	0.0014943	2.8256	0.083933	NM_001841	1269
90	PNMA6C	A_33_P3233436	-3.3349	0.0015328	2.8145	0.085139	NM_001170944	100287428
91		A_33_P3226546	-3.3267	0.0015707	2.8039	0.085408		NA
92	ITGA2B	A_24_P65373	3.3265	0.0015718	2.8036	0.085408	NM_000419	3674

93	F13A1	A_33_P3416097	3.3086	0.001658	2.7804	0.089123	NM_000129	2162
94	PMP22	A_23_P100711	-3.2996	0.0017028	2.7688	0.090394	NM_000304	5376
95	EMR4P	A_33_P3247320	-3.2966	0.0017178	2.765	0.090394	NR_024075	326342
96	TREML1	A_33_P3381777	3.2864	0.0017708	2.7518	0.091425	NM_178174	340205
97	TFF3	A_33_P3334305	-3.2858	0.001774	2.751	0.091425	NM_003226	7033
98	ALAS2	A_32_P385587	3.2732	0.0018414	2.7348	0.092074	NM_000032	212
99	ITGB3	A_24_P318656	3.2724	0.0018454	2.7339	0.092074	NM_000212	3690
100	IL3RA	A_32_P217750	-3.27	0.0018589	2.7307	0.092074	NM_002183	3563
101	THBS4	A_24_P260443	-3.2697	0.0018603	2.7304	0.092074	NM_003248	7060
102	SNAR-B2	A_21_P0000507	3.2587	0.0019216	2.7163	0.093372	NR_024230	NA
103	RNF208	A_33_P3244274	3.2583	0.0019239	2.7158	0.093372	BC016958	727800
104	ACACB	A_33_P3334220	-3.2479	0.0019839	2.7025	0.095362	NM_001093	32
105	PDE5A	A_33_P3378514	3.2378	0.0020436	2.6896	0.097293	NM_001083	8654
106	KCNJ15	A_33_P3376140	3.2264	0.0021134	2.675	0.099667	NM_170736	3772
107	MFSD6L	A_33_P3393331	-3.2172	0.0021708	2.6634	0.10142	NM_152599	162387
108	LOC389906	A_33_P3325229	3.2103	0.0022155	2.6545	0.10255	NR_034031	389906
109	TRIB1	A_24_P252497	-3.2071	0.0022361	2.6505	0.10255	NM_025195	10221
110	F13A1	A_32_P140139	3.2036	0.0022588	2.6461	0.10265	NM_000129	2162
111	EMR1	A_33_P3215803	-3.1919	0.0023373	2.6313	0.10446	NM_001974	2015
112	PPBP	A_23_P121596	3.1914	0.002341	2.6306	0.10446	NM_002704	5473
113	IL8	A_32_P87013	-3.1864	0.002375	2.6243	0.10446	NM_000584	3576
114	C6orf25	A_32_P168349	3.1854	0.0023821	2.623	0.10446	NM_138277	80739
115	EHD3	A_23_P79251	3.1754	0.0024526	2.6104	0.10661	NM_014600	30845
116	XLOC_008015	A_19_P00326248	3.1682	0.002504	2.6014	0.10791		NA
117		A_33_P3346333	-3.1512	0.0026307	2.5799	0.1124		NA
118	LEFTY1	A_23_P160336	3.1414	0.0027064	2.5676	0.11466	NM_020997	10637
119	GUCY1A3	A_23_P69573	3.1355	0.0027533	2.5601	0.11566	NM_000856	2982
120	CCL2	A_23_P89431	-3.1273	0.0028187	2.5499	0.11647	NM_002982	6347
121		A_33_P3349761	3.1273	0.0028191	2.5499	0.11647		NA
122		A_33_P3214516	-3.1062	0.0029952	2.5236	0.12273	M21784	NA
123	ZNRF3	A_24_P162373	-3.0933	0.0031086	2.5074	0.12634	NM_001206998	84133
124	NEFL	A_33_P3287785	3.0773	0.0032537	2.4876	0.13117	NM_006158	4747
125	NR4A3	A_23_P398566	-3.0723	0.0033003	2.4814	0.13199	NM_173200	8013
126	CD69	A_33_P3241021	-3.0648	0.0033717	2.4721	0.13377	AK303383	969
127	SCGB1C1	A_23_P27207	3.0555	0.0034627	2.4606	0.13596	NM_145651	147199
128	SLC16A14	A_24_P342829	-3.0536	0.0034812	2.4583	0.13596	NM_152527	151473
129	ABTB2	A_23_P356616	-3.0495	0.0035214	2.4533	0.13646	NM_145804	25841
130	SORD	A_33_P3413483	-3.0376	0.0036428	2.4386	0.13988	AK090738	6652
131	XLOC_l2_007424	A_21_P0011814	3.0349	0.0036712	2.4352	0.13988	CB178477	NA
132	ADORA3	A_23_P137931	-3.0327	0.0036936	2.4326	0.13988	NM_000677	140
133	SH2D2A	A_21_P0000028	-3.0293	0.003729	2.4284	0.14016	NM_001161444	NA
134	VWF	A_23_P105562	3.0132	0.0039032	2.4086	0.14561	NM_000552	7450
135	XLOC_008221	A_21_P0006461	-2.9927	0.0041346	2.3836	0.15257	BX331476	NA
136		A_33_P3304748	2.9873	0.004198	2.377	0.15257		NA
137		A_33_P3212704	2.9865	0.0042076	2.376	0.15257		NA
138	SERPINA1	A_33_P3289659	-2.9862	0.0042119	2.3755	0.15257	BX248257	5265
139	TSIX	A_19_P00316565	2.98	0.0042851	2.368	0.15307	NR_003255	NA

140	NR4A2	A_23_P131208	-2.9756	0.0043385	2.3627	0.15307	NM_006186	4929
141	CTDSPL	A_24_P251534	2.9755	0.0043397	2.3625	0.15307	NM_001008392	10217
142	RNF208	A_33_P3231670	2.9748	0.0043481	2.3617	0.15307	NM_031297	727800
143	CLU	A_23_P215913	2.9637	0.0044849	2.3482	0.15634	NM_001831	1191
144	CYP4F12	A_23_P108280	-2.9623	0.0045034	2.3465	0.15634	NM_023944	66002
145	C15orf26	A_23_P336612	2.9537	0.0046118	2.3361	0.159	NM_173528	161502
146	SIGLEC10	A_23_P208182	-2.9202	0.0050632	2.2956	0.17336	NM_033130	89790
147	LOC100506342	A_33_P3514859	-2.9009	0.0053402	2.2724	0.18024	XR_108862	100506342
148	SPTB	A_32_P134968	2.8985	0.0053757	2.2696	0.18024	NM_001024858	6710
149	SELP	A_23_P137697	2.8975	0.0053902	2.2684	0.18024	NM_003005	6403
150	IFIT1B	A_33_P3395532	2.8963	0.0054083	2.2669	0.18024	NM_001010987	439996
151	PDE2A	A_23_P401106	2.8841	0.0055932	2.2523	0.18517	NM_002599	5138
152		A_33_P3399755	2.8758	0.0057232	2.2424	0.18823		NA
153	ACOT11	A_23_P417415	-2.8689	0.0058319	2.2342	0.19055	NM_147161	26027
154	LOC645638	A_21_P0009342	-2.8615	0.0059519	2.2253	0.19224	NR_030732	NA
155	RAB2B	A_33_P3295803	2.861	0.0059607	2.2247	0.19224	NM_032846	84932
156	FSTL1	A_23_P212696	2.8527	0.0060966	2.2149	0.19537	NM_007085	11167
157	PRKAR2B	A_23_P42975	2.8501	0.0061411	2.2118	0.19554	NM_002736	5577
158	C3AR1	A_23_P2431	-2.8451	0.0062241	2.2059	0.19693	NM_004054	719
159	LINC00266-1	A_23_P355776	2.8412	0.0062913	2.2013	0.1978	NR_040415	140849
160	LOC728975	A_33_P3337019	2.8046	0.0069493	2.1581	0.21695	XR_110914	728975
161	PROS1	A_23_P73114	2.8017	0.0070048	2.1546	0.21695	NM_000313	5627
162	DDX43	A_23_P156445	-2.8003	0.0070306	2.153	0.21695	NM_018665	55510
163	P2RY14	A_24_P165864	-2.7936	0.0071601	2.1451	0.21939	NM_014879	9934
164	XLOC_013612	A_21_P0009991	2.7916	0.0071974	2.1428	0.21939		NA
165	LOC100506924	A_19_P00315587	2.7867	0.0072934	2.1371	0.22097	XR_110015	NA
166	LOC100507540	A_21_P0013589	-2.7777	0.0074737	2.1265	0.22507	XR_108989	NA
167	RHOBTB1	A_33_P3348288	2.7627	0.0077809	2.109	0.23091	NM_001242359	9886
168		A_33_P3422712	2.7572	0.0078965	2.1026	0.23091		NA
169	PAQR9	A_32_P196193	2.7565	0.0079106	2.1018	0.23091	NM_198504	344838
170	TNFRSF12A	A_23_P49338	-2.7557	0.0079268	2.1009	0.23091	NM_016639	51330
171	TMEM158	A_23_P369899	2.7552	0.0079387	2.1003	0.23091	NM_015444	25907
172		A_33_P3396326	2.7534	0.0079771	2.0982	0.23091		NA
173	FLJ39051	A_21_P0000607	2.7527	0.007991	2.0974	0.23091	NR_033839	NA
174	CEBPE	A_33_P3387991	-2.7473	0.0081071	2.0911	0.23218	NM_001805	1053
175	XLOC_009445	A_21_P0007354	2.7464	0.008128	2.09	0.23218	AK124475	NA
176	XLOC_l2_004866	A_21_P0011363	-2.7411	0.0082431	2.0839	0.23413	BC070492	NA
177	LOC100652951	A_21_P0014898	-2.7364	0.008348	2.0784	0.23577	XR_132888	NA
178	GP1BB	A_33_P3265030	2.7302	0.0084873	2.0712	0.23836	NM_000407	2812
179	ATP8B3	A_23_P79108	-2.7151	0.0088348	2.0538	0.24601	NM_138813	148229
180	CTTN	A_33_P3310780	2.7111	0.0089294	2.0492	0.24601	NM_005231	2017
181	XLOC_014105	A_19_P00813140	2.7089	0.0089825	2.0466	0.24601	BX648824	NA
182	ELOVL7	A_23_P359277	2.7087	0.0089854	2.0465	0.24601	NM_024930	79993
183	KREMEN1	A_23_P68851	2.7079	0.0090059	2.0455	0.24601	NM_001039570	83999
184	SOCS3	A_23_P207058	2.705	0.0090748	2.0422	0.24641	NM_003955	9021
185	XLOC_l2_001085	A_21_P0010646	-2.7018	0.0091511	2.0385	0.24641	AK090412	NA
186	GP6	A_33_P3461416	2.6994	0.0092098	2.0358	0.24641	NM_001083899	51206

187	CCT3	A_33_P3396831	2.6991	0.0092176	2.0354	0.24641		7203
188	WWC1	A_23_P81392	2.6948	0.0093226	2.0305	0.24789	NM_015238	23286
189	MRVI1-AS1	A_23_P396299	2.6853	0.0095591	2.0196	0.25284	NR_034094	100129827
190	LRRD1	A_33_P3420446	2.677	0.009771	2.0101	0.25708	NM_001161528	401387
191	XLOC_009790	A_21_P0007574	2.6687	0.009987	2.0006	0.26139		NA
192	LOC388948	A_19_P00321953	2.6632	0.010132	1.9943	0.26381	NR_034099	NA
193	NEURL	A_33_P3293524	2.6576	0.010284	1.9878	0.2652	NM_004210	9148
194	XLOC_007911	A_21_P0006410	2.6573	0.010292	1.9875	0.2652		NA
195	TMCC2	A_32_P133840	2.6426	0.010696	1.9708	0.27419	NM_014858	9911
196	SUV420H2	A_33_P3359473	-2.6257	0.011176	1.9517	0.28373	NM_032701	84787
197	AQP10	A_23_P126613	2.6255	0.011181	1.9515	0.28373	NM_080429	89872
198	XLOC_007052	A_19_P00316401	2.6136	0.011533	1.938	0.29119	BX500531	NA
199	XLOC_l2_000423	A_21_P0010727	2.6016	0.011897	1.9246	0.29886	AK311502	NA
200	TGFB1I1	A_33_P3236858	2.5982	0.012004	1.9207	0.30004	NM_001042454	7041
201	TSIX	A_19_P00321917	2.5926	0.012179	1.9144	0.3029	NR_003255	NA
202	PRSS36	A_24_P412734	2.5895	0.012275	1.911	0.30378	NM_173502	146547
203	XLOC_l2_008595	A_21_P0012113	2.5793	0.012605	1.8995	0.30762		NA
204	XLOC_009389	A_21_P0007325	2.579	0.012613	1.8992	0.30762		NA
205	XLOC_009548	A_21_P0007397	2.5781	0.012641	1.8982	0.30762		NA
206		A_33_P3407157	2.5771	0.012676	1.897	0.30762	DB050195	NA
207	XLOC_l2_012836	A_21_P0013052	2.5673	0.012998	1.8861	0.31338		NA
208	COL20A1	A_33_P3395952	2.5661	0.013039	1.8847	0.31338	NM_020882	57642
209	GPR87	A_23_P121120	2.5615	0.013194	1.8796	0.31559	NM_023915	53836
210	XLOC_000379	A_21_P0001011	2.5575	0.013329	1.8752	0.31728		NA
211	TNXB	A_23_P156708	2.5523	0.013509	1.8694	0.32006	NM_032470	7148
212	XLOC_013573	A_21_P0009860	2.5484	0.013646	1.865	0.32177	BC041864	NA
213	LY6G6F	A_33_P3214334	2.5456	0.013744	1.8619	0.32183	NM_001003693	259215
214	PDK2	A_21_P0000146	-2.5446	0.013777	1.8608	0.32183	NM_001199900	NA
215	LINC00442	A_21_P0008228	2.5362	0.014074	1.8516	0.32705	NR_026852	NA
216	C20orf108	A_33_P3306352	2.5343	0.014146	1.8494	0.32705		116151
217	UBE2O	A_24_P172993	2.5328	0.014197	1.8478	0.32705	NM_022066	63893
218	SELP	A_33_P3339100	2.5308	0.014272	1.8455	0.32727	NM_003005	6403
219	XLOC_007219	A_21_P0005631	2.5274	0.014395	1.8418	0.32858	BC014119	NA
220	GYPB	A_23_P212854	2.5234	0.014542	1.8374	0.32931	NM_002100	2994
221	UGT2B7	A_23_P136671	-2.5215	0.014614	1.8352	0.32931	NM_001074	7364
222	IGFBP2	A_23_P119943	2.5212	0.014624	1.8349	0.32931	NM_000597	3485
223	GP9	A_33_P3383226	2.5077	0.015132	1.8201	0.33861	NM_000174	2815
224	XLOC_008079	A_19_P00320275	2.5057	0.015211	1.8179	0.33861		NA
225	XLOC_l2_015441	A_21_P0013752	2.5038	0.015281	1.8158	0.33861		NA
226	XLOC_011940	A_21_P0009122	2.5032	0.015308	1.8151	0.33861		NA
227	CXCR6	A_23_P109913	-2.5009	0.015397	1.8126	0.33887	NM_006564	10663
228	LOC389906	A_32_P40377	2.4994	0.015456	1.8109	0.33887	NR_034031	389906
229	MYL9	A_23_P210425	2.4921	0.01574	1.803	0.34231	NM_181526	10398
230	SORD	A_23_P77103	-2.4919	0.015749	1.8027	0.34231	NM_003104	6652
231	ZNF880	A_24_P926783	-2.4877	0.015916	1.7982	0.34433	NM_001145434	400713
232	GNAZ	A_23_P416581	2.4829	0.016113	1.7928	0.34433	NM_002073	2781
233	USP9Y	A_33_P3725324	-2.4798	0.016237	1.7895	0.34433	NM_004654	8287

234	TTC25	A_23_P73150	2.4792	0.016261	1.7888	0.34433	NM_031421	83538
235	PRSS33	A_33_P3312779	-2.4791	0.016267	1.7887	0.34433	NM_152891	260429
236	IDO1	A_23_P112026	-2.479	0.016269	1.7886	0.34433	NM_002164	3620
237	XLOC_011807	A_21_P0008983	2.4777	0.016324	1.7872	0.34433		NA
238	HTR1B	A_23_P156824	2.4749	0.016438	1.7841	0.34528	NM_000863	3351
239	MMRN1	A_33_P3212257	2.4723	0.016544	1.7814	0.34605	NM_007351	22915
240	XLOC_001459	A_21_P0002210	2.4677	0.016736	1.7763	0.3486		NA
241	CD248	A_33_P3337485	-2.4616	0.016994	1.7697	0.35251	NM_020404	57124
242	SPRYD5	A_23_P150483	-2.457	0.017193	1.7646	0.35515	NM_032681	84767
243	S100A12	A_33_P3385785	2.4528	0.017374	1.7601	0.35742	NM_005621	6283
244	XLOC_014251	A_21_P0010362	2.4503	0.017484	1.7574	0.35821		NA
245	C19orf59	A_23_P330561	2.4437	0.017772	1.7503	0.36263	NM_174918	199675
246	S100A12	A_23_P74001	2.4367	0.018087	1.7426	0.36754	NM_005621	6283
247	PRKAR2B	A_33_P3304983	2.4331	0.018248	1.7388	0.36923	NM_002736	5577
248	DMRTC1	A_32_P179998	-2.4316	0.018317	1.7371	0.36923	NM_033053	63947
249	LINC00230A	A_33_P3308232	-2.4295	0.018413	1.7349	0.36966	NR_002161	401630
250	PVRL1	A_23_P76034	2.4212	0.018797	1.7259	0.3735	NM_203286	5818
251	ABCC3	A_23_P207507	2.4208	0.018814	1.7255	0.3735	NM_003786	8714
252	XLOC_l2_014423	A_21_P0013507	-2.4202	0.018844	1.7248	0.3735		NA
253	CXCL5	A_24_P277367	2.4189	0.018903	1.7235	0.3735	NM_002994	6374
254	CAT	A_23_P105138	-2.4158	0.019049	1.7201	0.37397	NM_001752	847
255	ITGAD	A_23_P129665	2.4148	0.019097	1.719	0.37397	NM_005353	3681
256	SNAR-G2	A_21_P0000509	2.4105	0.0193	1.7144	0.37397	NR_024244	NA
257	XLOC_000930	A_21_P0001158	2.4104	0.019303	1.7144	0.37397	AK056049	NA
258		A_32_P40375	2.4103	0.019311	1.7142	0.37397		NA
259	TNXB	A_33_P3381338	2.4083	0.019406	1.7121	0.37397	NM_019105	7148
260	LOC100129216	A_33_P3255531	2.4074	0.01945	1.7111	0.37397	NM_001242853	100129216
261	RAB27B	A_23_P107612	2.4009	0.019763	1.7042	0.37852	NM_004163	5874
262	XLOC_l2_006994	A_21_P0011776	2.398	0.019905	1.701	0.37948	BC068609	NA
263	ADM	A_23_P127948	2.3968	0.019964	1.6997	0.37948	NM_001124	133
264	EGR1	A_23_P214080	-2.395	0.020055	1.6978	0.37972	NM_001964	1958
265		A_33_P3233404	2.3935	0.020129	1.6962	0.37972	AB305916	NA
266	LINC00239	A_32_P176911	2.3898	0.02031	1.6923	0.38168	NR_026774	145200
267	ZBTB42	A_33_P3289128	-2.3837	0.02062	1.6857	0.38556	NM_001137601	100128927
268	FHL3	A_23_P114839	-2.3827	0.02067	1.6847	0.38556	NM_004468	2275
269	SPRR2A	A_33_P3260426	2.3777	0.020926	1.6793	0.38825	NM_005988	6700
270	GALNT14	A_23_P67847	2.3759	0.021016	1.6774	0.38825	NM_024572	79623
271	LOC643072	A_21_P0014102	2.3753	0.021047	1.6768	0.38825	XR_108434	NA
272	LRRN3	A_23_P31376	-2.3686	0.021397	1.6696	0.39255	NM_018334	54674
273	HRASLS	A_23_P57658	2.3678	0.021438	1.6688	0.39255	NM_020386	57110
274		A_33_P3387075	2.365	0.021586	1.6658	0.39382		NA
275	OLIG2	A_23_P211079	-2.3572	0.022	1.6576	0.39993	NM_005806	10215
276	PHACTR1	A_24_P943566	-2.3533	0.02221	1.6535	0.40227	AB051520	221692
277	CHRFAM7A	A_33_P3719083	-2.3499	0.022394	1.6499	0.40353	NM_139320	89832
278	XLOC_l2_015438	A_21_P0013747	-2.3491	0.022441	1.649	0.40353		NA
279	LOC100506546	A_21_P0014063	-2.3423	0.02281	1.6419	0.4087	XR_108325	NA
280	TTTY10	A_23_P320622	-2.3406	0.022905	1.6401	0.40894	NR_001542	246119

281	XLOC_005127	A_19_P00316820	2.338	0.023053	1.6373	0.41011	AK091028	NA
282	LOC100506930	A_21_P0011696	-2.3286	0.023583	1.6274	0.41805	NR_038279	NA
283	RHBG	A_23_P51690	2.3258	0.023745	1.6244	0.41943	NM_020407	57127
284		A_33_P3382887	2.3187	0.024156	1.617	0.42519		NA
285	HCRTR1	A_23_P74178	2.31	0.024665	1.6079	0.4299	NM_001525	3061
286		A_33_P3363305	2.309	0.024724	1.6069	0.4299		NA
287	LOC389906	A_21_P0013805	2.3082	0.024774	1.606	0.4299	NR_034031	NA
288	ALPL	A_24_P353619	2.3054	0.024942	1.6031	0.4299	NM_000478	249
289	EGR2	A_23_P46936	-2.304	0.025026	1.6016	0.4299	NM_000399	1959
290	XLOC_012332	A_21_P0009285	2.3034	0.02506	1.601	0.4299		NA
291		A_33_P3334428	2.303	0.025086	1.6006	0.4299		NA
292	XLOC_009337	A_21_P0007301	2.3026	0.025111	1.6001	0.4299		NA
293	WBSCR17	A_23_P111517	2.2972	0.025439	1.5945	0.4328	NM_022479	64409
294		A_33_P3370019	-2.2938	0.025647	1.591	0.4328	AK093943	NA
295	XLOC_014103	A_19_P00808865	-2.2928	0.025706	1.59	0.4328		NA
296	PRTFDC1	A_23_P202004	2.2928	0.025708	1.5899	0.4328	NM_020200	56952
297	LOC100506585	A_21_P0000855	-2.2926	0.025723	1.5897	0.4328	NR_038966	NA
298	LTBP1	A_23_P43810	2.291	0.02582	1.588	0.4328	NM_206943	4052
299	TPM1	A_24_P44462	2.2899	0.025887	1.5869	0.4328	NM_000366	7168
300	PNMA6A	A_33_P3292196	-2.2862	0.026121	1.583	0.43526	NM_032882	84968
301		A_33_P3244117	2.2845	0.026224	1.5813	0.43553	AK122666	NA
302	FLNA	A_33_P3408711	2.2826	0.026341	1.5794	0.43602	NM_001456	2316
303	XLOC_003839	A_21_P0003704	2.2788	0.026581	1.5754	0.43854		NA
304	MPL	A_24_P156769	2.2758	0.026777	1.5722	0.43917	NM_005373	4352
305	KREMEN1	A_33_P3262431	2.2742	0.026877	1.5706	0.43917	NM_032045	83999
306	TNS1	A_33_P3738458	2.2741	0.026883	1.5705	0.43917	NM_022648	7145
307	HIST1H2AC	A_23_P372860	2.271	0.027085	1.5673	0.43959	NM_003512	8334
308		A_33_P3224882	-2.2693	0.027194	1.5655	0.43959	Z11260	NA
309		A_33_P3364493	2.2647	0.02749	1.5608	0.43959		NA
310		A_24_P267686	2.2632	0.027594	1.5592	0.43959		NA
311	FLJ21408	A_21_P0000676	2.2626	0.02763	1.5586	0.43959	NR_037184	NA
312	PAGE2B	A_32_P109683	2.2619	0.027675	1.5579	0.43959	NM_001015038	389860
313	AOX2P	A_19_P00322315	2.2608	0.027747	1.5568	0.43959	NR_001557	NA
314	PF4	A_24_P79403	2.26	0.027805	1.5559	0.43959	NM_002619	5196
315	XLOC_010216	A_21_P0007773	2.2579	0.027941	1.5538	0.43959		NA
316	CDC20	A_23_P149200	2.2564	0.02804	1.5522	0.43959	NM_001255	991
317	OLFM4	A_24_P181254	2.2558	0.028083	1.5516	0.43959	NM_006418	10562
318	BPGM	A_23_P70843	2.2552	0.02812	1.551	0.43959	NM_199186	669
319	MAFIP	A_21_P0000766	2.2551	0.028128	1.5509	0.43959	NM_001190825	NA
320	GLP2R	A_33_P3398406	2.2549	0.028139	1.5507	0.43959	NM_004246	9340
321	XLOC_013623	A_21_P0009996	2.2494	0.028508	1.545	0.44275		NA
322	PVALB	A_23_P17844	2.2469	0.028681	1.5424	0.44275	NM_002854	5816
323	MALAT1	A_19_P00805006	-2.2436	0.028908	1.539	0.44275	NR_002819	NA
324	COL10A1	A_23_P214144	2.2413	0.02906	1.5367	0.44275	NM_000493	1300
325	TPM1	A_33_P3335966	2.2407	0.029101	1.5361	0.44275	NM_001018005	7168
326	DPPA5	A_32_P233950	2.2402	0.029136	1.5356	0.44275	NM_001025290	340168
327	NUDT13	A_23_P161324	2.2388	0.029234	1.5341	0.44275	NM_015901	25961

328	LOC100507254	A_21_P0004942	-2.2371	0.029355	1.5323	0.44275	NR_038981	NA	
329	PLSCR1	A_23_P69109	2.237	0.029362	1.5322	0.44275	NM_021105		5359
330	IGF2BP3	A_23_P19987	2.236	0.029431	1.5312	0.44275	NM_006547		10643
331	FAM46C	A_33_P3314176	2.2354	0.029471	1.5306	0.44275	NM_017709		54855
332	IGSF9B	A_33_P3325195	2.2332	0.029624	1.5284	0.44275	NM_014987		22997
333	HDC	A_23_P117662	-2.2327	0.02966	1.5278	0.44275	NM_002112		3067
334	TTTY12	A_23_P148641	2.2322	0.029697	1.5273	0.44275	NR_001551		83867
335	LOC100653060	A_32_P49668	2.232	0.029709	1.5271	0.44275	XM_003403393		100653060
336	XLOC_003276	A_21_P0003183	2.231	0.029777	1.5261	0.44275		NA	
337	XLOC_004308	A_21_P0003974	2.23	0.029847	1.5251	0.44275		NA	
338	XLOC_005847	A_21_P0005061	2.2267	0.030082	1.5217	0.44415		NA	
339	XLOC_002299	A_21_P0002097	2.2252	0.030186	1.5202	0.44415		NA	
340	G0S2	A_23_P74609	-2.2249	0.03021	1.5199	0.44415	NM_015714		50486
341	FKBP1A	A_23_P397238	-2.2237	0.030297	1.5186	0.44415	NM_054014		2280
342	TNFSF15	A_23_P94754	2.219	0.030628	1.5139	0.4473	NM_005118		9966
343	KCNH4	A_23_P124988	2.2182	0.030691	1.513	0.4473	NM_012285		23415
344	CST7	A_23_P68601	2.2161	0.030843	1.5108	0.44745	NM_003650		8530
345	PI16	A_33_P3215640	2.2156	0.03088	1.5103	0.44745	NM_153370		221476
346	LOC100505966	A_21_P0014164	2.2137	0.031014	1.5084	0.44808	XR_108660	NA	
347	RHOXF1	A_23_P85082	2.208	0.031435	1.5026	0.45287	NM_139282		158800
348	OPLAH	A_23_P170186	2.2059	0.031589	1.5005	0.45329	NM_017570		26873
349	HLA-DRB6	A_24_P169013	-2.2051	0.031646	1.4997	0.45329	NR_001298		3128
350	CHRNA7	A_32_P35969	-2.2038	0.03174	1.4984	0.45333	NM_001190455		1139
351	LOC100128252	A_32_P204795	-2.1995	0.032062	1.494	0.45643	NR_036522		100128252
352	KREMEN1	A_23_P211401	2.1985	0.032139	1.493	0.45643	NM_001039570		83999
353	TREM1	A_33_P3319905	-2.197	0.032253	1.4914	0.45675	NM_018643		54210
354	TPM1	A_23_P206018	2.1923	0.032609	1.4867	0.46049	NM_001018004		7168
355	LOC388588	A_33_P3394868	2.1898	0.032796	1.4842	0.46105	NM_001163724		388588
356	PBX1	A_24_P173823	2.1893	0.032834	1.4837	0.46105	NM_002585		5087
357	SCARNA6	A_33_P3230837	2.1874	0.032985	1.4817	0.46188	NR_003006		677772
358	XLOC_l2_015641	A_21_P0013798	2.185	0.033165	1.4793	0.46195		NA	
359	LOC155060	A_23_P254442	-2.1849	0.033174	1.4792	0.46195	NR_036573		155060
360	XLOC_005952	A_21_P0005343	2.1829	0.033331	1.4772	0.4624		NA	
361	CYP2B6	A_24_P339514	2.1821	0.033392	1.4764	0.4624	NM_000767		1555
362	CD177	A_21_P0011751	2.1747	0.033973	1.4689	0.46915	NM_020406	NA	
363	KCNG1	A_23_P210581	-2.1732	0.03409	1.4674	0.46946	NM_002237		3755
364	LOC641518	A_24_P213788	-2.169	0.034424	1.4631	0.47276	NR_029373		641518
365	LOC100506897	A_21_P0014234	-2.1666	0.034614	1.4607	0.47407	XR_133523	NA	
366	KRTAP19-2	A_33_P3311267	2.1643	0.034796	1.4585	0.47526	NM_181608		337969
367	ITM2C	A_24_P402690	-2.163	0.034905	1.4571	0.47545	NM_030926		81618
368	CD177	A_23_P259863	2.1616	0.03502	1.4557	0.47572	NM_020406		57126
369	MYB	A_23_P31073	-2.1573	0.035366	1.4514	0.47821	NM_005375		4602
370		A_33_P3806676	2.1569	0.035398	1.451	0.47821	AY751906	NA	
371	CD151	A_33_P3229196	2.1558	0.03549	1.4499	0.47821	NM_004357		977
372	RAB2B	A_23_P65466	2.1511	0.035874	1.4452	0.47997	NM_032846		84932
373	ACRBP	A_33_P3396370	2.1498	0.035982	1.4439	0.47997	NM_032489		84519
374	BTG2	A_23_P62901	-2.1492	0.036037	1.4433	0.47997	NM_006763		7832

375	MRGPRF	A_33_P3251984	2.1478	0.036148	1.4419	0.47997	NM_001098515	116535
376		A_33_P3250750	2.1471	0.036209	1.4412	0.47997	CU677518	NA
377	ASPSCR1	A_33_P3290588	2.1469	0.036226	1.441	0.47997	AK127159	79058
378	CD99L2	A_33_P3322892	2.1454	0.036353	1.4395	0.47997	BC025729	83692
379	PTCRA	A_33_P3229918	2.145	0.036389	1.439	0.47997	NM_001243168	171558
380	ANKFY1	A_33_P3300142	2.1432	0.036537	1.4373	0.48012	NM_016376	51479
381	MGAT3	A_24_P245838	-2.1423	0.036616	1.4363	0.48012	NM_002409	4248
382		A_33_P3370163	2.1408	0.036742	1.4348	0.48012		NA
383	NCR3	A_23_P251881	-2.1403	0.036785	1.4343	0.48012	NM_147130	259197
384	KIAA0040	A_33_P3243069	2.1389	0.036897	1.433	0.48034	NM_001162893	9674
385	LOC284263	A_33_P3663105	2.1306	0.037607	1.4247	0.4883	BC038429	284263
386	LOC401286	A_33_P3409238	-2.126	0.038007	1.4201	0.4903	XR_110199	401286
387	CD24	A_33_P3369844	-2.1252	0.038074	1.4194	0.4903	NM_013230	100133941
388	KRTAP15-1	A_24_P348845	2.1243	0.038152	1.4185	0.4903	NM_181623	254950
389	ARNT2	A_23_P83579	2.1241	0.038177	1.4182	0.4903	NM_014862	9915
390	XLOC_006980	A_21_P0005621	2.1232	0.038251	1.4174	0.4903	AK128880	NA
391	C9orf78	A_23_P9443	2.1216	0.03839	1.4158	0.49083	NM_016520	51759
392	GGT5	A_33_P3255304	-2.1183	0.038684	1.4125	0.49332	NM_001099781	2687
393		A_33_P3335401	-2.115	0.038979	1.4092	0.49546		NA
394	KIRREL	A_33_P3221489	2.1142	0.03905	1.4084	0.49546	NM_018240	55243
395	DACH1	A_33_P3316786	-2.1108	0.039351	1.405	0.49703	NM_080759	1602
396	XLOC_011047	A_21_P0008294	-2.1083	0.039576	1.4026	0.49703	BX648502	NA
397	PAQR6	A_23_P97283	2.1078	0.039622	1.4021	0.49703	NM_024897	79957
398	LOC100652851	A_33_P3313528	2.1075	0.039645	1.4018	0.49703	XM_003403468	100652851
399	CNKSR3	A_23_P134085	-2.1069	0.039702	1.4012	0.49703	NM_173515	154043
400	XLOC_l2_001826	A_21_P0010932	2.1061	0.03977	1.4004	0.49703	DA828100	NA
401	XLOC_012043	A_21_P0009138	2.1047	0.039898	1.399	0.49739		NA
402	ZSWIM7	A_33_P3269723	-2.1031	0.04005	1.3974	0.49803	NM_001042697	125150
403	LOC203274	A_33_P3474859	2.0986	0.040459	1.393	0.50157	BC110369	203274
404	XLOC_l2_009205	A_19_P00320814	2.0976	0.040547	1.392	0.50157		NA
405	GPR141	A_24_P334718	2.0957	0.040727	1.3901	0.50157	NM_181791	353345
406	PLXNB3	A_33_P3413038	2.0956	0.040736	1.39	0.50157	NM_001163257	5365
407	C12orf39	A_23_P2414	2.093	0.040975	1.3875	0.50246	AK311217	80763
408	FCGR1B	A_21_P0010728	2.0926	0.041009	1.3871	0.50246	NM_001244910	NA
409	CCDC64B	A_33_P3335590	2.0911	0.041156	1.3856	0.50271	NM_001103175	146439
410	SARM1	A_33_P3370763	2.0903	0.041231	1.3848	0.50271	NM_015077	23098
411		A_33_P3210258	-2.0886	0.041385	1.3832	0.50337	BC030554	NA
412	TMPRSS6	A_23_P211493	2.0865	0.041585	1.3811	0.50413	NM_153609	164656
413	FCGR1B	A_21_P0010561	2.0858	0.04165	1.3804	0.50413	NM_001244910	NA
414	CYB5R2	A_23_P2181	-2.084	0.041821	1.3786	0.50422	NM_016229	51700
415	EFNA3	A_24_P114032	2.0836	0.041858	1.3782	0.50422	NM_004952	1944
416	LUZP6	A_33_P3230090	2.0791	0.042283	1.3738	0.50811	NM_001128619	767558
417	XLOC_009336	A_21_P0007149	2.0755	0.042628	1.3703	0.51021		NA
418	PAGE2B	A_33_P3420862	2.0752	0.042662	1.37	0.51021	NM_001015038	389860
419	GNA12	A_24_P98086	2.0727	0.042899	1.3675	0.51173	NM_007353	2768
420		A_33_P3269380	2.0717	0.042997	1.3666	0.51173		NA
421	TEX22	A_33_P3261695	2.0707	0.043097	1.3656	0.51173	NM_001195082	647310

422	XLOC_007401	A_21_P0006151	2.0691	0.04325	1.364	0.51233		NA
423	CROCC	A_21_P0013793	2.0579	0.044348	1.3531	0.52411	NM_014675	NA
424	XLOC_005572	A_21_P0005151	2.0546	0.044683	1.3499	0.52682		NA
425	XLOC_001641	A_21_P0002608	2.0533	0.044809	1.3486	0.52706		NA
426	JAK3	A_23_P329112	2.0517	0.044973	1.347	0.52775	NM_000215	3718
427	CXCR2P1	A_33_P3375859	2.0499	0.045151	1.3453	0.52827	NR_002712	3580
428	SLC4A10	A_24_P930111	-2.0481	0.045334	1.3436	0.52827	NM_022058	57282
429	XLOC_006314	A_21_P0005271	2.0478	0.045363	1.3433	0.52827	AI198554	NA
430	SYTL4	A_24_P122337	2.0469	0.045454	1.3424	0.52827	NM_080737	94121
431	GPR97	A_33_P3305102	2.046	0.045546	1.3416	0.52827	NM_170776	222487
432	DCAF12	A_23_P255257	2.0431	0.045841	1.3387	0.53046	NM_015397	25853
433	ABP1	A_23_P59452	-2.041	0.046062	1.3367	0.53099	NM_001091	26
434		A_33_P3258324	-2.0406	0.046099	1.3363	0.53099		NA
435	CAMKK1	A_23_P431933	-2.0393	0.046236	1.335	0.53134	NM_032294	84254
436	NFKBID	A_23_P383422	-2.036	0.046577	1.3318	0.5336	NM_139239	84807
437	14-Sep	A_21_P0011897	2.0348	0.046698	1.3307	0.5336	NM_207366	NA
438	SLC4A10	A_24_P314786	-2.0339	0.046801	1.3297	0.5336	NM_022058	57282
439	CPA3	A_23_P18017	-2.033	0.046889	1.3289	0.5336	NM_001870	1359
440	TLR5	A_23_P85903	2.0319	0.047007	1.3278	0.5336	NM_003268	7100
441	FKBP8	A_33_P3272990	2.0313	0.047073	1.3272	0.5336	NM_012181	23770
442		A_33_P3370521	2.0283	0.047383	1.3244	0.5359	XM_001721393	NA
443	INHBB	A_23_P153964	2.027	0.047519	1.3231	0.53623	NM_002193	3625
444	XLOC_l2_008976	A_21_P0012167	2.024	0.047842	1.3202	0.53865		NA
445	OLIG1	A_23_P154849	-2.0218	0.048078	1.3181	0.5401	NM_138983	116448
446		A_33_P3325871	2.0207	0.048195	1.317	0.5402	BC004487	NA
447		A_33_P3358347	2.018	0.048477	1.3145	0.54117		NA
448		A_33_P3233819	2.0162	0.048675	1.3127	0.54117		NA
449		A_33_P3312799	2.0153	0.048774	1.3118	0.54117		NA
450	C1orf186	A_23_P95640	-2.0153	0.048774	1.3118	0.54117	NM_001007544	440712
451	KIAA1530	A_33_P3287113	2.0146	0.04885	1.3111	0.54117	AL390128	57654
452	ADAMTS17	A_33_P3309626	2.0138	0.048932	1.3104	0.54117	AK131344	170691
453		A_33_P3285371	2.0107	0.049273	1.3074	0.54374		NA
454	XLOC_002125	A_21_P0002057	-2.006	0.049787	1.3029	0.54657	AL355732	NA

Total in Data 1	535
Total in Data 2	454
Unique in Data 1	532
Unique in Data 2	373
Common in both	9
