# Supplementary material for: Association between Arsenic Level, Gene Expression in Asian Population, and In Vitro Carcinogenic Bladder Tumor
Source: Oxid Med Cell Longev. 2022 Jan 7;2022:3459855. doi: 10.1155/2022/3459855 (PMC8760535; doi:10.1155/2022/3459855)
Supplement: Supplementary 6 — Table S5: significant pathways in Data1 (low, high concentration) and Data2 (low, medium, and high concentrations) and the overlap between two (highlighted in pink). [file 3459855.f6.pdf]

Table S5. Significant pathways in Data-1(low, high concentration) and Data-2 (low, medium, high concentrations) and the overlap between two (highlighted in pink).

Data1

S.No.	Ingenuity Canonical Pathways	-log(p-value)	Ratio	Molecules
1	Pyridoxal 5'-phosphate Salvage Pathway	6.90E+00	1.67E-01	AKT2,BRAF,CDK1,CDK5,DAPK1,MAP2K4,PAK1,PIM1,PLK1,PRKCQ,PRKX
2	Salvage Pathways of Pyrimidine Ribonucleotides	5.97E+00	1.22E-01	AKT2,APOBEC3G,BRAF,CDK1,CDK5,DAPK1,MAP2K4,PAK1,PIM1,PLK1,PRKCQ,PRKX
3	Th1 and Th2 Activation Pathway	5.43E+00	8.77E-02	GFI1,HLA-DMA,HLA-DMB,HLA-DQB2,ICOS,IFNG,IKZF1,IL18,JAK3,mir-29,PRKCQ,PSEN1,PTGDR2,S1PR1,STAT4
4	Sperm Motility	5.24E+00	7.62E-02	DSTYK,EPHB3,ERBB2,FES,ITK,JAK3,LCK,MAP2K4,MAP3K11,MST1R,NTRK2,PDE4B,PLA2G4A,PLA2G7,PLCB1,PRKCQ,TNK1
5	Th2 Pathway	5.19E+00	9.56E-02	GFI1,HLA-DMA,HLA-DMB,HLA-DQB2,ICOS,IFNG,IKZF1,JAK3,PRKCQ,PSEN1,PTGDR2,S1PR1,STAT4
6	Systemic Lupus Erythematosus In B Cell Signaling Pathway	5.16E+00	6.91E-02	AKT2,BCL2,CBL,CHP1,IFNG,IGHA1,IGHG1,IL17A,IL18,IRF5,LCK,LTB,MYC,PIM2,PRKCQ,RAP1B,TLR8,TNFSF13,TNFSF13B
7	IL-15 Production	5.00E+00	9.92E-02	DSTYK,EPHB3,ERBB2,FES,ITK,JAK3,LCK,MAP2K4,MAP3K11,MST1R,NTRK2,TNK1
8	Altered T Cell and B Cell Signaling in Rheumatoid Arthritis	4.69E+00	1.11E-01	HLA-DMA,HLA-DMB,IFNG,IL17A,IL18,LTB,TLR8,TNFSF13,TNFSF13B,TRGV9
9	T Helper Cell Differentiation	4.64E+00	1.23E-01	HLA-DMA,HLA-DMB,ICOS,IFNG,IL17A,IL18,IL21R,STAT4,TRGV9
10	NF-κB Signaling	4.54E+00	7.82E-02	AKT2,BRAF,IL18,IL1R1,IRAK3,LCK,LTBR,MAP3K3,NTRK2,PRKCQ,RAP1B,TLR8,TNFSF13B,TRGV9
11	Dendritic Cell Maturation	4.44E+00	7.65E-02	AKT2,CD1C,CD1D,HLA-DMA,HLA-DMB,IGHG1,IL18,IRF8,LTB,LTBR,MAP2K4,PLCB1,STAT4,TRGV9
12	Prostanoid Biosynthesis	4.43E+00	4.00E-01	CYP2S1,HPGDS,PTGDS,TBXAS1
13	Role of MaAcrrtohprihtiasges, Fibroblasts and Endothelial Cells in Rheumatoid	4.40E+00	6.09E-02	AKT2,CCL2,CHP1,IGHG1,IL17A,IL18,IL1R1,IRAK3,LTB,LTBR,MAP2K4,MYC,PLCB1,PRKCQ,RAP1B,TLR8,TNFSF13B,VEGFA,WNT5B
14	Cardiac Hypertrophy Signaling (Enhanced)	4.34E+00	5.13E-02	AKT2,CACNA1C,CHP1,FGF13,GNG5,HDAC3,HDAC9,IFNG,IL13RA1,IL17A,IL18,IL1R1,IL21R,LTB,MAP2K4,MAP3K11,MAP3K3,MYC,PDE4B,PLCB1,PRKCQ,RAP1B,TNFSF13,TNFSF13B,WNT5B
15	CD28 Signaling in T Helper Cells	4.31E+00	9.17E-02	AKT2,CDC42,CHP1,HLA-DMA,HLA-DMB,ITK,LCK,MAP2K4,PAK1,PRKCQ,TRGV9
16	Th1 Pathway	4.28E+00	9.09E-02	HLA-DMA,HLA-DMB,HLA-DQB2,ICOS,IFNG,IL18,JAK3,mir-29,PRKCQ,PSEN1,STAT4
17	EIF2 Signaling	4.05E+00	6.70E-02	ACTA2,AKT2,BCL2,EIF1AX,EIF2S1,MYC,RAP1B,RPL13,RPS20,RPS26,RPS27A,RPS4X,RPSA,VEGFA,WARS1
18	Nur77 Signaling in T Lymphocytes	4.04E+00	1.18E-01	BCL2,CHP1,HDAC9,MAP3K3,PCNA,PRKCQ,RXRA,TRGV9
19	STAT3 Pathway	3.85E+00	8.15E-02	BCL2,IL13RA1,IL1R1,IL21R,MAP2K4,MAP3K11,MYC,NTRK2,PIM1,RAP1B,VEGFA
20	Tec Kinase Signaling	3.70E+00	7.32E-02	ACTA2,CDC42,FCER1A,GNG5,ITK,JAK3,LCK,MAP2K4,PAK1,PRKCQ,STAT4,TRGV9
21	HMGB1 Signaling	3.68E+00	7.27E-02	AKT2,CCL2,CDC42,IFNG,IL17A,IL18,IL1R1,LTB,MAP2K4,RAP1B,TNFSF13,TNFSF13B
22	Cdc42 Signaling	3.63E+00	7.19E-02	BAIAP2,CDC42,EXOC6,HLA-DMA,HLA-DMB,HLA-DQB2,ITK,MAP2K4,MAP3K11,PAK1,RASA1,TRGV9
23	Molecular Mechanisms of Cancer	3.57E+00	5.12E-02	AKT2,BCL2,BRAF,CBL,CCNE2,CDC42,CDK1,CDK5,JAK3,MAP2K4,MYC,PAK1,PLCB1,PRKCQ,PSEN1,RAP1B,RASA1,SMAD5,SMAD9,WNT5B
24	Role of MAPK Signaling in the Pathogenesis of Influenza	3.55E+00	1.00E-01	AKT2,BCL2,CCL2,IFNG,MAP2K4,PLA2G4A,PLA2G7,RAP1B
25	Natural Killer Cell Signaling	3.52E+00	6.60E-02	AKT2,CDC42,HSPA2,IFNG,IL18,JAK3,LCK,MAP3K11,MAP3K3,PAK1,PRKCQ,RAP1B,STAT4

Total in Data 1	180
Total in Data 2	50
Common in both	14
Unique in Data 1	166
Unique in Data 2	36

26	T Cell Exhaustion Signaling Pathway	3.44E+00	6.86E-02	AKT2,HLA-DMA,HLA-DMB,HLA-DQB2,IFNG,JAK3,MAP2K4,PRKCQ,RAP1B,STAT4,TRGV9,VEGFA
27	HER-2 Signaling in Breast Cancer	3.40E+00	9.52E-02	AKT2,CCNE2,CDC42,ERBB2,ITGB6,NRG1,PRKCQ,RAP1B
28	PKCθ Signaling in T Lymphocytes	3.33E+00	7.10E-02	CACNA1C,CHP1,HLA-DMA,HLA-DMB,LCK,MAP2K4,MAP3K11,MAP3K3,PRKCQ,RAP1B,TRGV9
29	Mitotic Roles of Polo-Like Kinase	3.32E+00	1.06E-01	CCNB2,CDK1,FBXO5,KIF11,PLK1,PLK4,SMC1A
30	Role of NFAT in Regulation of the Immune Response	3.31E+00	6.63E-02	AKT2,CHP1,FCER1A,GNG5,HLA-DMA,HLA-DMB,ITK,LCK,PLCB1,PRKCQ,RAP1B,TRGV9
31	IL-12 Signaling and Production in Macrophages	3.30E+00	7.58E-02	AKT2,APOE,IFNG,IL18,IRF8,MAP2K4,MST1R,PRKCQ,RXRA,STAT4
32	iCOS-iCOSL Signaling in T Helper Cells	3.23E+00	8.11E-02	AKT2,CHP1,HLA-DMA,HLA-DMB,ICOS,ITK,LCK,PRKCQ,TRGV9
33	HGF Signaling	3.23E+00	8.11E-02	AKT2,CDC42,ETS2,MAP2K4,MAP3K11,MAP3K3,PAK1,PRKCQ,RAP1B
34	Acute Myeloid Leukemia Signaling	3.23E+00	8.99E-02	AKT2,BRAF,IDH1,MAP2K4,MYC,PIM1,PIM2,RAP1B
35	DNA damage-induced 14-3-3σ Signaling	3.23E+00	2.11E-01	AKT2,CCNB2,CCNE2,CDK1
36	Neuroinflammation Signaling Pathway	3.17E+00	5.35E-02	AKT2,BCL2,CCL2,CHP1,HLA-DMA,HLA-DMB,IFNG,IL18,IL1R1,IRAK3,JAK3,MAP2K4,NCF2,PLA2G4A,PSEN1,TLR8
37	Production of Nitric Oxide and Reactive Oxygen Species in Macrophages	3.17E+00	6.38E-02	AKT2,APOE,CDC42,IFNG,IRF8,JAK3,MAP2K4,MAP3K11,MAP3K3,NCF2,PRKCQ,RAP1B
38	Th17 Activation Pathway	3.16E+00	8.79E-02	IFNG,IL17A,IL1R1,IL21R,IRAK3,JAK3,STAT4,TRGV9
39	Fcγ Receptor-mediated Phagocytosis in Macrophages and Monocytes	3.07E+00	8.51E-02	ACTA2,AKT2,CBL,CDC42,PAK1,PIP5K1A,PLD3,PRKCQ
40	Airway Pathology in Chronic Obstructive Pulmonary Disease	3.04E+00	7.63E-02	CCL2,FGF13,IFNG,IL17A,IL18,LTB,PTGDS,TNFSF13,TNFSF13B
41	Hepatic Fibrosis Signaling Pathway	3.04E+00	4.89E-02	ACTA2,AKT2,BCL2,BRAF,CACNA1C,CCL2,CDC42,IL17A,IL18,IL1R1,IRAK3,MAP2K4,MYC,NCF2,PRKCQ,RAP1B,VEGFA,WNT5B
42	Neuregulin Signaling	3.01E+00	8.33E-02	AKT2,CDK5,ERBB2,MYC,NRG1,PRKCQ,PSEN1,RAP1B
43	Communication between Innate and Adaptive Immune Cells	3.01E+00	8.33E-02	IFNG,IGHA1,IGHG1,IL18,TLR8,TNFSF13,TNFSF13B,TRGV9
44	IL-8 Signaling	2.93E+00	6.00E-02	AKT2,BCL2,BRAF,CDC42,GNG5,IRAK3,MAP2K4,NCF2,PLD3,PRKCQ,RAP1B,VEGFA
45	Phospholipase C Signaling	2.92E+00	5.45E-02	CDC42,CHP1,GNG5,HDAC3,HDAC9,IGHG1,ITK,LCK,PLA2G4A,PLCB1,PLD3,PRKCQ,RAP1B,TRGV9
46	Agrin Interactions at Neuromuscular Junction	2.89E+00	8.97E-02	ACTA2,CDC42,ERBB2,MAP2K4,NRG1,PAK1,RAP1B
47	BEX2 Signaling Pathway	2.85E+00	8.86E-02	AKT2,BCL2,ERBB2,ITGB6,MAP2K4,SMARCB1,VEGFA
48	SAPK/JNK Signaling	2.84E+00	7.84E-02	CDC42,GNG5,LCK,MAP2K4,MAP3K11,MAP3K3,RAP1B,TRGV9
49	Role of Pattern Recognition Receptors in Recognition of Bacteria and Viruses	2.78E+00	6.49E-02	EIF2S1,IFNG,IL17A,IL18,LTB,MAP2K4,PRKCQ,TLR8,TNFSF13,TNFSF13B
50	Huntington's Disease Signaling	2.78E+00	5.49E-02	AKT2,CDK5,GNG5,HDAC3,HDAC9,HSPA2,MAP2K4,PLCB1,PRKCQ,PSME2,RASA1,RPS27A,SH3GL3
51	mTOR Signaling	2.75E+00	5.71E-02	AKT2,CDC42,FKBP1A,PLD3,PRKCQ,RAP1B,RPS20,RPS26,RPS27A,RPS4X,RPSA,VEGFA
52	PD-1, PD-L1 cancer immunotherapy pathway	2.73E+00	7.55E-02	AKT2,HLA-DMA,HLA-DMB,HLA-DQB2,IFNG,JAK3,LCK,PRKCQ
53	Hepatic Cholestasis	2.70E+00	5.95E-02	IFNG,IL17A,IL18,IL1R1,IRAK3,LTB,MAP2K4,PRKCQ,RXRA,TNFSF13,TNFSF13B
54	Pancreatic Adenocarcinoma Signaling	2.66E+00	7.34E-02	AKT2,BCL2,CDC42,ERBB2,JAK3,MAP2K4,PLD3,VEGFA
55	RegulationP aOtfh Twhaey Epithelial Mesenchymal Transition By Growth Factors	2.65E+00	5.85E-02	AKT2,BRAF,CDC42,ERBB2,FGF13,JAK3,LTB,MAP2K4,RAP1B,TNFSF13,TNFSF13B
56	Role of Osteoblasts, Osteoclasts and Chondrocytes in Rheumatoid Arthritis	2.59E+00	5.45E-02	AKT2,BCL2,CBL,CHP1,IFNG,IL17A,IL18,IL1R1,MAP2K4,SMAD5,SMAD9,WNT5B
57	Rac Signaling	2.58E+00	7.14E-02	BAIAP2,CDC42,MAP2K4,MAP3K11,NCF2,PAK1,PIP5K1A,RAP1B

58	Calcium-induced T Lymphocyte Apoptosis	2.57E+00	9.09E-02	CHP1,HLA-DMA,HLA-DMB,LCK,PRKCQ,TRGV9
59	Eicosanoid Signaling	2.57E+00	9.09E-02	HPGDS,LTA4H,PLA2G4A,PLA2G7,PTGDS,TBXAS1
60	Colorectal Cancer Metastasis Signaling	2.53E+00	5.14E-02	AKT2,BRAF,CDC42,GNG5,IFNG,JAK3,MAP2K4,MYC,RAP1B,SIAH1,TLR8,VEGFA,WNT5B
61	fMLP Signaling in Neutrophils	2.49E+00	6.90E-02	CDC42,CHP1,FPR3,GNG5,NCF2,PLCB1,PRKCQ,RAP1B
62	Graft-versus-Host Disease Signaling	2.48E+00	1.04E-01	HLA-DMA,HLA-DMB,IFNG,IL18,TRGV9
63	ErbB Signaling	2.42E+00	7.45E-02	CDC42,ERBB2,MAP2K4,NRG1,PAK1,PRKCQ,RAP1B
64	GNRH Signaling	2.42E+00	5.78E-02	CACNA1C,CDC42,GNG5,MAP2K4,MAP3K11,MAP3K3,PAK1,PLCB1,PRKCQ,RAP1B
65	Primary Immunodeficiency Signaling	2.40E+00	1.00E-01	ICOS,IGHA1,IGHG1,JAK3,LCK
66	Ephrin B Signaling	2.38E+00	8.33E-02	CBL,CDC42,EPHB3,GNG5,ITSN1,PAK1
67	LXR/RXR Activation	2.38E+00	6.61E-02	APOE,CCL2,HMGCR,IL18,IL1R1,MSR1,RXRA,SCD
68	HIF1α Signaling	2.36E+00	5.37E-02	AKT2,BRAF,CHP1,HK2,HSPA2,IL17A,MAP2K4,NCF2,PRKCQ,RAP1B,VEGFA
69	VEGF Signaling	2.30E+00	7.07E-02	ACTA2,AKT2,BCL2,EIF1AX,EIF2S1,RAP1B,VEGFA
70	IL-15 Signaling	2.30E+00	8.00E-02	AKT2,IL17A,JAK3,LCK,MYC,RAP1B
71	Transcriptional Regulatory Network in Embryonic Stem Cells	2.26E+00	9.26E-02	GATA6,H4C2,H4C8,H4C9,ZFHX3
72	Protein Ubiquitination Pathway	2.25E+00	4.76E-02	CBL,DNAJC30,HSPA2,IFNG,PSMD10,PSMD7,PSME2,RPS27A,UBE2A,UBE2E2,UBE2T,UBE3A,USP18
73	Integrin Signaling	2.24E+00	5.16E-02	ACTA2,AKT2,ARF3,BRAF,CDC42,ITGB6,MAP2K4,MAP3K11,PAK1,RAP1B,TSPAN5
74	Senescence Pathway	2.23E+00	4.73E-02	AKT2,ASXL3,BRAF,CACNA1C,CCNB2,CDK1,CHP1,ETS2,MAP2K4,RAP1B,SMAD5,SMAD9,YPEL3
75	IL-7 Signaling Pathway	2.21E+00	7.69E-02	AKT2,BCL2,IFNG,IGHG1,JAK3,MYC
76	Reelin Signaling in Neurons	2.21E+00	6.20E-02	AKT2,APOE,CDC42,CDK5,LCK,MAP2K4,MAP3K11,RAP1B
77	NER Pathway	2.21E+00	6.80E-02	H4C2,H4C8,H4C9,PCNA,POLD2,RFC4,TOP2A
78	Regulation of eIF4 and p70S6K Signaling	2.20E+00	5.73E-02	AKT2,EIF1AX,EIF2S1,RAP1B,RPS20,RPS26,RPS27A,RPS4X,RPSA
79	Tryptophan Degradation to 2-amino-3-carboxymuconate Semialdehyde	2.20E+00	3.33E-01	HAAO,KYNU
80	Semaphorin Neuronal Repulsive Signaling Pathway	2.19E+00	6.15E-02	AKT2,CDK5,ERBB2,FES,MAP2K4,PAK1,PDE4B,VCAN
81	Cell Cycle Control of Chromosomal Replication	2.19E+00	8.93E-02	CDK1,CDK5,MCM8,PCNA,TOP2A
82	T Cell Receptor Signaling	2.16E+00	6.67E-02	CBL,ITK,LCK,MAP2K4,PRKCQ,RAP1B,RASA1
83	Renal Cell Carcinoma Signaling	2.16E+00	7.50E-02	AKT2,CDC42,PAK1,RAP1B,RPS27A,VEGFA
84	GADD45 Signaling	2.15E+00	1.58E-01	CCNE2,CDK1,PCNA
85	MSP-RON Signaling Pathway	2.12E+00	8.62E-02	ACTA2,CCL2,IFNG,KLK2,MST1R
86	Axonal Guidance Signaling	2.12E+00	3.93E-02	AKT2,BAIAP2,CDC42,CDK5,CHP1,EPHB3,ERBB2,FES,GNG5,ITSN1,NTRK2,PAK1,PLCB1,PRKCQ,RAP1B,RASA1,SEMA3C,VEGFA,WNT5B
87	Clathrin-mediated Endocytosis Signaling	2.09E+00	5.18E-02	ACTA2,APOE,CBL,CDC42,CHP1,FGF13,ITGB6,RPS27A,SH3GL3,VEGFA
88	RAR Activation	2.07E+00	5.15E-02	AKT2,MAP2K4,PNPLA4,PRKCQ,RDH13,RXRA,SMAD5,SMAD9,SMARCB1,VEGFA
89	Antigen Presentation Pathway	2.05E+00	1.03E-01	HLA-DMA,HLA-DMB,HLA-DQB2,IFNG
90	IL-4 Signaling	2.04E+00	7.06E-02	AKT2,HLA-DMA,HLA-DMB,IL13RA1,JAK3,RAP1B
91	Type I Diabetes Mellitus Signaling	2.03E+00	6.31E-02	BCL2,HLA-DMA,HLA-DMB,IFNG,IL1R1,MAP2K4,TRGV9
92	Allograft Rejection Signaling	2.01E+00	6.98E-02	HLA-DMA,HLA-DMB,HLA-DQB2,IFNG,IGHG1,TRGV9
93	Hereditary Breast Cancer Signaling	2.01E+00	5.71E-02	AKT2,CDK1,HDAC3,HDAC9,RAP1B,RFC4,RPS27A,SMARCB1
94	RANK Signaling in Osteoclasts	1.97E+00	6.82E-02	AKT2,CBL,CHP1,MAP2K4,MAP3K11,MAP3K3
95	Xenobiotic Metabolism General Signaling Pathway	1.95E+00	5.59E-02	AKT2,MAP2K4,MAP3K11,MAP3K3,MGST1,PRKCQ,RAP1B,RXRA
96	Phospholipases	1.95E+00	7.81E-02	PLA2G15,PLA2G4A,PLA2G7,PLCB1,PLD3

97	Salvage Pathways of Pyrimidine Deoxyribonucleotides	1.94E+00	2.50E-01	APOBEC3G,TK2
98	OX40 Signaling Pathway	1.92E+00	6.67E-02	BCL2,HLA-DMA,HLA-DMB,HLA-DQB2,MAP2K4,TRGV9
99	ErbB2-ErbB3 Signaling	1.92E+00	7.69E-02	ERBB2,JAK3,MYC,NRG1,RAP1B
100	DifferentiIaLl -R1e7gAu alantdio InL -o1f7 CFytokine Production in Intestinal Epithelial Cells by	1.92E+00	1.30E-01	CCL2,IFNG,IL17A
101	Tryptophan Degradation III (Eukaryotic)	1.92E+00	1.30E-01	CYP2S1,HAAO,KYNU
102	Role of Tissue Factor in Cancer	1.91E+00	5.98E-02	AKT2,CDC42,LCK,PAK1,PLCB1,RAP1B,VEGFA
103	Role of HyInpeflruceyntozakinemia/hyperchemokinemia in the Pathogenesis of	1.90E+00	9.30E-02	CCL2,IFNG,IL17A,IL18
104	BAG2 Signaling Pathway	1.90E+00	9.30E-02	ANXA2,HSPA2,MYC,PSME2
105	p38 MAPK Signaling	1.90E+00	5.93E-02	H3C3,IL18,IL1R1,IRAK3,MAP2K4,MYC,PLA2G4A
106	Role of NANOG in Mammalian Embryonic Stem Cell Pluripotency	1.88E+00	5.88E-02	AKT2,GATA6,JAK3,RAP1B,SMAD5,SMAD9,WNT5B
107	Glutathione Redox Reactions I	1.87E+00	1.25E-01	GPX1,GPX7,MGST1
108	Cell Cycle: G1/S Checkpoint Regulation	1.87E+00	7.46E-02	CCNE2,HDAC3,HDAC9,MYC,NRG1
109	Coronavirus Pathogenesis Pathway	1.84E+00	5.33E-02	BCL2,CCL2,MAP2K4,RPS20,RPS26,RPS27A,RPS4X,RPSA
110	Ephrin Receptor Signaling	1.84E+00	5.00E-02	AKT2,CDC42,EPHB3,GNG5,ITSN1,PAK1,RAP1B,RASA1,VEGFA
111	Insulin Secretion Signaling Pathway	1.84E+00	4.53E-02	CACNA1C,EIF2S1,JAK3,LCK,PC,PLCB1,PRKCQ,PRLR,RAP1B,SEC11A,STAT4
112	G Beta Gamma Signaling	1.82E+00	5.74E-02	AKT2,CACNA1C,CDC42,GNG5,PAK1,PRKCQ,RAP1B
113	Inhibition of ARE-Mediated mRNA Degradation Pathway	1.82E+00	5.74E-02	AKT2,DDX6,LTB,PSME2,TIA1,TNFSF13,TNFSF13B
114	Signaling by Rho Family GTPases	1.82E+00	4.51E-02	ACTA2,BAIAP2,CDC42,GNG5,MAP2K4,MAP3K11,NCF2,PAK1,PIP5K1A,RDX,SEPTIN6
115	Role of NFAT in Cardiac Hypertrophy	1.80E+00	4.67E-02	AKT2,CACNA1C,CHP1,GNG5,HDAC3,HDAC9,MAP2K4,PLCB1,PRKCQ,RAP1B
116	TGF-β Signaling	1.79E+00	6.25E-02	BCL2,CDC42,MAP2K4,RAP1B,SMAD5,SMAD9
117	Opioid Signaling Pathway	1.79E+00	4.45E-02	AKT2,BRAF,CACNA1C,CDC42,GNG5,LCK,MAP2K4,MYC,PLCB1,PRKCQ,RAP1B
118	Bladder Cancer Signaling	1.77E+00	6.19E-02	DAPK1,ERBB2,FGF13,MYC,RAP1B,VEGFA
119	Lipid Antigen Presentation by CD1	1.77E+00	1.15E-01	CD1C,CD1D,TRGV9
120	Estrogen-mediated S-phase Entry	1.77E+00	1.15E-01	CCNE2,CDK1,MYC
121	Phagosome Formation	1.77E+00	5.60E-02	CDC42,FCER1A,IGHG1,MSR1,PLCB1,PRKCQ,TLR8
122	Small Cell Lung Cancer Signaling	1.77E+00	7.04E-02	AKT2,BCL2,CCNE2,MYC,RXRA
123	Sertoli Cell-Sertoli Cell Junction Signaling	1.77E+00	4.86E-02	ACTA2,AKT2,CDC42,F11R,MAP2K4,MAP3K11,MAP3K3,RAP1B,SPTAN1
124	B Cell Receptor Signaling	1.77E+00	4.86E-02	AKT2,CDC42,IGHA1,IGHG1,MAP2K4,MAP3K11,MAP3K3,PRKCQ,RAP1B
125	Atherosclerosis Signaling	1.74E+00	5.51E-02	APOE,CCL2,IFNG,IL18,MSR1,PLA2G4A,PLA2G7
126	Apoptosis Signaling	1.73E+00	6.06E-02	BCL2,CDK1,MAP2K4,PRKCQ,RAP1B,SPTAN1
127	Autoimmune Thyroid Disease Signaling	1.71E+00	8.16E-02	HLA-DMA,HLA-DMB,IGHG1,TRGV9
128	Cell Cycle: G2/M DNA Damage Checkpoint Regulation	1.71E+00	8.16E-02	CCNB2,CDK1,PLK1,TOP2A
129	HOTAIR Regulatory Pathway	1.69E+00	5.00E-02	AKT2,ERBB2,H3C3,HOXD10,let-7,mir-29,MYC,WNT5B
130	Mouse Embryonic Stem Cell Pluripotency	1.66E+00	5.83E-02	AKT2,JAK3,MYC,RAP1B,SMAD5,SMAD9
131	Toll-like Receptor Signaling	1.65E+00	6.58E-02	IL18,IRAK3,MAP2K4,RPS27A,TLR8
132	Macropinocytosis Signaling	1.65E+00	6.58E-02	CDC42,ITGB6,PAK1,PRKCQ,RAP1B
133	UVC-Induced MAPK Signaling	1.65E+00	7.84E-02	BRAF,MAP2K4,PRKCQ,RAP1B

134	Estrogen Receptor Signaling	1.64E+00	3.96E-02	AKT2,BCL2,CACNA1C,GNG5,HDAC3,JAK3,MYC,PAK1,PCNA,PLCB1,PRKCQ,RAP1B,VEGFA
135	Systemic Lupus Erythematosus Signaling	1.62E+00	4.37E-02	AKT2,CBL,IGHG1,IL18,LCK,LSM7,PIM2,RAP1B,TNFSF13B,TRGV9
136	Leukocyte Extravasation Signaling	1.61E+00	4.57E-02	ACTA2,CDC42,F11R,ITK,MAP2K4,NCF2,PRKCQ,RAP1B,RDX
137	Glucose and Glucose-1-phosphate Degradation	1.59E+00	1.67E-01	HK2,RGN
138	NAD biosynthesis II (from tryptophan)	1.59E+00	1.67E-01	HAAO,KYNU
139	CXCR4 Signaling	1.59E+00	4.79E-02	AKT2,CDC42,GNG5,MAP2K4,PAK1,PLCB1,PRKCQ,RAP1B
140	IL-3 Signaling	1.59E+00	6.33E-02	AKT2,CHP1,PAK1,PRKCQ,RAP1B
141	Thyroid Cancer Signaling	1.59E+00	6.33E-02	AKT2,BRAF,MYC,NTRK2,RAP1B
142	Virus Entry via Endocytic Pathways	1.59E+00	5.61E-02	ACTA2,CDC42,ITGB6,ITSN1,PRKCQ,RAP1B
143	Telomerase Signaling	1.59E+00	5.61E-02	AKT2,ETS2,HDAC3,HDAC9,MYC,RAP1B
144	Systemic Lupus Erythematosus In T Cell Signaling Pathway	1.59E+00	3.89E-02	AKT2,CBL,CDC42,CHP1,HLA-DMA,HLA-DMB,HLA-DQB2,ICOS,IL17A,MAP2K4,RAP1B,RDX,TRGV9
145	IL-17 Signaling	1.57E+00	6.25E-02	AKT2,CCL2,IL17A,MAP2K4,RAP1B
146	G Protein Signaling Mediated by Tubby	1.57E+00	9.68E-02	GNG5,LCK,PLCB1
147	Paxillin Signaling	1.57E+00	5.56E-02	ACTA2,CDC42,ITGB6,MAP2K4,PAK1,RAP1B
148	Prolactin Signaling	1.55E+00	6.17E-02	MYC,NMI,PRKCQ,PRLR,RAP1B
149	Cyclins and Cell Cycle Regulation	1.55E+00	6.17E-02	CCNB2,CCNE2,CDK1,HDAC3,HDAC9
150	UDP-N-acetyl-D-galactosamine Biosynthesis II	1.53E+00	1.54E-01	HK2,UAP1
151	LPS-stimulated MAPK Signaling	1.53E+00	6.10E-02	CDC42,MAP2K4,PAK1,PRKCQ,RAP1B
152	Role of CHK Proteins in Cell Cycle Checkpoint Control	1.49E+00	7.02E-02	CDK1,PCNA,PLK1,RFC4
153	VEGF Family Ligand-Receptor Interactions	1.49E+00	5.95E-02	AKT2,PLA2G4A,PRKCQ,RAP1B,VEGFA
154	NGF Signaling	1.47E+00	5.26E-02	AKT2,CDC42,MAP2K4,MAP3K11,MAP3K3,RAP1B
155	Cancer Drug Resistance By Drug Efflux	1.47E+00	6.90E-02	ABCG2,AKT2,BRAF,RAP1B
156	Synaptogenesis Signaling Pathway	1.47E+00	3.85E-02	AKT2,APOE,BRAF,CADM1,CDC42,CDK5,EPHB3,ITSN1,LCK,NTRK2,PAK1,RAP1B
157	Inhibition of Angiogenesis by TSP1	1.46E+00	8.82E-02	AKT2,MAP2K4,VEGFA
158	Apelin Endothelial Signaling Pathway	1.45E+00	5.22E-02	AKT2,CCL2,MAP2K4,PLCB1,PRKCQ,RAP1B
159	PDGF Signaling	1.45E+00	5.81E-02	JAK3,MAP2K4,MYC,RAP1B,RASA1
160	DNA Methylation and Transcriptional Repression Signaling	1.43E+00	8.57E-02	H4C2,H4C8,H4C9
161	Fc Epsilon RI Signaling	1.42E+00	5.13E-02	AKT2,FCER1A,MAP2K4,PLA2G4A,PRKCQ,RAP1B
162	Role of PKR in Interferon Induction and Antiviral Response	1.42E+00	5.13E-02	EIF2S1,HSPA2,IFNG,IL18,MAP2K4,MSR1
163	Semaphorin Signaling in Neurons	1.42E+00	6.67E-02	CDC42,CDK5,FES,PAK1
164	Endometrial Cancer Signaling	1.42E+00	6.67E-02	AKT2,ERBB2,MYC,RAP1B
165	Ceramide Signaling	1.41E+00	5.68E-02	AKT2,BCL2,MAP2K4,RAP1B,S1PR1
166	IL-2 Signaling	1.40E+00	6.56E-02	AKT2,JAK3,LCK,RAP1B
167	Factors Promoting Cardiogenesis in Vertebrates	1.39E+00	4.67E-02	MAP2K4,MYC,PLCB1,PRKCQ,SMAD5,SMAD9,WNT5B
168	Crosstalk between Dendritic Cells and Natural Killer Cells	1.39E+00	5.62E-02	ACTA2,IFNG,IL18,LTB,LTBR
169	Cholecystokinin/Gastrin-mediated Signaling	1.39E+00	5.04E-02	CDC42,IL18,MAP2K4,PLCB1,PRKCQ,RAP1B
170	Formaldehyde Oxidation II (Glutathione-dependent)	1.38E+00	5.00E-01	ADH5
171	Actin Cytoskeleton Signaling	1.37E+00	4.13E-02	ACTA2,BAIAP2,CDC42,FGF13,PAK1,PIP5K1A,RAP1B,RDX,TMSB4Y
172	Mismatch Repair in Eukaryotes	1.36E+00	1.25E-01	PCNA,RFC4
173	IL-17A Signaling in Airway Cells	1.34E+00	6.25E-02	AKT2,IL17A,JAK3,MAP2K4

174	Endothelin-1 Signaling	1.33E+00	4.26E-02	BRAF,MYC,PLA2G4A,PLA2G7,PLCB1,PLD3,PRKCQ,RAP1B
175	CCR3 Signaling in Eosinophils	1.32E+00	4.84E-02	GNG5,PAK1,PLA2G4A,PLCB1,PRKCQ,RAP1B
176	Regulation of Actin-based Motility by Rho	1.31E+00	5.32E-02	ACTA2,BAIAP2,CDC42,PAK1,PIP5K1A
177	CCR5 Signaling in Macrophages	1.31E+00	5.32E-02	CACNA1C,GNG5,MAP2K4,PRKCQ,TRGV9
178	ILK Signaling	1.31E+00	4.21E-02	ACTA2,AKT2,CDC42,ITGB6,MAP2K4,MYC,TMSB4Y,VEGFA
179	Histamine Degradation	1.31E+00	1.18E-01	ALDH3B1,HNMT
180	IL-6 Signaling	1.31E+00	4.80E-02	AKT2,IL18,IL1R1,MAP2K4,RAP1B,VEGFA
	Data2			
**S.No.**	**Ingenuity Canonical Pathways**	**-log(p-value)**	**Ratio**	**Molecules**
1	Atherosclerosis Signaling	6.43E+00	8.66E-02	ALOX15,CCL2,CCR3,CLU,COL10A1,CXCL8,CXCR4,PLAAT1,SELP,SERPINA1,TNFRSF12A
2	Granulocyte Adhesion and Diapedesis	4.95E+00	6.11E-02	CCL2,CCL23,CCL4,CLDN5,CXCL5,CXCL8,CXCR4,ITGB3,PF4,PPBP,SELP
3	Agranulocyte Adhesion and Diapedesis	4.67E+00	5.70E-02	CCL2,CCL23,CCL4,CLDN5,CXCL5,CXCL8,CXCR4,MYL9,PF4,PPBP,SELP
4	Coagulation System	3.11E+00	1.14E-01	F13A1,PROS1,SERPINA1,VWF
5	Breast Cancer Regulation by Stathmin1	3.10E+00	2.88E-02	ADGRE1,ADGRG3,ADORA3,C3AR1,CCR3,CNR2,CXCR6,GLP2R,GPR141,GPR34,GPR87,HCRTR1,HTR1B,P2RY14,P2RY2,PRKAR2B,PTGDR2
6	Cellular Effects of Sildenafil (Viagra)	3.00E+00	5.34E-02	CACNG6,GUCY1A1,MYL9,PDE2A,PDE5A,PRKAR2B,SLC4A10
7	Role of IL-17F in Allergic Inflammatory Airway Diseases	2.81E+00	9.52E-02	CCL2,CCL4,CXCL5,CXCL8
8	Relaxin Signaling	2.66E+00	4.67E-02	GNA12,GNAZ,GUCY1A1,NFKBID,PDE2A,PDE5A,PRKAR2B
9	Phagosome Formation	2.41E+00	4.80E-02	C3AR1,FCGR1A,FCGR1B,IGHG1,RHOBTB1,TLR5
10	Role of IL-17A in Arthritis	2.41E+00	7.41E-02	CCL2,CXCL5,CXCL8,NFKBID
11	Th2 Pathway	2.23E+00	4.41E-02	CCR3,CXCR4,CXCR6,JAK3,PTGDR2,SOCS3
12	γ-glutamyl Cycle	2.14E+00	1.82E-01	GGT5,OPLAH
13	IL-17A Signaling in Fibroblasts	2.08E+00	8.57E-02	CCL2,CXCL5,NFKBID
14	Role of IL-17A in Psoriasis	2.00E+00	1.54E-01	CXCL5,CXCL8
15	Gustation Pathway	1.98E+00	3.90E-02	CACNG6,P2RY14,P2RY2,PDE2A,PDE5A,PRKAR2B
16	Caveolar-mediated Endocytosis Signaling	1.95E+00	5.48E-02	FLNA,ITGA2B,ITGAD,ITGB3
17	Leukotriene Biosynthesis	1.93E+00	1.43E-01	GGT5,LTC4S
18	Sorbitol Degradation I	1.92E+00	1.00E+00	SORD
19	Histamine Biosynthesis	1.92E+00	1.00E+00	HDC
20	Calcium Signaling	1.92E+00	3.40E-02	CACNG6,CAMKK1,CHRFAM7A,CHRNA7,MYL9,PRKAR2B,TPM1
21	TREM1 Signaling	1.91E+00	5.33E-02	CCL2,CXCL8,TLR5,TREM1
22	Intrinsic Prothrombin Activation Pathway	1.87E+00	7.14E-02	COL10A1,F13A1,PROS1
23	GP6 Signaling Pathway	1.85E+00	4.20E-02	COL10A1,COL20A1,GP6,ITGA2B,ITGB3
24	Role of Hypercytokinemia/hyperchemokinemia in the Pathogenesis of Influenza	1.84E+00	6.98E-02	CCL2,CCL4,CXCL8
25	CXCR4 Signaling	1.82E+00	3.59E-02	CXCR4,EGR1,GNA12,GNAZ,MYL9,RHOBTB1
26	Extrinsic Prothrombin Activation Pathway	1.82E+00	1.25E-01	F13A1,PROS1
27	Chemokine Signaling	1.81E+00	5.00E-02	CCL2,CCL4,CCR3,CXCR4
28	G-Protein Coupled Receptor Signaling	1.78E+00	2.94E-02	ADORA3,CNR2,HTR1B,NFKBID,P2RY14,PDE2A,PDE5A,PRKAR2B
29	Th1 and Th2 Activation Pathway	1.77E+00	3.51E-02	CCR3,CXCR4,CXCR6,JAK3,PTGDR2,SOCS3
30	Gαi Signaling	1.77E+00	4.00E-02	ADORA3,CNR2,HTR1B,P2RY14,PRKAR2B
31	Differential Regulation of Cytokine Production in Macrophages and T Helper Cells by IL-17A and IL-17F	1.72E+00	1.11E-01	CCL2,CCL4
32	cAMP-mediated signaling	1.70E+00	3.07E-02	ADORA3,CNR2,HTR1B,P2RY14,PDE2A,PDE5A,PRKAR2B

33	Dendritic Cell Maturation	1.64E+00	3.28E-02	COL10A1,FCGR1A,FCGR1B,IGHG1,IL23A,NFKBID
34	IL-1 Signaling	1.63E+00	4.40E-02	GNA12,GNAZ,NFKBID,PRKAR2B
35	Th17 Activation Pathway	1.63E+00	4.40E-02	DEFB131A/DEFB131B,IL23A,JAK3,SOCS3
36	Androgen Signaling	1.63E+00	3.68E-02	CACNG6,GNA12,GNAZ,PRKAR2B,TGFB1I1
37	Sertoli Cell-Sertoli Cell Junction Signaling	1.62E+00	3.24E-02	CLDN5,EPN2,GUCY1A1,NECTIN1,PRKAR2B,SPTB
38	Hepatic Fibrosis / Hepatic Stellate Cell Activation	1.61E+00	3.23E-02	CCL2,COL10A1,COL20A1,CXCL8,FGFR2,MYL9
39	Phototransduction Pathway	1.60E+00	5.66E-02	GUCY1A1,PRKAR2B,SAG
40	Production of Nitric Oxide and Reactive Oxygen Species in Macrophages	1.59E+00	3.19E-02	CAT,CLU,JAK3,NFKBID,RHOBTB1,SERPINA1
41	Communication between Innate and Adaptive Immune Cells	1.55E+00	4.17E-02	CCL4,CXCL8,IGHG1,TLR5
42	Differential Regulation of Cytokine Production in Intestinal Epithelial Cells by IL-17A and IL-17F	1.52E+00	8.70E-02	CCL2,CCL4
43	Nitric Oxide Signaling in the Cardiovascular System	1.51E+00	4.04E-02	GUCY1A1,PDE2A,PDE5A,PRKAR2B
44	Role of Macrophages, Fibroblasts and Endothelial Cells in Rheumatoid Arthritis	1.47E+00	2.56E-02	CCL2,CEBPE,CXCL8,FCGR1A,IGHG1,NFKBID,SOCS3,TLR5
45	Coronavirus Pathogenesis Pathway	1.47E+00	3.33E-02	CCL2,CXCL8,NFKBID,RPS4Y1,RPS4Y2
46	Biotin-carboxyl Carrier Protein Assembly	1.45E+00	3.33E-01	ACACB
47	IL-17A Signaling in Airway Cells	1.39E+00	4.69E-02	CXCL5,JAK3,NFKBID
48	PXR/RXR Activation	1.37E+00	4.62E-02	ABCC3,CYP2B6,PRKAR2B
49	Integrin Signaling	1.37E+00	2.82E-02	CTTN,ITGA2B,ITGAD,ITGB3,MYL9,RHOBTB1
50	Eicosanoid Signaling	1.35E+00	4.55E-02	ALOX15,LTC4S,PLAAT1
