# Supplementary material for: Association between Arsenic Level, Gene Expression in Asian Population, and In Vitro Carcinogenic Bladder Tumor
Source: Oxid Med Cell Longev. 2022 Jan 7;2022:3459855. doi: 10.1155/2022/3459855 (PMC8760535; doi:10.1155/2022/3459855)
Supplement: Supplementary 7 — Table S6: significant genes in Data1 (low and high concentrations) and Data2 (low, medium, and high concentrations) and the overlap between two (highlighted in green). [file 3459855.f7.pdf]

Table S6. Significant genes in Data1 (low, high concentrations) and Data2(low, medium, high concentrations), and the overlap between two (highlighted in green).

Data1

S.No.	Symbol	Name	t.stat	p.value	X.log10.p.	FDR	entrezgene	AffyID	HGNC
1	ZNF799	zinc finger protein 799	-3.9684	0.00048108	3.3178	0.76894	90576	90576_at	HGNC:28071
2	ZNF141	zinc finger protein 141	-3.9182	0.00054926	3.2602	0.76894	7700	7700_at	HGNC:12926
3	EEF2K	eukaryotic elongation factor 2 kinase	-3.8235	0.00070466	3.152	0.76894	29904	29904_at	HGNC:24615
4	ZBTB25	zinc finger and BTB domain containing 25	-3.6507	0.0011065	2.9561	0.76894	7597	7597_at	HGNC:13112
5	ZNF440	zinc finger protein 440	-3.6391	0.0011403	2.943	0.76894	126070	126070_at	HGNC:20874
6	MT1X	metallothionein 1X	3.5451	0.0014544	2.8373	0.76894	4501	4501_at	HGNC:7405
7	LMBR1	limb development membrane protein 1	-3.5332	0.0014997	2.824	0.76894	64327	64327_at	HGNC:13243
8	LRIG1	leucine rich repeats and immunoglobulin like domains 1	-3.52	0.0015514	2.8093	0.76894	26018	26018_at	HGNC:17360
9	FCER1G	Fc fragment of IgE receptor Ig	3.5142	0.001575	2.8027	0.76894	2207	2207_at	HGNC:3611
10	ZNF649	zinc finger protein 649	-3.4872	0.0016882	2.7726	0.76894	65251	65251_at	HGNC:25741
11	SPIC	Spi-C transcription factor	3.4863	0.001692	2.7716	0.76894	121599	121599_at	HGNC:29549
12	HEATR5B	HEAT repeat containing 5B	-3.3813	0.0022132	2.655	0.92199	54497	54497_at	HGNC:29273
13	TUBA1B	tubulin alpha 1b	3.3314	0.0025127	2.5999	0.92607	10376	10376_at	HGNC:18809
14	CCDC144NL	CCDC144A N-terminal pseudogene	-3.3189	0.0025935	2.5861	0.92607	339184	339184_at	HGNC:33735
15	BACE2	beta-secretase 2	-3.221	0.0033204	2.4788	0.94807	25825	25825_at	HGNC:934
16	ZNF43	zinc finger protein 43	-3.1478	0.003987	2.3994	0.94807	7594	7594_at	HGNC:13109
17	TRAF5	TNF receptor associated factor 5	-3.0832	0.0046806	2.3297	0.94807	7188	7188_at	HGNC:12035
18	GUSBP5	GUSB pseudogene 5	-3.067	0.0048724	2.3123	0.94807	441046	441046_at	HGNC:42319
19	NIPSNAP3B	nipsnap homolog 3B	-3.0639	0.0049095	2.309	0.94807	55335	55335_at	HGNC:23641
20	LY96	lymphocyte antigen 96	3.0103	0.0056027	2.2516	0.94807	23643	23643_at	HGNC:17156
21	NUP88	nucleoporin 88	-2.9758	0.0060961	2.215	0.94807	4927	4927_at	HGNC:8067
22	AP3S1	adaptor related protein complex 3 subunit sigma 1	2.9652	0.0062558	2.2037	0.94807	1176	1176_at	HGNC:2013
23	PMS2P5	PMS1 homolog 2, mismatch repair system component pseudogene 5	-2.9592	0.0063482	2.1973	0.94807	5383	5383_at	HGNC:9130
24	ZFP30	ZFP30 zinc finger protein	-2.9573	0.0063777	2.1953	0.94807	22835	22835_at	HGNC:29555
25	TIMM29	translocase of inner mitochondrial membrane 29	-2.957	0.0063832	2.195	0.94807	90580	90580_at	HGNC:25152
26	ZNF780B	zinc finger protein 780B	-2.9456	0.0065632	2.1829	0.94807	163131	163131_at	HGNC:33109
27	ACSS1	acyl-CoA synthetase short chain family member 1	-2.9284	0.0068436	2.1647	0.94807	84532	84532_at	HGNC:16091
28	MT1H	metallothionein 1H	2.917	0.0070358	2.1527	0.94807	4496	4496_at	HGNC:7400
29	ELP1	elongator complex protein 1	-2.8896	0.0075183	2.1239	0.94807	8518	8518_at	HGNC:5959
30	DDHD2	DDHD domain containing 2	-2.8841	0.0076179	2.1182	0.94807	23259	23259_at	HGNC:29106
31	CNR2	cannabinoid receptor 2	-2.8808	0.0076788	2.1147	0.94807	1269	1269_at	HGNC:2160
32	PEX1	peroxisomal biogenesis factor 1	-2.8364	0.0085455	2.0683	0.94807	5189	5189_at	HGNC:8850
33	TAS2R20	taste 2 receptor member 20	-2.83	0.0086796	2.0615	0.94807	259295	259295_at	HGNC:19109
34	SLC31A2	solute carrier family 31 member 2	2.8291	0.0086972	2.0606	0.94807	1318	1318_at	HGNC:11017
35	RTN4IP1	reticulon 4 interacting protein 1	-2.8143	0.0090109	2.0452	0.94807	84816	84816_at	HGNC:18647
36	CISH	cytokine inducible SH2 containing protein	2.8109	0.0090858	2.0416	0.94807	1154	1154_at	HGNC:1984
37	PPP1R2	protein phosphatase 1 regulatory inhibitor subunit 2	-2.81	0.0091037	2.0408	0.94807	5504	5504_at	HGNC:9288
38	SMC6	structural maintenance of chromosomes 6	-2.8053	0.0092083	2.0358	0.94807	79677	79677_at	HGNC:20466

39	BTN2A1	butyrophilin subfamily 2 member A1	-2.7887	0.00958	2.0186	0.94807	11120	11120_at	HGNC:1136
40	ZNF841	zinc finger protein 841	-2.7666	0.010098	1.9958	0.94807	284371	284371_at	HGNC:27611
41	SNX25	sorting nexin 25	-2.7633	0.010176	1.9924	0.94807	83891	83891_at	HGNC:21883
42	ALCAM	activated leukocyte cell adhesion molecule	-2.7529	0.010431	1.9817	0.94807	214	214_at	HGNC:400
43	AKTIP	AKT interacting protein	-2.7436	0.010665	1.972	0.94807	64400	64400_at	HGNC:16710
44	ATP6V0D1	ATPase H+ transporting V0 subunit d1	2.7278	0.011071	1.9558	0.94807	9114	9114_at	HGNC:13724
45	LPAR4	lysophosphatidic acid receptor 4	2.7241	0.011168	1.952	0.94807	2846	2846_at	HGNC:4478
46	MBD4	methyl-CpG binding domain 4, DNA glycosylase	-2.7227	0.011205	1.9506	0.94807	8930	8930_at	HGNC:6919
47	CARD11	caspase recruitment domain family member 11	-2.7158	0.011389	1.9435	0.94807	84433	84433_at	HGNC:16393
48	ZNF138	zinc finger protein 138	-2.715	0.011412	1.9426	0.94807	7697	7697_at	HGNC:12922
49	POLI	DNA polymerase iota	-2.6747	0.012545	1.9015	0.94807	11201	11201_at	HGNC:9182
50	MIR600HG	MIR600 host gene	-2.6734	0.012585	1.9001	0.94807	81571	81571_at	HGNC:23642
51	ADARB1	adenosine deaminase RNA specific B1	-2.6697	0.012695	1.8964	0.94807	104	104_at	HGNC:226
52	ERCC6L2	ERCC excision repair 6 like 2	-2.6692	0.012709	1.8959	0.94807	375748	375748_at	HGNC:26922
53	METTL18	methyltransferase like 18	-2.6675	0.012759	1.8942	0.94807	92342	92342_at	HGNC:28793
54	HIGD2A	HIG1 hypoxia inducible domain family member 2A	2.6597	0.012995	1.8862	0.94807	192286	192286_at	HGNC:28311
55	ZNF844	zinc finger protein 844	-2.6556	0.013121	1.882	0.94807	284391	284391_at	HGNC:25932
56	TTC39B	tetratricopeptide repeat domain 39B	-2.6541	0.013166	1.8805	0.94807	158219	158219_at	HGNC:23704
57	NEMP2	nuclear envelope integral membrane protein 2	-2.6513	0.013254	1.8777	0.94807	100131211	100131211_at	HGNC:33700
58	DDX55	DEAD-box helicase 55	-2.6444	0.013467	1.8707	0.94807	57696	57696_at	HGNC:20085
59	SESN1	sestrin 1	-2.6368	0.013709	1.863	0.94807	27244	27244_at	HGNC:21595
60	ZBTB16	zinc finger and BTB domain containing 16	-2.6296	0.013941	1.8557	0.94807	7704	7704_at	HGNC:12930
61	NUP205	nucleoporin 205	-2.6267	0.014034	1.8528	0.94807	23165	23165_at	HGNC:18658
62	VPS36	vacuolar protein sorting 36 homolog	-2.6195	0.014272	1.8455	0.94807	51028	51028_at	HGNC:20312
63	SERPING1	serpin family G member 1	2.6168	0.014363	1.8428	0.94807	710	710_at	HGNC:1228
64	ZNF506	zinc finger protein 506	-2.605	0.01476	1.8309	0.94807	440515	440515_at	HGNC:23780
65	TMEM156	transmembrane protein 156	-2.591	0.015248	1.8168	0.94807	80008	80008_at	HGNC:26260
66	SFMBT1	Scm like with four mbt domains 1	-2.5897	0.015294	1.8155	0.94807	51460	51460_at	HGNC:20255
67	KRTCAP2	keratinocyte associated protein 2	2.5857	0.015436	1.8115	0.94807	200185	200185_at	HGNC:28942
68	CHMP2A	charged multivesicular body protein 2A	2.5793	0.015665	1.8051	0.94807	27243	27243_at	HGNC:30216
69	PSENEN	presenilin enhancer, gamma-secretase subunit	2.5734	0.01588	1.7992	0.94807	55851	55851_at	HGNC:30100
70	TP53INP1	tumor protein p53 inducible nuclear protein 1	-2.5588	0.016426	1.7845	0.94807	94241	94241_at	HGNC:18022
71	DOP1B	DOP1 leucine zipper like protein B	-2.5566	0.016509	1.7823	0.94807	9980	9980_at	HGNC:1291
72	SEMA4A	semaphorin 4A	2.5521	0.01668	1.7778	0.94807	64218	64218_at	HGNC:10729
73	DLST	dihydrolipoamide S-succinyltransferase	-2.5394	0.017176	1.7651	0.94807	1743	1743_at	HGNC:2911
74	XCL1	X-C motif chemokine ligand 1	2.5393	0.017179	1.765	0.94807	6375	6375_at	HGNC:10645
75	MT2A	metallothionein 2A	2.539	0.017188	1.7648	0.94807	4502	4502_at	HGNC:7406
76	SPEF2	sperm flagellar 2	-2.5359	0.017311	1.7617	0.94807	79925	79925_at	HGNC:26293
77	PATJ	PATJ crumbs cell polarity complex component	-2.5309	0.017514	1.7566	0.94807	10207	10207_at	HGNC:28881
78	CPSF1	cleavage and polyadenylation specific factor 1	-2.5302	0.017541	1.756	0.94807	29894	29894_at	HGNC:2324
79	CENPC	centromere protein C	-2.528	0.01763	1.7537	0.94807	1060	1060_at	HGNC:1854
80	ZNF426	zinc finger protein 426	-2.5239	0.017796	1.7497	0.94807	79088	79088_at	HGNC:20725
81	MOB4	MOB family member 4, phocein	-2.5139	0.018207	1.7398	0.94807	25843	25843_at	HGNC:17261
82	RBM4B	RNA binding motif protein 4B	-2.5114	0.01831	1.7373	0.94807	83759	83759_at	HGNC:28842
83	TMC6	transmembrane channel like 6	-2.5092	0.018402	1.7351	0.94807	11322	11322_at	HGNC:18021
84	CBR4	carbonyl reductase 4	-2.504	0.018625	1.7299	0.94807	84869	84869_at	HGNC:25891
85	HIGD1A	HIG1 hypoxia inducible domain family member 1A	2.5017	0.018723	1.7276	0.94807	25994	25994_at	HGNC:29527

86	TBC1D10C	TBC1 domain family member 10C	-2.4879	0.019318	1.714	0.94807	374403	374403_at	HGNC:24702
87	ZNF33A	zinc finger protein 33A	-2.4801	0.019666	1.7063	0.94807	7581	7581_at	HGNC:13096
88	KCNQ5	potassium voltage-gated channel subfamily Q member 5	-2.4756	0.019867	1.7019	0.94807	56479	56479_at	HGNC:6299
89	COBLL1	cordon-bleu WH2 repeat protein like 1	-2.4692	0.020159	1.6955	0.94807	22837	22837_at	HGNC:23571
90	DEF8	differentially expressed in FDCP 8 homolog	-2.4635	0.020421	1.6899	0.94807	54849	54849_at	HGNC:25969
91	PRMT7	protein arginine methyltransferase 7	-2.4614	0.020517	1.6879	0.94807	54496	54496_at	HGNC:25557
92	RPL31	ribosomal protein L31	-2.4581	0.020672	1.6846	0.94807	6160	6160_at	HGNC:10334
93	UFSP2	UFM1 specific peptidase 2	-2.4471	0.021189	1.6739	0.94807	55325	55325_at	HGNC:25640
94	WDSUB1	WD repeat, sterile alpha motif and U-box domain containing 1	-2.4389	0.021588	1.6658	0.94807	151525	151525_at	HGNC:26697
95	DIP2A	disco interacting protein 2 homolog A	-2.4342	0.021815	1.6612	0.94807	23181	23181_at	HGNC:17217
96	DZIP3	DAZ interacting zinc finger protein 3	-2.4279	0.02213	1.655	0.94807	9666	9666_at	HGNC:30938
97	TIMP1	TIMP metallopeptidase inhibitor 1	2.4241	0.022321	1.6513	0.94807	7076	7076_at	HGNC:11820
98	ZBED5	zinc finger BED-type containing 5	-2.4226	0.022396	1.6498	0.94807	58486	58486_at	HGNC:30803
99	ARHGEF18	Rho/Rac guanine nucleotide exchange factor 18	-2.414	0.022831	1.6415	0.94807	23370	23370_at	HGNC:17090
100	RPL32	ribosomal protein L32	-2.4128	0.022895	1.6403	0.94807	6161	6161_at	HGNC:10336
101	SLC38A11	solute carrier family 38 member 11	-2.4122	0.022926	1.6397	0.94807	151258	151258_at	HGNC:26836
102	CRYGS	crystallin gamma S	-2.4038	0.023359	1.6315	0.94807	1427	1427_at	HGNC:2417
103	AIM2	absent in melanoma 2	-2.4026	0.023424	1.6303	0.94807	9447	9447_at	HGNC:357
104	EFCAB7	EF-hand calcium binding domain 7	-2.388	0.024199	1.6162	0.94807	84455	84455_at	HGNC:29379
105	RRP15	ribosomal RNA processing 15 homolog	-2.3875	0.024229	1.6157	0.94807	51018	51018_at	HGNC:24255
106	SNORA44	small nucleolar RNA, H/ACA box 44	-2.3825	0.024499	1.6109	0.94807	677825	677825_at	HGNC:32637
107	ZNF510	zinc finger protein 510	-2.3822	0.024513	1.6106	0.94807	22869	22869_at	HGNC:29161
108	PAPSS2	3'-phosphoadenosine 5'-phosphosulfate synthase 2	2.3779	0.02475	1.6064	0.94807	9060	9060_at	HGNC:8604
109	SNORA14A	small nucleolar RNA, H/ACA box 14A	-2.3756	0.02488	1.6042	0.94807	677801	677801_at	HGNC:32602
110	LOC100128398	uncharacterized LOC100128398	-2.3741	0.024961	1.6027	0.94807	100128398	100128398_at	
111	YBEY	ybeY metalloendoribonuclease	-2.3683	0.025284	1.5972	0.94807	54059	54059_at	HGNC:1299
112	MFSD1	major facilitator superfamily domain containing 1	2.3654	0.02545	1.5943	0.94807	64747	64747_at	HGNC:25874
113	RPL8	ribosomal protein L8	-2.3643	0.02551	1.5933	0.94807	6132	6132_at	HGNC:10368
114	GCNT2	glucosaminyl (N-acetyl) transferase 2 (I blood group)	-2.3605	0.025728	1.5896	0.94807	2651	2651_at	HGNC:4204
115	JUP	junction plakoglobin	2.3605	0.025729	1.5896	0.94807	3728	3728_at	HGNC:6207
116	IFT80	intraflagellar transport 80	-2.3572	0.025921	1.5864	0.94807	57560	57560_at	HGNC:29262
117	PIK3C2B	phosphatidylinositol-4-phosphate 3-kinase catalytic subunit type 2 beta	-2.3569	0.025934	1.5861	0.94807	5287	5287_at	HGNC:8972
118	DIS3L	DIS3 like exosome 3'-5' exoribonuclease	-2.3528	0.026172	1.5822	0.94807	115752	115752_at	HGNC:28698
119	S100A4	S100 calcium binding protein A4	2.3512	0.026264	1.5806	0.94807	6275	6275_at	HGNC:10494
120	FAM149B1	family with sequence similarity 149 member B1	-2.3508	0.026288	1.5802	0.94807	317662	317662_at	HGNC:29162
121	ZFP14	ZFP14 zinc finger protein	-2.3467	0.026526	1.5763	0.94807	57677	57677_at	HGNC:29312
122	SLC15A4	solute carrier family 15 member 4	2.344	0.026688	1.5737	0.94807	121260	121260_at	HGNC:23090
123	SMIM3	small integral membrane protein 3	2.3391	0.026977	1.569	0.94807	85027	85027_at	HGNC:30248
124	MRPS17	mitochondrial ribosomal protein S17	2.3247	0.027851	1.5552	0.94807	51373	51373_at	HGNC:14047
125	TNFRSF13B	TNF receptor superfamily member 13B	-2.3236	0.027919	1.5541	0.94807	23495	23495_at	HGNC:18153
126	LY6E	lymphocyte antigen 6 family member E	2.3209	0.028088	1.5515	0.94807	4061	4061_at	HGNC:6727
127	ENPP3	ectonucleotide pyrophosphatase/phosphodiesterase 3	-2.3185	0.028236	1.5492	0.94807	5169	5169_at	HGNC:3358
128	ZNF224	zinc finger protein 224	-2.317	0.028328	1.5478	0.94807	7767	7767_at	HGNC:13017
129	ZNF302	zinc finger protein 302	-2.3162	0.028377	1.547	0.94807	55900	55900_at	HGNC:13848
130	LIPA	lipase A, lysosomal acid type	2.3149	0.028463	1.5457	0.94807	3988	3988_at	HGNC:6617

131	NCAPD3	non-SMC condensin II complex subunit D3	-2.3116	0.028666	1.5426	0.94807	23310	23310_at	HGNC:28952	
132	ZDHHC23	zinc finger DHHC-type palmitoyltransferase 23	-2.3005	0.029375	1.532	0.94807	254887	254887_at	HGNC:28654	
133	NDUFAF6	NADH:ubiquinone oxidoreductase complex assembly factor 6	-2.3001	0.029404	1.5316	0.94807	137682	137682_at	HGNC:28625	
134	SNORD29	small nucleolar RNA, C/D box 29	-2.2957	0.029688	1.5274	0.94807	9297	9297_at	HGNC:10151	
135	GORAB	golgin, RAB6 interacting	-2.2944	0.029771	1.5262	0.94807	92344	92344_at	HGNC:25676	
136	KAT2A	lysine acetyltransferase 2A	-2.294	0.029795	1.5259	0.94807	2648	2648_at	HGNC:4201	
137	ZNF33B	zinc finger protein 33B	-2.2933	0.029843	1.5252	0.94807	7582	7582_at	HGNC:13097	
138	ATP10D	ATPase phospholipid transporting 10D (putative)	-2.2907	0.030016	1.5226	0.94807	57205	57205_at	HGNC:13549	
139	TAS2R19	taste 2 receptor member 19	-2.2895	0.030095	1.5215	0.94807	259294	259294_at	HGNC:19108	
140	CLUAP1	clusterin associated protein 1	-2.288	0.030191	1.5201	0.94807	23059	23059_at	HGNC:19009	
141	FLNB	filamin B	-2.2856	0.030354	1.5178	0.94807	2317	2317_at	HGNC:3755	
142	GGTA1P	glycoprotein alpha-galactosyltransferase 1, pseudogene	2.2814	0.030628	1.5139	0.94807	2681	2681_at	HGNC:4253	
143	SLC4A10	solute carrier family 4 member 10	-2.2791	0.030785	1.5117	0.94807	57282	57282_at	HGNC:13811	
144	ATG14	autophagy related 14	-2.2766	0.030957	1.5092	0.94807	22863	22863_at	HGNC:19962	
145	HNRNPH1	heterogeneous nuclear ribonucleoprotein H1	-2.2754	0.031033	1.5082	0.94807	3187	3187_at	HGNC:5041	
146	CTSS	cathepsin S	2.2753	0.031041	1.5081	0.94807	1520	1520_at	HGNC:2545	
147	PSIP1	PC4 and SFRS1 interacting protein 1	-2.2728	0.031214	1.5057	0.94807	11168	11168_at	HGNC:9527	
148	NR2C1	nuclear receptor subfamily 2 group C member 1	-2.2726	0.031228	1.5055	0.94807	7181	7181_at	HGNC:7971	
149	TTC13	tetratricopeptide repeat domain 13	-2.2684	0.031512	1.5015	0.94807	79573	79573_at	HGNC:26204	
150	DTX1	deltex E3 ubiquitin ligase 1	-2.2681	0.031534	1.5012	0.94807	1840	1840_at	HGNC:3060	
151	NT5E	5'-nucleotidase ecto	-2.2665	0.031647	1.4997	0.94807	4907	4907_at	HGNC:8021	
152	LY6G5C	lymphocyte antigen 6 family member G5C	2.2626	0.031912	1.496	0.94807	80741	80741_at	HGNC:13932	
153	ZNF767P	zinc finger family member 767, pseudogene	-2.2578	0.03225	1.4915	0.94807	79970	79970_at	HGNC:21884	
154	SNHG32	small nucleolar RNA host gene 32	-2.2573	0.032284	1.491	0.94807	50854	50854_at	HGNC:19078	
155	ZNF160	zinc finger protein 160	-2.2543	0.032499	1.4881	0.94807	90338	90338_at	HGNC:12948	
156	INTS4	integrator complex subunit 4	-2.2535	0.032551	1.4874	0.94807	92105	92105_at	HGNC:25048	
157	AQP10	aquaporin 10	2.2533	0.03257	1.4872	0.94807	89872	89872_at	HGNC:16029	
158	CERK	ceramide kinase	-2.2514	0.032701	1.4854	0.94807	64781	64781_at	HGNC:19256	
159	HAPLN3	hyaluronan and proteoglycan link protein 3	-2.246	0.033089	1.4803	0.94807	145864	145864_at	HGNC:21446	
160	ACVR1C	activin A receptor type 1C	-2.2451	0.033156	1.4794	0.94807	130399	130399_at	HGNC:18123	
161	UPF3A	UPF3A regulator of nonsense mediated mRNA decay	-2.2426	0.033331	1.4772	0.94807	65110	65110_at	HGNC:20332	
162	CCDC14	coiled-coil domain containing 14	-2.2403	0.033504	1.4749	0.94807	64770	64770_at	HGNC:25766	
163	EMP3	epithelial membrane protein 3	2.2353	0.033868	1.4702	0.94807	2014	2014_at	HGNC:3335	
164	C11orf65	chromosome 11 open reading frame 65	-2.235	0.033887	1.47	0.94807	160140	160140_at	HGNC:28519	
165	RAB6B	RAB6B, member RAS oncogene family	2.2311	0.034174	1.4663	0.94807	51560	51560_at	HGNC:14902	
166	PDPR	pyruvate dehydrogenase phosphatase regulatory subunit	2.2302	0.034242	1.4654	0.94807	55066	55066_at	HGNC:30264	
167	ZNF678	zinc finger protein 678	-2.2297	0.034276	1.465	0.94807	339500	339500_at	HGNC:28652	
168	FAM169A	family with sequence similarity 169 member A	-2.2263	0.034531	1.4618	0.94807	26049	26049_at	HGNC:29138	
169	SGCE	sarcoglycan epsilon	-2.2236	0.034732	1.4593	0.94807	8910	8910_at	HGNC:10808	
170	PSPH	phosphoserine phosphatase	-2.2199	0.035013	1.4558	0.94807	5723	5723_at	HGNC:9577	
171	SUDS3	SDS3 homolog, SIN3A corepressor complex component	-2.2089	0.035857	1.4454	0.94807	64426	64426_at	HGNC:29545	
172	ANKLE2	ankyrin repeat and LEM domain containing 2	-2.2068	0.036013	1.4435	0.94807	23141	23141_at	HGNC:29101	
173	LGALS1	galectin 1	2.2046	0.036185	1.4415	0.94807	3956	3956_at	HGNC:6561	
174	MT1F	metallothionein 1F	2.2038	0.036251	1.4407	0.94807	4494	4494_at	HGNC:7398	
175	SMAD5	SMAD family member 5	-2.2036	0.036263	1.4405	0.94807	4090	4090_at	HGNC:6771	
176	ABCA5	ATP binding cassette subfamily A member 5	-2.2024	0.036356	1.4394	0.94807	23461	23461_at	HGNC:35	

177	ZNF808	zinc finger protein 808	-2.2023	0.036364	1.4393	0.94807	388558	388558_at	HGNC:33230
178	BTAF1	B-TFIID TATA-box binding protein associated factor 1	-2.2003	0.036521	1.4375	0.94807	9044	9044_at	HGNC:17307
179	PTGDS	prostaglandin D2 synthase	-2.1973	0.036756	1.4347	0.94807	5730	5730_at	HGNC:9592
180	CABP5	calcium binding protein 5	2.1969	0.036793	1.4342	0.94807	56344	56344_at	HGNC:13714
181	ZNF75A	zinc finger protein 75a	-2.1948	0.036961	1.4323	0.94807	7627	7627_at	HGNC:13146
182	RAD54B	RAD54 homolog B	-2.1945	0.036978	1.4321	0.94807	25788	25788_at	HGNC:17228
183	CCDC122	coiled-coil domain containing 122	2.1944	0.036989	1.4319	0.94807	160857	160857_at	HGNC:26478
184	ZNF286A	zinc finger protein 286A	-2.1851	0.037731	1.4233	0.94807	57335	57335_at	HGNC:13501
185	OSGEPL1	O-sialoglycoprotein endopeptidase like 1	-2.1847	0.037767	1.4229	0.94807	64172	64172_at	HGNC:23075
186	KLF13	Kruppel like factor 13	-2.1819	0.037991	1.4203	0.94807	51621	51621_at	HGNC:13672
187	KLHDC2	kelch domain containing 2	-2.1802	0.038132	1.4187	0.94807	23588	23588_at	HGNC:20231
188	ZNF121	zinc finger protein 121	-2.1795	0.038188	1.4181	0.94807	7675	7675_at	HGNC:12904
189	PLCG1	phospholipase C gamma 1	-2.1737	0.038669	1.4126	0.94807	5335	5335_at	HGNC:9065
190	IFI30	IFI30 lysosomal thiol reductase	2.1659	0.039315	1.4054	0.94807	10437	10437_at	HGNC:5398
191	GTPBP6	GTP binding protein 6 (putative)	-2.1628	0.039579	1.4025	0.94807	8225	8225_at	HGNC:30189
192	RAB31	RAB31, member RAS oncogene family	2.1602	0.039799	1.4001	0.94807	11031	11031_at	HGNC:9771
193	LMO7	LIM domain 7	-2.1594	0.039865	1.3994	0.94807	4008	4008_at	HGNC:6646
194	GRAMD1C	GRAM domain containing 1C	-2.1593	0.039876	1.3993	0.94807	54762	54762_at	HGNC:25252
195	TAS2R14	taste 2 receptor member 14	-2.1592	0.039879	1.3993	0.94807	50840	50840_at	HGNC:14920
196	TAS2R10	taste 2 receptor member 10	-2.1587	0.039921	1.3988	0.94807	50839	50839_at	HGNC:14918
197	ZNF234	zinc finger protein 234	-2.15	0.040668	1.3908	0.94807	10780	10780_at	HGNC:13027
198	PPDPF	pancreatic progenitor cell differentiation and proliferation factor	2.1459	0.041027	1.3869	0.94807	79144	79144_at	HGNC:16142
199	CCR3	C-C motif chemokine receptor 3	-2.1419	0.041374	1.3833	0.94807	1232	1232_at	HGNC:1604
200	EBPL	EBP like	-2.1336	0.042113	1.3756	0.94807	84650	84650_at	HGNC:18061
201	CXCL16	C-X-C motif chemokine ligand 16	2.13	0.042438	1.3722	0.94807	58191	58191_at	HGNC:16642
202	ATP6V0B	ATPase H+ transporting V0 subunit b	2.1288	0.042541	1.3712	0.94807	533	533_at	HGNC:861
203	HLA-F-AS1	HLA-F antisense RNA 1	-2.1279	0.042624	1.3703	0.94807	285830	285830_at	HGNC:26645
204	HIP1	huntingtin interacting protein 1	-2.1214	0.043213	1.3644	0.94807	3092	3092_at	HGNC:4913
205	ITPR1	inositol 1,4,5-trisphosphate receptor type 1	-2.1203	0.043318	1.3633	0.94807	3708	3708_at	HGNC:6180
206	BTN2A2	butyrophilin subfamily 2 member A2	-2.1199	0.043349	1.363	0.94807	10385	10385_at	HGNC:1137
207	MYADM	myeloid associated differentiation marker	2.1194	0.0434	1.3625	0.94807	91663	91663_at	HGNC:7544
208	GAA	glucosidase alpha, acid	2.119	0.043435	1.3622	0.94807	2548	2548_at	HGNC:4065
209	PHTF2	putative homeodomain transcription factor 2	-2.1187	0.043458	1.3619	0.94807	57157	57157_at	HGNC:13411
210	CD163	CD163 molecule	2.1146	0.043837	1.3582	0.94807	9332	9332_at	HGNC:1631
211	ABI3	ABI family member 3	2.1138	0.043915	1.3574	0.94807	51225	51225_at	HGNC:29859
212	PAN2	poly(A) specific ribonuclease subunit PAN2	-2.1125	0.044036	1.3562	0.94807	9924	9924_at	HGNC:20074
213	C1QB	complement C1q B chain	2.1104	0.044225	1.3543	0.94807	713	713_at	HGNC:1242
214	DGKD	diacylglycerol kinase delta	-2.1085	0.044407	1.3526	0.94807	8527	8527_at	HGNC:2851
215	C18orf54	chromosome 18 open reading frame 54	-2.1027	0.044957	1.3472	0.94807	162681	162681_at	HGNC:13796
216	DNAJC10	DnaJ heat shock protein family (Hsp40) member C10	-2.1011	0.045101	1.3458	0.94807	54431	54431_at	HGNC:24637
217	C9orf40	chromosome 9 open reading frame 40	-2.1008	0.04513	1.3455	0.94807	55071	55071_at	HGNC:23433
218	XCL2	X-C motif chemokine ligand 2	-2.1007	0.04514	1.3454	0.94807	6846	6846_at	HGNC:10646
219	PIGT	phosphatidylinositol glycan anchor biosynthesis class T	2.0918	0.045998	1.3373	0.94807	51604	51604_at	HGNC:14938
220	HERC2	HECT and RLD domain containing E3 ubiquitin protein ligase 2	-2.0857	0.046584	1.3318	0.94807	8924	8924_at	HGNC:4868
221	ATP6V1F	ATPase H+ transporting V1 subunit F	2.0852	0.046633	1.3313	0.94807	9296	9296_at	HGNC:16832

222	LIMS1	LIM zinc finger domain containing 1	2.0827	0.046882	1.329	0.94807	3987	3987_at	HGNC:6616
223	C12orf65	chromosome 12 open reading frame 65	-2.0815	0.046995	1.328	0.94807	91574	91574_at	HGNC:26784
224	ZFP28	ZFP28 zinc finger protein	-2.0782	0.047324	1.3249	0.94807	140612	140612_at	HGNC:17801
225	ADCY10P1	ADCY10 pseudogene 1	-2.0724	0.047899	1.3197	0.94807	221442	221442_at	HGNC:44143
226	HS3ST1	heparan sulfate-glucosamine 3-sulfotransferase 1	-2.0718	0.047966	1.3191	0.94807	9957	9957_at	HGNC:5194
227	NHEJ1	non-homologous end joining factor 1	-2.0677	0.048377	1.3154	0.94807	79840	79840_at	HGNC:25737
228	ZNF251	zinc finger protein 251	-2.0664	0.048504	1.3142	0.94807	90987	90987_at	HGNC:13045
229	IL4R	interleukin 4 receptor	-2.0659	0.048554	1.3138	0.94807	3566	3566_at	HGNC:6015
230	ZNF836	zinc finger protein 836	-2.0571	0.049456	1.3058	0.94807	162962	162962_at	HGNC:34333
231	CRIM1	cysteine rich transmembrane BMP regulator 1	-2.0559	0.04958	1.3047	0.94807	51232	51232_at	HGNC:2359
232	JUNB	JunB proto-oncogene, AP-1 transcription factor subunit	2.0559	0.049581	1.3047	0.94807	3726	3726_at	HGNC:6205
	Data2								
**S.No.**	**Symbol**	**LOCUSLINK_ID**	**f.value**	**p.value**	**X.log10.p.**	**FDR**	**Fisher.s.LSD**	**GB_ACC**	**Description**
1	PP12719	NA	20.962	1.83E-07	6.7374	0.00091518	High - Low; High - Medium; Medium - Low	XM_003403527	Homo sapiens ubiquitin specific peptidase 9, Y-linked (USP9Y), mRNA [NM_004654]
2	MALAT1	NA	18.85	6.17E-07	6.2094	0.0015433	Low - High; Medium - High; Low - Medium	NR_002819	Homo sapiens taxilin gamma 2, pseudogene (TXLNG2P), transcript variant 1, non-coding RNA [NR_045128]
3	MALAT1	NA	17.04	1.83E-06	5.7372	0.0030522	Low - High; Medium - High; Low - Medium	NR_002819	Homo sapiens taxilin gamma 2, pseudogene (TXLNG2P), transcript variant 2, non-coding RNA [NR_045129]
4	LOC100506930	NA	14.838	7.32E-06	5.1356	0.0081549	Low - High; Medium - High; Low - Medium	NR_038279	Homo sapiens X (inactive)-specific transcript (non-protein coding) (XIST), non-coding RNA [NR_001564]
5	PTGS2	5743	14.67	8.16E-06	5.0885	0.0081549	Low - High; Medium - High; Low - Medium	NM_000963	Homo sapiens eukaryotic translation initiation factor 1A, Y-linked (EIF1AY), mRNA [NM_004681]
6	SFN	2810	14.251	1.07E-05	4.9698	0.0089317	Low - High; High - Medium; Low - Medium	NM_006142	BROAD Institute lincRNA (XLOC_008323), lincRNA [TCONS_00017647]
7	XLOC_012515	NA	13.839	1.41E-05	4.8522	0.010038	High - Low; High - Medium; Medium - Low		Homo sapiens X (inactive)-specific transcript (non-protein coding) (XIST), non-coding RNA [NR_001564]

8	LOC100507254	NA		12.102	4.54E-05	4.3425	0.028398	Low - High; Medium - High; Medium - Low	NR_038981	Homo sapiens X (inactive)-specific transcript (non-protein coding) (XIST), non-coding RNA [NR_001564]
9	IL8		3576	11.438	7.22E-05	4.1417	0.040085	Low - High; Medium - High; Low - Medium		Homo sapiens X (inactive)-specific transcript (non-protein coding) (XIST), non-coding RNA [NR_001564]
10	EGR2		1959	11.049	9.50E-05	4.0225	0.047467	Low - High; Medium - High; Low - Medium	NM_000399	Homo sapiens lysine (K)-specific demethylase 5D (KDM5D), transcript variant 2, mRNA [NM_004653]
11	SHISA4		149345	10.643	0.00012688	3.8966	0.0536	Low - High; High - Medium; Low - Medium	NM_198149	Homo sapiens X (inactive)-specific transcript (non-protein coding) (XIST), non-coding RNA [NR_001564]
12	C9orf40		55071	10.623	0.00012867	3.8905	0.0536	High - Low; High - Medium; Medium - Low	NM_017998	Homo sapiens X (inactive)-specific transcript (non-protein coding) (XIST), non-coding RNA [NR_001564]
13	XLOC_000683	NA		9.7984	0.00023413	3.6306	0.087545	High - Low; High - Medium; Medium - Low		Homo sapiens ribosomal protein S4, Y-linked 1 (RPS4Y1), mRNA [NM_001008]
14	BMS1P5	NA		9.6385	0.00026334	3.5795	0.087545	Low - High; Medium - High; Low - Medium	BC065722	PREDICTED: Homo sapiens gamma-taxilin-like (LOC100509121), mRNA [XM_003120334]
15	PSPH		5723	9.5717	0.00027663	3.5581	0.087545	High - Low; Medium - High; Medium - Low	NM_004577	ubiquitously transcribed tetratricopeptide repeat gene, Y-linked [Source:HGNC Symbol;Acc:12638] [ENST00000382893]
16	DSP		1832	9.4438	0.00030408	3.517	0.087545	Low - High; Medium - High; Medium - Low	NM_004415	PREDICTED: Homo sapiens hypothetical protein LOC100653022 (LOC100653022), mRNA [XM_003403527]
17	XLOC_012515	NA		9.4428	0.0003043	3.5167	0.087545	High - Low; High - Medium; Medium - Low		Homo sapiens ribosomal protein S4, Y-linked 2 (RPS4Y2), mRNA [NM_001039567]
18	XLOC_l2_015178	NA		9.3824	0.00031823	3.4973	0.087545	Low - High; Medium - High; Medium - Low	CB121594	Homo sapiens X (inactive)-specific transcript (non-protein coding) (XIST), non-coding RNA [NR_001564]

19	G0S2		50486	9.2946	0.0003397	3.4689	0.087545	Low - High; Medium - High; Low - Medium	NM_015714	BROAD Institute lincRNA (XLOC_008015), lincRNA [TCONS_00017433]
20	IL8		3576	9.2535	0.00035025	3.4556	0.087545	Low - High; High - Medium; Low - Medium	NM_000584	Homo sapiens non-protein coding RNA 185 (NCRNA00185), non-coding RNA [NR_001544]
21	PHACTR1		221692	9.0622	0.00040403	3.3936	0.092589	Low - High; Medium - High; Low - Medium	AB051520	Homo sapiens ubiquitously transcribed tetratricopeptide repeat gene, Y-linked (UTY), transcript variant 3, mRNA [NM_007125]
22	LOC643072	NA		8.9788	0.00043009	3.3664	0.092589	High - Low; High - Medium; Medium - Low	XR_108434	Homo sapiens DEAD (Asp-Glu-Ala-Asp) box polypeptide 3, Y-linked (DDX3Y), transcript variant 1, mRNA [NM_001122665]
23	FOS		2353	8.9515	0.000439	3.3575	0.092589	Low - High; Medium - High; Low - Medium	NM_005252	Homo sapiens metastasis associated lung adenocarcinoma transcript 1 (non-protein coding) (MALAT1), non-coding RNA [NR_002819]
24	PIK3R6		146850	8.9349	0.00044452	3.3521	0.092589	High - Low; Medium - High; Medium - Low	NM_001010855	Homo sapiens eukaryotic translation initiation factor 1A, Y-linked (EIF1AY), mRNA [NM_004681]
25	UTY		7404	8.7449	0.0005129	3.29	0.10067	Low - High; Medium - High; Low - Medium	BC012581	Homo sapiens metastasis associated lung adenocarcinoma transcript 1 (non-protein coding) (MALAT1), non-coding RNA [NR_002819]
26	BAMBI		25805	8.6696	0.00054295	3.2652	0.10067	High - Low; Medium - High; Medium - Low	NM_012342	BROAD Institute lincRNA (XLOC_008276), lincRNA [TCONS_00017607]
27	DOCK9		23348	8.6678	0.00054372	3.2646	0.10067	Low - High; High - Medium; Low - Medium	AK090793	Homo sapiens uncharacterized LOC100506930 (LOC100506930), transcript variant 2, non-coding RNA [NR_038279]
28	LOC643802	NA		8.3896	0.0006717	3.1728	0.1191	High - Low; High - Medium; Medium - Low	BX537874	Homo sapiens protein kinase, Y-linked, pseudogene (PRKY), non-coding RNA [NR_028062]

29	LOC100507254	NA		8.3526	0.00069094	3.1606	0.1191	Low - High; Medium - High; Medium - Low	NR_038981	Homo sapiens taxilin gamma 2, pseudogene (TXLNG2P), transcript variant 1, non-coding RNA [NR_045128]
30	CRISPLD2		83716	8.2679	0.0007372	3.1324	0.12149	High - Low; High - Medium; Medium - Low	NM_031476	BROAD Institute lincRNA (XLOC_008276), lincRNA [TCONS_00017606]
31	RAP1GAP2		23108	8.2395	0.00075338	3.123	0.12149	High - Low; High - Medium; Medium - Low	NM_015085	PREDICTED: Homo sapiens hypothetical LOC100506003 (LOC100506003), miscRNA [XR_109785]
32	BTG2		7832	8.1647	0.00079789	3.0981	0.12387	Low - High; Medium - High; Low - Medium	NM_006763	BROAD Institute lincRNA (XLOC_008276), lincRNA [TCONS_00017607]
33	TCL1A		8115	8.1327	0.00081773	3.0874	0.12387	Low - High; Medium - High; Medium - Low	NM_021966	BROAD Institute lincRNA (XLOC_008185), lincRNA [TCONS_00017074]
34	XLOC_008276	NA		8.0773	0.00085332	3.0689	0.12461	Low - High; Medium - High; Low - Medium		Homo sapiens stratifin (SFN), mRNA [NM_006142]
35	TXLNG2P		246126	8.0035	0.00090325	3.0442	0.12461	Low - High; Medium - High; Low - Medium	NR_045128	Homo sapiens regulator of G-protein signaling 1 (RGS1), mRNA [NM_002922]
36	XLOC_008276	NA		7.9791	0.00092041	3.036	0.12461	Low - High; Medium - High; Low - Medium		Homo sapiens testis-specific transcript, Y-linked 15 (non-protein coding) (TTTY15), non-coding RNA [NR_001545]
37	DSP		1832	7.9765	0.0009223	3.0351	0.12461	Low - High; Medium - High; Medium - Low	NM_004415	Homo sapiens interleukin 8 (IL8), mRNA [NM_000584]
38	SEC14L3		266629	7.8772	0.00099589	3.0018	0.12727	Low - High; Medium - High; Low - Medium	AK131358	Homo sapiens early growth response 3 (EGR3), transcript variant 1, mRNA [NM_004430]
39	TXLNG2P		246126	7.8639	0.0010062	2.9973	0.12727	Low - High; Medium - High; Low - Medium	NR_045129	Homo sapiens prostaglandin-endoperoxide synthase 2 (prostaglandin G/H synthase and cyclooxygenase) (PTGS2), mRNA [NM_000963]
40	GADD45G		10912	7.8484	0.0010183	2.9921	0.12727	High - Low; High - Medium; Medium - Low	NM_006705	Homo sapiens chemokine (C-X-C motif) receptor 4 (CXCR4), transcript variant 1, mRNA [NM_001008540]

41	MXI1		4601	7.799	0.0010581	2.9755	0.12901	Low - High; Medium - High; Medium - Low	NM_130439	BROAD Institute lincRNA (XLOC_012515), lincRNA [TCONS_00025694]
42	CCDC144A		9720	7.7248	0.0011209	2.9504	0.13341	Low - High; Medium - High; Low - Medium	NM_014695	interleukin 8 [Source:HGNC Symbol;Acc:6025] [ENST00000401931]
43	TAGAP		117289	7.689	0.0011525	2.9384	0.13398	Low - High; Medium - High; Low - Medium	NM_138810	Homo sapiens early growth response 2 (EGR2), transcript variant 1, mRNA [NM_000399]
44	LOC100134317	NA		7.6375	0.0011996	2.9209	0.1363	Low - High; Medium - High; Low - Medium	NR_029389	Homo sapiens zinc finger protein, Y-linked (ZFY), transcript variant 1, mRNA [NM_003411]
45	PSPHP1	NA		7.5814	0.0012534	2.9019	0.13923	High - Low; Medium - High; Medium - Low	BC065228	Homo sapiens testis-specific transcript, Y-linked 15 (non-protein coding) (TTTY15), non-coding RNA [NR_001545]
46	MXD3		83463	7.4533	0.0013855	2.8584	0.14828	High - Low; High - Medium; Medium - Low	NM_001142935	PREDICTED: Homo sapiens hypothetical LOC643072 (LOC643072), miscRNA [XR_108434]
47	1-Mar		64757	7.4453	0.0013941	2.8557	0.14828	High - Low; High - Medium; Medium - Low	NM_022746	Homo sapiens DEAD (Asp-Glu-Ala-Asp) box polypeptide 3, Y-linked (DDX3Y), transcript variant 2, mRNA [NM_004660]
48	LPCAT2		54947	7.346	0.0015072	2.8218	0.15696	High - Low; High - Medium; Medium - Low	NM_017839	Homo sapiens FBJ murine osteosarcoma viral oncogene homolog B (FOSB), transcript variant 1, mRNA [NM_006732]
49	USP9Y		8287	7.3133	0.0015464	2.8107	0.15777	Low - High; Medium - High; Low - Medium	NM_004654	Homo sapiens amphiregulin (AREG), mRNA [NM_001657]
50	APBA2		321	7.1973	0.0016945	2.771	0.16942	Low - High; Medium - High; Low - Medium	AK124794	Homo sapiens small ILF3/NF90-associated RNA B2 (SNAR-B2), small nuclear RNA [NR_024230]
51	BMS1P1	NA		7.1416	0.0017707	2.7519	0.17356	Low - High; Medium - High; Low - Medium	NR_026566	Homo sapiens early growth response 1 (EGR1), mRNA [NM_001964]

52	STK17B		9262	7.0427	0.0019151	2.7178	0.1838	Low - High; Medium - High; Low - Medium	BC052561	Homo sapiens ubiquitously transcribed tetratricopeptide repeat gene, Y-linked (UTY), transcript variant 1, mRNA [NM_182660]
53	CCDC144A	NA		6.9906	0.0019958	2.6999	0.1838	Low - High; Medium - High; Low - Medium	NM_014695	Homo sapiens adenosine A3 receptor (ADORA3), transcript variant 2, mRNA [NM_000677]
54	LOC100652951	NA		6.9874	0.0020009	2.6988	0.1838	Low - High; Medium - High; Low - Medium	XR_132888	Homo sapiens chemokine (C-C motif) ligand 3-like 3 (CCL3L3), mRNA [NM_001001437]
55	BNIP3L		665	6.9741	0.0020222	2.6942	0.1838	Low - High; Medium - High; Low - Medium	NM_004331	1718340A KALIG-1 gene. {Homo sapiens} (exp=-1; wgp=0; cg=0), partial (11%) [THC2783530]
56	CCL3L3		414062	6.9335	0.0020885	2.6802	0.1842	Low - High; Medium - High; Low - Medium	NM_001001437	Homo sapiens G0/G1switch 2 (G0S2), mRNA [NM_015714]
57	GALNT14		79623	6.9167	0.0021167	2.6743	0.1842	High - Low; Medium - High; Medium - Low	NM_024572	Homo sapiens phorbol-12-myristate-13-acetate-induced protein 1 (PMAIP1), mRNA [NM_021127]
58	AKNA		80709	6.9046	0.0021372	2.6702	0.1842	Low - High; Medium - High; Low - Medium	AB075848	Homo sapiens fibroblast growth factor receptor 2 (FGFR2), transcript variant 2, mRNA [NM_022970]
59	EIF1AY		9086	6.8716	0.002194	2.6588	0.1859	Low - High; Medium - High; Low - Medium	NM_004681	Homo sapiens FBJ murine osteosarcoma viral oncogene homolog (FOS), mRNA [NM_005252]
60	LRRC14		9684	6.8482	0.0022355	2.6506	0.18623	Low - High; Medium - High; Low - Medium	NM_014665	phosphatase and actin regulator 1 [Source:HGNC Symbol;Acc:20990] [ENST00000379350]
61	ARHGEF4		50649	6.8276	0.0022724	2.6435	0.18623	Low - High; Medium - High; Low - Medium	NM_032995	Homo sapiens dual specificity phosphatase 2 (DUSP2), mRNA [NM_004418]
62	UTY		7404	6.7397	0.0024378	2.613	0.19655	Low - High; Medium - High; Low - Medium	NM_182660	Homo sapiens purinergic receptor P2Y, G-protein coupled, 2 (P2RY2), transcript variant 1, mRNA [NM_176072]

63	PGLYRP1		8993	6.7117	0.0024932	2.6032	0.19783	High - Low; High - Medium; Medium - Low	NM_005091	Homo sapiens serine/threonine kinase 17b (apoptosis-inducing), mRNA (cDNA clone IMAGE:6280720), partial cds. [BC052561]
64	XLOC_l2_013931	NA		6.6696	0.0025787	2.5886	0.20142	Low - High; Medium - High; Low - Medium		Homo sapiens protease, serine, 33 (PRSS33), mRNA [NM_152891]
65	ZNHIT2		741	6.6193	0.0026849	2.5711	0.20599	Low - High; Medium - High; Low - Medium	NM_014205	Q6IMA3_RAT (Q6IMA3) RAS-like family 11 member A, partial (6%) [THC2749835]
66	XLOC_008323	NA		6.6033	0.0027196	2.5655	0.20599	Low - High; Medium - High; Low - Medium		Uncharacterized proteincDNA FLJ27030 fis, clone SLV07741 [Source:UniProtKB/TrEMBL;Acc:Q6ZNW3] [ENST00000369474]
67	NAMPT		10135	6.5207	0.0029064	2.5366	0.21685	Low - High; Medium - High; Low - Medium	AK023341	Homo sapiens period homolog 1 (Drosophila) (PER1), mRNA [NM_002616]
68	LOC645195		645195	6.4959	0.0029652	2.5279	0.21798	Low - High; Medium - High; Low - Medium	AK123450	Homo sapiens small ILF3/NF90-associated RNA D (SNAR-D), small nuclear RNA [NR_024243]
69	C17orf103		256302	6.4746	0.0030166	2.5205	0.21855	Low - High; Medium - High; Low - Medium	NM_152914	Homo sapiens uncharacterized LOC100507254 (LOC100507254), non-coding RNA [NR_038981]
70	ADORA3		140	6.3769	0.0032643	2.4862	0.22529	High - Low; Medium - High; Medium - Low	NM_000677	BROAD Institute lincRNA (XLOC_012515), lincRNA [TCONS_00025237]
71	XLOC_003400	NA		6.3544	0.0033242	2.4783	0.22529	High - Low; Medium - High; Medium - Low		Homo sapiens solute carrier family 29 (nucleoside transporters), member 1 (SLC29A1), nuclear gene encoding mitochondrial protein, transcript variant 1, mRNA [NM_001078177]

72	SNORD90	NA		6.3471	0.003344	2.4757	0.22529	Low - High; Medium - High; Medium - Low	NR_003071	Homo sapiens tumor necrosis factor receptor superfamily, member 12A (TNFRSF12A), mRNA [NM_016639]
73	BEND7		222389	6.3197	0.0034191	2.4661	0.22529	High - Low; High - Medium; Medium - Low	NM_152751	BROAD Institute lincRNA (XLOC_003772), lincRNA [TCONS_00008317]
74	XLOC_l2_004611	NA		6.2853	0.0035158	2.454	0.22529	Low - High; Medium - High; Low - Medium		PREDICTED: Homo sapiens hypothetical LOC100506342 (LOC100506342), miscRNA [XR_108862]
75	FAM27E3		100131997	6.2841	0.0035189	2.4536	0.22529	High - Low; Medium - High; Medium - Low	XM_001720463	PREDICTED: Homo sapiens hypothetical LOC100652951 (LOC100652951), miscRNA [XR_132888]
76	CYP1B1-AS1	NA		6.2721	0.0035535	2.4493	0.22529	High - Low; High - Medium; Medium - Low	NR_027252	Homo sapiens amiloride binding protein 1 (amine oxidase (copper-containing)) (ABP1), mRNA [NM_001091]
77	ABP1		26	6.2653	0.0035733	2.4469	0.22529	High - Low; Medium - High; Medium - Low	NM_001091	Q2NUU5_SODGM (Q2NUU5) Glutamate-aspartate ABC transporter ATP-binding component GltL, partial (7%) [THC2533854]
78	MXI1		4601	6.2441	0.0036352	2.4395	0.22529	Low - High; Medium - High; Medium - Low	NM_005962	Homo sapiens BTG family, member 2 (BTG2), mRNA [NM_006763]
79	XLOC_007635	NA		6.2404	0.0036461	2.4382	0.22529	Low - High; High - Medium; Low - Medium		Homo sapiens uncharacterized LOC100507254 (LOC100507254), non-coding RNA [NR_038981]
80	DDHD2	NA		6.2323	0.0036701	2.4353	0.22529	Low - High; Medium - High; Low - Medium	NM_001164234	Homo sapiens mRNA; cDNA DKFZp761E2117 (from clone DKFZp761E2117). [AL831857]
81	RHOXF1		158800	6.2314	0.0036728	2.435	0.22529	Low - High; High - Medium; Low - Medium	NM_139282	SEC14-like 3 (S. cerevisiae) [Source:HGNC Symbol;Acc:18655] [ENST00000415957]

82	TGM2		7052	6.2238	0.0036955	2.4323	0.22529	Low - High; High - Medium; Low - Medium	NM_198951	Homo sapiens D-dopachrome tautomerase-like (LOC391322), mRNA [NM_001144931]
83	USP9Y		8287	6.2027	0.0037595	2.4249	0.22603	Low - High; Medium - High; Low - Medium	NM_004654	Homo sapiens DNA-damage-inducible transcript 4 (DDIT4), mRNA [NM_019058]
84	KDM5D		8284	6.1902	0.0037981	2.4204	0.22603	Low - High; Medium - High; Low - Medium	NM_004653	BROAD Institute lincRNA (XLOC_012043), lincRNA [TCONS_00025079]
85	EGR1		1958	6.1528	0.0039155	2.4072	0.23027	Low - High; Medium - High; Low - Medium	NM_001964	PREDICTED: Homo sapiens hypothetical LOC100507540 (LOC100507540), miscRNA [XR_108989]
86	CA4		762	6.0952	0.0041036	2.3868	0.23601	High - Low; High - Medium; Medium - Low	NM_000717	Homo sapiens CYP1B1 antisense RNA 1 (non-protein coding) (CYP1B1-AS1), non-coding RNA [NR_027252]
87	TTTY15	NA		6.0941	0.0041074	2.3864	0.23601	Low - High; Medium - High; Low - Medium	NR_001545	601818170F1 NIH_MGC_58 Homo sapiens cDNA clone IMAGE:4042121 5', mRNA sequence [BF130157]
88	NPRL3		8131	6.0386	0.0042978	2.3668	0.24414	Low - High; Medium - High; Medium - Low	NM_001039476	Homo sapiens TBC1 domain family, member 7 (TBC1D7), transcript variant 3, mRNA [NM_001143965]
89	DUSP1		1843	6.0193	0.0043662	2.3599	0.2447	Low - High; Medium - High; Low - Medium	NM_004417	Homo sapiens vesicle amine transport protein 1 homolog (T. californica) (VAT1), mRNA [NM_006373]
90	LOC391322		391322	6.0083	0.0044055	2.356	0.2447	Low - High; High - Medium; Low - Medium	NM_001144931	Homo sapiens sphingomyelin phosphodiesterase 3, neutral membrane (neutral sphingomyelinase II) (SMPD3), mRNA [NM_018667]
91	XLOC_011248	NA		5.987	0.004483	2.3484	0.24627	Low - High; Medium - High; Low - Medium	BC035091	Homo sapiens cytochrome P450, family 4, subfamily F, polypeptide 12 (CYP4F12), mRNA [NM_023944]

92	HS3ST1		9957	5.9496	0.0046225	2.3351	0.25117	Low - High; Medium - High; Medium - Low	NM_005114	Homo sapiens Charcot-Leyden crystal protein (CLC), mRNA [NM_001828]
93		NA		5.9349	0.0046785	2.3299	0.25148	Low - High; Medium - High; Low - Medium	CR997556	Homo sapiens Rhox homeobox family, member 1 (RHOXF1), mRNA [NM_139282]
94		NA		5.9095	0.0047768	2.3209	0.25302	High - Low; High - Medium; Medium - Low	S55273	K-EST0169216 L10ChoCK0 Homo sapiens cDNA clone L10ChoCK0-27-D04 5', mRNA sequence [CB121594]
95	OVGP1		5016	5.9015	0.0048084	2.318	0.25302	Low - High; Medium - High; Low - Medium	NM_002557	Homo sapiens egf-like module containing, mucin-like, hormone receptor-like 1 (EMR1), mRNA [NM_001974]
96	CCDC144A	NA		5.8835	0.0048799	2.3116	0.25348	Low - High; Medium - High; Low - Medium	NM_014695	Homo sapiens sialic acid binding Ig-like lectin 8 (SIGLEC8), mRNA [NM_014442]
97	XIST	NA		5.8631	0.0049625	2.3043	0.25348	High - Low; High - Medium; Medium - Low	NR_001564	Homo sapiens UDP-N-acetyl-alpha-D-galactosamine:polypeptide N-acetylgalactosaminyltransferase 14 (GalNAc-T14) (GALNT14), mRNA [NM_024572]
98	XIST	NA		5.8614	0.0049693	2.3037	0.25348	High - Low; High - Medium; Medium - Low	NR_001564	Homo sapiens olfactory receptor, family 2, subfamily M, member 7 (OR2M7), mRNA [NM_001004691]
99	TNFRSF12A		51330	5.8355	0.0050761	2.2945	0.25431	High - Low; High - Medium; Medium - Low	NM_016639	Homo sapiens shisa homolog 4 (Xenopus laevis) (SHISA4), transcript variant 1, mRNA [NM_198149]
100	XLOC_l2_015849	NA		5.8329	0.0050873	2.2935	0.25431	Low - High; Medium - High; Low - Medium	BX111592	Homo sapiens cystatin F (leukocystatin) (CST7), mRNA [NM_003650]
101	XPNPEP3	NA		5.7945	0.0052505	2.2798	0.2589	Low - High; Medium - High; Low - Medium	NM_001204827	Homo sapiens chemokine (C-C motif) ligand 23 (CCL23), transcript variant CKbeta8-1, mRNA [NM_005064]

102	NEAT1		283131	5.7871	0.0052825	2.2772	0.2589	Low - High; Medium - High; Low - Medium	AF001893	HUMDSAEC mitochondrial 3-oxoacyl-CoA thiolase {Homo sapiens} (exp=-1; wgp=0; cg=0), partial (14%) [THC2567789]
103	FAM129C		199786	5.7356	0.0055116	2.2587	0.26537	Low - High; Medium - High; Medium - Low	NM_001098524	Homo sapiens chromosome 9 open reading frame 40 (C9orf40), mRNA [NM_017998]
104	XIST	NA		5.7204	0.0055811	2.2533	0.26537	High - Low; High - Medium; Medium - Low	NR_001564	Homo sapiens spinster homolog 3 (Drosophila) (SPNS3), mRNA [NM_182538]
105	WNK4		65266	5.7202	0.0055821	2.2532	0.26537	Low - High; High - Medium; Low - Medium	NM_032387	Homo sapiens amphiregulin (AREG), mRNA [NM_001657]
106	HNF1A-AS1		283460	5.7032	0.0056609	2.2471	0.26537	Low - High; Medium - High; Low - Medium	NR_024345	Homo sapiens calcium channel, voltage-dependent, gamma subunit 6 (CACNG6), transcript variant 1, mRNA [NM_145814]
107	EIF1AY		9086	5.6991	0.00568	2.2457	0.26537	Low - High; Medium - High; Low - Medium	NM_004681	Homo sapiens trefoil factor 3 (intestinal) (TFF3), mRNA [NM_003226]
108	XIST	NA		5.6799	0.0057707	2.2388	0.26711	High - Low; High - Medium; Medium - Low	NR_001564	Homo sapiens carbonic anhydrase XIV (CA14), mRNA [NM_012113]
109	XIST	NA		5.6656	0.0058394	2.2336	0.26781	High - Low; High - Medium; Medium - Low	NR_001564	Homo sapiens phosphoinositide-3-kinase, regulatory subunit 6 (PIK3R6), mRNA [NM_001010855]
110	KLF14		136259	5.6417	0.005956	2.225	0.26935	Low - High; Medium - High; Low - Medium	NM_138693	Homo sapiens TSIX transcript, XIST antisense RNA (non-protein coding) (TSIX), antisense RNA [NR_003255]
111	FCRL2		79368	5.6274	0.006027	2.2199	0.26935	Low - High; Medium - High; Low - Medium	NM_030764	Homo sapiens ubiquitin specific peptidase 9, Y-linked (USP9Y), mRNA [NM_004654]

112	BMS1P5	NA	5.6213	0.0060573	2.2177	0.26935	Low - High; Medium - High; Low - Medium	BC065722	Homo sapiens T-cell activation RhoGTPase activating protein (TAGAP), transcript variant 3, mRNA [NM_138810]
113	LOC100509121	NA	5.6151	0.0060885	2.2155	0.26935	Low - High; Medium - High; Low - Medium	XM_003120334	Homo sapiens chymotrypsin-like elastase family, member 2B (CELA2B), mRNA [NM_015849]
114	C4BPA	722	5.5431	0.0064632	2.1896	0.28286	Low - High; High - Medium; Low - Medium	NM_000715	Homo sapiens matrix metallopeptidase 19 (MMP19), transcript variant 1, mRNA [NM_002429]
115	GOLGA8A	NA	5.5225	0.0065745	2.1821	0.28286	Low - High; Medium - High; Low - Medium	NR_027409	D80009 Start codon is not identified {Homo sapiens} (exp=-1; wgp=0; cg=0), partial (12%) [THC2480131]
116	TXLNG2P	246126	5.5193	0.0065917	2.181	0.28286	Low - High; Medium - High; Low - Medium	NR_045128	full-length cDNA clone CS0DL011YN21 of B cells (Ramos cell line) Cot 25-normalized of Homo sapiens (human) [CR607309]
117	DDX3Y	8653	5.5118	0.0066333	2.1783	0.28286	Low - High; Medium - High; Low - Medium	NM_001122665	Homo sapiens nuclear receptor subfamily 4, group A, member 2 (NR4A2), mRNA [NM_006186]
118	XIST	NA	5.5039	0.0066768	2.1754	0.28286	High - Low; High - Medium; Medium - Low	NR_001564	BROAD Institute lincRNA (XLOC_009548), lincRNA [TCONS_00019754]
119	LOC158863	158863	5.4863	0.0067749	2.1691	0.2846	Low - High; Medium - High; Low - Medium	AL110203	BROAD Institute lincRNA (XLOC_002172), lincRNA [TCONS_00003324]
120	XIST	7503	5.4602	0.0069237	2.1597	0.28843	High - Low; High - Medium; Medium - Low	NR_001564	Homo sapiens CD69 molecule (CD69), transcript variant 1, mRNA [NM_001781]
121	CCDC144A	9720	5.446	0.0070058	2.1545	0.28944	Low - High; Medium - High; Low - Medium	NM_014695	dedicator of cytokinesis 9 [Source:HGNC Symbol;Acc:14132] [ENST00000340449]

122	LOC100128262	NA		5.4248	0.0071307	2.1469	0.28957	Low - High; Medium - High; Medium - Low	XR_110396	Homo sapiens G protein-coupled receptor 44 (GPR44), mRNA [NM_004778]
123	MPP1		4354	5.4162	0.007182	2.1438	0.28957	Low - High; Medium - High; Low - Medium	NM_001166461	Homo sapiens protease, serine, 33 (PRSS33), mRNA [NM_152891]
124	HCG18	NA		5.4107	0.0072148	2.1418	0.28957	Low - High; Medium - High; Low - Medium	NR_024052	Homo sapiens disrupted in schizophrenia 1 (DISC1), transcript variant q, mRNA [NM_001164554]
125	C2orf63		130162	5.4064	0.0072406	2.1402	0.28957	Low - High; High - Medium; Low - Medium	NM_152385	Homo sapiens BMP and activin membrane-bound inhibitor homolog (Xenopus laevis) (BAMBI), mRNA [NM_012342]
126	MIA		8190	5.373	0.0074455	2.1281	0.29369	High - Low; High - Medium; Medium - Low	NM_006533	AT-hook transcription factor [Source:HGNC Symbol;Acc:24108] [ENST00000312033]
127	THBS3		7059	5.3692	0.0074686	2.1268	0.29369	High - Low; High - Medium; Medium - Low	CR933610	Homo sapiens chemokine (C-C motif) ligand 4 (CCL4), transcript variant 1, mRNA [NM_002984]
128	LINC00482		284185	5.361	0.00752	2.1238	0.29369	High - Low; High - Medium; Medium - Low	NR_038080	Homo sapiens leucine rich repeat neuronal 3 (LRRN3), transcript variant 3, mRNA [NM_018334]
129	ANKRD36BP2		645784	5.3435	0.0076311	2.1174	0.29513	Low - High; Medium - High; Low - Medium	NR_015424	Homo sapiens complement component 3a receptor 1 (C3AR1), mRNA [NM_004054]
130	RPS4Y2		140032	5.3366	0.007675	2.1149	0.29513	Low - High; Medium - High; Low - Medium	NM_001039567	BROAD Institute lincRNA (XLOC_001595), lincRNA [TCONS_00003799]
131	XLOC_l2_002886	NA		5.3106	0.0078433	2.1055	0.2993	Low - High; Medium - High; Low - Medium		Homo sapiens chemokine (C-C motif) ligand 4 (CCL4), transcript variant 1, mRNA [NM_002984]
132	SULT1A2		6799	5.3001	0.0079126	2.1017	0.29966	High - Low; High - Medium; Medium - Low	NM_177528	RAB44, member RAS oncogene family [Source:HGNC Symbol;Acc:21068] [ENST00000229824]

133	LOC652119		652119	5.282	0.0080333	2.0951	0.30194	Low - High; Medium - High; Low - Medium	XM_001720798	Homo sapiens CCAAT/enhancer binding protein (C/EBP), epsilon (CEBPE), mRNA [NM_001805]
134	C1orf140		400804	5.2635	0.0081582	2.0884	0.30232	High - Low; Medium - High; Medium - Low	NR_024236	BROAD Institute lincRNA (XLOC_l2_005490), lincRNA [TCONS_l2_00010465]
135	FOSB		2354	5.2609	0.0081761	2.0875	0.30232	Low - High; Medium - High; Low - Medium	NM_006732	Homo sapiens RAP1 GTPase activating protein 2 (RAP1GAP2), transcript variant 1, mRNA [NM_015085]
136	RGS14		10636	5.2513	0.008242	2.084	0.30232	High - Low; High - Medium; Medium - Low	NM_006480	Homo sapiens small nuclear ribonucleoprotein polypeptide N pseudogene (LOC100129534), non-coding RNA [NR_024489]
137	REREP3		646396	5.2451	0.0082853	2.0817	0.30232	Low - High; Medium - High; Low - Medium	NR_033735	Homo sapiens V-set and transmembrane domain containing 1 (VSTM1), mRNA [NM_198481]
138	RPS4Y1		6192	5.2265	0.0084149	2.0749	0.30483	Low - High; Medium - High; Low - Medium	NM_001008	
139	GFRA1		2674	5.2021	0.008589	2.0661	0.30721	High - Low; High - Medium; Medium - Low	NM_005264	Homo sapiens chemokine (C-C motif) receptor 3 (CCR3), transcript variant 1, mRNA [NM_001837]
140	LOC100289580		100289580	5.2001	0.0086036	2.0653	0.30721	Low - High; Medium - High; Low - Medium	AK127064	Homo sapiens leukotriene C4 synthase (LTC4S), mRNA [NM_145867]
141	XLOC_008276	NA		5.1381	0.0090633	2.0427	0.32133	Low - High; Medium - High; Low - Medium		Homo sapiens peripheral myelin protein 22 (PMP22), transcript variant 1, mRNA [NM_000304]
142	LOC100506003	NA		5.1207	0.0091964	2.0364	0.32375	Low - High; Medium - High; Low - Medium	XR_109785	Homo sapiens potassium inwardly-rectifying channel, subfamily J, member 15 (KCNJ15), transcript variant 1, mRNA [NM_170736]
143	HILPDA		29923	5.1095	0.0092833	2.0323	0.32452	High - Low; Medium - High; Medium - Low	NM_013332	BROAD Institute lincRNA (XLOC_006338), lincRNA [TCONS_00014249]

144	LOC340335	340335	5.0614	0.0096671	2.0147	0.3356	Low - High; Medium - High; Low - Medium	AK074459	Homo sapiens CYP1B1 antisense RNA 1 (non-protein coding) (CYP1B1-AS1), non-coding RNA [NR_027252]
145	XLOC_000683	NA	5.0323	0.0099069	2.0041	0.34155	High - Low; High - Medium; Medium - Low	BQ637851	BROAD Institute lincRNA (XLOC_l2_015752), lincRNA [TCONS_l2_00030522]
146	LOC100505857	NA	5.0186	0.010022	1.999	0.34315	Low - High; Medium - High; Low - Medium	DA569430	hd15d07.y1 Human Retina cDNA (Un-normalized, unamplified): hd [BQ637851]
147	XLOC_008151	NA	5.0047	0.01014	1.9939	0.34484	Low - High; Medium - High; Low - Medium	BC015977	Homo sapiens 5-oxoprolinase (ATP-hydrolysing) (OPLAH), mRNA [NM_017570]
148	VPRBP	9730	4.9908	0.01026	1.9889	0.34654	Low - High; Medium - High; Low - Medium	NM_014703	Homo sapiens aminolevulinate, delta-, synthase 2 (ALAS2), nuclear gene encoding mitochondrial protein, transcript variant 1, mRNA [NM_000032]
149	LOC283663	283663	4.9745	0.010402	1.9829	0.34675	Low - High; Medium - High; Low - Medium	NR_024433	Homo sapiens protease, serine, 41 (PRSS41), mRNA [NM_001135086]
150	XLOC_012571	NA	4.9742	0.010405	1.9828	0.34675	Low - High; High - Medium; Low - Medium		Homo sapiens MAX interactor 1 (MXI1), transcript variant 2, mRNA [NM_130439]
151	ORM2	5005	4.9514	0.010607	1.9744	0.35081	High - Low; High - Medium; Medium - Low	NM_000608	Homo sapiens tribbles homolog 1 (Drosophila) (TRIB1), mRNA [NM_025195]
152		NA	4.9448	0.010667	1.972	0.35081	Low - High; Medium - High; Low - Medium	XR_110395	BROAD Institute lincRNA (XLOC_008015), lincRNA [TCONS_00017433]
153	XLOC_l2_013300	NA	4.9346	0.010759	1.9682	0.35153	High - Low; High - Medium; Low - Medium	DB053965	Homo sapiens desmoplakin (DSP), transcript variant 1, mRNA [NM_004415]
154	TBC1D7	51256	4.8782	0.011284	1.9475	0.3621	Low - High; High - Medium; Low - Medium	NM_001143965	BX117229 Soares_NFL_T_GBC_S1 Homo sapiens cDNA clone IMAGp998I184005, mRNA sequence [BX117229]

155	RN5-8S1		100008587	4.8771	0.011296	1.9471	0.3621	Low - High; Medium - High; Low - Medium	NR_003285	Homo sapiens LIMS3-LOC440895 readthrough (LIMS3-LOC440895), non-coding RNA [NR_027145]
156	ITGB4		3691	4.8763	0.011303	1.9468	0.3621	High - Low; High - Medium; Medium - Low	NM_000213	Homo sapiens G protein-coupled receptor 34 (GPR34), transcript variant 4, mRNA [NM_001097579]
157	CYP4F12		66002	4.8691	0.011372	1.9442	0.3621	High - Low; High - Medium; Medium - Low	NM_023944	Homo sapiens phosphoserine phosphatase (PSPH), mRNA [NM_004577]
158	HPR		3250	4.8501	0.011557	1.9372	0.36471	High - Low; High - Medium; Medium - Low	NM_020995	Homo sapiens dual specificity phosphatase 1 (DUSP1), mRNA [NM_004417]
159	FLOT2		2319	4.8457	0.0116	1.9355	0.36471	High - Low; Medium - High; Medium - Low	NM_004475	Homo sapiens transmembrane phosphoinositide 3-phosphatase and tensin homolog 2 pseudogene 6 (TPTE2P6), non-coding RNA [NR_002815]
160	PRKY		5616	4.8321	0.011735	1.9305	0.36504	Low - High; Medium - High; Medium - Low	NR_028062	
161	MMP19		4327	4.8299	0.011757	1.9297	0.36504	Low - High; High - Medium; Low - Medium	NM_002429	
162		NA		4.8208	0.011847	1.9264	0.36559	High - Low; High - Medium; Medium - Low		PREDICTED: Homo sapiens hypothetical LOC100506897 (LOC100506897), miscRNA [XR_133523]
163	OPLAH		26873	4.8115	0.011942	1.9229	0.36605	High - Low; High - Medium; Medium - Low	NM_017570	CR742343 Homo sapiens library (Ebert L) Homo sapiens cDNA clone IMAGp971K0167 ; IMAGE:1623289 5', mRNA sequence [CR742343]
164	C12orf51		283450	4.8047	0.01201	1.9204	0.36605	Low - High; Medium - High; Low - Medium	NM_001109662	AGENCOURT_6822137 NIH_MGC_106 Homo sapiens cDNA clone IMAGE:5935069 5', mRNA sequence [BQ053372]

165	LOC286058		286058	4.7864	0.012199	1.9137	0.36605	Low - High; High - Medium; Low - Medium	AL833160	Homo sapiens long intergenic non-protein coding RNA 230A (LINC00230A), non-coding RNA [NR_002161]
166		NA		4.7728	0.01234	1.9087	0.36605	High - Low; High - Medium; Medium - Low		Homo sapiens ret finger protein-like 4A (RFPL4A), mRNA [NM_001145014]
167	XLOC_011570	NA		4.7701	0.012368	1.9077	0.36605	Low - High; Medium - High; Low - Medium		Homo sapiens annexin A3 (ANXA3), mRNA [NM_005139]
168	FAM27A		548321	4.7698	0.012372	1.9076	0.36605	High - Low; Medium - High; Medium - Low	NR_024060	
169	LOC100128252		100128252	4.7668	0.012403	1.9065	0.36605	Low - High; Medium - High; Medium - Low	NR_036522	BROAD Institute lincRNA (XLOC_l2_004546), lincRNA [TCONS_l2_00008281]
170	TSPAN5		10098	4.7625	0.012448	1.9049	0.36605	Low - High; Medium - High; Medium - Low	NM_005723	PREDICTED: Homo sapiens hypothetical LOC100131510 (LOC100131510), miscRNA [XR_109960]
171	BEND7		222389	4.7461	0.012624	1.8988	0.36905	High - Low; High - Medium; Medium - Low	NM_001100912	Homo sapiens beta-site APP-cleaving enzyme 2 (BACE2), transcript variant a, mRNA [NM_012105]
172	MYBPH		4608	4.7219	0.012886	1.8899	0.37218	High - Low; High - Medium; Medium - Low	NM_004997	
173	PRSS33		260429	4.7194	0.012914	1.8889	0.37218	High - Low; Medium - High; Medium - Low	NM_152891	Homo sapiens cellular retinoic acid binding protein 2 (CRABP2), transcript variant 2, mRNA [NM_001199723]
174	CCDC144A	NA		4.7131	0.012984	1.8866	0.37218	Low - High; Medium - High; Low - Medium	NM_014695	Homo sapiens TSIX transcript, XIST antisense RNA (non-protein coding) (TSIX), antisense RNA [NR_003255]
175	MTERFD2		130916	4.709	0.013029	1.8851	0.37218	Low - High; Medium - High; Low - Medium	NM_182501	BROAD Institute lincRNA (XLOC_010052), lincRNA [TCONS_00020761]
176	CST7		8530	4.6947	0.013188	1.8798	0.37459	High - Low; Medium - High; Medium - Low	NM_003650	Homo sapiens small ILF3/NF90-associated RNA G2 (SNAR-G2), small nuclear RNA [NR_024244]

177	SNORA34	NA		4.6845	0.013304	1.876	0.37574	Low - High; Medium - High; Low - Medium	NR_002968	DB089380 TESTI4 Homo sapiens cDNA clone TESTI4037937 5', mRNA sequence [DB089380]
178	HEMGN		55363	4.6766	0.013393	1.8731	0.37605	Low - High; Medium - High; Low - Medium	NM_018437	Homo sapiens mRNA; cDNA DKFZp686J1738 (from clone DKFZp686J1738). [CR933610]
179	XLOC_008185	NA		4.6575	0.013613	1.8661	0.37605	High - Low; High - Medium; Medium - Low		BX111592 Soares_testis_NHT Homo sapiens cDNA clone IMAGp998D162621, mRNA sequence [BX111592]
180	LOC100129534		100129534	4.652	0.013677	1.864	0.37605	Low - High; Medium - High; Low - Medium	NR_024489	Homo sapiens tetratricopeptide repeat domain 24 (TTC24), mRNA [NM_001105669]
181	MMP9		4318	4.6498	0.013703	1.8632	0.37605	High - Low; High - Medium; Medium - Low	NM_004994	Homo sapiens arachidonate 15-lipoxygenase (ALOX15), mRNA [NM_001140]
182	PLAC8		51316	4.6494	0.013708	1.863	0.37605	Low - High; Medium - High; Low - Medium	NM_001130715	Homo sapiens ankyrin repeat and BTB (POZ) domain containing 2 (ABTB2), mRNA [NM_145804]
183	XLOC_l2_006196	NA		4.6444	0.013766	1.8612	0.37605	Low - High; Medium - High; Low - Medium		DKFZp779O0656_r1 779 (synonym: hncc1) Homo sapiens cDNA clone DKFZp779O0656 5', mRNA sequence [BX500531]
184	DDX3Y		8653	4.6193	0.014064	1.8519	0.3821	Low - High; Medium - High; Low - Medium	NM_004660	BROAD Institute lincRNA (XLOC_l2_012524), lincRNA [TCONS_l2_00024199]
185	MAP7		9053	4.6101	0.014175	1.8485	0.38304	Low - High; High - Medium; Low - Medium	NM_003980	Homo sapiens cDNA FLJ37626 fis, clone BRCOC2014748. [AK094945]
186	HAGH		3029	4.5773	0.014579	1.8363	0.39062	Low - High; Medium - High; Low - Medium	NM_001040427	Homo sapiens mRNA; cDNA DKFZp686J2011 (from clone DKFZp686J2011). [AL833160]
187	LOC100131510		100131510	4.5714	0.014652	1.8341	0.39062	High - Low; High - Medium; Low - Medium	XR_109960	Homo sapiens Fc receptor-like 2 (FCRL2), mRNA [NM_030764]

188	CA14		23632	4.5683	0.01469	1.833	0.39062	Low - High; High - Medium; Low - Medium	NM_012113	Homo sapiens coiled-coil domain containing 144A (CCDC144A), mRNA [NM_014695]
189	XLOC_005981	NA		4.5403	0.015047	1.8225	0.398	High - Low; High - Medium; Medium - Low	BG434565	full-length cDNA clone CS0DC007YI01 of Neuroblastoma Cot 25-normalized of Homo sapiens (human) [CR604878]
190	CSNK1G2		1455	4.5141	0.015388	1.8128	0.4032	Low - High; High - Medium; Low - Medium	NM_001319	Homo sapiens egf-like module containing, mucin-like, hormone receptor-like 4 pseudogene (EMR4P), non-coding RNA [NR_024075]
191		NA		4.5056	0.015501	1.8097	0.4032	Low - High; Medium - High; Low - Medium	AK093811	Homo sapiens peptidoglycan recognition protein 1 (PGLYRP1), mRNA [NM_005091]
192	RNASE2		6036	4.5047	0.015513	1.8093	0.4032	High - Low; High - Medium; Medium - Low	NM_002934	DB516369 RIKEN full-length enriched human cDNA library, testis Homo sapiens cDNA clone H013051C16 3', mRNA sequence [DB516369]
193	LOC100134391	NA		4.5006	0.015567	1.8078	0.4032	Low - High; Medium - High; Low - Medium	XR_109425	Homo sapiens small Cajal body-specific RNA 6 (SCARNA6), guide RNA [NR_003006]
194	TNNI3		7137	4.4827	0.015808	1.8011	0.40601	High - Low; High - Medium; Medium - Low	NM_000363	Homo sapiens ankyrin repeat domain 36B pseudogene 2 (ANKRD36BP2), non-coding RNA [NR_015424]
195		NA		4.4766	0.015891	1.7989	0.40601	Low - High; Medium - High; Low - Medium		Homo sapiens coiled-coil domain containing 64B (CCDC64B), mRNA [NM_001103175]
196	LOC100289090		100289090	4.4688	0.015998	1.7959	0.40601	Low - High; High - Medium; Low - Medium	AK057625	Homo sapiens growth arrest and DNA-damage-inducible, gamma (GADD45G), mRNA [NM_006705]

197	XLOC_004308	NA	4.4686	0.016	1.7959	0.40601	High - Low; High - Medium; Medium - Low		Homo sapiens purinergic receptor P2Y, G-protein coupled, 14 (P2RY14), transcript variant 2, mRNA [NM_014879]
198	TRAK2	66008	4.4493	0.016267	1.7887	0.40852	High - Low; Medium - High; Medium - Low	NM_015049	
199	DPYSL4	10570	4.4421	0.016369	1.786	0.40852	Low - High; High - Medium; Low - Medium	NM_006426	Homo sapiens cDNA clone IMAGE:5498355. [BC110369]
200		NA	4.4415	0.016377	1.7858	0.40852	Low - High; Medium - High; Low - Medium		Homo sapiens olfactory receptor, family 6, subfamily T, member 1 (OR6T1), mRNA [NM_001005187]
201	XLOC_000377	NA	4.438	0.016426	1.7845	0.40852	High - Low; Medium - High; Medium - Low		Homo sapiens small proline-rich protein 2A (SPRR2A), mRNA [NM_005988]
202	XLOC_007868	NA	4.4304	0.016533	1.7816	0.40916	Low - High; Medium - High; Low - Medium		Homo sapiens MAX interactor 1 (MXI1), transcript variant 1, mRNA [NM_005962]
203		NA	4.423	0.016639	1.7789	0.40974	Low - High; Medium - High; Low - Medium	AK097351	BROAD Institute lincRNA (XLOC_l2_014694), lincRNA [TCONS_l2_00028604]
204	TNS1	7145	4.3762	0.017322	1.7614	0.42105	Low - High; High - Medium; Low - Medium	NM_022648	Q1YA75_STAAU (Q1YA75) ABC transporter related, partial (5%) [THC2632354]
205	LOC339807	339807	4.3663	0.01747	1.7577	0.42105	Low - High; High - Medium; Low - Medium	NR_034023	Homo sapiens MOCO sulphurase C-terminal domain containing 1 (MOSC1), nuclear gene encoding mitochondrial protein, mRNA [NM_022746]
206	LINC00230A	401630	4.3579	0.017597	1.7546	0.42105	Low - High; Medium - High; Medium - Low	NR_002161	Homo sapiens cDNA FLJ43642 fis, clone STOMA2004925. [AK125630]
207	LOC100506965	NA	4.3572	0.017608	1.7543	0.42105	Low - High; Medium - High; Low - Medium	XR_110300	Homo sapiens leucine-rich repeats and death domain containing 1 (LRRD1), mRNA [NM_001161528]

208	PGF	5228	4.3542	0.017654	1.7532	0.42105	Low - High; Medium - High; Medium - Low	NM_002632	Homo sapiens S-antigen; retina and pineal gland (arrestin) (SAG), mRNA [NM_000541]
209	XLOC_011719	NA	4.3409	0.017857	1.7482	0.42105	Low - High; Medium - High; Low - Medium		Homo sapiens T-cell leukemia/lymphoma 1A (TCL1A), transcript variant 1, mRNA [NM_021966]
210	VWA2	340706	4.3366	0.017924	1.7466	0.42105	Low - High; High - Medium; Low - Medium	NM_198496	Homo sapiens HRAS-like suppressor 2 (HRASLS2), mRNA [NM_017878]
211	XLOC_l2_001496	NA	4.3363	0.017929	1.7464	0.42105	High - Low; High - Medium; Medium - Low		immunoglobulin heavy constant delta [Source:HGNC Symbol;Acc:5480] [ENST00000390556]
212	ORMDL3	94103	4.329	0.018042	1.7437	0.42105	Low - High; High - Medium; Low - Medium	NM_139280	Homo sapiens fatty acid amide hydrolase (FAAH), mRNA [NM_001441]
213	LOC100506262	NA	4.3213	0.018162	1.7408	0.42105	High - Low; Medium - High; Medium - Low	XR_109925	BROAD Institute lincRNA (XLOC_005939), lincRNA [TCONS_00012372]
214	LOC145837	NA	4.3186	0.018204	1.7398	0.42105	Low - High; High - Medium; Low - Medium	NR_026979	Homo sapiens X-prolyl aminopeptidase (aminopeptidase P) 3, putative (XPNPEP3), transcript variant 2, mRNA [NM_001204827]
215	XLOC_006476	NA	4.3156	0.018251	1.7387	0.42105	Low - High; Medium - High; Low - Medium		Homo sapiens transmembrane protein 146 (TMEM146), mRNA [NM_152784]
216	XLOC_005810	NA	4.3154	0.018254	1.7387	0.42105	High - Low; High - Medium; Medium - Low		Homo sapiens uncharacterized LOC145837 (LOC145837), non-coding RNA [NR_026979]
217	C5orf38	153571	4.309	0.018355	1.7363	0.42105	High - Low; High - Medium; Low - Medium	NM_178569	Homo sapiens cDNA FLJ41456 fis, clone BRSTN2012320. [AK123450]
218	RNASE2	6036	4.3077	0.018375	1.7358	0.42105	High - Low; High - Medium; Medium - Low	NM_002934	Homo sapiens interleukin 3 receptor, alpha (low affinity) (IL3RA), mRNA [NM_002183]
219	OBSCN	84033	4.3005	0.01849	1.7331	0.42105	Low - High; Medium - High; Low - Medium	NM_052843	Homo sapiens claudin 5 (CLDN5), transcript variant 1, mRNA [NM_001130861]

220	XLOC_004828	NA		4.298	0.01853	1.7321	0.42105	Low - High; High - Medium; Low - Medium		Homo sapiens uncharacterized LOC100134317 (LOC100134317), non-coding RNA [NR_029389]
221	UTY		7404	4.2918	0.01863	1.7298	0.4214	Low - High; Medium - High; Medium - Low	NM_007125	Homo sapiens zinc finger, HIT-type containing 2 (ZNHIT2), mRNA [NM_014205]
222	XLOC_002172	NA		4.2665	0.019041	1.7203	0.42603	Low - High; High - Medium; Low - Medium		BROAD Institute lincRNA (XLOC_007635), lincRNA [TCONS_00016263]
223		NA		4.2609	0.019132	1.7182	0.42603	Low - High; Medium - High; Low - Medium		Homo sapiens transglutaminase 2 (C polypeptide, protein-glutamine-gamma-glutamyltransferase) (TGM2), transcript variant 2, mRNA [NM_198951]
224	LOC100287633	NA		4.2595	0.019156	1.7177	0.42603	Low - High; High - Medium; Low - Medium	XM_002342728	Q1YA75_STAAU (Q1YA75) ABC transporter related, partial (5%) [THC2632354]
225	IER5L		389792	4.2584	0.019175	1.7173	0.42603	Low - High; Medium - High; Low - Medium	NM_203434	Homo sapiens chromosome 1 open reading frame 190 (C1orf190), mRNA [NM_001013615]
226	HBE1		3046	4.2369	0.019534	1.7092	0.43094	High - Low; High - Medium; Low - Medium	NM_005330	Homo sapiens chromosome 12 open reading frame 51 (C12orf51), mRNA [NM_001109662]
227	CSRNP1		64651	4.2348	0.019568	1.7084	0.43094	Low - High; High - Medium; Low - Medium	NM_033027	Homo sapiens interleukin 5 receptor, alpha (IL5RA), transcript variant 3, mRNA [NM_175725]
228	UNC5C		8633	4.2285	0.019677	1.706	0.43142	Low - High; Medium - High; Low - Medium	NM_003728	BROAD Institute lincRNA (XLOC_002996), lincRNA [TCONS_00006377]
229		NA		4.2113	0.019971	1.6996	0.43494	Low - High; Medium - High; Low - Medium	AJ315539	Homo sapiens ataxia, cerebellar, Cayman type (ATCAY), mRNA [NM_033064]

230	XLOC_007868	NA		4.209	0.020011	1.6987	0.43494	Low - High; Medium - High; Low - Medium		Homo sapiens cysteine-rich secretory protein LCCL domain containing 2 (CRISPLD2), mRNA [NM_031476]
231	NOTCH2NL		388677	4.1999	0.02017	1.6953	0.43648	Low - High; Medium - High; Low - Medium	AK075065	
232	PRR5		55615	4.1859	0.020415	1.69	0.4399	Low - High; Medium - High; Low - Medium	NM_015366	Homo sapiens G protein-coupled receptor 141 (GPR141), mRNA [NM_181791]
233	CYP1B1-AS1	NA		4.1684	0.020726	1.6835	0.44468	High - Low; High - Medium; Medium - Low	NR_027252	Homo sapiens HLA complex group 18 (non-protein coding) (HCG18), non-coding RNA [NR_024052]
234	TSIX	NA		4.1596	0.020885	1.6802	0.44617	High - Low; High - Medium; Low - Medium	NR_003255	Homo sapiens regulator of G-protein signaling 14 (RGS14), mRNA [NM_006480]
235	THPO		7066	4.1319	0.021393	1.6697	0.45096	Low - High; Medium - High; Low - Medium	NM_000460	Homo sapiens BMS1 pseudogene 1 (BMS1P1), non-coding RNA [NR_026566]
236	HEBP1		50865	4.127	0.021484	1.6679	0.45096	High - Low; Medium - High; Medium - Low	NM_015987	Homo sapiens leucine rich repeat containing 14 (LRRC14), mRNA [NM_014665]
237	XLOC_009567	NA		4.1259	0.021505	1.6675	0.45096	Low - High; Medium - High; Low - Medium		
238	HP		3240	4.1257	0.021509	1.6674	0.45096	High - Low; High - Medium; Medium - Low	NM_005143	Homo sapiens neuralized homolog (Drosophila) (NEURL), mRNA [NM_004210]
239	XLOC_004093	NA		4.1229	0.02156	1.6663	0.45096	Low - High; High - Medium; Low - Medium		
240	XLOC_012618	NA		4.1071	0.021857	1.6604	0.4551	Low - High; Medium - High; Low - Medium		Homo sapiens chromosome 7 open reading frame 68 (C7orf68), transcript variant 1, mRNA [NM_013332]
241	XLOC_011924	NA		4.1028	0.02194	1.6588	0.4551	Low - High; High - Medium; Low - Medium	BI055436	is39c09.y1 HR85 islet Homo sapiens cDNA clone IMAGE:6554705 5', mRNA sequence [CB178477]

242	XLOC_006338	NA	4.0939	0.02211	1.6554	0.45673	High - Low; High - Medium; Medium - Low		Homo sapiens Rho guanine nucleotide exchange factor (GEF) 4 (ARHGEF4), transcript variant 2, mRNA [NM_032995]
243	MSR1	4481	4.0817	0.022345	1.6508	0.45968	High - Low; Medium - High; Medium - Low	NM_138715	BROAD Institute lincRNA (XLOC_002975), lincRNA [TCONS_00006356]
244	HIST1H1D	3007	4.0748	0.02248	1.6482	0.46057	Low - High; Medium - High; Low - Medium	NM_005320	ALU1_HUMAN (P39188) Alu subfamily J sequence contamination warning entry, partial (6%) [THC2539716]
245	SESN3	143686	4.0664	0.022644	1.645	0.461	Low - High; Medium - High; Medium - Low	NM_144665	Homo sapiens insulin-like growth factor binding protein 2, 36kDa (IGFBP2), mRNA [NM_000597]
246		NA	4.0557	0.022857	1.641	0.461	Low - High; Medium - High; Low - Medium	AK129975	Homo sapiens protease, serine, 36 (PRSS36), mRNA [NM_173502]
247	XLOC_l2_015752	NA	4.0552	0.022866	1.6408	0.461	Low - High; High - Medium; Low - Medium		Homo sapiens MAX dimerization protein 3 (MXD3), transcript variant 2, mRNA [NM_001142935]
248	LPPR4	9890	4.0514	0.022941	1.6394	0.461	High - Low; Medium - High; Medium - Low	NM_014839	
249	XLOC_l2_009468	NA	4.0465	0.02304	1.6375	0.461	Low - High; High - Medium; Low - Medium		Homo sapiens GDNF family receptor alpha 4 (GFRA4), transcript variant 2, mRNA [NM_145762]
250		NA	4.0458	0.023055	1.6372	0.461	Low - High; Medium - High; Low - Medium		Homo sapiens chromosome 17 open reading frame 103 (C17orf103), mRNA [NM_152914]
251	LRG1	116844	4.0357	0.023257	1.6334	0.46267	High - Low; High - Medium; Medium - Low	NM_052972	
252	IRAK1	3654	4.0278	0.023418	1.6305	0.46267	Low - High; Medium - High; Low - Medium	NM_001569	Homo sapiens coiled-coil domain containing 144A (CCDC144A), mRNA [NM_014695]
253	LOC100653206	NA	4.024	0.023496	1.629	0.46267	High - Low; High - Medium; Medium - Low	XR_133524	Homo sapiens TAO kinase 2 (TAOK2), transcript variant 1, mRNA [NM_016151]

254		NA		4.0234	0.023508	1.6288	0.46267	High - Low; High - Medium; Medium - Low		Homo sapiens ubiquitin-conjugating enzyme E2O (UBE2O), mRNA [NM_022066]
255	XLOC_012575	NA		4.0174	0.023631	1.6265	0.46325	Low - High; Medium - High; Low - Medium		serpin peptidase inhibitor, clade A (alpha-1 antiproteinase, antitrypsin), member 1 [Source:HGNC Symbol;Acc:8941] [ENST00000402629]
256	MEX3D		399664	4.005	0.023887	1.6218	0.46644	Low - High; High - Medium; Low - Medium	NM_203304	Homo sapiens desmoplakin (DSP), transcript variant 1, mRNA [NM_004415]
257	GDF5		8200	3.9961	0.024074	1.6185	0.46827	Low - High; Medium - High; Low - Medium	NM_000557	Homo sapiens BCL2/adenovirus E1B 19kDa interacting protein 3-like (BNIP3L), mRNA [NM_004331]
258	CEBPE		1053	3.9831	0.024347	1.6136	0.47	High - Low; Medium - High; Medium - Low	NM_001805	Homo sapiens uncharacterized LOC399972 (FLJ39051), non-coding RNA [NR_033839]
259	CREB5		9586	3.9799	0.024416	1.6123	0.47	High - Low; High - Medium; Medium - Low	NM_182898	Homo sapiens arginine-fifty homeobox (ARGFX), mRNA [NM_001012659]
260	SYCE3		644186	3.9763	0.024492	1.611	0.47	High - Low; High - Medium; Low - Medium	NM_001123225	Homo sapiens cholinergic receptor, muscarinic 4 (CHRM4), mRNA [NM_000741]
261	XLOC_008609	NA		3.9718	0.024589	1.6093	0.47	Low - High; High - Medium; Low - Medium		Homo sapiens chromosome 2 open reading frame 63 (C2orf63), transcript variant 1, mRNA [NM_152385]
262	XLOC_001362	NA		3.9627	0.024785	1.6058	0.47	Low - High; Medium - High; Low - Medium		Homo sapiens olfactory receptor, family 1, subfamily S, member 2 (OR1S2), mRNA [NM_001004459]
263	XLOC_008527	NA		3.9545	0.024962	1.6027	0.47	Low - High; High - Medium; Low - Medium		BROAD Institute lincRNA (XLOC_001595), lincRNA [TCONS_00003800]

264	LOC151009		151009	3.9502	0.025057	1.6011	0.47	Low - High; High - Medium; Low - Medium	NR_027244	Homo sapiens placenta-specific 8 (PLAC8), transcript variant 3, mRNA [NM_001130715]
265		NA		3.9398	0.025284	1.5972	0.47	Low - High; High - Medium; Low - Medium	BX096650	BROAD Institute lincRNA (XLOC_004828), lincRNA [TCONS_00009667]
266	XLOC_l2_002767	NA		3.9397	0.025286	1.5971	0.47	Low - High; High - Medium; Low - Medium		Homo sapiens ankyrin repeat domain 20 family, member A5, pseudogene (ANKRD20A5P), non-coding RNA [NR_040113]
267	XLOC_002749	NA		3.9391	0.025301	1.5969	0.47	Low - High; Medium - High; Low - Medium		BROAD Institute lincRNA (XLOC_007868), lincRNA [TCONS_00016491]
268	ZNF502		91392	3.9336	0.025421	1.5948	0.47	Low - High; Medium - High; Low - Medium	NM_033210	PREDICTED: Homo sapiens arachidonate 15-lipoxygenase-like (LOC100652883), mRNA [XM_003403469]
269	GGT8P		645367	3.9275	0.025557	1.5925	0.47	Low - High; Medium - High; Low - Medium	NR_003503	Homo sapiens heparan sulfate (glucosamine) 3-O-sulfotransferase 1 (HS3ST1), mRNA [NM_005114]
270	SLC26A8		116369	3.9257	0.025598	1.5918	0.47	High - Low; Medium - High; Medium - Low	NM_052961	BROAD Institute lincRNA (XLOC_002125), lincRNA [TCONS_00003303]
271	ARGFX		503582	3.9193	0.02574	1.5894	0.47	Low - High; High - Medium; Low - Medium	NM_001012659	Homo sapiens beta-defensin 131-like (LOC100129216), mRNA [NM_001242853]
272	LOC100506533		100506533	3.9114	0.025918	1.5864	0.47	High - Low; Medium - High; Medium - Low	XM_003118505	Homo sapiens flotillin 2 (FLOT2), mRNA [NM_004475]
273	OLIG1		116448	3.9112	0.025924	1.5863	0.47	High - Low; Medium - High; Medium - Low	NM_138983	Homo sapiens uncharacterized LOC400804 (C1orf140), non-coding RNA [NR_024236]
274	ITGB2		3689	3.9051	0.026062	1.584	0.47	High - Low; Medium - High; Medium - Low		Homo sapiens DDHD domain containing 2 (DDHD2), transcript variant 3, mRNA [NM_001164234]
275	LENG8		114823	3.9016	0.026142	1.5827	0.47	Low - High; Medium - High; Low - Medium	NM_052925	Homo sapiens paraneoplastic antigen like 6C (PNMA6C), mRNA [NM_001170944]

276	ZFY		7544	3.8987	0.026209	1.5816	0.47	Low - High; Medium - High; Low - Medium	NM_003411	BROAD Institute lincRNA (XLOC_007219), lincRNA [TCONS_00014537]
277	FCGBP		8857	3.8922	0.026358	1.5791	0.47	Low - High; Medium - High; Low - Medium	NM_003890	Homo sapiens cDNA FLJ43625 fis, clone SPLEN2024127. [AK125613]
278	SNORD3B-1		26851	3.8919	0.026365	1.579	0.47	Low - High; Medium - High; Medium - Low	NR_003271	BROAD Institute lincRNA (XLOC_011570), lincRNA [TCONS_00023775]
279	CAMP		820	3.8897	0.026416	1.5781	0.47	High - Low; High - Medium; Medium - Low	NM_004345	guanylate cyclase 1, soluble, beta 2 [Source:HGNC Symbol;Acc:4686] [ENST00000389600]
280	CEACAM8		1088	3.8803	0.026634	1.5746	0.47	High - Low; High - Medium; Medium - Low	NM_001816	Homo sapiens ankyrin repeat domain 36 (ANKRD36), mRNA [NM_001164315]
281	ALPL		249	3.8781	0.026684	1.5737	0.47	High - Low; Medium - High; Medium - Low	NM_000478	Homo sapiens epsin 2 (EPN2), transcript variant 2, mRNA [NM_014964]
282	NCRNA00185		55410	3.8738	0.026785	1.5721	0.47	Low - High; Medium - High; Medium - Low	NR_001544	BROAD Institute lincRNA (XLOC_009790), lincRNA [TCONS_00020459]
283	RNASE3		6037	3.8721	0.026825	1.5715	0.47	High - Low; High - Medium; Low - Medium	NM_002935	BROAD Institute lincRNA (XLOC_013445), lincRNA [TCONS_00028489]
284	LMOD1		25802	3.8706	0.02686	1.5709	0.47	Low - High; Medium - High; Low - Medium	NM_012134	Homo sapiens adenylate cyclase 4 (ADCY4), transcript variant 3, mRNA [NM_001198568]
285	NASP		4678	3.8678	0.026926	1.5698	0.47	Low - High; High - Medium; Low - Medium	NM_002482	Homo sapiens progestin and adipoQ receptor family member VI (PAQR6), transcript variant 1, mRNA [NM_024897]
286	MOV10L1		54456	3.8663	0.026961	1.5693	0.47	Low - High; Medium - High; Low - Medium	NM_018995	Q40PK4_DESAC (Q40PK4) IMP dehydrogenase/GMP reductase:Histidine kinase, HAMP region:Bacterial chemotaxis sensory transducer precursor, partial (3%) [THC2722577]

287	XLOC_008586	NA		3.8648	0.026998	1.5687	0.47	Low - High; High - Medium; Low - Medium		Homo sapiens solute carrier family 16, member 14 (monocarboxylic acid transporter 14) (SLC16A14), mRNA [NM_152527]
288	NPPA		4878	3.8558	0.027211	1.5653	0.47	Low - High; Medium - High; Low - Medium		Homo sapiens HNF1A antisense RNA 1 (non-protein coding) (HNF1A-AS1), non-coding RNA [NR_024345]
289	XLOC_012145	NA		3.8524	0.02729	1.564	0.47	Low - High; High - Medium; Low - Medium		Homo sapiens transmembrane protein 164 (TMEM164), transcript variant 2, mRNA [NM_032227]
290	TUBB2A		7280	3.8467	0.027428	1.5618	0.47	Low - High; Medium - High; Medium - Low	NM_001069	
291	LOC728190		728190	3.8456	0.027455	1.5614	0.47	High - Low; Medium - High; Medium - Low	NR_024397	Homo sapiens tensin 1 (TNS1), mRNA [NM_022648]
292	C1orf190		541468	3.8443	0.027486	1.5609	0.47	Low - High; Medium - High; Low - Medium	NM_001013615	Q2NY92_XANOM (Q2NY92) Methyltransferase, partial (3%) [THC2714968]
293	TGFBR2		7048	3.8411	0.027562	1.5597	0.47	Low - High; Medium - High; Low - Medium	AJ786388	Homo sapiens cDNA FLJ43093 fis, clone CORDB1000140. [AK125083]
294	MDGA1		266727	3.8378	0.027642	1.5584	0.47	Low - High; Medium - High; Low - Medium	AK126965	Homo sapiens major facilitator superfamily domain containing 6-like (MFSD6L), mRNA [NM_152599]
295	CECR6		27439	3.8202	0.02807	1.5518	0.47359	High - Low; High - Medium; Medium - Low	NM_031890	Homo sapiens nuclear receptor subfamily 4, group A, member 2 (NR4A2), mRNA [NM_006186]
296	CHRM4		1132	3.8141	0.028222	1.5494	0.47359	High - Low; Medium - High; Medium - Low	NM_000741	RC0-GN0275-060201-032-g10 GN0275 Homo sapiens cDNA, mRNA sequence [BI055436]
297	MORN1		79906	3.8125	0.028262	1.5488	0.47359	Low - High; High - Medium; Low - Medium		BROAD Institute lincRNA (XLOC_007868), lincRNA [TCONS_00016491]

298	PAQR6	79957	3.8106	0.028309	1.5481	0.47359	High - Low; High - Medium; Medium - Low	NM_024897	Homo sapiens cannabinoid receptor 2 (macrophage) (CNR2), mRNA [NM_001841]
299	XLOC_009123	NA	3.8065	0.028409	1.5465	0.47359	High - Low; High - Medium; Low - Medium	XR_111174	Homo sapiens pyruvate dehydrogenase kinase, isozyme 2 (PDK2), nuclear gene encoding mitochondrial protein, transcript variant 4, mRNA [NM_001199900]
300	XLOC_014408	NA	3.8061	0.028421	1.5464	0.47359	Low - High; Medium - High; Low - Medium		Homo sapiens oviductal glycoprotein 1, 120kDa (OVGP1), mRNA [NM_002557]
301	LOC100287225	NA	3.7906	0.02881	1.5405	0.47635	Low - High; Medium - High; Low - Medium	NR_040074	CD69 molecule [Source:HGNC Symbol;Acc:1694] [ENST00000416624]
302	PRKDC	5591	3.7881	0.028871	1.5395	0.47635	High - Low; High - Medium; Medium - Low	NM_006904	Homo sapiens WNK lysine deficient protein kinase 4 (WNK4), mRNA [NM_032387]
303	ORM1	5004	3.7866	0.028911	1.5389	0.47635	High - Low; High - Medium; Medium - Low	NM_000607	Homo sapiens olfactomedin 4 (OLFM4), mRNA [NM_006418]
304	KCNG1	3755	3.7843	0.028968	1.5381	0.47635	Low - High; Medium - High; Medium - Low	NM_002237	Homo sapiens dipeptidyl-peptidase 6, mRNA (cDNA clone IMAGE:5494573), complete cds. [BC035912]
305	FAAH	2166	3.769	0.02936	1.5322	0.48121	Low - High; Medium - High; Low - Medium	NM_001441	BROAD Institute lincRNA (XLOC_004535), lincRNA [TCONS_00009501]
306	XLOC_002996	NA	3.7489	0.029884	1.5246	0.48772	Low - High; Medium - High; Low - Medium		Homo sapiens collagen, type XX, alpha 1 (COL20A1), mRNA [NM_020882]
307	XLOC_005900	NA	3.7379	0.030173	1.5204	0.48772	High - Low; Medium - High; Medium - Low	AK130765	Homo sapiens alkaline phosphatase, liver/bone/kidney (ALPL), transcript variant 1, mRNA [NM_000478]
308	MIR22HG	84981	3.7368	0.030201	1.52	0.48772	Low - High; Medium - High; Low - Medium	NR_028502	

309	XLOC_l2_015892	NA		3.7351	0.030248	1.5193	0.48772	Low - High; Medium - High; Low - Medium		Homo sapiens gamma-glutamyltransferase 8 pseudogene (GGT8P), non-coding RNA [NR_003503]
310	SYNJ2		8871	3.7339	0.030279	1.5189	0.48772	Low - High; Medium - High; Low - Medium	AK296242	Homo sapiens keratin associated protein 19-2 (KRTAP19-2), mRNA [NM_181608]
311	TNNT1		7138	3.7315	0.030342	1.518	0.48772	High - Low; High - Medium; Medium - Low	NM_003283	ALU1_HUMAN (P39188) Alu subfamily J sequence contamination warning entry, partial (7%) [THC2739199]
312	ADORA3		140	3.7264	0.030479	1.516	0.48835	High - Low; High - Medium; Medium - Low	NM_020683	Homo sapiens coiled-coil domain containing 144A (CCDC144A), mRNA [NM_014695]
313	EGR3		1960	3.7072	0.030999	1.5087	0.49275	Low - High; High - Medium; Low - Medium	NM_004430	Homo sapiens uncharacterized LOC100132987 (LOC100132987), non-coding RNA [NR_038985]
314	EYA1		2138	3.7002	0.031189	1.506	0.49275	Low - High; High - Medium; Low - Medium	NM_000503	BROAD Institute lincRNA (XLOC_l2_013963), lincRNA [TCONS_l2_00026845]
315	XLOC_001823	NA		3.6999	0.031197	1.5059	0.49275	Low - High; Medium - High; Medium - Low		Homo sapiens acyl-CoA thioesterase 11 (ACOT11), transcript variant 2, mRNA [NM_147161]
316		NA		3.6973	0.031269	1.5049	0.49275	Low - High; Medium - High; Low - Medium		Homo sapiens uncharacterized LOC339807 (LOC339807), non-coding RNA [NR_034023]
317	XLOC_l2_015855	NA		3.6971	0.031274	1.5048	0.49275	Low - High; Medium - High; Low - Medium		CR997556 RZPD no.9016 Homo sapiens cDNA clone RZPDp9016I2412 5', mRNA sequence [CR997556]
318	ANKRD20A5P		440482	3.6945	0.031345	1.5038	0.49275	High - Low; High - Medium; Low - Medium	NR_040113	Homo sapiens oligodendrocyte lineage transcription factor 2 (OLIG2), mRNA [NM_005806]
319		NA		3.6811	0.031718	1.4987	0.49705	Low - High; High - Medium; Low - Medium		Homo sapiens lysophosphatidylcholine acyltransferase 2 (LPCAT2), mRNA [NM_017839]

320	LOC100288292		100288292	3.6767	0.031842	1.497	0.49743	Low - High; Medium - High; Low - Medium	XR_132538	Q70J93_STRGR (Q70J93) NDP-3-ketoreductase, partial (6%) [THC2606101]
321	PANX2		56666	3.6639	0.032203	1.4921	0.5015	High - Low; High - Medium; Medium - Low	NM_052839	Homo sapiens cDNA FLJ13279 fis, clone OVARC1001055, moderately similar to PRE-B CELL ENHANCING FACTOR PRECURSOR. [AK023341]
322	RFPL4A		342931	3.6468	0.032689	1.4856	0.50196	Low - High; Medium - High; Medium - Low	NM_001145014	Homo sapiens unknown mRNA sequence. [AY010113]
323	XLOC_l2_015212	NA		3.6453	0.032733	1.485	0.50196	High - Low; High - Medium; Medium - Low		Homo sapiens chromosome 19 open reading frame 59 (C19orf59), mRNA [NM_174918]
324	RBM38		55544	3.6378	0.03295	1.4821	0.50196	Low - High; Medium - High; Medium - Low	NM_017495	Homo sapiens cDNA FLJ43119 fis, clone CTONG3003179. [AK125109]
325		NA		3.6377	0.032955	1.4821	0.50196	Low - High; Medium - High; Low - Medium	XR_132817	PREDICTED: Homo sapiens hypothetical LOC100132046 (LOC100132046), miscRNA [XR_132817]
326	VSIG10		54621	3.6324	0.033108	1.4801	0.50196	High - Low; High - Medium; Medium - Low	NM_019086	Homo sapiens potassium voltage-gated channel, subfamily G, member 1 (KCNG1), mRNA [NM_002237]
327	NAT10		55226	3.6317	0.03313	1.4798	0.50196	Low - High; Medium - High; Low - Medium	NM_024662	Homo sapiens zinc and ring finger 3 (ZNRF3), transcript variant 1, mRNA [NM_001206998]
328	XLOC_l2_013963	NA		3.6306	0.033161	1.4794	0.50196	Low - High; Medium - High; Low - Medium		BC010854 amyloid beta A4 precursor protein-binding, family B, member 1, isoform delta E9 {Homo sapiens} (exp=-1; wgp=0; cg=0), partial (22%) [THC2503819]

329	XLOC_012503	NA		3.6261	0.033292	1.4777	0.50196	High - Low; Medium - High; Medium - Low		amyloid beta (A4) precursor protein-binding, family A, member 2 [Source:HGNC Symbol;Acc:579] [ENST00000382938]
330	XLOC_002125	NA		3.6206	0.033455	1.4755	0.50196	Low - High; Medium - High; Low - Medium	AL355732	
331	ZNF646		9726	3.6153	0.033612	1.4735	0.50196	Low - High; High - Medium; Low - Medium	NM_014699	BROAD Institute lincRNA (XLOC_000535), lincRNA [TCONS_00001250]
332	CAV1		857	3.612	0.033708	1.4723	0.50196	Low - High; High - Medium; Low - Medium	NM_001753	Homo sapiens Fc fragment of IgG, high affinity Ib, receptor (CD64) (FCGR1B), transcript variant 1, mRNA [NM_001017986]
333	SPP1		6696	3.6076	0.033839	1.4706	0.50196	High - Low; High - Medium; Medium - Low	NM_001040058	BROAD Institute lincRNA (XLOC_007895), lincRNA [TCONS_00016511]
334	FOXB1		27023	3.6048	0.033924	1.4695	0.50196	Low - High; High - Medium; Low - Medium	NM_012182	Homo sapiens Kruppel-like factor 14 (KLF14), mRNA [NM_138693]
335	LGR6		59352	3.6039	0.033951	1.4691	0.50196	Low - High; Medium - High; Low - Medium	NM_001017403	Homo sapiens indoleamine 2,3-dioxygenase 1 (IDO1), mRNA [NM_002164]
336	YOD1		55432	3.5968	0.034164	1.4664	0.50196	High - Low; Medium - High; Medium - Low	NM_018566	HSU07000 breakpoint cluster region protein {Homo sapiens} (exp=-1; wgp=0; cg=0), partial (11%) [THC2481061]
337	ATP6V0D1		9114	3.5958	0.034194	1.466	0.50196	Low - High; Medium - High; Low - Medium	AK125522	Homo sapiens Vpr (HIV-1) binding protein (VPRBP), transcript variant 1, mRNA [NM_014703]
338	LOC100507195	NA		3.5897	0.03438	1.4637	0.50196	High - Low; High - Medium; Medium - Low	XR_110374	Homo sapiens cDNA FLJ37669 fis, clone BRHIP2011891. [AK094988]
339	GRB10		2887	3.5846	0.034534	1.4617	0.50196	High - Low; High - Medium; Medium - Low	NM_001001555	Homo sapiens unc-5 homolog C (C. elegans) (UNC5C), mRNA [NM_003728]
340	XLOC_002580	NA		3.5844	0.034539	1.4617	0.50196	Low - High; Medium - High; Low - Medium		Homo sapiens nuclear receptor subfamily 4, group A, member 3 (NR4A3), transcript variant 3, mRNA [NM_173200]

341	ACVRL1	94	3.5803	0.034666	1.4601	0.50196	High - Low; High - Medium; Medium - Low	NM_000020	Homo sapiens double homeobox 4 (DUX4), mRNA [NM_033178]
342	XLOC_007407	NA	3.5766	0.034779	1.4587	0.50196	Low - High; Medium - High; Low - Medium		Homo sapiens CD248 molecule, endosialin (CD248), mRNA [NM_020404]
343	C10orf76	79591	3.5758	0.034803	1.4584	0.50196	Low - High; Medium - High; Low - Medium	NM_024541	Homo sapiens small nucleolar RNA, H/ACA box 39 (SNORA39), small nucleolar RNA [NR_002972]
344		NA	3.5753	0.034818	1.4582	0.50196	Low - High; Medium - High; Low - Medium	BC043420	Homo sapiens transmembrane and coiled-coil domain family 2 (TMCC2), transcript variant 1, mRNA [NM_014858]
345	NCAPH	23397	3.5696	0.034993	1.456	0.50196	Low - High; Medium - High; Low - Medium	NM_015341	Homo sapiens carbonic anhydrase IV (CA4), mRNA [NM_000717]
346		NA	3.5692	0.035008	1.4558	0.50196	High - Low; Medium - High; Medium - Low	AF495725	
347	XLOC_004710	NA	3.5612	0.035256	1.4528	0.50196	Low - High; High - Medium; Low - Medium		PREDICTED: Homo sapiens hypothetical LOC100134391 (LOC100134391), miscRNA [XR_109425]
348	XLOC_012594	NA	3.5562	0.03541	1.4509	0.50196	Low - High; High - Medium; Low - Medium		BROAD Institute lincRNA (XLOC_000751), lincRNA [TCONS_00001436]
349	XLOC_011104	NA	3.5536	0.035491	1.4499	0.50196	High - Low; Medium - High; Medium - Low	AX721190	BROAD Institute lincRNA (XLOC_l2_015855), lincRNA [TCONS_l2_00030882]
350	TUBB8	347688	3.553	0.035511	1.4496	0.50196	High - Low; Medium - High; Medium - Low	NM_177987	Homo sapiens Fc fragment of IgG, high affinity Ib, receptor (CD64) (FCGR1B), transcript variant 3, mRNA [NM_001244910]
351	FAM127B	26071	3.5523	0.035532	1.4494	0.50196	High - Low; Medium - High; Medium - Low	NM_001078172	BROAD Institute lincRNA (XLOC_012166), lincRNA [TCONS_00025346]
352	C19orf59	199675	3.5494	0.035626	1.4482	0.50196	High - Low; High - Medium; Medium - Low	NM_174918	ALU2_HUMAN (P39189) Alu subfamily SB sequence contamination warning entry, partial (15%) [THC2520829]

353	LOC100131820	100131820	3.5461	0.035727	1.447	0.50196	Low - High; Medium - High; Low - Medium	AK125613	BROAD Institute lincRNA (XLOC_007407), lincRNA [TCONS_00016016]
354	XLOC_006277	NA	3.5408	0.035897	1.4449	0.50196	High - Low; High - Medium; Medium - Low		Homo sapiens non-SMC condensin I complex, subunit H (NCAPH), mRNA [NM_015341]
355	SLC41A3	54946	3.5405	0.035905	1.4448	0.50196	Low - High; Medium - High; Low - Medium	NM_017836	BROAD Institute lincRNA (XLOC_005082), lincRNA [TCONS_00010555]
356	NOP56	NA	3.5402	0.035916	1.4447	0.50196	High - Low; Medium - High; Medium - Low	NM_006392	Homo sapiens cDNA FLJ45013 fis, clone BRAWH3013508. [AK126960]
357	XLOC_008015	NA	3.5377	0.035996	1.4437	0.50196	High - Low; High - Medium; Medium - Low		BROAD Institute lincRNA (XLOC_003400), lincRNA [TCONS_00007354]
358	XLOC_011081	NA	3.5371	0.036013	1.4435	0.50196	High - Low; High - Medium; Low - Medium	AK124327	Homo sapiens uncharacterized LOC100505978 (LOC100505978), non-coding RNA [NR_038912]
359	XLOC_l2_003666	NA	3.5283	0.036296	1.4401	0.50196	Low - High; High - Medium; Low - Medium		adenosine monophosphate deaminase 3 [Source:HGNC Symbol;Acc:470] [ENST00000527261]
360	CEP104	9731	3.5257	0.03638	1.4391	0.50196	Low - High; Medium - High; Low - Medium	BC050721	Lymphocyte antigen 6 complex, locus G6ELymphocyte antigen 6 complex, locus G6E, isoform CRA_aUncharacterized protein [Source:UniProtKB/TrEMBL;Acc:A2ABS3] [ENST00000383417]
361	XLOC_002275	NA	3.5115	0.036839	1.4337	0.50196	Low - High; High - Medium; Low - Medium		Homo sapiens oligodendrocyte transcription factor 1 (OLIG1), mRNA [NM_138983]

362	XLOC_000884	NA		3.5091	0.036916	1.4328	0.50196	Low - High; High - Medium; Low - Medium		BX331476 Homo sapiens NEUROBLASTOMA COT 10-NORMALIZED Homo sapiens cDNA clone CS0DB006YM22 5-PRIME, mRNA sequence [BX331476]
363	LOC728052	NA		3.5067	0.036995	1.4319	0.50196	Low - High; Medium - High; Low - Medium	XR_108619	BROAD Institute lincRNA (XLOC_008609), lincRNA [TCONS_00018331]
364	XLOC_007738	NA		3.4971	0.037311	1.4282	0.50196	Low - High; Medium - High; Low - Medium		BROAD Institute lincRNA (XLOC_002263), lincRNA [TCONS_00004378]
365	NASP	NA		3.4958	0.037354	1.4277	0.50196	Low - High; High - Medium; Low - Medium	NM_002482	BROAD Institute lincRNA (XLOC_007974), lincRNA [TCONS_00017163]
366	CCDC144A		9720	3.4929	0.03745	1.4265	0.50196	Low - High; Medium - High; Low - Medium	NM_014695	Homo sapiens 3-hydroxymethyl-3-methylglutaryl-CoA lyase-like 1 (HMGCLL1), transcript variant 1, mRNA [NM_019036]
367	TUBB2A		7280	3.4918	0.037485	1.4261	0.50196	Low - High; Medium - High; Medium - Low	NM_001069	Homo sapiens 1-aminocyclopropane-1-carboxylate synthase homolog (Arabidopsis)(non-functional)-like (ACCSL), mRNA [NM_001031854]
368	RNF182		221687	3.4893	0.037569	1.4252	0.50196	High - Low; Medium - High; Medium - Low	NM_152737	
369	XLOC_003405	NA		3.4871	0.037645	1.4243	0.50196	Low - High; Medium - High; Low - Medium		Q9V7K8_DROME (Q9V7K8) CG15706-PA (RE36877p), partial (5%) [THC2675915]
370	LOC100507540	NA		3.4795	0.037897	1.4214	0.50196	High - Low; Medium - High; Medium - Low	XR_108989	Homo sapiens killer cell immunoglobulin-like receptor, two domains, long cytoplasmic tail, 5A (KIR2DL5A), mRNA [NM_020535]
371	LOC100506342		100506342	3.4656	0.038369	1.416	0.50196	Low - High; High - Medium; Low - Medium	XR_108862	Homo sapiens glutathione S-transferase theta 1 (GSTT1), mRNA [NM_000853]

372	ZNF385C	NA	3.4641	0.038419	1.4155	0.50196	Low - High; High - Medium; Low - Medium	NM_001242704	solute carrier family 25, member 37 [Source:HGNC Symbol;Acc:29786] [ENST00000519973]
373	SRRM3	222183	3.4623	0.03848	1.4148	0.50196	Low - High; High - Medium; Low - Medium	NM_001110199	Homo sapiens WDNM1-like pseudogene (LOC645638), non-coding RNA [NR_030732]
374		NA	3.4586	0.038606	1.4133	0.50196	Low - High; High - Medium; Low - Medium		Homo sapiens RAB2B, member RAS oncogene family (RAB2B), transcript variant 1, mRNA [NM_032846]
375	XLOC_l2_008609	NA	3.4542	0.038755	1.4117	0.50196	Low - High; High - Medium; Low - Medium		Homo sapiens kringle containing transmembrane protein 1 (KREMEN1), transcript variant 3, mRNA [NM_001039570]
376	XLOC_003194	NA	3.4529	0.038801	1.4112	0.50196	Low - High; High - Medium; Low - Medium		Homo sapiens Fc fragment of IgG, high affinity Ib, receptor (CD64) (FCGR1B), transcript variant 3, mRNA [NM_001244910]
377	RBM3	5935	3.4524	0.03882	1.4109	0.50196	Low - High; Medium - High; Low - Medium	NM_006743	Homo sapiens HOX transcript antisense RNA (non-protein coding) (HOTAIR), antisense RNA [NR_003716]
378		NA	3.448	0.03897	1.4093	0.50196	Low - High; High - Medium; Low - Medium		Homo sapiens growth differentiation factor 5 (GDF5), mRNA [NM_000557]
379	PLK3	1263	3.4434	0.039129	1.4075	0.50196	Low - High; High - Medium; Low - Medium	NM_004073	BROAD Institute lincRNA (XLOC_l2_000037), lincRNA [TCONS_l2_00000066]
380		NA	3.4418	0.039184	1.4069	0.50196	Low - High; Medium - High; Low - Medium	AK093943	Homo sapiens chromosome 5 open reading frame 38 (C5orf38), mRNA [NM_178569]
381	SLC6A10P	386757	3.4412	0.039204	1.4067	0.50196	Low - High; Medium - High; Medium - Low	NR_003083	BROAD Institute lincRNA (XLOC_005780), lincRNA [TCONS_00012221]
382	DACH1	1602	3.4379	0.039322	1.4054	0.50196	High - Low; High - Medium; Medium - Low	NM_080759	BROAD Institute lincRNA (XLOC_l2_015212), lincRNA [TCONS_l2_00029343]

383	HRC		3270	3.4379	0.039322	1.4054	0.50196	Low - High; Medium - High; Low - Medium	NM_002152	Homo sapiens testis-specific transcript, Y-linked 10 (non-protein coding) (TTTY10), non-coding RNA [NR_001542]
384	XLOC_005731	NA		3.4339	0.039462	1.4038	0.50196	Low - High; High - Medium; Low - Medium		BROAD Institute lincRNA (XLOC_004375), lincRNA [TCONS_00009956]
385	XLOC_000762	NA		3.4326	0.039508	1.4033	0.50196	Low - High; High - Medium; Low - Medium	AA773613	Uncharacterized proteincDNA FLJ40032 fis, clone STOMA2009256 [Source:UniProtKB/TrEMBL;Acc:Q8N843] [ENST00000358234]
386	XLOC_l2_010239	NA		3.4286	0.039645	1.4018	0.50196	Low - High; High - Medium; Low - Medium		Homo sapiens nitrogen permease regulator-like 3 (S. cerevisiae) (NPRL3), transcript variant 2, mRNA [NM_001039476]
387	LOC100507948	NA		3.4284	0.039653	1.4017	0.50196	Low - High; Medium - High; Low - Medium	XR_112445	Homo sapiens solute carrier family 26, member 8 (SLC26A8), transcript variant 1, mRNA [NM_052961]
388	FXYD5		53827	3.425	0.039774	1.4004	0.50196	Low - High; High - Medium; Low - Medium		PREDICTED: Homo sapiens hypothetical LOC100128262 (LOC100128262), miscRNA [XR_110396]
389	ERAP1		51752	3.4185	0.040004	1.3979	0.50196	High - Low; High - Medium; Medium - Low	NM_001040458	BROAD Institute lincRNA (XLOC_004710), lincRNA [TCONS_00010248]
390	BEST2		54831	3.4166	0.04007	1.3972	0.50196	Low - High; High - Medium; Low - Medium	NM_017682	Homo sapiens family with sequence similarity 129, member C (FAM129C), transcript variant 2, mRNA [NM_001098524]
391		NA		3.4163	0.040081	1.3971	0.50196	Low - High; Medium - High; Low - Medium		Homo sapiens cDNA FLJ45121 fis, clone BRAWH3036077. [AK127064]
392	LYG2		254773	3.4148	0.040135	1.3965	0.50196	Low - High; High - Medium; Low - Medium	AF323919	Homo sapiens CDC42 effector protein (Rho GTPase binding) 1 (CDC42EP1), mRNA [NM_152243]

393	CCL3	6348	3.4129	0.040204	1.3957	0.50196	Low - High; High - Medium; Low - Medium	NM_002983	Homo sapiens proline rich 5 (renal) (PRR5), transcript variant 2, mRNA [NM_015366]
394	GABRA3	2556	3.4116	0.040249	1.3952	0.50196	Low - High; Medium - High; Low - Medium	NM_000808	BROAD Institute lincRNA (XLOC_013981), lincRNA [TCONS_00029251]
395	LEPREL2	10536	3.4115	0.040253	1.3952	0.50196	High - Low; High - Medium; Low - Medium	NM_014262	BROAD Institute lincRNA (XLOC_l2_002886), lincRNA [TCONS_l2_00005503]
396	LOC100505874	NA	3.4093	0.040333	1.3943	0.50196	High - Low; High - Medium; Medium - Low	XR_110950	BROAD Institute lincRNA (XLOC_003839), lincRNA [TCONS_00008387]
397	LOC283688	NA	3.4074	0.040398	1.3936	0.50196	Low - High; High - Medium; Low - Medium	NR_033833	PREDICTED: Homo sapiens protein FAM27E2-like (LOC100287633), mRNA [XM_002342728]
398	TERC	7012	3.4033	0.040548	1.392	0.50196	Low - High; Medium - High; Low - Medium	NR_001566	Homo sapiens tachykinin receptor 2 (TACR2), mRNA [NM_001057]
399	LOC497256	497256	3.4013	0.040618	1.3913	0.50196	Low - High; High - Medium; Low - Medium	AK094988	Homo sapiens microtubule-associated protein 7 (MAP7), transcript variant 4, mRNA [NM_003980]
400		NA	3.3965	0.040795	1.3894	0.50196	High - Low; High - Medium; Medium - Low	AF058072	Homo sapiens MAP7 domain containing 2 (MAP7D2), transcript variant 2, mRNA [NM_152780]
401	OR6T1	219874	3.3943	0.040872	1.3886	0.50196	High - Low; High - Medium; Medium - Low	NM_001005187	Homo sapiens uncharacterized LOC92659 (LOC92659), non-coding RNA [NR_015454]
402	C5AR1	728	3.3921	0.040954	1.3877	0.50196	Low - High; Medium - High; Low - Medium	NM_001736	RST4038 Athersys RAGE Library Homo sapiens cDNA, mRNA sequence [BG185100]
403	FOXH1	8928	3.3916	0.040972	1.3875	0.50196	Low - High; Medium - High; Low - Medium	NM_003923	
404	XLOC_001370	NA	3.3838	0.041256	1.3845	0.50196	Low - High; Medium - High; Low - Medium		Homo sapiens SH2 domain containing 2A (SH2D2A), transcript variant 5, mRNA [NM_001161444]
405	GSPT1	2935	3.3815	0.041339	1.3836	0.50196	Low - High; Medium - High; Medium - Low	NM_002094	Homo sapiens synaptogyrin 1 (SYNGR1), transcript variant 1a, mRNA [NM_004711]

406	MIAT	NA		3.3812	0.041351	1.3835	0.50196	Low - High; Medium - High; Low - Medium	NR_003491	Homo sapiens E2F transcription factor 2 (E2F2), mRNA [NM_004091]
407	XLOC_003650	NA		3.3805	0.041379	1.3832	0.50196	Low - High; High - Medium; Low - Medium	CR742343	Homo sapiens arginine-glutamic acid dipeptide (RE) repeats pseudogene 3 (REREP3), non-coding RNA [NR_033735]
408	SERP2		387923	3.3805	0.041379	1.3832	0.50196	Low - High; High - Medium; Low - Medium	NM_001010897	Homo sapiens obscurin, cytoskeletal calmodulin and titin-interacting RhoGEF (OBSCN), transcript variant 1, mRNA [NM_052843]
409	OSM		5008	3.379	0.041432	1.3827	0.50196	Low - High; Medium - High; Low - Medium	NM_020530	
410	LOC100170939		100170939	3.3708	0.041735	1.3795	0.50196	Low - High; Medium - High; Low - Medium	NR_024054	Homo sapiens acetyl-CoA carboxylase beta (ACACB), mRNA [NM_001093]
411	CD24		100133941	3.366	0.041915	1.3776	0.50196	High - Low; Medium - High; Medium - Low	NM_013230	Synthetic construct DNA, clone: pF1KE0822, Homo sapiens OLFR1031 gene for olfactory receptor 1031, without stop codon, in Flexi system. [AB529253]
412	UBL4B		164153	3.359	0.042176	1.3749	0.50196	Low - High; Medium - High; Low - Medium	NM_203412	Homo sapiens cystatin SA (CST2), mRNA [NM_001322]
413	RNF24		11237	3.355	0.042325	1.3734	0.50196	High - Low; High - Medium; Medium - Low	NM_007219	Q6P2F0_HUMAN (Q6P2F0) UEV3 protein, partial (39%) [THC2788642]
414	NFE4		58160	3.3464	0.04265	1.3701	0.50196	High - Low; High - Medium; Medium - Low	NM_001085386	Synthetic construct Homo sapiens gateway clone IMAGE:100018551 3' read RAB35 mRNA. [CU677518]
415	LOC100129387		100129387	3.3464	0.042651	1.3701	0.50196	Low - High; Medium - High; Medium - Low	NR_024490	Homo sapiens uncharacterized LOC100128252 (LOC100128252), transcript variant 2, non-coding RNA [NR_036522]
416	TRIM10		10107	3.3459	0.042671	1.3699	0.50196	Low - High; Medium - High; Medium - Low	NM_052828	Homo sapiens synaptonemal complex central element protein 3 (SYCE3), mRNA [NM_001123225]

417	LOC100129826		100129826	3.3434	0.042764	1.3689	0.50196	Low - High; High - Medium; Low - Medium	AK126915	Homo sapiens chemokine (C-C motif) ligand 3 (CCL3), mRNA [NM_002983]
418	IGFBP2		3485	3.3426	0.042795	1.3686	0.50196	High - Low; High - Medium; Low - Medium	NM_000597	Human MEN1 region clone epsilon/beta mRNA, 3' fragment. [AF001893]
419	DDX17		10521	3.3419	0.04282	1.3683	0.50196	Low - High; Medium - High; Low - Medium	NM_006386	Homo sapiens guanylate binding protein 3 (GBP3), mRNA [NM_018284]
420	LRRN3		54674	3.3401	0.042892	1.3676	0.50196	Low - High; Medium - High; Low - Medium	NM_018334	Homo sapiens keratin associated protein 4-12 (KRTAP4-12), mRNA [NM_031854]
421	CBX7		23492	3.3357	0.04306	1.3659	0.50196	Low - High; High - Medium; Low - Medium	NM_175709	BROAD Institute lincRNA (XLOC_012571), lincRNA [TCONS_00025761]
422	Q6LRY1	NA		3.3347	0.043096	1.3656	0.50196	Low - High; High - Medium; Low - Medium		Homo sapiens transmembrane protein 176B (TMEM176B), transcript variant 1, mRNA [NM_014020]
423	XLOC_007370	NA		3.3344	0.04311	1.3654	0.50196	High - Low; High - Medium; Medium - Low	BG187845	Homo sapiens major histocompatibility complex, class II, DR beta 6 (pseudogene) (HLA-DRB6), non-coding RNA [NR_001298]
424	DNAL4		10126	3.3343	0.043113	1.3654	0.50196	Low - High; High - Medium; Low - Medium	NM_005740	Homo sapiens zinc finger protein 839 pseudogene (LOC389906), non-coding RNA [NR_034031]
425	TTTY10		246119	3.3335	0.043145	1.3651	0.50196	Low - High; Medium - High; Medium - Low	NR_001542	BROAD Institute lincRNA (XLOC_007868), lincRNA [TCONS_00016491]
426	ADAM12		8038	3.3322	0.043195	1.3646	0.50196	Low - High; High - Medium; Low - Medium	NM_021641	Homo sapiens heme binding protein 1 (HEBP1), mRNA [NM_015987]
427	NYX		60506	3.3321	0.043198	1.3645	0.50196	Low - High; Medium - High; Low - Medium	NM_022567	Homo sapiens triggering receptor expressed on myeloid cells-like 1 (TREML1), mRNA [NM_178174]
428	FLJ42709	NA		3.3317	0.043211	1.3644	0.50196	Low - High; Medium - High; Low - Medium	NR_021490	Homo sapiens kin of IRRE like (Drosophila) (KIRREL), mRNA [NM_018240]

429	GYPC		2995	3.3313	0.043228	1.3642	0.50196	Low - High; Medium - High; Medium - Low	NM_002101	BROAD Institute lincRNA (XLOC_009000), lincRNA [TCONS_00018649]
430	SLC6A8		6535	3.3309	0.043244	1.3641	0.50196	Low - High; Medium - High; Medium - Low	NM_005629	Homo sapiens POU class 4 homeobox 2 (POU4F2), mRNA [NM_004575]
431	FAM182B	NA		3.3255	0.043452	1.362	0.50196	Low - High; High - Medium; Low - Medium	BX648489	Homo sapiens long intergenic non-protein coding RNA 482 (LINC00482), non-coding RNA [NR_038080]
432	ARHGEF10		9639	3.3202	0.043658	1.3599	0.50196	Low - High; High - Medium; Low - Medium	BC040474	BROAD Institute lincRNA (XLOC_004093), lincRNA [TCONS_00008588]
433	FLJ39051	NA		3.32	0.043665	1.3599	0.50196	High - Low; High - Medium; Medium - Low	NR_033839	
434	TPST1		8460	3.3197	0.043679	1.3597	0.50196	High - Low; High - Medium; Medium - Low	NM_003596	Homo sapiens GDNF family receptor alpha 1 (GFRA1), transcript variant 1, mRNA [NM_005264]
435	DUX4L4		441056	3.3177	0.043754	1.359	0.50196	Low - High; Medium - High; Low - Medium	NM_001177376	PREDICTED: Homo sapiens hypothetical LOC100506965 (LOC100506965), miscRNA [XR_110300]
436	LYRM2		57226	3.313	0.043938	1.3572	0.50196	High - Low; Medium - High; Medium - Low	NM_020466	ab56g09.s1 Stratagene lung carcinoma 937218 Homo sapiens cDNA clone IMAGE:844864 3', mRNA sequence [AA773613]
437	XLOC_012831	NA		3.3124	0.04396	1.3569	0.50196	Low - High; Medium - High; Low - Medium	BC051727	Homo sapiens cDNA FLJ59054 complete cds, highly similar to D(1B) dopamine receptor. [AK301306]
438	LINC00260		84719	3.3119	0.04398	1.3567	0.50196	Low - High; High - Medium; Low - Medium	BC000988	PREDICTED: Homo sapiens family with sequence similarity 27, member E3 (FAM27E3), mRNA [XM_001720463]
439	XLOC_013557	NA		3.29	0.044848	1.3483	0.50762	Low - High; Medium - High; Low - Medium		Homo sapiens DEAD (Asp-Glu-Ala-Asp) box polypeptide 43 (DDX43), mRNA [NM_018665]

440	IRF2BPL		64207	3.2857	0.04502	1.3466	0.50762	Low - High; Medium - High; Low - Medium	NM_024496	Homo sapiens casein kinase 1, gamma 2 (CSNK1G2), mRNA [NM_001319]
441	XLOC_l2_007184	NA		3.2852	0.04504	1.3464	0.50762	Low - High; Medium - High; Low - Medium	AK093290	Homo sapiens membrane protein, palmitoylated 1, 55kDa (MPP1), transcript variant 3, mRNA [NM_001166461]
442	EPB49		2039	3.2829	0.045134	1.3455	0.50762	Low - High; Medium - High; Medium - Low	NM_001978	BROAD Institute lincRNA (XLOC_004188), lincRNA [TCONS_00008670]
443	SMR3A		26952	3.2807	0.045222	1.3446	0.50762	Low - High; Medium - High; Low - Medium	NM_012390	Homo sapiens progestin and adipoQ receptor family member IX (PAQR9), mRNA [NM_198504]
444		NA		3.2806	0.045226	1.3446	0.50762	Low - High; High - Medium; Low - Medium		PREDICTED: Homo sapiens hypothetical LOC100506546, transcript variant 2 (LOC100506546), miscRNA [XR_108325]
445	XLOC_011361	NA		3.2731	0.04553	1.3417	0.50762	Low - High; High - Medium; Low - Medium		Homo sapiens olfactory receptor, family 8, subfamily G, member 2 (OR8G2), mRNA [NM_001007249]
446	LOC642947		642947	3.2713	0.045601	1.341	0.50762	Low - High; High - Medium; Low - Medium	BC132887	Homo sapiens KN motif and ankyrin repeat domains 2 (KANK2), transcript variant 1, mRNA [NM_015493]
447	HIST1H4J		8363	3.2677	0.045749	1.3396	0.50762	Low - High; Medium - High; Low - Medium	NM_021968	PREDICTED: Homo sapiens double homeobox protein 4-like protein 4-like (LOC652119), mRNA [XM_001720798]
448	XLOC_l2_001085	NA		3.2633	0.04593	1.3379	0.50762	Low - High; Medium - High; Medium - Low	AK090412	BROAD Institute lincRNA (XLOC_l2_001421), lincRNA [TCONS_l2_00002836]
449	SLC26A11		284129	3.2627	0.045951	1.3377	0.50762	Low - High; High - Medium; Low - Medium	NM_001166347	BX096650 Soares_testis_NHT Homo sapiens cDNA clone IMAGp998C184111, mRNA sequence [BX096650]

450	LOC400682		400682	3.2625	0.045963	1.3376	0.50762	High - Low; High - Medium; Medium - Low	XM_003118492	BROAD Institute lincRNA (XLOC_009255), lincRNA [TCONS_00019107]
451	CELA2B		51032	3.2615	0.046002	1.3372	0.50762	Low - High; Medium - High; Low - Medium	NM_015849	
452	GOLGA8A		23015	3.2611	0.046019	1.3371	0.50762	Low - High; Medium - High; Low - Medium	NM_181077	Homo sapiens carcinoembryonic antigen-related cell adhesion molecule 18 (CEACAM18), mRNA [NM_001080405]
453	C14orf45		80127	3.2515	0.046416	1.3333	0.50762	Low - High; Medium - High; Low - Medium	NM_025057	Homo sapiens phosphodiesterase 2A, cGMP-stimulated (PDE2A), transcript variant 1, mRNA [NM_002599]
454	SCARA3		51435	3.2436	0.046744	1.3303	0.50762	Low - High; High - Medium; Low - Medium	NM_016240	Homo sapiens dachshund homolog 1 (Drosophila) (DACH1), transcript variant 1, mRNA [NM_080759]
455	FLJ43681		388574	3.2433	0.046757	1.3302	0.50762	High - Low; High - Medium; Medium - Low	NR_029406	UI-H-BW1-amz-e-06-0-UI.s2 NCI_CGAP_Sub7 Homo sapiens cDNA clone IMAGE:3071699 3', mRNA sequence [BF507452]
456	TAOK2		9344	3.243	0.046768	1.33	0.50762	Low - High; High - Medium; Low - Medium	NM_016151	Homo sapiens haptoglobin-related protein (HPR), mRNA [NM_020995]
457	ZDHHC23		254887	3.2426	0.046786	1.3299	0.50762	Low - High; Medium - High; Low - Medium	NM_173570	Homo sapiens CD24 molecule (CD24), mRNA [NM_013230]
458	CRABP1		1381	3.2378	0.046984	1.3281	0.50762	Low - High; Medium - High; Low - Medium	NM_004378	Homo sapiens tensin 1 (TNS1), mRNA [NM_022648]
459	PGM5		5239	3.2334	0.04717	1.3263	0.50762	High - Low; High - Medium; Medium - Low	NM_021965	Homo sapiens peptidase inhibitor 16 (PI16), transcript variant 1, mRNA [NM_153370]
460	BPI		671	3.2148	0.047963	1.3191	0.50762	High - Low; High - Medium; Medium - Low	NM_001725	Homo sapiens eyes absent homolog 1 (Drosophila) (EYA1), transcript variant 3, mRNA [NM_000503]
461	REEP1		65055	3.2095	0.048187	1.3171	0.50762	Low - High; High - Medium; Low - Medium	NM_022912	D80009 Start codon is not identified {Homo sapiens} (exp=-1; wgp=0; cg=0), partial (12%) [THC2480131]

462	LOC100652730	NA		3.2095	0.048188	1.3171	0.50762	Low - High; Medium - High; Low - Medium	XR_132670	ALG1_PONPY (Q5R7A2) Chitobiosyldiphosphodolichol beta-mannosyltransferase (GDP-mannose-dolichol diphosphochitobiose mannosyltransferase) (GDP-Man:GlcNAc2-PP-dolichol mannosyltransferase) (Beta-1,4-mannosyltransferase) , partial (14%) [THC2542402]
463		NA		3.2086	0.048227	1.3167	0.50762	High - Low; Medium - High; Medium - Low	BC033643	XM_797160 actin {Trypanosoma cruzi strain CL Brener} (exp=-1; wgp=0; cg=0), partial (9%) [THC2707351]
464	FBRS		64319	3.2078	0.048263	1.3164	0.50762	Low - High; Medium - High; Low - Medium	NM_001105079	Q6CC74_YARLI (Q6CC74) Similarity, partial (3%) [THC2487722]
465	NCRNA00083	NA		3.2077	0.048266	1.3164	0.50762	Low - High; Medium - High; Low - Medium	XR_108314	Homo sapiens homeobox A9 (HOXA9), mRNA [NM_152739]
466	PER1		5187	3.2074	0.048281	1.3162	0.50762	Low - High; High - Medium; Low - Medium	NM_002616	Homo sapiens submaxillary gland androgen regulated protein 3A (SMR3A), mRNA [NM_012390]
467	GBP3		2635	3.2058	0.048351	1.3156	0.50762	High - Low; High - Medium; Medium - Low	NM_018284	602506627F1 NIH_MGC_79 Homo sapiens cDNA clone IMAGE:4603783 5', mRNA sequence [BG434565]
468	LOC401109	NA		3.2035	0.048447	1.3147	0.50762	Low - High; Medium - High; Low - Medium	NR_034088	HCG1981372, isoform CRA_cNovel proteinUncharacterized protein [Source:UniProtKB/TrEMBL;Acc:B1B108] [ENST00000471090]
469	ADCY4		196883	3.1973	0.048718	1.3123	0.50762	Low - High; Medium - High; Low - Medium	NM_001198568	Homo sapiens mRNA; cDNA DKFZp586J1922 (from clone DKFZp586J1922) [AL110203]
470		NA		3.1966	0.04875	1.312	0.50762	Low - High; High - Medium; Low - Medium	AK021933	

471	UCKL1		54963	3.1944	0.048845	1.3112	0.50762	Low - High; Medium - High; Low - Medium	NM_017859	Homo sapiens monoglyceride lipase (MGLL), transcript variant 1, mRNA [NM_007283]
472	XLOC_l2_004706	NA		3.189	0.049081	1.3091	0.50762	Low - High; Medium - High; Low - Medium		Homo sapiens BEN domain containing 7 (BEND7), transcript variant 1, mRNA [NM_152751]
473	ANKRD20A2		441430	3.1854	0.04924	1.3077	0.50762	Low - High; Medium - High; Medium - Low	NM_001012421	BROAD Institute lincRNA (XLOC_000884), lincRNA [TCONS_00000554]
474	STAG3L2		442582	3.1849	0.049261	1.3075	0.50762	Low - High; Medium - High; Low - Medium	NR_040584	Homo sapiens lactotransferrin (LTF), transcript variant 1, mRNA [NM_002343]
475	LOC653075		653075	3.1823	0.049376	1.3065	0.50762	Low - High; Medium - High; Low - Medium	NR_033933	Homo sapiens CKLF-like MARVEL transmembrane domain containing 5 (CMTM5), transcript variant 3, mRNA [NM_001037288]
476	ACTL7A		10881	3.1809	0.049438	1.3059	0.50762	Low - High; Medium - High; Low - Medium	NM_006687	Homo sapiens chemokine (C-C motif) ligand 2 (CCL2), mRNA [NM_002982]
477	HGFAC		3083	3.1782	0.049557	1.3049	0.50762	Low - High; High - Medium; Low - Medium	NM_001528	Homo sapiens kringle containing transmembrane protein 1 (KREMEN1), transcript variant 2, mRNA [NM_032045]
478		NA		3.1771	0.049604	1.3045	0.50762	Low - High; High - Medium; Low - Medium	XR_109647	BROAD Institute lincRNA (XLOC_006277), lincRNA [TCONS_00013611]
479	LOC100133286		100133286	3.1761	0.04965	1.3041	0.50762	Low - High; Medium - High; Low - Medium	NR_040084	Homo sapiens uncharacterized LOC100190939 (LOC100190939), non-coding RNA [NR_024458]
480	LOC100129069		100129069	3.1747	0.049711	1.3036	0.50762	Low - High; High - Medium; Low - Medium	AK127359	BROAD Institute lincRNA (XLOC_013744), lincRNA [TCONS_00028390]
481	LOC100288594	NA		3.1745	0.04972	1.3035	0.50762	High - Low; Medium - High; Medium - Low	XR_108779	immunoglobulin heavy constant epsilon [Source:HGNC Symbol;Acc:5522] [ENST00000390541]

482	XLOC_001496	NA	3.1735	0.049764	1.3031	0.50762	High - Low; High - Medium; Medium - Low		Homo sapiens chemokine (C-C motif) receptor 2 (CCR2), transcript variant A, mRNA [NM_001123041]
483	IL17REL	400935	3.1729	0.049792	1.3028	0.50762	Low - High; Medium - High; Low - Medium	NM_001001694	BROAD Institute lincRNA (XLOC_008079), lincRNA [TCONS_00017468]
484	SEPT7P2	NA	3.1721	0.04983	1.3025	0.50762	Low - High; High - Medium; Low - Medium	AK056032	Homo sapiens leucine-rich alpha-2-glycoprotein 1 (LRG1), mRNA [NM_052972]
485	ZAN	7455	3.1705	0.049899	1.3019	0.50762	Low - High; Medium - High; Low - Medium	NM_173059	DA569430 HEMBA1 Homo sapiens cDNA clone HEMBA1002792 5', mRNA sequence [DA569430]
486	BPIFB2	80341	3.1702	0.049913	1.3018	0.50762	Low - High; Medium - High; Low - Medium	NM_025227	Homo sapiens uncharacterized LOC100128252 (LOC100128252), transcript variant 2, non-coding RNA [NR_036522]
487	C9orf29	652972	3.1699	0.049924	1.3017	0.50762	Low - High; Medium - High; Low - Medium	NR_034087	Homo sapiens chromosome 9 open reading frame 29 (C9orf29), non-coding RNA [NR_034087]

Total in Data 1	232
Total in Data 2	487
Unique in Data 1	232
Unique in Data 2	422
Common in both	6
