# Supplementary material for: Association between Arsenic Level, Gene Expression in Asian Population, and In Vitro Carcinogenic Bladder Tumor
Source: Oxid Med Cell Longev. 2022 Jan 7;2022:3459855. doi: 10.1155/2022/3459855 (PMC8760535; doi:10.1155/2022/3459855)
Supplement: Supplementary 8 — Table S7: significant pathways in Data1 (low and high concentrations) and Data2 (low, medium, and high concentrations) and the overlap between two (highlighted in pink). [file 3459855.f8.pdf]

Table S7. Significant pathways in Data1 (low, high concentrations) and Data2(low, medium, high concentrations), and the overlap between two (highlighted in pink).

	Data1			
**S.No.**	**Ingenuity Canonical Pathways**	**-log(p-value)**	**Ratio**	**Molecules**
1	Neuropathic Pain Signaling In Dorsal Horn Neurons	3.39E+00	5.94E-02	GRIA4,ITPR1,PIK3C2B,PIK3R1,PLCG1,PRKCE
2	Virus Entry via Endocytic Pathways	3.25E+00	5.61E-02	FLNB,ITGB3,PIK3C2B,PIK3R1,PLCG1,PRKCE
3	Macropinocytosis Signaling	3.10E+00	6.58E-02	ITGB3,PIK3C2B,PIK3R1,PLCG1,PRKCE
4	GP6 Signaling Pathway	3.01E+00	5.04E-02	FCER1G,ITGB3,ITPR1,PIK3C2B,PIK3R1,PRKCE
5	CD28 Signaling in T Helper Cells	2.99E+00	5.00E-02	CARD11,FCER1G,ITPR1,PIK3C2B,PIK3R1,PLCG1
6	NF-κB Activation by Viruses	2.95E+00	6.10E-02	ELP1,ITGB3,PIK3C2B,PIK3R1,PRKCE
7	HER-2 Signaling in Breast Cancer	2.91E+00	5.95E-02	ITGB3,PIK3C2B,PIK3R1,PLCG1,PRKCE
8	p70S6K Signaling	2.83E+00	4.65E-02	EEF2K,IL4R,PIK3C2B,PIK3R1,PLCG1,PRKCE
9	EGF Signaling	2.73E+00	7.27E-02	ITPR1,PIK3C2B,PIK3R1,PLCG1
10	Iron homeostasis signaling pathway	2.70E+00	4.38E-02	ATP6V0B,ATP6V0D1,ATP6V1F,CD163,SKP1,SMAD5
11	Apelin Cardiomyocyte Signaling Pathway	2.59E+00	5.05E-02	ITPR1,PIK3C2B,PIK3R1,PLCG1,PRKCE
12	Gap Junction Signaling	2.53E+00	3.54E-02	GRIA4,ITPR1,PIK3C2B,PIK3R1,PLCG1,PRKCE,TUBA1B
13	Thrombopoietin Signaling	2.52E+00	6.35E-02	PIK3C2B,PIK3R1,PLCG1,PRKCE
14	Calcium-induced T Lymphocyte Apoptosis	2.44E+00	6.06E-02	FCER1G,ITPR1,PLCG1,PRKCE
15	ErbB4 Signaling	2.42E+00	5.97E-02	PIK3C2B,PIK3R1,PLCG1,PRKCE
16	iCOS-iCOSL Signaling in T Helper Cells	2.38E+00	4.50E-02	FCER1G,ITPR1,PIK3C2B,PIK3R1,PLCG1
17	eNOS Signaling	2.38E+00	3.77E-02	ITPR1,LPAR4,PIK3C2B,PIK3R1,PLCG1,PRKCE
18	Growth Hormone Signaling	2.33E+00	5.63E-02	PIK3C2B,PIK3R1,PLCG1,PRKCE
19	Non-Small Cell Lung Cancer Signaling	2.29E+00	5.48E-02	ITPR1,PIK3C2B,PIK3R1,PLCG1
20	Fc Epsilon RI Signaling	2.28E+00	4.27E-02	FCER1G,PIK3C2B,PIK3R1,PLCG1,PRKCE
21	Renin-Angiotensin Signaling	2.27E+00	4.24E-02	ITPR1,PIK3C2B,PIK3R1,PLCG1,PRKCE
22	Glioma Invasiveness Signaling	2.27E+00	5.41E-02	ITGB3,PIK3C2B,PIK3R1,TIMP1
23	Erythropoietin Signaling	2.22E+00	5.26E-02	PIK3C2B,PIK3R1,PLCG1,PRKCE
24	GDNF Family Ligand-Receptor Interactions	2.22E+00	5.26E-02	ITPR1,PIK3C2B,PIK3R1,PLCG1
25	CCR3 Signaling in Eosinophils	2.18E+00	4.03E-02	CCR3,ITPR1,PIK3C2B,PIK3R1,PRKCE
26	Phagosome Formation	2.16E+00	4.00E-02	FCER1G,PIK3C2B,PIK3R1,PLCG1,PRKCE
27	14-3-3-mediated Signaling	2.13E+00	3.94E-02	PIK3C2B,PIK3R1,PLCG1,PRKCE,TUBA1B
28	P2Y Purigenic Receptor Signaling Pathway	2.13E+00	3.94E-02	ITGB3,PIK3C2B,PIK3R1,PLCG1,PRKCE
29	Prolactin Signaling	2.13E+00	4.94E-02	PIK3C2B,PIK3R1,PLCG1,PRKCE
30	Glycogen Degradation III	2.12E+00	1.43E-01	AGL,GAA
31	Role of NFAT in Regulation of the Immune Response	2.11E+00	3.31E-02	ELP1,FCER1G,ITPR1,PIK3C2B,PIK3R1,PLCG1
32	VEGF Family Ligand-Receptor Interactions	2.07E+00	4.76E-02	PIK3C2B,PIK3R1,PLCG1,PRKCE
33	FGF Signaling	2.07E+00	4.76E-02	ITPR1,PIK3C2B,PIK3R1,PLCG1
34	Insulin Secretion Signaling Pathway	2.06E+00	2.88E-02	EIF2S1,INSR,ITPR1,PIK3C2B,PIK3R1,PLCG1,PRKCE
35	Adipogenesis pathway	2.04E+00	3.73E-02	CDK5,DGKD,KAT2A,RBBP4,SMAD5
36	Endothelin-1 Signaling	2.03E+00	3.19E-02	ITPR1,PIK3C2B,PIK3R1,PLCG1,PNPLA8,PRKCE
37	Th2 Pathway	2.01E+00	3.68E-02	ACVR1C,CCR3,IL4R,PIK3C2B,PIK3R1
38	Synaptogenesis Signaling Pathway	1.99E+00	2.56E-02	CDK5,EFNB2,GRIA4,ITPR1,PIK3C2B,PIK3R1,PLCG1,PRKCE

Total in Data 1	112
Total in Data 2	45
Common in both	14
Unique in Data 1	98
Unique in Data 2	31

39	CTLA4 Signaling in Cytotoxic T Lymphocytes	1.99E+00	4.49E-02	FCER1G,PIK3C2B,PIK3R1,PLCG1
40	Leukocyte Extravasation Signaling	1.94E+00	3.05E-02	ITGB3,PIK3C2B,PIK3R1,PLCG1,PRKCE,TIMP1
41	Amyloid Processing	1.92E+00	6.00E-02	BACE2,CDK5,PRKCE
42	ErbB Signaling	1.91E+00	4.26E-02	PIK3C2B,PIK3R1,PLCG1,PRKCE
43	Neuregulin Signaling	1.88E+00	4.17E-02	CDK5,PIK3R1,PLCG1,PRKCE
44	UVB-Induced MAPK Signaling	1.88E+00	5.77E-02	PIK3C2B,PIK3R1,PRKCE
45	Lymphotoxin β Receptor Signaling	1.85E+00	5.66E-02	ELP1,PIK3C2B,PIK3R1
46	Factors Promoting Cardiogenesis in Vertebrates	1.84E+00	3.33E-02	ACVR1C,PLCG1,PRKCE,SMAD5,WNT10A
47	CREB Signaling in Neurons	1.84E+00	2.90E-02	GRIA4,ITPR1,PIK3C2B,PIK3R1,PLCG1,PRKCE
48	Nitric Oxide Signaling in the Cardiovascular System	1.83E+00	4.04E-02	ITPR1,PIK3C2B,PIK3R1,PRKCE
49	VEGF Signaling	1.83E+00	4.04E-02	EIF2S1,PIK3C2B,PIK3R1,PLCG1
50	Phagosome Maturation	1.83E+00	3.31E-02	ATP6V0B,ATP6V0D1,ATP6V1F,CTSS,TUBA1B
51	Gustation Pathway	1.80E+00	3.25E-02	ITPR1,TAS2R10,TAS2R14,TAS2R19,TAS2R20
52	PKCθ Signaling in T Lymphocytes	1.79E+00	3.23E-02	CARD11,FCER1G,PIK3C2B,PIK3R1,PLCG1
53	AMPK Signaling	1.78E+00	2.80E-02	EEF2K,HLTF,INSR,PIK3C2B,PIK3R1,RAB8A
54	Aldosterone Signaling in Epithelial Cells	1.75E+00	3.16E-02	ITPR1,PIK3C2B,PIK3R1,PLCG1,PRKCE
55	Gαq Signaling	1.75E+00	3.16E-02	ITPR1,PIK3C2B,PIK3R1,PLCG1,PRKCE
56	T Cell Receptor Signaling	1.74E+00	3.81E-02	CARD11,PIK3C2B,PIK3R1,PLCG1
57	HOTAIR Regulatory Pathway	1.73E+00	3.12E-02	DZIP3,PIK3C2B,PIK3R1,RBBP4,WNT10A
58	Sulfate Activation for Sulfonation	1.72E+00	5.00E-01	PAPSS2
59	Dopamine-DARPP32 Feedback in cAMP Signaling	1.70E+00	3.07E-02	CDK5,ITPR1,KCNJ5,PLCG1,PRKCE
60	EIF2 Signaling	1.69E+00	2.68E-02	EIF2S1,INSR,PIK3C2B,PIK3R1,RPL8,RPS27
61	Tec Kinase Signaling	1.69E+00	3.05E-02	FCER1G,PIK3C2B,PIK3R1,PLCG1,PRKCE
62	Glioma Signaling	1.68E+00	3.64E-02	PIK3C2B,PIK3R1,PLCG1,PRKCE
63	Glioblastoma Multiforme Signaling	1.68E+00	3.03E-02	ITPR1,PIK3C2B,PIK3R1,PLCG1,WNT10A
64	HGF Signaling	1.67E+00	3.60E-02	PIK3C2B,PIK3R1,PLCG1,PRKCE
65	Th1 and Th2 Activation Pathway	1.62E+00	2.92E-02	ACVR1C,CCR3,IL4R,PIK3C2B,PIK3R1
66	CD40 Signaling	1.62E+00	4.62E-02	ELP1,PIK3C2B,PIK3R1
67	Neuroinflammation Signaling Pathway	1.61E+00	2.34E-02	ACVR1C,BACE2,IDE,KCNJ5,PIK3C2B,PIK3R1,PLCG1
68	fMLP Signaling in Neutrophils	1.60E+00	3.45E-02	ITPR1,PIK3C2B,PIK3R1,PRKCE
69	Hepatic Fibrosis Signaling Pathway	1.60E+00	2.17E-02	ACVR1C,ITGB3,PIK3C2B,PIK3R1,PLCG1,PRKCE,TIMP1,WNT10A
70	Role of Tissue Factor in Cancer	1.59E+00	3.42E-02	ITGB3,P4HB,PIK3C2B,PIK3R1
71	Huntington's Disease Signaling	1.58E+00	2.53E-02	CDK5,HIP1,ITPR1,PIK3C2B,PIK3R1,PRKCE
72	T Cell Exhaustion Signaling Pathway	1.58E+00	2.86E-02	ACVR1C,FCER1G,PIK3C2B,PIK3R1,PLCG1
73	Role of NANOG in Mammalian Embryonic Stem Cell Pluripotency	1.57E+00	3.36E-02	PIK3C2B,PIK3R1,SMAD5,WNT10A
74	Role of JAK1 and JAK3 in γc Cytokine Signaling	1.55E+00	4.35E-02	IL4R,PIK3C2B,PIK3R1
75	SPINK1 General Cancer Pathway	1.55E+00	4.35E-02	MT1H,PIK3C2B,PIK3R1
76	Coenzyme A Biosynthesis	1.55E+00	3.33E-01	COASY
77	NF-κB Signaling	1.55E+00	2.79E-02	CARD11,FCER1G,INSR,PIK3C2B,PIK3R1
78	Small Cell Lung Cancer Signaling	1.52E+00	4.23E-02	MAX,PIK3C2B,PIK3R1
79	Heparan Sulfate Biosynthesis (Late Stages)	1.52E+00	4.23E-02	AARS1,CHST15,HS3ST1
80	Role of p14/p19ARF in Tumor Suppression	1.51E+00	6.90E-02	PIK3C2B,PIK3R1
81	Caveolar-mediated Endocytosis Signaling	1.49E+00	4.11E-02	FLNB,INSR,ITGB3
82	Leptin Signaling in Obesity	1.47E+00	4.05E-02	PIK3C2B,PIK3R1,PLCG1
83	Molecular Mechanisms of Cancer	1.47E+00	2.05E-02	ARHGEF18,CDK5,MAX,PIK3C2B,PIK3R1,PRKCE,SMAD5,WNT10A

84	Endocannabinoid Neuronal Synapse Pathway	1.47E+00	3.12E-02	GRIA4,ITPR1,KCNJ5,PLCG1
85	Synaptic Long Term Depression	1.46E+00	2.65E-02	GRIA4,ITPR1,PLCG1,PNPLA8,PRKCE
86	NRF2-mediated Oxidative Stress Response	1.46E+00	2.65E-02	DNAJA2,JUNB,PIK3C2B,PIK3R1,PRKCE
87	Angiopoietin Signaling	1.46E+00	4.00E-02	ELP1,PIK3C2B,PIK3R1
88	Synaptic Long Term Potentiation	1.46E+00	3.10E-02	GRIA4,ITPR1,PLCG1,PRKCE
89	Reelin Signaling in Neurons	1.46E+00	3.10E-02	CDK5,ITGB3,PIK3C2B,PIK3R1
90	G Protein Signaling Mediated by Tubby	1.46E+00	6.45E-02	INSR,PLCG1
91	ILK Signaling	1.45E+00	2.63E-02	FLNB,ITGB3,LIMS1,PIK3C2B,PIK3R1
92	Semaphorin Neuronal Repulsive Signaling Pathway	1.45E+00	3.08E-02	CDK5,PIK3C2B,PIK3R1,PLCG1
93	Neurotrophin/TRK Signaling	1.45E+00	3.95E-02	PIK3C2B,PIK3R1,PLCG1
94	RAR Activation	1.42E+00	2.58E-02	HLTF,PIK3R1,PRKCE,RDH13,SMAD5
95	Heparan Sulfate Biosynthesis	1.42E+00	3.85E-02	AARS1,CHST15,HS3ST1
96	IL-9 Signaling	1.41E+00	6.06E-02	PIK3C2B,PIK3R1
97	IL-3 Signaling	1.40E+00	3.80E-02	PIK3C2B,PIK3R1,PRKCE
98	Thyroid Cancer Signaling	1.40E+00	3.80E-02	INSR,PIK3C2B,PIK3R1
99	Human Embryonic Stem Cell Pluripotency	1.40E+00	2.96E-02	PIK3C2B,PIK3R1,SMAD5,WNT10A
100	Adrenomedullin signaling pathway	1.39E+00	2.54E-02	ITPR1,MAX,PIK3C2B,PIK3R1,PLCG1
101	IL-17 Signaling	1.39E+00	3.75E-02	PIK3C2B,PIK3R1,TIMP1
102	Axonal Guidance Signaling	1.37E+00	1.86E-02	CDK5,EFNB2,PIK3C2B,PIK3R1,PLCG1,PRKCE,SEMA4A,TUBA1B,WNT10A
103	PI3K Signaling in B Lymphocytes	1.37E+00	2.90E-02	IL4R,ITPR1,PIK3R1,PLCG1
104	LPS-stimulated MAPK Signaling	1.36E+00	3.66E-02	PIK3C2B,PIK3R1,PRKCE
105	2-ketoglutarate Dehydrogenase Complex	1.33E+00	2.00E-01	DLST
106	Serine Biosynthesis	1.33E+00	2.00E-01	PSPH
107	Myo-inositol Biosynthesis	1.33E+00	2.00E-01	IMPA2
108	Type II Diabetes Mellitus Signaling	1.33E+00	2.82E-02	INSR,PIK3C2B,PIK3R1,PRKCE
109	HIPPO signaling	1.32E+00	3.53E-02	PATJ,SKP1,SMAD5
110	IL-4 Signaling	1.32E+00	3.53E-02	IL4R,PIK3C2B,PIK3R1
111	PDGF Signaling	1.31E+00	3.49E-02	PIK3C2B,PIK3R1,PLCG1
112	Thrombin Signaling	1.31E+00	2.40E-02	ITPR1,PIK3C2B,PIK3R1,PLCG1,PRKCE
	Data2			
**S.No.**	**Ingenuity Canonical Pathways**	**-log(p-value)**	**Ratio**	**Molecules**
1	Osteoarthritis Pathway	3.49E+00	4.74E-02	ACVRL1,ALPL,CREB5,CXCL8,GDF5,MMP9,NAMPT,PGF,PTGS2,SPP1
2	ILK Signaling	3.20E+00	4.74E-02	CREB5,DSP,FOS,ITGB2,ITGB4,MMP9,PGF,PIK3R6,PTGS2
3	Atherosclerosis Signaling	2.97E+00	5.51E-02	CXCL8,ITGB2,MMP9,MSR1,ORM1,ORM2,TNFRSF12A
4	Acute Phase Response Signaling	2.74E+00	4.47E-02	C4BPA,CRABP1,FOS,HP,IRAK1,ORM1,ORM2,OSM
5	IL-8 Signaling	2.44E+00	4.00E-02	CXCL8,FOS,IRAK1,ITGB2,MMP9,PGF,PIK3R6,PTGS2
6	LXR/RXR Activation	2.39E+00	4.96E-02	HPR,MMP9,MSR1,ORM1,ORM2,PTGS2
7	Phagosome Formation	2.33E+00	4.80E-02	C5AR1,IGHG1,ITGB2,MSR1,PIK3R6,SCARA3
8	Role of Macrophages, Fibroblasts and Endothelial Cells in Rheumatoid Arthritis	2.24E+00	3.21E-02	C5AR1,CEBPE,CREB5,CXCL8,FOS,IGHG1,IRAK1,OSM,PGF,PIK3R6
9	Granulocyte Adhesion and Diapedesis	2.13E+00	3.89E-02	C5AR1,CCL3,CCL3L3,CXCL8,ITGB2,MMP19,MMP9
10	IGF-1 Signaling	2.02E+00	4.81E-02	FOS,GRB10,IGFBP2,PIK3R6,SFN
11	Agranulocyte Adhesion and Diapedesis	1.97E+00	3.63E-02	C5AR1,CCL3,CCL3L3,CXCL8,ITGB2,MMP19,MMP9
12	Complement System	1.97E+00	8.11E-02	C4BPA,C5AR1,ITGB2
13	Inhibition of Matrix Metalloproteases	1.91E+00	7.69E-02	ADAM12,MMP19,MMP9

14	Neuroinflammation Signaling Pathway	1.90E+00	3.01E-02	CCL3,CREB5,CXCL8,FOS,GABRA3,IRAK1,MMP9,PIK3R6,PTGS2
15	Caveolar-mediated Endocytosis Signaling	1.89E+00	5.48E-02	CAV1,FLOT2,ITGB2,ITGB4
16	Airway Pathology in Chronic Obstructive Pulmonary Disease	1.80E+00	4.24E-02	CXCL8,MMP9,ORM1,ORM2,OSM
17	Dermatan Sulfate Biosynthesis (Late Stages)	1.71E+00	6.52E-02	CHST12,HS3ST1,SULT1A2
18	GÎ±i Signaling	1.70E+00	4.00E-02	ADCY4,ADORA3,CAV1,CHRM4,RGS14
19	14-3-3-mediated Signaling	1.67E+00	3.94E-02	FOS,PIK3R6,SFN,TUBB2A,TUBB8
20	Chondroitin Sulfate Biosynthesis (Late Stages)	1.66E+00	6.25E-02	CHST12,HS3ST1,SULT1A2
21	Estrogen Receptor Signaling	1.66E+00	2.74E-02	ADCY4,CAV1,CREB5,FOS,MMP19,MMP9,PGF,PIK3R6,PRKDC
22	PDGF Signaling	1.65E+00	4.65E-02	CAV1,FOS,PIK3R6,SYNJ2
23	Hematopoiesis from Pluripotent Stem Cells	1.64E+00	6.12E-02	CXCL8,IGHG1,IGLC1
24	Role of IL-17A in Arthritis	1.53E+00	5.56E-02	CXCL8,PIK3R6,PTGS2
25	Communication between Innate and Adaptive Immune Cells	1.50E+00	4.17E-02	CCL3,CCL3L3,CXCL8,IGHG1
26	Chondroitin Sulfate Biosynthesis	1.49E+00	5.36E-02	CHST12,HS3ST1,SULT1A2
27	Bladder Cancer Signaling	1.49E+00	4.12E-02	CXCL8,MMP19,MMP9,PGF
28	Clathrin-mediated Endocytosis Signaling	1.47E+00	3.11E-02	ITGB2,ITGB4,ORM1,ORM2,PGF,PIK3R6
29	p53 Signaling	1.47E+00	4.08E-02	GADD45G,PIK3R6,PRKDC,SFN
30	VEGF Signaling	1.46E+00	4.04E-02	EIF1AY,PGF,PIK3R6,SFN
31	Methylglyoxal Degradation I	1.43E+00	3.33E-01	HAGH
32	Anandamide Degradation	1.43E+00	3.33E-01	FAAH
33	Dermatan Sulfate Biosynthesis	1.43E+00	5.08E-02	CHST12,HS3ST1,SULT1A2
34	Gap Junction Signaling	1.43E+00	3.03E-02	ADCY4,CAV1,CSNK1G2,PIK3R6,TUBB2A,TUBB8
35	IL-17A Signaling in Gastric Cells	1.42E+00	8.00E-02	CXCL8,FOS
36	Colorectal Cancer Metastasis Signaling	1.41E+00	2.77E-02	ADCY4,FOS,MMP19,MMP9,PGF,PIK3R6,PTGS2
37	Relaxin Signaling	1.40E+00	3.33E-02	ADCY4,FOS,MMP9,PIK3R6,RLN1
38	Coronavirus Pathogenesis Pathway	1.40E+00	3.33E-02	CXCL8,FOS,PTGS2,RPS4Y1,RPS4Y2
39	Hepatic Fibrosis Signaling Pathway	1.39E+00	2.45E-02	BAMBI,CCL3,CREB5,CXCL8,FOS,IRAK1,PGF,PIK3R6,SPP1
40	Thrombopoietin Signaling	1.36E+00	4.76E-02	FOS,PIK3R6,THPO
41	Virus Entry via Endocytic Pathways	1.35E+00	3.74E-02	CAV1,ITGB2,ITGB4,PIK3R6
42	Pancreatic Adenocarcinoma Signaling	1.33E+00	3.67E-02	MMP9,PGF,PIK3R6,PTGS2
43	CD40 Signaling	1.33E+00	4.62E-02	FOS,PIK3R6,PTGS2
44	eNOS Signaling	1.32E+00	3.14E-02	ADCY4,CAV1,CHRM4,PGF,PIK3R6
45	HOTAIR Regulatory Pathway	1.31E+00	3.12E-02	MMP19,MMP9,PIK3R6,RBM38,SPP1
