# Supplementary material for: Association between Arsenic Level, Gene Expression in Asian Population, and In Vitro Carcinogenic Bladder Tumor
Source: Oxid Med Cell Longev. 2022 Jan 7;2022:3459855. doi: 10.1155/2022/3459855 (PMC8760535; doi:10.1155/2022/3459855)
Supplement: Supplementary 9 — Table S8: significant genes in Data1 (low vs. high) and Data2 (low, medium, and high concentrations) and the overlap between various combinations. [file 3459855.f9.pdf]

Table S8. Significant genes in Data-1 (Low Vs High) and Data-2 (low, medium, and high concentrations) and the overlap between various combinations.

Conditions	total genes	Gene names
LowVsHigh_Data1-2, LowVsMed_Data1-2 MedVsHigh_Data1-2	4	XLOC_012515
		IL8
		TNS1
		MALAT1
LoeVsHigh_Data1-1 LowVsHigh_Data1-2 LowVsMed_Data1-2	2	ZDHHC23
		C9orf40
LoeVsHigh_Data1-1 LowVsHigh_Data1-2 MedVsHigh_Data1-2	2	HS3ST1
		DDHD2
LoeVsHigh_Data1-1 LowVsMed_Data1-2 MedVsHigh_Data1-2	1	PSPH
LowVsHigh_Data1-2 LowVsMed_Data1-2	315	
		C17orf103
		LOC151009
		KRTAP4-12
		BTG2
		XLOC_006924
		PRSS33
		LOC100289580
		C9orf29
		ADAM12
		IRAK1
		XLOC_008527
		LOC100129534
		LOC100134317
		LGR6
		RHOXF1
		NLRC4
		LOC100133612
		XLOC_012503
		NAT10
		XLOC_011924
		TGM2
		NASP
		CA14
		XLOC_002996
		CCL3L3
		LINC00260
		LOC100506437
		XLOC_l2_015100
		DACH1
		CST7
		ITGB4
		LOC283688
		CEACAM8

SLC26A8
BPIFB2
FAM27E3
XLOC_l2_002886
C19orf25
FAM182B
XLOC_004800
XLOC_011361
LOC388630
KIR2DL4
C14orf56
LOC286149
LMF1
XLOC_l2_013963
DNAL4
TERC
XLOC_009192
ZNHIT2
FLJ42709
XLOC_l2_013506
BAMBI
TNNI3
MORN1
NCRNA00083
LOC389273
OVGP1
XLOC_004093
ORMDL3
C1orf190
AKNA
NEAT1
XLOC_011719
C17orf65
FAM27A
XLOC_007868
LOC100506930
XLOC_010366
XLOC_000884
PRR5
NAALADL2
EGR1
VWA1
NYX
NCR2
ACTR5
RGS14
C10orf76

CPNE2
XLOC_000683
PHACTR1
C2orf63
SLC34A1
XLOC_005981
XLOC_009567
HP
FAAH
XLOC_012145
FLOT2
CHRM4
NPPA
LOC100132356
XLOC_002172
ZAN
SEC14L3
EYA1
CRABP1
RRP7B
HPR
HMX2
LOC645195
XLOC_002802
APBA2
LOC339807
LOC401320
XLOC_l2_015752
LOC399744
EGR2
CMA1
LOC100287633
UPK3B
LOC497256
XLOC_007895
LPCAT2
PGLYRP1
CSNK1G2
XLOC_l2_003666
PRR25
SYNJ2
SPP1
ANKDD1A
GPR141
BEND7
PTGS2
FLJ39051

TAOK2
IER5L
WNK4
XLOC_006670
MSR1
CYP4F12
SURF4
MEX3D
CAMP
OBSCN
SFN
LENG8
SLC26A11
LOC100506924
LOC100507540
G0S2
CSRNP1
UBL4B
XLOC_002580
MIAT
INHBB
LOC100133286
C1orf140
MIA
LOC100134391
AZU1
XLOC_004308
TNFRSF12A
XLOC_000762
CEP104
XLOC_007770
GPR88
XLOC_001362
LOC643802
IRF2BPL
XLOC_003462
LOC642947
CREB5
FBRS
XLOC_l2_011204
XLOC_008079
XLOC_l2_009468
OR9A2
ZNF333
SMR3A
SSX3
NCAPH

SHD
XLOC_001686
MDGA1
UCKL1
GRB10
XLOC_l2_010239
HOTAIR
DOCK9
GADD45G
ZNF385C
PP12719
XLOC_012227
XLOC_010065
NAMPT
XLOC_003194
CRYAA
XLOC_012165
LOC391322
LOC100507948
XLOC_011570
FLJ40434
CA4
XLOC_014393
NOP56
LAMB1
XLOC_007738
LYG2
MOV10L1
LOC100505978
PLA2G5
IL17REL
NPC1L1
FLJ43826
UNC5C
XLOC_008586
SAMD11
DOK3
FOS
XLOC_006193
SHISA4
ARHGEF4
XLOC_006277
CELA2B
SCARA3
CBX7
CAV1
ADORA3

ACTL7A
CYP1B1-AS1
CHRFAM7A
SYNE1
MXD3
HCG18
C12orf51
DUX4L4
GALNT14
PANX2
GDF5
KLF14
XLOC_006188
1-Mar
XLOC_012831
ACBD4
LOC100506342
XLOC_002643
XLOC_010730
TBC1D7
XLOC_013981
SLC41A3
XLOC_005900
LOC100129196
XLOC_007635
XLOC_009356
ABP1
XLOC_003650
XLOC_005731
BEST2
XLOC_014408
KIRREL3
BNIP3L
HRC
HEMGN
XLOC_004188
Q6LRY1
ADCY4
HILPDA
LOC100288292
LOC100129069
LOC652119
LOC145837
STAG3L2
FOXH1
KCNQ3
LOC731275

		ANKRD33
		XLOC_006476
		XLOC_006025
		FOSB
		XLOC_006638
		LOC100506220
		XLOC_l2_015562
		XLOC_l2_015855
		XLOC_013679
		XLOC_008609
		ZNF418
		XLOC_004828
		LCN2
		OR1S1
		IL28RA
		MIR22HG
		LOC401109
		LOC100652730
		HIST1H1D
		RBMY1B
		SERP2
		PIK3R6
		MMP19
		BPI
		CCL3
		HGFAC
		XLOC_012007
		CRISPLD2
		LOC100268168
		LOC100128714
		ARHGEF10
		TFF3
		XLOC_008151
		EVPL
		XLOC_l2_015532
		XLOC_012618
		STK17B
		REREP3
		LOC100129931
		LOC100652951
		ALPL
		XLOC_013826
		XLOC_001370
		C19orf59
		MMP9
		XLOC_006593
LowVsHigh_Data1-2 MedVsHigh_Data1-2	64	KCNG1

XLOC_l2_004611
UTY
DSP
TTTY15
XLOC_l2_013931
FAM129C
TXLNG2P
XLOC_011248
LINC00482
LOC283663
RAP1GAP2
RPS4Y2
GYPC
XLOC_l2_015849
RNASE2
XLOC_l2_001085
XLOC_008015
RBM38
LINC00230A
LOC100128252
TAGAP
XLOC_l2_015178
NOTCH2NL
NPRL3
LOC100506003
HAGH
GOLGA8A
USP9Y
FCRL2
CCDC144A
RPS4Y1
OASL
TSPAN5
LOC100509121
OPLAH
BMS1P1
TSIX
ANKRD20A2
XLOC_008323
SNORD90
RNASE3
TCL1A
MXI1
DDX3Y
XIST
XPNPEP3
LOC100129387

		GSPT1
		XLOC_008276
		XLOC_008185
		PRKY
		TRIM10
		LOC643072
		EIF1AY
		XLOC_l2_015892
		XLOC_001823
		GFRA1
		KDM5D
		PLAC8
		BMS1P5
		ZFY
		GABRA3
		LRRC14
LoeVsHigh_Data1-1 LowVsHigh_Data1-2	2	ATP6V0D1
		FCER1G
LowVsMed_Data1-2 MedVsHigh_Data1-2	19	ZNF646
		C5orf38
		XLOC_012571
		ANXA8L2
		XLOC_l2_013300
		SNORA74A
		LOC100506533
		MAP7
		DPYSL4
		ANKRD20A5P
		LOC100507254
		HEBP1
		TUBB2A
		HBE1
		XLOC_003400
		LOC728190
		TUBB8
		C4BPA
		PSPHP1
LoeVsHigh_Data1-1 LowVsMed_Data1-2	2	SEMA4A
		SGCE
LowVsHigh_Data1-2	189	ELMO2
		LOC100131702
		FAM153A
		ZSCAN18
		LOC100507803
		RTDR1
		HIST1H4J
		LOC653075

XLOC_001406
LOC100507131
ZNF347
STRADB
ACVRL1
CLCC1
C5AR1
HNF1A-AS1
GPR37L1
NT5M
XLOC_l2_011227
C14orf45
XLOC_013796
DNAH17
PAQR6
ANKRD35
GGT8P
LOC158863
ORM1
SNAR-D
TRIM3
THBS3
NFE4
XLOC_006338
LOC100131820
LOC155060
XLOC_002125
DNAH12
USP10
CAPN1
TRIP12
XLOC_001342
ERAP1
CHI3L1
XLOC_l2_006196
FCGBP
TBXA2R
LOC100506965
C6orf105
XLOC_009670
TRAT1
C5orf4
LOC100505736
SULT1A2
XLOC_013557
ATHL1
LOC100507195

MYBPH
LOC340335
XLOC_003772
LOC100130916
SFRP2
LOC100190939
ZNF502
XLOC_l2_015212
TTC24
LOC642648
CCR2
VWCE
LOC100507656
LMOD1
SUPT3H
THPO
TACR2
MPP1
XLOC_001745
PLA2G4C
FAM131C
TMEM146
OSM
DUSP1
LOC254896
XLOC_l2_014757
MRC2
FCAR
PRKDC
XLOC_002473
LOC100507218
CHMP3
VPRBP
XLOC_l2_002194
ANKRD36BP2
RBM3
KLC3
XLOC_009898
LOC283050
XLOC_l2_010064
XLOC_011346
XLOC_001496
BLK
XLOC_012575
LOC100287225
HIST1H4K
CCDC164

XLOC_l2_001496
CCDC64B
RERE
XLOC_001680
ACSM5
LGALS2
LOC728431
PDZK1IP1
GPC4
XLOC_005810
LOC100505874
LOC731424
XPO6
FAM153B
PLEK2
XLOC_003405
XLOC_002749
XLOC_003549
KREMEN1
TMPRSS5
CLCN1
XLOC_007370
SNORA34
FAM109B
SZT2
G6PD
LOC100129034
OR6T1
LTF
XLOC_012240
XLOC_003240
LOC100170939
XLOC_001148
RNF24
HOXA9
PRRC2A
LOC100190986
LRRN3
DDX17
BFSP1
XLOC_007407
GBP3
CECR6
NUDT13
ATP6V1G2
SNAR-B2
LOC100505857

LowVsMed_Data1-2

	TGFBR2
	ERVMER34-1
	PRDM5
	MTERFD2
	LOC100129726
	LRG1
	XLOC_011569
	TMEM145
	RORC
	ORM2
	PGM5
	UNC119
	ELMOD3
	LOC145216
	KLK1
	SNAR-G2
	SLC25A45
	NHSL2
	FAM181A
	XLOC_l2_011911
	LOC100653206
	TPST1
	LOC728052
	HOXD9
	XLOC_l2_007184
	LOC729175
	RN5-8S1
	CDC42EP1
	XLOC_006858
	Q3KR39
	KCNJ15
	VSIG10
	RUNDC3A
	LOC400682
	CFD
	NKIRAS2
	FLJ43681
	XLOC_l2_004706
	ACY3
	TNNT1
306	KCNK16
	XLOC_004585
	FLJ34208
	XLOC_001998
	CTAGE10P
	CNTNAP1
	XLOC_012594

XLOC_011104
C11orf42
CXCR4
SEMG1
PPP1R14A
LOC100129826
C2orf73
RBFOX3
C22orf45
XLOC_003863
C2orf49
TFDP2
XK
CEACAM6
ITLN1
SFTA2
KNDC1
NR4A2
XLOC_011181
LOC100505495
LYRM2
XLOC_009021
XLOC_l2_011854
CYP2F1
RGS16
LOC100506853
XLOC_004127
SIGLEC8
LOC100499227
XLOC_l2_008609
VKORC1
OR5AP2
ELANE
CLC
SLC18A3
YOD1
PLK3
XLOC_005681
DUX4
XLOC_013686
LOC404266
XLOC_013546
XLOC_012841
XLOC_013581
P2RX3
CSF1
ATOH8

UGT2B15
XLOC_000329
XLOC_005505
SEC14L1
DUSP2
LOC92659
C9orf50
XLOC_l2_000297
LOC728054
XLOC_002254
LOC619207
LOC100506775
LOC728377
FOXB1
XLOC_008558
LOC100132330
OR2W3
LOC100131831
RCAN2
LRRC10
FGF1
SEZ6
C9orf173
XLOC_007354
SLC6A18
LOC100506767
C9orf25
ADARB2-AS1
XLOC_007530
XLOC_l2_006640
RTN4RL1
PBX1
XLOC_013602
EMR1
ARHGEF16
XLOC_004535
Q20ZE8
CARD14
OTOA
RNF182
XLOC_l2_013242
ARGFX
VWA2
FAM66A
PITX1
XLOC_012335
LOC286058

XLOC_011645
BCORP1
XLOC_002049
KRT76
WBP1
LOC100288594
FHL2
MLL4
CNTNAP3B
CD177
CCDC134
LOC100132249
XLOC_005176
BTNL3
LOC727721
XLOC_006151
XLOC_l2_002767
XLOC_l2_007767
LPPR4
SEZ6L2
UNQ6975
XLOC_013562
XLOC_005083
TLR5
CLPS
CCL4
OLIG1
FAM186B
XLOC_001608
IL15RA
TRIB1
TDRD9
DUX4L9
RAB44
ITGB2
XLOC_l2_012071
XLOC_000550
LOC100506262
LOC388210
PCDHB13
MGC39584
KRT31
XLOC_004700
LINC00494
SEMA4B
DPRX
XLOC_001441

XLOC_000974
GTSCR1
EGR3
PRB3
XLOC_013785
SEPT7P2
LOC255167
ZSCAN1
DOT1L
LOC100506613
XLOC_001974
LOC148696
XLOC_002909
REEP1
PTGDR2
RGS1
XLOC_011515
SNORA29
PPAN
XLOC_014107
DEFA3
XLOC_001516
XLOC_009000
XLOC_004710
CGREF1
LOC100653060
XLOC_l2_004381
LOC100506380
NKAIN1
KRTAP10-12
LALBA
XLOC_007326
COL11A2
RINT1
TLE1
XLOC_l2_010014
PALM
CD24
NFASC
LOC729424
RNF167
AMFR
SLC5A6
ISLR
OLFM4
FLJ25694
PEA15

MESTIT1
OR3A3
CLDN3
FLJ39632
LOC100505697
MUC6
LOC400662
LOC200830
SRRM3
PRIMA1
FXYD5
NOTO
TRAK2
XLOC_008144
XLOC_000822
XLOC_007511
SPOCK2
MTVR2
XLOC_000377
XLOC_007438
ZNF501
PFDN6
XLOC_003714
THBS1
OR7E24
SMPD3
DCAF4L2
PMAIP1
XLOC_l2_000465
XLOC_010936
HSP90B1
CXCL1
XLOC_009038
XLOC_004346
XLOC_011519
GPR146
KLRAP1
LOC100233156
TRIM71
CPLX2
ARSD
SLC29A1
LIN9
TFAP2A
XLOC_l2_006718
XLOC_000630
LOC100134229

MYZAP
XLOC_013814
RGS12
MAP1LC3A
SNORA28
SPAG8
MEG3
GPR25
LOC100499221
Q8VGA8
LSR
ACTR3BP5
LOC100134240
ZNF488
XLOC_006486
XLOC_002275
LOC284757
LOC100134259
LOC100129869
XLOC_011978
PER1
XLOC_001847
NFXL1
LOC100289090
XLOC_014003
SNORA39
LOC648987
ALAS2
XLOC_l2_004817
RGPD1
XLOC_l2_011873
XLOC_008504
S100A12
FOXO3
CD69
FLJ42875
RNF222
FAM127B
B4GALT5
SPTBN4
OR51I2
XLOC_001938
XLOC_004577
XLOC_l2_014931
XLOC_l2_012374
C6orf147
ZFP57

		XLOC_l2_005415
		CEBPE
		OLIG2
		SLC22A10
		SH2D5
		ZBTB32
		AAK1
		GSTT1
		ACCSL
		PDE4A
		CYP1B1
		LOC441601
		XLOC_011880
		C12orf69
		SOAT2
		MYB
		NAT14
MedVsHigh_Data1-2	55	SLC5A11
		XLOC_011081
		NCRNA00185
		LOC100506159
		XLOC_l2_011118
		SLC6A10P
		LEPREL2
		DNAJA4
		QDPR
		CLEC2B
		HBZ
		DCAF12
		LOC100132207
		XLOC_008745
		XLOC_009123
		TNFAIP6
		ATP6AP1L
		XLOC_005119
		SYCE3
		EPB49
		LOC100506035
		XLOC_009389
		IGFBP2
		XLOC_003810
		KLRC4
		HLA-DQA1
		TRIM58
		UCP2
		MCF2L
		LOC100506854

		EIF2AK2
		XLOC_011718
		SESN3
		LOC100128262
		DDIT4
		PGF
		LCN10
		OAS2
		SLC6A8
		XLOC_012099
		HBM
		GLRX5
		E2F2
		HERC5
		KANK2
		LOC100131510
		RFPL4A
		SLC6A9
		ESPN
		XLOC_010686
		TTTY10
		SNORD3B-1
		BRWD1
		IFIT3
		MLLT4
LoeVsHigh_Data1-1	223	COBLL1
		KRTCAP2
		ZNF799
		BTN2A1
		ZFP28
		POLI
		CERK
		DOP1B
		TAS2R10
		DIP2A
		HERC2
		ZNF510
		ZNF506
		LPAR4
		SMIM3
		PAPSS2
		ZFP14
		CARD11
		TRAF5
		PLCG1
		ZNF43
		ATP6V0B

ZNF141
AQP10
LGALS1
SLC15A4
CABP5
SERPING1
ZNF75A
TMEM156
ALCAM
MRPS17
NIPSNAP3B
ZNF836
SNORA44
CCDC122
EBPL
EFCAB7
UPF3A
SNX25
BACE2
NDUFAF6
RPL32
NUP88
VPS36
MOB4
PAN2
CBR4
IFT80
KCNQ5
DTX1
PEX1
CCR3
ANKLE2
ZNF138
GORAB
TUBA1B
HEATR5B
SMC6
DGKD
FAM169A
GAA
DEF8
TTC13
TAS2R19
CTSS
NEMP2
C12orf65
AKTIP

DLST
NCAPD3
ZNF649
ZBTB16
HIGD1A
PDPR
RAB6B
RAD54B
MBD4
ZFP30
ZNF234
SNORA14A
ITPR1
PSENEN
LIPA
NT5E
ACVR1C
CRIM1
LOC100128398
TIMM29
FLNB
PSIP1
GCNT2
ARHGEF18
PPDPF
LRIG1
TMC6
OSGEPL1
CENPC
NHEJ1
LIMS1
SLC31A2
ZNF780B
CCDC14
EEF2K
SNHG32
MT1X
TIMP1
KAT2A
SPIC
MFSD1
RAB31
PIGT
KLHDC2
FAM149B1
HLA-F-AS1
ABI3

TAS2R14
XCL2
ZNF33B
SLC4A10
LY6G5C
GRAMD1C
DDX55
XCL1
ATP6V1F
ZNF767P
ADARB1
CD163
LMO7
HNRNPH1
PPP1R2
ACSS1
RBM4B
METTL18
GUSBP5
CNR2
PIK3C2B
CHMP2A
RTN4IP1
PTGDS
CPSF1
S100A4
YBEY
BTN2A2
ZNF808
SESN1
ZNF844
ZNF286A
ZNF302
UFSP2
JUNB
TP53INP1
TBC1D10C
ZNF841
TTC39B
IFI30
EMP3
ZNF426
ZNF121
ADCY10P1
MIR600HG
C1QB
AP3S1

ZNF160
PMS2P5
DZIP3
CCDC144NL
CRYGS
NR2C1
TNFRSF13B
IL4R
MT1F
JUP
INTS4
GTPBP6
LY6E
PATJ
ATP10D
HAPLN3
SUDS3
RPL8
ELP1
AIM2
NUP205
MT1H
DIS3L
RPL31
CLUAP1
SFMBT1
KLF13
C11orf65
HIP1
RRP15
GGTA1P
C18orf54
ABCA5
ZBTB25
HIGD2A
CXCL16
MT2A
CISH
ZNF33A
PHTF2
TAS2R20
LY96
ERCC6L2
ATG14
PRMT7
ZNF678
ENPP3

SLC38A11
SNORD29
LMBR1
ZNF251
BTAF1
ZNF440
SMAD5
ZBED5
ZNF224
MYADM
DNAJC10
SPEF2
WDSUB1
