# Supplementary material for: Association between Arsenic Level, Gene Expression in Asian Population, and In Vitro Carcinogenic Bladder Tumor
Source: Oxid Med Cell Longev. 2022 Jan 7;2022:3459855. doi: 10.1155/2022/3459855 (PMC8760535; doi:10.1155/2022/3459855)
Supplement: Supplementary 10 — Table S9: significant pathways in Data1 and Data2 (low, medium, and high concentrations) and the overlap between various combinations. [file 3459855.f10.pdf]

Table S9. Significant pathways in Data-1 and Data-2 (low, medium, and high concentrations) and the overlap between various combinations.

Population	Total	Pathways
Data-1, Data-2 (low, high) & Data-2 (low, medium)	2	ILK Signaling
		Neuroinflammation Signaling Pathway
Data-2 (low, high) & Data-2 (low, medium)	10	TREM1 Signaling
		IL-8 Signaling
		Communication between Innate and Adaptive Immune Cells
		MIF Regulation of Innate Immunity
		Osteoarthritis Pathway
		Airway Pathology in Chronic Obstructive Pulmonary Disease
		CDK5 Signaling
		Gαi Signaling
		Inhibition of Matrix Metalloproteases
		Atherosclerosis Signaling
Data-2 (low, high) & Data-2 (medium, high)	1	Methylglyoxal Degradation I
Data-1 & Data-2 (low, high)	6	GDNF Family Ligand-Receptor Interactions
		HOTAIR Regulatory Pathway
		Fc Epsilon RI Signaling
		PDGF Signaling
		VEGF Family Ligand-Receptor Interactions
		Phagosome Formation
Data-1 & Data-2 (low, medium)	3	Thyroid Cancer Signaling
		Caveolar-mediated Endocytosis Signaling
		Macropinocytosis Signaling
Data-1 & Data-2 (medium, high)	4	Serine Biosynthesis
		Phagosome Maturation
		EIF2 Signaling
		Iron homeostasis signaling pathway
Data-2 (low, high)	12	Neuroprotective Role of THOP1 in Alzheimer's Disease
		Hematopoiesis from Pluripotent Stem Cells
		Cell Cycle: G2/M DNA Damage Checkpoint Regulation
		LXR/RXR Activation
		Phospholipase C Signaling
		Eicosanoid Signaling
		Anandamide Degradation
		White Adipose Tissue Browning Pathway

		Sirtuin Signaling Pathway
		Acute Phase Response Signaling
		p38 MAPK Signaling
		MIF-mediated Glucocorticoid Regulation
Data-2 (low, medium)	20	ERK5 Signaling
		Pathogenesis of Multiple Sclerosis
		G-Protein Coupled Receptor Signaling
		Differential Regulation of Cytokine Production in Intestinal Epithelial Cells by IL-17A and IL-17F
		Role of IL-17A in Psoriasis
		Differential Regulation of Cytokine Production in Macrophages and T Helper Cells by IL-17A and IL-17F
		IL-17A Signaling in Gastric Cells
		Granulocyte Adhesion and Diapedesis
		Relaxin Signaling
		Role of IL-17F in Allergic Inflammatory Airway Diseases
		Bladder Cancer Signaling
		Breast Cancer Regulation by Stathmin1
		Role of Hypercytokinemia/hyperchemokinemia in the Pathogenesis of Influenza
		Aryl Hydrocarbon Receptor Signaling
		p53 Signaling
		Agranulocyte Adhesion and Diapedesis
		PI3K/AKT Signaling
		Role of IL-17A in Arthritis
		IGF-1 Signaling
		Glucocorticoid Receptor Signaling
Data-2 (medium, high)	11	Superpathway of Serine and Glycine Biosynthesis I
		Coronavirus Pathogenesis Pathway
		Remodeling of Epithelial Adherens Junctions
		mTOR Signaling
		Role of Pattern Recognition Receptors in Recognition of Bacteria and Viruses
		Sertoli Cell-Sertoli Cell Junction Signaling
		Germ Cell-Sertoli Cell Junction Signaling
		Phenylalanine Degradation I (Aerobic)

		γ-glutamyl Cycle
		Epithelial Adherens Junction Signaling
		Regulation of eIF4 and p70S6K Signaling
		Reelin Signaling in Neurons
		Molecular Mechanisms of Cancer
		fMLP Signaling in Neutrophils
		NF-κB Activation by Viruses
		Synaptic Long Term Depression
		Gap Junction Signaling
		PKCθ Signaling in T Lymphocytes
		Role of NFAT in Regulation of the Immune Response
		LPS-stimulated MAPK Signaling
		Glioblastoma Multiforme Signaling
		Glycogen Degradation III
		Insulin Secretion Signaling Pathway
		Renin-Angiotensin Signaling
		IL-3 Signaling
		Leptin Signaling in Obesity
		Neurotrophin/TRK Signaling
		Role of Tissue Factor in Cancer
		Glioma Signaling
		ErbB Signaling
		Semaphorin Neuronal Repulsive Signaling Pathway
		Thrombopoietin Signaling
		PI3K Signaling in B Lymphocytes
		IL-9 Signaling
		Adrenomedullin signaling pathway
		Angiopoietin Signaling
		Role of JAK1 and JAK3 in γc Cytokine Signaling
		iCOS-iCOSL Signaling in T Helper Cells
		Myo-inositol Biosynthesis
		Neuropathic Pain Signaling In Dorsal Horn Neurons
		Leukocyte Extravasation Signaling
		Apelin Cardiomyocyte Signaling Pathway
		T Cell Exhaustion Signaling Pathway
		CD40 Signaling

Data-1	97	CTLA4 Signaling in Cytotoxic T Lymphocytes
		Factors Promoting Cardiogenesis in Vertebrates
		2-ketoglutarate Dehydrogenase Complex
		Neuregulin Signaling
		Lymphotoxin β Receptor Signaling
		Adipogenesis pathway
		Heparan Sulfate Biosynthesis
		Non-Small Cell Lung Cancer Signaling
		GP6 Signaling Pathway
		NF-κB Signaling
		Role of p14/p19ARF in Tumor Suppression
		Synaptic Long Term Potentiation
		Gαq Signaling
		Th2 Pathway
		14-3-3-mediated Signaling
		CD28 Signaling in T Helper Cells
		UVB-Induced MAPK Signaling
		Hepatic Fibrosis Signaling Pathway
		ErbB4 Signaling
		G Protein Signaling Mediated by Tubby
		Heparan Sulfate Biosynthesis (Late Stages)
		Amyloid Processing
		Th1 and Th2 Activation Pathway
		Coenzyme A Biosynthesis
		Synaptogenesis Signaling Pathway
		Huntington's Disease Signaling
		CCR3 Signaling in Eosinophils
		eNOS Signaling
		P2Y Purigenic Receptor Signaling Pathway
		Prolactin Signaling
		Tec Kinase Signaling
		RAR Activation
		VEGF Signaling
		Virus Entry via Endocytic Pathways
		Endocannabinoid Neuronal Synapse Pathway
		p70S6K Signaling

		IL-4 Signaling
		Nitric Oxide Signaling in the Cardiovascular System
		HIPPO signaling
		Aldosterone Signaling in Epithelial Cells
		Small Cell Lung Cancer Signaling
		Human Embryonic Stem Cell Pluripotency
		Erythropoietin Signaling
		Sulfate Activation for Sulfonation
		CREB Signaling in Neurons
		Thrombin Signaling
		Type II Diabetes Mellitus Signaling
		Dopamine-DARPP32 Feedback in cAMP Signaling
		Growth Hormone Signaling
		FGF Signaling
		HGF Signaling
		HER-2 Signaling in Breast Cancer
		Glioma Invasiveness Signaling
		Gustation Pathway
		EGF Signaling
		IL-17 Signaling
		Axonal Guidance Signaling
		Endothelin-1 Signaling
		Calcium-induced T Lymphocyte Apoptosis
		SPINK1 General Cancer Pathway
		T Cell Receptor Signaling
		Role of NANOG in Mammalian Embryonic Stem Cell Pluripotency
		NRF2-mediated Oxidative Stress Response
		AMPK Signaling
