# Supplementary material for: Association between Arsenic Level, Gene Expression in Asian Population, and In Vitro Carcinogenic Bladder Tumor
Source: Oxid Med Cell Longev. 2022 Jan 7;2022:3459855. doi: 10.1155/2022/3459855 (PMC8760535; doi:10.1155/2022/3459855)
Supplement: Supplementary 11 — Table S10: list of 147 unique genes. [file 3459855.f11.pdf]

Table S10. List of 147 unique genes.

	GB_ACC	Symbol	Species.Scientific.Name	Annotation.Date	Sequence.Type	Sequence.Source	Target.Description	Representative.Public.ID	Gene.Title	Gene.Symbol	ENTREZ_GENE_ID	RefSeq.Transcript.ID	Gene.Ontology.Biological.Process	Gene.Ontology.Cellular.Component	Gene.Ontology.Molecular.Function
200938_s_at	AI920976	RERE	Homo sapiens	6-Oct-14	Consensus sequence	GenBank	Consensus includes gb:AI920976 /FEA=EST /DB_XREF=gi:5656940 /DB_XREF=est:wo16h05.x1 /CLONE=IMAGE:2455545 /UG=Hs.194369 arginine-glutamic acid dipeptide (RE) repeats /FL=gb:AF118275.1 gb:NM_012102.1 gb:AB036737.1	AI920976	arginine-glutamic acid dipeptide (RE) repeats	RERE	473	NM_001042681 /// NM_001042682 /// NM_012102 /// XM_005263464 /// XM_005263465 /// XM_005263466 /// XM_006710653 /// XM_006710654	0006338 // chromatin remodeling // inferred from electronic annotation /// 0006351 // transcription, DNA-templated // inferred from electronic annotation /// 0006355 // regulation of transcription, DNA-templated // inferred from electronic annotation /// 0006607 // NLS-bearing protein import into nucleus // traceable author statement /// 0007275 // multicellular organismal development // inferred from electronic annotation	0000118 // histone deacetylase complex // inferred from electronic annotation /// 0005634 // nucleus // non-traceable author statement /// 0005739 // mitochondrion // inferred from direct assay	0003677 // DNA binding // inferred from electronic annotation /// 0003682 // chromatin binding // inferred from electronic annotation /// 0003700 // sequence-specific DNA binding transcription factor activity // inferred from electronic annotation /// 0005515 // protein binding // inferred from physical interaction /// 0008267 // poly-glutamine tract binding // traceable author statement /// 0008270 // zinc ion binding // inferred from electronic annotation /// 0043565 // sequence-specific DNA binding // inferred from electronic annotation /// 0046872 // metal ion binding // inferred from electronic annotation

200939_s_at	NM_012102	RERE	Homo sapiens	6-Oct-14	Exemplar sequence	GenBank	gb:NM_012102.1 /DEF=Homo sapiens arginine-glutamic acid dipeptide (RE) repeats (RERE), mRNA. /FEA=mRNA /GEN=RERE /PROD=arginine-glutamic acid dipeptide (RE) repeats /DB_XREF=gi:6912253 /UG=Hs.194369 arginine-glutamic acid dipeptide (RE) repeats /FL=gb:AF118275.1 gb:NM_012102.1 gb:AB036737.1	NM_012102	arginine-glutamic acid dipeptide (RE) repeats	RERE	473	NM_001042681 /// NM_001042682 /// NM_012102 /// XM_005263464 /// XM_005263465 /// XM_005263466 /// XM_006710653 /// XM_006710654	0006338 // chromatin remodeling // inferred from electronic annotation /// 0006351 // transcription, DNA-templated // inferred from electronic annotation /// 0006355 // regulation of transcription, DNA-templated // inferred from electronic annotation /// 0006607 // NLS-bearing protein import into nucleus // traceable author statement /// 0007275 // multicellular organismal development // inferred from electronic annotation	0000118 // histone deacetylase complex // inferred from electronic annotation /// 0005634 // nucleus // non-traceable author statement /// 0005739 // mitochondrion // inferred from direct assay	0003677 // DNA binding // inferred from electronic annotation /// 0003682 // chromatin binding // inferred from electronic annotation /// 0003700 // sequence-specific DNA binding transcription factor activity // inferred from electronic annotation /// 0005515 // protein binding // inferred from physical interaction /// 0008267 // poly-glutamine tract binding // traceable author statement /// 0008270 // zinc ion binding // inferred from electronic annotation /// 0043565 // sequence-specific DNA binding // inferred from electronic annotation /// 0046872 // metal ion binding // inferred from electronic annotation

200940_s_at	AB036737	RERE	Homo sapiens	6-Oct-14	Exemplar sequence	GenBank	gb:AB036737.1 /DEF=Homo sapiens mRNA for RERE, complete cds. /FEA=mRNA /PROD=RERE /DB_XREF=gi:8096339 /UG=Hs.194369 arginine-glutamic acid dipeptide (RE) repeats /FL=gb:AF118275.1 gb:NM_012102.1 gb:AB036737.1	AB036737	arginine-glutamic acid dipeptide (RE) repeats	RERE	473	NM_001042681 /// NM_001042682 /// NM_012102 /// XM_005263464 /// XM_005263465 /// XM_005263466 /// XM_006710653 /// XM_006710654	0006338 // chromatin remodeling // inferred from electronic annotation /// 0006351 // transcription, DNA-templated // inferred from electronic annotation /// 0006355 // regulation of transcription, DNA-templated // inferred from electronic annotation /// 0006607 // NLS-bearing protein import into nucleus // traceable author statement /// 0007275 // multicellular organismal development // inferred from electronic annotation	0000118 // histone deacetylase complex // inferred from electronic annotation /// 0005634 // nucleus // non-traceable author statement /// 0005739 // mitochondrion // inferred from direct assay	0003677 // DNA binding // inferred from electronic annotation /// 0003682 // chromatin binding // inferred from electronic annotation /// 0003700 // sequence-specific DNA binding transcription factor activity // inferred from electronic annotation /// 0005515 // protein binding // inferred from physical interaction /// 0008267 // poly-glutamine tract binding // traceable author statement /// 0008270 // zinc ion binding // inferred from electronic annotation /// 0043565 // sequence-specific DNA binding // inferred from electronic annotation /// 0046872 // metal ion binding // inferred from electronic annotation

200962_at	AI348010	RPL31	Homo sapiens	6-Oct-14	Consensus sequence	GenBank	Consensus includes gb:AI348010 /FEA=EST /DB_XREF=gi:4085216 /DB_XREF=est:qp61b10.x1 /CLONE=IMAGE:1927483 /UG=Hs.184014 ribosomal protein L31 /FL=gb:NM_000993.1	AI348010	ribosomal protein L31	RPL31	6160	NM_000993 /// NM_001098577 /// NM_001099693	0000184 // nuclear-transcribed mRNA catabolic process, nonsense-mediated decay // traceable author statement /// 0006412 // translation // non-traceable author statement /// 0006412 // translation // traceable author statement /// 0006413 // translational initiation // traceable author statement /// 0006414 // translational elongation // traceable author statement /// 0006415 // translational termination // traceable author statement /// 0006614 // SRP-dependent cotranslational protein targeting to membrane // traceable author statement /// 0010467 // gene expression // traceable [illegible]	0005622 // intracellular // inferred from electronic annotation /// 0005829 // cytosol // traceable author statement /// 0005840 // ribosome // inferred from electronic annotation /// 0016020 // membrane // inferred from direct assay /// 0022625 // cytosolic large ribosomal subunit // inferred from direct assay /// 0030529 // ribonucleoprotein complex // inferred from electronic [illegible]	0003723 // RNA binding // traceable author statement /// 0003735 // structural constituent of ribosome // non-traceable author statement /// 0044822 // poly(A) RNA binding // inferred from direct assay

200963_x_at	NM_000993	RPL31	Homo sapiens	6-Oct-14	Exemplar sequence	GenBank	gb:NM_000993.1 /DEF=Homo sapiens ribosomal protein L31 (RPL31), mRNA. /FEA=mRNA /GEN=RPL31 /PROD=ribosomal protein L31 /DB_XREF=gi:4506632 /UG=Hs.184014 ribosomal protein L31 /FL=gb:NM_000993.1	NM_000993	ribosomal protein L31	RPL31	6160	NM_000993 /// NM_001098577 /// NM_001099693	0000184 // nuclear-transcribed mRNA catabolic process, nonsense-mediated decay // traceable author statement /// 0006412 // translation // non-traceable author statement /// 0006412 // translation // traceable author statement /// 0006413 // translational initiation // traceable author statement /// 0006414 // translational elongation // traceable author statement /// 0006415 // translational termination // traceable author statement /// 0006614 // SRP-dependent cotranslational protein targeting to membrane // traceable author statement /// 0010467 // gene expression // traceable [illegible]	0005622 // intracellular // inferred from electronic annotation /// 0005829 // cytosol // traceable author statement /// 0005840 // ribosome // inferred from electronic annotation /// 0016020 // membrane // inferred from direct assay /// 0022625 // cytosolic large ribosomal subunit // inferred from direct assay /// 0030529 // ribonucleoprotein complex // inferred from electronic [illegible]	0003723 // RNA binding // traceable author statement /// 0003735 // structural constituent of ribosome // non-traceable author statement /// 0044822 // poly(A) RNA binding // inferred from direct assay

201015_s_at	NM_021991	JUP	Homo sapiens	6-Oct-14	Exemplar sequence	GenBank	gb:NM_021991.1 /DEF=Homo sapiens junction plakoglobin (JUP), transcript variant 2, mRNA. /FEA=mRNA /GEN=JUP /PROD=junction plakoglobin, isoform 1 /DB_XREF=gi:12056467 /UG=Hs.2340 junction plakoglobin /FL=gb:NM_021991.1 gb:BC000441.1	NM_021991	junction plakoglobin	JUP	3728	NM_002230 /// NM_021991 /// XM_005257313 /// XM_006721871 /// XM_006721872 /// XM_006721873 /// XM_006721874 /// XM_006721875 /// XM_006721876 /// XM_006721877 /// XM_006721878	0000122 // negative regulation of transcription from RNA polymerase II promoter // not recorded /// 0000902 // cell morphogenesis // not recorded /// 0002159 // desmosome assembly // inferred from direct assay /// 0002159 // desmosome assembly // inferred from mutant phenotype /// 0003136 // negative regulation of heart induction by canonical Wnt signaling pathway // not recorded /// 0003181 // atrioventricular valve morphogenesis // not recorded /// 0003308 // negative regulation of Wnt signaling pathway involved in heart development // not recorded /// 0007016 // cytoskeletal anchoring at [illegible]	0005634 // nucleus // inferred from mutant phenotype /// 0005667 // transcription factor complex // not recorded /// 0005737 // cytoplasm // inferred from mutant phenotype /// 0005829 // cytosol // inferred from sequence or structural similarity /// 0005856 // cytoskeleton // inferred from sequence or structural similarity /// 0005882 // intermediate filament // inferred from [illegible]	0003713 // transcription coactivator activity // inferred from direct assay /// 0005198 // structural molecule activity // not recorded /// 0005198 // structural molecule activity // non-traceable author statement /// 0005199 // structural constituent of cell wall // inferred by curator /// 0005488 // binding // inferred from electronic annotation /// 0005515 // protein binding // inferred from physical interaction /// 0019901 // protein kinase binding // not recorded /// 0019903 // protein phosphatase binding // inferred from physical interaction /// 0042803 // protein homodimerization activity // inferred from sequence or structural similarity /// 0045294 // alpha-catenin binding // inferred from physical [illegible]

201041_s_at	NM_004417	DUSP1	Homo sapiens	6-Oct-14	Exemplar sequence	GenBank	gb:NM_004417.2 /DEF=Homo sapiens dual specificity phosphatase 1 (DUSP1), mRNA. /FEA=mRNA /GEN=DUSP1 /PROD=dual specificity phosphatase 1 /DB_XREF=gi:7108342 /UG=Hs.171695 dual specificity phosphatase 1 /FL=gb:NM_004417.2	NM_004417	dual specificity phosphatase 1	DUSP1	1843	NM_004417	0000188 // inactivation of MAPK activity // inferred from sequence or structural similarity /// 0001706 // endoderm formation // not recorded /// 0006470 // protein dephosphorylation // not recorded /// 0006950 // response to stress // inferred from electronic annotation /// 0006979 // response to oxidative stress // traceable author statement /// 0007049 // cell cycle // inferred from electronic annotation /// 0009416 // response to light stimulus // inferred from electronic annotation /// 0010033 // response to organic substance // inferred from electronic annotation /// 0016311 // dephosphorylation // inferred from electronic [illegible]	0005634 // nucleus // inferred from direct assay /// 0005654 // nucleoplasm // /// 0005737 // cytoplasm // inferred from direct assay	0004721 // phosphoprotein phosphatase activity // inferred from electronic annotation /// 0004725 // protein tyrosine phosphatase activity // inferred from electronic annotation /// 0004726 // non-membrane spanning protein tyrosine phosphatase activity // traceable author statement /// 0005515 // protein binding // inferred from physical interaction /// 0008138 // protein tyrosine/serine/threonine phosphatase activity // not recorded /// 0008330 // protein tyrosine/threonine phosphatase activity // inferred from electronic annotation /// 0016787 // hydrolase activity // inferred from electronic annotation /// 0016791 // phosphatase activity // inferred from electronic annotation /// [illegible]

201044_x_at	AA530892	DUSP1	Homo sapiens	6-Oct-14	Consensus sequence	GenBank	Consensus includes gb:AA530892 /FEA=EST /DB_XREF=gi:2273598 /DB_XREF=est:ni97d11.s1 /CLONE=IMAGE:984789 /UG=Hs.171695 dual specificity phosphatase 1 /FL=gb:NM_004417.2	AA530892	dual specificity phosphatase 1	DUSP1	1843	NM_004417	0000188 // inactivation of MAPK activity // inferred from sequence or structural similarity /// 0001706 // endoderm formation // not recorded /// 0006470 // protein dephosphorylation // not recorded /// 0006950 // response to stress // inferred from electronic annotation /// 0006979 // response to oxidative stress // traceable author statement /// 0007049 // cell cycle // inferred from electronic annotation /// 0009416 // response to light stimulus // inferred from electronic annotation /// 0010033 // response to organic substance // inferred from electronic annotation /// 0016311 // dephosphorylation // inferred from electronic [illegible]	0005634 // nucleus // inferred from direct assay /// 0005654 // nucleoplasm // /// 0005737 // cytoplasm // inferred from direct assay	0004721 // phosphoprotein phosphatase activity // inferred from electronic annotation /// 0004725 // protein tyrosine phosphatase activity // inferred from electronic annotation /// 0004726 // non-membrane spanning protein tyrosine phosphatase activity // traceable author statement /// 0005515 // protein binding // inferred from physical interaction /// 0008138 // protein tyrosine/serine/threonine phosphatase activity // not recorded /// 0008330 // protein tyrosine/threonine phosphatase activity // inferred from electronic annotation /// 0016787 // hydrolase activity // inferred from electronic annotation /// 0016791 // phosphatase activity // inferred from electronic annotation /// [illegible]

201107_s_at	AI812030	THBS1	Homo sapiens	6-Oct-14	Consensus sequence	GenBank	Consensus includes gb:AI812030 /FEA=EST /DB_XREF=gi:5398596 /DB_XREF=est:tw46c12.x1 /CLONE=IMAGE:2262742 /UG=Hs.87409 thrombospondin 1 /FL=gb:NM_003246.1	AI812030	thrombospondin 1	THBS1	7057	NM_003246	0000187 // activation of MAPK activity // inferred from mutant phenotype /// 0001666 // response to hypoxia // non-traceable author statement /// 0001937 // negative regulation of endothelial cell proliferation // inferred from direct assay /// 0001937 // negative regulation of endothelial cell proliferation // inferred from mutant phenotype /// 0001953 // negative regulation of cell-matrix adhesion // inferred from direct assay /// 0002040 // sprouting angiogenesis // inferred from mutant phenotype /// 0002544 // chronic inflammatory response // inferred from expression pattern /// 0002576 // platelet degranulation // [illegible]	0005576 // extracellular region // traceable author statement /// 0005577 // fibrinogen complex // inferred from direct assay /// 0005615 // extracellular space // inferred from direct assay /// 0005737 // cytoplasm // inferred from electronic annotation /// 0005783 // endoplasmic reticulum // inferred from sequence or structural similarity /// 0009897 // external side of plasma membrane [illegible]	0001786 // phosphatidylserine binding // inferred from direct assay /// 0001948 // glycoprotein binding // non-traceable author statement /// 0001968 // fibronectin binding // inferred from direct assay /// 0005178 // integrin binding // inferred from mutant phenotype /// 0005509 // calcium ion binding // non-traceable author statement /// 0005515 // protein binding // inferred from physical interaction /// 0008201 // heparin binding // inferred from direct assay /// 0017134 // fibroblast growth factor binding // inferred from direct assay /// 0030169 // low-density lipoprotein particle binding // inferred from direct assay /// 0042802 // identical protein binding // non-traceable author [illegible]

201108_s _at	BF055462	THBS1	Homo sapiens	6-Oct-14	Consensus sequence	GenBank	Consensus includes gb:BF055462 /FEA=EST /DB_XREF=gi:1 0809358 /DB_XREF=est: 7j80e06.x1 /CLONE=IMAGE :3392770 /UG=Hs.87409 thrombospondi n 1 /FL=gb:NM_00 3246.1	BF055462	thrombospon din 1	THBS1	7057	NM_003246	0000187 // activation of MAPK activity // inferred from mutant phenotype /// 0001666 // response to hypoxia // non-traceable author statement /// 0001937 // negative regulation of endothelial cell proliferation // inferred from direct assay /// 0001937 // negative regulation of endothelial cell proliferation // inferred from mutant phenotype /// 0001953 // negative regulation of cell-matrix adhesion // inferred from direct assay /// 0002040 // sprouting angiogenesis // inferred from mutant phenotype /// 0002544 // chronic inflammatory response // inferred from expression pattern /// 0002576 // platelet degranulation // [illegible]	0005576 // extracellular region // traceable author statement /// 0005577 // fibrinogen complex // inferred from direct assay /// 0005615 // extracellular space // inferred from direct assay /// 0005737 // cytoplasm // inferred from electronic annotation /// 0005783 // endoplasmic reticulum // inferred from sequence or structural similarity /// 0009897 // external side of plasma membrane [illegible]	0001786 // phosphatidylserine binding // inferred from direct assay /// 0001948 // glycoprotein binding // non-traceable author statement /// 0001968 // fibronectin binding // inferred from direct assay /// 0005178 // integrin binding // inferred from mutant phenotype /// 0005509 // calcium ion binding // non-traceable author statement /// 0005515 // protein binding // inferred from physical interaction /// 0008201 // heparin binding // inferred from direct assay /// 0017134 // fibroblast growth factor binding // inferred from direct assay /// 0030169 // low-density lipoprotein particle binding // inferred from direct assay /// 0042802 // identical protein binding // non-traceable author [illegible]

201109_s_at	AV726673	THBS1	Homo sapiens	6-Oct-14	Consensus sequence	GenBank	Consensus includes gb:AV726673 /FEA=EST /DB_XREF=gi:10836094 /DB_XREF=est:AV726673 /CLONE=HTCBGC12 /UG=Hs.87409 thrombospondin 1 /FL=gb:NM_003246.1	AV726673	thrombospondin 1	THBS1	7057	NM_003246	0000187 // activation of MAPK activity // inferred from mutant phenotype /// 0001666 // response to hypoxia // non-traceable author statement /// 0001937 // negative regulation of endothelial cell proliferation // inferred from direct assay /// 0001937 // negative regulation of endothelial cell proliferation // inferred from mutant phenotype /// 0001953 // negative regulation of cell-matrix adhesion // inferred from direct assay /// 0002040 // sprouting angiogenesis // inferred from mutant phenotype /// 0002544 // chronic inflammatory response // inferred from expression pattern /// 0002576 // platelet degranulation // [illegible]	0005576 // extracellular region // traceable author statement /// 0005577 // fibrinogen complex // inferred from direct assay /// 0005615 // extracellular space // inferred from direct assay /// 0005737 // cytoplasm // inferred from electronic annotation /// 0005783 // endoplasmic reticulum // inferred from sequence or structural similarity /// 0009897 // external side of plasma membrane [illegible]	0001786 // phosphatidylserine binding // inferred from direct assay /// 0001948 // glycoprotein binding // non-traceable author statement /// 0001968 // fibronectin binding // inferred from direct assay /// 0005178 // integrin binding // inferred from mutant phenotype /// 0005509 // calcium ion binding // non-traceable author statement /// 0005515 // protein binding // inferred from physical interaction /// 0008201 // heparin binding // inferred from direct assay /// 0017134 // fibroblast growth factor binding // inferred from direct assay /// 0030169 // low-density lipoprotein particle binding // inferred from direct assay /// 0042802 // identical protein binding // non-traceable author [illegible]

201110_s_at	NM_003246	THBS1	Homo sapiens	6-Oct-14	Exemplar sequence	GenBank	gb:NM_003246.1 /DEF=Homo sapiens thrombospondin 1 (THBS1), mRNA. /FEA=mRNA /GEN=THBS1 /PROD=thrombospondin 1 /DB_XREF=gi:4507484 /UG=Hs.87409 thrombospondin 1 /FL=gb:NM_003246.1	NM_003246	thrombospondin 1	THBS1	7057	NM_003246	0000187 // activation of MAPK activity // inferred from mutant phenotype /// 0001666 // response to hypoxia // non-traceable author statement /// 0001937 // negative regulation of endothelial cell proliferation // inferred from direct assay /// 0001937 // negative regulation of endothelial cell proliferation // inferred from mutant phenotype /// 0001953 // negative regulation of cell-matrix adhesion // inferred from direct assay /// 0002040 // sprouting angiogenesis // inferred from mutant phenotype /// 0002544 // chronic inflammatory response // inferred from expression pattern /// 0002576 // platelet degranulation // [illegible]	0005576 // extracellular region // traceable author statement /// 0005577 // fibrinogen complex // inferred from direct assay /// 0005615 // extracellular space // inferred from direct assay /// 0005737 // cytoplasm // inferred from electronic annotation /// 0005783 // endoplasmic reticulum // inferred from sequence or structural similarity /// 0009897 // external side of plasma membrane [illegible]	0001786 // phosphatidylserine binding // inferred from direct assay /// 0001948 // glycoprotein binding // non-traceable author statement /// 0001968 // fibronectin binding // inferred from direct assay /// 0005178 // integrin binding // inferred from mutant phenotype /// 0005509 // calcium ion binding // non-traceable author statement /// 0005515 // protein binding // inferred from physical interaction /// 0008201 // heparin binding // inferred from direct assay /// 0017134 // fibroblast growth factor binding // inferred from direct assay /// 0030169 // low-density lipoprotein particle binding // inferred from direct assay /// 0042802 // identical protein binding // non-traceable author [illegible]

201235_s_at	BG339064	BTG2	Homo sapiens	6-Oct-14	Consensus sequence	GenBank	Consensus includes gb:BG339064 /FEA=EST /DB_XREF=gi:13145502 /DB_XREF=est:602436889F1 /CLONE=IMAGE:4554551 /UG=Hs.75462 BTG family, member 2 /FL=gb:U72649.1 gb:NM_006763.1	BG339064	BTG family, member 2	BTG2	7832	NM_006763	0006281 // DNA repair // traceable author statement /// 0006351 // transcription, DNA-templated // inferred from electronic annotation /// 0006355 // regulation of transcription, DNA-templated // inferred from electronic annotation /// 0006479 // protein methylation // inferred from electronic annotation /// 0006974 // cellular response to DNA damage stimulus // inferred from direct assay /// 0008285 // negative regulation of cell proliferation // inferred from mutant phenotype /// 0008306 // associative learning // inferred from electronic annotation /// 0009612 // response to mechanical stimulus // [illegible]	0070062 // extracellular vesicular exosome // inferred from direct assay	0001077 // RNA polymerase II core promoter proximal region sequence-specific DNA binding transcription factor activity involved in positive regulation of transcription // inferred from electronic annotation /// 0005515 // protein binding // inferred from physical interaction

201236_s_at	NM_006763	BTG2	Homo sapiens	6-Oct-14	Exemplar sequence	GenBank	gb:NM_006763.1 /DEF=Homo sapiens BTG family, member 2 (BTG2), mRNA. /FEA=mRNA /GEN=BTG2 /PROD=BTG family, member 2 /DB_XREF=gi:5802987 /UG=Hs.75462 BTG family, member 2 /FL=gb:U72649.1 gb:NM_006763.1	NM_006763	BTG family, member 2	BTG2	7832	NM_006763	0006281 // DNA repair // traceable author statement /// 0006351 // transcription, DNA-templated // inferred from electronic annotation /// 0006355 // regulation of transcription, DNA-templated // inferred from electronic annotation /// 0006479 // protein methylation // inferred from electronic annotation /// 0006974 // cellular response to DNA damage stimulus // inferred from direct assay /// 0008285 // negative regulation of cell proliferation // inferred from mutant phenotype /// 0008306 // associative learning // inferred from electronic annotation /// 0009612 // response to mechanical stimulus // [illegible]	0070062 // extracellular vesicular exosome // inferred from direct assay	0001077 // RNA polymerase II core promoter proximal region sequence-specific DNA binding transcription factor activity involved in positive regulation of transcription // inferred from electronic annotation /// 0005515 // protein binding // inferred from physical interaction

201350_at	NM_004475	FLOT2	Homo sapiens	6-Oct-14	Exemplar sequence	GenBank	gb:NM_004475.1 /DEF=Homo sapiens flotillin 2 (FLOT2), mRNA. /FEA=mRNA /GEN=FLOT2 /PROD=flotillin 2 /DB_XREF=gi:4758393 /UG=Hs.184488 flotillin 2 /FL=gb:NM_004475.1 gb:M60922.1	NM_004475	flotillin 2	FLOT2	2319	NM_004475 /// XM_005257950 /// XM_005257952 /// XM_005257953	0007155 // cell adhesion // inferred from electronic annotation /// 0008544 // epidermis development // traceable author statement /// 0010629 // negative regulation of gene expression // inferred from mutant phenotype /// 0050821 // protein stabilization // inferred from mutant phenotype /// 0090002 // establishment of protein localization to plasma membrane // inferred from mutant phenotype /// 1902992 // negative regulation of amyloid precursor protein catabolic process // inferred from mutant phenotype	0002080 // acrosomal membrane // inferred from electronic annotation /// 0005768 // endosome // inferred from direct assay /// 0005886 // plasma membrane // non-traceable author statement /// 0005901 // caveola // inferred from direct assay /// 0009986 // cell surface // inferred from direct assay /// 0016020 // membrane // inferred from direct assay /// 0016020 // membrane // inferred from [illegible]	0005515 // protein binding // inferred from physical interaction /// 0035255 // ionotropic glutamate receptor binding // inferred from electronic annotation

201505_at	NM_002291	LAMB1	Homo sapiens	6-Oct-14	Exemplar sequence	GenBank	gb:NM_002291.1 /DEF=Homo sapiens laminin, beta 1 (LAMB1), mRNA. /FEA=mRNA /GEN=LAMB1 /PROD=laminin, beta 1 precursor /DB_XREF=gi:4504950 /UG=Hs.82124 laminin, beta 1 /FL=gb:M61916.1 gb:NM_002291.1	NM_002291	laminin, beta 1	LAMB1	3912	NM_002291	0007155 // cell adhesion // traceable author statement /// 0007162 // negative regulation of cell adhesion // inferred from electronic annotation /// 0007411 // axon guidance // traceable author statement /// 0007566 // embryo implantation // inferred from electronic annotation /// 0016192 // vesicle-mediated transport // inferred from electronic annotation /// 0016477 // cell migration // inferred from electronic annotation /// 0021812 // neuronal-glial interaction involved in cerebral cortex radial glia guided migration // inferred from mutant phenotype /// 0022617 // extracellular matrix disassembly // traceable author statement /// 0030198 // [illegible]	0005576 // extracellular region // traceable author statement /// 0005578 // proteinaceous extracellular matrix // inferred from electronic annotation /// 0005604 // basement membrane // inferred from direct assay /// 0005604 // basement membrane // traceable author statement /// 0005605 // basal lamina // inferred from electronic annotation /// 0005606 // laminin-1 complex // inferred from [illegible]	0005178 // integrin binding // inferred from electronic annotation /// 0005198 // structural molecule activity // non-traceable author statement /// 0005201 // extracellular matrix structural constituent // inferred from direct assay /// 0005515 // protein binding // inferred from electronic annotation /// 0019899 // enzyme binding // inferred from electronic annotation /// 0043208 // glycosphingolipid binding // inferred from electronic annotation

201546_at	NM_004238	TRIP12	Homo sapiens	6-Oct-14	Exemplar sequence	GenBank	gb:NM_004238.1 /DEF=Homo sapiens thyroid hormone receptor interactor 12 (TRIP12), mRNA. /FEA=mRNA /GEN=TRIP12 /PROD=thyroid hormone receptor interactor 12 /DB_XREF=gi:10863902 /UG=Hs.138617 thyroid hormone receptor interactor 12 /FL=gb:NM_004238.1 gb:D28476.1	NM_004238	thyroid hormone receptor interactor 12	TRIP12	9320	NM_001284214 /// NM_001284215 /// NM_001284216 /// NM_004238 /// XM_005246954 /// XM_005246955 /// XM_005246956 /// XM_005246957 /// XM_005246958 /// XM_005246960 /// XM_005246961 /// XM_005246962 /// XM_005246963 /// XM_006712852 ///	0006281 // DNA repair // inferred from electronic annotation /// 0006974 // cellular response to DNA damage stimulus // inferred from mutant phenotype /// 0008152 // metabolic process // inferred from electronic annotation /// 0009790 // embryo development // inferred from sequence or structural similarity /// 0016567 // protein ubiquitination // inferred from electronic annotation /// 0016567 // protein ubiquitination // non-traceable author statement /// 0042787 // protein ubiquitination involved in ubiquitin-dependent protein catabolic process // not recorded /// 0042787 // protein ubiquitination involved in ubiquitin-	0005634 // nucleus // not recorded /// 0005634 // nucleus // inferred from direct assay /// 0005654 // nucleoplasm // inferred from direct assay /// 0005737 // cytoplasm // not recorded	0004842 // ubiquitin-protein transferase activity // not recorded /// 0004842 // ubiquitin-protein transferase activity // inferred from direct assay /// 0004842 // ubiquitin-protein transferase activity // inferred from mutant phenotype /// 0005488 // binding // inferred from electronic annotation /// 0005515 // protein binding // inferred from physical interaction /// 0008270 // zinc ion binding // inferred from electronic annotation /// 0016874 // ligase activity // inferred from electronic annotation /// 0046966 // thyroid hormone receptor binding // inferred from direct assay

201638_s_at	BE676642	CPSF1	Homo sapiens	6-Oct-14	Consensus sequence	GenBank	Consensus includes gb:BE676642 /FEA=EST /DB_XREF=gi:10037183 /DB_XREF=est:7f33f02.x1 /CLONE=IMAGE:3296475 /UG=Hs.83727 cleavage and polyadenylation specific factor 1, 160kD subunit /FL=gb:U37012.1 gb:AB046744.1 gb:NM_013291.1	BE676642	cleavage and polyadenylation specific factor 1, 160kDa	CPSF1	29894	NM_013291 /// XM_006716548 /// XM_006716549 /// XM_006716550	0000398 // mRNA splicing, via spliceosome // traceable author statement /// 0006366 // transcription from RNA polymerase II promoter // traceable author statement /// 0006369 // termination of RNA polymerase II transcription // traceable author statement /// 0006378 // mRNA polyadenylation // inferred from direct assay /// 0006379 // mRNA cleavage // inferred from direct assay /// 0006397 // mRNA processing // inferred from electronic annotation /// 0006406 // mRNA export from nucleus // traceable author statement /// 0008380 // RNA splicing // traceable author statement /// 0010467 // [illegible]	0005634 // nucleus // inferred from electronic annotation /// 0005654 // nucleoplasm // traceable author statement /// 0005847 // mRNA cleavage and polyadenylation specificity factor complex // inferred from direct assay	0003676 // nucleic acid binding // inferred from electronic annotation /// 0003723 // RNA binding // inferred from electronic annotation /// 0003730 // mRNA 3'-UTR binding // inferred from direct assay /// 0005515 // protein binding // inferred from physical interaction

201639_s_at	NM_013291	CPSF1	Homo sapiens	6-Oct-14	Exemplar sequence	GenBank	gb:NM_013291.1 /DEF=Homo sapiens cleavage and polyadenylation specific factor 1, 160kD subunit (CPSF1), mRNA. /FEA=mRNA /GEN=CPSF1 /PROD=cleavage and polyadenylation specific factor 1,160kD subunit /DB_XREF=gi:9558724 /UG=Hs.83727 cleavage and polyadenylation specific factor 1, 160kD subunit /FL=gb:U37012.1 [illegible]	NM_013291	cleavage and polyadenylation specific factor 1, 160kDa /// microRNA 1234 /// microRNA 6849 /// microRNA 939	CPSF1 /// MIR1234 /// MIR6849 /// MIR939	29894 /// 100126351 /// 100302196 /// 102466749	NM_013291 /// NR_030635 /// NR_031600 /// NR_106908 /// XM_006716548 /// XM_006716549 /// XM_006716550	0000398 // mRNA splicing, via spliceosome // traceable author statement /// 0006366 // transcription from RNA polymerase II promoter // traceable author statement /// 0006369 // termination of RNA polymerase II transcription // traceable author statement /// 0006378 // mRNA polyadenylation // inferred from direct assay /// 0006379 // mRNA cleavage // inferred from direct assay /// 0006397 // mRNA processing // inferred from electronic annotation /// 0006406 // mRNA export from nucleus // traceable author statement /// 0008380 // RNA splicing // traceable author statement /// 0010467 // [illegible]	0005634 // nucleus // inferred from electronic annotation /// 0005654 // nucleoplasm // traceable author statement /// 0005847 // mRNA cleavage and polyadenylation specificity factor complex // inferred from direct assay	0003676 // nucleic acid binding // inferred from electronic annotation /// 0003723 // RNA binding // inferred from electronic annotation /// 0003730 // mRNA 3'-UTR binding // inferred from direct assay /// 0005515 // protein binding // inferred from physical interaction

201693_s_at	AV733950	EGR1	Homo sapiens	6-Oct-14	Consensus sequence	GenBank	Consensus includes gb:AV733950 /FEA=EST /DB_XREF=gi:10851495 /DB_XREF=est:AV733950 /CLONE=cdAADG12 /UG=Hs.326035 early growth response 1 /FL=gb:M62829.1 gb:NM_001964.1	AV733950	early growth response 1	EGR1	1958	NM_001964	0000122 // negative regulation of transcription from RNA polymerase II promoter // inferred from electronic annotation /// 0001975 // response to amphetamine // inferred from electronic annotation /// 0006351 // transcription, DNA-templated // inferred from electronic annotation /// 0006355 // regulation of transcription, DNA-templated // inferred from electronic annotation /// 0006366 // transcription from RNA polymerase II promoter // inferred from direct assay /// 0007611 // learning or memory // inferred from electronic annotation /// 0007623 // circadian rhythm // inferred from electronic annotation /// [illegible]	0005634 // nucleus // inferred from direct assay /// 0005634 // nucleus // inferred from sequence or structural similarity /// 0005654 // nucleoplasm // traceable author statement /// 0005730 // nucleolus // inferred from direct assay /// 0005737 // cytoplasm // inferred from direct assay /// 0005737 // cytoplasm // inferred from sequence or structural similarity	0000976 // transcription regulatory region sequence-specific DNA binding // inferred from direct assay /// 0000977 // RNA polymerase II regulatory region sequence-specific DNA binding // inferred from electronic annotation /// 0000979 // RNA polymerase II core promoter sequence-specific DNA binding // inferred from electronic annotation /// 0000982 // RNA polymerase II core promoter proximal region sequence-specific DNA binding transcription factor activity // inferred from electronic annotation /// 0001077 // RNA polymerase II core promoter proximal region sequence-specific DNA binding transcription factor activity involved in positive regulation of transcription // inferred from electronic annotation /// 0003676 // [illegible]

201694_s_at	NM_001964	EGR1	Homo sapiens	6-Oct-14	Exemplar sequence	GenBank	gb:NM_001964.1 /DEF=Homo sapiens early growth response 1 (EGR1), mRNA. /FEA=mRNA /GEN=EGR1 /PROD=early growth response 1 /DB_XREF=gi:4503492 /UG=Hs.326035 early growth response 1 /FL=gb:M62829.1 gb:NM_001964.1	NM_001964	early growth response 1	EGR1	1958	NM_001964	0000122 // negative regulation of transcription from RNA polymerase II promoter // inferred from electronic annotation /// 0001975 // response to amphetamine // inferred from electronic annotation /// 0006351 // transcription, DNA-templated // inferred from electronic annotation /// 0006355 // regulation of transcription, DNA-templated // inferred from electronic annotation /// 0006366 // transcription from RNA polymerase II promoter // inferred from direct assay /// 0007611 // learning or memory // inferred from electronic annotation /// 0007623 // circadian rhythm // inferred from electronic annotation /// [illegible]	0005634 // nucleus // inferred from direct assay /// 0005634 // nucleus // inferred from sequence or structural similarity /// 0005654 // nucleoplasm // traceable author statement /// 0005730 // nucleolus // inferred from direct assay /// 0005737 // cytoplasm // inferred from direct assay /// 0005737 // cytoplasm // inferred from sequence or structural similarity	0000976 // transcription regulatory region sequence-specific DNA binding // inferred from direct assay /// 0000977 // RNA polymerase II regulatory region sequence-specific DNA binding // inferred from electronic annotation /// 0000979 // RNA polymerase II core promoter sequence-specific DNA binding // inferred from electronic annotation /// 0000982 // RNA polymerase II core promoter proximal region sequence-specific DNA binding transcription factor activity // inferred from electronic annotation /// 0001077 // RNA polymerase II core promoter proximal region sequence-specific DNA binding transcription factor activity involved in positive regulation of transcription // inferred from electronic annotation /// 0003676 // [illegible]

201801_s_at	AF079117	SLC29A1	Homo sapiens	6-Oct-14	Exemplar sequence	GenBank	gb:AF079117.1 /DEF=Homo sapiens equilibrative NBMPR-sensitive nucleoside transporter (ENT1) mRNA, complete cds. /FEA=mRNA /GEN=ENT1 /PROD=equilibrative NBMPR-sensitive nucleosidetransporter /DB_XREF=gi:3694939 /UG=Hs.25450 solute carrier family 29 (nucleoside transporters), member 1 /FL=gb:BC001382.1 [illegible]	AF079117	solute carrier family 29 (equilibrative nucleoside transporter), member 1	SLC29A1	2030	NM_001078174 /// NM_001078175 /// NM_001078176 /// NM_001078177 /// NM_004955 /// XM_005248875 /// XM_005248876 /// XM_005248878 /// XM_005248879 /// XM_005248880 /// XM_005248881 /// XM_005248882	0006139 // nucleobase-containing compound metabolic process // traceable author statement /// 0006810 // transport // inferred from electronic annotation /// 0007595 // lactation // inferred from electronic annotation /// 0015858 // nucleoside transport // inferred from direct assay /// 0015862 // uridine transport // inferred from electronic annotation /// 0030431 // sleep // inferred from electronic annotation /// 0055085 // transmembrane transport // traceable author statement /// 0060079 // regulation of excitatory postsynaptic membrane potential // inferred from electronic annotation /// 0071333 // cellular response to glucose [illegible]	0005886 // plasma membrane // traceable author statement /// 0005887 // integral component of plasma membrane // traceable author statement /// 0016020 // membrane // traceable author statement /// 0016021 // integral component of membrane // inferred from electronic annotation /// 0016323 // basolateral plasma membrane // inferred from direct assay /// 0016324 // apical [illegible]	0005337 // nucleoside transmembrane transporter activity // inferred from electronic annotation

201802_at	NM_004955	SLC29A1	Homo sapiens	6-Oct-14	Exemplar sequence	GenBank	gb:NM_004955.1 /DEF=Homo sapiens solute carrier family 29 (nucleoside transporters), member 1 (SLC29A1), mRNA. /FEA=mRNA /GEN=SLC29A1 /PROD=solute carrier family 29 (nucleosidetransporters), member 1 /DB_XREF=gi:4826715 /UG=Hs.25450 solute carrier family 29 (nucleoside transporters), member 1 /FL=gb:BC001382.1 [illegible]	NM_004955	solute carrier family 29 (equilibrative nucleoside transporter), member 1	SLC29A1	2030	NM_001078174 /// NM_001078175 /// NM_001078176 /// NM_001078177 /// NM_004955 /// XM_005248875 /// XM_005248876 /// XM_005248878 /// XM_005248879 /// XM_005248880 /// XM_005248881 /// XM_005248882	0006139 // nucleobase-containing compound metabolic process // traceable author statement /// 0006810 // transport // inferred from electronic annotation /// 0007595 // lactation // inferred from electronic annotation /// 0015858 // nucleoside transport // inferred from direct assay /// 0015862 // uridine transport // inferred from electronic annotation /// 0030431 // sleep // inferred from electronic annotation /// 0055085 // transmembrane transport // traceable author statement /// 0060079 // regulation of excitatory postsynaptic membrane potential // inferred from electronic annotation /// 0071333 // cellular response to glucose [illegible]	0005886 // plasma membrane // traceable author statement /// 0005887 // integral component of plasma membrane // traceable author statement /// 0016020 // membrane // traceable author statement /// 0016021 // integral component of membrane // inferred from electronic annotation /// 0016323 // basolateral plasma membrane // inferred from direct assay /// 0016324 // apical [illegible]	0005337 // nucleoside transmembrane transporter activity // inferred from electronic annotation

201912_s_at	NM_002094	GSPT1	Homo sapiens	6-Oct-14	Exemplar sequence	GenBank	gb:NM_002094.1 /DEF=Homo sapiens G1 to S phase transition 1 (GSPT1), mRNA. /FEA=mRNA /GEN=GSPT1 /PROD=G1 to S phase transition 1 /DB_XREF=gi:4504166 /UG=Hs.2707 G1 to S phase transition 1 /FL=gb:NM_002094.1	NM_002094	G1 to S phase transition 1	GSPT1	2935	NM_001130006 /// NM_001130007 /// NM_002094 /// XM_005255274 /// XM_005255275	0000082 // G1/S transition of mitotic cell cycle // traceable author statement /// 0000184 // nuclear-transcribed mRNA catabolic process, nonsense-mediated decay // traceable author statement /// 0006184 // GTP catabolic process // inferred from electronic annotation /// 0006184 // GTP catabolic process // traceable author statement /// 0006412 // translation // inferred from electronic annotation /// 0006415 // translational termination // inferred from mutant phenotype /// 0006479 // protein methylation // inferred from direct assay	0005622 // intracellular // non-traceable author statement	0000166 // nucleotide binding // inferred from electronic annotation /// 0003747 // translation release factor activity // inferred from mutant phenotype /// 0003924 // GTPase activity // traceable author statement /// 0005515 // protein binding // inferred from physical interaction /// 0005525 // GTP binding // inferred from electronic annotation /// 0044822 // poly(A) RNA binding // inferred from direct assay

201951_at	BF242905	ALCAM	Homo sapiens	6-Oct-14	Consensus sequence	GenBank	Consensus includes gb:BF242905 /FEA=EST /DB_XREF=gi:11156833 /DB_XREF=est:601877949F1 /CLONE=IMAGE:4106028 /UG=Hs.10247 activated leucocyte cell adhesion molecule /FL=gb:NM_001627.1 gb:L38608.1	BF242905	activated leukocyte cell adhesion molecule	ALCAM	214	NM_001243280 /// NM_001243281 /// NM_001243283 /// NM_001627	0007155 // cell adhesion // inferred from electronic annotation /// 0007165 // signal transduction // traceable author statement /// 0007411 // axon guidance // traceable author statement /// 0008045 // motor neuron axon guidance // inferred from electronic annotation	0005576 // extracellular region // inferred from electronic annotation /// 0009897 // external side of plasma membrane // inferred from electronic annotation /// 0016020 // membrane // inferred from electronic annotation /// 0016021 // integral component of membrane // inferred from electronic annotation /// 0030424 // axon // inferred from electronic annotation /// [illegible]	0005102 // receptor binding // traceable author statement /// 0005515 // protein binding // inferred from electronic annotation

201952_at	AA156721	ALCAM	Homo sapiens	6-Oct-14	Consensus sequence	GenBank	Consensus includes gb:AA156721 /FEA=EST /DB_XREF=gi:1728335 /DB_XREF=est:zl18b04.s1 /CLONE=IMAGE:502255 /UG=Hs.10247 activated leucocyte cell adhesion molecule /FL=gb:NM_001627.1 gb:L38608.1	AA156721	activated leukocyte cell adhesion molecule	ALCAM	214	NM_001243280 /// NM_001243281 /// NM_001243283 /// NM_001627	0007155 // cell adhesion // inferred from electronic annotation /// 0007165 // signal transduction // traceable author statement /// 0007411 // axon guidance // traceable author statement /// 0008045 // motor neuron axon guidance // inferred from electronic annotation	0005576 // extracellular region // inferred from electronic annotation /// 0009897 // external side of plasma membrane // inferred from electronic annotation /// 0016020 // membrane // inferred from electronic annotation /// 0016021 // integral component of membrane // inferred from electronic annotation /// 0030424 // axon // inferred from electronic annotation /// [illegible]	0005102 // receptor binding // traceable author statement /// 0005515 // protein binding // inferred from electronic annotation
202082_s_at	AV748469	SEC14L1	Homo sapiens	6-Oct-14	Consensus sequence	GenBank	Consensus includes gb:AV748469 /FEA=EST /DB_XREF=gi:10906317 /DB_XREF=est:AV748469 /CLONE=NPCAXA07 /UG=Hs.75232 SEC14 (S. cerevisiae)-like 1 /FL=gb:D67029.1 gb:NM_003003.1	AV748469	SEC14-like 1 (S. cerevisiae)	SEC14L1	6397	NM_001039573 /// NM_001143998 /// NM_001143999 /// NM_001144001 /// NM_001204408 /// NM_001204410 /// NM_003003	0006810 // transport // inferred from electronic annotation	0005794 // Golgi apparatus // traceable author statement /// 0016020 // membrane // traceable author statement /// 0016021 // integral component of membrane // inferred from electronic annotation	

202083_s_at	AI017770	SEC14L1	Homo sapiens	6-Oct-14	Consensus sequence	GenBank	Consensus includes gb:AI017770 /FEA=EST /DB_XREF=gi:3232106 /DB_XREF=est:ov32b12.x1 /CLONE=IMAGE:1639007 /UG=Hs.75232 SEC14 (S. cerevisiae)-like 1 /FL=gb:D67029.1 gb:NM_003003.1	AI017770	SEC14-like 1 (S. cerevisiae)	SEC14L1	6397	NM_001039573 /// NM_001143998 /// NM_001143999 /// NM_001144001 /// NM_001204408 /// NM_001204410 /// NM_003003	0006810 // transport // inferred from electronic annotation	0005794 // Golgi apparatus // traceable author statement /// 0016020 // membrane // traceable author statement /// 0016021 // integral component of membrane // inferred from electronic annotation	
202084_s_at	NM_003003	SEC14L1	Homo sapiens	6-Oct-14	Exemplar sequence	GenBank	gb:NM_003003.1 /DEF=Homo sapiens SEC14 (S. cerevisiae)-like 1 (SEC14L1), mRNA. /FEA=mRNA /GEN=SEC14L1 /PROD=SEC14 (S. cerevisiae)-like 1 /DB_XREF=gi:4506866 /UG=Hs.75232 SEC14 (S. cerevisiae)-like 1 /FL=gb:D67029.1 gb:NM_003003.1	NM_003003	SEC14-like 1 (S. cerevisiae)	SEC14L1	6397	NM_001039573 /// NM_001143998 /// NM_001143999 /// NM_001144001 /// NM_001204408 /// NM_001204410 /// NM_003003	0006810 // transport // inferred from electronic annotation	0005794 // Golgi apparatus // traceable author statement /// 0016020 // membrane // traceable author statement /// 0016021 // integral component of membrane // inferred from electronic annotation	

202124_s_at	AV705253	TRAK2	Homo sapiens	6-Oct-14	Consensus sequence	GenBank	Consensus includes gb:AV705253 /FEA=EST /DB_XREF=gi:10722559 /DB_XREF=est:AV705253 /CLONE=ADBBQF03 /UG=Hs.154248 amyotrophic lateral sclerosis 2 (juvenile) chromosome region, candidate 3 /FL=gb:AB038951.1 gb:NM_015049.1	AV705253	trafficking protein, kinesin binding 2	TRAK2	66008	NM_015049 /// XM_006712714	0006357 // regulation of transcription from RNA polymerase II promoter // inferred from electronic annotation /// 0006493 // protein O-linked glycosylation // inferred from electronic annotation /// 0006605 // protein targeting // inferred from electronic annotation /// 0006810 // transport // inferred from electronic annotation	0005634 // nucleus // inferred from electronic annotation /// 0005737 // cytoplasm // inferred from sequence or structural similarity /// 0005739 // mitochondrion // inferred from sequence or structural similarity /// 0005768 // endosome // inferred from electronic annotation /// 0005769 // early endosome // inferred from electronic annotation /// 0005886 // plasma [illegible]	0005102 // receptor binding // inferred from sequence or structural similarity /// 0005515 // protein binding // inferred from physical interaction /// 0019899 // enzyme binding // inferred from electronic annotation /// 0050811 // GABA receptor binding // non-traceable author statement

202125_s_at	NM_015049	TRAK2	Homo sapiens	6-Oct-14	Exemplar sequence	GenBank	gb:NM_015049.1 /DEF=Homo sapiens amyotrophic lateral sclerosis 2 (juvenile) chromosome region, candidate 3 (ALS2CR3), mRNA. /FEA=mRNA /GEN=ALS2CR3 /PROD=amyotrophic lateral sclerosis 2 (juvenile)chromosome region, candidate 3 /DB_XREF=gi:13027379 /UG=Hs.154248 amyotrophic lateral sclerosis 2 (juvenile) chromosome region, [illegible]	NM_015049	trafficking protein, kinesin binding 2	TRAK2	66008	NM_015049 /// XM_006712714	0006357 // regulation of transcription from RNA polymerase II promoter // inferred from electronic annotation /// 0006493 // protein O-linked glycosylation // inferred from electronic annotation /// 0006605 // protein targeting // inferred from electronic annotation /// 0006810 // transport // inferred from electronic annotation	0005634 // nucleus // inferred from electronic annotation /// 0005737 // cytoplasm // inferred from sequence or structural similarity /// 0005739 // mitochondrion // inferred from sequence or structural similarity /// 0005768 // endosome // inferred from electronic annotation /// 0005769 // early endosome // inferred from electronic annotation /// 0005886 // plasma [illegible]	0005102 // receptor binding // inferred from sequence or structural similarity /// 0005515 // protein binding // inferred from physical interaction /// 0019899 // enzyme binding // inferred from electronic annotation /// 0050811 // GABA receptor binding // non-traceable author statement

202165_at	BF966540	PPP1R2	Homo sapiens	6-Oct-14	Consensus sequence	GenBank	Consensus includes gb:BF966540 /FEA=EST /DB_XREF=gi:12333755 /DB_XREF=est:602287009T1 /CLONE=IMAGE:4375586 /UG=Hs.267819 protein phosphatase 1, regulatory (inhibitor) subunit 2 /FL=gb:NM_006241.1	BF966540	protein phosphatase 1, regulatory (inhibitor) subunit 2	PPP1R2	5504	NM_001291504 /// NM_001291505 /// NM_006241 /// XM_006713682 /// XM_006713683	0005975 // carbohydrate metabolic process // inferred from electronic annotation /// 0005977 // glycogen metabolic process // inferred from electronic annotation /// 0006091 // generation of precursor metabolites and energy // traceable author statement /// 0009966 // regulation of signal transduction // inferred from electronic annotation /// 0043086 // negative regulation of catalytic activity // inferred from electronic annotation /// 0043086 // negative regulation of catalytic activity // traceable author statement /// 0043666 // regulation of phosphoprotein phosphatase activity // inferred from electronic		0004864 // protein phosphatase inhibitor activity // inferred from electronic annotation /// 0004865 // protein serine/threonine phosphatase inhibitor activity // traceable author statement /// 0005515 // protein binding // inferred from physical interaction

202166_s_at	NM_006241	PPP1R2	Homo sapiens	6-Oct-14	Exemplar sequence	GenBank	gb:NM_006241.1 /DEF=Homo sapiens protein phosphatase 1, regulatory (inhibitor) subunit 2 (PPP1R2), mRNA. /FEA=mRNA /GEN=PPP1R2 /PROD=protein phosphatase 1, regulatory (inhibitor)subunit 2 /DB_XREF=gi:5453945 /UG=Hs.267819 protein phosphatase 1, regulatory (inhibitor) subunit 2 /FL=gb:NM_006241.1	NM_006241	protein phosphatase 1, regulatory (inhibitor) subunit 2	PPP1R2	5504	NM_001291504 /// NM_001291505 /// NM_006241 /// XM_006713682 /// XM_006713683	0005975 // carbohydrate metabolic process // inferred from electronic annotation /// 0005977 // glycogen metabolic process // inferred from electronic annotation /// 0006091 // generation of precursor metabolites and energy // traceable author statement /// 0009966 // regulation of signal transduction // inferred from electronic annotation /// 0043086 // negative regulation of catalytic activity // inferred from electronic annotation /// 0043086 // negative regulation of catalytic activity // traceable author statement /// 0043666 // regulation of phosphoprotein phosphatase activity // inferred from electronic		0004864 // protein phosphatase inhibitor activity // inferred from electronic annotation /// 0004865 // protein serine/threonine phosphatase inhibitor activity // traceable author statement /// 0005515 // protein binding // inferred from physical interaction

202182_at	NM_021078	KAT2A	Homo sapiens	6-Oct-14	Exemplar sequence	GenBank	gb:NM_021078.1 /DEF=Homo sapiens GCN5 (general control of amino-acid synthesis, yeast, homolog)-like 2 (GCN5L2), mRNA. /FEA=mRNA /GEN=GCN5L2 /PROD=GCN5 (general control of amino-acid synthesis,yeast, homolog)-like 2 /DB_XREF=gi:10835100 /UG=Hs.101067 GCN5 (general control of amino-acid synthesis, yeast, homolog)-like 2 /FL=gb:NM_02	NM_021078	K(lysine) acetyltransferase 2A	KAT2A	2648	NM_021078 /// XM_006721817 /// XM_006721818	0001701 // in utero embryonic development // inferred from electronic annotation /// 0001756 // somitogenesis // inferred from electronic annotation /// 0001843 // neural tube closure // inferred from electronic annotation /// 0006325 // chromatin organization // traceable author statement /// 0006338 // chromatin remodeling // traceable author statement /// 0006351 // transcription, DNA-templated // inferred from electronic annotation /// 0006355 // regulation of transcription, DNA-templated // inferred from electronic annotation /// 0006357 // regulation of transcription from RNA polymerase II	0000123 // histone acetyltransferase complex // inferred from electronic annotation /// 0005615 // extracellular space // inferred from direct assay /// 0005634 // nucleus // inferred from electronic annotation /// 0005654 // nucleoplasm // traceable author statement /// 0005671 // Ada2/Gcn5/Ada3 transcription activator complex // inferred from direct assay /// 0030914 // STAGA complex // inferred from	0003682 // chromatin binding // inferred from electronic annotation /// 0003713 // transcription coactivator activity // inferred from direct assay /// 0004402 // histone acetyltransferase activity // inferred from direct assay /// 0005515 // protein binding // inferred from physical interaction /// 0008080 // N-acetyltransferase activity // inferred from electronic annotation /// 0010484 // H3 histone acetyltransferase activity // inferred from direct assay /// 0016740 // transferase activity // inferred from electronic annotation /// 0016746 // transferase activity, transferring acyl groups // inferred from electronic annotation /// 0042826 // histone deacetylase binding // inferred from physical interaction /// 0043997 //

202241_at	NM_025195	TRIB1	Homo sapiens	6-Oct-14	Exemplar sequence	GenBank	gb:NM_025195.1 /DEF=Homo sapiens phosphoprotein regulated by mitogenic pathways (C8FW), mRNA. /FEA=mRNA /GEN=C8FW /PROD=G-protein-coupled receptor induced protein /DB_XREF=gi:13399327 /UG=Hs.7837 phosphoprotein regulated by mitogenic pathways /FL=gb:AF205437.1 gb:NM_025195.1	NM_025195	tribbles pseudokinase 1	TRIB1	10221	NM_001282985 /// NM_025195 /// XM_006716496 /// XM_006716497 /// XR_428373	0006468 // protein phosphorylation // traceable author statement /// 0006469 // negative regulation of protein kinase activity // inferred from mutant phenotype /// 0007254 // JNK cascade // inferred from mutant phenotype /// 0014912 // negative regulation of smooth muscle cell migration // inferred from mutant phenotype /// 0031665 // negative regulation of lipopolysaccharide-mediated signaling pathway // inferred from mutant phenotype /// 0032436 // positive regulation of proteasomal ubiquitin-dependent protein catabolic process // inferred from sequence or structural similarity /// 0032496 // response to [illegible]	0005634 // nucleus // inferred from direct assay /// 0005737 // cytoplasm // inferred from direct assay	0004672 // protein kinase activity // traceable author statement /// 0004860 // protein kinase inhibitor activity // inferred from mutant phenotype /// 0005524 // ATP binding // traceable author statement /// 0008134 // transcription factor binding // inferred from physical interaction /// 0016772 // transferase activity, transferring phosphorus-containing groups // inferred from electronic annotation /// 0031434 // mitogen-activated protein kinase kinase binding // inferred from direct assay /// 0031625 // ubiquitin protein ligase binding // inferred from sequence or structural similarity /// 0055106 // ubiquitin-protein transferase regulator activity // inferred from sequence or structural similarity

202275_at	NM_000402	G6PD	Homo sapiens	6-Oct-14	Exemplar sequence	GenBank	gb:NM_000402.1 /DEF=Homo sapiens glucose-6-phosphate dehydrogenase (G6PD), nuclear gene encoding mitochondrial protein, mRNA. /FEA=mRNA /GEN=G6PD /PROD=glucose-6-phosphate dehydrogenase /DB_XREF=gi:4503844 /UG=Hs.80206 glucose-6-phosphate dehydrogenase /FL=gb:BC000337.1 gb:M21248.1 gb:NM_000402.1	NM_000402	glucose-6-phosphate dehydrogenase	G6PD	2539	NM_000402 /// NM_001042351 /// XM_005274657 /// XM_005274658	0001816 // cytokine production // inferred from mutant phenotype /// 0005975 // carbohydrate metabolic process // traceable author statement /// 0006006 // glucose metabolic process // inferred from electronic annotation /// 0006098 // pentose-phosphate shunt // inferred from direct assay /// 0006098 // pentose-phosphate shunt // traceable author statement /// 0006629 // lipid metabolic process // traceable author statement /// 0006695 // cholesterol biosynthetic process // inferred from mutant phenotype /// 0006739 // NADP metabolic process // inferred from direct assay /// 0006740 // NADPH [illegible]	0005634 // nucleus // inferred from electronic annotation /// 0005737 // cytoplasm // inferred from direct assay /// 0005813 // centrosome // inferred from direct assay /// 0005829 // cytosol // inferred from direct assay /// 0005829 // cytosol // traceable author statement /// 0009898 // cytoplasmic side of plasma membrane // inferred from direct assay /// 0016020 // membrane // inferred from direct assay /// [illegible]	0004345 // glucose-6-phosphate dehydrogenase activity // inferred from direct assay /// 0004345 // glucose-6-phosphate dehydrogenase activity // inferred from mutant phenotype /// 0005515 // protein binding // inferred from physical interaction /// 0005536 // glucose binding // inferred from direct assay /// 0005536 // glucose binding // inferred from mutant phenotype /// 0016491 // oxidoreductase activity // inferred from electronic annotation /// 0030246 // carbohydrate binding // inferred from electronic annotation /// 0042803 // protein homodimerization activity // inferred from physical interaction /// 0050661 // NADP binding // inferred from direct assay

202551_s_at	BG546884	CRIM1	Homo sapiens	6-Oct-14	Consensus sequence	GenBank	Consensus includes gb:BG546884 /FEA=EST /DB_XREF=gi:13545549 /DB_XREF=est:602574066F1 /CLONE=IMAGE:4702049 /UG=Hs.19280 cysteine-rich motor neuron 1 /FL=gb:NM_016441.1 gb:AF167706.1	BG546884	cysteine rich transmembrane BMP regulator 1 (chordin-like) /// uncharacterized LOC101929500	CRIM1 /// LOC101929500	51232 /// 101929500	NM_016441 /// XM_005264357 /// XM_005264358 /// XR_244983 /// XR_249109 /// XR_251054 /// XR_426983	0001558 // regulation of cell growth // inferred from electronic annotation /// 0007399 // nervous system development // traceable author statement /// 0010951 // negative regulation of endopeptidase activity // inferred from electronic annotation /// 0043086 // negative regulation of catalytic activity // inferred from electronic annotation /// 0048009 // insulin-like growth factor receptor signaling pathway // traceable author statement	0005576 // extracellular region // inferred from electronic annotation /// 0005886 // plasma membrane // inferred from electronic annotation /// 0016020 // membrane // inferred from electronic annotation /// 0016021 // integral component of membrane // inferred from electronic annotation /// 0070062 // extracellular vesicular exosome // inferred from direct assay	0004857 // enzyme inhibitor activity // inferred from electronic annotation /// 0004867 // serine-type endopeptidase inhibitor activity // inferred from electronic annotation /// 0005010 // insulin-like growth factor-activated receptor activity // traceable author statement /// 0005515 // protein binding // inferred from electronic annotation /// 0005520 // insulin-like growth factor binding // inferred from electronic annotation /// 0030165 // PDZ domain binding // inferred from direct assay

202552_s_at	NM_016441	CRIM1	Homo sapiens	6-Oct-14	Exemplar sequence	GenBank	gb:NM_016441.1 /DEF=Homo sapiens cysteine-rich motor neuron 1 (CRIM1), mRNA. /FEA=mRNA /GEN=CRIM1 /PROD=cysteine-rich motor neuron 1 /DB_XREF=gi:10092638 /UG=Hs.19280 cysteine-rich motor neuron 1 /FL=gb:NM_016441.1 gb:AF167706.1	NM_016441	cysteine rich transmembrane BMP regulator 1 (chordin-like) /// uncharacterized LOC101929500	CRIM1 /// LOC101929500	51232 /// 101929500	NM_016441 /// XM_005264357 /// XM_005264358 /// XR_244983 /// XR_249109 /// XR_251054 /// XR_426983	0001558 // regulation of cell growth // inferred from electronic annotation /// 0007399 // nervous system development // traceable author statement /// 0010951 // negative regulation of endopeptidase activity // inferred from electronic annotation /// 0043086 // negative regulation of catalytic activity // inferred from electronic annotation /// 0048009 // insulin-like growth factor receptor signaling pathway // traceable author statement	0005576 // extracellular region // inferred from electronic annotation /// 0005886 // plasma membrane // inferred from electronic annotation /// 0016020 // membrane // inferred from electronic annotation /// 0016021 // integral component of membrane // inferred from electronic annotation /// 0070062 // extracellular vesicular exosome // inferred from direct assay	0004857 // enzyme inhibitor activity // inferred from electronic annotation /// 0004867 // serine-type endopeptidase inhibitor activity // inferred from electronic annotation /// 0005010 // insulin-like growth factor-activated receptor activity // traceable author statement /// 0005515 // protein binding // inferred from electronic annotation /// 0005520 // insulin-like growth factor binding // inferred from electronic annotation /// 0030165 // PDZ domain binding // inferred from direct assay

202573_at	AL530441	CSNK1G2	Homo sapiens	6-Oct-14	Consensus sequence	GenBank	Consensus includes gb:AL530441 /FEA=EST /DB_XREF=gi:12793934 /DB_XREF=est:AL530441 /CLONE=CS0DD007YK14 (3 prime) /UG=Hs.181390 casein kinase 1, gamma 2 /FL=gb:NM_001319.2 gb:U89896.1	AL530441	casein kinase 1, gamma 2	CSNK1G2	1455	NM_001319 /// XM_005259498 /// XM_005259499 /// XM_005259500 /// XM_005259501 /// XM_005259502 /// XR_430129	0006468 // protein phosphorylation // traceable author statement /// 0006665 // sphingolipid metabolic process // traceable author statement /// 0007165 // signal transduction // traceable author statement /// 0008152 // metabolic process // inferred from electronic annotation /// 0016055 // Wnt signaling pathway // inferred from electronic annotation /// 0016310 // phosphorylation // inferred from electronic annotation /// 0030148 // sphingolipid biosynthetic process // traceable author statement /// 0044281 // small molecule metabolic process // traceable author statement /// 0046777 //	0005737 // cytoplasm // inferred from direct assay /// 0005829 // cytosol // traceable author statement /// 0016020 // membrane // inferred from direct assay	0000166 // nucleotide binding // inferred from electronic annotation /// 0000287 // magnesium ion binding // inferred from electronic annotation /// 0001948 // glycoprotein binding // inferred from electronic annotation /// 0004672 // protein kinase activity // inferred from electronic annotation /// 0004674 // protein serine/threonine kinase activity // traceable author statement /// 0004713 // protein tyrosine kinase activity // inferred from electronic annotation /// 0005515 // protein binding // inferred from physical interaction /// 0005524 // ATP binding // inferred from electronic annotation /// 0016301 // kinase activity // inferred from electronic annotation /// 0016740 // [illegible]

202574_s_at	NM_001319	CSNK1G2	Homo sapiens	6-Oct-14	Exemplar sequence	GenBank	gb:NM_001319.2 /DEF=Homo sapiens casein kinase 1, gamma 2 (CSNK1G2), mRNA. /FEA=mRNA /GEN=CSNK1G2 /PROD=casein kinase 1, gamma 2 /DB_XREF=gi:11079647 /UG=Hs.181390 casein kinase 1, gamma 2 /FL=gb:NM_001319.2 gb:U89896.1	NM_001319	casein kinase 1, gamma 2	CSNK1G2	1455	NM_001319 /// XM_005259498 /// XM_005259499 /// XM_005259500 /// XM_005259501 /// XM_005259502 /// XR_430129	0006468 // protein phosphorylation // traceable author statement /// 0006665 // sphingolipid metabolic process // traceable author statement /// 0007165 // signal transduction // traceable author statement /// 0008152 // metabolic process // inferred from electronic annotation /// 0016055 // Wnt signaling pathway // inferred from electronic annotation /// 0016310 // phosphorylation // inferred from electronic annotation /// 0030148 // sphingolipid biosynthetic process // traceable author statement /// 0044281 // small molecule metabolic process // traceable author statement /// 0046777 //	0005737 // cytoplasm // inferred from direct assay /// 0005829 // cytosol // traceable author statement /// 0016020 // membrane // inferred from direct assay	0000166 // nucleotide binding // inferred from electronic annotation /// 0000287 // magnesium ion binding // inferred from electronic annotation /// 0001948 // glycoprotein binding // inferred from electronic annotation /// 0004672 // protein kinase activity // inferred from electronic annotation /// 0004674 // protein serine/threonine kinase activity // traceable author statement /// 0004713 // protein tyrosine kinase activity // inferred from electronic annotation /// 0005515 // protein binding // inferred from physical interaction /// 0005524 // ATP binding // inferred from electronic annotation /// 0016301 // kinase activity // inferred from electronic annotation /// 0016740 //

202887_s_at	NM_019058	DDIT4	Homo sapiens	6-Oct-14	Exemplar sequence	GenBank	gb:NM_019058.1 /DEF=Homo sapiens hypothetical protein (FLJ20500), mRNA. /FEA=mRNA /GEN=FLJ20500 /PROD=hypothetical protein /DB_XREF=gi:9506686 /UG=Hs.111244 hypothetical protein /FL=gb:AL136668.1 gb:NM_019058.1	NM_019058	DNA-damage-inducible transcript 4	DDIT4	54541	NM_019058	0001666 // response to hypoxia // inferred from direct assay /// 0001764 // neuron migration // inferred from sequence or structural similarity /// 0006915 // apoptotic process // inferred from electronic annotation /// 0007420 // brain development // inferred from sequence or structural similarity /// 0008283 // cell proliferation // inferred from sequence or structural similarity /// 0009968 // negative regulation of signal transduction // inferred from electronic annotation /// 0010801 // negative regulation of peptidyl-threonine phosphorylation // inferred from sequence or structural similarity /// [illegible]	0005622 // intracellular // inferred from direct assay /// 0005737 // cytoplasm // inferred from direct assay /// 0005739 // mitochondrion // inferred from electronic annotation /// 0005829 // cytosol // inferred from electronic annotation	0071889 // 14-3-3 protein binding // inferred from electronic annotation

202947_s_at	NM_002101	GYPC	Homo sapiens	6-Oct-14	Exemplar sequence	GenBank	gb:NM_002101.2 /DEF=Homo sapiens glycophorin C (Gerbich blood group) (GYPC), transcript variant 1, mRNA. /FEA=mRNA /GEN=GYPC /PROD=glycophorin C, isoform 1 /DB_XREF=gi:8051606 /UG=Hs.81994 glycophorin C (Gerbich blood group) /FL=gb:M36284.1 gb:M11802.1 gb:NM_002101.2	NM_002101	glycophorin C (Gerbich blood group)	GYPC	2995	NM_001256584 /// NM_002101 /// NM_016815 /// XM_005263653 /// XM_006712460		0005886 // plasma membrane // non-traceable author statement /// 0005887 // integral component of plasma membrane // traceable author statement /// 0016020 // membrane // inferred from direct assay /// 0016020 // membrane // inferred from electronic annotation /// 0016021 // integral component of membrane // inferred from electronic annotation /// 0030863 // cortical [illegible]	0005515 // protein binding // inferred from physical interaction

202988_s_at	NM_002922	RGS1	Homo sapiens	6-Oct-14	Exemplar sequence	GenBank	gb:NM_002922.1 /DEF=Homo sapiens regulator of G-protein signalling 1 (RGS1), mRNA. /FEA=mRNA /GEN=RGS1 /PROD=regulator of G-protein signalling 1 /DB_XREF=gi:4506514 /UG=Hs.75256 regulator of G-protein signalling 1 /FL=gb:NM_002922.1	NM_002922	regulator of G-protein signaling 1	RGS1	5996	NM_002922	0006955 // immune response // traceable author statement /// 0007165 // signal transduction // non-traceable author statement /// 0007193 // adenylate cyclase-inhibiting G-protein coupled receptor signaling pathway // traceable author statement /// 0009968 // negative regulation of signal transduction // inferred from electronic annotation /// 0038032 // termination of G-protein coupled receptor signaling pathway // inferred from electronic annotation /// 0043547 // positive regulation of GTPase activity // not recorded /// 0043547 // positive regulation of GTPase activity // traceable [illegible]	0005737 // cytoplasm // not recorded /// 0005886 // plasma membrane // not recorded	0005096 // GTPase activator activity // not recorded /// 0005516 // calmodulin binding // traceable author statement

202989_at	NM_002922	RGS1	Homo sapiens	6-Oct-14	Exemplar sequence	GenBank	gb:NM_002922.1 /DEF=Homo sapiens regulator of G-protein signalling 1 (RGS1), mRNA. /FEA=mRNA /GEN=RGS1 /PROD=regulator of G-protein signalling 1 /DB_XREF=gi:4506514 /UG=Hs.75256 regulator of G-protein signalling 1 /FL=gb:NM_002922.1	NM_002922	regulator of G-protein signaling 1	RGS1	5996	NM_002922	0006955 // immune response // traceable author statement /// 0007165 // signal transduction // non-traceable author statement /// 0007193 // adenylate cyclase-inhibiting G-protein coupled receptor signaling pathway // traceable author statement /// 0009968 // negative regulation of signal transduction // inferred from electronic annotation /// 0038032 // termination of G-protein coupled receptor signaling pathway // inferred from electronic annotation /// 0043547 // positive regulation of GTPase activity // not recorded /// 0043547 // positive regulation of GTPase activity // traceable [illegible]	0005737 // cytoplasm // not recorded /// 0005886 // plasma membrane // not recorded	0005096 // GTPase activator activity // not recorded /// 0005516 // calmodulin binding // traceable author statement

203117_s_at	NM_014871	PAN2	Homo sapiens	6-Oct-14	Exemplar sequence	GenBank	gb:NM_014871.1 /DEF=Homo sapiens KIAA0710 gene product (KIAA0710), mRNA. /FEA=mRNA /GEN=KIAA0710 /PROD=KIAA0710 gene product /DB_XREF=gi:7662257 /UG=Hs.273397 KIAA0710 gene product /FL=gb:AB014610.1 gb:NM_014871.1	NM_014871	PAN2 poly(A) specific ribonuclease subunit	PAN2	9924	NM_001127460 /// NM_001166279 /// NM_014871	0000184 // nuclear-transcribed mRNA catabolic process, nonsense-mediated decay // inferred from electronic annotation /// 0000288 // nuclear-transcribed mRNA catabolic process, deadenylation-dependent decay // traceable author statement /// 0000289 // nuclear-transcribed mRNA poly(A) tail shortening // traceable author statement /// 0006397 // mRNA processing // inferred from electronic annotation /// 0006511 // ubiquitin-dependent protein catabolic process // inferred from electronic annotation /// 0010467 // gene expression // traceable author statement /// 0016070 // RNA metabolic process // traceable author [illegible]	0000932 // cytoplasmic mRNA processing body // inferred from electronic annotation /// 0005634 // nucleus // inferred from electronic annotation /// 0005737 // cytoplasm // inferred from electronic annotation /// 0005829 // cytosol // traceable author statement /// 0031251 // PAN complex // inferred from direct assay	0000175 // 3'-5'-exoribonuclease activity // inferred from direct assay /// 0003676 // nucleic acid binding // inferred from electronic annotation /// 0004221 // ubiquitin thiolesterase activity // inferred from electronic annotation /// 0004518 // nuclease activity // inferred from electronic annotation /// 0004527 // exonuclease activity // inferred from electronic annotation /// 0004535 // poly(A)-specific ribonuclease activity // inferred from direct assay /// 0004535 // poly(A)-specific ribonuclease activity // traceable author statement /// 0005515 // protein binding // inferred from physical interaction /// 0016787 // hydrolase activity // inferred from electronic annotation

203289_s_at	BE791629	NPRL3	Homo sapiens	6-Oct-14	Consensus sequence	GenBank	Consensus includes gb:BE791629 /FEA=EST /DB_XREF=gi:10212827 /DB_XREF=est:601585959F1 /CLONE=IMAGE:3940215 /UG=Hs.19699 Conserved gene telomeric to alpha globin cluster /FL=gb:NM_012075.1	BE791629	nitrogen permease regulator-like 3 (S. cerevisiae)	NPRL3	8131	NM_001039476 /// NM_001077350 /// NM_001243247 /// NM_001243248 /// NM_001243249 /// NM_012075	0003281 // ventricular septum development // inferred from electronic annotation /// 0035909 // aorta morphogenesis // inferred from electronic annotation /// 0043547 // positive regulation of GTPase activity // inferred from electronic annotation /// 0048738 // cardiac muscle tissue development // inferred from electronic annotation /// 0060021 // palate development // inferred from electronic annotation		0005096 // GTPase activator activity // inferred from electronic annotation

203290_at	NM_002122	HLA-DQA1	Homo sapiens	6-Oct-14	Exemplar sequence	GenBank	gb:NM_002122.1 /DEF=Homo sapiens major histocompatibility complex, class II, DQ alpha 1 (HLA-DQA1), mRNA. /FEA=mRNA /GEN=HLA-DQA1 /PROD=major histocompatibility complex, class II, DQalpha 1 /DB_XREF=gi:4504406 /UG=Hs.198253 major histocompatibility complex, class II, DQ alpha 1 /FL=gb:M33906.1 gb:M17846.1 gb:M17847.1 [illegible]	NM_002122	major histocompatibility complex, class II, DQ alpha 1	HLA-DQA1	3117	NM_002122 /// XM_003846468 /// XM_005274953 /// XM_005275108 /// XM_005275333 /// XM_005275542 /// XM_005275544 /// XM_006715079 /// XM_006725484 /// XM_006725999	0002376 // immune system process // inferred from electronic annotation /// 0002504 // antigen processing and presentation of peptide or polysaccharide antigen via MHC class II // inferred from electronic annotation /// 0006955 // immune response // non-traceable author statement /// 0019221 // cytokine-mediated signaling pathway // traceable author statement /// 0019882 // antigen processing and presentation // inferred from electronic annotation /// 0019886 // antigen processing and presentation of exogenous peptide antigen via MHC class II // traceable author statement /// 0031295 // T cell costimulation // [illegible]	0000139 // Golgi membrane // traceable author statement /// 0005764 // lysosome // inferred from electronic annotation /// 0005765 // lysosomal membrane // traceable author statement /// 0005768 // endosome // inferred from electronic annotation /// 0005783 // endoplasmic reticulum // inferred from electronic annotation /// 0005789 // endoplasmic [illegible]	0005515 // protein binding // inferred from electronic annotation /// 0032395 // MHC class II receptor activity // non-traceable author statement /// 0032395 // MHC class II receptor activity // traceable author statement /// 0042605 // peptide antigen binding // inferred from sequence or structural similarity

203314_at	NM_012227	GTPBP6	Homo sapiens	6-Oct-14	Exemplar sequence	GenBank	gb:NM_012227.1 /DEF=Homo sapiens Pseudoautosomal GTP-binding protein-like (PGPL), mRNA. /FEA=mRNA /GEN=PGPL /PROD=Pseudoautosomal GTP-binding protein-likeprotein /DB_XREF=gi:6912587 /UG=Hs.101033 Pseudoautosomal GTP-binding protein-like /FL=gb:NM_012227.1	NM_012227	GTP binding protein 6 (putative)	GTPBP6	8225	NM_012227 /// XM_006724447 /// XM_006724868	0015684 // ferrous iron transport // inferred from electronic annotation	0016021 // integral component of membrane // inferred from electronic annotation	0000166 // nucleotide binding // inferred from electronic annotation /// 0005525 // GTP binding // inferred from electronic annotation /// 0015093 // ferrous iron transmembrane transporter activity // inferred from electronic annotation /// 0046872 // metal ion binding // inferred from electronic annotation

203729_at	NM_001425	EMP3	Homo sapiens	6-Oct-14	Exemplar sequence	GenBank	gb:NM_001425.1 /DEF=Homo sapiens epithelial membrane protein 3 (EMP3), mRNA. /FEA=mRNA /GEN=EMP3 /PROD=epithelial membrane protein 3 /DB_XREF=gi:4503562 /UG=Hs.9999 epithelial membrane protein 3 /FL=gb:U52101.1 gb:U87947.1 gb:NM_001425.1	NM_001425	epithelial membrane protein 3	EMP3	2014	NM_001425	0008285 // negative regulation of cell proliferation // traceable author statement /// 0016049 // cell growth // inferred from electronic annotation	0016020 // membrane // inferred from electronic annotation /// 0016021 // integral component of membrane // inferred from electronic annotation	

203865_s_at	NM_015833	ADARB1	Homo sapiens	6-Oct-14	Exemplar sequence	GenBank	gb:NM_015833.1 /DEF=Homo sapiens adenosine deaminase, RNA-specific, B1 (homolog of rat RED1) (ADARB1), transcript variant DRABA2b, mRNA. /FEA=mRNA /GEN=ADARB1 /PROD=RNA-specific adenosine deaminase B1, isoformDRABA2b /DB_XREF=gi:7669476 /UG=Hs.85302 adenosine deaminase, RNA-specific, [illegible]	NM_015833	adenosine deaminase, RNA-specific, B1	ADARB1	104	NM_001033049 /// NM_001112 /// NM_001160230 /// NM_015833 /// NM_015834 /// NR_027672 /// NR_027673 /// NR_027674 /// NR_073200 /// XM_006723953 /// XM_006723954 /// XM_006723955 /// XM_006723956 /// XM_006723957 /// XM_006723958	0002376 // immune system process // inferred from electronic annotation /// 0006382 // adenosine to inosine editing // inferred from direct assay /// 0006382 // adenosine to inosine editing // traceable author statement /// 0006396 // RNA processing // inferred from direct assay /// 0006397 // mRNA processing // inferred from electronic annotation /// 0008285 // negative regulation of cell proliferation // inferred from direct assay /// 0010467 // gene expression // traceable author statement /// 0016553 // base conversion or substitution editing // inferred by curator /// 0016556 // mRNA modification // [illegible]	0005634 // nucleus // inferred from direct assay /// 0005654 // nucleoplasm // traceable author statement /// 0005730 // nucleolus // inferred from direct assay	0003723 // RNA binding // inferred from direct assay /// 0003725 // double-stranded RNA binding // inferred from direct assay /// 0003726 // double-stranded RNA adenosine deaminase activity // inferred from direct assay /// 0003729 // mRNA binding // traceable author statement /// 0004000 // adenosine deaminase activity // inferred from electronic annotation /// 0005515 // protein binding // inferred from physical interaction /// 0016787 // hydrolase activity // inferred from electronic annotation /// 0044822 // poly(A) RNA binding // inferred from direct assay /// 0046872 // metal ion binding // inferred from electronic annotation

203939_at	NM_002526	NT5E	Homo sapiens	6-Oct-14	Exemplar sequence	GenBank	gb:NM_002526.1 /DEF=Homo sapiens 5 nucleotidase (CD73) (NT5), mRNA. /FEA=mRNA /GEN=NT5 /PROD=5 nucleotidase /DB_XREF=gi:4505466 /UG=Hs.153952 5 nucleotidase (CD73) /FL=gb:NM_002526.1	NM_002526	5'-nucleotidase, ecto (CD73)	NT5E	4907	NM_001204813 /// NM_002526	0006144 // purine nucleobase metabolic process // traceable author statement /// 0006195 // purine nucleotide catabolic process // traceable author statement /// 0006196 // AMP catabolic process // inferred from electronic annotation /// 0006206 // pyrimidine nucleobase metabolic process // traceable author statement /// 0006259 // DNA metabolic process // traceable author statement /// 0009166 // nucleotide catabolic process // inferred from electronic annotation /// 0016311 // dephosphorylation // not recorded /// 0016311 // dephosphorylation // inferred from electronic [illegible]	0005737 // cytoplasm // inferred from direct assay /// 0005886 // plasma membrane // inferred from direct assay /// 0005886 // plasma membrane // traceable author statement /// 0016020 // membrane // traceable author statement /// 0031225 // anchored component of membrane // inferred from electronic annotation /// 0070062 // extracellular vesicular exosome // inferred from [illegible]	0000166 // nucleotide binding // inferred from electronic annotation /// 0008253 // 5'-nucleotidase activity // not recorded /// 0016787 // hydrolase activity // inferred from electronic annotation /// 0016788 // hydrolase activity, acting on ester bonds // inferred from electronic annotation /// 0046872 // metal ion binding // inferred from electronic annotation

203953_s_at	BE791251	CLDN3	Homo sapiens	6-Oct-14	Consensus sequence	GenBank	Consensus includes gb:BE791251 /FEA=EST /DB_XREF=gi:10212449 /DB_XREF=est:601583966F1 /CLONE=IMAGE:3938249 /UG=Hs.25640 claudin 3 /FL=gb:AB000714.1 gb:NM_001306.1	BE791251	claudin 3	CLDN3	1365	NM_001306	0001666 // response to hypoxia // inferred from expression pattern /// 0007165 // signal transduction // traceable author statement /// 0016338 // calcium-independent cell-cell adhesion // inferred from electronic annotation	0005886 // plasma membrane // inferred from electronic annotation /// 0005887 // integral component of plasma membrane // traceable author statement /// 0005923 // tight junction // inferred from electronic annotation /// 0016020 // membrane // inferred from electronic annotation /// 0016021 // integral component of membrane // traceable author statement /// 0030054 // cell [illegible]	0004888 // transmembrane signaling receptor activity // traceable author statement /// 0005198 // structural molecule activity // inferred from electronic annotation /// 0005515 // protein binding // inferred from electronic annotation /// 0042802 // identical protein binding // inferred from electronic annotation

203954_x_at	NM_001306	CLDN3	Homo sapiens	6-Oct-14	Exemplar sequence	GenBank	gb:NM_001306.1 /DEF=Homo sapiens claudin 3 (CLDN3), mRNA. /FEA=mRNA /GEN=CLDN3 /PROD=claudin 3 /DB_XREF=gi:4502874 /UG=Hs.25640 claudin 3 /FL=gb:AB000714.1 gb:NM_001306.1	NM_001306	claudin 3	CLDN3	1365	NM_001306	0001666 // response to hypoxia // inferred from expression pattern /// 0007165 // signal transduction // traceable author statement /// 0016338 // calcium-independent cell-cell adhesion // inferred from electronic annotation	0005886 // plasma membrane // inferred from electronic annotation /// 0005887 // integral component of plasma membrane // traceable author statement /// 0005923 // tight junction // inferred from electronic annotation /// 0016020 // membrane // inferred from electronic annotation /// 0016021 // integral component of membrane // traceable author statement /// 0030054 // cell [illegible]	0004888 // transmembrane signaling receptor activity // traceable author statement /// 0005198 // structural molecule activity // inferred from electronic annotation /// 0005515 // protein binding // inferred from electronic annotation /// 0042802 // identical protein binding // inferred from electronic annotation

204087_s_at	NM_021095	SLC5A6	Homo sapiens	6-Oct-14	Exemplar sequence	GenBank	gb:NM_021095.1 /DEF=Homo sapiens solute carrier family 5 (sodium-dependent vitamin transporter), member 6 (SLC5A6), mRNA. /FEA=mRNA /GEN=SLC5A6 /PROD=solute carrier family 5 (sodium-dependentvitamin transporter), member 6 /DB_XREF=gi:10863878 /UG=Hs.321579 solute carrier family 5 (sodium-dependent	NM_021095	solute carrier family 5 (sodium/multivitamin and iodide cotransporter), member 6	SLC5A6	8884	NM_021095 /// NR_028323 /// XM_006712128 /// XM_006712129 /// XM_006712130 /// XM_006712131	0006766 // vitamin metabolic process // traceable author statement /// 0006767 // water-soluble vitamin metabolic process // traceable author statement /// 0006768 // biotin metabolic process // traceable author statement /// 0006810 // transport // traceable author statement /// 0006811 // ion transport // inferred from electronic annotation /// 0006814 // sodium ion transport // inferred from electronic annotation /// 0015878 // biotin transport // inferred from electronic annotation /// 0015887 // pantothenate transmembrane transport // inferred from electronic annotation /// 0015939 // pantothenate metabolic	0005886 // plasma membrane // traceable author statement /// 0005887 // integral component of plasma membrane // traceable author statement /// 0012506 // vesicle membrane // inferred from electronic annotation /// 0016020 // membrane // traceable author statement /// 0016021 // integral component of membrane // inferred from electronic annotation /// 0031526 // brush	0005215 // transporter activity // inferred from electronic annotation /// 0008523 // sodium-dependent multivitamin transmembrane transporter activity // inferred from electronic annotation /// 0015293 // symporter activity // inferred from electronic annotation

204131_s_at	N25732	FOXO3	Homo sapiens	6-Oct-14	Consensus sequence	GenBank	Consensus includes gb:N25732 /FEA=EST /DB_XREF=gi:1140080 /DB_XREF=est:yx83c03.s1 /CLONE=IMAGE:268324 /UG=Hs.14845 forkhead box O3A /FL=gb:AF032886.1 gb:NM_001455.1	N25732	forkhead box O3	FOXO3	2309	NM_001455 /// NM_201559 /// XM_005266867 /// XM_005266868	0000122 // negative regulation of transcription from RNA polymerase II promoter // inferred from mutant phenotype /// 0001542 // ovulation from ovarian follicle // inferred from electronic annotation /// 0001544 // initiation of primordial ovarian follicle growth // inferred from electronic annotation /// 0001547 // antral ovarian follicle growth // inferred from electronic annotation /// 0001556 // oocyte maturation // inferred from electronic annotation /// 0006351 // transcription, DNA-templated // inferred from electronic annotation /// 0006355 // regulation of transcription, DNA-templated // inferred [illegible]	0005634 // nucleus // inferred from direct assay /// 0005654 // nucleoplasm // traceable author statement /// 0005737 // cytoplasm // inferred from direct assay /// 0005829 // cytosol // not recorded /// 0016020 // membrane // inferred from electronic annotation	0001047 // core promoter binding // inferred from sequence or structural similarity /// 0003677 // DNA binding // inferred from direct assay /// 0003700 // sequence-specific DNA binding transcription factor activity // inferred from direct assay /// 0005515 // protein binding // inferred from physical interaction /// 0008301 // DNA binding, bending // not recorded /// 0019901 // protein kinase binding // inferred from physical interaction /// 0031490 // chromatin DNA binding // inferred from sequence or structural similarity /// 0043565 // sequence-specific DNA binding // inferred from direct assay

204132_s_at	NM_001455	FOXO3	Homo sapiens	6-Oct-14	Exemplar sequence	GenBank	gb:NM_001455.1 /DEF=Homo sapiens forkhead box O3A (FOXO3A), mRNA. /FEA=mRNA /GEN=FOXO3A /PROD=forkhead box O3A /DB_XREF=gi:4503738 /UG=Hs.14845 forkhead box O3A /FL=gb:AF032886.1 gb:NM_001455.1	NM_001455	forkhead box O3 /// forkhead box O3B pseudogene	FOXO3 /// FOXO3B	2309 /// 2310	NM_001455 /// NM_201559 /// NR_026718 /// XM_005266867 /// XM_005266868	0000122 // negative regulation of transcription from RNA polymerase II promoter // inferred from mutant phenotype /// 0001542 // ovulation from ovarian follicle // inferred from electronic annotation /// 0001544 // initiation of primordial ovarian follicle growth // inferred from electronic annotation /// 0001547 // antral ovarian follicle growth // inferred from electronic annotation /// 0001556 // oocyte maturation // inferred from electronic annotation /// 0006351 // transcription, DNA-templated // inferred from electronic annotation /// 0006355 // regulation of transcription, DNA-templated // inferred [illegible]	0005634 // nucleus // inferred from direct assay /// 0005654 // nucleoplasm // traceable author statement /// 0005737 // cytoplasm // inferred from direct assay /// 0005829 // cytosol // not recorded /// 0016020 // membrane // inferred from electronic annotation	0001047 // core promoter binding // inferred from sequence or structural similarity /// 0003677 // DNA binding // inferred from direct assay /// 0003700 // sequence-specific DNA binding transcription factor activity // inferred from direct assay /// 0005515 // protein binding // inferred from physical interaction /// 0008301 // DNA binding, bending // not recorded /// 0019901 // protein kinase binding // inferred from physical interaction /// 0031490 // chromatin DNA binding // inferred from sequence or structural similarity /// 0043565 // sequence-specific DNA binding // inferred from direct assay

204141_at	NM_001069	TUBB2A	Homo sapiens	6-Oct-14	Exemplar sequence	GenBank	gb:NM_001069.1 /DEF=Homo sapiens tubulin, beta polypeptide (TUBB), mRNA. /FEA=mRNA /GEN=TUBB /PROD=tubulin, beta polypeptide /DB_XREF=gi:4507728 /UG=Hs.179661 tubulin, beta polypeptide /FL=gb:BC001194.1 gb:NM_001069.1	NM_001069	tubulin, beta 2A class IIa	TUBB2A	7280	NM_001069	0006184 // GTP catabolic process // inferred from electronic annotation /// 0006457 // protein folding // traceable author statement /// 0007017 // microtubule-based process // inferred from electronic annotation /// 0044267 // cellular protein metabolic process // traceable author statement /// 0051084 // 'de novo' posttranslational protein folding // traceable author statement /// 0051258 // protein polymerization // inferred from electronic annotation	0005634 // nucleus // inferred from direct assay /// 0005737 // cytoplasm // inferred from electronic annotation /// 0005856 // cytoskeleton // inferred from electronic annotation /// 0005874 // microtubule // inferred from direct assay /// 0005886 // plasma membrane // inferred from direct assay /// 0015630 // microtubule cytoskeleton // inferred from direct assay /// 0043234 // protein [illegible]	0000166 // nucleotide binding // inferred from electronic annotation /// 0003924 // GTPase activity // inferred from electronic annotation /// 0005200 // structural constituent of cytoskeleton // inferred from electronic annotation /// 0005525 // GTP binding // inferred from electronic annotation

204285_s_at	AI857639	PMAIP1	Homo sapiens	6-Oct-14	Consensus sequence	GenBank	Consensus includes gb:AI857639 /FEA=EST /DB_XREF=gi:5511255 /DB_XREF=est:wk95g09.x1 /CLONE=IMAGE:2423200 /UG=Hs.96 phorbol-12-myristate-13-acetate-induced protein 1 /FL=gb:NM_021127.1	AI857639	phorbol-12-myristate-13-acetate-induced protein 1	PMAIP1	5366	NM_021127	0001836 // release of cytochrome c from mitochondria // inferred from electronic annotation /// 0006915 // apoptotic process // inferred from mutant phenotype /// 0006915 // apoptotic process // traceable author statement /// 0006919 // activation of cysteine-type endopeptidase activity involved in apoptotic process // inferred from electronic annotation /// 0006974 // cellular response to DNA damage stimulus // inferred from electronic annotation /// 0008630 // intrinsic apoptotic signaling pathway in response to DNA damage // inferred from electronic annotation /// 0009411 // response to UV // inferred [illegible]	0005634 // nucleus // inferred from direct assay /// 0005739 // mitochondrion // inferred from direct assay /// 0005741 // mitochondrial outer membrane // traceable author statement /// 0005829 // cytosol // inferred from direct assay /// 0005829 // cytosol // traceable author statement	0005515 // protein binding // inferred from physical interaction

204286_s_at	NM_021127	PMAIP1	Homo sapiens	6-Oct-14	Exemplar sequence	GenBank	gb:NM_021127.1 /DEF=Homo sapiens phorbol-12-myristate-13-acetate-induced protein 1 (PMAIP1), mRNA. /FEA=mRNA /GEN=PMAIP1 /PROD=phorbol-12-myristate-13-acetate-induced protein1 /DB_XREF=gi:10863922 /UG=Hs.96 phorbol-12-myristate-13-acetate-induced protein 1 /FL=gb:NM_021127.1	NM_021127	phorbol-12-myristate-13-acetate-induced protein 1	PMAIP1	5366	NM_021127	0001836 // release of cytochrome c from mitochondria // inferred from electronic annotation /// 0006915 // apoptotic process // inferred from mutant phenotype /// 0006915 // apoptotic process // traceable author statement /// 0006919 // activation of cysteine-type endopeptidase activity involved in apoptotic process // inferred from electronic annotation /// 0006974 // cellular response to DNA damage stimulus // inferred from electronic annotation /// 0008630 // intrinsic apoptotic signaling pathway in response to DNA damage // inferred from electronic annotation /// 0009411 // response to UV // inferred [illegible]	0005634 // nucleus // inferred from direct assay /// 0005739 // mitochondrion // inferred from direct assay /// 0005741 // mitochondrial outer membrane // traceable author statement /// 0005829 // cytosol // inferred from direct assay /// 0005829 // cytosol // traceable author statement	0005515 // protein binding // inferred from physical interaction

204326_x_at	NM_002450	MT1X	Homo sapiens	6-Oct-14	Exemplar sequence	GenBank	gb:NM_002450.1 /DEF=Homo sapiens metallothionein 1L (MT1L), mRNA. /FEA=mRNA /GEN=MT1L /PROD=metallothionein 1L /DB_XREF=gi:4505270 /UG=Hs.94360 metallothionein 1L /FL=gb:NM_002450.1	NM_002450	metallothionein 1X	MT1X	4501	NM_005952	0010038 // response to metal ion // traceable author statement /// 0036018 // cellular response to erythropoietin // inferred from expression pattern /// 0045926 // negative regulation of growth // inferred from sequence or structural similarity /// 0071276 // cellular response to cadmium ion // inferred from expression pattern /// 0071294 // cellular response to zinc ion // inferred from expression pattern	0005634 // nucleus // inferred from direct assay /// 0005737 // cytoplasm // inferred from direct assay /// 0048471 // perinuclear region of cytoplasm // inferred from sequence or structural similarity	0008270 // zinc ion binding // inferred from sequence or structural similarity /// 0046872 // metal ion binding // traceable author statement

204352_at	NM_004619	TRAF5	Homo sapiens	6-Oct-14	Exemplar sequence	GenBank	gb:NM_004619.1 /DEF=Homo sapiens TNF receptor-associated factor 5 (TRAF5), mRNA. /FEA=mRNA /GEN=TRAF5 /PROD=TNF receptor-associated factor 5 /DB_XREF=gi:11321602 /UG=Hs.29736 TNF receptor-associated factor 5 /FL=gb:NM_004619.1 gb:AB000509.1	NM_004619	TNF receptor-associated factor 5	TRAF5	7188	NM_001033910 /// NM_004619 /// NM_145759 /// XM_005273249 /// XM_006711524	0006915 // apoptotic process // inferred from electronic annotation /// 0007165 // signal transduction // inferred from electronic annotation /// 0008284 // positive regulation of cell proliferation // inferred from electronic annotation /// 0016567 // protein ubiquitination // inferred from electronic annotation /// 0042981 // regulation of apoptotic process // inferred from electronic annotation /// 0043123 // positive regulation of I-kappaB kinase/NF-kappaB signaling // inferred from expression pattern /// 0051091 // positive regulation of sequence-specific DNA binding transcription factor activity // inferred from [illegible]	0005737 // cytoplasm // inferred from direct assay /// 0005813 // centrosome // inferred from direct assay /// 0005829 // cytosol // inferred from direct assay /// 0009898 // cytoplasmic side of plasma membrane // inferred from sequence or structural similarity /// 0035631 // CD40 receptor complex // inferred from sequence or structural similarity	0004842 // ubiquitin-protein transferase activity // inferred from electronic annotation /// 0004871 // signal transducer activity // inferred from electronic annotation /// 0005515 // protein binding // inferred from physical interaction /// 0008270 // zinc ion binding // inferred from electronic annotation /// 0031625 // ubiquitin protein ligase binding // inferred from physical interaction /// 0031996 // thioesterase binding // inferred from physical interaction /// 0046872 // metal ion binding // inferred from electronic annotation

204364_s_at	BE535746	REEP1	Homo sapiens	6-Oct-14	Consensus sequence	GenBank	Consensus includes gb:BE535746 /FEA=EST /DB_XREF=gi:9764391 /DB_XREF=est:601060419F1 /CLONE=IMAGE:3446788 /UG=Hs.7358 hypothetical protein FLJ13110 /FL=gb:NM_022912.1	BE535746	receptor accessory protein 1	REEP1	65055	NM_001164730 /// NM_001164731 /// NM_001164732 /// NM_022912 /// XM_005264502 /// XM_005264503 /// XM_005264504 /// XM_005264505 /// XM_006712078	0008219 // cell death // inferred from electronic annotation /// 0051205 // protein insertion into membrane // inferred from direct assay /// 0071786 // endoplasmic reticulum tubular network organization // inferred from mutant phenotype	0005737 // cytoplasm // inferred from direct assay /// 0005739 // mitochondrion // inferred from electronic annotation /// 0005783 // endoplasmic reticulum // inferred from direct assay /// 0016020 // membrane // inferred from direct assay /// 0016021 // integral component of membrane // inferred from electronic annotation /// 0031966 // mitochondrial [illegible]	0005515 // protein binding // inferred from physical interaction /// 0008017 // microtubule binding // inferred from direct assay /// 0031849 // olfactory receptor binding // inferred from mutant phenotype

204365_s_at	NM_022912	REEP1	Homo sapiens	6-Oct-14	Exemplar sequence	GenBank	gb:NM_022912.1 /DEF=Homo sapiens hypothetical protein FLJ13110 (FLJ13110), mRNA. /FEA=mRNA /GEN=FLJ13110 /PROD=hypothetical protein FLJ13110 /DB_XREF=gi:12597656 /UG=Hs.7358 hypothetical protein FLJ13110 /FL=gb:NM_022912.1	NM_022912	receptor accessory protein 1	REEP1	65055	NM_001164730 /// NM_001164731 /// NM_001164732 /// NM_022912 /// XM_005264502 /// XM_005264503 /// XM_005264504 /// XM_005264505 /// XM_006712078	0008219 // cell death // inferred from electronic annotation /// 0051205 // protein insertion into membrane // inferred from direct assay /// 0071786 // endoplasmic reticulum tubular network organization // inferred from mutant phenotype	0005737 // cytoplasm // inferred from direct assay /// 0005739 // mitochondrion // inferred from electronic annotation /// 0005783 // endoplasmic reticulum // inferred from direct assay /// 0016020 // membrane // inferred from direct assay /// 0016021 // integral component of membrane // inferred from electronic annotation /// 0031966 // mitochondrial [illegible]	0005515 // protein binding // inferred from physical interaction /// 0008017 // microtubule binding // inferred from direct assay /// 0031849 // olfactory receptor binding // inferred from mutant phenotype

204376_at	NM_014703	VPRBP	Homo sapiens	6-Oct-14	Exemplar sequence	GenBank	gb:NM_014703.1 /DEF=Homo sapiens KIAA0800 gene product (KIAA0800), mRNA. /FEA=mRNA /GEN=KIAA0800 /PROD=KIAA0800 gene product /DB_XREF=gi:7662315 /UG=Hs.118738 KIAA0800 gene product /FL=gb:AB018343.1 gb:NM_014703.1	NM_014703	Vpr (HIV-1) binding protein	VPRBP	9730	NM_001171904 /// NM_014703 /// XM_005276751 /// XM_005276752 /// XM_005276753 /// XM_005276754 /// XM_005276755 /// XM_006713426 /// XM_006713427 /// XM_006713428 /// XR_254197 /// XR_254198 /// XR_427298 /// XR_427299	0000122 // negative regulation of transcription from RNA polymerase II promoter // inferred from direct assay /// 0006351 // transcription, DNA-templated // inferred from electronic annotation /// 0006355 // regulation of transcription, DNA-templated // inferred from electronic annotation /// 0016032 // viral process // inferred from electronic annotation /// 0016310 // phosphorylation // inferred from electronic annotation /// 0016567 // protein ubiquitination // inferred from electronic annotation /// 0016568 // chromatin modification // inferred from electronic annotation /// 0030183 // B cell differentiation // [illegible]	0005634 // nucleus // inferred from direct assay /// 0005737 // cytoplasm // inferred from electronic annotation /// 0008180 // COP9 signalosome // inferred from direct assay	0000166 // nucleotide binding // inferred from electronic annotation /// 0004674 // protein serine/threonine kinase activity // inferred from electronic annotation /// 0005488 // binding // inferred from electronic annotation /// 0005515 // protein binding // inferred from physical interaction /// 0005524 // ATP binding // inferred from electronic annotation /// 0016301 // kinase activity // inferred from electronic annotation /// 0016740 // transferase activity // inferred from electronic annotation /// 1990244 // histone kinase activity (H2A-T120 specific) // inferred from direct assay

204377_s_at	NM_014703	VPRBP	Homo sapiens	6-Oct-14	Exemplar sequence	GenBank	gb:NM_014703.1 /DEF=Homo sapiens KIAA0800 gene product (KIAA0800), mRNA. /FEA=mRNA /GEN=KIAA0800 /PROD=KIAA0800 gene product /DB_XREF=gi:7662315 /UG=Hs.118738 KIAA0800 gene product /FL=gb:AB018343.1 gb:NM_014703.1	NM_014703	Vpr (HIV-1) binding protein	VPRBP	9730	NM_001171904 /// NM_014703 /// XM_005276751 /// XM_005276752 /// XM_005276753 /// XM_005276754 /// XM_005276755 /// XM_006713426 /// XM_006713427 /// XM_006713428 /// XR_254197 /// XR_254198 /// XR_427298 /// XR_427299	0000122 // negative regulation of transcription from RNA polymerase II promoter // inferred from direct assay /// 0006351 // transcription, DNA-templated // inferred from electronic annotation /// 0006355 // regulation of transcription, DNA-templated // inferred from electronic annotation /// 0016032 // viral process // inferred from electronic annotation /// 0016310 // phosphorylation // inferred from electronic annotation /// 0016567 // protein ubiquitination // inferred from electronic annotation /// 0016568 // chromatin modification // inferred from electronic annotation /// 0030183 // B cell differentiation // [illegible]	0005634 // nucleus // inferred from direct assay /// 0005737 // cytoplasm // inferred from electronic annotation /// 0008180 // COP9 signalosome // inferred from direct assay	0000166 // nucleotide binding // inferred from electronic annotation /// 0004674 // protein serine/threonine kinase activity // inferred from electronic annotation /// 0005488 // binding // inferred from electronic annotation /// 0005515 // protein binding // inferred from physical interaction /// 0005524 // ATP binding // inferred from electronic annotation /// 0016301 // kinase activity // inferred from electronic annotation /// 0016740 // transferase activity // inferred from electronic annotation /// 1990244 // histone kinase activity (H2A-T120 specific) // inferred from direct assay

204688_at	NM_003919	SGCE	Homo sapiens	6-Oct-14	Exemplar sequence	GenBank	gb:NM_003919.1 /DEF=Homo sapiens sarcoglycan, epsilon (SGCE), mRNA. /FEA=mRNA /GEN=SGCE /PROD=sarcoglycan, epsilon /DB_XREF=gi:10835046 /UG=Hs.110708 sarcoglycan, epsilon /FL=gb:NM_003919.1 gb:AF036364.1	NM_003919	sarcoglycan, epsilon	SGCE	8910	NM_001099400 /// NM_001099401 /// NM_003919 /// XM_005250675 /// XM_005250677 /// XM_005250679 /// XM_006716165 /// XM_006716166 /// XM_006716167 /// XR_428187	0007160 // cell-matrix adhesion // traceable author statement /// 0007517 // muscle organ development // traceable author statement	0005737 // cytoplasm // inferred from electronic annotation /// 0005794 // Golgi apparatus // inferred from sequence or structural similarity /// 0005856 // cytoskeleton // inferred from electronic annotation /// 0005886 // plasma membrane // inferred from sequence or structural similarity /// 0005887 // integral component of plasma membrane // traceable author [illegible]	0005509 // calcium ion binding // inferred from electronic annotation

204747_at	NM_001549	IFIT3	Homo sapiens	6-Oct-14	Exemplar sequence	GenBank	gb:NM_001549.1 /DEF=Homo sapiens interferon-induced protein with tetratricopeptide repeats 4 (IFIT4), mRNA. /FEA=mRNA /GEN=IFIT4 /PROD=interferon-induced protein withtetratricopeptide repeats 4 /DB_XREF=gi:4504586 /UG=Hs.181874 interferon-induced protein with tetratricopeptide repeats 4 /FL=gb:BC001383.1	NM_001549	interferon-induced protein with tetratricopeptide repeats 3	IFIT3	3437	NM_001031683 /// NM_001289758 /// NM_001289759 /// NM_001549	0002376 // immune system process // inferred from electronic annotation /// 0008285 // negative regulation of cell proliferation // inferred from direct assay /// 0009615 // response to virus // inferred from mutant phenotype /// 0019221 // cytokine-mediated signaling pathway // traceable author statement /// 0035457 // cellular response to interferon-alpha // inferred from electronic annotation /// 0043066 // negative regulation of apoptotic process // inferred from direct assay /// 0045087 // innate immune response // inferred from electronic annotation /// 0051607 // defense response to virus //	0005737 // cytoplasm // inferred from direct assay /// 0005739 // mitochondrion // inferred from direct assay /// 0005829 // cytosol // traceable author statement	0005515 // protein binding // inferred from physical interaction /// 0042802 // identical protein binding // inferred from physical interaction

204748_at	NM_000963	PTGS2	Homo sapiens	6-Oct-14	Exemplar sequence	GenBank	gb:NM_000963.1 /DEF=Homo sapiens prostaglandin-endoperoxide synthase 2 (prostaglandin GH synthase and cyclooxygenase) (PTGS2), mRNA. /FEA=mRNA /GEN=PTGS2 /PROD=prostaglandin-endoperoxide synthase 2(prostaglandin GH synthase and cyclooxygenase) /DB_XREF=gi:4506264 /UG=Hs.196384 prostaglandin-	NM_000963	prostaglandin-endoperoxide synthase 2 (prostaglandin G/H synthase and cyclooxygenase)	PTGS2	5743	NM_000963	0001516 // prostaglandin biosynthetic process // inferred from sequence or structural similarity /// 0001516 // prostaglandin biosynthetic process // non-traceable author statement /// 0001525 // angiogenesis // inferred from electronic annotation /// 0006629 // lipid metabolic process // inferred from electronic annotation /// 0006631 // fatty acid metabolic process // inferred from electronic annotation /// 0006633 // fatty acid biosynthetic process // inferred from electronic annotation /// 0006693 // prostaglandin metabolic process // traceable author statement /// 0006928 // cellular component movement // traceable author	0005634 // nucleus // inferred from sequence or structural similarity /// 0005737 // cytoplasm // inferred from direct assay /// 0005737 // cytoplasm // inferred from sequence or structural similarity /// 0005783 // endoplasmic reticulum // inferred from electronic annotation /// 0005789 // endoplasmic reticulum membrane // traceable author statement ///	0004601 // peroxidase activity // non-traceable author statement /// 0004666 // prostaglandin-endoperoxide synthase activity // inferred from direct assay /// 0005515 // protein binding // inferred from electronic annotation /// 0008289 // lipid binding // inferred from electronic annotation /// 0016491 // oxidoreductase activity // inferred from electronic annotation /// 0016702 // oxidoreductase activity, acting on single donors with incorporation of molecular oxygen, incorporation of two atoms of oxygen // inferred from electronic annotation /// 0019899 // enzyme binding // inferred from physical interaction /// 0020037 // heme binding // inferred from sequence or structural similarity ///

204798_at	NM_005375	MYB	Homo sapiens	6-Oct-14	Exemplar sequence	GenBank	gb:NM_005375.1 /DEF=Homo sapiens v-myb avian myeloblastosis viral oncogene homolog (MYB), mRNA. /FEA=mRNA /GEN=MYB /PROD=v-myb avian myeloblastosis viral oncogenehomolog /DB_XREF=gi:4885496 /UG=Hs.1334 v-myb avian myeloblastosis viral oncogene homolog /FL=gb:M15024.1 gb:AF104863.1 gb:NM_005375	NM_005375	v-myb avian myeloblastosis viral oncogene homolog	MYB	4602	NM_001130172 /// NM_001130173 /// NM_001161656 /// NM_001161657 /// NM_001161658 /// NM_001161659 /// NM_001161660 /// NM_005375 /// XM_006715495	0000082 // G1/S transition of mitotic cell cycle // inferred from electronic annotation /// 0000122 // negative regulation of transcription from RNA polymerase II promoter // inferred from mutant phenotype /// 0001701 // in utero embryonic development // inferred from electronic annotation /// 0006260 // DNA replication // inferred from electronic annotation /// 0006338 // chromatin remodeling // inferred from mutant phenotype /// 0006351 // transcription, DNA-templated // inferred from electronic annotation /// 0006355 // regulation of transcription, DNA-templated // non-traceable author [illegible]	0005634 // nucleus // inferred from electronic annotation /// 0016363 // nuclear matrix // non-traceable author statement	0000978 // RNA polymerase II core promoter proximal region sequence-specific DNA binding // inferred from direct assay /// 0001077 // RNA polymerase II core promoter proximal region sequence-specific DNA binding transcription factor activity involved in positive regulation of transcription // inferred by curator /// 0003677 // DNA binding // inferred from electronic annotation /// 0003682 // chromatin binding // inferred from electronic annotation /// 0003700 // sequence-specific DNA binding transcription factor activity // inferred from electronic annotation /// 0005515 // protein binding // inferred from physical interaction

204972_at	NM_016817	OAS2	Homo sapiens	6-Oct-14	Exemplar sequence	GenBank	gb:NM_016817.1 /DEF=Homo sapiens 2-5oligoadenylate synthetase 2 (OAS2), transcript variant 1, mRNA. /FEA=mRNA /GEN=OAS2 /PROD=2-5oligoadenylate synthetase 2, isoform p71 /DB_XREF=gi:8051624 /UG=Hs.264981 2-5oligoadenylate synthetase 2 /FL=gb:M87434.1 gb:NM_016817.1	NM_016817	2'-5'-oligoadenylate synthetase 2, 69/71kDa	OAS2	4939	NM_001032731 /// NM_002535 /// NM_016817	0002376 // immune system process // inferred from electronic annotation /// 0006139 // nucleobase-containing compound metabolic process // traceable author statement /// 0006164 // purine nucleotide biosynthetic process // inferred from direct assay /// 0006401 // RNA catabolic process // inferred from electronic annotation /// 0006486 // protein glycosylation // inferred from direct assay /// 0006955 // immune response // inferred from electronic annotation /// 0008152 // metabolic process // inferred from electronic annotation /// 0009615 // response to virus // traceable author statement /// 0018377 // protein myristoylation // [illegible]	0005634 // nucleus // inferred from electronic annotation /// 0005737 // cytoplasm // inferred from direct assay /// 0005739 // mitochondrion // inferred from electronic annotation /// 0005783 // endoplasmic reticulum // inferred from electronic annotation /// 0005829 // cytosol // traceable author statement /// 0016020 // membrane // traceable author statement /// 0043231 // [illegible]	0000166 // nucleotide binding // inferred from electronic annotation /// 0001730 // 2'-5'-oligoadenylate synthetase activity // inferred from direct assay /// 0001730 // 2'-5'-oligoadenylate synthetase activity // inferred from mutant phenotype /// 0003723 // RNA binding // inferred from electronic annotation /// 0003725 // double-stranded RNA binding // inferred from direct assay /// 0003725 // double-stranded RNA binding // inferred from mutant phenotype /// 0005524 // ATP binding // inferred from mutant phenotype /// 0008270 // zinc ion binding // inferred from direct assay /// 0016740 // transferase activity // inferred from electronic annotation /// 0016779 // [illegible]

205000_at	NM_004660	DDX3Y	Homo sapiens	6-Oct-14	Exemplar sequence	GenBank	gb:NM_004660.2 /DEF=Homo sapiens DEADH (Asp-Glu-Ala-AspHis) box polypeptide, Y chromosome (DBY), mRNA. /FEA=mRNA /GEN=DBY /PROD=DEADH (Asp-Glu-Ala-AspHis) box polypeptide, Ychromosome /DB_XREF=gi:13514808 /UG=Hs.99120 DEADH (Asp-Glu-Ala-AspHis) box polypeptide, Y chromosome /FL=gb:NM_004660.2 gb:AF000984.1 gb:AF000985.1	NM_004660	DEAD (Asp-Glu-Ala-Asp) box helicase 3, Y-linked	DDX3Y	8653	NM_001122665 /// NM_004660 /// XM_005262564 /// XM_006724877 /// XM_006724878	0006200 // ATP catabolic process // inferred from electronic annotation /// 0008152 // metabolic process // inferred from electronic annotation	0005634 // nucleus // inferred from electronic annotation /// 0005737 // cytoplasm // inferred from electronic annotation /// 0016020 // membrane // inferred from direct assay	0000166 // nucleotide binding // inferred from electronic annotation /// 0003676 // nucleic acid binding // inferred from electronic annotation /// 0003677 // DNA binding // inferred from electronic annotation /// 0003723 // RNA binding // inferred from electronic annotation /// 0004386 // helicase activity // inferred from electronic annotation /// 0005524 // ATP binding // inferred from electronic annotation /// 0008026 // ATP-dependent helicase activity // inferred from electronic annotation /// 0016787 // hydrolase activity // inferred from electronic annotation

205001_s_at	AF000985	DDX3Y	Homo sapiens	6-Oct-14	Exemplar sequence	GenBank	gb:AF000985.1 /DEF=Homo sapiens dead box, Y isoform (DBY) mRNA, alternative transcript 1, complete cds. /FEA=mRNA /GEN=DBY /PROD=dead box, Y isoform /DB_XREF=gi:2580555 /UG=Hs.99120 DEADH (Asp-Glu-Ala-AspHis) box polypeptide, Y chromosome /FL=gb:NM_004660.2 gb:AF000984.1 gb:AF000985.1	AF000985	DEAD (Asp-Glu-Ala-Asp) box helicase 3, Y-linked	DDX3Y	8653	NM_001122665 /// NM_004660 /// XM_005262564 /// XM_006724877 /// XM_006724878	0006200 // ATP catabolic process // inferred from electronic annotation /// 0008152 // metabolic process // inferred from electronic annotation	0005634 // nucleus // inferred from electronic annotation /// 0005737 // cytoplasm // inferred from electronic annotation /// 0016020 // membrane // inferred from direct assay	0000166 // nucleotide binding // inferred from electronic annotation /// 0003676 // nucleic acid binding // inferred from electronic annotation /// 0003677 // DNA binding // inferred from electronic annotation /// 0003723 // RNA binding // inferred from electronic annotation /// 0004386 // helicase activity // inferred from electronic annotation /// 0005524 // ATP binding // inferred from electronic annotation /// 0008026 // ATP-dependent helicase activity // inferred from electronic annotation /// 0016787 // hydrolase activity // inferred from electronic annotation

205012_s_at	NM_005326	HAGH	Homo sapiens	6-Oct-14	Exemplar sequence	GenBank	gb:NM_005326.1 /DEF=Homo sapiens hydroxyacyl glutathione hydrolase (HAGH), mRNA. /FEA=mRNA /GEN=HAGH /PROD=hydroxyacyl glutathione hydrolase /DB_XREF=gi:4885388 /UG=Hs.155482 hydroxyacyl glutathione hydrolase /FL=gb:BC000840.1 gb:BC002627.1 gb:NM_005326.1	NM_005326	hydroxyacylglutathione hydrolase	HAGH	3029	NM_001040427 /// NM_001286249 /// NM_005326	0006750 // glutathione biosynthetic process // inferred from direct assay /// 0008152 // metabolic process // inferred from electronic annotation	0005737 // cytoplasm // inferred from direct assay /// 0005739 // mitochondrion // inferred from electronic annotation /// 0005759 // mitochondrial matrix // inferred from direct assay /// 0005759 // mitochondrial matrix // inferred from electronic annotation /// 0070062 // extracellular vesicular exosome // inferred from direct assay	0004416 // hydroxyacylglutathione hydrolase activity // inferred from direct assay /// 0008270 // zinc ion binding // inferred from electronic annotation /// 0016787 // hydrolase activity // inferred from electronic annotation /// 0046872 // metal ion binding // inferred from electronic annotation

205040_at	NM_000607	ORM1	Homo sapiens	6-Oct-14	Exemplar sequence	GenBank	gb:NM_000607.1 /DEF=Homo sapiens orosomucoid 1 (ORM1), mRNA. /FEA=mRNA /GEN=ORM1 /PROD=orosomucoid 1 precursor /DB_XREF=gi:9257231 /UG=Hs.572 orosomucoid 1 /FL=gb:M13692.1 gb:NM_000607.1	NM_000607	orosomucoid 1	ORM1	5004	NM_000607	0002682 // regulation of immune system process // inferred from electronic annotation /// 0006810 // transport // inferred from electronic annotation /// 0006953 // acute-phase response // inferred from electronic annotation /// 0006954 // inflammatory response // traceable author statement	0005576 // extracellular region // non-traceable author statement /// 0005615 // extracellular space // inferred from direct assay /// 0070062 // extracellular vesicular exosome // inferred from direct assay /// 0072562 // blood microparticle // inferred from direct assay	0005515 // protein binding // inferred from physical interaction
205041_s_at	NM_000607	ORM1	Homo sapiens	6-Oct-14	Exemplar sequence	GenBank	gb:NM_000607.1 /DEF=Homo sapiens orosomucoid 1 (ORM1), mRNA. /FEA=mRNA /GEN=ORM1 /PROD=orosomucoid 1 precursor /DB_XREF=gi:9257231 /UG=Hs.572 orosomucoid 1 /FL=gb:M13692.1 gb:NM_000607.1	NM_000607	orosomucoid 1 /// orosomucoid 2	ORM1 /// ORM2	5004 /// 5005	NM_000607 /// NM_000608	0002682 // regulation of immune system process // inferred from electronic annotation /// 0006810 // transport // inferred from electronic annotation /// 0006953 // acute-phase response // inferred from electronic annotation /// 0006954 // inflammatory response // traceable author statement	0005576 // extracellular region // non-traceable author statement /// 0005615 // extracellular space // inferred from direct assay /// 0070062 // extracellular vesicular exosome // inferred from direct assay /// 0072562 // blood microparticle // inferred from direct assay	0005515 // protein binding // inferred from physical interaction

205048_s_at	NM_003832	PSPH	Homo sapiens	6-Oct-14	Exemplar sequence	GenBank	gb:NM_003832.1 /DEF=Homo sapiens phosphoserine phosphatase-like (PSPHL), mRNA. /FEA=mRNA /GEN=PSPHL /PROD=L-3-phosphoserine phosphatase homolog /DB_XREF=gi:4502934 /UG=Hs.76845 phosphoserine phosphatase-like /FL=gb:NM_003832.1	NM_003832	phosphoserine phosphatase	PSPH	5723	NM_004577 /// XM_005271773 /// XM_005271774 /// XM_005271775 /// XM_005271776 /// XM_005271777 /// XM_006715760 /// XM_006715761	0006563 // L-serine metabolic process // inferred from direct assay /// 0006564 // L-serine biosynthetic process // not recorded /// 0006564 // L-serine biosynthetic process // traceable author statement /// 0008152 // metabolic process // inferred from electronic annotation /// 0008652 // cellular amino acid biosynthetic process // traceable author statement /// 0009612 // response to mechanical stimulus // inferred from electronic annotation /// 0016311 // dephosphorylation // inferred from direct assay /// 0016311 // dephosphorylation // inferred from electronic annotation /// 0016311 // dephosphorylation // [illegible]	0005737 // cytoplasm // not recorded /// 0005829 // cytosol // traceable author statement /// 0043005 // neuron projection // inferred from electronic annotation	0000287 // magnesium ion binding // inferred from direct assay /// 0004647 // phosphoserine phosphatase activity // inferred from direct assay /// 0004647 // phosphoserine phosphatase activity // traceable author statement /// 0005509 // calcium ion binding // inferred from direct assay /// 0008253 // 5'-nucleotidase activity // inferred from electronic annotation /// 0016787 // hydrolase activity // inferred from electronic annotation /// 0016791 // phosphatase activity // inferred from electronic annotation /// 0042803 // protein homodimerization activity // inferred from physical interaction /// 0046872 // metal ion binding // inferred from electronic annotation

205117_at	X59065	FGF1	Homo sapiens	6-Oct-14	Consensus sequence	GenBank	Consensus includes gb:X59065 /DEF=H.sapiens FGF gene, exon 3 /FEA=mRNA /DB_XREF=gi:31359 /UG=Hs.75297 fibroblast growth factor 1 (acidic) /FL=gb:M13361.1 gb:NM_000800.1	X59065	fibroblast growth factor 1 (acidic)	FGF1	2246	NM_000800 /// NM_001144892 /// NM_001144934 /// NM_001144935 /// NM_001257205 /// NM_001257206 /// NM_001257207 /// NM_001257208 /// NM_001257209 /// NM_001257210 /// NM_001257211 /// NM_001257212 /// NM_033136 /// NM_033137 /// XM_005268390 ///	0001525 // angiogenesis // inferred from electronic annotation /// 0001759 // organ induction // inferred from electronic annotation /// 0001934 // positive regulation of protein phosphorylation // inferred from electronic annotation /// 0007165 // signal transduction // non-traceable author statement /// 0007173 // epidermal growth factor receptor signaling pathway // traceable author statement /// 0007275 // multicellular organismal development // traceable author statement /// 0008284 // positive regulation of cell proliferation // inferred from genetic interaction /// 0008286 // insulin receptor signaling pathway // traceable [illegible]	0005576 // extracellular region // inferred from direct assay /// 0005576 // extracellular region // traceable author statement /// 0005578 // proteinaceous extracellular matrix // inferred from electronic annotation /// 0005615 // extracellular space // inferred from direct assay /// 0005634 // nucleus // inferred from direct assay /// 0005730 // nucleolus // inferred from direct assay /// 0005737 // cytoplasm // [illegible]	0005102 // receptor binding // inferred from electronic annotation /// 0005104 // fibroblast growth factor receptor binding // inferred from physical interaction /// 0005515 // protein binding // inferred from physical interaction /// 0008083 // growth factor activity // inferred from direct assay /// 0008201 // heparin binding // inferred from direct assay /// 0030544 // Hsp70 protein binding // inferred from electronic annotation /// 0044548 // S100 protein binding // inferred from physical interaction

205157_s_at	NM_000422	JUP	Homo sapiens	6-Oct-14	Exemplar sequence	GenBank	gb:NM_000422.1 /DEF=Homo sapiens keratin 17 (KRT17), mRNA. /FEA=mRNA /GEN=KRT17 /PROD=keratin 17 /DB_XREF=gi:4557700 /UG=Hs.2785 keratin 17 /FL=gb:NM_000422.1	NM_000422	junction plakoglobin /// keratin 17	JUP /// KRT17	3728 /// 3872	NM_000422 /// NM_002230 /// NM_021991 /// XM_005257313 /// XM_005257349 /// XM_006721871 /// XM_006721872 /// XM_006721873 /// XM_006721874 /// XM_006721875 /// XM_006721876 /// XM_006721877 /// XM_006721878	0000122 // negative regulation of transcription from RNA polymerase II promoter // not recorded /// 0000902 // cell morphogenesis // not recorded /// 0002009 // morphogenesis of an epithelium // inferred from electronic annotation /// 0002159 // desmosome assembly // inferred from direct assay /// 0002159 // desmosome assembly // inferred from mutant phenotype /// 0003136 // negative regulation of heart induction by canonical Wnt signaling pathway // not recorded /// 0003181 // atrioventricular valve morphogenesis // not recorded /// 0003308 // negative regulation of Wnt signaling pathway	0005634 // nucleus // inferred from mutant phenotype /// 0005667 // transcription factor complex // not recorded /// 0005737 // cytoplasm // inferred from mutant phenotype /// 0005737 // cytoplasm // inferred from electronic annotation /// 0005829 // cytosol // inferred from sequence or structural similarity /// 0005856 // cytoskeleton // inferred from sequence or structural	0003713 // transcription coactivator activity // inferred from direct assay /// 0005198 // structural molecule activity // not recorded /// 0005198 // structural molecule activity // non-traceable author statement /// 0005199 // structural constituent of cell wall // inferred by curator /// 0005200 // structural constituent of cytoskeleton // traceable author statement /// 0005488 // binding // inferred from electronic annotation /// 0005515 // protein binding // inferred from physical interaction /// 0019901 // protein kinase binding // not recorded /// 0019903 // protein phosphatase binding // inferred from physical interaction /// 0032395 // MHC class II receptor activity // inferred from direct assay /// 0042289 // MHC class II

205187_at	AF010601	SMAD5	Homo sapiens	6-Oct-14	Exemplar sequence	GenBank	gb:AF010601.1 /DEF=Homo sapiens SMAD5 (Smad5) mRNA, complete cds. /FEA=mRNA /GEN=Smad5 /PROD=SMAD5 /DB_XREF=gi:2317785 /UG=Hs.37501 MAD (mothers against decapentaplegic, Drosophila) homolog 5 /FL=gb:U59913.1 gb:U73825.1 gb:AF009678.1 gb:AF010601.1 gb:NM_005903.1	AF010601	SMAD family member 5	SMAD5	4090	NM_001001419 /// NM_001001420 /// NM_005903	0001503 // ossification // inferred from electronic annotation /// 0001657 // ureteric bud development // inferred from electronic annotation /// 0001880 // Mullerian duct regression // inferred from electronic annotation /// 0002051 // osteoblast fate commitment // inferred from electronic annotation /// 0006351 // transcription, DNA-templated // inferred from electronic annotation /// 0006355 // regulation of transcription, DNA-templated // inferred from electronic annotation /// 0006468 // protein phosphorylation // inferred from electronic annotation /// 0007165 // signal transduction // non-traceable author [illegible]	0005622 // intracellular // non-traceable author statement /// 0005634 // nucleus // non-traceable author statement /// 0005634 // nucleus // traceable author statement /// 0005654 // nucleoplasm // traceable author statement /// 0005667 // transcription factor complex // inferred from electronic annotation /// 0005737 // cytoplasm // inferred from electronic annotation /// [illegible]	0000979 // RNA polymerase II core promoter sequence-specific DNA binding // inferred from electronic annotation /// 0003677 // DNA binding // inferred from electronic annotation /// 0003700 // sequence-specific DNA binding transcription factor activity // inferred from electronic annotation /// 0005057 // receptor signaling protein activity // non-traceable author statement /// 0005515 // protein binding // inferred from physical interaction /// 0030618 // transforming growth factor beta receptor, pathway-specific cytoplasmic mediator activity // traceable author statement /// 0031625 // ubiquitin protein ligase binding // inferred from physical interaction /// 0046872 // metal ion binding // inferred from electronic

205188_s_at	NM_005903	SMAD5	Homo sapiens	6-Oct-14	Exemplar sequence	GenBank	gb:NM_005903.1 /DEF=Homo sapiens MAD (mothers against decapentaplegic, Drosophila) homolog 5 (MADH5), mRNA. /FEA=mRNA /GEN=MADH5 /PROD=MAD (mothers against decapentaplegic,Drosophila) homolog 5 /DB_XREF=gi:5174514 /UG=Hs.37501 MAD (mothers against decapentaplegic, Drosophila) homolog 5 /FL=gb:U59913.	NM_005903	SMAD family member 5	SMAD5	4090	NM_001001419 /// NM_001001420 /// NM_005903	0001503 // ossification // inferred from electronic annotation /// 0001657 // ureteric bud development // inferred from electronic annotation /// 0001880 // Mullerian duct regression // inferred from electronic annotation /// 0002051 // osteoblast fate commitment // inferred from electronic annotation /// 0006351 // transcription, DNA-templated // inferred from electronic annotation /// 0006355 // regulation of transcription, DNA-templated // inferred from electronic annotation /// 0006468 // protein phosphorylation // inferred from electronic annotation /// 0007165 // signal transduction // non-traceable author	0005622 // intracellular // non-traceable author statement /// 0005634 // nucleus // non-traceable author statement /// 0005634 // nucleus // traceable author statement /// 0005654 // nucleoplasm // traceable author statement /// 0005667 // transcription factor complex // inferred from electronic annotation /// 0005737 // cytoplasm // inferred from electronic annotation ///	0000979 // RNA polymerase II core promoter sequence-specific DNA binding // inferred from electronic annotation /// 0003677 // DNA binding // inferred from electronic annotation /// 0003700 // sequence-specific DNA binding transcription factor activity // inferred from electronic annotation /// 0005057 // receptor signaling protein activity // non-traceable author statement /// 0005515 // protein binding // inferred from physical interaction /// 0030618 // transforming growth factor beta receptor, pathway-specific cytoplasmic mediator activity // traceable author statement /// 0031625 // ubiquitin protein ligase binding // inferred from physical interaction /// 0046872 // metal ion binding // inferred from electronic

205194_at	NM_004577	PSPH	Homo sapiens	6-Oct-14	Exemplar sequence	GenBank	gb:NM_004577.1 /DEF=Homo sapiens phosphoserine phosphatase (PSPH), mRNA. /FEA=mRNA /GEN=PSPH /PROD=phosphoserine phosphatase /DB_XREF=gi:4758971 /UG=Hs.56407 phosphoserine phosphatase /FL=gb:NM_004577.1	NM_004577	phosphoserine phosphatase	PSPH	5723	NM_004577 /// XM_005271773 /// XM_005271774 /// XM_005271775 /// XM_005271776 /// XM_005271777 /// XM_006715760 /// XM_006715761	0006563 // L-serine metabolic process // inferred from direct assay /// 0006564 // L-serine biosynthetic process // not recorded /// 0006564 // L-serine biosynthetic process // traceable author statement /// 0008152 // metabolic process // inferred from electronic annotation /// 0008652 // cellular amino acid biosynthetic process // traceable author statement /// 0009612 // response to mechanical stimulus // inferred from electronic annotation /// 0016311 // dephosphorylation // inferred from direct assay /// 0016311 // dephosphorylation // inferred from electronic annotation /// 0016311 // dephosphorylation // [illegible]	0005737 // cytoplasm // not recorded /// 0005829 // cytosol // traceable author statement /// 0043005 // neuron projection // inferred from electronic annotation	0000287 // magnesium ion binding // inferred from direct assay /// 0004647 // phosphoserine phosphatase activity // inferred from direct assay /// 0004647 // phosphoserine phosphatase activity // traceable author statement /// 0005509 // calcium ion binding // inferred from direct assay /// 0008253 // 5'-nucleotidase activity // inferred from electronic annotation /// 0016787 // hydrolase activity // inferred from electronic annotation /// 0016791 // phosphatase activity // inferred from electronic annotation /// 0042803 // protein homodimerization activity // inferred from physical interaction /// 0046872 // metal ion binding // inferred from electronic annotation

205214_at	NM_004226	STK17B	Homo sapiens	6-Oct-14	Exemplar sequence	GenBank	gb:NM_004226.1 /DEF=Homo sapiens serinethreonine kinase 17b (apoptosis-inducing) (STK17B), mRNA. /FEA=mRNA /GEN=STK17B /PROD=serinethreonine kinase 17b(apoptosis-inducing) /DB_XREF=gi:4758193 /UG=Hs.120996 serinethreonine kinase 17b (apoptosis-inducing) /FL=gb:AB011421.1 gb:NM_004226.1	NM_004226	serine/threonine kinase 17b	STK17B	9262	NM_004226	0006468 // protein phosphorylation // inferred from direct assay /// 0006915 // apoptotic process // inferred from electronic annotation /// 0012501 // programmed cell death // inferred from electronic annotation /// 0016310 // phosphorylation // inferred from electronic annotation /// 0035556 // intracellular signal transduction // inferred from direct assay /// 0046777 // protein autophosphorylation // inferred from sequence or structural similarity /// 2000271 // positive regulation of fibroblast apoptotic process // inferred from mutant phenotype	0005634 // nucleus // inferred from direct assay /// 0005793 // endoplasmic reticulum-Golgi intermediate compartment // inferred from electronic annotation /// 0005886 // plasma membrane // inferred from electronic annotation /// 0015629 // actin cytoskeleton // inferred from electronic annotation /// 0016020 // membrane // inferred from electronic annotation	0000166 // nucleotide binding // inferred from electronic annotation /// 0004672 // protein kinase activity // inferred from sequence or structural similarity /// 0004674 // protein serine/threonine kinase activity // inferred from direct assay /// 0004713 // protein tyrosine kinase activity // inferred from electronic annotation /// 0005515 // protein binding // inferred from electronic annotation /// 0005524 // ATP binding // inferred from direct assay /// 0016301 // kinase activity // inferred from electronic annotation /// 0016740 // transferase activity // inferred from electronic annotation /// 0016772 // transferase activity, transferring phosphorus-containing groups // inferred from [illegible]

205298_s_at	W58757	BTN2A2	Homo sapiens	6-Oct-14	Consensus sequence	GenBank	Consensus includes gb:W58757 /FEA=EST /DB_XREF=gi:1364334 /DB_XREF=est:zd24d07.s1 /CLONE=IMAGE:341581 /UG=Hs.91813 butyrophilin, subfamily 2, member A2 /FL=gb:U90550.1 gb:NM_006995.2	W58757	butyrophilin, subfamily 2, member A2	BTN2A2	10385	NM_001197237 /// NM_001197238 /// NM_001197239 /// NM_001197240 /// NM_006995 /// NM_181531 /// XM_005248797 /// XM_005248798 /// XM_006714953 /// XM_006714954 /// XM_006714955 /// XM_006714956	0031324 // negative regulation of cellular metabolic process // inferred from sequence or structural similarity /// 0046007 // negative regulation of activated T cell proliferation // inferred from sequence or structural similarity /// 0050710 // negative regulation of cytokine secretion // inferred from sequence or structural similarity	0016020 // membrane // inferred from electronic annotation /// 0016021 // integral component of membrane // inferred from electronic annotation /// 0070062 // extracellular vesicular exosome // inferred from direct assay	0005515 // protein binding // inferred from electronic annotation
205299_s_at	NM_006995	BTN2A2	Homo sapiens	6-Oct-14	Exemplar sequence	GenBank	gb:NM_006995.2 /DEF=Homo sapiens butyrophilin, subfamily 2, member A2 (BTN2A2), mRNA. /FEA=mRNA /GEN=BTN2A2 /PROD=butyrophilin, subfamily 2, member A2 /DB_XREF=gi:6453812 /UG=Hs.91813 butyrophilin, subfamily 2, member A2 /FL=gb:U90550.1 gb:NM_006995.2	NM_006995	butyrophilin, subfamily 2, member A2	BTN2A2	10385	NM_001197237 /// NM_001197238 /// NM_001197239 /// NM_001197240 /// NM_006995 /// NM_181531 /// XM_005248797 /// XM_005248798 /// XM_006714953 /// XM_006714954 /// XM_006714955 /// XM_006714956	0031324 // negative regulation of cellular metabolic process // inferred from sequence or structural similarity /// 0046007 // negative regulation of activated T cell proliferation // inferred from sequence or structural similarity /// 0050710 // negative regulation of cytokine secretion // inferred from sequence or structural similarity	0016020 // membrane // inferred from electronic annotation /// 0016021 // integral component of membrane // inferred from electronic annotation /// 0070062 // extracellular vesicular exosome // inferred from direct assay	0005515 // protein binding // inferred from electronic annotation

205434_s_at	AW451954	AAK1	Homo sapiens	6-Oct-14	Consensus sequence	GenBank	Consensus includes gb:AW451954 /FEA=EST /DB_XREF=gi:6992730 /DB_XREF=est:UI-H-BI3-alt-h-06-0-UI.s1 /CLONE=IMAGE:3068771 /UG=Hs.135941 KIAA1048 protein /FL=gb:AB028971.1 gb:NM_014911.1	AW451954	AP2 associated kinase 1	AAK1	22848	NM_014911	0006468 // protein phosphorylation // inferred from direct assay /// 0006897 // endocytosis // inferred from electronic annotation /// 0016310 // phosphorylation // inferred from electronic annotation /// 0032880 // regulation of protein localization // inferred from direct assay /// 0045747 // positive regulation of Notch signaling pathway // inferred from direct assay /// 0046777 // protein autophosphorylation // inferred from sequence or structural similarity /// 0050821 // protein stabilization // inferred from direct assay /// 2000369 // regulation of clathrin-mediated endocytosis // inferred [illegible]	0005886 // plasma membrane // inferred from electronic annotation /// 0005905 // coated pit // inferred from electronic annotation /// 0016020 // membrane // inferred from electronic annotation /// 0019897 // extrinsic component of plasma membrane // inferred from sequence or structural similarity /// 0030136 // clathrin-coated vesicle // inferred from sequence or structural [illegible]	0000166 // nucleotide binding // inferred from electronic annotation /// 0004672 // protein kinase activity // inferred from electronic annotation /// 0004674 // protein serine/threonine kinase activity // inferred from sequence or structural similarity /// 0004713 // protein tyrosine kinase activity // inferred from electronic annotation /// 0005112 // Notch binding // inferred from direct assay /// 0005515 // protein binding // inferred from physical interaction /// 0005524 // ATP binding // inferred from electronic annotation /// 0016301 // kinase activity // inferred from electronic annotation /// 0016740 // transferase activity // inferred from electronic annotation /// 0016772 // transferase [illegible]

205435_s_at	NM_014911	AAK1	Homo sapiens	6-Oct-14	Exemplar sequence	GenBank	gb:NM_014911.1 /DEF=Homo sapiens KIAA1048 protein (KIAA1048), mRNA. /FEA=mRNA /GEN=KIAA1048 /PROD=KIAA1048 protein /DB_XREF=gi:7662461 /UG=Hs.135941 KIAA1048 protein /FL=gb:AB028971.1 gb:NM_014911.1	NM_014911	AP2 associated kinase 1	AAK1	22848	NM_014911	0006468 // protein phosphorylation // inferred from direct assay /// 0006897 // endocytosis // inferred from electronic annotation /// 0016310 // phosphorylation // inferred from electronic annotation /// 0032880 // regulation of protein localization // inferred from direct assay /// 0045747 // positive regulation of Notch signaling pathway // inferred from direct assay /// 0046777 // protein autophosphorylation // inferred from sequence or structural similarity /// 0050821 // protein stabilization // inferred from direct assay /// 2000369 // regulation of clathrin-mediated endocytosis // inferred [illegible]	0005886 // plasma membrane // inferred from electronic annotation /// 0005905 // coated pit // inferred from electronic annotation /// 0016020 // membrane // inferred from electronic annotation /// 0019897 // extrinsic component of plasma membrane // inferred from sequence or structural similarity /// 0030136 // clathrin-coated vesicle // inferred from sequence or structural [illegible]	0000166 // nucleotide binding // inferred from electronic annotation /// 0004672 // protein kinase activity // inferred from electronic annotation /// 0004674 // protein serine/threonine kinase activity // inferred from sequence or structural similarity /// 0004713 // protein tyrosine kinase activity // inferred from electronic annotation /// 0005112 // Notch binding // inferred from direct assay /// 0005515 // protein binding // inferred from physical interaction /// 0005524 // ATP binding // inferred from electronic annotation /// 0016301 // kinase activity // inferred from electronic annotation /// 0016740 // transferase activity // inferred from electronic annotation /// 0016772 // transferase [illegible]

205465_x_at	BF000296	HS3ST1	Homo sapiens	6-Oct-14	Consensus sequence	GenBank	Consensus includes gb:BF000296 /FEA=EST /DB_XREF=gi:10700571 /DB_XREF=est:7h25e12.x1 /CLONE=IMAGE:3317038 /UG=Hs.40968 heparan sulfate (glucosamine) 3-O-sulfotransferase 1 /FL=gb:AF019386.1 gb:NM_005114.1	BF000296	heparan sulfate (glucosamine) 3-O-sulfotransferase 1	HS3ST1	9957	NM_005114 /// XM_005248221	0005975 // carbohydrate metabolic process // traceable author statement /// 0006024 // glycosaminoglycan biosynthetic process // traceable author statement /// 0030203 // glycosaminoglycan metabolic process // traceable author statement /// 0044281 // small molecule metabolic process // traceable author statement	0005737 // cytoplasm // inferred from direct assay /// 0005794 // Golgi apparatus // inferred from electronic annotation /// 0005796 // Golgi lumen // traceable author statement /// 0016021 // integral component of membrane // traceable author statement	0008146 // sulfotransferase activity // traceable author statement /// 0008467 // [heparan sulfate]-glucosamine 3-sulfotransferase 1 activity // inferred from electronic annotation /// 0016740 // transferase activity // inferred from electronic annotation

205466_s_at	NM_005114	HS3ST1	Homo sapiens	6-Oct-14	Exemplar sequence	GenBank	gb:NM_005114.1 /DEF=Homo sapiens heparan sulfate (glucosamine) 3-O-sulfotransferase 1 (HS3ST1), mRNA. /FEA=mRNA /GEN=HS3ST1 /PROD=heparan sulfate D-glucosaminyl3-O-sulfotransferase 1 precursor /DB_XREF=gi:4826763 /UG=Hs.40968 heparan sulfate (glucosamine) 3-O-sulfotransferase 1 /FL=gb:AF019386.1	NM_005114	heparan sulfate (glucosamine) 3-O-sulfotransferase 1	HS3ST1	9957	NM_005114 /// XM_005248221	0005975 // carbohydrate metabolic process // traceable author statement /// 0006024 // glycosaminoglycan biosynthetic process // traceable author statement /// 0030203 // glycosaminoglycan metabolic process // traceable author statement /// 0044281 // small molecule metabolic process // traceable author statement	0005737 // cytoplasm // inferred from direct assay /// 0005794 // Golgi apparatus // inferred from electronic annotation /// 0005796 // Golgi lumen // traceable author statement /// 0016021 // integral component of membrane // traceable author statement	0008146 // sulfotransferase activity // traceable author statement /// 0008467 // [heparan sulfate]-glucosamine 3-sulfotransferase 1 activity // inferred from electronic annotation /// 0016740 // transferase activity // inferred from electronic annotation

205937_at	NM_006569	CGREF1	Homo sapiens	6-Oct-14	Exemplar sequence	GenBank	gb:NM_006569.1 /DEF=Homo sapiens cell growth regulatory with EF-hand domain (CGR11), mRNA. /FEA=mRNA /GEN=CGR11 /PROD=cell growth regulatory with EF-hand domain /DB_XREF=gi:5729762 /UG=Hs.159525 cell growth regulatory with EF-hand domain /FL=gb:U66468.1 gb:NM_006569.1	NM_006569	cell growth regulator with EF-hand domain 1	CGREF1	10669	NM_001166239 /// NM_001166240 /// NM_001166241 /// NM_006569	0006950 // response to stress // traceable author statement /// 0007049 // cell cycle // inferred from electronic annotation /// 0007050 // cell cycle arrest // inferred from electronic annotation /// 0007155 // cell adhesion // inferred from electronic annotation /// 0008285 // negative regulation of cell proliferation // traceable author statement /// 0030308 // negative regulation of cell growth // inferred from electronic annotation	0005576 // extracellular region // inferred from electronic annotation	0005509 // calcium ion binding // inferred from electronic annotation /// 0046872 // metal ion binding // inferred from electronic annotation

206189_at	NM_003728	UNC5C	Homo sapiens	6-Oct-14	Exemplar sequence	GenBank	gb:NM_003728.1 /DEF=Homo sapiens unc5 (C.elegans homolog) c (UNC5C), mRNA. /FEA=mRNA /GEN=UNC5C /PROD=unc5 (C.elegans homolog) c /DB_XREF=gi:4507836 /UG=Hs.44553 unc5 (C.elegans homolog) c /FL=gb:AF055634.1 gb:NM_003728.1	NM_003728	unc-5 homolog C (C. elegans)	UNC5C	8633	NM_003728 /// XM_005263321	0006915 // apoptotic process // inferred from electronic annotation /// 0007165 // signal transduction // inferred from electronic annotation /// 0007275 // multicellular organismal development // inferred from electronic annotation /// 0007411 // axon guidance // traceable author statement /// 0007420 // brain development // inferred from electronic annotation /// 0030334 // regulation of cell migration // inferred from electronic annotation /// 0033564 // anterior/posterior axon guidance // inferred from electronic annotation /// 0038007 // netrin-activated signaling pathway // inferred from [illegible]	0005886 // plasma membrane // inferred from electronic annotation /// 0016020 // membrane // inferred from electronic annotation /// 0016021 // integral component of membrane // inferred from electronic annotation	0005042 // netrin receptor activity // inferred from electronic annotation /// 0005515 // protein binding // inferred from electronic annotation

206461_x_at	NM_005951	MT1H	Homo sapiens	6-Oct-14	Exemplar sequence	GenBank	gb:NM_005951.1 /DEF=Homo sapiens metallothionein 1H (MT1H), mRNA. /FEA=mRNA /GEN=MT1H /PROD=metallothionein 1H /DB_XREF=gi:10835084 /UG=Hs.2667 metallothionein 1H /FL=gb:NM_005951.1	NM_005951	metallothionein 1H	MT1H	4496	NM_005951	0045926 // negative regulation of growth // inferred from sequence or structural similarity /// 0071276 // cellular response to cadmium ion // inferred from expression pattern /// 0071294 // cellular response to zinc ion // inferred from expression pattern	0005634 // nucleus // inferred from sequence or structural similarity /// 0005737 // cytoplasm // inferred from sequence or structural similarity /// 0048471 // perinuclear region of cytoplasm // inferred from sequence or structural similarity	0005515 // protein binding // inferred from physical interaction /// 0008270 // zinc ion binding // inferred from sequence or structural similarity /// 0046872 // metal ion binding // inferred from electronic annotation

206553_at	NM_002535	OAS2	Homo sapiens	6-Oct-14	Exemplar sequence	GenBank	gb:NM_002535.1 /DEF=Homo sapiens 2-5oligoadenylate synthetase 2 (OAS2), transcript variant 2, mRNA. /FEA=mRNA /GEN=OAS2 /PROD=2-5oligoadenylate synthetase 2, isoform p69 /DB_XREF=gi:4505484 /UG=Hs.264981 2-5oligoadenylate synthetase 2 /FL=gb:M87284.1 gb:NM_002535.1	NM_002535	2'-5'-oligoadenylate synthetase 2, 69/71kDa	OAS2	4939	NM_001032731 /// NM_002535 /// NM_016817	0002376 // immune system process // inferred from electronic annotation /// 0006139 // nucleobase-containing compound metabolic process // traceable author statement /// 0006164 // purine nucleotide biosynthetic process // inferred from direct assay /// 0006401 // RNA catabolic process // inferred from electronic annotation /// 0006486 // protein glycosylation // inferred from direct assay /// 0006955 // immune response // inferred from electronic annotation /// 0008152 // metabolic process // inferred from electronic annotation /// 0009615 // response to virus // traceable author statement /// 0018377 // protein myristoylation // [illegible]	0005634 // nucleus // inferred from electronic annotation /// 0005737 // cytoplasm // inferred from direct assay /// 0005739 // mitochondrion // inferred from electronic annotation /// 0005783 // endoplasmic reticulum // inferred from electronic annotation /// 0005829 // cytosol // traceable author statement /// 0016020 // membrane // traceable author statement /// 0043231 // [illegible]	0000166 // nucleotide binding // inferred from electronic annotation /// 0001730 // 2'-5'-oligoadenylate synthetase activity // inferred from direct assay /// 0001730 // 2'-5'-oligoadenylate synthetase activity // inferred from mutant phenotype /// 0003723 // RNA binding // inferred from electronic annotation /// 0003725 // double-stranded RNA binding // inferred from direct assay /// 0003725 // double-stranded RNA binding // inferred from mutant phenotype /// 0005524 // ATP binding // inferred from mutant phenotype /// 0008270 // zinc ion binding // inferred from direct assay /// 0016740 // transferase activity // inferred from electronic annotation /// 0016779 // [illegible]

206584_at	NM_015364	LY96	Homo sapiens	6-Oct-14	Exemplar sequence	GenBank	gb:NM_015364.1 /DEF=Homo sapiens MD-2 protein (MD-2), mRNA. /FEA=mRNA /GEN=MD-2 /PROD=MD-2 protein /DB_XREF=gi:7662503 /UG=Hs.69328 MD-2 protein /FL=gb:AB018549.1 gb:NM_015364.1 gb:AF168121.1	NM_015364	lymphocyte antigen 96	LY96	23643	NM_001195797 /// NM_015364	0002224 // toll-like receptor signaling pathway // traceable author statement /// 0002376 // immune system process // inferred from electronic annotation /// 0002755 // MyD88-dependent toll-like receptor signaling pathway // traceable author statement /// 0002756 // MyD88-independent toll-like receptor signaling pathway // traceable author statement /// 0006954 // inflammatory response // inferred from electronic annotation /// 0006968 // cellular defense response // traceable author statement /// 0007166 // cell surface receptor signaling pathway // traceable author [illegible]	0005576 // extracellular region // inferred from electronic annotation /// 0005615 // extracellular space // inferred from electronic annotation /// 0005886 // plasma membrane // traceable author statement /// 0010008 // endosome membrane // traceable author statement /// 0046696 // lipopolysaccharide receptor complex // inferred from direct assay	0001875 // lipopolysaccharide receptor activity // inferred from direct assay /// 0005515 // protein binding // inferred from physical interaction /// 0015026 // coreceptor activity // traceable author statement

206698_at	NM_021083	XK	Homo sapiens	6-Oct-14	Exemplar sequence	GenBank	gb:NM_021083.1 /DEF=Homo sapiens Kell blood group precursor (McLeod phenotype) (XK), mRNA. /FEA=mRNA /GEN=XK /PROD=Kell blood group precursor (McLeod phenotype) /DB_XREF=gi:10835266 /UG=Hs.78919 Kell blood group precursor (McLeod phenotype) /FL=gb:NM_021083.1	NM_021083	X-linked Kx blood group	XK	7504	NM_021083	0006810 // transport // traceable author statement /// 0006865 // amino acid transport // inferred from electronic annotation	0016020 // membrane // inferred from electronic annotation /// 0016021 // integral component of membrane // traceable author statement	0005215 // transporter activity // traceable author statement /// 0005515 // protein binding // inferred from physical interaction

206871_at	NM_001972	ELANE	Homo sapiens	6-Oct-14	Exemplar sequence	GenBank	gb:NM_001972.1 /DEF=Homo sapiens elastase 2, neutrophil (ELA2), mRNA. /FEA=mRNA /GEN=ELA2 /PROD=elastase 2, neutrophil /DB_XREF=gi:4503548 /UG=Hs.99863 elastase 2, neutrophil /FL=gb:M34379.1 gb:NM_001972.1	NM_001972	elastase, neutrophil expressed	ELANE	1991	NM_001972 /// XM_005259517 /// XM_006725397	0000122 // negative regulation of transcription from RNA polymerase II promoter // inferred from mutant phenotype /// 0001878 // response to yeast // inferred from electronic annotation /// 0002438 // acute inflammatory response to antigenic stimulus // inferred from electronic annotation /// 0006508 // proteolysis // inferred from direct assay /// 0006874 // cellular calcium ion homeostasis // non-traceable author statement /// 0006909 // phagocytosis // inferred from electronic annotation /// 0009411 // response to UV // inferred from direct assay /// 0022617 // extracellular matrix disassembly // traceable author [illegible]	0005576 // extracellular region // non-traceable author statement /// 0005576 // extracellular region // traceable author statement /// 0005737 // cytoplasm // inferred from direct assay /// 0009986 // cell surface // inferred from direct assay /// 0017053 // transcriptional repressor complex // inferred from direct assay /// 0030141 // secretory granule // inferred from direct assay /// 0070062 // extracellular [illegible]	0001106 // RNA polymerase II transcription corepressor activity // inferred from mutant phenotype /// 0002020 // protease binding // inferred from physical interaction /// 0003824 // catalytic activity // inferred from electronic annotation /// 0004175 // endopeptidase activity // inferred from direct assay /// 0004252 // serine-type endopeptidase activity // inferred from electronic annotation /// 0005515 // protein binding // inferred from physical interaction /// 0008201 // heparin binding // inferred from direct assay /// 0008233 // peptidase activity // inferred from direct assay /// 0008236 // serine-type peptidase activity // inferred from electronic annotation /// 0016787 // hydrolase activity // inferred from electronic annotation /// [illegible]

206931_at	NM_003441	ZNF141	Homo sapiens	6-Oct-14	Exemplar sequence	GenBank	gb:NM_003441.1 /DEF=Homo sapiens zinc finger protein 141 (clone pHZ-44) (ZNF141), mRNA. /FEA=mRNA /GEN=ZNF141 /PROD=zinc finger protein 141 (clone pHZ-44) /DB_XREF=gi:4507992 /UG=Hs.193677 zinc finger protein 141 (clone pHZ-44) /FL=gb:L15309.1 gb:NM_003441.1	NM_003441	zinc finger protein 141	ZNF141	7700	NM_003441 /// XM_005278365	0006351 // transcription, DNA-templated // inferred from electronic annotation /// 0006355 // regulation of transcription, DNA-templated // inferred from electronic annotation /// 0006366 // transcription from RNA polymerase II promoter // traceable author statement /// 0007275 // multicellular organismal development // inferred from electronic annotation /// 0009653 // anatomical structure morphogenesis // traceable author statement /// 0035108 // limb morphogenesis // inferred from mutant phenotype	0005622 // intracellular // inferred from electronic annotation /// 0005634 // nucleus // inferred from electronic annotation	0003676 // nucleic acid binding // inferred from electronic annotation /// 0003677 // DNA binding // inferred from electronic annotation /// 0046872 // metal ion binding // inferred from electronic annotation

206978_at	NM_000647	CCR2	Homo sapiens	6-Oct-14	Exemplar sequence	GenBank	gb:NM_000647.2 /DEF=Homo sapiens chemokine (C-C motif) receptor 2 (CCR2), mRNA. /FEA=mRNA /GEN=CCR2 /PROD=chemokine (C-C motif) receptor 2 /DB_XREF=gi:4827072 /UG=Hs.395 chemokine (C-C motif) receptor 2 /FL=gb:U03882.1 gb:NM_000647.2	NM_000647	chemokine (C-C motif) receptor 2	CCR2	729230	NM_001123041 /// NM_001123396	0001974 // blood vessel remodeling // inferred from sequence or structural similarity /// 0002407 // dendritic cell chemotaxis // traceable author statement /// 0002827 // positive regulation of T-helper 1 type immune response // inferred from sequence or structural similarity /// 0002829 // negative regulation of type 2 immune response // inferred from sequence or structural similarity /// 0006874 // cellular calcium ion homeostasis // inferred from sequence or structural similarity /// 0006935 // chemotaxis // traceable author statement /// 0006954 // inflammatory response // inferred from electronic annotation /// 0006955 // [illegible]	0005737 // cytoplasm // inferred from direct assay /// 0005829 // cytosol // inferred from sequence or structural similarity /// 0005886 // plasma membrane // inferred from direct assay /// 0005886 // plasma membrane // traceable author statement /// 0005887 // integral component of plasma membrane // traceable author statement /// 0016020 // membrane // inferred from electronic [illegible]	0004871 // signal transducer activity // inferred from electronic annotation /// 0004930 // G-protein coupled receptor activity // inferred from electronic annotation /// 0004950 // chemokine receptor activity // traceable author statement /// 0016493 // C-C chemokine receptor activity // inferred from electronic annotation /// 0031727 // CCR2 chemokine receptor binding // inferred from direct assay /// 0042803 // protein homodimerization activity // inferred from sequence or structural similarity

207090_x_at	NM_014898	ZFP30	Homo sapiens	6-Oct-14	Exemplar sequence	GenBank	gb:NM_014898.1 /DEF=Homo sapiens KIAA0961 protein (KIAA0961), mRNA. /FEA=mRNA /GEN=KIAA0961 /PROD=KIAA0961 protein /DB_XREF=gi:7662407 /UG=Hs.189464 KIAA0961 protein /FL=gb:AB023178.1 gb:NM_014898.1	NM_014898	ZFP30 zinc finger protein	ZFP30	22835	NM_014898 /// XM_005258665 /// XM_006723087 /// XM_006723088 /// XM_006723089	0006351 // transcription, DNA-templated // inferred from electronic annotation /// 0006355 // regulation of transcription, DNA-templated // not recorded	0005622 // intracellular // inferred from electronic annotation /// 0005634 // nucleus // inferred from electronic annotation	0003676 // nucleic acid binding // inferred from electronic annotation /// 0003677 // DNA binding // inferred from electronic annotation /// 0003700 // sequence-specific DNA binding transcription factor activity // not recorded /// 0046872 // metal ion binding // inferred from electronic annotation

207122_x_at	NM_001054	SULT1A2	Homo sapiens	6-Oct-14	Exemplar sequence	GenBank	gb:NM_001054.1 /DEF=Homo sapiens sulfotransferase family, cytosolic, 1A, phenol-preferring, member 2 (SULT1A2), mRNA. /FEA=mRNA /GEN=SULT1A2 /PROD=sulfotransferase family, cytosolic, 1A,phenol-preferring, member 2 /DB_XREF=gi:4507302 /UG=Hs.272462 sulfotransferase family, cytosolic, 1A, phenol-preferring, [illegible]	NM_001054	sulfotransferase family, cytosolic, 1A, phenol-preferring, member 2	SULT1A2	6799	NM_001054 /// NM_177528 /// XM_006721076 /// XM_006721077	0006584 // catecholamine metabolic process // inferred from electronic annotation /// 0006629 // lipid metabolic process // inferred from electronic annotation /// 0006805 // xenobiotic metabolic process // inferred from direct assay /// 0006805 // xenobiotic metabolic process // traceable author statement /// 0008152 // metabolic process // inferred from electronic annotation /// 0008202 // steroid metabolic process // inferred from electronic annotation /// 0009309 // amine biosynthetic process // traceable author statement /// 0018958 // phenol-containing compound metabolic process // inferred from direct assay [illegible]	0005737 // cytoplasm // inferred from electronic annotation /// 0005829 // cytosol // traceable author statement	0004062 // aryl sulfotransferase activity // inferred from direct assay /// 0008146 // sulfotransferase activity // traceable author statement /// 0016740 // transferase activity // inferred from electronic annotation /// 0047894 // flavonol 3-sulfotransferase activity // inferred from direct assay

207198_s_at	NM_004987	LIMS1	Homo sapiens	6-Oct-14	Exemplar sequence	GenBank	gb:NM_004987.2 /DEF=Homo sapiens LIM and senescent cell antigen-like domains 1 (LIMS1), mRNA. /FEA=mRNA /GEN=LIMS1 /PROD=LIM and senescent cell antigen-like domains 1 /DB_XREF=gi:13518025 /UG=Hs.112378 LIM and senescent cell antigen-like domains 1 /FL=gb:U09284.2 gb:NM_004987.2	NM_004987	LIM and senescent cell antigen-like domains 1 /// LIM and senescent cell antigen-like domains 3-like	LIMS1 /// LIMS3L	3987 /// 100288695	NM_001193482 /// NM_001193483 /// NM_001193484 /// NM_001193485 /// NM_001193488 /// NM_001205288 /// NM_004987 /// NR_038099 /// XM_005263563 /// XM_005263946 /// XM_005263947 /// XM_005263948 /// XM_005263949 /// XM_005263950 /// XM_005263951 ///	0007569 // cell aging // traceable author statement /// 0034329 // cell junction assembly // traceable author statement /// 0045892 // negative regulation of transcription, DNA-templated // inferred from direct assay /// 0071560 // cellular response to transforming growth factor beta stimulus // inferred from expression pattern	0005737 // cytoplasm // inferred from electronic annotation /// 0005829 // cytosol // traceable author statement /// 0005886 // plasma membrane // inferred from electronic annotation /// 0005925 // focal adhesion // inferred from direct assay /// 0016020 // membrane // inferred from electronic annotation /// 0030054 // cell junction // inferred from electronic annotation /// 0048471 // [illegible]	0005515 // protein binding // inferred from physical interaction /// 0008270 // zinc ion binding // inferred from direct assay /// 0008270 // zinc ion binding // inferred from electronic annotation /// 0046872 // metal ion binding // inferred from electronic annotation

207392_x_at	NM_001076	UGT2B15	Homo sapiens	6-Oct-14	Exemplar sequence	GenBank	gb:NM_001076.1 /DEF=Homo sapiens UDP glycosyltransferase 2 family, polypeptide B15 (UGT2B15), mRNA. /FEA=mRNA /GEN=UGT2B15 /PROD=UDP glycosyltransferase 2 family, polypeptideB15 /DB_XREF=gi:4507818 /UG=Hs.150207 UDP glycosyltransferase 2 family, polypeptide B15 /FL=gb:NM_001076.1 gb:U08854.1 gb:AF180322.1	NM_001076	UDP glucuronosyltransferase 2 family, polypeptide B15	UGT2B15	7366	NM_001076	0006805 // xenobiotic metabolic process // traceable author statement /// 0008152 // metabolic process // inferred from electronic annotation /// 0008202 // steroid metabolic process // traceable author statement /// 0052695 // cellular glucuronidation // inferred from direct assay	0005783 // endoplasmic reticulum // inferred from electronic annotation /// 0005789 // endoplasmic reticulum membrane // inferred from electronic annotation /// 0016020 // membrane // inferred from electronic annotation /// 0016021 // integral component of membrane // inferred from electronic annotation /// 0031090 // organelle [illegible]	0001972 // retinoic acid binding // inferred from direct assay /// 0015020 // glucuronosyltransferase activity // inferred from direct assay /// 0016740 // transferase activity // inferred from electronic annotation /// 0016757 // transferase activity, transferring glycosyl groups // inferred from electronic annotation /// 0016758 // transferase activity, transferring hexosyl groups // inferred from electronic annotation

207641_at	NM_012452	TNFRSF13B	Homo sapiens	6-Oct-14	Exemplar sequence	GenBank	gb:NM_012452.1 /DEF=Homo sapiens transmembrane activator and CAML interactor (TACI), mRNA. /FEA=mRNA /GEN=TACI /PROD=transmembrane activator and CAML interactor /DB_XREF=gi:6912693 /UG=Hs.158341 transmembrane activator and CAML interactor /FL=gb:AF023614.1 gb:NM_012452.1	NM_012452	tumor necrosis factor receptor superfamily, member 13B	TNFRSF13B	23495	NM_012452	0001782 // B cell homeostasis // inferred from electronic annotation /// 0002376 // immune system process // inferred from electronic annotation /// 0007166 // cell surface receptor signaling pathway // traceable author statement /// 0030889 // negative regulation of B cell proliferation // inferred from electronic annotation	0005887 // integral component of plasma membrane // inferred from electronic annotation /// 0009897 // external side of plasma membrane // inferred from electronic annotation /// 0016020 // membrane // inferred from electronic annotation /// 0016021 // integral component of membrane // inferred from electronic annotation	0004872 // receptor activity // traceable author statement /// 0005515 // protein binding // inferred from physical interaction

207674_at	NM_002000	FCAR	Homo sapiens	6-Oct-14	Exemplar sequence	GenBank	gb:NM_002000.1 /DEF=Homo sapiens Fc fragment of IgA, receptor for (FCAR), mRNA. /FEA=mRNA /GEN=FCAR /PROD=Fc fragment of IgA, receptor for /DB_XREF=gi:4503672 /UG=Hs.193122 Fc fragment of IgA, receptor for /FL=gb:D87855.1 gb:D87861.1 gb:NM_002000.1	NM_002000	Fc fragment of IgA, receptor for	FCAR	2204	NM_002000 /// NM_133269 /// NM_133271 /// NM_133272 /// NM_133273 /// NM_133274 /// NM_133277 /// NM_133278 /// NM_133279 /// NM_133280	0006955 // immune response // traceable author statement	0005576 // extracellular region // inferred from electronic annotation /// 0005886 // plasma membrane // inferred from direct assay /// 0005887 // integral component of plasma membrane // traceable author statement /// 0016020 // membrane // inferred from electronic annotation /// 0016021 // integral component of membrane // inferred from electronic annotation	0019862 // IgA binding // inferred from electronic annotation

207794_at	NM_000648	CCR2	Homo sapiens	6-Oct-14	Exemplar sequence	GenBank	gb:NM_000648.1 /DEF=Homo sapiens chemokine (C-C motif) receptor 2 (CCR2), mRNA. /FEA=mRNA /GEN=CCR2 /PROD=chemokine (C-C motif) receptor 2 /DB_XREF=gi:4757937 /UG=Hs.395 chemokine (C-C motif) receptor 2 /FL=gb:U03905.1 gb:NM_000648.1 gb:D29984.1	NM_000648	chemokine (C-C motif) receptor 2	CCR2	729230	NM_001123041 /// NM_001123396	0001974 // blood vessel remodeling // inferred from sequence or structural similarity /// 0002407 // dendritic cell chemotaxis // traceable author statement /// 0002827 // positive regulation of T-helper 1 type immune response // inferred from sequence or structural similarity /// 0002829 // negative regulation of type 2 immune response // inferred from sequence or structural similarity /// 0006874 // cellular calcium ion homeostasis // inferred from sequence or structural similarity /// 0006935 // chemotaxis // traceable author statement /// 0006954 // inflammatory response // inferred from electronic annotation /// 0006955 // [illegible]	0005737 // cytoplasm // inferred from direct assay /// 0005829 // cytosol // inferred from sequence or structural similarity /// 0005886 // plasma membrane // inferred from direct assay /// 0005886 // plasma membrane // traceable author statement /// 0005887 // integral component of plasma membrane // traceable author statement /// 0016020 // membrane // inferred from electronic [illegible]	0004871 // signal transducer activity // inferred from electronic annotation /// 0004930 // G-protein coupled receptor activity // inferred from electronic annotation /// 0004950 // chemokine receptor activity // traceable author statement /// 0016493 // C-C chemokine receptor activity // inferred from electronic annotation /// 0031727 // CCR2 chemokine receptor binding // inferred from direct assay /// 0042803 // protein homodimerization activity // inferred from sequence or structural similarity

207999_s_at	NM_001112	ADARB1	Homo sapiens	6-Oct-14	Exemplar sequence	GenBank	gb:NM_001112.1 /DEF=Homo sapiens adenosine deaminase, RNA-specific, B1 (homolog of rat RED1) (ADARB1), transcript variant DRADA2a, mRNA. /FEA=mRNA /GEN=ADARB1 /PROD=RNA-specific adenosine deaminase B1, isoformDRADA2a /DB_XREF=gi:4501918 /UG=Hs.85302 adenosine deaminase, RNA-specific, [illegible]	NM_001112	adenosine deaminase, RNA-specific, B1	ADARB1	104	NM_001033049 /// NM_001112 /// NM_001160230 /// NM_015833 /// NM_015834 /// NR_027672 /// NR_027673 /// NR_027674 /// NR_073200 /// XM_006723953 /// XM_006723954 /// XM_006723955 /// XM_006723956 /// XM_006723957 /// XM_006723958	0002376 // immune system process // inferred from electronic annotation /// 0006382 // adenosine to inosine editing // inferred from direct assay /// 0006382 // adenosine to inosine editing // traceable author statement /// 0006396 // RNA processing // inferred from direct assay /// 0006397 // mRNA processing // inferred from electronic annotation /// 0008285 // negative regulation of cell proliferation // inferred from direct assay /// 0010467 // gene expression // traceable author statement /// 0016553 // base conversion or substitution editing // inferred by curator /// 0016556 // mRNA modification // [illegible]	0005634 // nucleus // inferred from direct assay /// 0005654 // nucleoplasm // traceable author statement /// 0005730 // nucleolus // inferred from direct assay	0003723 // RNA binding // inferred from direct assay /// 0003725 // double-stranded RNA binding // inferred from direct assay /// 0003726 // double-stranded RNA adenosine deaminase activity // inferred from direct assay /// 0003729 // mRNA binding // traceable author statement /// 0004000 // adenosine deaminase activity // inferred from electronic annotation /// 0005515 // protein binding // inferred from physical interaction /// 0016787 // hydrolase activity // inferred from electronic annotation /// 0044822 // poly(A) RNA binding // inferred from direct assay /// 0046872 // metal ion binding // inferred from electronic annotation

208072_s_at	NM_003648	DGKD	Homo sapiens	6-Oct-14	Exemplar sequence	GenBank	gb:NM_003648.1 /DEF=Homo sapiens diacylglycerol kinase, delta (130kD) (DGKD), mRNA. /FEA=mRNA /GEN=DGKD /PROD=diacylglycerol kinase, delta (130kD) /DB_XREF=gi:4503310 /UG=Hs.115907 diacylglycerol kinase, delta (130kD) /FL=gb:D73409.1 gb:NM_003648.1	NM_003648	diacylglycerol kinase, delta 130kDa	DGKD	8527	NM_003648 /// NM_152879 /// XM_005246107 /// XM_005246108 /// XM_005246109 /// XM_005246110 /// XR_427118 /// XR_427119	0006810 // transport // inferred from electronic annotation /// 0006897 // endocytosis // inferred from electronic annotation /// 0007165 // signal transduction // traceable author statement /// 0007173 // epidermal growth factor receptor signaling pathway // non-traceable author statement /// 0007205 // protein kinase C-activating G-protein coupled receptor signaling pathway // non-traceable author statement /// 0007275 // multicellular organismal development // non-traceable author statement /// 0007596 // blood coagulation // traceable author statement /// 0008152 // metabolic process // inferred from electronic	0005737 // cytoplasm // inferred from direct assay /// 0005886 // plasma membrane // inferred from direct assay /// 0005886 // plasma membrane // traceable author statement /// 0016020 // membrane // inferred from electronic annotation /// 0016023 // cytoplasmic membrane-bounded vesicle // inferred from direct assay	0000166 // nucleotide binding // inferred from electronic annotation /// 0003951 // NAD+ kinase activity // inferred from electronic annotation /// 0004143 // diacylglycerol kinase activity // inferred from direct assay /// 0004143 // diacylglycerol kinase activity // traceable author statement /// 0005515 // protein binding // inferred from physical interaction /// 0005524 // ATP binding // inferred from electronic annotation /// 0005543 // phospholipid binding // inferred from electronic annotation /// 0016301 // kinase activity // inferred from electronic annotation /// 0016740 // transferase activity // inferred from electronic annotation /// 0019992 // diacylglycerol binding // non-traceable

208132_x_at	NM_004638	PRRC2A	Homo sapiens	6-Oct-14	Exemplar sequence	GenBank	gb:NM_004638.1 /DEF=Homo sapiens HLA-B associated transcript-2 (D6S51E), mRNA. /FEA=mRNA /GEN=D6S51E /PROD=HLA-B associated transcript-2 /DB_XREF=gi:4758107 /UG=Hs.25911 HLA-B associated transcript-2 /FL=gb:M33509.1 gb:NM_004638.1	NM_004638	proline-rich coiled-coil 2A	PRRC2A	7916	NM_004638 /// NM_080686 /// XM_006715191 /// XM_006725507 /// XM_006725508 /// XM_006725723 /// XM_006725724 /// XM_006725833 /// XM_006725834 /// XM_006725922 /// XM_006726023 /// XM_006726024 /// XM_006726111 /// XM_006726112		0005634 // nucleus // inferred from electronic annotation /// 0005737 // cytoplasm // inferred from electronic annotation /// 0016020 // membrane // inferred from direct assay /// 0070062 // extracellular vesicular exosome // inferred from direct assay	0005515 // protein binding // inferred from physical interaction /// 0044822 // poly(A) RNA binding // inferred from direct assay

208151_x_at	NM_030881	DDX17	Homo sapiens	6-Oct-14	Exemplar sequence	GenBank	gb:NM_030881.1 /DEF=Homo sapiens DEADH (Asp-Glu-Ala-AspHis) box polypeptide 17 (72kD) (DDX17), transcript variant 2, mRNA. /FEA=mRNA /GEN=DDX17 /PROD=DEADH (Asp-Glu-Ala-AspHis) box polypeptide 17,isoform 2 /DB_XREF=gi:13787203 /FL=gb:NM_030881.1	NM_030881	DEAD (Asp-Glu-Ala-Asp) box helicase 17	DDX17	10521	NM_001098504 /// NM_001098505 /// NM_006386 /// NM_030881	0006200 // ATP catabolic process // inferred from electronic annotation /// 0006200 // ATP catabolic process // traceable author statement /// 0006351 // transcription, DNA-templated // inferred from electronic annotation /// 0006355 // regulation of transcription, DNA-templated // inferred from electronic annotation /// 0006396 // RNA processing // traceable author statement /// 0008152 // metabolic process // inferred from electronic annotation /// 0009791 // post-embryonic development // inferred from electronic annotation /// 0033148 // positive regulation of intracellular estrogen [illegible]	0005634 // nucleus // inferred from direct assay /// 0005730 // nucleolus // inferred from electronic annotation /// 0016020 // membrane // inferred from direct assay	0000166 // nucleotide binding // inferred from electronic annotation /// 0003676 // nucleic acid binding // inferred from electronic annotation /// 0003713 // transcription coactivator activity // inferred from direct assay /// 0003723 // RNA binding // traceable author statement /// 0003724 // RNA helicase activity // traceable author statement /// 0004386 // helicase activity // inferred from electronic annotation /// 0005515 // protein binding // inferred from physical interaction /// 0005524 // ATP binding // inferred from electronic annotation /// 0008026 // ATP-dependent helicase activity // inferred from electronic annotation /// 0008186 // RNA-dependent ATPase activity // traceable [illegible]

208240_s_at	NM_013394	FGF1	Homo sapiens	6-Oct-14	Exemplar sequence	GenBank	gb:NM_013394.1 /DEF=Homo sapiens acid fibroblast growth factor-like protein (GLIO703), mRNA. /FEA=mRNA /GEN=GLIO703 /PROD=acid fibroblast growth factor-like protein /DB_XREF=gi:7019394 /UG=Hs.278954 acid fibroblast growth factor-like protein /FL=gb:AF211169.1 gb:NM_013394.1	NM_013394	fibroblast growth factor 1 (acidic)	FGF1	2246	NM_000800 /// NM_001144892 /// NM_001144934 /// NM_001144935 /// NM_001257205 /// NM_001257206 /// NM_001257207 /// NM_001257208 /// NM_001257209 /// NM_001257210 /// NM_001257211 /// NM_001257212 /// NM_033136 /// NM_033137 /// XM_005268390 /// [illegible]	0001525 // angiogenesis // inferred from electronic annotation /// 0001759 // organ induction // inferred from electronic annotation /// 0001934 // positive regulation of protein phosphorylation // inferred from electronic annotation /// 0007165 // signal transduction // non-traceable author statement /// 0007173 // epidermal growth factor receptor signaling pathway // traceable author statement /// 0007275 // multicellular organismal development // traceable author statement /// 0008284 // positive regulation of cell proliferation // inferred from genetic interaction /// 0008286 // insulin receptor signaling pathway // traceable [illegible]	0005576 // extracellular region // inferred from direct assay /// 0005576 // extracellular region // traceable author statement /// 0005578 // proteinaceous extracellular matrix // inferred from electronic annotation /// 0005615 // extracellular space // inferred from direct assay /// 0005634 // nucleus // inferred from direct assay /// 0005730 // nucleolus // inferred from direct assay /// 0005737 // cytoplasm // [illegible]	0005102 // receptor binding // inferred from electronic annotation /// 0005104 // fibroblast growth factor receptor binding // inferred from physical interaction /// 0005515 // protein binding // inferred from physical interaction /// 0008083 // growth factor activity // inferred from direct assay /// 0008201 // heparin binding // inferred from direct assay /// 0030544 // Hsp70 protein binding // inferred from electronic annotation /// 0044548 // S100 protein binding // inferred from physical interaction

208581_x_at	NM_005952	MT1X	Homo sapiens	6-Oct-14	Exemplar sequence	GenBank	gb:NM_005952.1 /DEF=Homo sapiens metallothionein 1X (MT1X), mRNA. /FEA=CDS /GEN=MT1X /PROD=metallothionein 1X /DB_XREF=gi:10835231 /UG=Hs.278462 metallothionein 1X /FL=gb:NM_005952.1	NM_005952	metallothionein 1X	MT1X	4501	NM_005952	0010038 // response to metal ion // traceable author statement /// 0036018 // cellular response to erythropoietin // inferred from expression pattern /// 0045926 // negative regulation of growth // inferred from sequence or structural similarity /// 0071276 // cellular response to cadmium ion // inferred from expression pattern /// 0071294 // cellular response to zinc ion // inferred from expression pattern	0005634 // nucleus // inferred from direct assay /// 0005737 // cytoplasm // inferred from direct assay /// 0048471 // perinuclear region of cytoplasm // inferred from sequence or structural similarity	0008270 // zinc ion binding // inferred from sequence or structural similarity /// 0046872 // metal ion binding // traceable author statement

208650_s_at	BG327863	CD24	Homo sapiens	6-Oct-14	Consensus sequence	GenBank	Consensus includes gb:BG327863 /FEA=EST /DB_XREF=gi:13134301 /DB_XREF=est:602426876F1 /CLONE=IMAGE:4564675 /UG=Hs.286124 CD24 antigen (small cell lung carcinoma cluster 4 antigen) /FL=gb:M58664.1 gb:L33930.1 gb:NM_013230.1	BG327863	CD24 molecule	CD24	1E+08	NM_001291737 /// NM_001291738 /// NM_001291739 /// NM_013230 /// NR_117089 /// NR_117090 /// XM_005278329	0001666 // response to hypoxia // inferred from expression pattern /// 0001775 // cell activation // inferred from direct assay /// 0001959 // regulation of cytokine-mediated signaling pathway // inferred from sequence or structural similarity /// 0002237 // response to molecule of bacterial origin // inferred from sequence or structural similarity /// 0002768 // immune response-regulating cell surface receptor signaling pathway // inferred by curator /// 0007155 // cell adhesion // inferred from electronic annotation /// 0007204 // positive regulation of cytosolic calcium ion concentration // inferred from direct assay /// 0007411 // axon [illegible]	0005886 // plasma membrane // inferred from electronic annotation /// 0009986 // cell surface // inferred from direct assay /// 0016020 // membrane // inferred from direct assay /// 0031225 // anchored component of membrane // inferred from electronic annotation /// 0045121 // membrane raft // inferred from direct assay	0004871 // signal transducer activity // non-traceable author statement /// 0005515 // protein binding // inferred from physical interaction /// 0019901 // protein kinase binding // inferred from physical interaction /// 0030296 // protein tyrosine kinase activator activity // inferred from direct assay

208651_x_at	M58664	CD24	Homo sapiens	6-Oct-14	Exemplar sequence	GenBank	gb:M58664.1 /DEF=Homo sapiens CD24 signal transducer mRNA, complete cds. /FEA=mRNA /PROD=signal transducer CD24 /DB_XREF=gi:180167 /UG=Hs.286124 CD24 antigen (small cell lung carcinoma cluster 4 antigen) /FL=gb:M58664.1 gb:L33930.1 gb:NM_013230.1	M58664	CD24 molecule	CD24	1E+08	NM_001291737 /// NM_001291738 /// NM_001291739 /// NM_013230 /// NR_117089 /// NR_117090 /// XM_005278329	0001666 // response to hypoxia // inferred from expression pattern /// 0001775 // cell activation // inferred from direct assay /// 0001959 // regulation of cytokine-mediated signaling pathway // inferred from sequence or structural similarity /// 0002237 // response to molecule of bacterial origin // inferred from sequence or structural similarity /// 0002768 // immune response-regulating cell surface receptor signaling pathway // inferred by curator /// 0007155 // cell adhesion // inferred from electronic annotation /// 0007204 // positive regulation of cytosolic calcium ion concentration // inferred from direct assay /// 0007411 // axon [illegible]	0005886 // plasma membrane // inferred from electronic annotation /// 0009986 // cell surface // inferred from direct assay /// 0016020 // membrane // inferred from direct assay /// 0031225 // anchored component of membrane // inferred from electronic annotation /// 0045121 // membrane raft // inferred from direct assay	0004871 // signal transducer activity // non-traceable author statement /// 0005515 // protein binding // inferred from physical interaction /// 0019901 // protein kinase binding // inferred from physical interaction /// 0030296 // protein tyrosine kinase activator activity // inferred from direct assay

208718_at	Z97056	DDX17	Homo sapiens	6-Oct-14	Consensus sequence	GenBank	Consensus includes gb:Z97056 /DEF=Human DNA sequence from clone RP3-434P1 on chromosome 22 Contains the KCNJ4 gene for inwardly rectifying potassium channel J4 (hippocampal inward rectifier, HIR, HRK1, HIRK2, KIR2.3), the KDELR3 gene for KDEL (Lys-Asp-Glu-Leu) endoplasmic reticulu... /FEA=mRNA_5 /DB_XREF=gi:2 832593	Z97056	DEAD (Asp-Glu-Ala-Asp) box helicase 17	DDX17	10521	NM_001098504 /// NM_001098505 /// NM_006386 /// NM_030881	0006200 // ATP catabolic process // inferred from electronic annotation /// 0006200 // ATP catabolic process // traceable author statement /// 0006351 // transcription, DNA-templated // inferred from electronic annotation /// 0006355 // regulation of transcription, DNA-templated // inferred from electronic annotation /// 0006396 // RNA processing // traceable author statement /// 0008152 // metabolic process // inferred from electronic annotation /// 0009791 // post-embryonic development // inferred from electronic annotation /// 0033148 // positive regulation of intracellular estrogen	0005634 // nucleus // inferred from direct assay /// 0005730 // nucleolus // inferred from electronic annotation /// 0016020 // membrane // inferred from direct assay	0000166 // nucleotide binding // inferred from electronic annotation /// 0003676 // nucleic acid binding // inferred from electronic annotation /// 0003713 // transcription coactivator activity // inferred from direct assay /// 0003723 // RNA binding // traceable author statement /// 0003724 // RNA helicase activity // traceable author statement /// 0004386 // helicase activity // inferred from electronic annotation /// 0005515 // protein binding // inferred from physical interaction /// 0005524 // ATP binding // inferred from electronic annotation /// 0008026 // ATP-dependent helicase activity // inferred from electronic annotation /// 0008186 // RNA-dependent ATPase activity // traceable

208719_s_at	U59321	DDX17	Homo sapiens	6-Oct-14	Exemplar sequence	GenBank	gb:U59321.1 /DEF=Human DEAD-box protein p72 (P72) mRNA, complete cds. /FEA=mRNA /GEN=P72 /PROD=DEAD-box protein p72 /DB_XREF=gi:1592564 /UG=Hs.6179 DEADH (Asp-Glu-Ala-AspHis) box polypeptide 17 (72kD) /FL=gb:BC000595.1 gb:NM_006386.2 gb:U59321.1	U59321	DEAD (Asp-Glu-Ala-Asp) box helicase 17	DDX17	10521	NM_001098504 /// NM_001098505 /// NM_006386 /// NM_030881	0006200 // ATP catabolic process // inferred from electronic annotation /// 0006200 // ATP catabolic process // traceable author statement /// 0006351 // transcription, DNA-templated // inferred from electronic annotation /// 0006355 // regulation of transcription, DNA-templated // inferred from electronic annotation /// 0006396 // RNA processing // traceable author statement /// 0008152 // metabolic process // inferred from electronic annotation /// 0009791 // post-embryonic development // inferred from electronic annotation /// 0033148 // positive regulation of intracellular estrogen [illegible]	0005634 // nucleus // inferred from direct assay /// 0005730 // nucleolus // inferred from electronic annotation /// 0016020 // membrane // inferred from direct assay	0000166 // nucleotide binding // inferred from electronic annotation /// 0003676 // nucleic acid binding // inferred from electronic annotation /// 0003713 // transcription coactivator activity // inferred from direct assay /// 0003723 // RNA binding // traceable author statement /// 0003724 // RNA helicase activity // traceable author statement /// 0004386 // helicase activity // inferred from electronic annotation /// 0005515 // protein binding // inferred from physical interaction /// 0005524 // ATP binding // inferred from electronic annotation /// 0008026 // ATP-dependent helicase activity // inferred from electronic annotation /// 0008186 // RNA-dependent ATPase activity // traceable [illegible]
208797_s_at	AI829170	GOLGA8A	Homo sapiens	6-Oct-14	Consensus sequence	GenBank	Consensus includes gb:AI829170 /FEA=EST /DB_XREF=gi:5449841 /DB_XREF=est:wk76b03.x1 /CLONE=IMAGE:2421293 /UG=Hs.182982 golgin-67 /FL=gb:NM_015003.1 gb:AF204231.1	AI829170	golgin A8 family, member A	GOLGA8A	23015	NM_181077 /// NR_027409 /// XM_006720440		0005794 // Golgi apparatus // inferred from electronic annotation /// 0016020 // membrane // inferred from electronic annotation /// 0032580 // Golgi cisterna membrane // inferred from electronic annotation	

208798_x_at	AF204231	GOLGA8A	Homo sapiens	6-Oct-14	Exemplar sequence	GenBank	gb:AF204231.1 /DEF=Homo sapiens 88-kDa Golgi protein (GM88) mRNA, complete cds. /FEA=mRNA /GEN=GM88 /PROD=88-kDa Golgi protein /DB_XREF=gi:6808610 /UG=Hs.182982 golgin-67 /FL=gb:NM_015003.1 gb:AF204231.1	AF204231	golgin A8 family, member A	GOLGA8A	23015	NM_181077 /// NR_027409 /// XM_006720440		0005794 // Golgi apparatus // inferred from electronic annotation /// 0016020 // membrane // inferred from electronic annotation /// 0032580 // Golgi cisterna membrane // inferred from electronic annotation	

209189_at	BC004490	FOS	Homo sapiens	6-Oct-14	Exemplar sequence	GenBank	gb:BC004490.1 /DEF=Homo sapiens, v-fos FBJ murine osteosarcoma viral oncogene homolog, clone MGC:11074, mRNA, complete cds. /FEA=mRNA /PROD=v-fos FBJ murine osteosarcoma viral oncogenehomolog /DB_XREF=gi:13325363 /UG=Hs.25647 v-fos FBJ murine osteosarcoma viral oncogene homolog /FL=gb:BC004490.1	BC004490	FBJ murine osteosarcoma viral oncogene homolog	FOS	2353	NM_005252	0001661 // conditioned taste aversion // inferred from electronic annotation /// 0002224 // toll-like receptor signaling pathway // traceable author statement /// 0002755 // MyD88-dependent toll-like receptor signaling pathway // traceable author statement /// 0002756 // MyD88-independent toll-like receptor signaling pathway // traceable author statement /// 0006306 // DNA methylation // traceable author statement /// 0006355 // regulation of transcription, DNA-templated // inferred from electronic annotation /// 0006357 // regulation of transcription from RNA polymerase II	0005634 // nucleus // inferred from direct assay /// 0005654 // nucleoplasm // traceable author statement /// 0005667 // transcription factor complex // inferred from electronic annotation /// 0005730 // nucleolus // inferred from direct assay /// 0005737 // cytoplasm // inferred from electronic annotation /// 0005783 // endoplasmic reticulum // inferred from electronic	0003677 // DNA binding // inferred from electronic annotation /// 0003690 // double-stranded DNA binding // inferred from electronic annotation /// 0003700 // sequence-specific DNA binding transcription factor activity // inferred from direct assay /// 0005515 // protein binding // inferred from physical interaction /// 0008134 // transcription factor binding // inferred from electronic annotation /// 0043565 // sequence-specific DNA binding // inferred from electronic annotation /// 0044212 // transcription regulatory region DNA binding // inferred from direct assay /// 0070412 // R-SMAD binding // inferred from physical interaction

209201_x _at	L01639	CXCR4	Homo sapiens	6-Oct-14	Exemplar sequence	GenBank	gb:L01639.1 /DEF=Human (clone HSY3RR) neuropeptide Y receptor (NPYR) mRNA, complete cds. /FEA=mRNA /GEN=NPYR /PROD=neuropeptide Y receptor /DB_XREF=gi:189313 /UG=Hs.89414 chemokine (C-X-C motif), receptor 4 (fusin) /FL=gb:L01639.1 gb:AF025375.1 gb:M99293.1 gb:L06797.1 gb:NM_003467.1 gb:AF147204.1	L01639	chemokine (C-X-C motif) receptor 4	CXCR4	7852	NM_001008540 /// NM_003467	0000187 // activation of MAPK activity // traceable author statement /// 0001569 // patterning of blood vessels // inferred from electronic annotation /// 0001666 // response to hypoxia // inferred from expression pattern /// 0001667 // ameboidal cell migration // inferred from electronic annotation /// 0001764 // neuron migration // inferred from electronic annotation /// 0002407 // dendritic cell chemotaxis // traceable author statement /// 0006915 // apoptotic process // traceable author statement /// 0006935 // chemotaxis // inferred from electronic annotation /// 0006954 // inflammatory response // traceable author [illegible]	0005737 // cytoplasm // traceable author statement /// 0005764 // lysosome // inferred from direct assay /// 0005768 // endosome // inferred from electronic annotation /// 0005769 // early endosome // inferred from direct assay /// 0005770 // late endosome // inferred from direct assay /// 0005886 // plasma membrane // inferred from direct assay /// 0005886 // plasma membrane // [illegible]	0001618 // virus receptor activity // inferred from electronic annotation /// 0003779 // actin binding // inferred from direct assay /// 0004871 // signal transducer activity // inferred from electronic annotation /// 0004930 // G-protein coupled receptor activity // traceable author statement /// 0004950 // chemokine receptor activity // inferred from electronic annotation /// 0005515 // protein binding // inferred from physical interaction /// 0015026 // coreceptor activity // traceable author statement /// 0016494 // C-X-C chemokine receptor activity // non-traceable author statement /// 0019955 // cytokine binding // inferred from electronic annotation /// 0031625 // ubiquitin protein ligase binding // [illegible]

209324_s_at	BF304996	RGS16	Homo sapiens	6-Oct-14	Consensus sequence	GenBank	Consensus includes gb:BF304996 /FEA=EST /DB_XREF=gi:11251810 /DB_XREF=est:601888511F1 /CLONE=IMAGE:4122242 /UG=Hs.183601 regulator of G-protein signalling 16 /FL=gb:U70426.1 gb:U94829.1 gb:NM_002928.1	BF304996	regulator of G-protein signaling 16	RGS16	6004	NM_002928	0007601 // visual perception // traceable author statement /// 0008277 // regulation of G-protein coupled receptor protein signaling pathway // traceable author statement /// 0009968 // negative regulation of signal transduction // inferred from electronic annotation /// 0038032 // termination of G-protein coupled receptor signaling pathway // inferred from electronic annotation /// 0043547 // positive regulation of GTPase activity // not recorded /// 0043547 // positive regulation of GTPase activity // traceable author statement	0005737 // cytoplasm // not recorded /// 0005886 // plasma membrane // not recorded	0005096 // GTPase activator activity // not recorded /// 0005515 // protein binding // inferred from physical interaction /// 0005516 // calmodulin binding // traceable author statement

209325_s_at	U94829	RGS16	Homo sapiens	6-Oct-14	Exemplar sequence	GenBank	gb:U94829.1 /DEF=Human retinally abundant regulator of G-protein signaling hRGS-r (hRGS-r) mRNA, complete cds. /FEA=mRNA /GEN=hRGS-r /PROD=retinally abundant regulator of G-proteinsignaling hRGS-r /DB_XREF=gi:2605639 /UG=Hs.183601 regulator of G-protein signalling 16 /FL=gb:U70426.1 gb:U94829.1 gb:NM_002928.1	U94829	regulator of G-protein signaling 16	RGS16	6004	NM_002928	0007601 // visual perception // traceable author statement /// 0008277 // regulation of G-protein coupled receptor protein signaling pathway // traceable author statement /// 0009968 // negative regulation of signal transduction // inferred from electronic annotation /// 0038032 // termination of G-protein coupled receptor signaling pathway // inferred from electronic annotation /// 0043547 // positive regulation of GTPase activity // not recorded /// 0043547 // positive regulation of GTPase activity // traceable author statement	0005737 // cytoplasm // not recorded /// 0005886 // plasma membrane // not recorded	0005096 // GTPase activator activity // not recorded /// 0005515 // protein binding // inferred from physical interaction /// 0005516 // calmodulin binding // traceable author statement

209372_x_at	BF971587	TUBB2A	Homo sapiens	6-Oct-14	Consensus sequence	GenBank	Consensus includes gb:BF971587 /FEA=EST /DB_XREF=gi:12338802 /DB_XREF=est:602239834F1 /CLONE=IMAGE:4328385 /UG=Hs.179661 tubulin, beta polypeptide /FL=gb:BC001352.1	BF971587	tubulin, beta 2A class IIa /// tubulin, beta 2B class IIb	TUBB2A /// TUBB2B	7280 /// 347733	NM_001069 /// NM_178012	0001764 // neuron migration // inferred from mutant phenotype /// 0006184 // GTP catabolic process // inferred from electronic annotation /// 0006457 // protein folding // traceable author statement /// 0007017 // microtubule-based process // inferred from electronic annotation /// 0044267 // cellular protein metabolic process // traceable author statement /// 0051084 // 'de novo' posttranslational protein folding // traceable author statement /// 0051258 // protein polymerization // inferred from electronic annotation	0005634 // nucleus // inferred from direct assay /// 0005737 // cytoplasm // inferred from electronic annotation /// 0005856 // cytoskeleton // inferred from electronic annotation /// 0005874 // microtubule // inferred from direct assay /// 0005886 // plasma membrane // inferred from direct assay /// 0015630 // microtubule cytoskeleton // inferred from direct assay /// 0043234 // protein [illegible]	0000166 // nucleotide binding // inferred from electronic annotation /// 0003924 // GTPase activity // inferred from electronic annotation /// 0005200 // structural constituent of cytoskeleton // inferred from electronic annotation /// 0005515 // protein binding // inferred from physical interaction /// 0005525 // GTP binding // inferred from electronic annotation

209430_at	AJ001017	BTAF1	Homo sapiens	6-Oct-14	Consensus sequence	GenBank	Consensus includes gb:AJ001017.2 /DEF=Homo sapiens partial mRNA for TBP-associated factor 170 (TAFII170). /FEA=mRNA /GEN=TAFII170 /PROD=TBP associated factor /DB_XREF=gi:7018281 /UG=Hs.180930 TBP-associated factor 172 /FL=gb:AF038362.1	AJ001017	BTAF1 RNA polymerase II, B-TFIID transcription factor-associated, 170kDa	BTAF1	9044	NM_003972 /// XM_005270265 /// XM_005270266 /// XR_246113	0008152 // metabolic process // inferred from electronic annotation /// 0035562 // negative regulation of chromatin binding // inferred from mutant phenotype /// 0045892 // negative regulation of transcription, DNA-templated // non-traceable author statement	0005634 // nucleus // inferred from electronic annotation	0000166 // nucleotide binding // inferred from electronic annotation /// 0003676 // nucleic acid binding // inferred from electronic annotation /// 0003677 // DNA binding // inferred from electronic annotation /// 0003700 // sequence-specific DNA binding transcription factor activity // non-traceable author statement /// 0004386 // helicase activity // inferred from electronic annotation /// 0005488 // binding // inferred from electronic annotation /// 0005515 // protein binding // inferred from electronic annotation /// 0005524 // ATP binding // inferred from electronic annotation /// 0016787 // hydrolase activity // inferred from electronic annotation

209497_s_at	BC003503	RBM4B	Homo sapiens	6-Oct-14	Exemplar sequence	GenBank	gb:BC003503.1 /DEF=Homo sapiens, Similar to RIKEN cDNA 4921506I22 gene, clone MGC:10380, mRNA, complete cds. /FEA=mRNA /PROD=Similar to RIKEN cDNA 4921506I22 gene /DB_XREF=gi:13097557 /UG=Hs.49994 Homo sapiens, clone MGC:10871, mRNA, complete cds /FL=gb:BC003503.1 gb:BC004951.1	BC003503	RNA binding motif protein 4B	RBM4B	83759	NM_001286135 /// NM_031492 /// XR_247213 /// XR_247214	0006397 // mRNA processing // inferred from electronic annotation /// 0006417 // regulation of translation // inferred from sequence or structural similarity /// 0007623 // circadian rhythm // inferred from sequence or structural similarity /// 0008380 // RNA splicing // inferred from electronic annotation /// 0010628 // positive regulation of gene expression // inferred from electronic annotation /// 0032922 // circadian regulation of gene expression // inferred from sequence or structural similarity /// 0043153 // entrainment of circadian clock by photoperiod // inferred from sequence or structural similarity	0005634 // nucleus // inferred from electronic annotation /// 0005730 // nucleolus // inferred from electronic annotation	0000166 // nucleotide binding // inferred from electronic annotation /// 0003676 // nucleic acid binding // inferred from electronic annotation /// 0003723 // RNA binding // inferred from electronic annotation /// 0005515 // protein binding // inferred from physical interaction /// 0008270 // zinc ion binding // inferred from electronic annotation /// 0044822 // poly(A) RNA binding // inferred from direct assay /// 0046872 // metal ion binding // inferred from electronic annotation

209579_s_at	AL556619	MBD4	Homo sapiens	6-Oct-14	Consensus sequence	GenBank	Consensus includes gb:AL556619 /FEA=EST /DB_XREF=gi:12899460 /DB_XREF=est:AL556619 /CLONE=CS0DK006YH06 (5 prime) /UG=Hs.35947 methyl-CpG binding domain protein 4 /FL=gb:AF072250.1 gb:NM_003925.1 gb:AF114784.1	AL556619	methyl-CpG binding domain protein 4	MBD4	8930	NM_001276270 /// NM_001276271 /// NM_001276272 /// NM_001276273 /// NM_003925	0000737 // DNA catabolic process, endonucleolytic // traceable author statement /// 0006281 // DNA repair // traceable author statement /// 0006284 // base-excision repair // traceable author statement /// 0006285 // base-excision repair, AP site formation // traceable author statement /// 0006974 // cellular response to DNA damage stimulus // inferred from electronic annotation /// 0007095 // mitotic G2 DNA damage checkpoint // inferred from electronic annotation /// 0008630 // intrinsic apoptotic signaling pathway in response to DNA damage // inferred from electronic annotation /// 0009314 // response to radiation // inferred from electronic [illegible]	0000785 // chromatin // inferred from electronic annotation /// 0005634 // nucleus // inferred from direct assay /// 0005654 // nucleoplasm // traceable author statement /// 0005730 // nucleolus // inferred from direct assay /// 0005737 // cytoplasm // inferred from electronic annotation	0003677 // DNA binding // inferred from electronic annotation /// 0003696 // satellite DNA binding // traceable author statement /// 0003824 // catalytic activity // inferred from electronic annotation /// 0004520 // endodeoxyribonuclease activity // traceable author statement /// 0005515 // protein binding // inferred from physical interaction /// 0008263 // pyrimidine-specific mismatch base pair DNA N-glycosylase activity // inferred from direct assay /// 0016787 // hydrolase activity // inferred from electronic annotation

209580_s_at	AF114784	MBD4	Homo sapiens	6-Oct-14	Exemplar sequence	GenBank	gb:AF114784.1 /DEF=Homo sapiens methyl-CpG binding endonuclease (MED1) mRNA, complete cds. /FEA=mRNA /GEN=MED1 /PROD=methyl-CpG binding endonuclease /DB_XREF=gi:4539758 /UG=Hs.35947 methyl-CpG binding domain protein 4 /FL=gb:AF072250.1 gb:NM_003925.1 gb:AF114784.1	AF114784	methyl-CpG binding domain protein 4	MBD4	8930	NM_001276270 /// NM_001276271 /// NM_001276272 /// NM_001276273 /// NM_003925	0000737 // DNA catabolic process, endonucleolytic // traceable author statement /// 0006281 // DNA repair // traceable author statement /// 0006284 // base-excision repair // traceable author statement /// 0006285 // base-excision repair, AP site formation // traceable author statement /// 0006974 // cellular response to DNA damage stimulus // inferred from electronic annotation /// 0007095 // mitotic G2 DNA damage checkpoint // inferred from electronic annotation /// 0008630 // intrinsic apoptotic signaling pathway in response to DNA damage // inferred from electronic annotation /// 0009314 // response to radiation // inferred from electronic [illegible]	0000785 // chromatin // inferred from electronic annotation /// 0005634 // nucleus // inferred from direct assay /// 0005654 // nucleoplasm // traceable author statement /// 0005730 // nucleolus // inferred from direct assay /// 0005737 // cytoplasm // inferred from electronic annotation	0003677 // DNA binding // inferred from electronic annotation /// 0003696 // satellite DNA binding // traceable author statement /// 0003824 // catalytic activity // inferred from electronic annotation /// 0004520 // endodeoxyribonuclease activity // traceable author statement /// 0005515 // protein binding // inferred from physical interaction /// 0008263 // pyrimidine-specific mismatch base pair DNA N-glycosylase activity // inferred from direct assay /// 0016787 // hydrolase activity // inferred from electronic annotation

209701_at	D16217	ERAP1	Homo sapiens	6-Oct-14	Consensus sequence	GenBank	Consensus includes gb:D16217.1 /DEF=Human mRNA for calpastatin, complete cds. /FEA=mRNA /GEN=CAST /PROD=calpastatin /DB_XREF=gi:303598 /UG=Hs.279607 calpastatin /FL=gb:D16217.1	D16217	endoplasmic reticulum aminopeptidase 1	ERAP1	51752	NM_001040458 /// NM_001198541 /// NM_016442 /// XM_005272015 /// XM_005272016	0001525 // angiogenesis // traceable author statement /// 0002376 // immune system process // inferred from electronic annotation /// 0002474 // antigen processing and presentation of peptide antigen via MHC class I // traceable author statement /// 0006508 // proteolysis // inferred from electronic annotation /// 0006509 // membrane protein ectodomain proteolysis // inferred from direct assay /// 0008217 // regulation of blood pressure // non-traceable author statement /// 0008217 // regulation of blood pressure // traceable author statement /// 0009617 // response to bacterium // inferred from expression pattern ///	0005576 // extracellular region // inferred from direct assay /// 0005615 // extracellular space // inferred from direct assay /// 0005737 // cytoplasm // inferred from electronic annotation /// 0005783 // endoplasmic reticulum // non-traceable author statement /// 0005788 // endoplasmic reticulum lumen // traceable author statement /// 0005789 // endoplasmic reticulum membrane //	0004177 // aminopeptidase activity // inferred from direct assay /// 0005138 // interleukin-6 receptor binding // inferred from physical interaction /// 0005151 // interleukin-1, Type II receptor binding // traceable author statement /// 0005515 // protein binding // inferred from physical interaction /// 0008233 // peptidase activity // inferred from electronic annotation /// 0008235 // metalloexopeptidase activity // inferred from direct assay /// 0008237 // metallopeptidase activity // inferred from electronic annotation /// 0008270 // zinc ion binding // non-traceable author statement /// 0016787 // hydrolase activity // inferred from electronic annotation /// 0046872 // metal ion binding // inferred from electronic

209732_at	BC005254	CLEC2B	Homo sapiens	6-Oct-14	Exemplar sequence	GenBank	gb:BC005254.1 /DEF=Homo sapiens, Similar to C-type (calcium dependent, carbohydrate-recognition domain) lectin, superfamily member 2 (activation-induced), clone MGC:12289, mRNA, complete cds. /FEA=mRNA /PROD=Similar to C-type (calcium dependent,carbohydrate-recognition domain) lectin, superfamilymember 2 (activation-[illegible]	BC005254	C-type lectin domain family 2, member B	CLEC2B	9976	NM_005127		0005887 // integral component of plasma membrane // traceable author statement /// 0016020 // membrane // inferred from electronic annotation /// 0016021 // integral component of membrane // inferred from electronic annotation	0030246 // carbohydrate binding // inferred from electronic annotation

209771_x_at	AA761181	CD24	Homo sapiens	6-Oct-14	Consensus sequence	GenBank	Consensus includes gb:AA761181 /FEA=EST /DB_XREF=gi:2810111 /DB_XREF=est:nz09g03.s1 /CLONE=IMAGE:1287316 /UG=Hs.286124 CD24 antigen (small cell lung carcinoma cluster 4 antigen) /FL=gb:X69397.1	AA761181	CD24 molecule	CD24	1E+08	NM_001291737 /// NM_001291738 /// NM_001291739 /// NM_013230 /// NR_117089 /// NR_117090 /// XM_005278329	0001666 // response to hypoxia // inferred from expression pattern /// 0001775 // cell activation // inferred from direct assay /// 0001959 // regulation of cytokine-mediated signaling pathway // inferred from sequence or structural similarity /// 0002237 // response to molecule of bacterial origin // inferred from sequence or structural similarity /// 0002768 // immune response-regulating cell surface receptor signaling pathway // inferred by curator /// 0007155 // cell adhesion // inferred from electronic annotation /// 0007204 // positive regulation of cytosolic calcium ion concentration // inferred from direct assay /// 0007411 // axon [illegible]	0005886 // plasma membrane // inferred from electronic annotation /// 0009986 // cell surface // inferred from direct assay /// 0016020 // membrane // inferred from direct assay /// 0031225 // anchored component of membrane // inferred from electronic annotation /// 0045121 // membrane raft // inferred from direct assay	0004871 // signal transducer activity // non-traceable author statement /// 0005515 // protein binding // inferred from physical interaction /// 0019901 // protein kinase binding // inferred from physical interaction /// 0030296 // protein tyrosine kinase activator activity // inferred from direct assay

209772_s_at	X69397	CD24	Homo sapiens	6-Oct-14	Exemplar sequence	GenBank	gb:X69397.1 /DEF=H.sapiens CD24 gene, complete CDS. /FEA=mRNA /GEN=CD24 /PROD=cell surface antigen /DB_XREF=gi:396167 /UG=Hs.286124 CD24 antigen (small cell lung carcinoma cluster 4 antigen) /FL=gb:X69397.1	X69397	CD24 molecule	CD24	1E+08	NM_001291737 /// NM_001291738 /// NM_001291739 /// NM_013230 /// NR_117089 /// NR_117090 /// XM_005278329	0001666 // response to hypoxia // inferred from expression pattern /// 0001775 // cell activation // inferred from direct assay /// 0001959 // regulation of cytokine-mediated signaling pathway // inferred from sequence or structural similarity /// 0002237 // response to molecule of bacterial origin // inferred from sequence or structural similarity /// 0002768 // immune response-regulating cell surface receptor signaling pathway // inferred by curator /// 0007155 // cell adhesion // inferred from electronic annotation /// 0007204 // positive regulation of cytosolic calcium ion concentration // inferred from direct assay /// 0007411 // axon [illegible]	0005886 // plasma membrane // inferred from electronic annotation /// 0009986 // cell surface // inferred from direct assay /// 0016020 // membrane // inferred from direct assay /// 0031225 // anchored component of membrane // inferred from electronic annotation /// 0045121 // membrane raft // inferred from direct assay	0004871 // signal transducer activity // non-traceable author statement /// 0005515 // protein binding // inferred from physical interaction /// 0019901 // protein kinase binding // inferred from physical interaction /// 0030296 // protein tyrosine kinase activator activity // inferred from direct assay

209780_at	AL136883	PHTF2	Homo sapiens	6-Oct-14	Exemplar sequence	GenBank	gb:AL136883.1 /DEF=Homo sapiens mRNA; cDNA DKFZp434D166 (from clone DKFZp434D166); complete cds. /FEA=mRNA /GEN=DKFZp434D166 /PROD=hypothetical protein /DB_XREF=gi:12053266 /UG=Hs.128653 hypothetical protein DKFZp564F013 /FL=gb:AL136883.1	AL136883	putative homeodomain transcription factor 2	PHTF2	57157	NM_001127357 /// NM_001127358 /// NM_001127359 /// NM_001127360 /// NM_020432 /// XM_005250508 /// XM_005250509 /// XM_006716063	0006351 // transcription, DNA-templated // inferred from electronic annotation /// 0006355 // regulation of transcription, DNA-templated // inferred from electronic annotation	0005634 // nucleus // inferred from electronic annotation /// 0005783 // endoplasmic reticulum // inferred from direct assay	0003677 // DNA binding // inferred from electronic annotation

209785_s_at	AF065214	PLA2G4C	Homo sapiens	6-Oct-14	Exemplar sequence	GenBank	gb:AF065214.1 /DEF=Homo sapiens cytosolic phospholipase A2 gamma (cPLA2 gamma) mRNA, complete cds. /FEA=mRNA /GEN=cPLA2 gamma /PROD=cytosolic phospholipase A2 gamma /DB_XREF=gi:3811344 /UG=Hs.18858 phospholipase A2, group IVC (cytosolic, calcium-independent) /FL=gb:AF058921.1 gb:AF065214.1	AF065214	phospholipase A2, group IVC (cytosolic, calcium-independent)	PLA2G4C	8605	NM_001159322 /// NM_001159323 /// NM_003706 /// XM_006723451 /// XM_006723452 /// XM_006723453	0006629 // lipid metabolic process // inferred from electronic annotation /// 0006644 // phospholipid metabolic process // traceable author statement /// 0006954 // inflammatory response // non-traceable author statement /// 0007567 // parturition // non-traceable author statement /// 0008152 // metabolic process // inferred from electronic annotation /// 0009395 // phospholipid catabolic process // inferred from electronic annotation /// 0016042 // lipid catabolic process // inferred from electronic annotation /// 0019369 // arachidonic acid metabolic process // non-traceable author statement /// 0035556 // intracellular signal	0005635 // nuclear envelope // inferred from electronic annotation /// 0005654 // nucleoplasm // inferred from electronic annotation /// 0005789 // endoplasmic reticulum membrane // traceable author statement /// 0005829 // cytosol // traceable author statement /// 0005938 // cell cortex // inferred from electronic annotation /// 0016020 // membrane // traceable author statement	0004620 // phospholipase activity // inferred from electronic annotation /// 0004623 // phospholipase A2 activity // inferred from electronic annotation /// 0005543 // phospholipid binding // non-traceable author statement /// 0016787 // hydrolase activity // inferred from electronic annotation /// 0047499 // calcium-independent phospholipase A2 activity // inferred from direct assay

209788_s_at	AF183569	ERAP1	Homo sapiens	6-Oct-14	Exemplar sequence	GenBank	gb:AF183569.1 /DEF=Homo sapiens aminopeptidase PILS (APPILS) mRNA, complete cds. /FEA=mRNA /GEN=APPILS /PROD=aminopeptidase PILS /DB_XREF=gi:6642986 /UG=Hs.247043 type 1 tumor necrosis factor receptor shedding aminopeptidase regulator /FL=gb:AF183569.1 gb:AF222340.1 gb:NM_016442.1	AF183569	endoplasmic reticulum aminopeptidase 1	ERAP1	51752	NM_001040458 /// NM_001198541 /// NM_016442 /// XM_005272015 /// XM_005272016	0001525 // angiogenesis // traceable author statement /// 0002376 // immune system process // inferred from electronic annotation /// 0002474 // antigen processing and presentation of peptide antigen via MHC class I // traceable author statement /// 0006508 // proteolysis // inferred from electronic annotation /// 0006509 // membrane protein ectodomain proteolysis // inferred from direct assay /// 0008217 // regulation of blood pressure // non-traceable author statement /// 0008217 // regulation of blood pressure // traceable author statement /// 0009617 // response to bacterium // inferred from expression pattern /// [illegible]	0005576 // extracellular region // inferred from direct assay /// 0005615 // extracellular space // inferred from direct assay /// 0005737 // cytoplasm // inferred from electronic annotation /// 0005783 // endoplasmic reticulum // non-traceable author statement /// 0005788 // endoplasmic reticulum lumen // traceable author statement /// 0005789 // endoplasmic reticulum membrane // [illegible]	0004177 // aminopeptidase activity // inferred from direct assay /// 0005138 // interleukin-6 receptor binding // inferred from physical interaction /// 0005151 // interleukin-1, Type II receptor binding // traceable author statement /// 0005515 // protein binding // inferred from physical interaction /// 0008233 // peptidase activity // inferred from electronic annotation /// 0008235 // metalloexopeptidase activity // inferred from direct assay /// 0008237 // metallopeptidase activity // inferred from electronic annotation /// 0008270 // zinc ion binding // non-traceable author statement /// 0016787 // hydrolase activity // inferred from electronic annotation /// 0046872 // metal ion binding // inferred from electronic
209890_at	AF065389	TSPAN5	Homo sapiens	6-Oct-14	Exemplar sequence	GenBank	gb:AF065389.1 /DEF=Homo sapiens tetraspan NET-4 mRNA, complete cds. /FEA=mRNA /PROD=tetraspan NET-4 /DB_XREF=gi:3152702 /UG=Hs.20709 tetraspan 5 /FL=gb:AF065389.1 gb:NM_005723.1	AF065389	tetraspanin 5	TSPAN5	10098	NM_005723 /// XM_005262680 /// XR_244617	0045747 // positive regulation of Notch signaling pathway // inferred from mutant phenotype /// 0051604 // protein maturation // inferred from mutant phenotype /// 0090002 // establishment of protein localization to plasma membrane // inferred from mutant phenotype	0005886 // plasma membrane // inferred from direct assay /// 0016020 // membrane // inferred from electronic annotation /// 0016021 // integral component of membrane // inferred from electronic annotation	0019899 // enzyme binding // inferred from physical interaction

209979_at	AF001042	ADARB1	Homo sapiens	6-Oct-14	Exemplar sequence	GenBank	gb:AF001042.1 /DEF=Homo sapiens RNA editase (RED1) mRNA, complete cds. /FEA=mRNA /GEN=RED1 /PROD=RNA editase /DB_XREF=gi:2114492 /UG=Hs.85302 adenosine deaminase, RNA-specific, B1 (homolog of rat RED1) /FL=gb:AF001042.1	AF001042	adenosine deaminase, RNA-specific, B1	ADARB1	104	NM_001033049 /// NM_001112 /// NM_001160230 /// NM_015833 /// NM_015834 /// NR_027672 /// NR_027673 /// NR_027674 /// NR_073200 /// XM_006723953 /// XM_006723954 /// XM_006723955 /// XM_006723956 /// XM_006723957 /// XM_006723958	0002376 // immune system process // inferred from electronic annotation /// 0006382 // adenosine to inosine editing // inferred from direct assay /// 0006382 // adenosine to inosine editing // traceable author statement /// 0006396 // RNA processing // inferred from direct assay /// 0006397 // mRNA processing // inferred from electronic annotation /// 0008285 // negative regulation of cell proliferation // inferred from direct assay /// 0010467 // gene expression // traceable author statement /// 0016553 // base conversion or substitution editing // inferred by curator /// 0016556 // mRNA modification // [illegible]	0005634 // nucleus // inferred from direct assay /// 0005654 // nucleoplasm // traceable author statement /// 0005730 // nucleolus // inferred from direct assay	0003723 // RNA binding // inferred from direct assay /// 0003725 // double-stranded RNA binding // inferred from direct assay /// 0003726 // double-stranded RNA adenosine deaminase activity // inferred from direct assay /// 0003729 // mRNA binding // traceable author statement /// 0004000 // adenosine deaminase activity // inferred from electronic annotation /// 0005515 // protein binding // inferred from physical interaction /// 0016787 // hydrolase activity // inferred from electronic annotation /// 0044822 // poly(A) RNA binding // inferred from direct assay /// 0046872 // metal ion binding // inferred from electronic annotation

210385_s_at	AF106037	ERAP1	Homo sapiens	6-Oct-14	Exemplar sequence	GenBank	gb:AF106037.1 /DEF=Homo sapiens adipocyte-derived leucine aminopeptidase mRNA, complete cds. /FEA=mRNA /PROD=adipocyte-derived leucine aminopeptidase /DB_XREF=gi:6381988 /UG=Hs.247043 type 1 tumor necrosis factor receptor shedding aminopeptidase regulator /FL=gb:AF106037.1	AF106037	endoplasmic reticulum aminopeptidase 1	ERAP1	51752	NM_001040458 /// NM_001198541 /// NM_016442 /// XM_005272015 /// XM_005272016	0001525 // angiogenesis // traceable author statement /// 0002376 // immune system process // inferred from electronic annotation /// 0002474 // antigen processing and presentation of peptide antigen via MHC class I // traceable author statement /// 0006508 // proteolysis // inferred from electronic annotation /// 0006509 // membrane protein ectodomain proteolysis // inferred from direct assay /// 0008217 // regulation of blood pressure // non-traceable author statement /// 0008217 // regulation of blood pressure // traceable author statement /// 0009617 // response to bacterium // inferred from expression pattern /// [illegible]	0005576 // extracellular region // inferred from direct assay /// 0005615 // extracellular space // inferred from direct assay /// 0005737 // cytoplasm // inferred from electronic annotation /// 0005783 // endoplasmic reticulum // non-traceable author statement /// 0005788 // endoplasmic reticulum lumen // traceable author statement /// 0005789 // endoplasmic reticulum membrane // [illegible]	0004177 // aminopeptidase activity // inferred from direct assay /// 0005138 // interleukin-6 receptor binding // inferred from physical interaction /// 0005151 // interleukin-1, Type II receptor binding // traceable author statement /// 0005515 // protein binding // inferred from physical interaction /// 0008233 // peptidase activity // inferred from electronic annotation /// 0008235 // metalloexopeptidase activity // inferred from direct assay /// 0008237 // metallopeptidase activity // inferred from electronic annotation /// 0008270 // zinc ion binding // non-traceable author statement /// 0016787 // hydrolase activity // inferred from electronic annotation /// 0046872 // metal ion binding // inferred from electronic

210424_s_at	AF163441	GOLGA8A	Homo sapiens	6-Oct-14	Exemplar sequence	GenBank	gb:AF163441.1 /DEF=Homo sapiens golgin 67 mRNA, complete cds. /FEA=mRNA /PROD=golgin 67 /DB_XREF=gi:6969979 /UG=Hs.182982 golgin-67 /FL=gb:AF163441.1 gb:AF164622.1	AF163441	golgin A8 family, member A /// golgin A8 family, member B /// uncharacterized LOC101930583	GOLGA8A /// GOLGA8B /// LOC101930583	23015 /// 440270 /// 101930583	NM_001023567 /// NM_181077 /// NR_027409 /// NR_027410 /// XM_005254389 /// XM_005254391 /// XM_006720440 /// XM_006720530 /// XR_248092 /// XR_248093 /// XR_248094 /// XR_248096 /// XR_424599 /// XR_424600 /// XR_424601 /// XR_424602		0005794 // Golgi apparatus // inferred from electronic annotation /// 0016020 // membrane // inferred from electronic annotation /// 0032580 // Golgi cisterna membrane // inferred from electronic annotation	
210425_x_at	AF164622	GOLGA8A	Homo sapiens	6-Oct-14	Exemplar sequence	GenBank	gb:AF164622.1 /DEF=Homo sapiens golgin-67 (GOLGA5) mRNA, complete cds. /FEA=mRNA /GEN=GOLGA5 /PROD=golgin-67 /DB_XREF=gi:7211437 /UG=Hs.182982 golgin-67 /FL=gb:AF163441.1 gb:AF164622.1	AF164622	golgin A8 family, member A /// golgin A8 family, member B /// uncharacterized LOC101930583	GOLGA8A /// GOLGA8B /// LOC101930583	23015 /// 440270 /// 101930583	NM_001023567 /// NM_181077 /// NR_027409 /// NR_027410 /// XM_005254389 /// XM_005254391 /// XM_006720440 /// XM_006720530 /// XR_248092 /// XR_248093 /// XR_248094 /// XR_248096 /// XR_424599 /// XR_424600 /// XR_424601 /// XR_424602		0005794 // Golgi apparatus // inferred from electronic annotation /// 0016020 // membrane // inferred from electronic annotation /// 0032580 // Golgi cisterna membrane // inferred from electronic annotation	

210655_s_at	AF041336	FOXO3	Homo sapiens	6-Oct-14	Exemplar sequence	GenBank	gb:AF041336.1 /DEF=Homo sapiens fork head-like protein (FKHRL1P2) mRNA, complete cds. /FEA=mRNA /GEN=FKHRL1P2 /PROD=fork head-like protein /DB_XREF=gi:4106722 /UG=Hs.14845 forkhead box O3A /FL=gb:AF041336.1	AF041336	forkhead box O3 /// forkhead box O3B pseudogene	FOXO3 /// FOXO3B	2309 /// 2310	NM_001455 /// NM_201559 /// NR_026718 /// XM_005266867 /// XM_005266868	0000122 // negative regulation of transcription from RNA polymerase II promoter // inferred from mutant phenotype /// 0001542 // ovulation from ovarian follicle // inferred from electronic annotation /// 0001544 // initiation of primordial ovarian follicle growth // inferred from electronic annotation /// 0001547 // antral ovarian follicle growth // inferred from electronic annotation /// 0001556 // oocyte maturation // inferred from electronic annotation /// 0006351 // transcription, DNA-templated // inferred from electronic annotation /// 0006355 // regulation of transcription, DNA-templated // inferred [illegible]	0005634 // nucleus // inferred from direct assay /// 0005654 // nucleoplasm // traceable author statement /// 0005737 // cytoplasm // inferred from direct assay /// 0005829 // cytosol // not recorded /// 0016020 // membrane // inferred from electronic annotation	0001047 // core promoter binding // inferred from sequence or structural similarity /// 0003677 // DNA binding // inferred from direct assay /// 0003700 // sequence-specific DNA binding transcription factor activity // inferred from direct assay /// 0005515 // protein binding // inferred from physical interaction /// 0008301 // DNA binding, bending // not recorded /// 0019901 // protein kinase binding // inferred from physical interaction /// 0031490 // chromatin DNA binding // inferred from sequence or structural similarity /// 0043565 // sequence-specific DNA binding // inferred from direct assay

210672_s_at	BC004185	NPRL3	Homo sapiens	6-Oct-14	Exemplar sequence	GenBank	gb:BC004185.1 /DEF=Homo sapiens, Similar to Conserved gene telomeric to alpha globin cluster, clone MGC:2816, mRNA, complete cds. /FEA=mRNA /PROD=Similar to Conserved gene telomeric to alphaglobin cluster /DB_XREF=gi:13278839 /UG=Hs.19699 Conserved gene telomeric to alpha globin cluster /FL=gb:BC004185.1	BC004185	nitrogen permease regulator-like 3 (S. cerevisiae)	NPRL3	8131	NM_001039476 /// NM_001077350 /// NM_001243247 /// NM_001243248 /// NM_001243249 /// NM_012075	0003281 // ventricular septum development // inferred from electronic annotation /// 0035909 // aorta morphogenesis // inferred from electronic annotation /// 0043547 // positive regulation of GTPase activity // inferred from electronic annotation /// 0048738 // cardiac muscle tissue development // inferred from electronic annotation /// 0060021 // palate development // inferred from electronic annotation		0005096 // GTPase activator activity // inferred from electronic annotation
210794_s_at	AF119863	MEG3	Homo sapiens	6-Oct-14	Consensus sequence	GenBank	Consensus includes gb:AF119863.1 /DEF=Homo sapiens PRO2160 mRNA, complete cds. /FEA=mRNA /PROD=PRO2160 /DB_XREF=gi:7770162 /UG=Hs.112844 maternally expressed 3 /FL=gb:AF119863.1	AF119863	maternally expressed 3 (non-protein coding)	MEG3	55384	NR_002766 /// NR_003530 /// NR_003531 /// NR_033358 /// NR_033359 /// NR_033360 /// NR_046464 /// NR_046465 /// NR_046466 /// NR_046467 /// NR_046468 /// NR_046469 /// NR_046470 /// NR_046471 /// NR_046472 /// NR_046473			

211020_at	L19659	GCNT2	Homo sapiens	6-Oct-14	Exemplar sequence	GenBank	gb:L19659.1 /DEF=Human I beta 1-6 N-acetylglucosaminyltransferase mRNA, complete cds. /FEA=mRNA /PROD=I beta 1-6 N-acetylglucosaminyltransferase /DB_XREF=gi:307297 /UG=Hs.934 glucosaminyl (N-acetyl) transferase 2, I-branching enzyme /FL=gb:L19659.1 gb:NM_001491.1	L19659	glucosaminyl (N-acetyl) transferase 2, I-branching enzyme (I blood group)	GCNT2	2651	NM_001491 /// NM_145649 /// NM_145655 /// XM_005248997 /// XM_005248999 /// XM_006715052 /// XM_006715053 /// XM_006715054 /// XM_006715055	0006024 // glycosaminoglycan biosynthetic process // traceable author statement /// 0006486 // protein glycosylation // inferred from electronic annotation /// 0006486 // protein glycosylation // inferred from mutant phenotype /// 0007179 // transforming growth factor beta receptor signaling pathway // inferred from mutant phenotype /// 0007179 // transforming growth factor beta receptor signaling pathway // inferred from sequence or structural similarity /// 0007275 // multicellular organismal development // traceable author statement /// 0008152 // metabolic process // inferred from electronic [illegible]	0000139 // Golgi membrane // inferred from electronic annotation /// 0005794 // Golgi apparatus // inferred from electronic annotation /// 0016020 // membrane // traceable author statement /// 0016021 // integral component of membrane // inferred from electronic annotation	0008109 // N-acetyllactosaminide beta-1,6-N-acetylglucosaminyltransferase activity // inferred from electronic annotation /// 0008109 // N-acetyllactosaminide beta-1,6-N-acetylglucosaminyltransferase activity // inferred from mutant phenotype /// 0008375 // acetylglucosaminyltransferase activity // inferred from electronic annotation /// 0016740 // transferase activity // inferred from electronic annotation /// 0016757 // transferase activity, transferring glycosyl groups // inferred from electronic annotation

211154_at	D32047	THPO	Homo sapiens	6-Oct-14	Exemplar sequence	GenBank	gb:D32047.1 /DEF=Homo sapiens mRNA for thrombopoietin, complete cds. /FEA=mRNA /PROD=thrombopoietin /DB_XREF=gi:2351117 /UG=Hs.1166 thrombopoietin (myeloproliferative leukemia virus oncogene ligand, megakaryocyte growth and development factor) /FL=gb:D32047.1	D32047	thrombopoietin	THPO	7066	NM_000460 /// NM_001177597 /// NM_001177598 /// NM_001289997 /// NM_001289998 /// NM_001290003 /// NM_001290022 /// NM_001290026 /// NM_001290027 /// NM_001290028 /// NM_199228 /// NM_199356 /// XM_005247737 /// XM_006713738 /// XM_006713739	0001934 // positive regulation of protein phosphorylation // inferred from sequence or structural similarity /// 0007275 // multicellular organismal development // traceable author statement /// 0007596 // blood coagulation // traceable author statement /// 0008283 // cell proliferation // inferred from electronic annotation /// 0030099 // myeloid cell differentiation // inferred from electronic annotation /// 0030168 // platelet activation // traceable author statement /// 0038163 // thrombopoietin-mediated signaling pathway // inferred from sequence or structural similarity /// 0045654 // positive [illegible]	0005576 // extracellular region // inferred from electronic annotation /// 0005615 // extracellular space // inferred from electronic annotation	0005125 // cytokine activity // inferred from electronic annotation /// 0005179 // hormone activity // inferred from electronic annotation /// 0008083 // growth factor activity // traceable author statement

211155_s_at	D32047	THPO	Homo sapiens	6-Oct-14	Exemplar sequence	GenBank	gb:D32047.1 /DEF=Homo sapiens mRNA for thrombopoietin, complete cds. /FEA=mRNA /PROD=thrombopoietin /DB_XREF=gi:2351117 /UG=Hs.1166 thrombopoietin (myeloproliferative leukemia virus oncogene ligand, megakaryocyte growth and development factor) /FL=gb:D32047.1	D32047	thrombopoietin	THPO	7066	NM_000460 /// NM_001177597 /// NM_001177598 /// NM_001289997 /// NM_001289998 /// NM_001290003 /// NM_001290022 /// NM_001290026 /// NM_001290027 /// NM_001290028 /// NM_199228 /// NM_199356 /// XM_005247737 /// XM_006713738 /// XM_006713739	0001934 // positive regulation of protein phosphorylation // inferred from sequence or structural similarity /// 0007275 // multicellular organismal development // traceable author statement /// 0007596 // blood coagulation // traceable author statement /// 0008283 // cell proliferation // inferred from electronic annotation /// 0030099 // myeloid cell differentiation // inferred from electronic annotation /// 0030168 // platelet activation // traceable author statement /// 0038163 // thrombopoietin-mediated signaling pathway // inferred from sequence or structural similarity /// 0045654 // positive [illegible]	0005576 // extracellular region // inferred from electronic annotation /// 0005615 // extracellular space // inferred from electronic annotation	0005125 // cytokine activity // inferred from electronic annotation /// 0005179 // hormone activity // inferred from electronic annotation /// 0008083 // growth factor activity // traceable author statement

211186_s_at	BC002695	AAK1	Homo sapiens	6-Oct-14	Exemplar sequence	GenBank	gb:BC002695.1 /DEF=Homo sapiens, Similar to KIAA1048 protein, clone MGC:3535, mRNA, complete cds. /FEA=mRNA /PROD=Similar to KIAA1048 protein /DB_XREF=gi:12803718 /UG=Hs.135941 KIAA1048 protein /FL=gb:BC002695.1	BC002695	AP2 associated kinase 1	AAK1	22848	NM_014911	0006468 // protein phosphorylation // inferred from direct assay /// 0006897 // endocytosis // inferred from electronic annotation /// 0016310 // phosphorylation // inferred from electronic annotation /// 0032880 // regulation of protein localization // inferred from direct assay /// 0045747 // positive regulation of Notch signaling pathway // inferred from direct assay /// 0046777 // protein autophosphorylation // inferred from sequence or structural similarity /// 0050821 // protein stabilization // inferred from direct assay /// 2000369 // regulation of clathrin-mediated endocytosis // inferred [illegible]	0005886 // plasma membrane // inferred from electronic annotation /// 0005905 // coated pit // inferred from electronic annotation /// 0016020 // membrane // inferred from electronic annotation /// 0019897 // extrinsic component of plasma membrane // inferred from sequence or structural similarity /// 0030136 // clathrin-coated vesicle // inferred from sequence or structural [illegible]	0000166 // nucleotide binding // inferred from electronic annotation /// 0004672 // protein kinase activity // inferred from electronic annotation /// 0004674 // protein serine/threonine kinase activity // inferred from sequence or structural similarity /// 0004713 // protein tyrosine kinase activity // inferred from electronic annotation /// 0005112 // Notch binding // inferred from direct assay /// 0005515 // protein binding // inferred from physical interaction /// 0005524 // ATP binding // inferred from electronic annotation /// 0016301 // kinase activity // inferred from electronic annotation /// 0016740 // transferase activity // inferred from electronic annotation /// 0016772 // transferase [illegible]

211299_s_at	BC003683	FLOT2	Homo sapiens	6-Oct-14	Exemplar sequence	GenBank	gb:BC003683.1 /DEF=Homo sapiens, Similar to flotillin 2, clone MGC:5052, mRNA, complete cds. /FEA=mRNA /PROD=Similar to flotillin 2 /DB_XREF=gi:13277549 /UG=Hs.184488 flotillin 2 /FL=gb:BC003683.1	BC003683	flotillin 2	FLOT2	2319	NM_004475 /// XM_005257950 /// XM_005257952 /// XM_005257953	0007155 // cell adhesion // inferred from electronic annotation /// 0008544 // epidermis development // traceable author statement /// 0010629 // negative regulation of gene expression // inferred from mutant phenotype /// 0050821 // protein stabilization // inferred from mutant phenotype /// 0090002 // establishment of protein localization to plasma membrane // inferred from mutant phenotype /// 1902992 // negative regulation of amyloid precursor protein catabolic process // inferred from mutant phenotype	0002080 // acrosomal membrane // inferred from electronic annotation /// 0005768 // endosome // inferred from direct assay /// 0005886 // plasma membrane // non-traceable author statement /// 0005901 // caveola // inferred from direct assay /// 0009986 // cell surface // inferred from direct assay /// 0016020 // membrane // inferred from direct assay /// 0016020 // membrane // inferred from [illegible]	0005515 // protein binding // inferred from physical interaction /// 0035255 // ionotropic glutamate receptor binding // inferred from electronic annotation

211305_x_at	U56236	FCAR	Homo sapiens	6-Oct-14	Exemplar sequence	GenBank	gb:U56236.1 /DEF=Human Fc alpha receptor b mRNA, complete cds. /FEA=mRNA /PROD=Fcalpha Rb /DB_XREF=gi:1326228 /UG=Hs.193122 Fc fragment of IgA, receptor for /FL=gb:U56236.1	U56236	Fc fragment of IgA, receptor for	FCAR	2204	NM_002000 /// NM_133269 /// NM_133271 /// NM_133272 /// NM_133273 /// NM_133274 /// NM_133277 /// NM_133278 /// NM_133279 /// NM_133280	0006955 // immune response // traceable author statement	0005576 // extracellular region // inferred from electronic annotation /// 0005886 // plasma membrane // inferred from direct assay /// 0005887 // integral component of plasma membrane // traceable author statement /// 0016020 // membrane // inferred from electronic annotation /// 0016021 // integral component of membrane // inferred from electronic annotation	0019862 // IgA binding // inferred from electronic annotation

211306_s_at	U56237	FCAR	Homo sapiens	6-Oct-14	Exemplar sequence	GenBank	gb:U56237.1 /DEF=Human Fc alpha receptor b deltaS2 mRNA, complete cds. /FEA=mRNA /PROD=FcalphaRbdeltaS2 /DB_XREF=gi:1326230 /UG=Hs.193122 Fc fragment of IgA, receptor for /FL=gb:U56237.1	U56237	Fc fragment of IgA, receptor for	FCAR	2204	NM_002000 /// NM_133269 /// NM_133271 /// NM_133272 /// NM_133273 /// NM_133274 /// NM_133277 /// NM_133278 /// NM_133279 /// NM_133280	0006955 // immune response // traceable author statement	0005576 // extracellular region // inferred from electronic annotation /// 0005886 // plasma membrane // inferred from direct assay /// 0005887 // integral component of plasma membrane // traceable author statement /// 0016020 // membrane // inferred from electronic annotation /// 0016021 // integral component of membrane // inferred from electronic annotation	0019862 // IgA binding // inferred from electronic annotation

211307_s_at	U43677	FCAR	Homo sapiens	6-Oct-14	Exemplar sequence	GenBank	gb:U43677.1 /DEF=Human Fc alpha receptor, splice variant FcalphaR a.3 (CD89) mRNA, complete cds. /FEA=mRNA /GEN=CD89 /PROD=Fc alpha receptor /DB_XREF=gi:1439557 /UG=Hs.193122 Fc fragment of IgA, receptor for /FL=gb:U43677.1 gb:D87854.1 gb:D87856.1	U43677	Fc fragment of IgA, receptor for	FCAR	2204	NM_002000 /// NM_133269 /// NM_133271 /// NM_133272 /// NM_133273 /// NM_133274 /// NM_133277 /// NM_133278 /// NM_133279 /// NM_133280	0006955 // immune response // traceable author statement	0005576 // extracellular region // inferred from electronic annotation /// 0005886 // plasma membrane // inferred from direct assay /// 0005887 // integral component of plasma membrane // traceable author statement /// 0016020 // membrane // inferred from electronic annotation /// 0016021 // integral component of membrane // inferred from electronic annotation	0019862 // IgA binding // inferred from electronic annotation

211385_x_at	U28169	SULT1A2	Homo sapiens	6-Oct-14	Exemplar sequence	GenBank	gb:U28169.1 /DEF=Human clone hast4v aryl sulfotransferase mRNA, complete cds. /FEA=mRNA /PROD=aryl sulfotransferase /DB_XREF=gi:881502 /UG=Hs.272462 sulfotransferase family, cytosolic, 1A, phenol-preferring, member 2 /FL=gb:U28169.1 gb:U28170.1	U28169	sulfotransferase family, cytosolic, 1A, phenol-preferring, member 2	SULT1A2	6799	NM_001054 /// NM_177528 /// XM_006721076 /// XM_006721077	0006584 // catecholamine metabolic process // inferred from electronic annotation /// 0006629 // lipid metabolic process // inferred from electronic annotation /// 0006805 // xenobiotic metabolic process // inferred from direct assay /// 0006805 // xenobiotic metabolic process // traceable author statement /// 0008152 // metabolic process // inferred from electronic annotation /// 0008202 // steroid metabolic process // inferred from electronic annotation /// 0009309 // amine biosynthetic process // traceable author statement /// 0018958 // phenol-containing compound metabolic process // inferred from direct assay [illegible]	0005737 // cytoplasm // inferred from electronic annotation /// 0005829 // cytosol // traceable author statement	0004062 // aryl sulfotransferase activity // inferred from direct assay /// 0008146 // sulfotransferase activity // traceable author statement /// 0016740 // transferase activity // inferred from electronic annotation /// 0047894 // flavonol 3-sulfotransferase activity // inferred from direct assay
211596_s_at	AB050468	LRIG1	Homo sapiens	6-Oct-14	Exemplar sequence	GenBank	gb:AB050468.1 /DEF=Homo sapiens mRNA for membrane glycoprotein LIG-1, complete cds. /FEA=mRNA /GEN=lig-1 /PROD=membrane glycoprotein LIG-1 /DB_XREF=gi:13537354 /FL=gb:AB050468.1	AB050468	leucine-rich repeats and immunoglobulin-like domains 1	LRIG1	26018	NM_015541		0016020 // membrane // inferred from electronic annotation /// 0016021 // integral component of membrane // inferred from electronic annotation	0005515 // protein binding // inferred from electronic annotation

211651_s_at	M20206	LAMB1	Homo sapiens	6-Oct-14	Exemplar sequence	GenBank	gb:M20206.1 /DEF=Human laminin B1 mRNA, complete cds. /FEA=mRNA /GEN=LAMB1 /DB_XREF=gi:186914 /FL=gb:M20206.1	M20206	laminin, beta 1	LAMB1	3912	NM_002291	0007155 // cell adhesion // traceable author statement /// 0007162 // negative regulation of cell adhesion // inferred from electronic annotation /// 0007411 // axon guidance // traceable author statement /// 0007566 // embryo implantation // inferred from electronic annotation /// 0016192 // vesicle-mediated transport // inferred from electronic annotation /// 0016477 // cell migration // inferred from electronic annotation /// 0021812 // neuronal-glial interaction involved in cerebral cortex radial glia guided migration // inferred from mutant phenotype /// 0022617 // extracellular matrix disassembly // traceable author statement /// 0030198 // [illegible]	0005576 // extracellular region // traceable author statement /// 0005578 // proteinaceous extracellular matrix // inferred from electronic annotation /// 0005604 // basement membrane // inferred from direct assay /// 0005604 // basement membrane // traceable author statement /// 0005605 // basal lamina // inferred from electronic annotation /// 0005606 // laminin-1 complex // inferred from [illegible]	0005178 // integrin binding // inferred from electronic annotation /// 0005198 // structural molecule activity // non-traceable author statement /// 0005201 // extracellular matrix structural constituent // inferred from direct assay /// 0005515 // protein binding // inferred from electronic annotation /// 0019899 // enzyme binding // inferred from electronic annotation /// 0043208 // glycosphingolipid binding // inferred from electronic annotation

211816_x_at	D87858	FCAR	Homo sapiens	6-Oct-14	Exemplar sequence	GenBank	gb:D87858.1 /DEF=Homo sapiens mRNA for CD89_U04, complete cds. /FEA=CDS /PROD=CD89_U04 /DB_XREF=gi:1561545 /UG=Hs.193122 Fc fragment of IgA, receptor for /FL=gb:D87858.1 gb:D87860.1	D87858	Fc fragment of IgA, receptor for	FCAR	2204	NM_002000 /// NM_133269 /// NM_133271 /// NM_133272 /// NM_133273 /// NM_133274 /// NM_133277 /// NM_133278 /// NM_133279 /// NM_133280	0006955 // immune response // traceable author statement	0005576 // extracellular region // inferred from electronic annotation /// 0005886 // plasma membrane // inferred from direct assay /// 0005887 // integral component of plasma membrane // traceable author statement /// 0016020 // membrane // inferred from electronic annotation /// 0016021 // integral component of membrane // inferred from electronic annotation	0019862 // IgA binding // inferred from electronic annotation

211831_s_at	U59495	THPO	Homo sapiens	6-Oct-14	Exemplar sequence	GenBank	gb:U59495.1 /DEF=Human truncated thrombopoietin (thTPO) mRNA, complete cds. /FEA=CDS /GEN=thTPO /PROD=truncated thrombopoietin /DB_XREF=gi:1401249 /UG=Hs.1166 thrombopoietin (myeloproliferative leukemia virus oncogene ligand, megakaryocyte growth and development factor) /FL=gb:U59495.1	U59495	thrombopoietin	THPO	7066	NM_000460 /// NM_001177597 /// NM_001177598 /// NM_001289997 /// NM_001289998 /// NM_001290003 /// NM_001290022 /// NM_001290026 /// NM_001290027 /// NM_001290028 /// NM_199228 /// NM_199356 /// XM_005247737 /// XM_006713738 /// XM_006713739	0001934 // positive regulation of protein phosphorylation // inferred from sequence or structural similarity /// 0007275 // multicellular organismal development // traceable author statement /// 0007596 // blood coagulation // traceable author statement /// 0008283 // cell proliferation // inferred from electronic annotation /// 0030099 // myeloid cell differentiation // inferred from electronic annotation /// 0030168 // platelet activation // traceable author statement /// 0038163 // thrombopoietin-mediated signaling pathway // inferred from sequence or structural similarity /// 0045654 // positive [illegible]	0005576 // extracellular region // inferred from electronic annotation /// 0005615 // extracellular space // inferred from electronic annotation	0005125 // cytokine activity // inferred from electronic annotation /// 0005179 // hormone activity // inferred from electronic annotation /// 0008083 // growth factor activity // traceable author statement

211919_s_at	AF348491	CXCR4	Homo sapiens	6-Oct-14	Exemplar sequence	GenBank	gb:AF348491.1 /DEF=Homo sapiens chemokine receptor CXCR4 mRNA, complete cds. /FEA=CDS /PROD=chemokine receptor CXCR4 /DB_XREF=gi:13549089 /FL=gb:AF348491.1	AF348491	chemokine (C-X-C motif) receptor 4	CXCR4	7852	NM_001008540 /// NM_003467	0000187 // activation of MAPK activity // traceable author statement /// 0001569 // patterning of blood vessels // inferred from electronic annotation /// 0001666 // response to hypoxia // inferred from expression pattern /// 0001667 // ameboidal cell migration // inferred from electronic annotation /// 0001764 // neuron migration // inferred from electronic annotation /// 0002407 // dendritic cell chemotaxis // traceable author statement /// 0006915 // apoptotic process // traceable author statement /// 0006935 // chemotaxis // inferred from electronic annotation /// 0006954 // inflammatory response // traceable author [illegible]	0005737 // cytoplasm // traceable author statement /// 0005764 // lysosome // inferred from direct assay /// 0005768 // endosome // inferred from electronic annotation /// 0005769 // early endosome // inferred from direct assay /// 0005770 // late endosome // inferred from direct assay /// 0005886 // plasma membrane // inferred from direct assay /// 0005886 // plasma membrane // [illegible]	0001618 // virus receptor activity // inferred from electronic annotation /// 0003779 // actin binding // inferred from direct assay /// 0004871 // signal transducer activity // inferred from electronic annotation /// 0004930 // G-protein coupled receptor activity // traceable author statement /// 0004950 // chemokine receptor activity // inferred from electronic annotation /// 0005515 // protein binding // inferred from physical interaction /// 0015026 // coreceptor activity // traceable author statement /// 0016494 // C-X-C chemokine receptor activity // non-traceable author statement /// 0019955 // cytokine binding // inferred from electronic annotation /// 0031625 // ubiquitin protein ligase binding // [illegible]

212081_x_at	AF129756	PRRC2A	Homo sapiens	6-Oct-14	Consensus sequence	GenBank	Consensus includes gb:AF129756 /DEF=Homo sapiens MSH55 gene, partial cds; and CLIC1, DDAH, G6b, G6c, G5b, G6d, G6e, G6f, BAT5, G5b, CSK2B, BAT4, G4, Apo M, BAT3, BAT2, AIF-1, 1C7, LST-1, LTB, TNF, and LTA genes, complete cds /FEA=mRNA_20 /DB_XREF=gi:4337095 /UG=Hs.25911 HLA-B associated transcript-2	AF129756	proline-rich coiled-coil 2A	PRRC2A	7916	NM_004638 /// NM_080686 /// XM_006715191 /// XM_006725507 /// XM_006725508 /// XM_006725723 /// XM_006725724 /// XM_006725833 /// XM_006725834 /// XM_006725922 /// XM_006726023 /// XM_006726024 /// XM_006726111 /// XM_006726112		0005634 // nucleus // inferred from electronic annotation /// 0005737 // cytoplasm // inferred from electronic annotation /// 0016020 // membrane // inferred from direct assay /// 0070062 // extracellular vesicular exosome // inferred from direct assay	0005515 // protein binding // inferred from physical interaction /// 0044822 // poly(A) RNA binding // inferred from direct assay

212185_x_at	NM_005953	MT2A	Homo sapiens	6-Oct-14	Consensus sequence	GenBank	Consensus includes gb:NM_005953.1 /DEF=Homo sapiens metallothionein 2A (MT2A), mRNA. /FEA=CDS /GEN=MT2A /PROD=metallothionein 2A /DB_XREF=gi:5174763 /UG=Hs.118786 metallothionein 2A /FL=gb:NM_005953.1	NM_005953	metallothionein 2A	MT2A	4502	NM_005953	0006878 // cellular copper ion homeostasis // traceable author statement /// 0019221 // cytokine-mediated signaling pathway // traceable author statement /// 0035690 // cellular response to drug // inferred from direct assay /// 0036016 // cellular response to interleukin-3 // inferred from expression pattern /// 0036018 // cellular response to erythropoietin // inferred from expression pattern /// 0045926 // negative regulation of growth // inferred from sequence or structural similarity /// 0060333 // interferon-gamma-mediated signaling pathway // traceable author statement /// 0071294 // [illegible]	0005634 // nucleus // inferred from sequence or structural similarity /// 0005737 // cytoplasm // inferred from sequence or structural similarity /// 0005829 // cytosol // traceable author statement /// 0048471 // perinuclear region of cytoplasm // inferred from sequence or structural similarity	0005515 // protein binding // inferred from physical interaction /// 0008144 // drug binding // inferred from direct assay /// 0008270 // zinc ion binding // inferred from direct assay /// 0008270 // zinc ion binding // inferred from sequence or structural similarity /// 0046872 // metal ion binding // inferred from electronic annotation

212236_x_at	Z19574	JUP	Homo sapiens	6-Oct-14	Consensus sequence	GenBank	Consensus includes gb:Z19574 /DEF=H.sapiens gene for cytokeratin 17 /FEA=mRNA /DB_XREF=gi:30378 /UG=Hs.2785 keratin 17	Z19574	junction plakoglobin /// keratin 17	JUP /// KRT17	3728 /// 3872	NM_000422 /// NM_002230 /// NM_021991 /// XM_005257313 /// XM_005257349 /// XM_006721871 /// XM_006721872 /// XM_006721873 /// XM_006721874 /// XM_006721875 /// XM_006721876 /// XM_006721877 /// XM_006721878	0000122 // negative regulation of transcription from RNA polymerase II promoter // not recorded /// 0000902 // cell morphogenesis // not recorded /// 0002009 // morphogenesis of an epithelium // inferred from electronic annotation /// 0002159 // desmosome assembly // inferred from direct assay /// 0002159 // desmosome assembly // inferred from mutant phenotype /// 0003136 // negative regulation of heart induction by canonical Wnt signaling pathway // not recorded /// 0003181 // atrioventricular valve morphogenesis // not recorded /// 0003308 // negative regulation of Wnt signaling pathway [illegible]	0005634 // nucleus // inferred from mutant phenotype /// 0005667 // transcription factor complex // not recorded /// 0005737 // cytoplasm // inferred from mutant phenotype /// 0005737 // cytoplasm // inferred from electronic annotation /// 0005829 // cytosol // inferred from sequence or structural similarity /// 0005856 // cytoskeleton // inferred from sequence or structural [illegible]	0003713 // transcription coactivator activity // inferred from direct assay /// 0005198 // structural molecule activity // not recorded /// 0005198 // structural molecule activity // non-traceable author statement /// 0005199 // structural constituent of cell wall // inferred by curator /// 0005200 // structural constituent of cytoskeleton // traceable author statement /// 0005488 // binding // inferred from electronic annotation /// 0005515 // protein binding // inferred from physical interaction /// 0019901 // protein kinase binding // not recorded /// 0019903 // protein phosphatase binding // inferred from physical interaction /// 0032395 // MHC class II receptor activity // inferred from direct assay /// 0042289 // MHC class II [illegible]

212430_at	AL109955	RBM38	Homo sapiens	6-Oct-14	Consensus sequence	GenBank	Consensus includes gb:AL109955 /DEF=Human DNA sequence from clone RP4-800J21 on chromosome 20 Contains ESTs, STSs, GSSs and CpG islands. Contains the 3 part of the RAE1 gene for a homolog to RNA export protein 1 from S.pombe and the gene for the ssDNA binding protein SEB4D.n /FEA=mRNA_1 /DB_XREF=gi:11558768 /UG=Hs.236361 [illegible]	AL109955	RNA binding motif protein 38	RBM38	55544	NM_001291780 /// NM_017495 /// NM_183425 /// XM_005260445 /// XM_005260446	0006397 // mRNA processing // inferred from electronic annotation /// 0006977 // DNA damage response, signal transduction by p53 class mediator resulting in cell cycle arrest // inferred from direct assay /// 0006978 // DNA damage response, signal transduction by p53 class mediator resulting in transcription of p21 class mediator // inferred from direct assay /// 0007049 // cell cycle // inferred from electronic annotation /// 0007050 // cell cycle arrest // inferred from direct assay /// 0008285 // negative regulation of cell proliferation // inferred from direct assay /// 0008380 // RNA splicing // inferred from electronic [illegible]	0005634 // nucleus // inferred from direct assay /// 0005737 // cytoplasm // inferred from electronic annotation /// 0005829 // cytosol // inferred from direct assay /// 0005829 // cytosol // inferred from electronic annotation	0000166 // nucleotide binding // inferred from electronic annotation /// 0003676 // nucleic acid binding // inferred from electronic annotation /// 0003723 // RNA binding // inferred from electronic annotation /// 0003729 // mRNA binding // inferred from direct assay /// 0003729 // mRNA binding // inferred from electronic annotation /// 0003730 // mRNA 3'-UTR binding // inferred from direct assay /// 0044822 // poly(A) RNA binding // inferred from direct assay

212538_at	AL576253	DOCK9	Homo sapiens	6-Oct-14	Consensus sequence	GenBank	Consensus includes gb:AL576253 /FEA=EST /DB_XREF=gi:12938214 /DB_XREF=est: AL576253 /CLONE=CS0DI073YM22 (3 prime) /UG=Hs.8021 KIAA1058 protein	AL576253	dedicator of cytokinesis 9	DOCK9	23348	NM_001130048 /// NM_001130049 /// NM_001130050 /// NM_015296 /// XM_005254034 /// XM_005254035 /// XM_005254036 /// XM_006719922 /// XM_006719923 /// XM_006719924 /// XM_006719925 /// XM_006719926 /// XM_006719927 /// XM_006719928 ///	0007264 // small GTPase mediated signal transduction // inferred from electronic annotation /// 0007596 // blood coagulation // traceable author statement /// 0032319 // regulation of Rho GTPase activity // inferred from electronic annotation /// 0032321 // positive regulation of Rho GTPase activity // inferred from electronic annotation /// 0043547 // positive regulation of GTPase activity // inferred from electronic annotation	0005829 // cytosol // traceable author statement /// 0012505 // endomembrane system // inferred from electronic annotation /// 0016020 // membrane // inferred from electronic annotation	0005085 // guanyl-nucleotide exchange factor activity // inferred from electronic annotation /// 0005089 // Rho guanyl-nucleotide exchange factor activity // inferred from electronic annotation /// 0005488 // binding // inferred from electronic annotation /// 0005515 // protein binding // inferred from physical interaction /// 0017048 // Rho GTPase binding // inferred from electronic annotation

212671_s_at	BG397856	HLA-DQA1	Homo sapiens	6-Oct-14	Consensus sequence	GenBank	Consensus includes gb:BG397856 /FEA=EST /DB_XREF=gi:13291304 /DB_XREF=est:602438950F1 /CLONE=IMAGE:4564956 /UG=Hs.198253 major histocompatibility complex, class II, DQ alpha 1	BG397856	major histocompatibility complex, class II, DQ alpha 1 /// major histocompatibility complex, class II, DQ alpha 2 /// HLA class II histocompatibility antigen, DQ alpha 1 chain-like	HLA-DQA1 /// HLA-DQA2 /// LOC100509457	3117 /// 3118 /// 100509457	NM_002122 /// NM_020056 /// XM_003120269 /// XM_003846468 /// XM_005274953 /// XM_005275108 /// XM_005275333 /// XM_005275542 /// XM_005275544 /// XM_005276080 /// XM_006715079 /// XM_006725484 /// XM_006725999	0002376 // immune system process // inferred from electronic annotation /// 0002504 // antigen processing and presentation of peptide or polysaccharide antigen via MHC class II // inferred from electronic annotation /// 0006955 // immune response // non-traceable author statement /// 0019221 // cytokine-mediated signaling pathway // traceable author statement /// 0019882 // antigen processing and presentation // inferred from electronic annotation /// 0019886 // antigen processing and presentation of exogenous peptide antigen via MHC class II // traceable author statement /// 0031295 // T cell costimulation // [illegible]	0000139 // Golgi membrane // traceable author statement /// 0005764 // lysosome // inferred from electronic annotation /// 0005765 // lysosomal membrane // traceable author statement /// 0005768 // endosome // inferred from electronic annotation /// 0005783 // endoplasmic reticulum // inferred from electronic annotation /// 0005789 // endoplasmic [illegible]	0005515 // protein binding // inferred from electronic annotation /// 0032395 // MHC class II receptor activity // non-traceable author statement /// 0032395 // MHC class II receptor activity // traceable author statement /// 0042605 // peptide antigen binding // inferred from sequence or structural similarity

212687_at	AL110164	LIMS1	Homo sapiens	6-Oct-14	Consensus sequence	GenBank	Consensus includes gb:AL110164.1 /DEF=Homo sapiens mRNA; cDNA DKFZp586I0324 (from clone DKFZp586I0324). /FEA=mRNA /DB_XREF=gi:5817069 /UG=Hs.193700 Homo sapiens mRNA; cDNA DKFZp586I0324 (from clone DKFZp586I0324)	AL110164	LIM and senescent cell antigen-like domains 1 /// LIM and senescent cell antigen-like domains 3 /// LIM and senescent cell antigen-like domains 3-like	LIMS1 /// LIMS3 /// LIMS3L	3987 /// 96626 /// 100288695	NM_001193482 /// NM_001193483 /// NM_001193484 /// NM_001193485 /// NM_001193488 /// NM_001205288 /// NM_004987 /// NM_033514 /// NR_027467 /// NR_038099 /// XM_005263563 /// XM_005263946 /// XM_005263947 /// XM_005263948 /// XM_005263949 /// XM_005263950 ///	0007569 // cell aging // traceable author statement /// 0034329 // cell junction assembly // traceable author statement /// 0045892 // negative regulation of transcription, DNA-templated // inferred from direct assay /// 0071560 // cellular response to transforming growth factor beta stimulus // inferred from expression pattern	0005737 // cytoplasm // inferred from electronic annotation /// 0005829 // cytosol // traceable author statement /// 0005886 // plasma membrane // inferred from electronic annotation /// 0005925 // focal adhesion // inferred from direct assay /// 0016020 // membrane // inferred from electronic annotation /// 0030054 // cell junction // inferred from electronic annotation /// 0048471 //	0005515 // protein binding // inferred from physical interaction /// 0008270 // zinc ion binding // inferred from direct assay /// 0008270 // zinc ion binding // inferred from electronic annotation /// 0046872 // metal ion binding // inferred from electronic annotation
212732_at	AI950273	MEG3	Homo sapiens	6-Oct-14	Consensus sequence	GenBank	Consensus includes gb:AI950273 /FEA=EST /DB_XREF=gi:5742583 /DB_XREF=est:wq37h05.x1 /CLONE=IMAGE:2473497 /UG=Hs.112844 maternally expressed 3	AI950273	maternally expressed 3 (non-protein coding)	MEG3	55384	NR_002766 /// NR_003530 /// NR_003531 /// NR_033358 /// NR_033359 /// NR_033360 /// NR_046464 /// NR_046465 /// NR_046466 /// NR_046467 /// NR_046468 /// NR_046469 /// NR_046470 /// NR_046471 /// NR_046472 /// NR_046473			

212768_s_at	AL390736	OLFM4	Homo sapiens	6-Oct-14	Consensus sequence	GenBank	Consensus includes gb:AL390736 /DEF=Human DNA sequence from clone RP11-209J19 on chromosome 13 Contains ESTs, STSs and GSSs. Contains the gene for the GW112 protein with two isoforms (GW112 and KIAA4294) /FEA=mRNA /DB_XREF=gi:11182238 /UG=Hs.273321 differentially expressed in hematopoietic lineages	AL390736	olfactomedin 4	OLFM4	10562	NM_006418	0007155 // cell adhesion // inferred from electronic annotation /// 0008152 // metabolic process // inferred from electronic annotation /// 0010939 // regulation of necrotic cell death // inferred from mutant phenotype /// 0042981 // regulation of apoptotic process // inferred from mutant phenotype /// 0043124 // negative regulation of I-kappaB kinase/NF-kappaB signaling // inferred from electronic annotation /// 0050764 // regulation of phagocytosis // inferred from mutant phenotype /// 0050777 // negative regulation of immune response // inferred from electronic annotation /// 0051260 // protein homooligomerization // inferred from mutant [illegible]	0005576 // extracellular region // inferred from electronic annotation /// 0005615 // extracellular space // inferred from direct assay /// 0005615 // extracellular space // inferred from mutant phenotype /// 0005739 // mitochondrion // inferred from electronic annotation /// 0005886 // plasma membrane // inferred from direct assay /// 0030141 // secretory granule // inferred from direct assay /// 0042581 // specific [illegible]	0003824 // catalytic activity // inferred from electronic annotation /// 0005515 // protein binding // inferred from electronic annotation /// 0042803 // protein homodimerization activity // inferred from mutant phenotype /// 0045296 // cadherin binding // inferred from mutant phenotype

212914_at	AV648364	CBX7	Homo sapiens	6-Oct-14	Consensus sequence	GenBank	Consensus includes gb:AV648364 /FEA=EST /DB_XREF=gi:9869378 /DB_XREF=est: AV648364 /CLONE=GLCBIF04 /UG=Hs.152151 plakophilin 4	AV648364	chromobox homolog 7	CBX7	23492	NM_175709 /// XM_005261413 /// XM_006724174 /// XM_006724175 /// XM_006724176 /// XM_006724177 /// XM_006724178	0000122 // negative regulation of transcription from RNA polymerase II promoter // inferred from mutant phenotype /// 0006351 // transcription, DNA-templated // inferred from electronic annotation /// 0006355 // regulation of transcription, DNA-templated // inferred from electronic annotation /// 0016568 // chromatin modification // inferred from electronic annotation	0000790 // nuclear chromatin // inferred from direct assay /// 0000792 // heterochromatin // inferred from electronic annotation /// 0005634 // nucleus // inferred from direct assay /// 0005730 // nucleolus // inferred from direct assay /// 0005737 // cytoplasm // inferred from direct assay /// 0031519 // PcG protein complex // inferred from direct assay /// 0035102 // PRC1 complex // inferred from	0003682 // chromatin binding // inferred from electronic annotation /// 0003727 // single-stranded RNA binding // inferred from electronic annotation /// 0005515 // protein binding // inferred from physical interaction /// 0035064 // methylated histone binding // inferred from electronic annotation

212935_at	AB002360	MCF2L	Homo sapiens	6-Oct-14	Consensus sequence	GenBank	Consensus includes gb:AB002360.1 /DEF=Human mRNA for KIAA0362 gene, partial cds. /FEA=mRNA /GEN=KIAA0362 /DB_XREF=gi:2224664 /UG=Hs.25515 MCF.2 cell line derived transforming sequence-like	AB002360	MCF.2 cell line derived transforming sequence-like	MCF2L	23263	NM_001112732 /// NM_024979 /// XM_005268307 /// XM_005268308 /// XM_005268309 /// XM_005268310 /// XM_005268312 /// XM_005268313 /// XM_005268314 /// XM_005268315 /// XM_005268316 /// XM_006719966 /// XM_006719967 /// XM_006719968 /// XR_429282	0007264 // small GTPase mediated signal transduction // traceable author statement /// 0007266 // Rho protein signal transduction // inferred from electronic annotation /// 0032319 // regulation of Rho GTPase activity // inferred from electronic annotation /// 0032321 // positive regulation of Rho GTPase activity // inferred from electronic annotation /// 0035023 // regulation of Rho protein signal transduction // inferred from electronic annotation /// 0035556 // intracellular signal transduction // inferred from electronic annotation /// 0043065 // positive regulation of apoptotic process // traceable author [illegible]	0005615 // extracellular space // inferred from direct assay /// 0005737 // cytoplasm // inferred from electronic annotation /// 0005829 // cytosol // traceable author statement /// 0005886 // plasma membrane // inferred from electronic annotation /// 0016020 // membrane // inferred from electronic annotation /// 0030027 // lamellipodium // inferred from electronic annotation	0005085 // guanyl-nucleotide exchange factor activity // inferred from electronic annotation /// 0005089 // Rho guanyl-nucleotide exchange factor activity // inferred from electronic annotation /// 0005515 // protein binding // inferred from electronic annotation /// 0005543 // phospholipid binding // inferred from electronic annotation /// 0005545 // 1-phosphatidylinositol binding // inferred from electronic annotation

212949_at	D38553	NCAPH	Homo sapiens	6-Oct-14	Consensus sequence	GenBank	Consensus includes gb:D38553.1 /DEF=Homo sapiens HCAP-H mRNA, partial cds. /FEA=mRNA /GEN=HCAP-H /DB_XREF=gi:559714 /UG=Hs.1192 KIAA0074 protein	D38553	non-SMC condensin I complex, subunit H	NCAPH	23397	NM_001281710 /// NM_001281711 /// NM_001281712 /// NM_015341 /// XM_005263907 /// XM_005263908 /// XM_006712388	0000278 // mitotic cell cycle // traceable author statement /// 0007049 // cell cycle // inferred from electronic annotation /// 0007067 // mitotic nuclear division // inferred from electronic annotation /// 0007076 // mitotic chromosome condensation // inferred from direct assay /// 0030261 // chromosome condensation // inferred from electronic annotation /// 0051301 // cell division // inferred from electronic annotation	0000796 // condensin complex // inferred from direct assay /// 0005634 // nucleus // inferred from electronic annotation /// 0005694 // chromosome // inferred from electronic annotation /// 0005737 // cytoplasm // inferred from direct assay /// 0005829 // cytosol // traceable author statement /// 0015630 // microtubule cytoskeleton // inferred from direct assay /// 0016020 // membrane // [illegible]	0005515 // protein binding // inferred from physical interaction

213528_at	AL035369	METTL18	Homo sapiens	6-Oct-14	Consensus sequence	GenBank	Consensus includes gb:AL035369.1 /DEF=H.sapiens novel gene from PAC 117P20, chromosome 1. /FEA=mRNA /PROD=hypothetical protein /DB_XREF=gi:4239681 /UG=Hs.33922 H.sapiens novel gene from PAC 117P20, chromosome 1	AL035369	methyltransferase like 18	METTL18	92342	NM_033418 /// XM_005245606 /// XM_006711627	0006479 // protein methylation // inferred from electronic annotation /// 0032259 // methylation // inferred from electronic annotation	0005737 // cytoplasm // inferred from electronic annotation	0008168 // methyltransferase activity // inferred from electronic annotation /// 0008276 // protein methyltransferase activity // inferred from electronic annotation /// 0016740 // transferase activity // inferred from electronic annotation
213629_x_at	BF246115	MT1F	Homo sapiens	6-Oct-14	Consensus sequence	GenBank	Consensus includes gb:BF246115 /FEA=EST /DB_XREF=gi:11160133 /DB_XREF=est:601854068F1 /CLONE=IMAGE:4073921 /UG=Hs.8765 RNA helicase-related protein	BF246115	metallothionein 1F	MT1F	4494	NM_005949	0045926 // negative regulation of growth // inferred from sequence or structural similarity /// 0071276 // cellular response to cadmium ion // inferred from expression pattern /// 0071294 // cellular response to zinc ion // inferred from sequence or structural similarity	0005634 // nucleus // inferred from direct assay /// 0005737 // cytoplasm // inferred from direct assay /// 0048471 // perinuclear region of cytoplasm // inferred from sequence or structural similarity	0005507 // copper ion binding // non-traceable author statement /// 0008270 // zinc ion binding // inferred from sequence or structural similarity /// 0046870 // cadmium ion binding // non-traceable author statement /// 0046872 // metal ion binding // inferred from electronic annotation

213638_at	AW054711	PHACTR1	Homo sapiens	6-Oct-14	Consensus sequence	GenBank	Consensus includes gb:AW054711 /FEA=EST /DB_XREF=gi:5920414 /DB_XREF=est: wz96g08.x1 /CLONE=IMAGE:2566718 /UG=Hs.121591 Human DNA sequence from PAC 257A7 on chromosome 6p24. Contains two unknown genes and ESTs, STSs and a GSS	AW054711	phosphatase and actin regulator 1	PHACTR1	221692	NM_001242648 /// NM_030948 /// XM_005248933 /// XM_005248934 /// XM_005248935 /// XM_005248936 /// XM_005248937 /// XM_006715021	0031032 // actomyosin structure organization // inferred from mutant phenotype /// 0031532 // actin cytoskeleton reorganization // inferred from sequence or structural similarity /// 0043086 // negative regulation of catalytic activity // inferred from electronic annotation /// 0043149 // stress fiber assembly // inferred from mutant phenotype /// 0048870 // cell motility // inferred from mutant phenotype	0005634 // nucleus // inferred from sequence or structural similarity /// 0005737 // cytoplasm // inferred from electronic annotation /// 0005737 // cytoplasm // inferred from direct assay /// 0005829 // cytosol // inferred from sequence or structural similarity /// 0030054 // cell junction // inferred from electronic annotation /// 0045202 // synapse // inferred from electronic annotation	0003779 // actin binding // inferred from sequence or structural similarity /// 0004864 // protein phosphatase inhibitor activity // inferred from electronic annotation

213650_at	AW006438	GOLGA8A	Homo sapiens	6-Oct-14	Consensus sequence	GenBank	Consensus includes gb:AW006438 /FEA=EST /DB_XREF=gi:5855216 /DB_XREF=est:wr16f06.x1 /CLONE=IMAGE:2481731 /UG=Hs.182982 golgin-67	AW006438	golgin A8 family, member A /// golgin A8 family, member B /// uncharacterized LOC101930583	GOLGA8A /// GOLGA8B /// LOC101930583	23015 /// 440270 /// 101930583	NM_001023567 /// NM_181077 /// NR_027409 /// NR_027410 /// XM_005254389 /// XM_005254391 /// XM_006720440 /// XM_006720530 /// XR_248092 /// XR_248093 /// XR_248094 /// XR_248096 /// XR_424599 /// XR_424600 /// XR_424601 /// XR_424602		0005794 // Golgi apparatus // inferred from electronic annotation /// 0016020 // membrane // inferred from electronic annotation /// 0032580 // Golgi cisterna membrane // inferred from electronic annotation	

213774_s_at	AW614578	PPP1R2	Homo sapiens	6-Oct-14	Consensus sequence	GenBank	Consensus includes gb:AW614578 /FEA=EST /DB_XREF=gi:7319764 /DB_XREF=est:hh01f08.x1 /CLONE=IMAGE:2953863 /UG=Hs.267819 protein phosphatase 1, regulatory (inhibitor) subunit 2	AW614578	protein phosphatase 1, regulatory (inhibitor) subunit 2	PPP1R2	5504	NM_001291504 /// NM_001291505 /// NM_006241 /// XM_006713682 /// XM_006713683	0005977 // glycogen metabolic process // inferred from electronic annotation /// 0006091 // generation of precursor metabolites and energy // traceable author statement /// 0009966 // regulation of signal transduction // inferred from electronic annotation /// 0043086 // negative regulation of catalytic activity // inferred from electronic annotation /// 0043086 // negative regulation of catalytic activity // traceable author statement /// 0043666 // regulation of phosphoprotein phosphatase activity // inferred from electronic annotation		0004865 // protein serine/threonine phosphatase inhibitor activity // traceable author statement /// 0005515 // protein binding // inferred from physical interaction

213831_at	X00452	HLA-DQA1	Homo sapiens	6-Oct-14	Consensus sequence	GenBank	Consensus includes gb:X00452.1 /DEF=Human mRNA for DC classII histocompatibility antigen alpha-chain. /FEA=mRNA /PROD=DC classII histocompatibility antigenalpha-chain /DB_XREF=gi:32265 /UG=Hs.198253 major histocompatibility complex, class II, DQ alpha 1	X00452	major histocompatibility complex, class II, DQ alpha 1 /// HLA class II histocompatibility antigen, DQ alpha 1 chain-like	HLA-DQA1 /// LOC100509457	3117 /// 100509457	NM_002122 /// XM_003120269 /// XM_003846468 /// XM_005274953 /// XM_005275108 /// XM_005275333 /// XM_005275542 /// XM_005275544 /// XM_005276080 /// XM_006715079 /// XM_006725484 /// XM_006725999	0002376 // immune system process // inferred from electronic annotation /// 0002504 // antigen processing and presentation of peptide or polysaccharide antigen via MHC class II // inferred from electronic annotation /// 0006955 // immune response // non-traceable author statement /// 0019221 // cytokine-mediated signaling pathway // traceable author statement /// 0019882 // antigen processing and presentation // inferred from electronic annotation /// 0019886 // antigen processing and presentation of exogenous peptide antigen via MHC class II // traceable author statement /// 0031295 // T cell costimulation //	0000139 // Golgi membrane // traceable author statement /// 0005764 // lysosome // inferred from electronic annotation /// 0005765 // lysosomal membrane // traceable author statement /// 0005768 // endosome // inferred from electronic annotation /// 0005783 // endoplasmic reticulum // inferred from electronic annotation /// 0005789 // endoplasmic	0005515 // protein binding // inferred from electronic annotation /// 0032395 // MHC class II receptor activity // non-traceable author statement /// 0032395 // MHC class II receptor activity // traceable author statement /// 0042605 // peptide antigen binding // inferred from sequence or structural similarity
213991_s_at	BF940710	HS3ST1	Homo sapiens	6-Oct-14	Consensus sequence	GenBank	Consensus includes gb:BF940710 /FEA=EST /DB_XREF=gi:12358030 /DB_XREF=est:nae24b08.x1 /CLONE=IMAGE:3435999 /UG=Hs.40968 heparan sulfate (glucosamine) 3-O-sulfotransferase 1	BF940710	heparan sulfate (glucosamine) 3-O-sulfotransferase 1	HS3ST1	9957	NM_005114 /// XM_005248221	0005975 // carbohydrate metabolic process // traceable author statement /// 0006024 // glycosaminoglycan biosynthetic process // traceable author statement /// 0030203 // glycosaminoglycan metabolic process // traceable author statement /// 0044281 // small molecule metabolic process // traceable author statement	0005796 // Golgi lumen // traceable author statement /// 0016021 // integral component of membrane // traceable author statement	0008146 // sulfotransferase activity // traceable author statement /// 0008467 // [heparan sulfate]-glucosamine 3-sulfotransferase 1 activity // inferred from electronic annotation

213998_s_at	AW188131	DDX17	Homo sapiens	6-Oct-14	Consensus sequence	GenBank	Consensus includes gb:AW188131 /FEA=EST /DB_XREF=gi:6462567 /DB_XREF=est:xj92f11.x1 /CLONE=IMAGE:2664717 /UG=Hs.6179 DEADH (Asp-Glu-Ala-AspHis) box polypeptide 17 (72kD)	AW188131	DEAD (Asp-Glu-Ala-Asp) box helicase 17	DDX17	10521	NM_001098504 /// NM_001098505 /// NM_006386 /// NM_030881	0006200 // ATP catabolic process // inferred from electronic annotation /// 0006200 // ATP catabolic process // traceable author statement /// 0006351 // transcription, DNA-templated // inferred from electronic annotation /// 0006355 // regulation of transcription, DNA-templated // inferred from electronic annotation /// 0006396 // RNA processing // traceable author statement /// 0008152 // metabolic process // inferred from electronic annotation /// 0009791 // post-embryonic development // inferred from electronic annotation /// 0033148 // positive regulation of intracellular estrogen [illegible]	0005634 // nucleus // inferred from direct assay /// 0005730 // nucleolus // inferred from electronic annotation /// 0016020 // membrane // inferred from direct assay	0000166 // nucleotide binding // inferred from electronic annotation /// 0003676 // nucleic acid binding // inferred from electronic annotation /// 0003713 // transcription coactivator activity // inferred from direct assay /// 0003723 // RNA binding // traceable author statement /// 0003724 // RNA helicase activity // traceable author statement /// 0004386 // helicase activity // inferred from electronic annotation /// 0005515 // protein binding // inferred from physical interaction /// 0005524 // ATP binding // inferred from electronic annotation /// 0008026 // ATP-dependent helicase activity // inferred from electronic annotation /// 0008186 // RNA-dependent ATPase activity // traceable [illegible]

214012_at	BE551138	ERAP1	Homo sapiens	6-Oct-14	Consensus sequence	GenBank	Consensus includes gb:BE551138 /FEA=EST /DB_XREF=gi:9792830 /DB_XREF=est:7b55a07.x1 /CLONE=IMAGE:3232116 /UG=Hs.247043 type 1 tumor necrosis factor receptor shedding aminopeptidase regulator	BE551138	endoplasmic reticulum aminopeptidase 1	ERAP1	51752	NM_001040458 /// NM_001198541 /// NM_016442 /// XM_005272015 /// XM_005272016	0001525 // angiogenesis // traceable author statement /// 0002376 // immune system process // inferred from electronic annotation /// 0002474 // antigen processing and presentation of peptide antigen via MHC class I // traceable author statement /// 0006508 // proteolysis // inferred from electronic annotation /// 0006509 // membrane protein ectodomain proteolysis // inferred from direct assay /// 0008217 // regulation of blood pressure // non-traceable author statement /// 0008217 // regulation of blood pressure // traceable author statement /// 0009617 // response to bacterium // inferred from expression pattern ///	0005576 // extracellular region // inferred from direct assay /// 0005615 // extracellular space // inferred from direct assay /// 0005737 // cytoplasm // inferred from electronic annotation /// 0005783 // endoplasmic reticulum // non-traceable author statement /// 0005788 // endoplasmic reticulum lumen // traceable author statement /// 0005789 // endoplasmic reticulum membrane //	0004177 // aminopeptidase activity // inferred from direct assay /// 0005138 // interleukin-6 receptor binding // inferred from physical interaction /// 0005151 // interleukin-1, Type II receptor binding // traceable author statement /// 0005515 // protein binding // inferred from physical interaction /// 0008233 // peptidase activity // inferred from electronic annotation /// 0008235 // metalloexopeptidase activity // inferred from direct assay /// 0008237 // metallopeptidase activity // inferred from electronic annotation /// 0008270 // zinc ion binding // non-traceable author statement /// 0016787 // hydrolase activity // inferred from electronic annotation /// 0046872 // metal ion binding // inferred from electronic

214034_at	AB011097	ERAP1	Homo sapiens	6-Oct-14	Consensus sequence	GenBank	Consensus includes gb:AB011097.1 /DEF=Homo sapiens mRNA for KIAA0525 protein, partial cds. /FEA=mRNA /GEN=KIAA0525 /PROD=KIAA0525 protein /DB_XREF=gi:3043573 /UG=Hs.247043 type 1 tumor necrosis factor receptor shedding aminopeptidase regulator	AB011097	endoplasmic reticulum aminopeptidase 1	ERAP1	51752	NM_001040458 /// NM_001198541 /// NM_016442 /// XM_005272015 /// XM_005272016	0001525 // angiogenesis // traceable author statement /// 0002376 // immune system process // inferred from electronic annotation /// 0002474 // antigen processing and presentation of peptide antigen via MHC class I // traceable author statement /// 0006508 // proteolysis // inferred from electronic annotation /// 0006509 // membrane protein ectodomain proteolysis // inferred from direct assay /// 0008217 // regulation of blood pressure // non-traceable author statement /// 0008217 // regulation of blood pressure // traceable author statement /// 0009617 // response to bacterium // inferred from expression pattern ///	0005576 // extracellular region // inferred from direct assay /// 0005615 // extracellular space // inferred from direct assay /// 0005737 // cytoplasm // inferred from electronic annotation /// 0005783 // endoplasmic reticulum // non-traceable author statement /// 0005788 // endoplasmic reticulum lumen // traceable author statement /// 0005789 // endoplasmic reticulum membrane //	0004177 // aminopeptidase activity // inferred from direct assay /// 0005138 // interleukin-6 receptor binding // inferred from physical interaction /// 0005151 // interleukin-1, Type II receptor binding // traceable author statement /// 0005515 // protein binding // inferred from physical interaction /// 0008233 // peptidase activity // inferred from electronic annotation /// 0008235 // metalloexopeptidase activity // inferred from direct assay /// 0008237 // metallopeptidase activity // inferred from electronic annotation /// 0008270 // zinc ion binding // non-traceable author statement /// 0016787 // hydrolase activity // inferred from electronic annotation /// 0046872 // metal ion binding // inferred from electronic

214047_s_at	AI913365	MBD4	Homo sapiens	6-Oct-14	Consensus sequence	GenBank	Consensus includes gb:AI913365 /FEA=EST /DB_XREF=gi:5633220 /DB_XREF=est:wa11d06.x1 /CLONE=IMAGE:2297771 /UG=Hs.35947 methyl-CpG binding domain protein 4	AI913365	methyl-CpG binding domain protein 4	MBD4	8930	NM_001276270 /// NM_001276271 /// NM_001276272 /// NM_001276273 /// NM_003925	0000737 // DNA catabolic process, endonucleolytic // traceable author statement /// 0006281 // DNA repair // traceable author statement /// 0006284 // base-excision repair // traceable author statement /// 0006285 // base-excision repair, AP site formation // traceable author statement /// 0006974 // cellular response to DNA damage stimulus // inferred from electronic annotation /// 0007095 // mitotic G2 DNA damage checkpoint // inferred from electronic annotation /// 0008630 // intrinsic apoptotic signaling pathway in response to DNA damage // inferred from electronic annotation /// 0009314 // response to radiation // inferred from electronic [illegible]	0000785 // chromatin // inferred from electronic annotation /// 0005634 // nucleus // inferred from direct assay /// 0005654 // nucleoplasm // traceable author statement /// 0005730 // nucleolus // inferred from direct assay /// 0005737 // cytoplasm // inferred from electronic annotation	0003677 // DNA binding // inferred from electronic annotation /// 0003696 // satellite DNA binding // traceable author statement /// 0003824 // catalytic activity // inferred from electronic annotation /// 0004520 // endodeoxyribonuclease activity // traceable author statement /// 0005515 // protein binding // inferred from physical interaction /// 0008263 // pyrimidine-specific mismatch base pair DNA N-glycosylase activity // inferred from direct assay /// 0016787 // hydrolase activity // inferred from electronic annotation

214048_at	AI913365	MBD4	Homo sapiens	6-Oct-14	Consensus sequence	GenBank	Consensus includes gb:AI913365 /FEA=EST /DB_XREF=gi:5633220 /DB_XREF=est:wa11d06.x1 /CLONE=IMAGE:2297771 /UG=Hs.35947 methyl-CpG binding domain protein 4	AI913365	methyl-CpG binding domain protein 4	MBD4	8930	NM_001276270 /// NM_001276271 /// NM_001276272 /// NM_001276273 /// NM_003925	0000737 // DNA catabolic process, endonucleolytic // traceable author statement /// 0006281 // DNA repair // traceable author statement /// 0006284 // base-excision repair // traceable author statement /// 0006285 // base-excision repair, AP site formation // traceable author statement /// 0006974 // cellular response to DNA damage stimulus // inferred from electronic annotation /// 0007095 // mitotic G2 DNA damage checkpoint // inferred from electronic annotation /// 0008630 // intrinsic apoptotic signaling pathway in response to DNA damage // inferred from electronic annotation /// 0009314 // response to radiation // inferred from electronic [illegible]	0000785 // chromatin // inferred from electronic annotation /// 0005634 // nucleus // inferred from direct assay /// 0005654 // nucleoplasm // traceable author statement /// 0005730 // nucleolus // inferred from direct assay /// 0005737 // cytoplasm // inferred from electronic annotation	0003677 // DNA binding // inferred from electronic annotation /// 0003696 // satellite DNA binding // traceable author statement /// 0003824 // catalytic activity // inferred from electronic annotation /// 0004520 // endodeoxyribonuclease activity // traceable author statement /// 0005515 // protein binding // inferred from physical interaction /// 0008263 // pyrimidine-specific mismatch base pair DNA N-glycosylase activity // inferred from direct assay /// 0016787 // hydrolase activity // inferred from electronic annotation

214201_x_at	AA742237	PRRC2A	Homo sapiens	6-Oct-14	Consensus sequence	GenBank	Consensus includes gb:AA742237 /FEA=EST /DB_XREF=gi:2784237 /DB_XREF=est: ny80a09.s1 /CLONE=IMAGE:1284568 /UG=Hs.25911 HLA-B associated transcript-2	AA742237	proline-rich coiled-coil 2A	PRRC2A	7916	NM_004638 /// NM_080686 /// XM_006715191 /// XM_006725507 /// XM_006725508 /// XM_006725723 /// XM_006725724 /// XM_006725833 /// XM_006725834 /// XM_006725922 /// XM_006726023 /// XM_006726024 /// XM_006726111 /// XM_006726112		0005634 // nucleus // inferred from electronic annotation /// 0005737 // cytoplasm // inferred from electronic annotation /// 0016020 // membrane // inferred from direct assay /// 0070062 // extracellular vesicular exosome // inferred from direct assay	0005515 // protein binding // inferred from physical interaction /// 0044822 // poly(A) RNA binding // inferred from direct assay
214273_x_at	AV704353	NPRL3	Homo sapiens	6-Oct-14	Consensus sequence	GenBank	Consensus includes gb:AV704353 /FEA=EST /DB_XREF=gi:10721671 /DB_XREF=est: AV704353 /CLONE=ADBCNG09 /UG=Hs.19699 Conserved gene telomeric to alpha globin cluster	AV704353	nitrogen permease regulator-like 3 (S. cerevisiae)	NPRL3	8131	NM_001039476 /// NM_001077350 /// NM_001243247 /// NM_001243248 /// NM_001243249 /// NM_012075	0003281 // ventricular septum development // inferred from electronic annotation /// 0035909 // aorta morphogenesis // inferred from electronic annotation /// 0043547 // positive regulation of GTPase activity // inferred from electronic annotation /// 0048738 // cardiac muscle tissue development // inferred from electronic annotation /// 0060021 // palate development // inferred from electronic annotation		0005096 // GTPase activator activity // inferred from electronic annotation

214465_at	NM_000608	ORM1	Homo sapiens	6-Oct-14	Consensus sequence	GenBank	Consensus includes gb:NM_000608.1 /DEF=Homo sapiens orosomucoid 2 (ORM2), mRNA. /FEA=CDS /GEN=ORM2 /PROD=orosomucoid 2 /DB_XREF=gi:4505528 /UG=Hs.278388 orosomucoid 2 /FL=gb:NM_000608.1	NM_000608	orosomucoid 1 /// orosomucoid 2	ORM1 /// ORM2	5004 /// 5005	NM_000607 /// NM_000608	0002682 // regulation of immune system process // inferred from electronic annotation /// 0006810 // transport // inferred from electronic annotation /// 0006953 // acute-phase response // inferred from electronic annotation /// 0006954 // inflammatory response // traceable author statement	0005576 // extracellular region // non-traceable author statement /// 0005615 // extracellular space // inferred from direct assay /// 0070062 // extracellular vesicular exosome // inferred from direct assay /// 0072562 // blood microparticle // inferred from direct assay	0005515 // protein binding // inferred from physical interaction

214565_s_at	NM_012390	SMR3A	Homo sapiens	6-Oct-14	Consensus sequence	GenBank	Consensus includes gb:NM_012390.1 /DEF=Homo sapiens protein homologous to salivary proline-rich protein P-B (PBI), mRNA. /FEA=CDS /GEN=PBI /PROD=protein homologous to salivary proline-richprotein P-B /DB_XREF=gi:6912577 /UG=Hs.166099 protein homologous to salivary proline-rich protein P-B /FL=gb:NM_012390.1	NM_012390	submaxillary gland androgen regulated protein 3A /// submaxillary gland androgen regulated protein 3B	SMR3A /// SMR3B	10879 /// 26952	NM_006685 /// NM_012390		0005576 // extracellular region // inferred from electronic annotation /// 0005615 // extracellular space // traceable author statement	

214566_at	NM_012390	SMR3A	Homo sapiens	6-Oct-14	Consensus sequence	GenBank	Consensus includes gb:NM_012390.1 /DEF=Homo sapiens protein homologous to salivary proline-rich protein P-B (PBI), mRNA. /FEA=CDS /GEN=PBI /PROD=protein homologous to salivary proline-richprotein P-B /DB_XREF=gi:6912577 /UG=Hs.166099 protein homologous to salivary proline-rich protein P-B /FL=gb:NM_012390.1	NM_012390	submaxillary gland androgen regulated protein 3A	SMR3A	26952	NM_012390		0005576 // extracellular region // inferred from electronic annotation	
214715_x_at	AK024789	ZNF160	Homo sapiens	6-Oct-14	Consensus sequence	GenBank	Consensus includes gb:AK024789.1 /DEF=Homo sapiens cDNA: FLJ21136 fis, clone CAS07469. /FEA=mRNA /DB_XREF=gi:10437175 /UG=Hs.206882 Homo sapiens mRNA for FLJ00032 protein, partial cds	AK024789	zinc finger protein 160	ZNF160	90338	NM_001102603 /// NM_033288 /// NM_198893 /// XM_005259378 /// XM_005259379 /// XM_005259380 /// XM_006723461 /// XM_006723462	0006351 // transcription, DNA-templated // inferred from electronic annotation /// 0006355 // regulation of transcription, DNA-templated // inferred from electronic annotation /// 0030097 // hemopoiesis // non-traceable author statement	0005622 // intracellular // inferred from electronic annotation /// 0005634 // nucleus // inferred from electronic annotation	0003676 // nucleic acid binding // inferred from electronic annotation /// 0003677 // DNA binding // inferred from electronic annotation /// 0046872 // metal ion binding // inferred from electronic annotation

214764_at	AW029169	RRP15	Homo sapiens	6-Oct-14	Consensus sequence	GenBank	Consensus includes gb:AW029169 /FEA=EST /DB_XREF=gi:5887925 /DB_XREF=est: wx06g01.x1 /CLONE=IMAGE:2542896 /UG=Hs.158241 KIAA0507 protein	AW029169	ribosomal RNA processing 15 homolog (S. cerevisiae)	RRP15	51018	NM_016052		0005634 // nucleus // inferred from direct assay /// 0005730 // nucleolus // inferred from direct assay /// 0005739 // mitochondrion // inferred from direct assay	
214865_at	AC004490	DOT1L	Homo sapiens	6-Oct-14	Consensus sequence	GenBank	Consensus includes gb:AC004490 /DEF=Homo sapiens chromosome 19, cosmid R29381 /FEA=CDS /DB_XREF=gi:2995576 /UG=Hs.156015 Homo sapiens chromosome 19, cosmid R29381	AC004490	DOT1-like histone H3K79 methyltransferase	DOT1L	84444	NM_032482 /// XM_005259659 /// XM_005259660 /// XM_006722923	0016568 // chromatin modification // inferred from electronic annotation /// 0016571 // histone methylation // inferred from electronic annotation /// 0032259 // methylation // inferred from electronic annotation /// 0034968 // histone lysine methylation // inferred from direct assay /// 0034968 // histone lysine methylation // inferred from electronic annotation /// 0046425 // regulation of JAK-STAT cascade // inferred from direct assay /// 2000677 // regulation of transcription regulatory region DNA binding // inferred from mutant phenotype	0005634 // nucleus // inferred from electronic annotation	0003677 // DNA binding // inferred from electronic annotation /// 0005515 // protein binding // inferred from physical interaction /// 0008134 // transcription factor binding // inferred from physical interaction /// 0008168 // methyltransferase activity // inferred from electronic annotation /// 0016740 // transferase activity // inferred from electronic annotation /// 0018024 // histone-lysine N-methyltransferase activity // inferred from direct assay /// 0042054 // histone methyltransferase activity // inferred from electronic annotation

214956_at	AF090101	AAK1	Homo sapiens	6-Oct-14	Consensus sequence	GenBank	Consensus includes gb:AF090101.1 /DEF=Homo sapiens clone IMAGE 30714. /FEA=mRNA /DB_XREF=gi:4063636 /UG=Hs.177837 Homo sapiens clone IMAGE 30714	AF090101	AP2 associated kinase 1	AAK1	22848	NM_014911	0006468 // protein phosphorylation // inferred from direct assay /// 0006897 // endocytosis // inferred from electronic annotation /// 0016310 // phosphorylation // inferred from electronic annotation /// 0032880 // regulation of protein localization // inferred from direct assay /// 0045747 // positive regulation of Notch signaling pathway // inferred from direct assay /// 0046777 // protein autophosphorylation // inferred from sequence or structural similarity /// 0050821 // protein stabilization // inferred from direct assay /// 2000369 // regulation of clathrin-mediated endocytosis // inferred [illegible]	0005886 // plasma membrane // inferred from electronic annotation /// 0005905 // coated pit // inferred from electronic annotation /// 0016020 // membrane // inferred from electronic annotation /// 0019897 // extrinsic component of plasma membrane // inferred from sequence or structural similarity /// 0030136 // clathrin-coated vesicle // inferred from sequence or structural [illegible]	0000166 // nucleotide binding // inferred from electronic annotation /// 0004672 // protein kinase activity // inferred from electronic annotation /// 0004674 // protein serine/threonine kinase activity // inferred from sequence or structural similarity /// 0004713 // protein tyrosine kinase activity // inferred from electronic annotation /// 0005112 // Notch binding // inferred from direct assay /// 0005515 // protein binding // inferred from physical interaction /// 0005524 // ATP binding // inferred from electronic annotation /// 0016301 // kinase activity // inferred from electronic annotation /// 0016740 // transferase activity // inferred from electronic annotation /// 0016772 // transferase [illegible]

214998_at	AF090100	AAK1	Homo sapiens	6-Oct-14	Consensus sequence	GenBank	Consensus includes gb:AF090100.1 /DEF=Homo sapiens clone IMAGE 23915. /FEA=mRNA /DB_XREF=gi:4063635 /UG=Hs.170241 Homo sapiens clone IMAGE 23915	AF090100	AP2 associated kinase 1	AAK1	22848	NM_014911	0006468 // protein phosphorylation // inferred from direct assay /// 0006897 // endocytosis // inferred from electronic annotation /// 0016310 // phosphorylation // inferred from electronic annotation /// 0032880 // regulation of protein localization // inferred from direct assay /// 0045747 // positive regulation of Notch signaling pathway // inferred from direct assay /// 0046777 // protein autophosphorylation // inferred from sequence or structural similarity /// 0050821 // protein stabilization // inferred from direct assay /// 2000369 // regulation of clathrin-mediated endocytosis // inferred	0005886 // plasma membrane // inferred from electronic annotation /// 0005905 // coated pit // inferred from electronic annotation /// 0016020 // membrane // inferred from electronic annotation /// 0019897 // extrinsic component of plasma membrane // inferred from sequence or structural similarity /// 0030136 // clathrin-coated vesicle // inferred from sequence or structural	0000166 // nucleotide binding // inferred from electronic annotation /// 0004672 // protein kinase activity // inferred from electronic annotation /// 0004674 // protein serine/threonine kinase activity // inferred from sequence or structural similarity /// 0004713 // protein tyrosine kinase activity // inferred from electronic annotation /// 0005112 // Notch binding // inferred from direct assay /// 0005515 // protein binding // inferred from physical interaction /// 0005524 // ATP binding // inferred from electronic annotation /// 0016301 // kinase activity // inferred from electronic annotation /// 0016740 // transferase activity // inferred from electronic annotation /// 0016772 // transferase

215041_s_at	BE259050	DOCK9	Homo sapiens	6-Oct-14	Consensus sequence	GenBank	Consensus includes gb:BE259050 /FEA=EST /DB_XREF=gi:9129548 /DB_XREF=est:601110055F1 /CLONE=IMAGE:3350965 /UG=Hs.307137 Human DNA sequence from clone RP11-155N3 on chromosome 13 Contains ESTs, STSs and GSSs. Contains the 3 part of a novel gene similar to KIAA0694, the KIAA1058 gene and a putative novel gene	BE259050	dedicator of cytokinesis 9	DOCK9	23348	NM_001130048 /// NM_001130049 /// NM_001130050 /// NM_015296 /// XM_005254034 /// XM_005254035 /// XM_005254036 /// XM_006719922 /// XM_006719923 /// XM_006719924 /// XM_006719925 /// XM_006719926 /// XM_006719927 /// XM_006719928 ///	0007264 // small GTPase mediated signal transduction // inferred from electronic annotation /// 0007596 // blood coagulation // traceable author statement /// 0032319 // regulation of Rho GTPase activity // inferred from electronic annotation /// 0032321 // positive regulation of Rho GTPase activity // inferred from electronic annotation /// 0043547 // positive regulation of GTPase activity // inferred from electronic annotation	0005829 // cytosol // traceable author statement /// 0012505 // endomembrane system // inferred from electronic annotation /// 0016020 // membrane // inferred from electronic annotation	0005085 // guanyl-nucleotide exchange factor activity // inferred from electronic annotation /// 0005089 // Rho guanyl-nucleotide exchange factor activity // inferred from electronic annotation /// 0005488 // binding // inferred from electronic annotation /// 0005515 // protein binding // inferred from physical interaction /// 0017048 // Rho GTPase binding // inferred from electronic annotation

215047_at	AL080170	TRIM58	Homo sapiens	6-Oct-14	Consensus sequence	GenBank	Consensus includes gb:AL080170.1 /DEF=Homo sapiens mRNA; cDNA DKFZp434C091 (from clone DKFZp434C091); partial cds. /FEA=mRNA /GEN=DKFZp434C091 /PROD=hypothetical protein /DB_XREF=gi:5262639 /UG=Hs.51692 DKFZP434C091 protein	AL080170	tripartite motif containing 58	TRIM58	25893	NM_015431	0007165 // signal transduction // inferred from electronic annotation /// 0007186 // G-protein coupled receptor signaling pathway // inferred from electronic annotation /// 0007608 // sensory perception of smell // inferred from electronic annotation /// 0050896 // response to stimulus // inferred from electronic annotation /// 0050911 // detection of chemical stimulus involved in sensory perception of smell // inferred from electronic annotation	0005622 // intracellular // inferred from electronic annotation /// 0005886 // plasma membrane // inferred from electronic annotation /// 0005886 // plasma membrane // traceable author statement /// 0016020 // membrane // inferred from electronic annotation /// 0016021 // integral component of membrane // inferred from electronic annotation	0004871 // signal transducer activity // inferred from electronic annotation /// 0004930 // G-protein coupled receptor activity // inferred from electronic annotation /// 0004984 // olfactory receptor activity // inferred from electronic annotation /// 0005515 // protein binding // inferred from electronic annotation /// 0008270 // zinc ion binding // inferred from electronic annotation /// 0046872 // metal ion binding // inferred from electronic annotation

215139_at	AL137508	ARHGEF10	Homo sapiens	6-Oct-14	Consensus sequence	GenBank	Consensus includes gb:AL137508.1 /DEF=Homo sapiens mRNA; cDNA DKFZp761P221 (from clone DKFZp761P221) . /FEA=mRNA /DB_XREF=gi:6808163 /UG=Hs.224403 Homo sapiens mRNA; cDNA DKFZp761P221 (from clone DKFZp761P221)	AL137508	Rho guanine nucleotide exchange factor (GEF) 10	ARHGEF 10	9639	NM_014629 /// XM_005266039 /// XM_005266040 /// XM_005266041 /// XM_005266042 /// XM_006716238 /// XM_006716239 /// XM_006716240 /// XM_006716241 /// XM_006725104 /// XM_006725105 /// XM_006725106 /// XM_006725107 /// XM_006725108 ///	0022011 // myelination in peripheral nervous system // inferred from mutant phenotype /// 0032319 // regulation of Rho GTPase activity // inferred from direct assay /// 0032319 // regulation of Rho GTPase activity // inferred from electronic annotation /// 0032321 // positive regulation of Rho GTPase activity // inferred from direct assay /// 0032321 // positive regulation of Rho GTPase activity // inferred from electronic annotation /// 0033126 // positive regulation of GTP catabolic process // inferred from direct assay /// 0035023 // regulation of Rho protein signal transduction // inferred from electronic annotation /// 0043547 // [illegible]	0005813 // centrosome // inferred from direct assay /// 0005829 // cytosol // non-traceable author statement	0005085 // guanyl-nucleotide exchange factor activity // inferred from electronic annotation /// 0005089 // Rho guanyl-nucleotide exchange factor activity // inferred from direct assay /// 0005515 // protein binding // inferred from physical interaction /// 0019894 // kinesin binding // inferred from physical interaction

215152_at	AI357042	MYB	Homo sapiens	6-Oct-14	Consensus sequence	GenBank	Consensus includes gb:AI357042 /FEA=EST /DB_XREF=gi:4108663 /DB_XREF=est:qx62b04.x1 /CLONE=IMAGE:2005903 /UG=Hs.1334 v-myb avian myeloblastosis viral oncogene homolog	AI357042	v-myb avian myeloblastosis viral oncogene homolog	MYB	4602	NM_001130172 /// NM_001130173 /// NM_001161656 /// NM_001161657 /// NM_001161658 /// NM_001161659 /// NM_001161660 /// NM_005375 /// XM_006715495	0000082 // G1/S transition of mitotic cell cycle // inferred from electronic annotation /// 0000122 // negative regulation of transcription from RNA polymerase II promoter // inferred from mutant phenotype /// 0001701 // in utero embryonic development // inferred from electronic annotation /// 0006260 // DNA replication // inferred from electronic annotation /// 0006338 // chromatin remodeling // inferred from mutant phenotype /// 0006351 // transcription, DNA-templated // inferred from electronic annotation /// 0006355 // regulation of transcription, DNA-templated // non-traceable author [illegible]	0005634 // nucleus // inferred from electronic annotation /// 0016363 // nuclear matrix // non-traceable author statement	0000978 // RNA polymerase II core promoter proximal region sequence-specific DNA binding // inferred from direct assay /// 0001077 // RNA polymerase II core promoter proximal region sequence-specific DNA binding transcription factor activity involved in positive regulation of transcription // inferred by curator /// 0003677 // DNA binding // inferred from electronic annotation /// 0003682 // chromatin binding // inferred from electronic annotation /// 0003700 // sequence-specific DNA binding transcription factor activity // inferred from electronic annotation /// 0005515 // protein binding // inferred from physical interaction

215237_at	AW450751	DOCK9	Homo sapiens	6-Oct-14	Consensus sequence	GenBank	Consensus includes gb:AW450751 /FEA=EST /DB_XREF=gi:6991527 /DB_XREF=est:UI-H-BI3-akq-f-11-0-UI.s1 /CLONE=IMAGE:2735372 /UG=Hs.8021 KIAA1058 protein	AW450751	dedicator of cytokinesis 9	DOCK9	23348	NM_001130048 /// NM_001130049 /// NM_001130050 /// NM_015296 /// XM_005254034 /// XM_005254035 /// XM_005254036 /// XM_006719922 /// XM_006719923 /// XM_006719924 /// XM_006719925 /// XM_006719926 /// XM_006719927 /// XM_006719928 ///	0007264 // small GTPase mediated signal transduction // inferred from electronic annotation /// 0007596 // blood coagulation // traceable author statement /// 0032319 // regulation of Rho GTPase activity // inferred from electronic annotation /// 0032321 // positive regulation of Rho GTPase activity // inferred from electronic annotation /// 0043547 // positive regulation of GTPase activity // inferred from electronic annotation	0005829 // cytosol // traceable author statement /// 0012505 // endomembrane system // inferred from electronic annotation /// 0016020 // membrane // inferred from electronic annotation	0005085 // guanyl-nucleotide exchange factor activity // inferred from electronic annotation /// 0005089 // Rho guanyl-nucleotide exchange factor activity // inferred from electronic annotation /// 0005515 // protein binding // inferred from physical interaction /// 0017048 // Rho GTPase binding // inferred from electronic annotation

215238_s_at	AW450751	DOCK9	Homo sapiens	6-Oct-14	Consensus sequence	GenBank	Consensus includes gb:AW450751 /FEA=EST /DB_XREF=gi:6991527 /DB_XREF=est:UI-H-BI3-akq-f-11-0-UI.s1 /CLONE=IMAGE:2735372 /UG=Hs.8021 KIAA1058 protein	AW450751	dedicator of cytokinesis 9	DOCK9	23348	NM_001130048 /// NM_001130049 /// NM_001130050 /// NM_015296 /// XM_005254034 /// XM_005254035 /// XM_005254036 /// XM_006719922 /// XM_006719923 /// XM_006719924 /// XM_006719925 /// XM_006719926 /// XM_006719927 /// XM_006719928 ///	0007264 // small GTPase mediated signal transduction // inferred from electronic annotation /// 0007596 // blood coagulation // traceable author statement /// 0032319 // regulation of Rho GTPase activity // inferred from electronic annotation /// 0032321 // positive regulation of Rho GTPase activity // inferred from electronic annotation /// 0043547 // positive regulation of GTPase activity // inferred from electronic annotation	0005829 // cytosol // traceable author statement /// 0012505 // endomembrane system // inferred from electronic annotation /// 0016020 // membrane // inferred from electronic annotation	0005085 // guanyl-nucleotide exchange factor activity // inferred from electronic annotation /// 0005089 // Rho guanyl-nucleotide exchange factor activity // inferred from electronic annotation /// 0005488 // binding // inferred from electronic annotation /// 0005515 // protein binding // inferred from physical interaction /// 0017048 // Rho GTPase binding // inferred from electronic annotation

215286_s_at	AL050389	PHTF2	Homo sapiens	6-Oct-14	Consensus sequence	GenBank	Consensus includes gb:AL050389.1 /DEF=Homo sapiens mRNA; cDNA DKFZp564F013 (from clone DKFZp564F013). /FEA=mRNA /PROD=hypothetical protein /DB_XREF=gi:4914570 /UG=Hs.128653 hypothetical protein DKFZp564F013	AL050389	putative homeodomain transcription factor 2	PHTF2	57157	NM_001127357 /// NM_001127358 /// NM_001127359 /// NM_001127360 /// NM_020432 /// XM_005250508 /// XM_005250509 /// XM_006716063	0006351 // transcription, DNA-templated // inferred from electronic annotation /// 0006355 // regulation of transcription, DNA-templated // inferred from electronic annotation	0005634 // nucleus // inferred from electronic annotation /// 0005783 // endoplasmic reticulum // inferred from direct assay	0003677 // DNA binding // inferred from electronic annotation

215438_x_at	BE906054	GSPT1	Homo sapiens	6-Oct-14	Consensus sequence	GenBank	Consensus includes gb:BE906054 /FEA=EST /DB_XREF=gi:10399395 /DB_XREF=est:601497073F1 /CLONE=IMAGE:3899034 /UG=Hs.2707 G1 to S phase transition 1	BE906054	G1 to S phase transition 1	GSPT1	2935	NM_001130006 /// NM_001130007 /// NM_002094 /// XM_005255274 /// XM_005255275	0000082 // G1/S transition of mitotic cell cycle // traceable author statement /// 0000184 // nuclear-transcribed mRNA catabolic process, nonsense-mediated decay // traceable author statement /// 0006184 // GTP catabolic process // inferred from electronic annotation /// 0006184 // GTP catabolic process // traceable author statement /// 0006412 // translation // inferred from electronic annotation /// 0006415 // translational termination // inferred from mutant phenotype /// 0006479 // protein methylation // inferred from direct assay	0005622 // intracellular // non-traceable author statement	0000166 // nucleotide binding // inferred from electronic annotation /// 0003747 // translation release factor activity // inferred from mutant phenotype /// 0003924 // GTPase activity // traceable author statement /// 0005515 // protein binding // inferred from physical interaction /// 0005525 // GTP binding // inferred from electronic annotation /// 0044822 // poly(A) RNA binding // inferred from direct assay

215775_at	BF084105	THBS1	Homo sapiens	6-Oct-14	Consensus sequence	GenBank	Consensus includes gb:BF084105 /FEA=EST /DB_XREF=gi:10877935 /DB_XREF=est: QV4-CT0491-140900-398-e06 /UG=Hs.247954 Human thrombospondin-1 gene, partial cds	BF084105	thrombospondin 1	THBS1	7057	NM_003246	0000187 // activation of MAPK activity // inferred from mutant phenotype /// 0001666 // response to hypoxia // non-traceable author statement /// 0001937 // negative regulation of endothelial cell proliferation // inferred from direct assay /// 0001937 // negative regulation of endothelial cell proliferation // inferred from mutant phenotype /// 0001953 // negative regulation of cell-matrix adhesion // inferred from direct assay /// 0002040 // sprouting angiogenesis // inferred from mutant phenotype /// 0002544 // chronic inflammatory response // inferred from expression pattern /// 0002576 // platelet degranulation // [illegible]	0005576 // extracellular region // traceable author statement /// 0005577 // fibrinogen complex // inferred from direct assay /// 0005615 // extracellular space // inferred from direct assay /// 0005737 // cytoplasm // inferred from electronic annotation /// 0005783 // endoplasmic reticulum // inferred from sequence or structural similarity /// 0009897 // external side of plasma membrane [illegible]	0001786 // phosphatidylserine binding // inferred from direct assay /// 0001948 // glycoprotein binding // non-traceable author statement /// 0001968 // fibronectin binding // inferred from direct assay /// 0005178 // integrin binding // inferred from mutant phenotype /// 0005509 // calcium ion binding // non-traceable author statement /// 0005515 // protein binding // inferred from physical interaction /// 0008201 // heparin binding // inferred from direct assay /// 0017134 // fibroblast growth factor binding // inferred from direct assay /// 0030169 // low-density lipoprotein particle binding // inferred from direct assay /// 0042802 // identical protein binding // non-traceable author [illegible]
216045_at	AB011137	CCDC144A	Homo sapiens	6-Oct-14	Consensus sequence	GenBank	Consensus includes gb:AB011137.2 /DEF=Homo sapiens mRNA for KIAA0565 protein, partial cds. /FEA=mRNA /GEN=KIAA0565 /PROD=KIAA0565 protein /DB_XREF=gi:6683716 /UG=Hs.300938 KIAA0565 gene product	AB011137	coiled-coil domain containing 144A	CCDC144A	9720	NM_014695 /// XM_005256877 /// XM_006721607 /// XM_006721608			

216379_x_at	AK000168	CD24	Homo sapiens	6-Oct-14	Consensus sequence	GenBank	Consensus includes gb:AK000168.1 /DEF=Homo sapiens cDNA FLJ20161 fis, clone COL09252, highly similar to L33930 Homo sapiens CD24 signal transducer mRNA. /FEA=mRNA /DB_XREF=gi:7020079 /UG=Hs.332045 Homo sapiens cDNA FLJ20161 fis, clone COL09252, highly similar to L33930 Homo sapiens CD24 signal transducer	AK000168	CD24 molecule	CD24	1E+08	NM_001291737 /// NM_001291738 /// NM_001291739 /// NM_013230 /// NR_117089 /// NR_117090 /// XM_005278329	0001666 // response to hypoxia // inferred from expression pattern /// 0001775 // cell activation // inferred from direct assay /// 0001959 // regulation of cytokine-mediated signaling pathway // inferred from sequence or structural similarity /// 0002237 // response to molecule of bacterial origin // inferred from sequence or structural similarity /// 0002768 // immune response-regulating cell surface receptor signaling pathway // inferred by curator /// 0007155 // cell adhesion // inferred from electronic annotation /// 0007204 // positive regulation of cytosolic calcium ion concentration // inferred from direct assay /// 0007411 // axon [illegible]	0005886 // plasma membrane // inferred from electronic annotation /// 0009986 // cell surface // inferred from direct assay /// 0016020 // membrane // inferred from direct assay /// 0031225 // anchored component of membrane // inferred from electronic annotation /// 0045121 // membrane raft // inferred from direct assay	0004871 // signal transducer activity // non-traceable author statement /// 0005515 // protein binding // inferred from physical interaction /// 0019901 // protein kinase binding // inferred from physical interaction /// 0030296 // protein tyrosine kinase activator activity // inferred from direct assay

216620_s_at	AF009205	ARHGEF10	Homo sapiens	6-Oct-14	Consensus sequence	GenBank	Consensus includes gb:AF009205.1 /DEF=Homo sapiens clone L5 unknown mRNA, partial cds. /FEA=mRNA /PROD=unknown /DB_XREF=gi:2454511 /UG=Hs.20695 Rho guanine nucleotide exchange factor (GEF) 10	AF009205	Rho guanine nucleotide exchange factor (GEF) 10	ARHGEF 10	9639	NM_014629 /// XM_005266039 /// XM_005266040 /// XM_005266041 /// XM_005266042 /// XM_006716238 /// XM_006716239 /// XM_006716240 /// XM_006716241 /// XM_006725104 /// XM_006725105 /// XM_006725106 /// XM_006725107 /// XM_006725108 ///	0022011 // myelination in peripheral nervous system // inferred from mutant phenotype /// 0032319 // regulation of Rho GTPase activity // inferred from direct assay /// 0032319 // regulation of Rho GTPase activity // inferred from electronic annotation /// 0032321 // positive regulation of Rho GTPase activity // inferred from direct assay /// 0032321 // positive regulation of Rho GTPase activity // inferred from electronic annotation /// 0033126 // positive regulation of GTP catabolic process // inferred from direct assay /// 0035023 // regulation of Rho protein signal transduction // inferred from electronic annotation /// 0043547 // [illegible]	0005813 // centrosome // inferred from direct assay /// 0005829 // cytosol // non-traceable author statement	0005085 // guanyl-nucleotide exchange factor activity // inferred from electronic annotation /// 0005089 // Rho guanyl-nucleotide exchange factor activity // inferred from direct assay /// 0005515 // protein binding // inferred from physical interaction /// 0019894 // kinesin binding // inferred from physical interaction

216686_at	AL137717	ANKRD36BP2	Homo sapiens	6-Oct-14	Consensus sequence	GenBank	Consensus includes gb:AL137717.1 /DEF=Homo sapiens mRNA; cDNA DKFZp434J1630 (from clone DKFZp434J1630). /FEA=mRNA /DB_XREF=gi:6808134 /UG=Hs.306469 Homo sapiens mRNA; cDNA DKFZp434J1630 (from clone DKFZp434J1630)	AL137717	ankyrin repeat domain 36B pseudogene 2	ANKRD36BP2	645784	NR_015424				

216687_x_at	U06641	UGT2B15	Homo sapiens	6-Oct-14	Consensus sequence	GenBank	Consensus includes gb:U06641.1 /DEF=Human UDP glucuronosyltransferase mRNA, partial cds. /FEA=mRNA /PROD=UDP glucuronosyltransferase /DB_XREF=gi:458398 /UG=Hs.150207 UDP glycosyltransferase 2 family, polypeptide B15	U06641	UDP glucuronosyltransferase 2 family, polypeptide B15	UGT2B15	7366	NM_001076	0006805 // xenobiotic metabolic process // traceable author statement /// 0008152 // metabolic process // inferred from electronic annotation /// 0008202 // steroid metabolic process // traceable author statement /// 0052695 // cellular glucuronidation // inferred from direct assay	0005783 // endoplasmic reticulum // inferred from electronic annotation /// 0005789 // endoplasmic reticulum membrane // inferred from electronic annotation /// 0016020 // membrane // inferred from electronic annotation /// 0016021 // integral component of membrane // inferred from electronic annotation /// 0031090 // organelle [illegible]	0001972 // retinoic acid binding // inferred from direct assay /// 0015020 // glucuronosyltransferase activity // inferred from direct assay /// 0016740 // transferase activity // inferred from electronic annotation /// 0016757 // transferase activity, transferring glycosyl groups // inferred from electronic annotation /// 0016758 // transferase activity, transferring hexosyl groups // inferred from electronic annotation

216834_at	S59049	RGS1	Homo sapiens	6-Oct-14	Consensus sequence	GenBank	Consensus includes gb:S59049.1 /DEF=BL34=B cell activation gene human, mRNA, 1398 nt. /FEA=mRNA /GEN=BL34 /DB_XREF=gi:299704 /UG=Hs.75256 regulator of G-protein signalling 1	S59049	regulator of G-protein signaling 1	RGS1	5996	NM_002922	0006955 // immune response // traceable author statement /// 0007165 // signal transduction // non-traceable author statement /// 0007193 // adenylate cyclase-inhibiting G-protein coupled receptor signaling pathway // traceable author statement /// 0009968 // negative regulation of signal transduction // inferred from electronic annotation /// 0038032 // termination of G-protein coupled receptor signaling pathway // inferred from electronic annotation /// 0043547 // positive regulation of GTPase activity // not recorded /// 0043547 // positive regulation of GTPase activity // traceable [illegible]	0005737 // cytoplasm // not recorded /// 0005886 // plasma membrane // not recorded	0005096 // GTPase activator activity // not recorded /// 0005516 // calmodulin binding // traceable author statement

216983_s_at	BC002889	ZNF224	Homo sapiens	6-Oct-14	Consensus sequence	GenBank	Consensus includes gb:BC002889.1 /DEF=Homo sapiens, clone IMAGE:3941350, mRNA, partial cds. /FEA=mRNA /PROD=Unknown (protein for IMAGE:3941350) /DB_XREF=gi:12804072 /UG=Hs.122605 Homo sapiens cDNA: FLJ22124 fis, clone HEP19352	BC002889	zinc finger protein 224	ZNF224	7767	NM_013398 /// XM_005259221	0006351 // transcription, DNA-templated // inferred from electronic annotation /// 0006355 // regulation of transcription, DNA-templated // inferred from electronic annotation /// 0045892 // negative regulation of transcription, DNA-templated // inferred from direct assay	0005622 // intracellular // inferred from electronic annotation /// 0005634 // nucleus // inferred from direct assay /// 0017053 // transcriptional repressor complex // inferred from direct assay /// 0031965 // nuclear membrane // inferred from direct assay	0003676 // nucleic acid binding // inferred from electronic annotation /// 0003677 // DNA binding // inferred from electronic annotation /// 0003700 // sequence-specific DNA binding transcription factor activity // not recorded /// 0005515 // protein binding // inferred from physical interaction /// 0046872 // metal ion binding // inferred from electronic annotation

217028_at	AJ224869	CXCR4	Homo sapiens	6-Oct-14	Consensus sequence	GenBank	Consensus includes gb:AJ224869 /DEF=Homo sapiens CXCR4 gene encoding receptor CXCR4 /FEA=mRNA /DB_XREF=gi:3059119 /UG=Hs.89414 chemokine (C-X-C motif), receptor 4 (fusin)	AJ224869	chemokine (C-X-C motif) receptor 4	CXCR4	7852	NM_001008540 /// NM_003467	0000187 // activation of MAPK activity // traceable author statement /// 0001569 // patterning of blood vessels // inferred from electronic annotation /// 0001666 // response to hypoxia // inferred from expression pattern /// 0001667 // ameboidal cell migration // inferred from electronic annotation /// 0001764 // neuron migration // inferred from electronic annotation /// 0002407 // dendritic cell chemotaxis // traceable author statement /// 0006915 // apoptotic process // traceable author statement /// 0006935 // chemotaxis // inferred from electronic annotation /// 0006954 // inflammatory response // traceable author [illegible]	0005737 // cytoplasm // traceable author statement /// 0005764 // lysosome // inferred from direct assay /// 0005768 // endosome // inferred from electronic annotation /// 0005769 // early endosome // inferred from direct assay /// 0005770 // late endosome // inferred from direct assay /// 0005886 // plasma membrane // inferred from direct assay /// 0005886 // plasma membrane // [illegible]	0001618 // virus receptor activity // inferred from electronic annotation /// 0003779 // actin binding // inferred from direct assay /// 0004871 // signal transducer activity // inferred from electronic annotation /// 0004930 // G-protein coupled receptor activity // traceable author statement /// 0004950 // chemokine receptor activity // inferred from electronic annotation /// 0005515 // protein binding // inferred from physical interaction /// 0015026 // coreceptor activity // traceable author statement /// 0016494 // C-X-C chemokine receptor activity // non-traceable author statement /// 0019955 // cytokine binding // inferred from electronic annotation /// 0031625 // ubiquitin protein ligase binding // [illegible]
217097_s_at	AC004990	PHTF2	Homo sapiens	6-Oct-14	Consensus sequence	GenBank	Consensus includes gb:AC004990 /DEF=Homo sapiens PAC clone RP5-1185I7 from 7q11.23-q21 /FEA=CDS_1 /DB_XREF=gi:3924668 /UG=Hs.128653 hypothetical protein DKFZp564F013	AC004990	putative homeodomain transcription factor 2	PHTF2	57157	NM_001127357 /// NM_001127358 /// NM_001127359 /// NM_001127360 /// NM_020432 /// XM_005250508 /// XM_005250509 /// XM_006716063	0006351 // transcription, DNA-templated // inferred from electronic annotation /// 0006355 // regulation of transcription, DNA-templated // inferred from electronic annotation	0005634 // nucleus // inferred from electronic annotation /// 0005783 // endoplasmic reticulum // inferred from direct assay	0003677 // DNA binding // inferred from electronic annotation

217165_x_at	M10943	MT1F	Homo sapiens	6-Oct-14	Consensus sequence	GenBank	Consensus includes gb:M10943 /DEF=Human metallothionein-If gene (hMT-If) /FEA=CDS /DB_XREF=gi:187540 /UG=Hs.203936 metallothionein 1F (functional)	M10943	metallothionein 1F	MT1F	4494	NM_005949	0045926 // negative regulation of growth // inferred from sequence or structural similarity /// 0071276 // cellular response to cadmium ion // inferred from expression pattern /// 0071294 // cellular response to zinc ion // inferred from sequence or structural similarity	0005634 // nucleus // inferred from direct assay /// 0005737 // cytoplasm // inferred from direct assay /// 0048471 // perinuclear region of cytoplasm // inferred from sequence or structural similarity	0005507 // copper ion binding // non-traceable author statement /// 0008270 // zinc ion binding // inferred from sequence or structural similarity /// 0046870 // cadmium ion binding // non-traceable author statement /// 0046872 // metal ion binding // inferred from electronic annotation
217399_s_at	AF032887	FOXO3	Homo sapiens	6-Oct-14	Consensus sequence	GenBank	Consensus includes gb:AF032887 /DEF=Homo sapiens forkhead (FKHRL1P1) pseudogene, chromosome 17 /FEA=CDS /DB_XREF=gi:2895505 /UG=Hs.274468 forkhead box O3B	AF032887	forkhead box O3 /// forkhead box O3B pseudogene	FOXO3 /// FOXO3B	2309 /// 2310	NM_001455 /// NM_201559 /// NR_026718 /// XM_005266867 /// XM_005266868	0000122 // negative regulation of transcription from RNA polymerase II promoter // inferred from mutant phenotype /// 0001542 // ovulation from ovarian follicle // inferred from electronic annotation /// 0001544 // initiation of primordial ovarian follicle growth // inferred from electronic annotation /// 0001547 // antral ovarian follicle growth // inferred from electronic annotation /// 0001556 // oocyte maturation // inferred from electronic annotation /// 0006351 // transcription, DNA-templated // inferred from electronic annotation /// 0006355 // regulation of transcription, DNA-templated // inferred [illegible]	0005634 // nucleus // inferred from direct assay /// 0005654 // nucleoplasm // traceable author statement /// 0005737 // cytoplasm // inferred from direct assay /// 0005829 // cytosol // not recorded /// 0016020 // membrane // inferred from electronic annotation	0001047 // core promoter binding // inferred from sequence or structural similarity /// 0003677 // DNA binding // inferred from direct assay /// 0003700 // sequence-specific DNA binding transcription factor activity // inferred from direct assay /// 0005515 // protein binding // inferred from physical interaction /// 0008301 // DNA binding, bending // not recorded /// 0019901 // protein kinase binding // inferred from physical interaction /// 0031490 // chromatin DNA binding // inferred from sequence or structural similarity /// 0043565 // sequence-specific DNA binding // inferred from direct assay

217503_at	AA203487	STK17B	Homo sapiens	6-Oct-14	Consensus sequence	GenBank	Consensus includes gb:AA203487 /FEA=EST /DB_XREF=gi:1799460 /DB_XREF=est:zx53d03.r1 /CLONE=IMAGE:446213 /UG=Hs.314363 ESTs	AA203487	serine/threonine kinase 17b	STK17B	9262	NM_004226	0006468 // protein phosphorylation // inferred from direct assay /// 0006915 // apoptotic process // inferred from electronic annotation /// 0012501 // programmed cell death // inferred from electronic annotation /// 0016310 // phosphorylation // inferred from electronic annotation /// 0035556 // intracellular signal transduction // inferred from direct assay /// 0046777 // protein autophosphorylation // inferred from sequence or structural similarity /// 2000271 // positive regulation of fibroblast apoptotic process // inferred from mutant phenotype	0005634 // nucleus // inferred from direct assay /// 0005793 // endoplasmic reticulum-Golgi intermediate compartment // inferred from electronic annotation /// 0005886 // plasma membrane // inferred from electronic annotation /// 0015629 // actin cytoskeleton // inferred from electronic annotation /// 0016020 // membrane // inferred from electronic annotation	0000166 // nucleotide binding // inferred from electronic annotation /// 0004672 // protein kinase activity // inferred from sequence or structural similarity /// 0004674 // protein serine/threonine kinase activity // inferred from direct assay /// 0004713 // protein tyrosine kinase activity // inferred from electronic annotation /// 0005515 // protein binding // inferred from electronic annotation /// 0005524 // ATP binding // inferred from direct assay /// 0016301 // kinase activity // inferred from electronic annotation /// 0016740 // transferase activity // inferred from electronic annotation /// 0016772 // transferase activity, transferring phosphorus-containing groups // inferred from [illegible]

217595_at	AV701723	GSPT1	Homo sapiens	6-Oct-14	Consensus sequence	GenBank	Consensus includes gb:AV701723 /FEA=EST /DB_XREF=gi:10718053 /DB_XREF=est:AV701723 /CLONE=ADBCTC02 /UG=Hs.237849 ESTs	AV701723	G1 to S phase transition 1	GSPT1	2935	NM_001130006 /// NM_001130007 /// NM_002094 /// XM_005255274 /// XM_005255275	0000082 // G1/S transition of mitotic cell cycle // traceable author statement /// 0000184 // nuclear-transcribed mRNA catabolic process, nonsense-mediated decay // traceable author statement /// 0006184 // GTP catabolic process // inferred from electronic annotation /// 0006184 // GTP catabolic process // traceable author statement /// 0006412 // translation // inferred from electronic annotation /// 0006415 // translational termination // inferred from mutant phenotype /// 0006479 // protein methylation // inferred from direct assay	0005622 // intracellular // non-traceable author statement	0000166 // nucleotide binding // inferred from electronic annotation /// 0003747 // translation release factor activity // inferred from mutant phenotype /// 0003924 // GTPase activity // traceable author statement /// 0005515 // protein binding // inferred from physical interaction /// 0005525 // GTP binding // inferred from electronic annotation /// 0044822 // poly(A) RNA binding // inferred from direct assay
217663_at	AW264320	ZNF234	Homo sapiens	6-Oct-14	Consensus sequence	GenBank	Consensus includes gb:AW264320 /FEA=EST /DB_XREF=gi:6641062 /DB_XREF=est:xq98c06.x1 /CLONE=IMAGE:2758666 /UG=Hs.235992 ESTs	AW264320	zinc finger protein 234	ZNF234	10780	NM_001144824 /// NM_006630 /// XM_006722973 /// XM_006722974 /// XM_006722975	0006351 // transcription, DNA-templated // inferred from electronic annotation /// 0006355 // regulation of transcription, DNA-templated // not recorded	0005622 // intracellular // inferred from electronic annotation /// 0005634 // nucleus // inferred from direct assay	0003676 // nucleic acid binding // inferred from electronic annotation /// 0003677 // DNA binding // inferred from electronic annotation /// 0003700 // sequence-specific DNA binding transcription factor activity // not recorded /// 0046872 // metal ion binding // inferred from electronic annotation

217738_at	BF575514	NAMPT	Homo sapiens	6-Oct-14	Consensus sequence	GenBank	Consensus includes gb:BF575514 /FEA=EST /DB_XREF=gi:11649318 /DB_XREF=est:602133090F1 /CLONE=IMAGE:4288079 /UG=Hs.239138 pre-B-cell colony-enhancing factor /FL=gb:U02020.1 gb:NM_005746.1	BF575514	nicotinamide phosphoribosyltransferase	NAMPT	10135	NM_005746 /// NM_182790 /// XM_005250100	0006766 // vitamin metabolic process // traceable author statement /// 0006767 // water-soluble vitamin metabolic process // traceable author statement /// 0006769 // nicotinamide metabolic process // traceable author statement /// 0007165 // signal transduction // traceable author statement /// 0007267 // cell-cell signaling // traceable author statement /// 0008284 // positive regulation of cell proliferation // traceable author statement /// 0008286 // insulin receptor signaling pathway // inferred from direct assay /// 0009435 // NAD biosynthetic process // inferred from [illegible]	0005576 // extracellular region // inferred from electronic annotation /// 0005615 // extracellular space // inferred from electronic annotation /// 0005634 // nucleus // inferred from electronic annotation /// 0005737 // cytoplasm // inferred from electronic annotation /// 0005829 // cytosol // traceable author statement /// 0070062 // extracellular vesicular exosome // inferred from direct assay	0004514 // nicotinate-nucleotide diphosphorylase (carboxylating) activity // inferred from electronic annotation /// 0004516 // nicotinate phosphoribosyltransferase activity // inferred from electronic annotation /// 0005125 // cytokine activity // inferred from electronic annotation /// 0005515 // protein binding // inferred from physical interaction /// 0016740 // transferase activity // inferred from electronic annotation /// 0016757 // transferase activity, transferring glycosyl groups // inferred from electronic annotation /// 0047280 // nicotinamide phosphoribosyltransferase activity // inferred from electronic annotation

217739_s_at	NM_005746	NAMPT	Homo sapiens	6-Oct-14	Exemplar sequence	GenBank	gb:NM_005746.1 /DEF=Homo sapiens pre-B-cell colony-enhancing factor (PBEF), mRNA. /FEA=mRNA /GEN=PBEF /PROD=pre-B-cell colony-enhancing factor /DB_XREF=gi:5031976 /UG=Hs.239138 pre-B-cell colony-enhancing factor /FL=gb:U02020.1 gb:NM_005746.1	NM_005746	nicotinamide phosphoribosyltransferase	NAMPT	10135	NM_005746 /// NM_182790 /// XM_005250100	0006766 // vitamin metabolic process // traceable author statement /// 0006767 // water-soluble vitamin metabolic process // traceable author statement /// 0006769 // nicotinamide metabolic process // traceable author statement /// 0007165 // signal transduction // traceable author statement /// 0007267 // cell-cell signaling // traceable author statement /// 0008284 // positive regulation of cell proliferation // traceable author statement /// 0008286 // insulin receptor signaling pathway // inferred from direct assay /// 0009435 // NAD biosynthetic process // inferred from [illegible]	0005576 // extracellular region // inferred from electronic annotation /// 0005615 // extracellular space // inferred from electronic annotation /// 0005634 // nucleus // inferred from electronic annotation /// 0005737 // cytoplasm // inferred from electronic annotation /// 0005829 // cytosol // traceable author statement /// 0070062 // extracellular vesicular exosome // inferred from direct assay	0004514 // nicotinate-nucleotide diphosphorylase (carboxylating) activity // inferred from electronic annotation /// 0004516 // nicotinate phosphoribosyltransferase activity // inferred from electronic annotation /// 0005125 // cytokine activity // inferred from electronic annotation /// 0005515 // protein binding // inferred from physical interaction /// 0016740 // transferase activity // inferred from electronic annotation /// 0016757 // transferase activity, transferring glycosyl groups // inferred from electronic annotation /// 0047280 // nicotinamide phosphoribosyltransferase activity // inferred from electronic annotation

217837_s_at	NM_016079	CHMP3	Homo sapiens	6-Oct-14	Exemplar sequence	GenBank	gb:NM_016079.1 /DEF=Homo sapiens CGI-149 protein (LOC51652), mRNA. /FEA=mRNA /GEN=LOC51652 /PROD=CGI-149 protein /DB_XREF=gi:7706352 /UG=Hs.189658 CGI-149 protein /FL=gb:BC004419.1 gb:AF151907.1 gb:AF219226.1 gb:NM_016079.1	NM_016079	charged multivesicular body protein 3 /// RNF103-CHMP3 readthrough	CHMP3 /// RNF103-CHMP3	51652 /// 100526767	NM_001005753 /// NM_001193517 /// NM_001198954 /// NM_016079 /// NR_036454	0006810 // transport // inferred from electronic annotation /// 0006915 // apoptotic process // inferred from electronic annotation /// 0007049 // cell cycle // inferred from electronic annotation /// 0015031 // protein transport // inferred from electronic annotation /// 0016032 // viral process // traceable author statement /// 0016197 // endosomal transport // traceable author statement /// 0019058 // viral life cycle // traceable author statement /// 0050792 // regulation of viral process // inferred from mutant phenotype /// 0051301 // cell division // inferred from electronic annotation /// 0061024 // membrane organization // traceable [illegible]	0005737 // cytoplasm // inferred from electronic annotation /// 0005768 // endosome // inferred from electronic annotation /// 0005829 // cytosol // traceable author statement /// 0016020 // membrane // inferred from electronic annotation /// 0031902 // late endosome membrane // inferred from electronic annotation /// 0070062 // extracellular vesicular exosome [illegible]	0005515 // protein binding // inferred from physical interaction

217867_x_at	NM_012105	BACE2	Homo sapiens	6-Oct-14	Exemplar sequence	GenBank	gb:NM_012105.1 /DEF=Homo sapiens beta-site APP-cleaving enzyme 2 (BACE2), mRNA. /FEA=mRNA /GEN=BACE2 /PROD=beta-site APP-cleaving enzyme 2 /DB_XREF=gi:6912263 /UG=Hs.271411 beta-site APP-cleaving enzyme 2 /FL=gb:AF117892.1 gb:AF050171.1 gb:AF200192.1 gb:AF200342.1 gb:AF204944.1 gb:AF178532.1	NM_012105	beta-site APP-cleaving enzyme 2	BACE2	25825	NM_012105 /// NM_138991 /// NM_138992	0006508 // proteolysis // non-traceable author statement /// 0006509 // membrane protein ectodomain proteolysis // inferred from direct assay /// 0016486 // peptide hormone processing // non-traceable author statement /// 0042985 // negative regulation of amyloid precursor protein biosynthetic process // inferred from mutant phenotype	0005768 // endosome // inferred from electronic annotation /// 0005783 // endoplasmic reticulum // inferred from electronic annotation /// 0005794 // Golgi apparatus // inferred from direct assay /// 0009986 // cell surface // inferred from electronic annotation /// 0016020 // membrane // inferred from electronic annotation /// 0016021 // integral component of	0004190 // aspartic-type endopeptidase activity // inferred from direct assay /// 0008233 // peptidase activity // inferred from electronic annotation /// 0016787 // hydrolase activity // inferred from electronic annotation

217906_at	NM_014315	KLHDC2	Homo sapiens	6-Oct-14	Exemplar sequence	GenBank	gb:NM_014315.1 /DEF=Homo sapiens host cell factor homolog (LCP), mRNA. /FEA=mRNA /GEN=LCP /PROD=host cell factor homolog /DB_XREF=gi:7657300 /UG=Hs.20597 host cell factor homolog /FL=gb:BC002335.1 gb:AF113131.1 gb:NM_014315.1 gb:AF244137.1	NM_014315	kelch domain containing 2	KLHDC2	23588	NM_014315 /// XM_006720094			0005634 // nucleus // inferred from electronic annotation	0005515 // protein binding // inferred from electronic annotation

218240_at	NM_017595	NKIRAS2	Homo sapiens	6-Oct-14	Exemplar sequence	GenBank	gb:NM_017595.1 /DEF=Homo sapiens I-kappa-B-interacting Ras-like protein 2 (KBRAS2), mRNA. /FEA=mRNA /GEN=KBRAS2 /PROD=I-kappa-B-interacting Ras-like protein 2 /DB_XREF=gi:8922150 /UG=Hs.22937 I-kappa-B-interacting Ras-like protein 2 /FL=gb:NM_017595.1	NM_017595	NFKB inhibitor interacting Ras-like 2	NKIRAS2	28511	NM_001001349 /// NM_001144927 /// NM_001144928 /// NM_001144929 /// NM_017595 /// XM_005257251	0006184 // GTP catabolic process // inferred from electronic annotation /// 0007165 // signal transduction // inferred from electronic annotation /// 0007249 // I-kappaB kinase/NF-kappaB signaling // non-traceable author statement /// 0007264 // small GTPase mediated signal transduction // inferred from electronic annotation /// 0015031 // protein transport // inferred from electronic annotation	0005622 // intracellular // inferred from electronic annotation /// 0005737 // cytoplasm // inferred from electronic annotation /// 0016020 // membrane // inferred from electronic annotation	0000166 // nucleotide binding // inferred from electronic annotation /// 0003924 // GTPase activity // non-traceable author statement /// 0005525 // GTP binding // inferred from electronic annotation

218368_s_at	NM_016639	TNFRSF12A	Homo sapiens	6-Oct-14	Exemplar sequence	GenBank	gb:NM_016639.1 /DEF=Homo sapiens type I transmembrane protein Fn14 (FN14), mRNA. /FEA=mRNA /GEN=FN14 /PROD=type I transmembrane protein Fn14 /DB_XREF=gi:7706185 /UG=Hs.10086 type I transmembrane protein Fn14 /FL=gb:BC002718.1 gb:AB035480.1 gb:NM_016639.1 gb:AF191148.1	NM_016639	tumor necrosis factor receptor superfamily, member 12A	TNFRSF12A	51330	NM_016639	0001525 // angiogenesis // inferred from electronic annotation /// 0006915 // apoptotic process // inferred from electronic annotation /// 0006928 // cellular component movement // traceable author statement /// 0006931 // substrate-dependent cell migration, cell attachment to substrate // inferred from electronic annotation /// 0007155 // cell adhesion // inferred from electronic annotation /// 0007275 // multicellular organismal development // inferred from electronic annotation /// 0007275 // multicellular organismal development // traceable author statement /// 0008219 // cell death // inferred from electronic annotation /// 0030154 // [illegible]	0001726 // ruffle // inferred from electronic annotation /// 0005886 // plasma membrane // inferred from electronic annotation /// 0009986 // cell surface // inferred from electronic annotation /// 0016020 // membrane // inferred from electronic annotation /// 0016021 // integral component of membrane // inferred from electronic annotation	0005515 // protein binding // inferred from physical interaction

218373_at	NM_022476	AKTIP	Homo sapiens	6-Oct-14	Exemplar sequence	GenBank	gb:NM_022476.1 /DEF=Homo sapiens hypothetical protein FLJ13258 similar to fused toes (FLJ13258), mRNA. /FEA=mRNA /GEN=FLJ13258 /PROD=hypothetical protein FLJ13258 similar to fusedtoes /DB_XREF=gi:11968026 /UG=Hs.288929 hypothetical protein FLJ13258 similar to fused toes /FL=gb:NM_022476.1	NM_022476	AKT interacting protein	AKTIP	64400	NM_001012398 /// NM_022476 /// XM_005256094 /// XM_005256095 /// XM_005256096 /// XM_005256097 /// XM_005256098	0001934 // positive regulation of protein phosphorylation // inferred from direct assay /// 0006810 // transport // inferred from electronic annotation /// 0006915 // apoptotic process // inferred from electronic annotation /// 0007032 // endosome organization // inferred from mutant phenotype /// 0007040 // lysosome organization // inferred from mutant phenotype /// 0008152 // metabolic process // inferred from electronic annotation /// 0008333 // endosome to lysosome transport // inferred from mutant phenotype /// 0015031 // protein transport // inferred from electronic annotation /// 0032092 // positive regulation of protein [illegible]	0005730 // nucleolus // inferred from direct assay /// 0005737 // cytoplasm // inferred from electronic annotation /// 0005737 // cytoplasm // inferred from direct assay /// 0005794 // Golgi apparatus // inferred from direct assay /// 0005886 // plasma membrane // inferred from direct assay /// 0016020 // membrane // inferred from electronic annotation /// 0030897 // HOPS [illegible]	0005515 // protein binding // inferred from physical interaction /// 0016881 // acid-amino acid ligase activity // inferred from electronic annotation /// 0019787 // small conjugating protein ligase activity // non-traceable author statement

218507_at	NM_013332	HILPDA	Homo sapiens	6-Oct-14	Exemplar sequence	GenBank	gb:NM_013332.1 /DEF=Homo sapiens hypoxia-inducible protein 2 (HIG2), mRNA. /FEA=mRNA /GEN=HIG2 /PROD=hypoxia-inducible protein 2 /DB_XREF=gi:7019408 /UG=Hs.61762 hypoxia-inducible protein 2 /FL=gb:BC001863.1 gb:AF144755.1 gb:NM_013332.1	NM_013332	hypoxia inducible lipid droplet-associated	HILPDA	29923	NM_001098786 /// NM_013332	0001819 // positive regulation of cytokine production // inferred from direct assay /// 0006950 // response to stress // non-traceable author statement /// 0008284 // positive regulation of cell proliferation // inferred from direct assay /// 0010884 // positive regulation of lipid storage // inferred from direct assay /// 0035425 // autocrine signaling // inferred from direct assay	0005576 // extracellular region // inferred from electronic annotation /// 0005615 // extracellular space // inferred from direct assay /// 0005811 // lipid particle // inferred from direct assay /// 0009986 // cell surface // inferred from direct assay /// 0016020 // membrane // inferred from electronic annotation /// 0016021 // integral component of membrane // inferred from electronic annotation /// [illegible]	0005102 // receptor binding // inferred from physical interaction /// 0005515 // protein binding // inferred from physical interaction

218533_s_at	NM_017859	UCKL1	Homo sapiens	6-Oct-14	Exemplar sequence	GenBank	gb:NM_017859.1 /DEF=Homo sapiens hypothetical protein FLJ20517 (FLJ20517), mRNA. /FEA=mRNA /GEN=FLJ20517 /PROD=hypothetical protein FLJ20517 /DB_XREF=gi:8923486 /UG=Hs.39850 hypothetical protein FLJ20517 /FL=gb:NM_017859.1	NM_017859	uridine-cytidine kinase 1-like 1	UCKL1	54963	NM_001193379 /// NM_017859 /// XM_005260216 /// XM_006723806 /// XM_006723807 /// XM_006723808 /// XM_006723809 /// XM_006723810 /// XM_006723811 /// XM_006723812 /// XM_006723813 /// XR_244120 /// XR_244121 /// XR_430299 /// XR_430300 /// XR_430301 /// XR_430302 /// XR_430303	0008152 // metabolic process // inferred from electronic annotation /// 0016032 // viral process // inferred from electronic annotation /// 0016310 // phosphorylation // inferred from electronic annotation /// 0019048 // modulation by virus of host morphology or physiology // inferred from electronic annotation /// 0044206 // UMP salvage // inferred from electronic annotation /// 0044211 // CTP salvage // inferred from electronic annotation	0005634 // nucleus // inferred from electronic annotation /// 0005737 // cytoplasm // inferred from electronic annotation /// 0005783 // endoplasmic reticulum // inferred from direct assay	0000166 // nucleotide binding // inferred from electronic annotation /// 0004849 // uridine kinase activity // inferred from electronic annotation /// 0005515 // protein binding // inferred from physical interaction /// 0005524 // ATP binding // inferred from electronic annotation /// 0016301 // kinase activity // inferred from electronic annotation /// 0016740 // transferase activity // inferred from electronic annotation /// 0016773 // phosphotransferase activity, alcohol group as acceptor // inferred from electronic annotation

218598_at	NM_021930	RINT1	Homo sapiens	6-Oct-14	Exemplar sequence	GenBank	gb:NM_021930.1 /DEF=Homo sapiens hypothetical protein FLJ11785 (FLJ11785), mRNA. /FEA=mRNA /GEN=FLJ11785 /PROD=hypothetical protein FLJ11785 /DB_XREF=gi:11345465 /UG=Hs.44625 Rad50-interacting protein 1 /FL=gb:NM_021930.1 gb:AF317622.1	NM_021930	RAD50 interactor 1	RINT1	60561	NM_021930 /// XM_005250524	0006810 // transport // inferred from electronic annotation /// 0007049 // cell cycle // inferred from electronic annotation /// 0015031 // protein transport // inferred from electronic annotation /// 0016192 // vesicle-mediated transport // inferred from electronic annotation /// 0031572 // G2 DNA damage checkpoint // inferred from mutant phenotype	0005737 // cytoplasm // inferred from electronic annotation /// 0005737 // cytoplasm // inferred from direct assay /// 0005783 // endoplasmic reticulum // inferred from direct assay /// 0005789 // endoplasmic reticulum membrane // inferred from electronic annotation /// 0016020 // membrane // inferred from electronic annotation	0005515 // protein binding // inferred from physical interaction

218781_at	NM_024624	SMC6	Homo sapiens	6-Oct-14	Exemplar sequence	GenBank	gb:NM_024624.1 /DEF=Homo sapiens hypothetical protein FLJ22116 (FLJ22116), mRNA. /FEA=mRNA /GEN=FLJ22116 /PROD=hypothetical protein FLJ22116 /DB_XREF=gi:13375847 /UG=Hs.34497 hypothetical protein FLJ22116 /FL=gb:AL136544.1 gb:NM_024624.1	NM_024624	structural maintenance of chromosomes 6	SMC6	79677	NM_001142286 /// NM_024624 /// XM_005262628 /// XM_006712103	0000722 // telomere maintenance via recombination // inferred from mutant phenotype /// 0000724 // double-strand break repair via homologous recombination // inferred from electronic annotation /// 0006281 // DNA repair // inferred from electronic annotation /// 0006310 // DNA recombination // inferred from electronic annotation /// 0006974 // cellular response to DNA damage stimulus // inferred from electronic annotation /// 0090398 // cellular senescence // inferred from mutant phenotype	0000781 // chromosome, telomeric region // inferred from direct assay /// 0005622 // intracellular // inferred from direct assay /// 0005634 // nucleus // inferred from direct assay /// 0005694 // chromosome // inferred from electronic annotation /// 0005730 // nucleolus // inferred from direct assay /// 0016605 // PML body // inferred from direct assay /// 0030915 // Smc5-Smc6 complex // [illegible]	0000166 // nucleotide binding // inferred from electronic annotation /// 0005515 // protein binding // inferred from physical interaction /// 0005524 // ATP binding // inferred from electronic annotation

218904_s_at	NM_017998	C9orf40	Homo sapiens	6-Oct-14	Exemplar sequence	GenBank	gb:NM_017998.1 /DEF=Homo sapiens hypothetical protein FLJ10110 (FLJ10110), mRNA. /FEA=mRNA /GEN=FLJ10110 /PROD=hypothetical protein FLJ10110 /DB_XREF=gi:8922232 /UG=Hs.264363 hypothetical protein FLJ10110 /FL=gb:NM_017998.1	NM_017998	chromosome 9 open reading frame 40	C9orf40	55071	NM_017998			

219014_at	NM_016619	PLAC8	Homo sapiens	6-Oct-14	Exemplar sequence	GenBank	gb:NM_016619.1 /DEF=Homo sapiens hypothetical protein (LOC51316), mRNA. /FEA=mRNA /GEN=LOC51316 /PROD=hypothetical protein /DB_XREF=gi:7706157 /UG=Hs.107139 hypothetical protein /FL=gb:AF208846.1 gb:NM_016619.1	NM_016619	placenta-specific 8	PLAC8	51316	NM_001130715 /// NM_001130716 /// NM_016619	0008284 // positive regulation of cell proliferation // inferred from electronic annotation /// 0009409 // response to cold // inferred from electronic annotation /// 0040015 // negative regulation of multicellular organism growth // inferred from electronic annotation /// 0042742 // defense response to bacterium // inferred from electronic annotation /// 0043066 // negative regulation of apoptotic process // inferred from electronic annotation /// 0045944 // positive regulation of transcription from RNA polymerase II promoter // inferred from electronic annotation /// 0050873 // brown fat cell differentiation // inferred [illegible]		0003682 // chromatin binding // inferred from electronic annotation

219037_at	NM_016052	RRP15	Homo sapiens	6-Oct-14	Exemplar sequence	GenBank	gb:NM_016052.1 /DEF=Homo sapiens CGI-115 protein (LOC51018), mRNA. /FEA=mRNA /GEN=LOC51018 /PROD=CGI-115 protein /DB_XREF=gi:7705619 /UG=Hs.56043 CGI-115 protein /FL=gb:AF151873.1 gb:NM_016052.1	NM_016052	ribosomal RNA processing 15 homolog (S. cerevisiae)	RRP15	51018	NM_016052		0005634 // nucleus // inferred from direct assay /// 0005730 // nucleolus // inferred from direct assay /// 0005739 // mitochondrion // inferred from direct assay	

219168_s_at	NM_017701	PRR5	Homo sapiens	6-Oct-14	Exemplar sequence	GenBank	gb:NM_017701.1 /DEF=Homo sapiens hypothetical protein FLJ20185 (FLJ20185), mRNA. /FEA=mRNA /GEN=FLJ20185 /PROD=hypothetical protein FLJ20185 /DB_XREF=gi:8923174 /UG=Hs.272972 hypothetical protein FLJ20185 /FL=gb:NM_017701.1	NM_017701	proline rich 5 (renal)	PRR5	55615	NM_001017528 /// NM_001017529 /// NM_001017530 /// NM_001198721 /// NM_015366 /// NM_181333	0001934 // positive regulation of protein phosphorylation // inferred from electronic annotation /// 0007049 // cell cycle // inferred from electronic annotation /// 0007165 // signal transduction // inferred from electronic annotation /// 0007264 // small GTPase mediated signal transduction // traceable author statement /// 0014068 // positive regulation of phosphatidylinositol 3-kinase signaling // inferred from electronic annotation /// 0030036 // actin cytoskeleton organization // inferred from direct assay /// 0030335 // positive regulation of cell migration // inferred from direct assay /// 0032321 [illegible]	0005622 // intracellular // inferred from electronic annotation /// 0005829 // cytosol // traceable author statement /// 0031932 // TORC2 complex // inferred from direct assay	0005096 // GTPase activator activity // inferred from electronic annotation /// 0005096 // GTPase activator activity // inferred from direct assay /// 0005100 // Rho GTPase activator activity // inferred from direct assay /// 0005515 // protein binding // inferred from physical interaction

219175_s_at	NM_017836	SLC41A3	Homo sapiens	6-Oct-14	Exemplar sequence	GenBank	gb:NM_017836.1 /DEF=Homo sapiens hypothetical protein FLJ20473 (FLJ20473), mRNA. /FEA=mRNA /GEN=FLJ20473 /PROD=hypothetical protein FLJ20473 /DB_XREF=gi:8923438 /UG=Hs.330592 hypothetical protein FLJ20473 /FL=gb:NM_017836.1	NM_017836	solute carrier family 41, member 3	SLC41A3	54946	NM_001008485 /// NM_001008486 /// NM_001008487 /// NM_001164475 /// NM_017836 /// XM_005247559 /// XM_005247560 /// XM_005247561 /// XM_005247562 /// XM_005247563 /// XM_005247564 /// XM_005247565 /// XM_006713681	0006812 // cation transport // inferred from electronic annotation /// 0008152 // metabolic process // inferred from electronic annotation	0005886 // plasma membrane // inferred from direct assay /// 0016020 // membrane // inferred from electronic annotation /// 0016021 // integral component of membrane // inferred from electronic annotation	0008324 // cation transmembrane transporter activity // inferred from electronic annotation

219259_at	NM_022367	SEMA4A	Homo sapiens	6-Oct-14	Exemplar sequence	GenBank	gb:NM_022367.1 /DEF=Homo sapiens hypothetical protein FLJ12287 similar to semaphorins (FLJ12287), mRNA. /FEA=mRNA /GEN=FLJ12287 /PROD=hypothetical protein FLJ12287 similar tosemaphorins /DB_XREF=gi:11641290 /UG=Hs.7634 hypothetical protein FLJ12287 similar to semaphorins /FL=gb:NM_022367.1 [illegible]	NM_022367	sema domain, immunoglobulin domain (Ig), transmembrane domain (TM) and short cytoplasmic domain, (semaphorin) 4A	SEMA4A	64218	NM_001193300 /// NM_001193301 /// NM_001193302 /// NM_022367	0001525 // angiogenesis // inferred from electronic annotation /// 0002292 // T cell differentiation involved in immune response // inferred from electronic annotation /// 0002376 // immune system process // inferred from electronic annotation /// 0007275 // multicellular organismal development // inferred from electronic annotation /// 0007399 // nervous system development // inferred from electronic annotation /// 0007409 // axonogenesis // inferred from electronic annotation /// 0007411 // axon guidance // traceable author statement /// 0008360 // regulation of cell shape // inferred from electronic annotation /// [illegible]	0005886 // plasma membrane // traceable author statement /// 0016020 // membrane // inferred from electronic annotation /// 0016021 // integral component of membrane // inferred from electronic annotation	0004872 // receptor activity // inferred from electronic annotation /// 0005515 // protein binding // inferred from physical interaction

219416_at	NM_016240	SCARA3	Homo sapiens	6-Oct-14	Exemplar sequence	GenBank	gb:NM_016240.1 /DEF=Homo sapiens CSR1 protein (CSR1), mRNA. /FEA=mRNA /GEN=CSR1 /PROD=CSR1 protein /DB_XREF=gi:7705335 /UG=Hs.128856 CSR1 protein /FL=gb:AB007829.1 gb:NM_016240.1	NM_016240	scavenger receptor class A, member 3	SCARA3	51435	NM_016240 /// NM_182826 /// XM_005273527	0006898 // receptor-mediated endocytosis // traceable author statement /// 0006979 // response to oxidative stress // traceable author statement /// 0009650 // UV protection // traceable author statement	0000139 // Golgi membrane // inferred from electronic annotation /// 0005581 // collagen trimer // inferred from electronic annotation /// 0005783 // endoplasmic reticulum // inferred from electronic annotation /// 0005789 // endoplasmic reticulum membrane // inferred from electronic annotation /// 0005794 // Golgi apparatus // inferred from electronic [illegible]	0005044 // scavenger receptor activity // traceable author statement

219418_at	NM_024782	NHEJ1	Homo sapiens	6-Oct-14	Exemplar sequence	GenBank	gb:NM_024782.1 /DEF=Homo sapiens hypothetical protein FLJ12610 (FLJ12610), mRNA. /FEA=mRNA /GEN=FLJ12610 /PROD=hypothetical protein FLJ12610 /DB_XREF=gi:13376141 /UG=Hs.146139 hypothetical protein FLJ12610 /FL=gb:NM_024782.1	NM_024782	nonhomologous end-joining factor 1 /// solute carrier family 23, member 3	NHEJ1 /// SLC23A3	79840 /// 151295	NM_001144889 /// NM_001144890 /// NM_024782 /// NM_144712	0006281 // DNA repair // inferred from electronic annotation /// 0006302 // double-strand break repair // inferred from electronic annotation /// 0006303 // double-strand break repair via nonhomologous end joining // inferred from mutant phenotype /// 0006310 // DNA recombination // inferred from electronic annotation /// 0006810 // transport // inferred from electronic annotation /// 0006974 // cellular response to DNA damage stimulus // inferred from electronic annotation /// 0007417 // central nervous system development // non-traceable author statement /// 0010212 // response to ionizing radiation // inferred from [illegible]	0005634 // nucleus // inferred from direct assay /// 0016020 // membrane // inferred from electronic annotation /// 0016021 // integral component of membrane // inferred from electronic annotation /// 0070419 // nonhomologous end joining complex // inferred from direct assay	0003677 // DNA binding // inferred from electronic annotation /// 0005215 // transporter activity // inferred from electronic annotation /// 0005515 // protein binding // inferred from physical interaction

219422_at	U72763	ESPN	Homo sapiens	6-Oct-14	Consensus sequence	GenBank	Consensus includes gb:U72763.1 /DEF=Human death receptor 3 (DR3) mRNA, complete cds. /FEA=CDS /GEN=DR3 /PROD=death receptor 3 /DB_XREF=gi:1669511 /UG=Hs.180338 tumor necrosis factor receptor superfamily, member 12 (translocating chain-association membrane protein) /FL=gb:U72763.1 gb:U74611.1 gb:U78029.1 gb:U94501.1 [illegible]	U72763	espin	ESPN	83715	NM_031475 /// XM_005263501	0007605 // sensory perception of sound // inferred from electronic annotation /// 0007626 // locomotory behavior // inferred from electronic annotation /// 0030046 // parallel actin filament bundle assembly // inferred from electronic annotation /// 0051017 // actin filament bundle assembly // inferred from electronic annotation /// 0051491 // positive regulation of filopodium assembly // inferred from electronic annotation /// 0051494 // negative regulation of cytoskeleton organization // inferred from electronic annotation	0005737 // cytoplasm // inferred from electronic annotation /// 0005856 // cytoskeleton // inferred from electronic annotation /// 0005902 // microvillus // inferred from electronic annotation /// 0005903 // brush border // inferred from sequence or structural similarity /// 0015629 // actin cytoskeleton // inferred from electronic annotation /// 0031941 // filamentous actin [illegible]	0003779 // actin binding // inferred from electronic annotation /// 0017124 // SH3 domain binding // inferred from sequence or structural similarity /// 0051015 // actin filament binding // inferred from sequence or structural similarity

219481_at	NM_024525	TTC13	Homo sapiens	6-Oct-14	Exemplar sequence	GenBank	gb:NM_024525.1 /DEF=Homo sapiens hypothetical protein FLJ22584 (FLJ22584), mRNA. /FEA=mRNA /GEN=FLJ22584 /PROD=hypothetical protein FLJ22584 /DB_XREF=gi:13375669 /UG=Hs.4947 hypothetical protein FLJ22584 /FL=gb:NM_024525.1	NM_024525	tetratricopeptide repeat domain 13	TTC13	79573	NM_001122835 /// NM_024525 /// XM_005273260 /// XM_005273261 /// XM_005273262 /// XM_005273264 /// XM_006711814 /// XM_006711815			0005515 // protein binding // inferred from electronic annotation			

219630_at	NM_005764	PDZK1IP1	Homo sapiens	6-Oct-14	Exemplar sequence	GenBank	gb:NM_005764.1 /DEF=Homo sapiens epithelial protein up-regulated in carcinoma, membrane associated protein 17 (DD96), mRNA. /FEA=mRNA /GEN=DD96 /PROD=epithelial protein up-regulated in carcinoma,membrane associated protein 17 /DB_XREF=gi:5031656 /UG=Hs.271473 epithelial protein up-regulated in carcinoma, [illegible]	NM_005764	PDZK1 interacting protein 1	PDZK1IP1	10158	NM_005764		0016020 // membrane // inferred from electronic annotation /// 0016021 // integral component of membrane // inferred from electronic annotation /// 0070062 // extracellular vesicular exosome // inferred from direct assay	

219646_at	NM_017702	DEF8	Homo sapiens	6-Oct-14	Exemplar sequence	GenBank	gb:NM_017702.1 /DEF=Homo sapiens hypothetical protein FLJ20186 (FLJ20186), mRNA. /FEA=mRNA /GEN=FLJ20186 /PROD=hypothetical protein FLJ20186 /DB_XREF=gi:8923176 /UG=Hs.65021 hypothetical protein FLJ20186 /FL=gb:NM_017702.1	NM_017702	differentially expressed in FDCP 8 homolog (mouse)	DEF8	54849	NM_001242816 /// NM_001242817 /// NM_001242818 /// NM_001242819 /// NM_001242820 /// NM_001242821 /// NM_001242822 /// NM_017702 /// NM_207514 /// XM_005256316 /// XM_005256318 /// XM_006721207 /// XM_006721208 /// XM_006721209 /// XM_006721210	0035556 // intracellular signal transduction // inferred from electronic annotation		0008270 // zinc ion binding // inferred from electronic annotation /// 0046872 // metal ion binding // inferred from electronic annotation

219708_at	NM_020201	NT5M	Homo sapiens	6-Oct-14	Exemplar sequence	GenBank	gb:NM_020201.1 /DEF=Homo sapiens 5(3)-deoxyribonucleotidase (dNT-2); nuclear gene for mitochondrial product (LOC56953), mRNA. /FEA=mRNA /GEN=LOC56953 /PROD=5(3)-deoxyribonucleotidase (dNT-2); nuclear genefor mitochondrial product /DB_XREF=gi:9910371 /UG=Hs.166145 5(3)-deoxyribonucleotidase (dNT-2); nuclear [illegible]	NM_020201	5',3'-nucleotidase, mitochondrial	NT5M	56953	NM_020201 /// XM_005256731 /// XM_005256732 /// XM_005256733 /// XM_005256734 /// XM_005256737	0006206 // pyrimidine nucleobase metabolic process // traceable author statement /// 0006260 // DNA replication // traceable author statement /// 0009117 // nucleotide metabolic process // inferred from electronic annotation /// 0009223 // pyrimidine deoxyribonucleotide catabolic process // traceable author statement /// 0016311 // dephosphorylation // not recorded /// 0016311 // dephosphorylation // inferred from electronic annotation /// 0016311 // dephosphorylation // traceable author statement /// 0044281 // small molecule metabolic process // traceable author statement /// [illegible]	0005739 // mitochondrion // traceable author statement /// 0005759 // mitochondrial matrix // traceable author statement	0000166 // nucleotide binding // inferred from electronic annotation /// 0008252 // nucleotidase activity // traceable author statement /// 0008253 // 5'-nucleotidase activity // not recorded /// 0016787 // hydrolase activity // inferred from electronic annotation /// 0016791 // phosphatase activity // inferred from electronic annotation /// 0046872 // metal ion binding // inferred from electronic annotation

219863_at	NM_016323	HERC5	Homo sapiens	6-Oct-14	Exemplar sequence	GenBank	gb:NM_016323.1 /DEF=Homo sapiens cyclin-E binding protein 1 (LOC51191), mRNA. /FEA=mRNA /GEN=LOC51191 /PROD=cyclin-E binding protein 1 /DB_XREF=gi:7705930 /UG=Hs.26663 cyclin-E binding protein 1 /FL=gb:AB027289.1 gb:NM_016323.1	NM_016323	HECT and RLD domain containing E3 ubiquitin protein ligase 5	HERC5	51191	NM_016323	0000079 // regulation of cyclin-dependent protein serine/threonine kinase activity // traceable author statement /// 0002376 // immune system process // inferred from electronic annotation /// 0008152 // metabolic process // inferred from electronic annotation /// 0016567 // protein ubiquitination // inferred from electronic annotation /// 0019221 // cytokine-mediated signaling pathway // traceable author statement /// 0032020 // ISG15-protein conjugation // inferred from direct assay /// 0032480 // negative regulation of type I interferon production // traceable author statement /// 0042787 // protein [illegible]	0005634 // nucleus // not recorded /// 0005737 // cytoplasm // not recorded /// 0005829 // cytosol // traceable author statement /// 0048471 // perinuclear region of cytoplasm // inferred from electronic annotation	0004842 // ubiquitin-protein transferase activity // not recorded /// 0005515 // protein binding // inferred from physical interaction /// 0016874 // ligase activity // inferred from electronic annotation /// 0042296 // ISG15 ligase activity // inferred from direct assay /// 0044822 // poly(A) RNA binding // inferred from direct assay

220019_s_at	NM_005774	ZNF224	Homo sapiens	6-Oct-14	Exemplar sequence	GenBank	gb:NM_005774.1 /DEF=Homo sapiens zinc finger protein 255 (ZNF255), mRNA. /FEA=mRNA /GEN=ZNF255 /PROD=zinc finger protein 255 /DB_XREF=gi:5031614 /UG=Hs.181696 zinc finger protein 255 /FL=gb:AF067164.1 gb:NM_005774.1	NM_005774	zinc finger protein 224	ZNF224	7767	NM_013398 /// XM_005259221	0006351 // transcription, DNA-templated // inferred from electronic annotation /// 0006355 // regulation of transcription, DNA-templated // inferred from electronic annotation /// 0045892 // negative regulation of transcription, DNA-templated // inferred from direct assay	0005622 // intracellular // inferred from electronic annotation /// 0005634 // nucleus // inferred from direct assay /// 0017053 // transcriptional repressor complex // inferred from direct assay /// 0031965 // nuclear membrane // inferred from direct assay	0003676 // nucleic acid binding // inferred from electronic annotation /// 0003677 // DNA binding // inferred from electronic annotation /// 0003700 // sequence-specific DNA binding transcription factor activity // not recorded /// 0005515 // protein binding // inferred from physical interaction /// 0046872 // metal ion binding // inferred from electronic annotation

220088_at	NM_001736	C5AR1	Homo sapiens	6-Oct-14	Exemplar sequence	GenBank	gb:NM_001736.1 /DEF=Homo sapiens complement component 5 receptor 1 (C5a ligand) (C5R1), mRNA. /FEA=mRNA /GEN=C5R1 /PROD=complement component 5 receptor 1 (C5a ligand) /DB_XREF=gi:4502508 /UG=Hs.2161 complement component 5 receptor 1 (C5a ligand) /FL=gb:M62505.1 gb:NM_001736.1	NM_001736	complement component 5a receptor 1	C5AR1	728	NM_001736 /// XM_005259190	0000187 // activation of MAPK activity // traceable author statement /// 0002430 // complement receptor mediated signaling pathway // inferred from electronic annotation /// 0006915 // apoptotic process // inferred from electronic annotation /// 0006935 // chemotaxis // traceable author statement /// 0006954 // inflammatory response // inferred from electronic annotation /// 0006955 // immune response // traceable author statement /// 0006968 // cellular defense response // traceable author statement /// 0007165 // signal transduction // traceable author statement /// 0007186 // G-protein coupled [illegible]	0005886 // plasma membrane // traceable author statement /// 0005887 // integral component of plasma membrane // traceable author statement /// 0009986 // cell surface // inferred from electronic annotation /// 0016020 // membrane // inferred from electronic annotation /// 0016021 // integral component of membrane // inferred from electronic annotation /// 0016323 // [illegible]	0001856 // complement component C5a binding // inferred from electronic annotation /// 0004871 // signal transducer activity // inferred from electronic annotation /// 0004878 // complement component C5a receptor activity // inferred from direct assay /// 0004930 // G-protein coupled receptor activity // inferred from electronic annotation /// 0004942 // anaphylatoxin receptor activity // inferred from electronic annotation /// 0004944 // C5a anaphylatoxin receptor activity // inferred from direct assay /// 0004982 // N-formyl peptide receptor activity // inferred from electronic annotation

220236_at	NM_017990	PDPR	Homo sapiens	6-Oct-14	Exemplar sequence	GenBank	gb:NM_017990.2 /DEF=Homo sapiens hypothetical protein FLJ10079 (FLJ10079), mRNA. /FEA=mRNA /GEN=FLJ10079 /PROD=hypothetical protein FLJ10079 /DB_XREF=gi:13775157 /UG=Hs.261215 hypothetical protein FLJ10079 /FL=gb:NM_017990.2	NM_017990	pyruvate dehydrogenase phosphatase regulatory subunit	PDPR	55066	NM_017990 /// XM_005256015 /// XM_005256016 /// XM_005256019 /// XM_006721224 /// XM_006721225 /// XM_006721226 /// XR_429721	0006090 // pyruvate metabolic process // traceable author statement /// 0006546 // glycine catabolic process // inferred from electronic annotation /// 0010510 // regulation of acetyl-CoA biosynthetic process from pyruvate // traceable author statement /// 0032259 // methylation // inferred from electronic annotation /// 0044237 // cellular metabolic process // traceable author statement /// 0044281 // small molecule metabolic process // traceable author statement /// 0055114 // oxidation-reduction process // inferred from electronic annotation	0005737 // cytoplasm // inferred from direct assay /// 0005739 // mitochondrion // inferred from electronic annotation /// 0005759 // mitochondrial matrix // traceable author statement	0004047 // aminomethyltransferase activity // inferred from electronic annotation /// 0016491 // oxidoreductase activity // inferred from electronic annotation

220313_at	NM_022049	GPR88	Homo sapiens	6-Oct-14	Exemplar sequence	GenBank	gb:NM_022049.1 /DEF=Homo sapiens G-protein coupled receptor 88 (GPR88), mRNA. /FEA=mRNA /GEN=GPR88 /PROD=G-protein coupled receptor 88 /DB_XREF=gi:11545752 /UG=Hs.170053 G-protein coupled receptor 88 /FL=gb:AB042410.1 gb:NM_022049.1	NM_022049	G protein-coupled receptor 88	GPR88	54112	NM_022049	0007165 // signal transduction // inferred from electronic annotation /// 0007186 // G-protein coupled receptor signaling pathway // non-traceable author statement /// 0007626 // locomotory behavior // inferred from electronic annotation /// 0019228 // neuronal action potential // inferred from electronic annotation /// 0050885 // neuromuscular process controlling balance // inferred from electronic annotation	0005886 // plasma membrane // inferred from electronic annotation /// 0016020 // membrane // inferred from electronic annotation /// 0016021 // integral component of membrane // inferred from electronic annotation	0004871 // signal transducer activity // inferred from electronic annotation /// 0004930 // G-protein coupled receptor activity // inferred from electronic annotation

220320_at	NM_024872	DOK3	Homo sapiens	6-Oct-14	Exemplar sequence	GenBank	gb:NM_024872.1 /DEF=Homo sapiens hypothetical protein FLJ22570 (FLJ22570), mRNA. /FEA=mRNA /GEN=FLJ22570 /PROD=hypothetical protein FLJ22570 /DB_XREF=gi:13376307 /UG=Hs.205450 hypothetical protein FLJ22570 /FL=gb:NM_024872.1	NM_024872	docking protein 3	DOK3	79930	NM_001144875 /// NM_001144876 /// NM_024872 /// XM_005265984 /// XM_005265985 /// XM_005265987 /// XM_006714920	0007265 // Ras protein signal transduction // inferred from electronic annotation	0005737 // cytoplasm // inferred from electronic annotation /// 0005886 // plasma membrane // inferred from electronic annotation /// 0016020 // membrane // inferred from electronic annotation	0005158 // insulin receptor binding // inferred from electronic annotation /// 0005515 // protein binding // inferred from physical interaction /// 0005543 // phospholipid binding // inferred from electronic annotation

220363_s_at	NM_022086	ELMO2	Homo sapiens	6-Oct-14	Exemplar sequence	GenBank	gb:NM_022086.1 /DEF=Homo sapiens hypothetical protein FLJ11656 (FLJ11656), mRNA. /FEA=mRNA /GEN=FLJ11656 /PROD=hypothetical protein FLJ11656 /DB_XREF=gi:11545798 /UG=Hs.96560 hypothetical protein FLJ11656 /FL=gb:NM_022086.1	NM_022086	engulfment and cell motility 2	ELMO2	63916	NM_022086 /// NM_133171 /// NM_182764 /// XM_005260496 /// XM_005260497 /// XM_005260498 /// XM_005260499 /// XM_005260500 /// XM_005260501 /// XM_005260502 /// XM_006723854 /// XM_006723855	0006909 // phagocytosis // inferred from electronic annotation /// 0006915 // apoptotic process // inferred from electronic annotation /// 0038096 // Fc-gamma receptor signaling pathway involved in phagocytosis // traceable author statement /// 0045087 // innate immune response // traceable author statement /// 0060326 // cell chemotaxis // inferred from mutant phenotype	0005737 // cytoplasm // inferred from electronic annotation /// 0005829 // cytosol // inferred from direct assay /// 0005829 // cytosol // traceable author statement /// 0005856 // cytoskeleton // inferred from electronic annotation /// 0016020 // membrane // inferred from direct assay	0005488 // binding // inferred from electronic annotation /// 0005515 // protein binding // inferred from physical interaction /// 0005543 // phospholipid binding // inferred from electronic annotation /// 0017124 // SH3 domain binding // inferred from electronic annotation /// 0030971 // receptor tyrosine kinase binding // inferred from physical interaction

220395_at	NM_018602	DNAJA4	Homo sapiens	6-Oct-14	Exemplar sequence	GenBank	gb:NM_018602.1 /DEF=Homo sapiens hypothetical protein PRO1472 (PRO1472), mRNA. /FEA=mRNA /GEN=PRO1472 /PROD=hypothetical protein PRO1472 /DB_XREF=gi:8924043 /UG=Hs.154662 DnaJ (Hsp40) homolog, subfamily A, member 4 /FL=gb:AF116663.1 gb:NM_018602.1	NM_018602	DnaJ (Hsp40) homolog, subfamily A, member 4	DNAJA4	55466	NM_001130182 /// NM_001130183 /// NM_018602 /// XM_005254535 /// XM_006720601	0006457 // protein folding // inferred from electronic annotation /// 0009408 // response to heat // inferred from electronic annotation /// 0042026 // protein refolding // inferred from direct assay /// 0090084 // negative regulation of inclusion body assembly // inferred from direct assay	0005829 // cytosol // inferred from direct assay /// 0016020 // membrane // inferred from direct assay	0005524 // ATP binding // inferred from electronic annotation /// 0031072 // heat shock protein binding // inferred from electronic annotation /// 0046872 // metal ion binding // inferred from electronic annotation /// 0051082 // unfolded protein binding // inferred from direct assay /// 0051087 // chaperone binding // inferred from physical interaction

220464_at	NM_024979	MCF2L	Homo sapiens	6-Oct-14	Exemplar sequence	GenBank	gb:NM_024979.1 /DEF=Homo sapiens hypothetical protein FLJ12122 (FLJ12122), mRNA. /FEA=mRNA /GEN=FLJ12122 /PROD=hypothetical protein FLJ12122 /DB_XREF=gi:13376481 /UG=Hs.287488 hypothetical protein FLJ12122 /FL=gb:NM_024979.1	NM_024979	MCF.2 cell line derived transforming sequence-like	MCF2L	23263	NM_001112732 /// NM_024979 /// XM_005268307 /// XM_005268308 /// XM_005268309 /// XM_005268310 /// XM_005268312 /// XM_005268313 /// XM_005268314 /// XM_005268315 /// XM_005268316 /// XM_006719966 /// XM_006719967 /// XM_006719968 /// XR_429282	0007264 // small GTPase mediated signal transduction // traceable author statement /// 0007266 // Rho protein signal transduction // inferred from electronic annotation /// 0032319 // regulation of Rho GTPase activity // inferred from electronic annotation /// 0032321 // positive regulation of Rho GTPase activity // inferred from electronic annotation /// 0035023 // regulation of Rho protein signal transduction // inferred from electronic annotation /// 0035556 // intracellular signal transduction // inferred from electronic annotation /// 0043065 // positive regulation of apoptotic process // traceable author [illegible]	0005615 // extracellular space // inferred from direct assay /// 0005737 // cytoplasm // inferred from electronic annotation /// 0005829 // cytosol // traceable author statement /// 0005886 // plasma membrane // inferred from electronic annotation /// 0016020 // membrane // inferred from electronic annotation /// 0030027 // lamellipodium // inferred from electronic annotation	0005085 // guanyl-nucleotide exchange factor activity // inferred from electronic annotation /// 0005089 // Rho guanyl-nucleotide exchange factor activity // inferred from electronic annotation /// 0005545 // 1-phosphatidylinositol binding // inferred from electronic annotation

220792_at	NM_018699	PRDM5	Homo sapiens	6-Oct-14	Exemplar sequence	GenBank	gb:NM_018699.1 /DEF=Homo sapiens PR domain containing 5 (PRDM5), mRNA. /FEA=mRNA /GEN=PRDM5 /PROD=PR domain containing 5 /DB_XREF=gi:8923963 /UG=Hs.192867 PR domain containing 5 /FL=gb:AF272897.1 gb:NM_018699.1	NM_018699	PR domain containing 5	PRDM5	11107	NM_018699 /// XM_005262706 /// XM_005262707 /// XM_005262708 /// XM_006714072	0000278 // mitotic cell cycle // inferred from mutant phenotype /// 0006351 // transcription, DNA-templated // inferred from electronic annotation /// 0006355 // regulation of transcription, DNA-templated // inferred from electronic annotation /// 0016568 // chromatin modification // inferred from electronic annotation /// 0016575 // histone deacetylation // inferred from mutant phenotype /// 0032259 // methylation // inferred from electronic annotation /// 0045892 // negative regulation of transcription, DNA-templated // inferred from direct assay /// 0051567 // histone H3-K9 methylation // inferred [illegible]	0005634 // nucleus // inferred from direct assay	0000976 // transcription regulatory region sequence-specific DNA binding // inferred from electronic annotation /// 0003676 // nucleic acid binding // inferred from electronic annotation /// 0003677 // DNA binding // inferred from electronic annotation /// 0005515 // protein binding // inferred from physical interaction /// 0008168 // methyltransferase activity // inferred from electronic annotation /// 0016740 // transferase activity // inferred from electronic annotation /// 0043565 // sequence-specific DNA binding // inferred from direct assay /// 0044212 // transcription regulatory region DNA binding // inferred from direct assay /// 0046872 // metal ion binding // inferred from electronic annotation [illegible]

221311_x_at	NM_020466	LYRM2	Homo sapiens	6-Oct-14	Exemplar sequence	GenBank	gb:NM_020466.1 /DEF=Homo sapiens hypothetical protein dJ122O8.2 (DJ122O8.2), mRNA. /FEA=CDS /GEN=DJ122O8.2 /PROD=hypothetical protein dJ122O8.2 /DB_XREF=gi:10092688 /UG=Hs.132094 hypothetical protein dJ122O8.2 /FL=gb:NM_020466.1	NM_020466	LYR motif containing 2	LYRM2	57226	NM_020466 /// NR_028493 /// NR_028494 /// NR_028495		0005739 // mitochondrion // inferred from electronic annotation	

221391_at	NM_023922	TAS2R14	Homo sapiens	6-Oct-14	Exemplar sequence	GenBank	gb:NM_023922.1 /DEF=Homo sapiens taste receptor, family B, member 1 (TRB1), mRNA. /FEA=CDS /GEN=TRB1 /PROD=taste receptor, family B, member 1 /DB_XREF=gi:12965181 /UG=Hs.272394 taste receptor, type 2, member 14 /FL=gb:NM_023922.1	NM_023922	taste receptor, type 2, member 14	TAS2R14	50840	NM_023922	0001580 // detection of chemical stimulus involved in sensory perception of bitter taste // inferred from direct assay /// 0007165 // signal transduction // inferred from electronic annotation /// 0007186 // G-protein coupled receptor signaling pathway // inferred by curator /// 0050896 // response to stimulus // inferred from electronic annotation /// 0050909 // sensory perception of taste // inferred from electronic annotation	0005886 // plasma membrane // traceable author statement /// 0016020 // membrane // inferred from electronic annotation /// 0016021 // integral component of membrane // inferred by curator	0004871 // signal transducer activity // inferred from electronic annotation /// 0004930 // G-protein coupled receptor activity // inferred from electronic annotation /// 0008527 // taste receptor activity // inferred from direct assay /// 0008527 // taste receptor activity // traceable author statement /// 0033038 // bitter taste receptor activity // inferred from direct assay

221450_x_at	NM_018933	PCDHB13	Homo sapiens	6-Oct-14	Exemplar sequence	GenBank	gb:NM_018933.1 /DEF=Homo sapiens protocadherin beta 13 (PCDHB13), mRNA. /FEA=CDS /GEN=PCDHB13 /PROD=protocadherin beta 13 /DB_XREF=gi:11036653 /UG=Hs.283803 protocadherin beta 13 /FL=gb:NM_018933.1 gb:AF152492.1	NM_018933	protocadherin beta 13	PCDHB13	56123	NM_018933	0007155 // cell adhesion // inferred from electronic annotation /// 0007156 // homophilic cell adhesion // inferred from electronic annotation /// 0007268 // synaptic transmission // traceable author statement /// 0007416 // synapse assembly // traceable author statement /// 0016339 // calcium-dependent cell-cell adhesion // non-traceable author statement	0005886 // plasma membrane // inferred from electronic annotation /// 0016020 // membrane // inferred from electronic annotation /// 0016021 // integral component of membrane // non-traceable author statement	0005509 // calcium ion binding // inferred from electronic annotation

221478_at	AL132665	BNIP3L	Homo sapiens	6-Oct-14	Consensus sequence	GenBank	Consensus includes gb:AL132665.1 /DEF=Homo sapiens mRNA; cDNA DKFZp566E034 (from clone DKFZp566E034) ; complete cds. /FEA=mRNA /DB_XREF=gi:6137021 /UG=Hs.132955 BCL2adenovirus E1B 19kD-interacting protein 3-like /FL=gb:AF060922.1 gb:AB004788.1 gb:AF067396.1 gb:NM_004331.1 gb:AL132665.1	AL132665	BCL2/adenovirus E1B 19kDa interacting protein 3-like	BNIP3L	665	NM_004331 /// XM_005273617	0006915 // apoptotic process // inferred from electronic annotation /// 0016032 // viral process // inferred from electronic annotation /// 0019048 // modulation by virus of host morphology or physiology // inferred from electronic annotation /// 0035694 // mitochondrial protein catabolic process // inferred from mutant phenotype /// 0043065 // positive regulation of apoptotic process // inferred from direct assay /// 0043066 // negative regulation of apoptotic process // inferred from direct assay /// 0051607 // defense response to virus // inferred from direct assay /// 0097345 // mitochondrial outer membrane [illegible]	0005634 // nucleus // inferred from electronic annotation /// 0005635 // nuclear envelope // inferred from direct assay /// 0005739 // mitochondrion // inferred from direct assay /// 0005740 // mitochondrial envelope // inferred from electronic annotation /// 0005741 // mitochondrial outer membrane // inferred from mutant phenotype /// 0005783 // endoplasmic reticulum // inferred from [illegible]	0005515 // protein binding // inferred from physical interaction /// 0005521 // lamin binding // inferred from direct assay /// 0005521 // lamin binding // inferred from physical interaction /// 0042802 // identical protein binding // inferred from physical interaction /// 0042803 // protein homodimerization activity // inferred from direct assay /// 0046982 // protein heterodimerization activity // inferred from direct assay

221479_s_at	AF060922	BNIP3L	Homo sapiens	6-Oct-14	Exemplar sequence	GenBank	gb:AF060922.1 /DEF=Homo sapiens clone 016a05 My020 protein mRNA, complete cds. /FEA=mRNA /PROD=My020 protein /DB_XREF=gi:12001981 /UG=Hs.132955 BCL2adenovirus E1B 19kD-interacting protein 3-like /FL=gb:AF060922.1 gb:AB004788.1 gb:AF067396.1 gb:NM_004331.1 gb:AL132665.1	AF060922	BCL2/adenovirus E1B 19kDa interacting protein 3-like	BNIP3L	665	NM_004331 /// XM_005273617	0006915 // apoptotic process // inferred from electronic annotation /// 0016032 // viral process // inferred from electronic annotation /// 0019048 // modulation by virus of host morphology or physiology // inferred from electronic annotation /// 0035694 // mitochondrial protein catabolic process // inferred from mutant phenotype /// 0043065 // positive regulation of apoptotic process // inferred from direct assay /// 0043066 // negative regulation of apoptotic process // inferred from direct assay /// 0051607 // defense response to virus // inferred from direct assay /// 0097345 // mitochondrial outer membrane [illegible]	0005634 // nucleus // inferred from electronic annotation /// 0005635 // nuclear envelope // inferred from direct assay /// 0005739 // mitochondrion // inferred from direct assay /// 0005740 // mitochondrial envelope // inferred from electronic annotation /// 0005741 // mitochondrial outer membrane // inferred from mutant phenotype /// 0005783 // endoplasmic reticulum // inferred from [illegible]	0005515 // protein binding // inferred from physical interaction /// 0005521 // lamin binding // inferred from direct assay /// 0005521 // lamin binding // inferred from physical interaction /// 0042802 // identical protein binding // inferred from physical interaction /// 0042803 // protein homodimerization activity // inferred from direct assay /// 0046982 // protein heterodimerization activity // inferred from direct assay

221528_s_at	BC000143	ELMO2	Homo sapiens	6-Oct-14	Exemplar sequence	GenBank	gb:BC000143.1 /DEF=Homo sapiens, Similar to hypothetical protein FLJ11656, clone MGC:5247, mRNA, complete cds. /FEA=mRNA /PROD=Similar to hypothetical protein FLJ11656 /DB_XREF=gi:12652782 /UG=Hs.96560 hypothetical protein FLJ11656 /FL=gb:BC000143.1	BC000143	engulfment and cell motility 2	ELMO2	63916	NM_022086 /// NM_133171 /// NM_182764 /// XM_005260496 /// XM_005260497 /// XM_005260498 /// XM_005260499 /// XM_005260500 /// XM_005260501 /// XM_005260502 /// XM_006723854 /// XM_006723855	0006909 // phagocytosis // inferred from electronic annotation /// 0006915 // apoptotic process // inferred from electronic annotation /// 0038096 // Fc-gamma receptor signaling pathway involved in phagocytosis // traceable author statement /// 0045087 // innate immune response // traceable author statement /// 0060326 // cell chemotaxis // inferred from mutant phenotype	0005737 // cytoplasm // inferred from electronic annotation /// 0005829 // cytosol // inferred from direct assay /// 0005829 // cytosol // traceable author statement /// 0005856 // cytoskeleton // inferred from electronic annotation /// 0016020 // membrane // inferred from direct assay	0005488 // binding // inferred from electronic annotation /// 0005515 // protein binding // inferred from physical interaction /// 0005543 // phospholipid binding // inferred from electronic annotation /// 0017124 // SH3 domain binding // inferred from electronic annotation /// 0030971 // receptor tyrosine kinase binding // inferred from physical interaction

221593_s_at	BC001663	RPL31	Homo sapiens	6-Oct-14	Exemplar sequence	GenBank	gb:BC001663.1 /DEF=Homo sapiens, Similar to ribosomal protein L31, clone MGC:1641, mRNA, complete cds. /FEA=mRNA /PROD=Similar to ribosomal protein L31 /DB_XREF=gi:12804504 /UG=Hs.164170 vascular Rab-GAPTBC-containing /FL=gb:BC001663.1	BC001663	ribosomal protein L31	RPL31	6160	NM_000993 /// NM_001098577 /// NM_001099693	0000184 // nuclear-transcribed mRNA catabolic process, nonsense-mediated decay // traceable author statement /// 0006412 // translation // non-traceable author statement /// 0006412 // translation // traceable author statement /// 0006413 // translational initiation // traceable author statement /// 0006414 // translational elongation // traceable author statement /// 0006415 // translational termination // traceable author statement /// 0006614 // SRP-dependent cotranslational protein targeting to membrane // traceable author statement /// 0010467 // gene expression // traceable [illegible]	0005622 // intracellular // inferred from electronic annotation /// 0005829 // cytosol // traceable author statement /// 0005840 // ribosome // inferred from electronic annotation /// 0016020 // membrane // inferred from direct assay /// 0022625 // cytosolic large ribosomal subunit // inferred from direct assay /// 0030529 // ribonucleoprotein complex // inferred from electronic [illegible]	0003723 // RNA binding // traceable author statement /// 0003735 // structural constituent of ribosome // non-traceable author statement /// 0044822 // poly(A) RNA binding // inferred from direct assay

221643_s_at	AF016005	RERE	Homo sapiens	6-Oct-14	Exemplar sequence	GenBank	gb:AF016005.1 /DEF=Homo sapiens chromosome 1 atrophin-1 related protein (DRPLA) mRNA, complete cds. /FEA=mRNA /GEN=DRPLA /PROD=atrophin-1 related protein /DB_XREF=gi:3411014 /UG=Hs.194369 arginine-glutamic acid dipeptide (RE) repeats /FL=gb:AF016005.1	AF016005	arginine-glutamic acid dipeptide (RE) repeats	RERE	473	NM_001042681 /// NM_001042682 /// NM_012102 /// XM_005263464 /// XM_005263465 /// XM_005263466 /// XM_006710653 /// XM_006710654	0006338 // chromatin remodeling // inferred from electronic annotation /// 0006351 // transcription, DNA-templated // inferred from electronic annotation /// 0006355 // regulation of transcription, DNA-templated // inferred from electronic annotation /// 0006607 // NLS-bearing protein import into nucleus // traceable author statement /// 0007275 // multicellular organismal development // inferred from electronic annotation	0000118 // histone deacetylase complex // inferred from electronic annotation /// 0005634 // nucleus // non-traceable author statement /// 0005739 // mitochondrion // inferred from direct assay	0003677 // DNA binding // inferred from electronic annotation /// 0003682 // chromatin binding // inferred from electronic annotation /// 0003700 // sequence-specific DNA binding transcription factor activity // inferred from electronic annotation /// 0005515 // protein binding // inferred from physical interaction /// 0008267 // poly-glutamine tract binding // traceable author statement /// 0008270 // zinc ion binding // inferred from electronic annotation /// 0043565 // sequence-specific DNA binding // inferred from electronic annotation /// 0046872 // metal ion binding // inferred from electronic annotation

221649_s_at	BC000535	PPAN	Homo sapiens	6-Oct-14	Exemplar sequence	GenBank	gb:BC000535.1 /DEF=Homo sapiens, clone MGC:3007, mRNA, complete cds. /FEA=mRNA /PROD=Unknown (protein for MGC:3007) /DB_XREF=gi:12653526 /UG=Hs.65302 peter pan (Drosophila) homolog /FL=gb:BC000535.1	BC000535	peter pan homolog (Drosophila) /// PPAN-P2RY11 readthrough	PPAN /// PPAN-P2RY11	56342 /// 692312	NM_001040664 /// NM_001198690 /// NM_020230	0001973 // adenosine receptor signaling pathway // inferred from direct assay /// 0006952 // defense response // traceable author statement /// 0007165 // signal transduction // inferred from electronic annotation /// 0007186 // G-protein coupled receptor signaling pathway // inferred from direct assay /// 0007186 // G-protein coupled receptor signaling pathway // inferred from electronic annotation /// 0007190 // activation of adenylate cyclase activity // traceable author statement /// 0007200 // phospholipase C-activating G-protein coupled receptor signaling pathway // traceable author statement /// [illegible]	0005634 // nucleus // inferred from direct assay /// 0005730 // nucleolus // inferred from electronic annotation /// 0005737 // cytoplasm // inferred from direct assay /// 0005886 // plasma membrane // inferred from electronic annotation /// 0005886 // plasma membrane // traceable author statement /// 0005887 // integral component of plasma membrane // inferred by curator /// [illegible]	0004871 // signal transducer activity // inferred from electronic annotation /// 0004872 // receptor activity // traceable author statement /// 0004930 // G-protein coupled receptor activity // inferred from electronic annotation /// 0030594 // neurotransmitter receptor activity // inferred from direct assay /// 0044822 // poly(A) RNA binding // inferred from direct assay /// 0045028 // G-protein coupled purinergic nucleotide receptor activity // inferred from electronic annotation /// 0045031 // ATP-activated nucleotide receptor activity // inferred from direct assay

222105_s_at	AA452565	NKIRAS2	Homo sapiens	6-Oct-14	Consensus sequence	GenBank	Consensus includes gb:AA452565 /FEA=EST /DB_XREF=gi:2166234 /DB_XREF=est:zx35g11.s1 /CLONE=IMAGE:788516 /UG=Hs.22937 I-kappa-B-interacting Ras-like protein 2 /FL=gb:AF229840.1	AA452565	NFKB inhibitor interacting Ras-like 2	NKIRAS2	28511	NM_001001349 /// NM_001144927 /// NM_001144928 /// NM_001144929 /// NM_017595 /// XM_005257251	0006184 // GTP catabolic process // inferred from electronic annotation /// 0007165 // signal transduction // inferred from electronic annotation /// 0007249 // I-kappaB kinase/NF-kappaB signaling // non-traceable author statement /// 0007264 // small GTPase mediated signal transduction // inferred from electronic annotation /// 0015031 // protein transport // inferred from electronic annotation	0005622 // intracellular // inferred from electronic annotation /// 0005737 // cytoplasm // inferred from electronic annotation /// 0016020 // membrane // inferred from electronic annotation	0000166 // nucleotide binding // inferred from electronic annotation /// 0003924 // GTPase activity // non-traceable author statement /// 0005525 // GTP binding // inferred from electronic annotation
222279_at	AI669379	HLA-F-AS1	Homo sapiens	6-Oct-14	Consensus sequence	GenBank	Consensus includes gb:AI669379 /FEA=EST /DB_XREF=gi:4834153 /DB_XREF=est:ty31g04.x1 /CLONE=IMAGE:2280726 /UG=Hs.178036 ESTs, Moderately similar to RL2B_HUMAN 60S RIBOSOMAL PROTEIN L23A H.sapiens	AI669379	HLA-F antisense RNA 1	HLA-F-AS1	285830	NM_001003807 /// NR_026972 /// NR_026973	0006955 // immune response // inferred from electronic annotation /// 0019882 // antigen processing and presentation // inferred from electronic annotation	0016020 // membrane // inferred from electronic annotation	

266_s_at	L33930	CD24	Homo sapiens	6-Oct-14	Exemplar sequence	GenBank	L33930 /FEATURE= /DEFINITION=HUMCD24B Homo sapiens CD24 signal transducer mRNA, complete cds and 3' region	L33930	CD24 molecule	CD24	1E+08	NM_001291737 /// NM_001291738 /// NM_001291739 /// NM_013230 /// NR_117089 /// NR_117090 /// XM_005278329	0001666 // response to hypoxia // inferred from expression pattern /// 0001775 // cell activation // inferred from direct assay /// 0001959 // regulation of cytokine-mediated signaling pathway // inferred from sequence or structural similarity /// 0002237 // response to molecule of bacterial origin // inferred from sequence or structural similarity /// 0002768 // immune response-regulating cell surface receptor signaling pathway // inferred by curator /// 0007155 // cell adhesion // inferred from electronic annotation /// 0007204 // positive regulation of cytosolic calcium ion concentration // inferred from direct assay /// 0007411 // axon [illegible]	0005886 // plasma membrane // inferred from electronic annotation /// 0009986 // cell surface // inferred from direct assay /// 0016020 // membrane // inferred from direct assay /// 0031225 // anchored component of membrane // inferred from electronic annotation /// 0045121 // membrane raft // inferred from direct assay	0004871 // signal transducer activity // non-traceable author statement /// 0005515 // protein binding // inferred from physical interaction /// 0019901 // protein kinase binding // inferred from physical interaction /// 0030296 // protein tyrosine kinase activator activity // inferred from direct assay

33132_at	U37012	CPSF1	Homo sapiens	6-Oct-14	Consensus sequence	GenBank	Cluster Incl. U37012:Human cleavage and polyadenylation specificity factor mRNA, complete cds /cds=(51,4379) /gb=U37012 /gi=1045573 /ug=Hs.83727 /len=4463	U37012	cleavage and polyadenylation specific factor 1, 160kDa /// microRNA 1234 /// microRNA 6849 /// microRNA 939	CPSF1 /// MIR1234 /// MIR6849 /// MIR939	29894 /// 100126351 /// 100302196 /// 102466749	NM_013291 /// NR_030635 /// NR_031600 /// NR_106908 /// XM_006716548 /// XM_006716549 /// XM_006716550	0000398 // mRNA splicing, via spliceosome // traceable author statement /// 0006366 // transcription from RNA polymerase II promoter // traceable author statement /// 0006369 // termination of RNA polymerase II transcription // traceable author statement /// 0006378 // mRNA polyadenylation // inferred from direct assay /// 0006379 // mRNA cleavage // inferred from direct assay /// 0006397 // mRNA processing // inferred from electronic annotation /// 0006406 // mRNA export from nucleus // traceable author statement /// 0008380 // RNA splicing // traceable author statement /// 0010467 // [illegible]	0005634 // nucleus // inferred from electronic annotation /// 0005654 // nucleoplasm // traceable author statement /// 0005847 // mRNA cleavage and polyadenylation specificity factor complex // inferred from direct assay	0003676 // nucleic acid binding // inferred from electronic annotation /// 0003723 // RNA binding // inferred from electronic annotation /// 0003730 // mRNA 3'-UTR binding // inferred from direct assay /// 0005515 // protein binding // inferred from physical interaction

35147_at	AB002360	MCF2L	Homo sapiens	6-Oct-14	Consensus sequence	GenBank	Cluster Incl. AB002360:Human mRNA for KIAA0362 gene, partial cds /cds=(0,3327) /gb=AB002360 /gi=2224664 /ug=Hs.25515 /len=5391	AB002360	MCF.2 cell line derived transforming sequence-like	MCF2L	23263	NM_001112732 /// NM_024979 /// XM_005268307 /// XM_005268308 /// XM_005268309 /// XM_005268310 /// XM_005268312 /// XM_005268313 /// XM_005268314 /// XM_005268315 /// XM_005268316 /// XM_006719966 /// XM_006719967 /// XM_006719968 /// XR_429282	0007264 // small GTPase mediated signal transduction // traceable author statement /// 0007266 // Rho protein signal transduction // inferred from electronic annotation /// 0032319 // regulation of Rho GTPase activity // inferred from electronic annotation /// 0032321 // positive regulation of Rho GTPase activity // inferred from electronic annotation /// 0035023 // regulation of Rho protein signal transduction // inferred from electronic annotation /// 0035556 // intracellular signal transduction // inferred from electronic annotation /// 0043065 // positive regulation of apoptotic process // traceable author [illegible]	0005615 // extracellular space // inferred from direct assay /// 0005737 // cytoplasm // inferred from electronic annotation /// 0005829 // cytosol // traceable author statement /// 0005886 // plasma membrane // inferred from electronic annotation /// 0016020 // membrane // inferred from electronic annotation /// 0030027 // lamellipodium // inferred from electronic annotation	0005085 // guanyl-nucleotide exchange factor activity // inferred from electronic annotation /// 0005089 // Rho guanyl-nucleotide exchange factor activity // inferred from electronic annotation /// 0005515 // protein binding // inferred from electronic annotation /// 0005543 // phospholipid binding // inferred from electronic annotation /// 0005545 // 1-phosphatidylinositol binding // inferred from electronic annotation

47069_at	AA533284	PRR5	Homo sapiens	6-Oct-14	Consensus sequence	GenBank	Cluster Incl. AA533284:nj66e12.s1 Homo sapiens cDNA /clone=IMAGE-997486 /gb=AA533284 /gi=2277380 /ug=Hs.79601 /len=613	AA533284	proline rich 5 (renal)	PRR5	55615	NM_001017528 /// NM_001017529 /// NM_001017530 /// NM_001198721 /// NM_015366 /// NM_181333	0001934 // positive regulation of protein phosphorylation // inferred from electronic annotation /// 0007049 // cell cycle // inferred from electronic annotation /// 0007165 // signal transduction // inferred from electronic annotation /// 0007264 // small GTPase mediated signal transduction // traceable author statement /// 0014068 // positive regulation of phosphatidylinositol 3-kinase signaling // inferred from electronic annotation /// 0030036 // actin cytoskeleton organization // inferred from direct assay /// 0030335 // positive regulation of cell migration // inferred from direct assay /// 0032321 [illegible]	0005622 // intracellular // inferred from electronic annotation /// 0005829 // cytosol // traceable author statement /// 0031932 // TORC2 complex // inferred from direct assay	0005096 // GTPase activator activity // inferred from electronic annotation /// 0005096 // GTPase activator activity // inferred from direct assay /// 0005100 // Rho GTPase activator activity // inferred from direct assay /// 0005515 // protein binding // inferred from physical interaction

55692_at	W22924	ELMO2	Homo sapiens	6-Oct-14	Consensus sequence	GenBank	Cluster Incl. W22924:75H3 Homo sapiens cDNA /clone=(not-directional) /gb=W22924 /gi=1299757 /ug=Hs.96560 /len=792	W22924	engulfment and cell motility 2	ELMO2	63916	NM_022086 /// NM_133171 /// NM_182764 /// XM_005260496 /// XM_005260497 /// XM_005260498 /// XM_005260499 /// XM_005260500 /// XM_005260501 /// XM_005260502 /// XM_006723854 /// XM_006723855	0006909 // phagocytosis // inferred from electronic annotation /// 0006915 // apoptotic process // inferred from electronic annotation /// 0038096 // Fc-gamma receptor signaling pathway involved in phagocytosis // traceable author statement /// 0045087 // innate immune response // traceable author statement /// 0060326 // cell chemotaxis // inferred from mutant phenotype	0005737 // cytoplasm // inferred from electronic annotation /// 0005829 // cytosol // inferred from direct assay /// 0005829 // cytosol // traceable author statement /// 0005856 // cytoskeleton // inferred from electronic annotation /// 0016020 // membrane // inferred from direct assay	0005488 // binding // inferred from electronic annotation /// 0005515 // protein binding // inferred from physical interaction /// 0005543 // phospholipid binding // inferred from electronic annotation /// 0017124 // SH3 domain binding // inferred from electronic annotation /// 0030971 // receptor tyrosine kinase binding // inferred from physical interaction
