# Supplementary material for: Association between Arsenic Level, Gene Expression in Asian Population, and In Vitro Carcinogenic Bladder Tumor
Source: Oxid Med Cell Longev. 2022 Jan 7;2022:3459855. doi: 10.1155/2022/3459855 (PMC8760535; doi:10.1155/2022/3459855)
Supplement: Supplementary 12 — Table S11: survival information of 18 genes differentially expressed between sex and common between ATO and arsenic exposed human. [file 3459855.f12.pdf]

Table S11. Survival information of 18 genes differentially expressed between sex and common between ATO and aersin exposed human

Genes	Survival
BTG2	Prognostic marker in breast cancer (favorable)
CD24	Prognostic marker in breast cancer (unfavorable), colorectal cancer (favorable) and liver cancer (unfavorable)
OLFM4	Prognostic marker in breast cancer (favorable)
BACE2	Prognostic marker in cervical cancer (unfavorable)
CXCR4	Prognostic marker in renal cancer (unfavorable), ovarian cancer (favorable) and stomach cancer (unfavorable)
PHACTR1	Prognostic marker in renal cancer (unfavorable)
CRIM1	Prognostic marker in renal cancer (favorable)
TNFRSF12A	Prognostic marker in renal cancer (unfavorable), head and neck cancer (unfavorable)
TSPAN5	Prognostic marker in renal cancer (unfavorable) and stomach cancer (unfavorable)
RGS	Prognostic marker in renal cancer (unfavorable) and stomach cancer (unfavorable)
PMAIP1	Prognostic marker in renal cancer (unfavorable)
SLC29A1	Prognostic marker in renal cancer (unfavorable)
DDIT4	Prognostic marker in renal cancer (unfavorable), pancreatic cancer (unfavorable) and head and neck cancer (unfavorable)
DDX3Y	Prognostic marker in head and neck cancer (favorable)
MYB	Prognostic marker in prostate cancer (favorable) and stomach cancer (favorable)
EGR1	Gene product is not prognostic
TRIB1	Gene product is not prognostic
SMAD5	Gene product is not prognostic
